
JOURNAL INFORMATION
==============================
NLM Title Abbreviation: JBMR Plus
Journal ID: JBMR Plus
NLM Journal ID: 101707013
Journal ID: jbmrplus

JBMR Plus

EISSN: 2473-4039
Publisher: Oxford University Press

ARTICLE INFORMATION
==============================
PMCID: PMC12092079
DOI: 10.1093/jbmrpl/ziaf082
Article ID: ziaf082
Article version: 1
Subjects: Abstracts, AcademicSubjects/MED00010, AcademicSubjects/MED00160, AcademicSubjects/MED00250

Abstracts of the ECTS 2025 Congress

Publication date: 2025 May
Electronic publication date: 2025 May 21
Volume: 9
Issue: Suppl 2
First page: ii1
Last page: ii329
Copyright: © The Author(s) 2025. Published by Oxford University Press on behalf of the American Society for Bone and Mineral Research.
Copyright year: 2025
License: This is an Open Access article distributed under the terms of the Creative Commons Attribution License (https://creativecommons.org/licenses/by/4.0/), which permits unrestricted reuse, distribution, and reproduction in any medium, provided the original work is properly cited.
License URL: https://creativecommons.org/licenses/by/4.0/

Page count: 1

==============================

### Abstracts of the ECTS 2025 Congress

### ECTS 2025 Congress

52nd European Calcified Tissue Society Congress

23 – 26 May 2025

**Scientific Programme Committee**

**Chair:**
Peter Pietschmann (Vienna, Austria)

**Co-Chairs:**
Bo Abrahamsen (Holbæk, Denmark)
**Clinical Co-Chair:**
Lorenz Hofbauer (Dresden, Germany)
**Basic Co-Chair:**
Cristina Sobacchi (Rozzano, Italy)
**Local Organizing Committee (LOC)-Chair:**
Christian Muschitz (Vienna, Austria)

**Members:**
Amina Valjevac (Sarajevo, Bosnia & Herzegovina)
Andrea Burden (Zurich, Switzerland)
Drenka Trivanovic (Belgrade, Serbia) – ECTS Academy
Barbara Hauser (London, United Kingdom)
Pawel Szulc (Lyon, France)

**International Associate Members:**
Nathalie Sims (Fitzroy, Australia)
Sundeep Khosla (Rochester, USA)

**Local Organising Committee:**
Christian Muschitz (LOC Chair)
Astrid Fahrleitner-Pammer (Graz, Austria)
Barbara Obermayer-Pietsch (Graz, Austria)
Rudolf Gasser (Innsbruck, Austria)
Sonja Gamsjäger (Vienna, Austria)

**Abstract Review Panel**
Each abstract was scored blind.

Bo Abrahamsen (Denmark)
Giovanni Adami (Italy)
Nerea Alonso (Austria)
Athanasios Anastasilakis (Greece)
Christina Andreasen (Denmark)
Natasha Appelman-Dijkstra (Netherlands)
Korhan Baklacı (Turkey)
Ulrike Baschant (Germany)
Duncan Bassett (United Kingdom)
Giulia Battafarano (Italy)
Zhanna Belaya (Russia C.I.S.)
Teresita Bellido (United States)
Francis Berenbaum (France)
Clemens Bergwitz (United States)
Claudine Blin (France)
Jean-Jacques Body (Belgium)
Jens Bollerslev (Norway)
Annemieke Boot (Netherlands)
Aline Bozec (Germany)
Nathalie Bravenboer (Netherlands)
Ludmila Brunerova (Czech Republic)
Andrea Burden (Switzerland)
Björn Busse (Germany)
Natalie C Butterfield (United Kingdom)
M Leonor Cancela (Portugal)
Geert Carmeliet (Belgium)
Mara Carsote (Romania)
Antonino Catalano (Italy)
Roland Chapurlat (France)
Chantal Chenu (United Kingdom)
Gavin Clunie (United Kingdom)
Martine Cohen-Solal (France)
Graziana Colaianni (Italy)
Luciano Colangelo (Italy)
Juliet Compston (United Kingdom)
Stella D'oronzo (Italy)
Bess Dawson-Hughes (United States)
Teun De Vries (Netherlands)
Judit Donáth (Hungary)
Eleni Douni (Greece)
Patricia Ducy (United States)
Claire Edwards (United Kingdom)
Florent Elefteriou (United States)
Ari Elson (Israel)
Karol Estrada (United States)
Vincent Everts (Netherlands)

Colin Farquharson (United Kingdom)
Verena Fischer (Germany)
Lars Folkestad (Denmark)
Antonella Forlino (Italy)
Melissa M Formosa (Malta)
Seiji Fukumoto (Japan)
Thomas Funck-Brentano (France)
Sonja Gamsjaeger (Austria)
Rudolf Gasser (Austria)
Kaare Gautvik (Norway)
Valerie Geoffroy (France)
Jeroen Geurts (Switzerland)
Fernando Gianfrancesco (Italy)
Alanna Green (United Kingdom)
Jenny Gregory (United Kingdom)
Jill Griffin (United Kingdom)
Agi Grigoriadis (United Kingdom)
Daniel Grinberg (Spain)
Bettina Groetsch (Germany)
Núria Guañabens (Spain)
Peyman Hadji (Germany)
Melanie Haffner-Luntzer (Germany)
Morten Svarer Hansen (Denmark)
Barbara Hauser (United Kingdom)
Eric Hay (France)
Eric Hesse (Germany)
Lorenz Hofbauer (Germany)
Ingunn Holen (United Kingdom)
Carmen Huesa (United Kingdom)
Anita Ignatius (Germany)
Yuuki Imai (Japan)
Jelena Jadzic (Serbia)
Abbas Jafari (Denmark)
Katharina Jähn-Rickert (Germany)
Franz Jakob (Germany)
Hervé Kempf (France)
Uwe Kornak (Germany)
Marie Helene Lafage Proust (France)
Laurence Legeai-Mallet (France)
Willem Lems (Netherlands)
Ulf Lerner (Sweden)
Thomas Levin Andersen (Denmark)
Paul Lips (Netherlands)
Yajun Liu (China)
Christa Maes (Belgium)
Outi Mäkitie (Finland)
Polyzois Makras (Greece)
Radmila Matijevic (Serbia)

Antonio Maurizi (Italy)
Sandro Mazzaferro (Italy)
Laura McCabe (United States)
Michelle Mcdonald (Australia)
Carolina Medina-Gomez (Netherlands)
Ciro Menale (Italy)
Daniela Merlotti (Italy)
Petar Milovanovic (Serbia)
Salvatore Minisola (Italy)
Barbara Misof (Austria)
Geert Mortier (Belgium)
Ralph Müller (Switzerland)
Anda Mihaela Naciu (Italy)
Amy Naylor (United Kingdom)
Riko Nishimura (Japan)
George Nuki (United Kingdom)
Barbara Obermayer-Pietsch (Austria)
Isabel Orriss (United Kingdom)
Eric Orwoll (United States)
Julien Paccou (France)
Biagio Palmisano (Italy)
Eleftherios Paschalis (Austria)
Olivier Peyruchaud (France)
Peter Pietschmann (Austria)
Richard Pikner (Czech Republic)
Marco Ponzetti (Denmark)
Sylvain Provot (France)
Adalbert Raimann (Austria)
Stuart Ralston (United Kingdom)
Alexander Rauch (Denmark)
Martina Rauner (Germany)
David Reid (United Kingdom)
Lars Rejnmark (Denmark)
Mara Riminucci (Italy)
Ilaria Roato (Italy)
Pamela Gehron Robey (United States)
Tim Rolvien (Germany)
Michela Rossi (Italy)
Antonio Rossi (Italy)
Katrine Hass Rubin (Denmark)
Kenneth Saag (United States)
Syazrah Salam (United Kingdom)
Martin Schepelmann (Austria)
Thorsten Schinke (Germany)
Katharina Schmidt-Bleek (Germany)
Federica Scotto di Carlo (Italy)
Karan Shah (United Kingdom)
Heide Siggelkow (Germany)

Natalie Sims (Australia)
Iulia Simona Soare (Romania)
Cristina Sobacchi (Italy)
Kent Søe (Denmark)
Anne Sophie Sølling (Denmark)
Nicole Sommer (Austria)
Antonia Sophocleous (Cyprus)
Katherine Staines (United Kingdom)
Jakob Starup-Linde (Denmark)
Steve Stegen (Belgium)
Gudrun Stenbeck (United Kingdom)
Pawel Szulc (France)
Hanna Taipaleenmaki (Germany)
Michaela Tencerova (Czech Republic)
Anna Maria Teti (Italy)
Jonathan Tobias (United Kingdom)
Drenka Trivanovic (Serbia)
Elena Tsourdi (Germany)
Jan Tuckermann (Germany)
Mustafa Unal (Turkey)
Maria Teresa Valenti (Italy)
Jeroen van de Peppel (Netherlands)
Bram van der Eerden (Netherlands)
Marjolein Van Driel (Netherlands)
Wim Van Hul (Belgium)
Dorien van Looy (Belgium)
Charlotte Verroken (Belgium)
Laurence Vico (France)
Anna Villa (Italy)
Felix Nikolai von Brackel (Germany)
Ning Wang (United Kingdom)
Ling Wang (China)
Elizabeth Winter (Netherlands)
Weibo Xia (China)
Maria Yavropoulou (Greece)
Timur Yorgan (Germany)
Vit Zikan (Czech Republic)
Carola Zillikens (Netherlands)
Philippe Zysset (Switzerland)

**Poster number abbreviations:**

PLOxx – Plenary Oral Presentation
COPxx – Concurrent Oral Presentation
Pxx – Poster
NDxx – New Data Poster

**Plenary Oral Presentations 1: Bone Health and Energy Metabolism**

**PLO01**

**Specific overexpression of 11ß-HSD1 as a model of local glucocorticoid action**

_Heidi Lunze_[1], *Ann-Kathrin Eiers*[2], *Mirko Peitzsch*[3], *Sabine Vettorazzi*[2], *Jan Tuckermann*[2], *Lorenz C. Hofbauer*[4], *Martina Rauner*[4], *Holger Henneicke*[4], *Ulrike Baschant*[4]
[1]*Medical Faculty and University Hospital Carl Gustav Carus- University of Technology Dresden, Department of Medicine III & Center for Healthy Aging, Dresden, Germany*
[2]*Ulm University, Comparative Molecular Endocrinology, Ulm, Germany*
[3]*Medical Faculty and University Hospital Carl Gustav Carus- Dresden University of Technology- Dresden, Clinical Chemistry and Laboratory Medicine, Dresden, Germany*
[4]*Medical Faculty and University Hospital Carl Gustav Carus- Dresden University of Technology- Dresden, Department of Medicine III & Center for Healthy Aging, Dresden, Germany*

**Abstract Text**

Glucocorticoids (GCs) are commonly used to treat inflammatory bone diseases like rheumatoid arthritis and osteoarthritis. However, their use can lead to adverse effects such as changes in glucose and bone metabolism, contributing to conditions like diabetes and osteoporosis. While physiological GC levels are important for proper bone function, controlling GC action in specific cell types could provide anti-inflammatory benefits while minimizing side effects.

We developed a mouse model with cell-type-specific overexpression of 11β-HSD1, an enzyme that activates GC receptor signaling. 11β-HSD1 overexpressing mice were crossed with Rosa26ERT2Cre and Osterix-Cre mice for ubiquitous and osteoprogenitor-specific GC activation. Serum corticosterone levels were measured by LC/MS, and 11β-HSD1 overexpression was confirmed at gene and protein levels by qPCR and Western blot. Bone metabolism was evaluated using µCT and histological analysis.

Serum corticosterone levels were elevated (5-fold, p=0.05) in Cre+ 11β-HSD1$^{Rosa26ERT2Cre}$ mice compared to controls. Similar increases were observed in 11β-HSD1$^{OsxCre}$ mice (5-fold, p<0.01). The highest 11β-HSD1 expression was found in the liver of 11β-HSD1$^{Rosa26ERT2Cre}$ mice (7-fold, p=0.01). Additionally, overexpression of 11ß-HSD1 was also detected at protein level in osteoblasts.

Evaluation of trabecular bone showed significant changes only in female femora in 11β-HSD1$^{Rosa26ERT2Cre}$ mice, with a 70% reduction in bone mass (p<0.05) and a 50% decrease in bone mineral density (p<0.05). In contrast, constitutive osteoprogenitor-specific overexpression of 11β-HSD1 in OsxCre mice led to a 2-fold increase in trabecular femoral BV/TV in males (p<0.05) and a 27% increase in trabeculae numbers in both sexes (p<0.05), indicating anabolic effects of local steroid production. Cortical thickness decreased by 12% (p<0.05). Initial findings also showed a significant decrease (12%) in cortical thickness in homozygous OsxCre mice ($p$<0.05).

These results suggest that 11β-HSD1 overexpression can activate local GC production without additional GC treatment. Future studies will focus on whether cell-type-specific GC activation can reduce bone inflammation while maintaining bone homeostasis.

**PLO02**

**Fracture risk and treatment thresholds in patients with diabetes**

*Jakob Starup-Linde[1], Katrine Hygum[1], Henrik Støvring[2], Claus Gravholt[1], Jens-Erik Beck Jensen[3], Pia Eiken[4], Pernille Hermann[5], Bente Langdahl[1], Torben Harsløf[1]*
*[1]Aarhus University Hospital, Department of Endocrinology, Aarhus, Denmark*
*[2]Aarhus University Hospital, Steno Diabetes Center Aarhus, Aarhus, Denmark*
*[3]Hvidovre Hospital, Department of Endocrinology, Hvidovre, Denmark*
*[4]Bispebjerg Hospital, Department of Endocrinology, Copenhagen, Denmark*
*[5]Odense University Hospital, Department of Endocrinology, Odense, Denmark*

**Abstract Text**

**Background:** Type 1 diabetes (T1D) and type 2 diabetes (T2D) are associated with an increased fracture risk. A bone mineral density (BMD) T-score threshold of -2.5 for initiation of anti-osteoporotic therapy is commonly used, but this may not be applicable to individuals with Purpose or T2D.

**Aim:** In this population-based case-control study we aimed to determine T-score thresholds for T1D and T2D with equivalent risk of fractures as that of individuals without diabetes and a T-score of -2.5.

**Methods:** We collected dual energy x-ray absorptiometry (DXA) data (2000-2019), information on diagnoses (1977-2019) and redemption of prescribed medications (1997-2019) from the National Danish Registries which are linked by a unique identifier. Cases were individuals with the first incident major osteoporotic fracture (MOF) within two years before or one year after a DXA and controls were fracture free and matched on age, gender, and period of the DXA. Logistic regression modelling was used in the case-control analysis.

**Results:** We identified 17,703 cases and 17,703 controls. T1D and T2D were associated with an increased risk of MOF (odds ratio: 1.8, 95 % CI:1.4;2.3 and 1.2, 95 % CI:1.1;1.3, respectively ($p<0.05$ for all)) adjusted for hip BMD T-score. T1D and T2D patients had a similar risk of MOF at -1.4 and -2.1 T-score, respectively, as individuals without diabetes with a T-score of -2.5. For hip fracture, the equivalent risk was correspondingly reached with T-scores of -1.9 and -1.6 (Figure 1). Similar findings were observed for femoral neck and lumbar spine BMD T-scores.

Conclusions: Compared to those without diabetes, we found a markedly increased fracture risk in patients with T1D and T2D independent of BMD. Our study suggests thresholds for treatment initiation in T1D and T2D should be increased to T-scores of -1.6 and -1.8 at the hip, femoral neck, and lumbar spine, respectively.

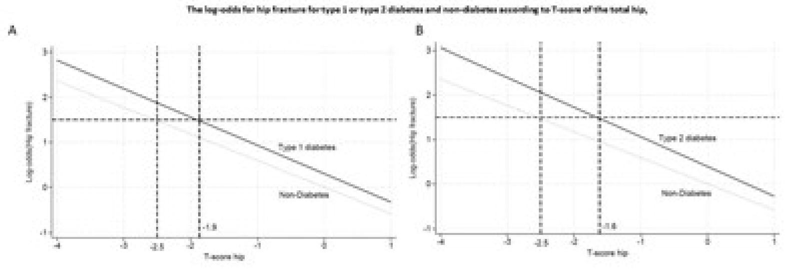

**PLO03**

**High fat diet differently affects bone and energy metabolism in obesity-prone C57Bl/6J and obesity-resistant A/J male mice**

*<u>Dung K. Nguyen</u>[1], Michaela Ferencakova[1], Martina Dzubanova[1], Andrea Benova[1], Elena Golovina[1], Jan Prochazka[2], Frantisek Spoutil[3], Tomas Cajka[4], Marek Vrbacky[5], Kristina Bardova[6], Martin Rossmeisl[6], Jan Kopecky[6], Michaela Tencerova[7]*

*[1]Institute of Physiology of the Czech Academy of Sciences, Laboratory of Molecular Physiology of Bone, Prague, Czech Republic*

*[2]Institute of Molecular Genetics of the Czech Academy of Sciences Prague, Czech Centre for Phenogenomics & Laboratory of Transgenic Models of Diseases, Prague, Czech Republic*

*[3]Institute of Molecular Genetics of the Czech Academy of Sciences, Czech Centre for Phenogenomics & Laboratory of Transgenic Models of Diseases, Prague, Czech Republic*

*[4]Institute of Physiology of the CzechAcademy of Sciences, Laboratory of Translational Metabolomics, Prague, Czech Republic*

*[5]Institute of Physiology of the Czech Academy ofSciences, Laboratory of Bioenergetics, Prague, Czech Republic*

*[6]Institute of Physiology of the CzechAcademy of Sciences, Laboratory of Adipose Tissue Biology, Prague, Czech Republic*

*[7]Institute of Physiology of the CzechAcademy of Sciences, Laboratory of Molecular Physiology of Bone, Prague, Czech Republic*

**Abstract Text**

**Background:** Obesity-associated bone fractures are not manifested in all obese individuals. However, the underlying mechanism behind this phenomenon has not been well described. Thus, we investigated the effect of obesity on bone and bone marrow stromal stem cell (BMSCs) metabolism in obesity-prone C57BL/6J (B6) mice and obesity-resistant A/J mice under obesogenic condition.

**Methods:** Eight-week-old B6 and A/J male mice were either fed with normal diet (ND) or high-fat-diet (HFD) (60 % kcal from fat) (n=10/group) for up to 5 months and examined for metabolic and bone parameters. Primary BMSCs were obtained from treated mice for cellular analyses. Proteomic analysis of bone marrow (BM) was performed using LC-MS.

**Results:** After 3 and 5 months of HFD, B6 mice gained body weight (+1.5-fold, p<0.001) and impaired glucose tolerance (+1.3-fold, p<0.05) comparing to A/J mice. HFD impaired trabecular and cortical bone in B6 mice, which was more pronounced after 5 months than 3 months. In constrast to B6, HFD A/J mice did not manifest trabecular bone loss (p>0.10) and increased cortical thickness and decreased porosity between 3 and 5 months (p<0.001). These obesity-induced changes were accompanied by lower bone marrow adiposity in A/J vs B6 mice (p<0.01). Further, primary BMSCs isolated from A/J mice manifested higher glycolytic profile compared to respiratory profile in B6 BMSCs (p<0.001) suggesting a higher osteogenic potential of A/J BMSCs under HFD. Proteomic analysis revealed increased levels of antioxidant enzymes (e.g. GLO1, CAR3) in A/J compared to B6 mice.

**Conclusion:** Our data show that obesity induced worse impact on bone and fat metabolism in B6 compared to A/J mice associated with lower BMSC glycolytic capacity in B6 mice. These changes were accompanied by increased levels of antioxidative enzymes in A/J HFD BM suggesting protection from HFD-induced oxidative stress. Further analyses are needed to uncover the mechanism behind this phenomenon.

**PLO04**
**The association between GLP-1 receptor agonists and fractures: an observational case-control study**

*Annika Kvist*[1], *Rikke Viggers*[1,2,3], *Peter Vestergaard*[1,2,3]
[1]*Region North Denmark, Steno Diabetes Center North Denmark, Aalborg, Denmark*
[2]*Aalborg University Hospital, Department of Clinical Medicine, Aalborg, Denmark*
[3]*Aalborg University Hospital, Department of Endocrionology, Aalborg, Denmark*

**Abstract Text**

**Background and aims:** GLP-1 receptor agonists (GLP-1RA) was introduced as a treatment of type 2 diabetes (T2D) with a side effect of weight loss. Since, the medications have been approved as a weight loss medication. Rapid, greater weight loss can result in significant bone loss, resulting in an increased fracture risk.

**Methods:** We identified persons with a fracture in 2017-2021 aged 30-90 years and matched them 1:2 with controls (without any fractures) on sex and year of birth. Logistic regression models were used to calculate odds ratios for any fracture event and major osteoporotic fracture (MOF) events with GLP-1RA as the exposure. Furthermore, analyses were adjusted by risk factors.

**Results:** 337,713 persons with a fracture (controls) and 675,426 controls were identified. In the unadjusted analyses there were no difference in the use of GLP-1RA between persons with and without fractures (any fracture OR [CI]: 1.027 [0.991-1.065], MOF OR [CI]: 1.023 [0.973-1.076]). In the adjusted analyses, GLP-1RA were used less in the group that sustained any fracture (OR [CI]: 0.950 [0.914-0.988]) or MOF (OR [CI]: 0.845 [0.801-0.891]) compared to the controls.

**Conclusions:** Fracture risk might be reduced using GLP-1RA.

**PLO05**

**The Local Role of Antidiabetic Medications Liraglutide, Canagliflozin, and Metformin on BMAT and Bone in Knee OA: Insights from an Ex Vivo Model**

*Léa Loisay[1], Léonie Reinert[1], Alexander Antoniadis[2], Thibaut Royon[2], Maja Kaegi[2], Elke Moradpour[1], Thomas Hügle[1], Jeroen Geurts[1]*
*[1]Lausanne University Hospital, Department of Rheumatology, Lausanne, Switzerland*
*[2]Lausanne University Hospital, Department of Orthopaedics and Traumatology, Lausanne, Switzerland*

**Abstract Text**

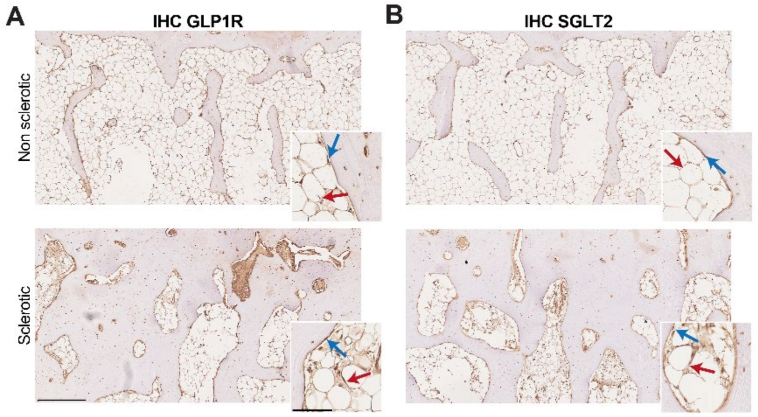

Figure 1: Localization of GLP1R and SGLT2 by IHC.
A. Representative images of GLP1R IHC in NS and SC samples; B. Representative images of SGLT2 in NS and SC samples
In low magnification (scale bar: 500 μm) and high magnification (scale bar: 100 μm )
In high magnification images, blue arrows point at osteoblast and red arrows point at BMAd.

**Background:** Bone marrow adipose tissue (BMAT) is now recognized for its role in bone remodeling under high energy demands, as seen in osteoarthritis (OA). With the increasing prevalence of OA risk factors, obesity and diabetes, targeting adipocyte metabolism with antidiabetic drugs like metformin, canagliflozin (SGLT2 inhibitor), and liraglutide (GLP1 receptor (GLP1R) agonist) can present novel treatment options in OA. Recent studies suggest these drugs alleviate OA symptoms. However, the effects on BMAT and bone remodeling remain unclear.

**Purpose:** Here, by repurposing antidiabetic drugs metformin, canagliflozin, and liraglutide, we investigated the effects of these drugs on BMAT and bone remodeling in knee OA.

**Methods:** Non-sclerotic (NS) and sclerotic (SC) tissues were collected from patients (n=12) undergoing knee replacement. Expression of GLP1R and SGLT2 was assessed by IHC. NS and SC bone explants were treated for 7 days with either metformin, liraglutide, canagliflozin, or vehicle. BMAT lipolysis products (FFA and glycerol) and bone formation (pro-collagen type I and ALP) were measured by ELISA or colorimetric assay.

**Results:** GLP1R and SGLT2 were expressed in both adipocytes and osteoblasts (Figure 1A and 1B). Treatment did not change biomarkers in NS samples. However, in SC samples, metformin and liraglutide modulated lipolysis by reducing FFA (-38%, p=0.0003 and -35%, p=0.014) and glycerol levels (-48%, p=0.009 and -32%, p=0.078), but not canagliflozin. Bone markers were only significantly modulated by canagliflozin (pro-collagen type I: -30%, p=0.0475). Interestingly, the change in FFA levels with metformin positively correlated with the change of ALP activity (r=0.6154, p=0.0373).

**Conclusion:** This study offers new insights into the effects of antidiabetic drugs in OA subchondral bone, highlighting their ability to inhibit lipolysis and modulate bone activity under high-energy demand. Future approaches combining metformin and canagliflozin may alleviate local symptoms of OA by modifying pathological bone formation but need to be verified in long-term studies.

**Plenary Oral Presentations 2: Emerging Therapies**

**PLO07**

**The iron regulator Tfr2 influences the development of heterotopic ossification**

*<u>Sven Spangenberg</u>[1], Maja Vujić Spasić[2], Sandro Altamura[3], Anupam Sinha[4], Lorenz C. Hofbauer[1], Martina Rauner[1], Ulrike Baschant[1]*
*[1]Dresden University of Technology, Department of Medicine III & Center for Healthy Aging, Dresden, Germany*
*[2]University of Ulm, Institute of Comparative Molecular Endocrinology, Ulm, Germany*
*[3]University of Heidelberg, Department of Pediatric Oncology- Hematology and Immunology, Heidelberg, Germany*
*[4]Dresden University of Technology, Department of Medicine III, Dresden, Germany*

**Abstract Text**

**Background:** Heterotopic ossification (HO) is bone formation at abnormal anatomical sites with crucial involvement of the bone morphogenetic protein (BMP) signaling pathway. Recently, we identified the iron regulator transferrin receptor 2 (Tfr2) as a novel binding partner for BMPs and BMPR thereby exerting extra-hepatic effects on bone formation.

**Purpose:** To investigate the influence of Tfr2 *in vivo*. We compared the effects of Tfr2 on HO to other iron regulators (hereditary hemochromatosis protein (Hfe) and Ferroportin (Fpn))

**Methods:** HO was induced by injecting BMP-2 into the M. tibialis anterior of iron-overloaded $Tfr2^{-/-}$, $Hfe^{-/-}$ and $Fpn^{C326S}$ mutant mice, $Tfr2^{OsxCre}$ mice and their littermate controls. Micro-CT, iron and histological measurements were performed at day 14. The inflammatory phase was examined by flow cytometry and bulk RNA sequencing (day 3).

**Results:** $Tfr2^{-/-}$ mice showed more severe HO reflected by enhanced areas of infiltrating immune cells, chondrocytes and ossified tissue (3.6-fold; p<0.001). Flow cytometry analysis confirmed an increase in pro-inflammatory macrophages at day 3 (59.14% vs 36.40% of CD45+ cells; p<0.01).
GO analysis of M. tibialis anterior revealed an upregulation of pro-osteogenic Wnt, NOTCH and Hedgehog signaling in $Tfr2^{-/-}$ mice, the latter one being implicated in HO development by forming a macrophage-supported microenvironment. Tfr2-deficiency in Osx+ cells did not alter iron levels but

HO was increased by 1.9-fold ($p<0.05$).
Similar to Tfr2$^{-/-}$ mice, Hfe$^{-/-}$ mice also developed more HO (1.40 vs 0.72 mm$^3$ BV; p=0.06). Contrarily, mice with a mutation in Fpn rendering them severely iron-overloaded (76-fold in liver; $p<0.01$), tended to show less HO formation (0.30 vs 1.20 mm$^3$ BV; p=0.15).

**Conclusion:** Tfr2 plays a cell-intrinsic role in the development of HO, most likely in osteoprogenitor cells via pro-osteogenic signaling pathways such as Hedgehog signaling. Opposing findings in Hfe$^{-/-}$ and Fpn mutant mice demonstrate the iron-independent effects of iron regulatory proteins on HO progression.

**PLO08**

**Palopegteriparatide Improves Skeletal Dynamics in Adults With Chronic Hypoparathyroidism: 162-week Results From the Phase 2 PaTH Forward Trial**

*Elena Tsourdi[1], Mishaela R. Rubin[2], Aliya A. Khan[3], Peter Schwarz[4], Intekhab Ahmed[5], Andrea Palermo[6], Filomena Cetani[7], Uberto Pagotto[8], Rajesh Jain[9], Carol Zhao[10], Michael Ominsky[11], Bryant Lai[11], Jenny Ukena[11], Aimee D. Shu[11], Lars Rejnmark[12]*
*[1]Technische Universität Dresden, Department of Medicine III, Dresden, Germany*
*[2]Columbia University, Clinic, New York- NY, United States*
*[3]McMaster University, Clinic, Hamilton- ON, Canada*
*[4]Rigshospitalet, Center for Kræft og Organsygdomme, Copenhagen, Denmark*
*[5]Thomas Jefferson University, Endocrinology & Metabolism, Philadelphia- PA, United States*
*[6]Campus Bio-medico University, Unit of Metabolic Bone and Thyroid Disorders, Rom, Italy*
*[7]University Hospital of Pisa, Endocrine Unit, Pisa, Italy*
*[8]IRCCS Azienda Ospedaliero-Universitaria di Bologna,*
*Division of Endocrinology and Diabetes Prevention and Care, Bologna, Italy*
*[9]University of Chicago, Department of Medicine, Chicago- IL, United States*
*[10]Ascendis Pharma Inc, Global Biometrics, Palo Alto- CA, United States*
*[11]Ascendis Pharma Inc, Clinical Development, Palo Alto- CA, United States*
*[12]Aarhus University Hospital, Department of Endocrinology and Internal Medicine, Aarhus N, Denmark*

**Abstract Text**

**Background**: Chronic hypoparathyroidism is associated with low bone remodeling that predisposes to increased bone mineral density (BMD) due to the missing physiological effects of parathyroid hormone (PTH). Palopegteriparatide (YORVIPATH®; TransCon PTH) is approved by the European Commission as a PTH replacement therapy for adults with chronic hypoparathyroidism. This analysis investigated skeletal endpoints in response to treatment with palopegteriparatide through week 162 of the PaTH Forward trial.

**Methods**: PaTH Forward is a phase 2, randomized, double-blind, placebo-controlled 4-week trial followed by an ongoing open-label extension through week 266. Serum bone turnover markers procollagen type 1 N-terminal propeptide (P1NP) and C-terminal telopeptide of type 1 collagen (CTx) and BMD measured by DXA at the lumbar spine, total hip, femoral neck, and 1/3 distal radius were assessed.

**Results**: Fifty-seven of 59 (97%) participants remained in the trial and received palopegteriparatide treatment through week 162. At week 162, 52 (91%) participants achieved independence from conventional therapy (≤600 mg/day elemental calcium and no active vitamin D); mean serum calcium was maintained in the normal range (8.3-10.6 mg/dL) from baseline through week 162. With exposure to palopegteriparatide, mean CTx and P1NP initially increased, peaked by week 26, declined thereafter, and remained stable above baseline levels through week 162 (Figure). With palopegteriparatide treatment, mean BMD Z-scores declined from elevated baseline levels characteristic of chronic hypoparathyroidism toward age and sex-matched norms and remained above zero through week 162 (Fig). Trends in BMD changes were consistent regardless of age, sex, menopausal status, and duration of hypoparathyroidism. Higher baseline BMD Z-scores were associated with greater declines in BMD over time. No new safety signals were identified.

Skeletal Responses to Palopegteriparatide

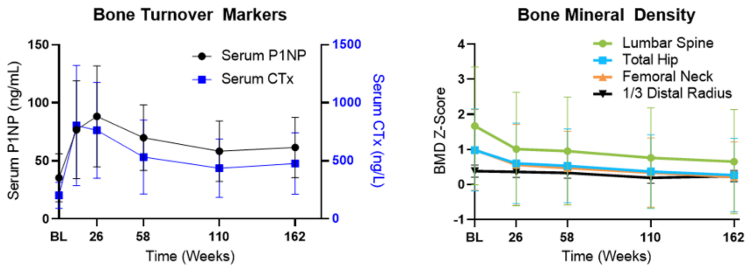

Means ± SD at each time point are plotted.

**Conclusions**: These 162-week results show that continued PTH replacement therapy with palopegteriparatide sustains temporal changes trending toward a new skeletal steady state closer to age-appropriate norms in adults with chronic hypoparathyroidism.

**PLO09**

**Bone pain in Fibrous dysplasia does not rely on aberrant sensory nerve sprouting or neuroma formation**

*Biagio Palmisano[1], Chiara Tavanti[2], Farinacci Giorgia[2], Giorgio Gosti[3], Marco Leonetti[3], Samantha Donsante[2], Giuseppe Giannicola[4], Natasha Appelman-Dijkstra[5], Alessandro Corsi[2], Ernesto Ippolito[6], Mara Riminucci[2]*
*[1]Sapienza University, Department of Molecular Medicine, Roma, Italy*
*[2]Sapienza University, Department of Molecular Medicine, Rome, Italy*
*[3]Italian National Research Council, Center for Life Nano- & Neuro-Science, Rome, Italy*
*[4]Sapienza University,*
*Department of Anatomical- Histological- Medico Legal and Orthopaedic Sciences, Rome, Italy*
*[5]Leiden University Medical Centre, Internal Medicine- Division of Endocrinology, Leiden, Netherlands*
*[6]Tor Vergata University, Department of Orthopaedic Surgery, Rome, Italy*

**Abstract Text**

Bone pain is a major symptom of many skeletal disorders. Fibrous dysplasia (FD) is a genetic disease due to activating mutations of the stimulatory G protein α-subunit (Gαs). Bone pain in FD associates with skeletal deformities and fractures or nerve impingement by the pathological tissue. However, FD patients often complain of a pain that does not correlate with their disease burden. Although multiple hypotheses have been made, the pathogenetic mechanisms of FD pain remain largely unexplored.

In this study, we first investigated pain behavior in a mouse model of FD (EF1α-GαsR201C). Using the burrowing (BT), nesting (NT), open field (OFT) and tail flick (TFT) tests we showed that female and male FD mice at all ages developed a pain-like behavior (unpaired t-test, WT vs FD: BT $p<0.0001$; NT: $p<0.0001$; OFT: $p<0.05$) and delayed response to nociceptive stimuli (TFT: $p<0.05$). Interestingly, as in FD patients, pain behavior did not correlate with mouse disease burden. Then, by generating a triple transgenic model of FD expressing GFP in nerve fibers, we showed that the skeletal innervation pattern was preserved in FD mice and within bone lesions, linear sensory nerve fibers were detected in the fibrotic marrow by immunofluorescence (Figure 1). Finally, by analyzing bone biopsies from FD patients, we provided the first evidence that sensory nerve fibers were rarely detected within the pathological human tissue. Interestingly, higher gene expression of *BDNF* and *NGF* neurotrophins was found in FD compared to healthy donors' (HD) samples (Mann-Whitney test, HD vs FD: *BDNF* p=0.08; *NGF* p=0.43).

These data demonstrate that bone pain is an intrinsic and reproducible feature of FD. They also show that, albeit sensory nerve fibers are found within FD lesions and may contribute to the unpleasant sensation that accompanies the disease, pathological sensory nerve sprouting or neuroma formation are not detected in the Gαs-mutated skeleton.

**Figure 1**

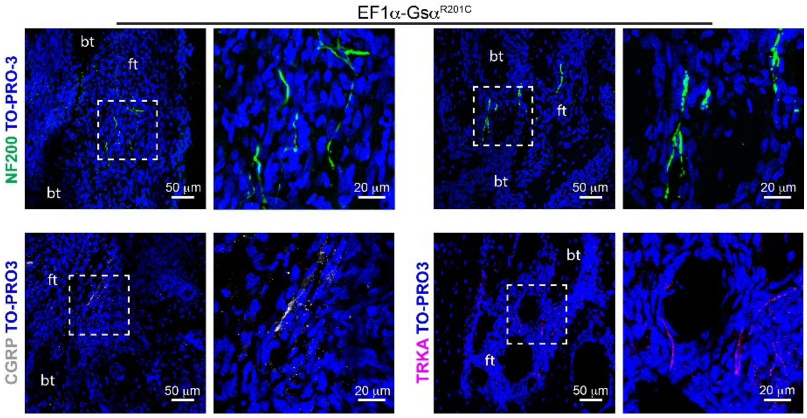

**PLO10**

**A randomized controlled trial comparing the efficacy of denosumab, teriparatide, or combination of denosumab and teriparatide on bone microarchitecture in patients being initiated on glucocorticoids**

*Durairaj Arjunan*[1], *Jayaditya Ghosh*[1], *Sadam Hussain*[1], *Mani Sangar*[1], *Vandana Dhiman*[1], *Rimesh Pal*[1], *Rama Walia*[1], *Vishali Gupta*[2], *Sanjay Bhadada*[1]

[1]PGIMER, Endocrinology, Chandigarh, India
[2]PGIMER, Ophthalmology, Chandigarh, India

**Abstract Text**

**Background**: Glucocorticoid-induced osteoporosis (GIO) is a common complication in patients on long-term steroid therapy. This randomized controlled trial evaluates the efficacy of teriparatide, denosumab, or combination of teriparatide with denosumab in the prevention of GIO.

**Methods**: Patients with uveitis who were started on oral prednisolone (>5 mg prednisolone for > 3 months) were included. They were randomized into three groups: Group A (denosumab), Group B (teriparatide), and Group C (Teriparatide+Denosumab). All participants underwent baseline bone turnover markers, DXA, Trabecular Bone Score and HR-pQCT and were followed up every six months for one year.

**Results**: 60 participants were enrolled, and 41 patients completed 1 year of follow-up. The mean age was 42.5 ± 11.3 years with a male-to-female ratio 1:2. The cumulative prednisolone dose was 5.2 grams. At 1 year of follow-up, Group B exhibited higher levels of P1NP and CTX than the other two groups. Group B showed significantly higher BMD at the lumbar spine and total hip compared to Groups A and C. HR-pQCT revealed more favorable bone microarchitecture in Group B (Table).

**Conclusion**: Teriparatide demonstrated superior efficacy in preserving BMD and bone microarchitecture compared to denosumab or combining both drugs in patients with long-term glucocorticoids. The findings indicate that teriparatide effectively counteracts the deleterious effects of glucocorticoids on bone health, maintaining favorable bone microarchitecture.

**Table: Parameters at one year**

| Parameter | Group A(n=13) | Group B(n=14) | Group C(n=14) | p-value |
|---|---|---|---|---|
| LS-BMD(g/cm$^2$) | 0.871±0.096 | 0.993±0.085 | 0.856±0.070 | .016$^{*\#}$ |
| TH-BMD(g/cm$^2$) | 0.794±0.048 | 0.943±0.129 | 0.871±0.092 | .043$^{*\#}$ |
| TBS | 1.353±0.054 | 1.385±0.054 | 1.258±0.058 | .021$^{*\#}$ |
| CTX(pg/ml) | 42.9±20.6 | 516.8±313.0 | 36.4±33.7 | .038$^{*\#}$ |
| P1NP(ng/ml) | 26.0±13.1 | 103.4±57.4 | 27.8±14.4 | .011$^{*\#}$ |
| Total.vBMD[mg HA/ccm] | 225.7±50.2 | 317.5±36.0 | 254.2±39.8 | .001$^{*\#\$}$ |
| Trabecular vBMD[mg HA/ccm] | 116.0±31.8 | 136.4±45.0 | 98.9±11.3 | .099 |
| Cortical vBMD[mg HA/ccm] | 779.3±94.1 | 898.3±66.4 | 875.6±62.6 | .030$^{*\#\$}$ |
| Cortical.thickness[mm] | 0.816±0.224 | 1.110±0.186 | 0.919±0.138 | .016$^{*\#\$}$ |
| Trabecular.Number [1/mm] | 1.238±0.362 | 1.443±0.256 | 1.219±0.222 | .028$^{*\#}$ |
| Trabecular.Separation [mm] | 0.878±0.290 | 0.694±0.14 | 0.821±0.158 | .035$^{*\#}$ |

*** A vs B**
**# B vs C**
**$ A vs C**

**PLO11**

**Oxr1 Regulates Osteoclast Function Through Keap1-P62 Signaling: From Molecular Mechanism to Therapeutic Repurposing**

*Xiaojun Chen*[1], *Xiangyun Jin*[2], *Killugudi Swaminatha Iyer*[3], *Nathan J. Pavlos*[1], *Haibo Jiang*[4], *Kai Chen*[1]
[1]*The University of Western Australia, School of Biomedical Sciences, Perth, Australia*
[2]*Shanghai Jiao Tong University, Renji Hospital- School of Medicine, Shanghai, China*
[3]*The University of Western Australia, School of Molecular Sciences, Perth, Australia*
[4]*The University of Hong Kong, Department of Chemistry, HongKong, China*

**Abstract Text**

**Background:** Osteoclasts, specialized bone-resorbing cells, are essential for regulating skeletal homeostasis. Abnormal osteoclast activity contributes to severe bone disorders.

**Purpose:** To better understand osteoclast-specific molecules for developing more selective therapeutic interventions.

**Methods:** Public scRNA-seq data mining identified Oxr1 as a key regulator of osteoclastogenesis. Immunostainings mapped its spatial expression in the mice bone microenvironment. These findings were further validated through qPCR and Western Blotting assays. To investigate regulatory mechanisms of Oxr1, we used siRNA-mediated knockdown of Oxr1, followed by bulk RNA-seq, bone resorption assays, and electron microscopy (EM). Mechanistically, co-immunoprecipitation (CO-IP) and surface plasmon resonance (SPR) assays were performed to identify proteins interacting with Oxr1. High-throughput screening of FDA-approved drugs against Oxr1 domains was performed to identify repurposable therapeutics. Animal procedures were approved by the AEEC-WMU (wydw 2023-0619).

**Results:** Immunostainings revealed that Oxr1 is specifically expressed in osteoclasts (Fig.A). Oxr1 protein levels peaked at Day 5 ($p < 0.0001$). Interestingly, Oxr1 deletion significantly reduced resorbed area per osteoclast to 0.05% ($p < 0.001$, Fig.B). EM showed disrupted mitochondrial integrity, while bulk RNA-seq analysis (GSEA) indicated downregulation of oxidative phosphorylation (NES = -2.71, $p < 0.001$) and autophagy pathways. EM results indicated the accumulations of autophagosomes following Oxr1 knockdown (Fig.B). CO-IP and SPR assays demonstrated that Oxr1 binds directly to Keap1 (Fig.C), a key mitophagy mediator that promotes P62-dependent mitochondrial ubiquitination. TRAP staining showed the P62-mediated mitophagy inducer (PMI) effectively rescued the phenotype induced by Oxr1 knockdown ($p < 0.05$, $n = 4$, Fig.D). Additionally, Velpatasvir was identified as a direct Oxr1 binder (KD = 156 nM) and significantly increased bone volume fraction, reaching 6.7 ± 1.2% ($p < 0.01$, $n = 6$) in ovariectomized mice (Fig.E).

**Conclusion:** Oxr1 facilitates Keap1-dependent mitochondrial ubiquitination for promoting autophagy to support osteoclastogenesis, thereby representing a potential therapeutic target for osteoclast-associated bone disorders.

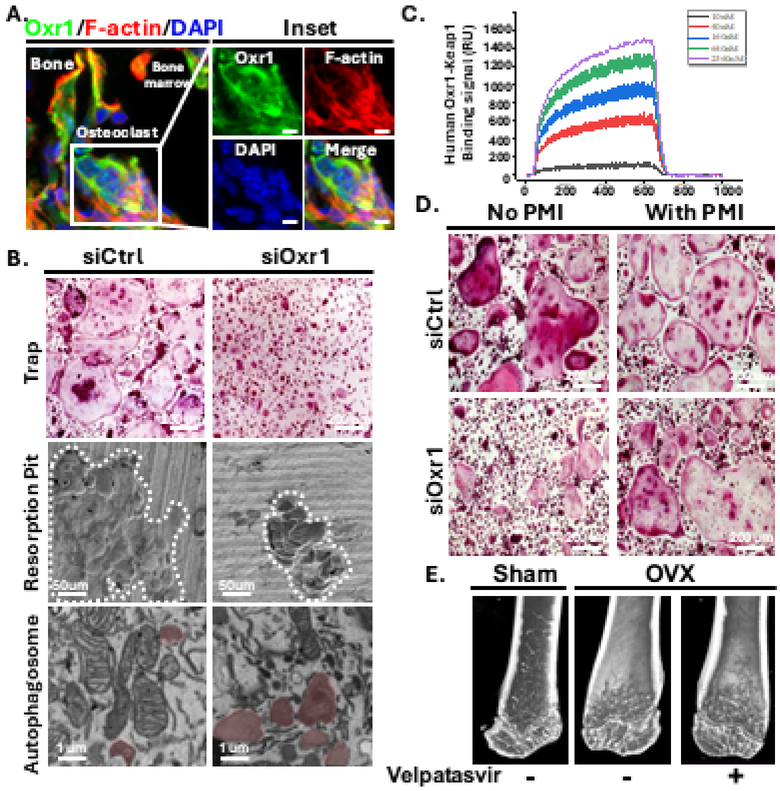

**PLO12**

**Evidence-Based Recommendations for the Management of Osteoporosis in Systemic Mastocytosis: A Systematic Review and Meta-Analysis**

*Giovanni Adami*[1], *Francesca Pistillo*[1], *Gaia Tabacco*[2], *Tiago Rama*[3], *Jens Peter Panse*[4], *Christof Wilke*[5], *Willem Lems*[6], *Siggelkow Heide*[5]

[1]*University of Verona, Rheumatology Unit, Verona, Italy*

[2]*Policlinico Universitario Campus Bio-Medico, UOC Patologie osteo-metaboliche e della tiroide, Rome, Italy*

[3]*Faculdade de Medicina da Universidade do Porto, Allergology and Immunology, Porto, Portugal*

[4]*University RWTH Medical School, Department of Hematology, Aachen, Germany*

[5]*University of Göttingen, Department of Gastroenterology and Endocrinology, Göttingen, Germany*

[6]*VU University medical centre, Rheumatology Unit, Amsterdam, Netherlands*

**Abstract Text**

**Background:** Osteoporosis and fractures are common in systemic mastocytosis (SM), but guidelines on their management are lacking. To address this, we conducted a systematic literature review (SLR) and meta-analysis to provide evidence-based recommendations on behalf of the European Calcified Tissue Society (ECTS).

**Methods:** We retrieved over 8000 records, narrowing to 84 studies that met inclusion criteria according to 8 PICOs covering all aspects of bone health in adult mastocytosis. A random-effects meta-analysis was performed to determine pooled prevalence rates of osteoporosis, osteopetrosis, osteolytic lesions, bone pain, and fractures. We developed recommendations based on the evidence, voting on the level of agreement for each. Risk of bias was assessed using a ROBIN table.

**Results:** Osteoporosis prevalence was 26% (95% CI 21-31%) **figure 1**, osteopetrosis 7% (95% CI 5-8%), osteolytic lesions 2% (95% CI 1-3%), bone pain 32% (95% CI 16-49%), and fractures 29% (95% CI 20-37%). High heterogeneity ($I^2$>70%) was observed. ROBINS table were produced and showed on average low-quality evidence. Sixteen recommendations were eventually drafted and voted by the task force, including routine DXA for osteoporosis screening and vertebral fracture assessment (strong recommendation), additional screening for osteosclerotic and lytic lesions with spine and hip X-rays (conditional), and regular DXA follow-ups due to accelerated bone loss. Calcium, vitamin D supplementation, and multidisciplinary management were strongly recommended. Treatment with bisphosphonates was favored as first-line therapy, while denosumab was recommended for specific conditions. Exploratory use of teriparatide, romosozumab and avapritinib was discussed for severe cases or refractory symptoms. Agreement was overall high.

**Conclusion:** This comprehensive analysis highlights the substantial burden of bone disease in SM and provides a robust framework for screening and management. Further research is needed to refine treatment strategies and explore novel therapeutic options. These recommendations aim to improve outcomes through early identification and targeted interventions for osteoporosis in mastocytosis.

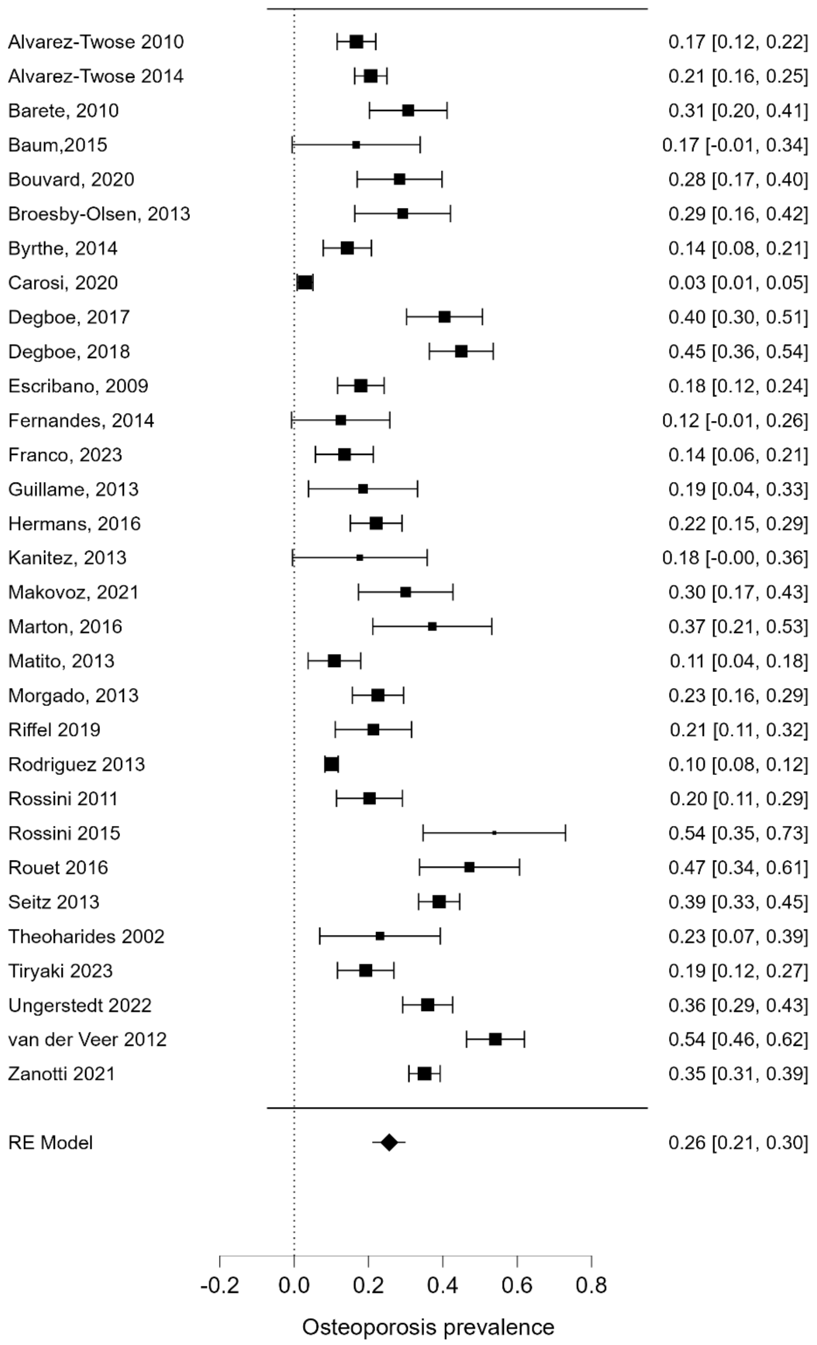

### Plenary Oral Presentations 3: All Paths lead to Bone

### PLO13

### Angiogenesis is uncoupled from osteogenesis during skull bone regeneration

*<u>Gabriele Bixel</u>[1], Kishor K. Sivaraj[1], Melanie Timmen[2], Vishal Mohanakrishnan[1], Anusha Aravamudhan[1], Bong-Ihn Koh[1], Hyun-Woo Jeong[1], Kai Kruse[1], Richard Stange[2], Ralf Adams[1]*

[1]Max Planck Institute for Molecular Biomedicine, Tissue Morphogenesis, Muenster, Germany
[2]Institute of Musculoskeletal Medicine, Regenerative Musculoskeletal Medicine, Muenster, Germany

**Abstract Text**

**Background:** Bone regeneration requires a well-orchestrated cellular and molecular response including robust vascularization and recruitment of mesenchymal and osteogenic cells. In femoral fractures, angiogenesis and osteogenesis are closely coupled during the complex healing process.

Purpose: To understand the role of the vasculature in skull bone healing after lesion injury.

Methods: Skull bone healing after drill hole injury was imaged by longitudinal intravital two-photon microscopy in adult transgenic mice expressing *Flk1-GFP, sp7-mCherry* mice (n=5), endothelial Notch GOF and LOF mice (n=8 per group, ethic-app: 81-02.04.2018.A171). Single cell profiling was used to investigate expression of genes involved in bone healing and angiogenesis.

Results: Here, we show using advanced longitudinal intravital multiphoton microscopy that early vascular sprouting is not directly coupled to osteoprogenitor invasion during calvarial bone regeneration. Early osteoprogenitors emerging from the periosteum give rise to bone-forming osteoblasts at the injured calvarial edge. Microvessels growing within the lesions are not associated with osteoprogenitors. Subsequently, osteogenic cells collectively invade the vascularized and perfused lesion as a multicellular layer and promote regenerative ossification. Vascular sprouting and remodeling result in dynamic changes in blood flow to accommodate the growing bone. Single cell profiling of injured skull bone shows mesenchymal stromal cell heterogeneity comparable to femoral fractures with an increase in cell types that promote bone regeneration. Expression of angiogenesis and hypoxia-related genes is slightly increased, reflecting ossification of a vascularized lesion site. Endothelial Notch alters vascular sprouting and regeneration in skull bone repair without affecting ossification.

Conclusions: After lesion injury of the skull, the vasculature initially grows alone without the co-migrating osteoprogenitors that prepare the injured bone tissue for subsequent ossification. This type of bone healing is therefore fundamentally different from long bone healing after fracture injury. Our findings have clinical implications for bone regeneration and therapies that target the vascularity of healing bone.

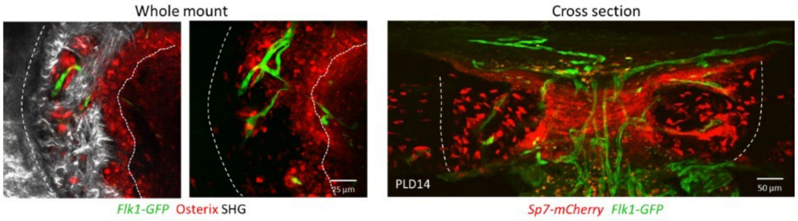

Figure 1: Two-photon microscopy of healing skull bone at post-lesion day 14 (PLD14) shows new bone matrix (white) formed by a multicellular layer of osteoblasts and progenitor cells (red) that collectively invade the already vascularized lesion site (Bixel et al. 2024, Nat. Comm. 15, 4575).

**PLO14**

### Prevalence of normal, low and very low BMD in Artificial Intelligence (AI) -identified vertebral fractures (VF).

*Muhammad Kassim Javaid*[1], *Tiago Santos*[2], *Madeleine Sampson*[3], *Anosh Rasool*[3], *Mark Baxter*[4], *Charlotte Toogood*[5], *Elizabeth Curtis*[6]
[1]*University of Oxford, NDORMS, Headington, United Kingdom*
[2]*University Hospital Southampton NHS Foundation Trust, Medicine for Older People, Southampton, United Kingdom*
[3]*University Hospital Southampton NHS Foundation Trust, Radiology, Southampton, United Kingdom*
[4]*Hospital Southampton NHS Foundation Trust, Medicine for Older People, Southampton, United Kingdom*
[5]*University Hospital Southampton NHS Foundation Trust, Fracture Liaison Service, Southampton, United Kingdom*
[6]*University of Southampton- Southampton General Hospital, MRC Lifecourse Epidemiology Centre, Southampton, United Kingdom*

**Abstract Text**

**Background:** AI-models effectively identify patients with VF from existing CT scans. Access to anabolic osteoporosis therapy is often dependent on the lowest T score but the proportion of these adults with low and very low BMD is unknown.

**Purpose:** To describe BMD in women and men aged over 50 years with AI-identified VF.

**Methods:** Consecutive CT scans in adults aged 50 years plus were analyzed using the Nanox HealthVCF algorithm. AI flagged scans were then clinically confirmed and patients ≤ 75 years were referred to DXA scanning. Older patients were referred if they had multiple fractures. Logistic regression was used to predict BMD categories ≤-2.5 or ≤-3.5.

**Results:** From 01/08/23 - 04/12/2024, 11,092 CT scans were sent for AI analysis. Of the 3,741 scans flagged by the AI with potential VF, 1,336 were clinically confirmed as moderate or severe VF. Of the first 218 referred and completed a DXA, 82/218 (37.6%) had a lowest T score ≤-2.5 and 13/218 (6.0%) had a T score of ≤-3.5. 36/127 (28.4%) of men with clinically confirmed VF had a T score ≤-2.5 and 3.9% of men a T score ≤-3.5%. A third of women and 41.2% of men had a lowest BMD between -2.4 and -1 and 16.5% and 30% had a T score > -1 respectively (Figure). Age at time of CT scan did not predict BMD categories (p=0.1).

**Conclusion:** Approximately half of women and 30% of men with a confirmed AI-identified VF had a T score of ≤-2.5. A significant proportion of older adults with moderate/ severe vertebral fractures had normal BMD and further work is required to establish their treatment needs.

**Figure**
Relationship between Age at time of CT scan and Lowest T score in clinically confirmed moderate/

severe vertebral fracture identified using AI

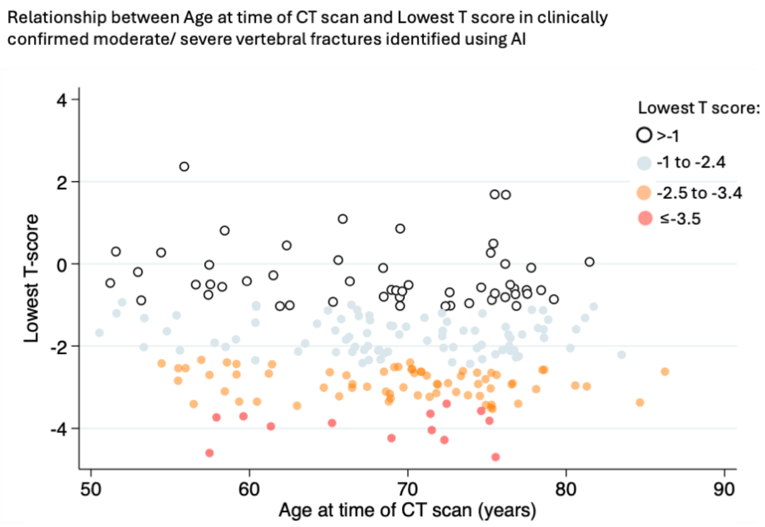

**PLO15**

### Discovery of core genes for osteoarthritis using the omnigenic model and zebrafish validation

*Rory Stewart[1], Andrii Iakovliev[1], Fraser Collins[1], Athina(A.Spiliopoulou@ed.ac.uk) Spiliopoulou[1], Cosimo De Bari[1], Paul McKeigue[2], Erika Kague[1]*

*[1]Institute of Genetics and Cancer, College of Medicine and Veterinary Medicine, Edinburgh, United Kingdom*

*[2]Usher Institute, College of Medicine and Veterinary Medicine, Edinburgh, United Kingdom*

### Abstract Text

Osteoarthritis is a debilitating joint disease, affecting over 500 million people worldwide with no disease-modifying drugs are currently available. Genome-Wide Association Studies (GWAS) have identified over 100 loci associated with OA. However, GWAS identify genes with small effect sizes and have not led to therapeutic breakthroughs. A new genetic model, the omnigenic model, postulates that peripheral genes with small effect sizes coalesce on expression of a sparse set of core genes that have a direct impact on phenotype, thereby providing attractive therapeutic targets.

We aimed to identify OA core genes using the omnigenic model and supported by functional studies in zebrafish. We performed Genome-wide aggregated trans-effects (GATE) analysis on 25,013 hip-OA cases and 459,105 controls from UK Biobank. Trans-scores were computed by aggregating the effects of multiple trans-eQTLs (eQTLGen Phase 1) from plasma proteins across datasets, including 35,559 Icelanders (DeCODE), 54,306 participants (UK Biobank), 3,301 (INTERVAL study), 8,293 Finns, and 3,394 participants (IMPROVE study). Associations between trans-scores and OA were analysed using summary GWAS statistics.

GATE analysis identified 14 putative core genes. Core genes clustered into distinct pathways implicated in OA, including bone/cartilage development, neuronal signalling, and inflammation. We used single cell transcriptomic data from human and mice joints to prioritise genes for functional validation in zebrafish. Zebrafish orthologs of core genes; *CHAD* (LogOR=-0.034, P=$1\times10^{-6}$), *SORT1* (LogOR=0.029, P=$2\times10^{-5}$), *SPARCL1* (LogOR=0.045, P=$2\times10^{-10}$), and *UCMA* (LogOR=0.032, P=$1\times10^{-6}$) were targeted using CRISPR/CAS9 to study gene function during joint development. Zebrafish G0 crispants carrying mosaic mutations were analysed at 5 days-post-fertilisation using cartilage staining and linear measurements in ImageJ.

Our data suggests that mutations in core genes result in altered cartilage element and joint morphology (Figure).

*Figure: A) Key measurements taken from alcian blue imaging. B) Imaging from alcian blue protocol, 5 days post-fertilisation zebrafish control (wild-type) and crispants. C) Measurement data taken from controls and crispants. i) Ratio of average big length to average small length. ii) Average big width.*

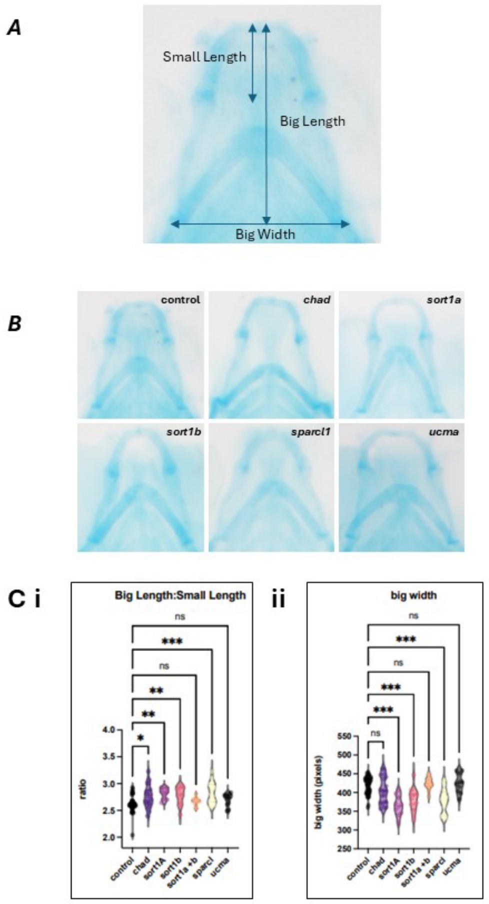

In conclusion, our study identified distinct core genes in OA, whose changes may lead to in significant impacts on joint health, offering potential targets for future therapeutic interventions.

**PLO16**

### MRI-Based Muscle Status Analysis: An AI-based Approach to Sarcopenia Assessment Using the UK Biobank

*Hyeon Su Kim[1], Dong-Won Byun[2], Deog-Yoon Kim[3], Jun-Il Yoo[4]*
*[1]Inha University Hospital, Orthopedics Surgery, Incheon, Korea- South*
*[2]Soonchunhyang University Hospital,*
*Division of Endocrinology and Metabolism- Department of Internal Medicine, Seoul, Korea- South*
*[3]Kyung Hee University School of Medicine, Department of Nuclear Medicine, Seoul, Korea- South*
*[4]Inha University Hospital, Orhopedics Surgery, Incheon, Korea- South*

### Abstract Text

**Introduction:** Sarcopenia, defined by a progressive decline in skeletal muscle mass and function, is a significant health risk in older adults, leading to frailty and increased mortality. Dual-energy X-ray absorptiometry (DEXA) is widely used to measure skeletal muscle mass but lacks the ability to assess muscle quality and distribution in detail. Magnetic resonance imaging (MRI) offers superior soft tissue contrast, enabling precise muscle segmentation and volume quantification.

**Purpose:** This study aims to develop an MRI-based segmentation model for accurate quantification of individual thigh muscle volumes and to explore its relationship with DEXA-derived skeletal muscle index (SMI) and sarcopenia diagnosis. The research seeks to address the limitations of DEXA and enhance sarcopenia evaluation and management in clinical settings.

**Methods:** Whole-body MRI scans using the DIXON technique from 6,657 participants aged 65 and older from the UK Biobank imaging study were analyzed. A UNETR (U-net Transformer) model was trained on 30 cases to segment 27 thigh muscles categorized into Anterior, Posterior, Medial, and Gluteal groups. Segmentation accuracy was evaluated using the Dice similarity coefficient. MRI-derived muscle volumes were compared with DEXA-based SMI and sarcopenia diagnoses, and statistical analyses were conducted to assess their relationships.

**Results:** The UNETR model achieved an average Dice score of 0.91 across all muscle regions, demonstrating high segmentation accuracy. MRI-derived muscle volumes strongly correlated with DEXA-based SMI (**$p < 0.001$**). Sarcopenic individuals had significantly lower muscle volumes across all regions compared to non-sarcopenic individuals (**$p < 0.001$**). Anterior thigh muscles showed the strongest association with sarcopenia diagnosis (**$p < 0.001$**).

**Conclusion:** This study introduces a robust MRI-based segmentation model for evaluating thigh muscle volumes. MRI provides deeper insights into muscle quality and distribution, offering a complementary and superior method to DEXA for sarcopenia diagnosis, with potential for improved clinical applications.

**PLO17**

### AnnexinA1 binding to Dectin-1 regulates osteoclast differentiation

*Gülnaz Guliyeva[1], Anja Derer[2], Georg Schett[1], Aline Bozec[1], Bettina Groetsch[1]*
*[1]Uniklinikum Erlangen, Department of Medicine 3 - Rheumatology and Immunology, Erlangen, Germany*
*[2]Uniklinikum Erlangen, Strahlenklinik, Erlangen, Germany*

**Abstract Text**

Bone homeostasis relies on the removal of dead cells by macrophages and osteoclasts, with phagocytic recognition via phosphatidylserine bound to bridging molecules like AnnexinA1. Notably, increased cell death is linked to enhanced bone resorption by osteoclasts. The C-type lectin receptor Dectin-1, originally recognized as the primary β-glucan receptor involved in antibacterial tissue homeostasis, has recently been shown to bind further ligands such as annexins. Phosphorylation of the hemITAM motif downstream of Dectin-1 initiates a signaling cascade that plays a key role in osteoclast differentiation. Therefore, it is likely that apoptotic cells within the bone environment activate Dectin-1 on myeloid cells, influencing osteoclast differentiation and maintaining bone homeostasis in non-inflammatory conditions.

We first assessed the bone homeostatic state of Dectin-1 knock out mice (clec7a-/-) compared to littermate controls. *Clec7a-/-* mice showed an increased bone volume (BV/TV; 0.12 vs. 0.18 %; p=0.02) due to decreased osteoclast numbers (N.OC/B.Pm.;3.92 vs. 2.87 1/mm; p=0.0018). In vitro, ß-glucan ligand activation of Dectin-1 in wt pre-osteoclasts induced Dectin-1 expression (0.0003 vs. 0.002 relative expression to actin p=0.02) and the number of polynucleated osteoclasts (30.6 vs. 52.1 OC >5nuclei; p=0.002). The β-glucan effect on osteoclasts was Dectin-1 dependent, as osteoclasts derived from clec7a-/- mice did not respond to β-glucan stimulation. Next, to examine whether apoptotic cells express AnnexinA1 and influence osteoclast differentiation, we exposed wild-type splenocytes to g-irradiation. A 4gray dose of g-irradiation caused a time-dependent increase in AnnexinA1 expression. Moreover, irradiated splenocytes induced the number of polynucleated osteoclasts (30.8 vs. 50.1 OC>5nuclei) that was Dectin-1 dependent.

Our data suggest that AnnexinA1 expressed on apoptotic cells binds to Dectin-1 on pre-osteoclasts to regulate osteoclast differentiation and bone resorption under non-inflammatory conditions. The aim of our research is to uncover the molecular mechanism of AnnexinA1 induced Dectin-1 signaling on osteoclast activation and its implications in bone remodeling.

**PLO18**

**Association of varying annualized body mass index changes with the risk of rapid annualized total hip bone mineral density loss in adults over 50**

*Benjamin Bakke Hansen[1], Sören Möller[1], Bente Langdahl[2,3], Niklas Rye Jørgensen[4,5], Peter Vestergaard[6,7], Bryan Haddock[8], Lars Folkestad[9], Uffe Kock Wiil[10], Katrine Hass Rubin[1], Bo Abrahamsen[1,11]*
*[1]Research Unit OPEN - Odense University Hospital,*
*Department of Clinical Research - University of Southern Denmark, Odense, Denmark*
*[2]Aarhus University, Department of Clinical Medicine, Aarhus, Denmark*
*[3]Aarhus University Hospital, Department of Endocrinology, Aarhus, Denmark*

[4]Rigshospitalet, Department of Clinical Biochemistry, Copenhagen, Denmark
[5]Faculty of Health and Medical Sciences, Department of Clinical Medicine - University of Copenhagen, Copenhagen, Denmark
[6]Aalborg University Hospital, Steno Diabetes Center North Denmark, Aalborg, Denmark
[7]Aalborg University Hospital, Department of Endocrinology and Clinical Medicine, Aalborg, Denmark
[8]Rigshospitalet, Department of Clinical Physiology and Nuclear Medicine, Glostrup, Denmark
[9]Odense University Hospital, Department of Endocrinology, Odense, Denmark
[10]University of Southern Denmark, Health Informatics and Technology- Maersk Mc-Kinney Moller Institute, Odense, Denmark
[11]Holbæk Hospital, Department of Medicine 1, Holbæk, Denmark

**Abstract Text**

**Background:** Body mass index (BMI) is positively correlated with bone mineral density (BMD), through larger mechanical loading on weight-bearing bones. The association between annualized BMI changes (ΔBMI) on annualized BMD change (ΔBMD) remains unclear and complex.

**Purpose:** Assess how annualized ΔBMI impacts the risk of losing at least 5% total hip BMD annually.

**Methods:** Danish routine-care DXA-scans (2010–2023) for individuals aged 50+, enriched with health register data on prescriptions and I.V. bisphosphonates. Mixed-effects logistic regression (MELR) was used to account for repeated DXA-scans and individual-level clustering.

**Outcome:** >5% Annualized total hip ΔBMD loss.

**Exposure:** Annualized ΔBMI.

**Results:** MELR included 402,035 DXA-scans from 133,574 individuals, 79% women aged 69 ± 8 years.

Proportions of outcome, increased as ΔBMI deviated from stable ΔBMI levels (Table 1). Men had significantly elevated odds-ratio (OR) compared to women at positive or near zero ΔBMI. (Table 1).

There is a U-shaped association in the mean conditional probability of the outcome across ΔBMI levels (Figure 1).

| | *No. DXA Scans* | | *% Outcome Observed* | | *Contrast in OR [95CI]*[ab] |
|---|---|---|---|---|---|
| **Annualized ΔBMI (kg/m$^2$)** | **Men** | **Women** | **Men** | **Women** | |
| **< -3ΔBMI** | 179 | 692 | 28.5% | 27.0% | 0.95 [0.53-1.71] |
| **-3 to -2ΔBMI** | 271 | 1452 | 14.0% | 15.1% | 1.10 [0.62-1.96] |
| **-2 to -1ΔBMI** | 1370 | 7123 | 5.8% | 7.4% | 1.20 [0.84-1.73] |
| **-1 to -0.5ΔBMI** | 2922 | 15165 | 2.9% | 3.1% | 0.92 [0.65-1.30] |
| **±0.5 ΔBMI** | 30380 | 175420 | 1.4% | 1.0% | 0.70 [0.59-0.82]* |
| **+0.5 to +1ΔBMI** | 3382 | 18071 | 2.0% | 1.4% | 0.63 [0.42-0.93]* |

| | | | | | |
|---|---|---|---|---|---|
| **+1 to +2ΔBMI** | 1776 | 7840 | 3.9% | 2.4% | 0.57 [0.38-0.86]* |
| **+2 to +3ΔBMI** | 358 | 1486 | 7.8% | 3.0% | 0.29 [0.14-0.60]* |
| **> +3ΔBMI** | 183 | 612 | 13.7% | 5.6% | 0.33 [0.14-0.77]* |

**p < 0.05*

[a]**Reference category:** *Men. Bonferroni-adjusted*

[b]**Adjusted:** *Age,AOM & GC Exposure,Absolute BMI,T-score (Total Hip)*

**Figure**

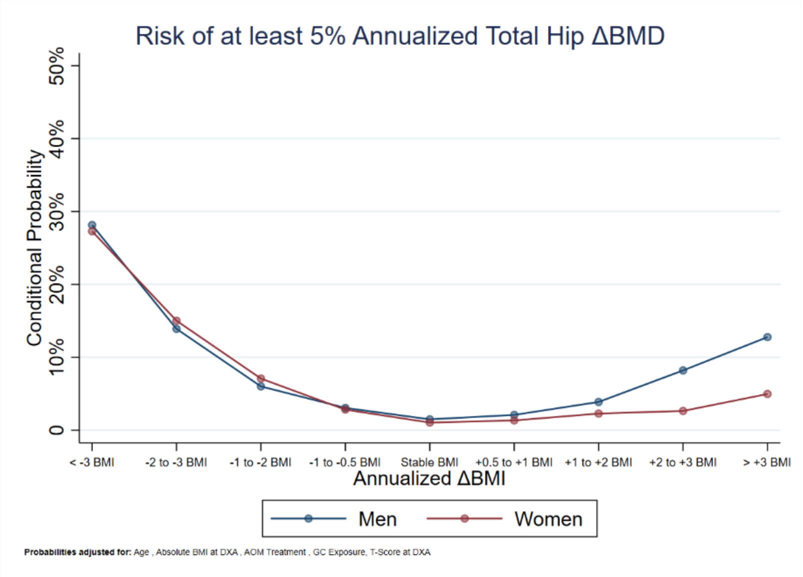

**Conclusion:** A U-shaped association exists between annual ΔBMI and experiencing >5% hip BMD loss annually, particularly at negative ΔBMI. Men appear at higher risk at large ΔBMI gains, compared to women, though absolute risks remains low except at the highest levels of ΔBMI.

### Concurrent Oral Presentations

### Concurrent Oral Presentations 1: Rare Bone Diseases - Basic Aspect

### COP01

### Bone phenotype in a novel mouse model of mild haploinsufficient osteogenesis imperfecta

*Laura Ventura*[1], *Lidiia Zhytnik*[1], *Anja Niehoff*[2], *Myrthe E. Hoogeland*[3], *Vivian de Waard*[3], *Astrid D. Bakker*[4], *Frank Zaucke*[5], *Elisabeth M.W. Eekhoff*[6], *Dimitra Micha*[1]
[1]*Amsterdam UMC, Human Genetics, Amsterdam, Netherlands*
[2]*German Sport University Cologne, Institute of Biomechanics and Orthopaedics, Köln, Germany*

[3]University of Amsterdam, Medical Biochemistry, Amsterdam, Netherlands
[4]Academic Centre for Dentistry Amsterdam, Oral Cell Biology, Amsterdam, Netherlands
[5]University Hospital Frankfurt, Dr. Rolf M. Schwiete Research Unit for Osteoarthritis, Frankfurt, Germany
[6]Amsterdam UMC, Endocrinology and Metabolism, Amsterdam, Netherlands

**Abstract Text**

**Background**: Osteogenesis Imperfecta (OI) is a genetic disorder causing bone fragility, with OI type 1 being the mildest form. It is caused by a deficiency of collagen type I due to *COL1A1* gene variants, leading to fractures. Despite representing >30% of OI patients, research has been limited by the lack of effective mouse models.

**Aim:** This study aimed to develop and characterize a new mouse model for mild haploinsufficient OI.

**Methods:** A mild haploinsufficient OI (hiOI) mouse model was created using CRISPR/Cas to induce a heterozygous deletion of the entire *Col1a1* allele in C57BL/6J mice. 20 hiOI and 20 wild-type (WT) mice (50% males) were analysed at 8 weeks of age. Markers for bone formation (P1NP) and resorption (TRAcP 5b) were measured in serum with ELISA. The expression of collagen type I chains in bone and additional bone markers was investigated using RT-qPCR. Bone RNA sequencing was performed ($n_{WT}$=5; $n_{hiOI}$=5; males). Bone analysis included microcomputed tomography (µCT, histology (Masson Goldner's trichrome; $n_{WT}$=9; $n_{hiOI}$=9; males), and 3-point bending test ($n_{WT}$=18, 50% males; $n_{hiOI}$=13, 69% males).

**Results**: A significantly lower *Col1a1*/*Col1a2* ratio (p≤0.0001) was found in hiOI mice, reflecting collagen deficiency. Increased expression of bone turnover markers highlighted the presence of a metabolic bone disorder. RNA sequencing revealed upregulation of 51 genes related to collagen biosynthesis and extracellular matrix formation. P1NP was significantly reduced (p≤0.001) and positively correlated with the *Col1a1/Col1a2* ratio (p=0.0088, r=0.4137). TRAcP was unaltered, indicating that osteoclast number was not impaired. µCT and histological analysis confirmed impaired bone structure in hiOI mice, and the 3-point bending test revealed reduced biomechanical properties, including stiffness, maximum force and moment of inertia (p≤0.05), maximum energy (p≤0.001), and maximum energy-stress-strain (p≤0.001).

**Conclusion:** The hiOI mouse model mimics the mild haploinsufficient form of OI, making it a valuable tool for advancing research and therapies.

**COP02**

**Modeling the KIF5B p.K91R Mutation: Insights into KIF5B-Associated Skeletal Dysplasia**

*Victoria Sarne[1,2], Stéphane Blouin[3], Richard Arnhold[4], Peter Nikkels[5], Winfried Neuhaus[6,7], Hannes Steinkellner[1], Thomas Dechat[8], Franco Laccone[1]*
[1]Medical University of Vienna, Institute of Medical Genetics, Vienna, Austria
[2]University of Vienna,
Vienna Doctoral School of Pharmaceutical- Nutritional and Sport Sciences PhaNuSpo-, Vienna, Austria

[3]Ludwig Boltzmann Institute of Osteology, Material Science, Vienna, Austria
[4]Danube Hospital, Pathologisch-Bakteriologisches Institut, Vienna, Austria
[5]Wilhelmina Children's Hospital, Department of Pathology, Utrecht, Netherlands
[6]AIT – Austrian Institute of Technology GmbH, Competence Unit Molecular Diagnostics, Vienna, Austria
[7]Danube Private University, Department of Medicine, Krems, Austria
[8]Ludwig Boltzmann Institute of Osteology, Skeletal Cryo-Biopsy, Vienna, Austria

**Abstract Text**

**Introduction:** *KIF5B* encodes Kinesin Family Member 5B, a motor protein crucial for intracellular transport. Although the underlying mechanisms for its involvement in skeletal development are unclear, recent evidence points to a potentially critical role for KIF5B in this process. A specific KIF5B mutation (p.K91R) was identified in two human patients with severe skeletal abnormalities (Itai et al., 2022). We now report a third case: a fetus with an unclassified skeletal dysplasia, further implicating KIF5B in skeletal development.

**Purpose:** To investigate the impact of the p.K91R mutation on skeletal development, we established a mouse model to study the *in vivo* effects on bone growth and structure. Understanding the role of KIF5B in skeletal development could provide crucial insights into the underlying mechanisms of skeletal dysplasia and inform potential therapeutic strategies for affected patients.

**Methods:** Micro-computed tomography (µCT) and growth plate histology were performed on p.K91R mutant mice and their wildtype littermates at P7, P28, and P84 to assess skeletal morphology and growth plate organization.

**Results:** p.K91R mutant mice exhibited significantly reduced body weights compared to wildtype littermates at P28 (8.636 g vs. 12.55 g, respectively; $p$<0.0001) and P84 (18.50 g vs. 22.83 g; $p$=0.0003). µCT analysis revealed shorter femoral lengths (P84: 12.01 mm vs. 14.74 mm; $p$<0.001) and reduced perimeters (P84: 4.6423 mm vs. 4.988 mm $p$=0.0116) persisting into adulthood, with notable differences in trabecular architecture at earlier stages. At P28, mutant mice displayed reduced trabecular number (2.211 $mm^{-1}$ vs. 3.160 $mm^{-1}$; $p$=0.0404) and increased spacing (0.3882 mm vs. 0.2558 mm; $p$=0.0263), which balanced out by P84. Growth plate histology of P7 mutant mice revealed a disorganized structure, closely resembling the abnormalities observed in the human fetal case.

**Conclusion:** The p.K91R mutation disrupts skeletal growth and long bone microarchitecture, producing a phenotype in mice that mirrors the skeletal dysplasia observed in humans.

**COP03**

**Skeletal manifestations and cross-correction after ex-vivo gene therapy in Mucopolysaccharidosis type I-Hurler**

*Ludovica Santi[1], Stefania Crippa[1], Valentina Capo[1], Evelyn Oliva Savoia[1], Gaia Alberti[1], Selene Ceriotti[1], Giulia Consiglieri[2], Francesca Tucci[2], Mara Riminucci[3], Alessandro Corsi[3], Andrea Barbero[4],*

*Ivan Martin[4], Francesca Sanvito[5], Rossana Norata[5], Martina Rocchi[5], Simona Bolamperti[6], Isabella Villa[6], Alessandro Aiuti[1], Anna Villa[1], Maria Ester Bernardo[1]*
*[1]IRCSS San Raffaele Scientific Institute, San Raffaele-Telethon Institute for Gene Therapy SR-Tiget, Milan, Italy*
*[2]IRCSS San Raffaele Scientific Institute,*
*Pediatric Immunohematology and Bone Marrow Transplantation Unit, Milan, Italy*
*[3]Sapienza University, Department of Molecular Medicine, Rome, Italy*
*[4]University Hospital Basel, Tissue Engineering Department of Biomedicine, Basel, Switzerland*
*[5]IRCCS San Raffaele Scientific Institute, GLP San Raffaele Telethon Institute for Gene Therapy, Milan, Italy*
*[6]IRCCS San Raffaele Scientific Institute, Osteoporosis and bone and mineral metabolism Unit, Milan, Italy*

**Abstract Text**

The severe and progressive dysostosis multiplex that affects Mucopolysaccharidosis type I-Hurler (MPSIH) patients, resulting in unmet clinical need, was stabilized in 8 patients treated in a phase I/II clinical trial (NCT03488394) with ex-vivo hematopoietic stem cell gene therapy (HSC-GT). As the molecular mechanisms underlying skeletal damage and cross-correction after HSC-GT are not fully elucidated, we investigated osteoclasts (OCs), mesenchymal stromal cells (MSCs), osteoblasts (OBs) and chondrocytes (CHs) of MPSIH patients enrolled in the trial before and after HSC-GT. The 2D in vitro characterization of patient-derived OCs and MSCs did not show any alterations as compared to healthy donors (HDs), although during the intramembranous ossification patient-derived OBs showed intracellular glycosaminoglycans accumulation. The in vitro scaffold-free 3D chondrogenic pellets (CHPs), developed to study the endochondral ossification, revealed a reduced expression of COLX and SOX9 in patient-derived samples compared to HDs, but normalization of the inflammatory TLR4 expression in samples after HSC-GT. The in vivo subcutaneous implantation of patient-derived CHPs into immunodeficient MPSI mice showed a reduced capability to develop bone ossicles demonstrated by incomplete formation of the marrow cavity, residual cartilaginous tissue and reduced expression of CD31 (Fig.1A). The histological analyses of the osteomedullary biopsies of MPSIH patients revealed vacuolation of the enthesis tissue which was completely rescued after HSC-GT (Fig.1B). Moreover, a reduction of bone trabecular tissue coupled with expansion of bone marrow adipose tissue was found in samples after HSC-GT, indicating the need for further monitoring. A deep analysis of the skeletal disease and correction, performed in the MPSI immunocompetent murine model treated with HSC-GT, showed normalization of osteocytes and periosteum vacuolation, whereas alterations of the growth plate (Fig.1C) and the articular cartilage were improved, together with histomorphometric and pQCT long bones trabecular and cortical parameters (Fig.1D), confirming an early beneficial effect of HSC-GT on MPSIH typical skeletal features.

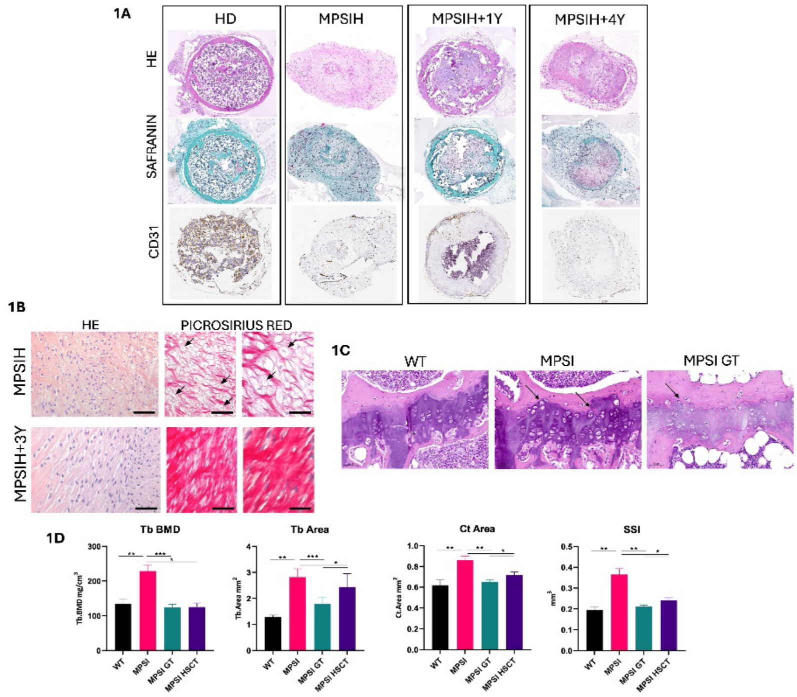

**Fig1A**: Representative histological images of bone ossicles of healthy donor (HD), MPSIH patient before HSC-GT and 1 year (MPSIH+1Y) and 4 years (MPSIH+4Y) after HSC-GT after 8 weeks of subcutaneous implantation into NSG MPSI mice stained with HE, Safranin and CD31. **Fig1B**: Representative histological images of the enthesis tissue of patient's osteomedullary biopsies (BOM) before and after HSC-GT stained with HE and Picrosirius Red. Arrows indicate vacuolated cells. **Fig1C**: Representative histological images of the growth plate of the following mice: WT, untreated MPSI and MPSI gene therapy-treated mice (MPSI GT). Arrows indicate vacuolated chondrocytes. **Fig1D**: Quantification of the trabecular (Tb) bone mineral density (BMD), Tb area, cortical (CT) area, and stress strain index (SSI) of WT, MPSI, MPSI GT and MPSI HSCT-treated mice (n=5 Mann-Whiteny test *p<0,05 **p<0,01).

**COP04**

### WTX/AMER1 is a key driver of osteogenic differentiation in humans while inhibition of Wnt ligands blunts osteosclerosis in osteopathia striata with cranial sclerosis

*Clément Nachef*[1,2]*, Arnaud Vanjak*[1,2]*, Sylvie Thomasseau*[1]*, Morgane Bourmaud*[1]*, Badre Rabhi*[1]*, Guillaume Mabilleau*[3]*, Martine Cohen-Solal*[1,2]*, Amélie Coudert*[1,4]*, Thomas Funck-Brentano*[1,2]
[1]*BIOSCAR, Inserm U1132, Paris, France*
[2]*Université de Paris, Rheumatology, Paris, France*
[3]*RMes, UMRS Inserm 1229, Angers, France*
[4]*Université de Paris, Odontology, Paris, France*

### Abstract Text

Osteopathia striata with cranial sclerosis (OSCS, OMIM#300373) is a genetic osteosclerotic disease caused by *WTX/AMER1* mutations with no specific therapy. Mutant WTX enhances Wnt signaling in osteoblasts by inhibiting the β-catenin degradation complex, thereby increasing bone formation. The objective of our study was: 1/ to characterize mutant osteoblasts function obtained from human samples; 2/ to investigate the effects of LGK-974, a Porcupine inhibitor blocking all Wnt ligands, in a murine model of OSCS.

We first collected bone samples from spinal surgeries in a patient with OSCS and 3 control patients with osteoarthritis to perform histological analyses and primary bone-cell cultures. Histological analysis of OSCS bone samples showed increased trabecular thickness, and reduced bone marrow adiposity (-40%, $p<0.05$). Primary osteoblasts derived from the patient with OSCS revealed accelerated mineralization capacity starting as early as day 7.

We then treated Prx1-Cre;Wtxflox/Y and WT mice from 8 to 11 weeks either by LGK-974 or vehicle. At 11 weeks, mice were healthy with unchanged body weight. LGK-974 significantly decreased BMD by 13% at the femur ($p<0.0001$) and by 10% at the total body ($p<0.0001$). In hemizygous males treated by LGK-974, bone microarchitecture parameters analysis showed a 23% decrease of BV/TV ($p<0.0001$), as well as Tb.N ($p<0.01$), and Ct.Th ($p<0.01$). OS and BFR decreased by 23% and 43%, respectively ($p<0.0001$), while TRAP+ cells increased by 20% ($p<0.01$).

Conclusion: While mutant WTX promotes osteoprogenitors in the osteogenic pathway, the inhibition of Wnt ligands reduces osteosclerosis, by decreasing bone formation and increasing bone resorption. Repurposing LGK-974 could be a relevant therapeutic approach to manage patients with OSCS.

**COP05**

**Enhancing Gene Therapy feasibility for Osteopetrosis through HSC expansion**

*Valeria Iannello[1,2], Elena Rizzoli[1,2], Alessandra Zecchillo[1,3], Elena Draghici[1], Michela Milani[1], Andrea Cappelleri[4,5], Roberta Gioia[6], Marta Filibian[6], Antonella Forlino[7], Valentina Capo[1,8], Anna Villa[1,8]*
*[1]IRCCS Ospedale San Raffaele, San Raffaele Telethon Institute for Gene Therapy SR-TIGET, Milan, Italy*
*[2]IRCCS Ospedale San Raffaele, Vita-Salute San Raffaele University, Milan, Italy*
*[3]University of Milano-Bicocca, The Molecular and Translational Medicine Doctoral Program DIMET, Milan, Italy*
*[4]UniMi Foundation, Mouse & Animal Pathology Laboratory, Milan, Italy*
*[5]University of Milan, Department of Veterinary Medicine and Animal Sciences, Lodi, Italy*
*[6]University of Pavia, Biomedical Imaging Laboratory, Pavia, Italy*
*[7]University of Pavia, Department of Molecular Medicine- Biochemistry Unit, Pavia, Italy*
*[8]The National Research Council CNR Institute for Genetic and Biomedical Research IRGB, Milan Unit, Milan, Italy*

**Abstract Text**

**Introduction:** Autosomal recessive osteopetrosis (ARO) is a rare genetic infant disease, caused by mutations in the *TCIRG1* gene, leading to impaired osteoclast function and defective bone resorption. This results in bone marrow failure, increased circulating CD34+ cells and premature death. The standard treatment, hematopoietic stem cell (HSC) transplantation, has limitations due to disease severity. Gene therapy (GT) could reduce complications, while *ex vivo* expansion of HSCs may enhance hematopoietic reconstitution and gene correction feasibility.

**Purpose:** We developed an optimized protocol that combines HSC expansion with GT to enhance treatment efficacy and feasibility for osteopetrosis in which limited HSCs are available.

**Methods**: HSCs (LSK CD34-CD150+) were isolated from the spleens of 10-day-old *oc/oc* pups. After 8 days of expansion, cells were transduced with a PGK.TCIRG1 lentiviral vector. Flow cytometry analysis assessed growth and phenotype. After 20 days of culture, we transplanted conditioned neonatal *oc/oc* pups with the day-0 equivalents of 30 HSCs. Transduction efficacy was assessed by vector copy number (VCN), while osteoclast functionality was assessed using CTX-I and PTH ELISAs, histology and micro-CT techniques.

**Results:** We observed up to $3.1x10^5$-fold cell expansion and flow cytometry analysis of 30-day-culture showed that expanded HSCs maintained 50% expression of primitive Sca1+Kit+ markers. These cultures could be efficiently transduced *in vitro* (mean VCN = 4) without altering their marker expression and growth rate. Additionally, osteoclast functionality in *oc/oc* cells was restored after lentiviral transduction and expansion. After transplantation, *oc/oc* mice exhibited long-term survival (up to 20 weeks) and a VCN of 2 in hematopoietic organs, indicating long-term engraftment capacity of transduced and expanded cells. Importantly, no evident signs of osteopetrosis were observed in the transplanted *oc/oc* mice.

**Conclusion:** Long-term *ex vivo* HSC expansion facilitates GT feasibility for the treatment of osteopetrosis. GT-treated *oc/oc* mice show long-term survival and significant improvements in disease phenotype, demonstrating its potential therapeutic effectiveness

**COP06**

**Bone-targeted enzyme replacement therapy ameliorates hearing loss by correcting hypomineralization and osteocyte properties of auditory ossicles in Enpp1-deficient mice**

*Ana Ocokoljic*[1], *Shivani Srivastava*[2], *Simon von Kroge*[1,3], *Paul R. Stabach*[2], *Björn Busse*[3], *Keith Weise*[2], *Ralf Oheim*[3], *Tim Rolvien*[1], *Demetrios T. Braddock*[2]
[1]*University Medical Center Hamburg-Eppendorf,*
*Department of Trauma and Orthopedic Surgery- Division of Orthopaedics, Hamburg, Germany*
[2]*Yale University School of Medicine, Department of Pathology, New Haven, United States*
[3]*University Medical Center Hamburg-Eppendorf, Institute of Osteology and Biomechanics, Hamburg, Germany*

**Abstract Text**

**Background:** Hearing loss is common in conditions caused by ENPP1 deficiency, such as generalized arterial calcification of infancy and autosomal recessive hypophosphatemic rickets type 2. Mechanistically, it is hypothesized that poor mineralization of the auditory ossicles leads to impaired sound transmission in the middle ear (i.e. conductive hearing loss).

**Purpose:** Here, we investigated whether enzyme replacement therapy (ERT) improves hearing loss in an Enpp1 deficient mouse model and whether this is associated with corrected bone properties in the ossicles.

**Methods:** $Enpp1^{asj/asj}$ mice were subjected to either a soluble or bone-targeted ERT of ENPP1-Fc (Fig. 1A). Hearing function and bone properties of the malleus, the first bone of the ossicular chain, were evaluated compared to untreated and wild-type mice (Fig. 1B). All experimental procedures were approved by official authorities and complied with current guidelines.

**Results:** The hearing deficits observed in *Enpp1*$^{asj/asj}$ mice were partially or fully corrected by both soluble and bone-targeted ERT, as evidenced by restoration of auditory brainstem response thresholds, with dose dependence and generally stronger effect of bone-targeted ERT (Fig. 1C). This was not only associated with corrected blood plasma markers of mineral metabolism (ALP (U/ml); Veh: 1.07 vs bone-targeted ERT 0.5 mg/kg: 0.66, p<0.001) but also improved matrix mineralization and restored osteocyte properties in the malleus (Fig. 1D). We further evidenced mineralized osteocyte lacunae in mice for the first time, fully corrected by the bone-targeted (N.Min.Lc/B.Ar (1/mm$^2$); Veh: 446 vs bone-targeted ERT 1 mg/kg: 232, p=0.005), but not soluble, ERT. In contrast to the human phenomenon of osteocyte lacunar mineralization (micropetrosis), the intra-lacunar mineral was overall less mineralized than the adjacent bone.

**Conclusion:** Our results point to the potential benefit of ERT in preventing hearing loss in patients with ENPP1 deficiency, possibly mediated by correction of bone properties of the auditory ossicles.

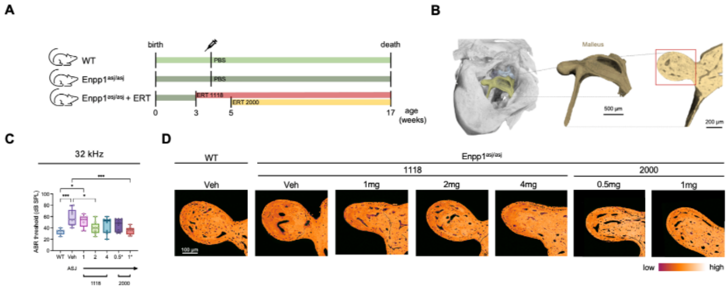

**Figure Legends:** (A) Schematic representation of the enzyme replacement therapy (ERT) regimen. (B) Representative µCT scan of the ossicles within the temporal bone. Three-dimensional and cross-section view of the malleus showing the defined region of interest (malleal processus brevis). (C) Functional hearing tests (ABR thresholds) of 17-week old wild-type, untreated and treated *Enpp1*$^{asj/asj}$ mice at 32 kHz. Statistical analysis was conducted with ANOVA (Dunn). (D) Representative quantitative backscattered electron images of the malleus from 17-week old wild-type, untreated and treated *Enpp1*$^{asj/asj}$ mice displaying the mineralization levels in pseudocolors (high and low mineralization are represented by bright and dark pixels, respectively). Scale bars: 100 µm.

### Concurrent Oral Presentations 1: Treatment of Osteoporosis and Fracture Risk

### COP07

### Weekly vitamin D and daily calcium carbonate over 48-weeks increase bone density in adolescents with HIV and 25(OH)D <75nmol/L: a placebo-controlled trial in Southern Africa

*Celia Gregson*[1], *Tafadzwa Madanhire*[1], *Nyasha V Dzavakwa*[2], *Lackson Kasonka*[3], *Hildah Banda-Mabuda*[3], *Tsitsi Bandason*[2], *Molly Chisenga*[4], *Suzanne Filteau*[5], *Katharina Kranzer*[5], *Hilda Mujuru*[6], *Ulrich E Schaible*[7], *Sarah L Rowland-Jones*[8], *Victoria Simms*[5], *Rashida A Ferrand*[5]
[1]*University of Bristol, Musculoskeletal Research Unit, Bristol, United Kingdom*
[2]*Biomedical Research and Training Institute, The Health Research Unit Zimbabwe, Harare, Zimbabwe*
[3]*University Teaching Hospital, Department of Obstetrics and Gynaecology, Lusaka, Zambia*
[4]*University Teaching Hospita, Department of Obstetrics and Gynaecology, Lusaka, Zambia*
[5]*London School of Hygiene and Tropical Medicine, Clinical Research Department, London, United Kingdom*

[6]*University of Zimbabwe, Department of Paediatrics, Harare, Zimbabwe*
[7]*Leibniz Lung Centre, Research Centre Borstel, Borstel, Germany*
[8]*University of Oxford, Nuffield Department of Medicine, Oxford, United Kingdom*

**Abstract Text**

**Background**: HIV adversely affects skeletal development despite antiretroviral therapy (ART); vitamin D insufficiency is common.

**Purpose**: Our trial determined whether vitamin D and calcium supplementation improves bone density in adolescents with HIV.

**Methods**: A multi-country individually randomised, double-blinded placebo-controlled trial of weekly vitamin D (20,000IU) plus daily calcium carbonate (500mg) for 48 weeks, recruited adolescents with HIV aged 11-19 years, taking ART for ≥6 months from HIV clinics in Zimbabwe and Zambia. The primary outcome was DXA-measured total body less-head bone mineral density (TBLH-BMD) Z-score using a UK reference population; secondary outcome being lumbar spine bone mineral apparent density (LS-BMAD) Z-score. Linear regression compared arms adjusting for site and baseline value. Sub-group analyses by baseline vitamin D insufficiency (defined in this region as 25(OH)D <75nmol/l) were pre-specified.

**Results**: 842 adolescents [(53.2% female] were enrolled; most (75.9%) had 25(OH)D <75nmol/l. At 48 weeks 751 (89.2%) had a DXA. Overall, there was no difference by arm in mean 48-week TBLH-BMD Z-score (-1.56 (SD 1.12) in intervention vs -1.53 (1.18) control arm, adjusted mean difference (AMD) -0.03 (95%CI -0.08, 0.02)). Findings were similar for LS-BMAD Z-score. However, in those with baseline 25(OH)D <75nmol/l both TBLH-BMD and LS-BMAD Z-scores were higher in those randomized to supplementation (Table-1). There was no evidence of interaction by age, sex or puberty. No drug-related severe adverse events occurred.

**Conclusions:** High-dose vitamin D and low-dose calcium, safe and cheap interventions during adolescence, may promote bone accrual towards maximizing peak bone mass, which may reduce future fracture risk in African adolescents growing up with HIV.

| Baseline 25(OH)D concentration | N | Supplemented Mean(SD) | Placebo (Mean(SD) | AMD (95%CI) | P-value | Interaction P-value |
|---|---|---|---|---|---|---|
| | TBLH-BMD Z-score | | | | | |
| <75nmol/L | 562 | -1.53(1.22) | -1.61(1.13) | 0.04 (0.00,0.08) | 0.027 | 0.078 |
| ≥75nmol/l | 189 | -1.52(1.03) | -1.45(1.12) | -0.05 (-0.16,0.07) | 0.44 | |
| | LS-BMAD Z-score | | | | | |

| | | | | | | |
|---|---|---|---|---|---|---|
| <75nmol/L | 558 | -0.64(1.19) | -0.71(1.13) | 0.04 (0.02,0.11) | 0.016 | 0.013 |
| ≥75nmol/l | 188 | -0.51(1.08) | -0.70(1.24) | -0.10 (-0.23,0.03) | 0.13 | |

**COP08**

**Performance of HiVe, a New Fracture Risk Score for Incident Hip and Vertebral Fractures: Validation and Comparison with FRAX Using Data from the SOF Study**

*Claus-Christian Glüer[1], Klaus Engelke[2], Friederike Thomasius[3], Li-Yung Lui[4], Steven R. Cummings[4,5]*
*[1]Christian Albrechts University Kiel,*
*Section Biomedical Imaging - Department of Radiology and Neuroradiology, Kiel, Germany*
*[2]Friedrich Alexander University Erlangen Nürnberg, Department of Medicine 3, Erlangen, Germany*
*[3]Frankfurter Hormon- und Osteoporosezentrum, ibid., Frankfurt, Germany*
*[4]California Pacific Medical Center Research Institute, San Francisco Coordinating Center, San Francisco, United States*
*[5]University of California San Francisco, Department of Epidemiology and Biostatistics, San Francisco, United States*

**Abstract Text**

A new fracture risk prediction algorithm has been developed for the official German Osteoporosis Guidelines of the Dachverband Osteologie: unlike other approaches, this Hip/Vertebrae fracture risk score (HiVe FRS) is calculated for 3-year incidence of hip or vertebral fractures, and is modelled as (i) population based, age and sex specific base risk of subjects without known clinical risk factors, multiplied by (ii) the relative risk of clinical risk factors (cRFs), and (iii) DXA Z-Scores areal bone mineral density of the total hip (aBMDth). Relative risks (RRs) are applied only for the 2 strongest cRFs of a given subject picked from a list of 33 cRFs that had been identified by the guideline group (through systematic review of 2267 peer reviewed publications).

Here, we validated the HiVe 3yr-FRS by applying it to women from the Study of Osteoporotic Fractures (SOF), for whom, as benchmark, results for FRAX with aBMD of the femoral neck for hip and major osteoporotic fractures had been calculated. We compared the predictive accuracy of HiVe versus FRAX by comparing risk prediction with actual numbers of incident hip or clinical vertebral fractures (follow-up time: 3 years for HiVe, 10 years for FRAX). Fracture risk was modelled based on logistic regression analysis.

In SOF, 25 of the 33 guideline-identified cRFs were available, for 7007 women, age 67-90. AUC was larger for the HiVe FRS vs any of the FRAX settings (table).

43% of women were in the "at risk" group above a HiVe intervention threshold of 3%. FRAX intervention thresholds were chosen to also identify 43%. This resulted in 40% (6.0/4.3) to 70% (6.0/3.5) higher ratios of incident fracture in the group "at risk" over the "low risk" group for HiVe compared to FRAX (table).
Conclusion: the HiVe FRS shows better performance compared to any of the FRAX settings.

| Risk prediction tool | Fx outcome: F-U time & fx | AUC | % women in "at risk" group above the intervention threshold | Ratio %women with incident fracture in "at risk" / "low risk" groups |
|---|---|---|---|---|
| HiVe 3y FRS | 3yr HF/VF | 0.79* | 43% @ >3.0% risk | 6.1 = (4.6% / 0.75%) |
| *FRAX (HIPwBMD)** | *3yr HF/VF* | *0.75** | *43% @ >3.5% risk* | *4.7 = (4.3% / 0.9%)* |
| FRAX (HIPwBMD) | 10yr HF/VF | 0.74 | 43% @ >3.5% risk | 3.8 = (16.1% / 4.2%) |
| FRAX (HIPwBMD) | 10yr HF | 0.74 | 43% @ >3.5% risk | 4.3 = (12.8% /3.0%) |
| FRAX (MOFwBMD) | 10yr HF/VF | 0.72 | 43% @ >16% risk | 3.5 = (15.7% / 4.5%) |
| FRAX (MOFwBMD) | 10yr HF | 0.73 | 43% @ >15% risk | 3.7 = (12.3% / 3.3%) |

*: *Data for FRAX (HFwtBMD) for 3yr incidence of HF & VF provided as link to test comparison of HiVe and FRAX results in same setting: difference was significant at p<0.0024 (DeLong 1988).*
FRAX tools all with BMD (results without BMD are poorer).
Legend: "F-U time & fx": follow-up time to collect incident fractures (fx) as gold standard (HF: hip fracture, VF: clinical vertebral fractures). "AUC": Area under curve of ROC analysis. "Ratio women with incident fracture in "at risk" / "low risk" group": ratio of %women with incident fractures in group above intervention threshold (predicted "at risk") over %women with incident fractures in group below intervention threshold (predicted "low risk") - larger is better.

**COP09**

**Increase in Serum DKK1 Levels Attenuates the Anabolic Response to Romosozumab in Postmenopausal Osteoporosis**

*Giovanni Adami*[1]*, Filippo Montanari*[1]*, Francesco Pollastri*[1]*, Anna Piccinelli*[1]*, Camilla Benini*[1]*,*
*Emma Pasetto*[1]*, Davide Gatti*[1]*, Ombretta Viapiana*[1]*, Maurizio Rossini*[1]
[1]*University of Verona, Rheumatology Unit, Verona, Italy*

**Abstract Text**

**Background:** Romosozumab, a monoclonal antibody against sclerostin, initially exhibits strong anabolic effects in treating osteoporosis, though its efficacy diminishes after six months as bone formation markers decline. We hypothesized that increased levels of Dickkopf-1 (Dkk1), a Wnt pathway inhibitor, might contribute to this attenuation by suppressing osteoblast activity.

**Methods:** We did a 12-month prospective observational study on postmenopausal women with osteoporosis naive to anti-osteoporosis treatment and treated with romosozumab. Serum Dkk1, Procollagen Type I N-Terminal Propeptide (P1NP), C-terminal telopeptide of type I collagen (CTX), and sclerostin were measured at baseline (M0) and at 3 (M3), 6 (M6), and 12 months (M12). Bone mineral density (BMD) was assessed at M0, M6, and M12. Linear mixed-effects models were used to analyze associations between Dkk1 and P1NP.

**Results:** 59 women were included. Dkk1 increased significantly from 38.9 pmol/L at baseline to 44.2 pmol/L at M12 (p = 0.003). P1NP increased significantly from M0 to M3 (89.4 pg/mL to 115.4 pg/mL, p = 0.004) before declining to 61.5 pg/mL at M12 (p < 0.001). CTX levels significantly decreased from 0.477 pg/mL at baseline to 0.269 pg/mL at M12 (p < 0.001). Sclerostin levels increased steeply from 36.3 pmol/L at baseline to over 1500 pmol/L at all time points (p < 0.001). BMD significantly improved across all skeletal sites. A significant association was found between Dkk1 increase and P1NP decrease between M3 and M12 (estimate -0.909, p = 0.032), suggesting a compensatory mechanism influencing romosozumab's effects.

**Conclusions:** Romosozumab improves BMD in postmenopausal osteoporosis but is associated with increased Dkk1, which may reduce its anabolic effects over time. These findings underscore the potential of dual inhibitors targeting sclerostin and Dkk1 to mitigate this limitation and enhance therapeutic outcomes.

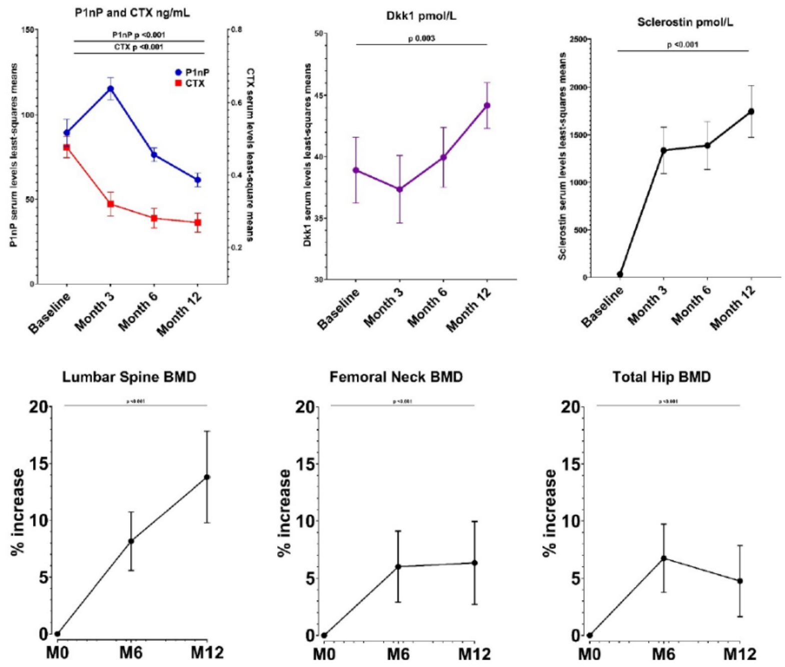

### COP10

### Epidemiology and Risk Factors of Atypical Femoral Fractures: A Retrospective Registry Study using Subtrochanteric and Femoral Shaft Fractures as Proxy

*Asger Ahlmann Bech[1], Annika Vestergaard Kvist[1], Peter Vestergaard[1], Nicklas Højgaard-Hessellund Rasmussen[1]*
*[1]Aalborg University Hospital, Steno Diabetes Center North Denmark, Aalborg, Denmark*

### Abstract Text

**Introduction:** Atypical femoral fractures (AFFs) are rare but exhibit distinct characteristics and are frequently associated with bisphosphonate use. Additional risk factors, including comorbidities such as diabetes and arthritis, have also been proposed.

**Purpose:** This study aimed to evaluate trends in AFF incidence over the past 25 years and to assess the influence of various exposures for this risk, using subtrochanteric, shaft, and lower end femoral fractures (ST/FS) as proxy for AFFs.

**Methods:** Utilizing the Danish National Patient Registry, 335,755 femoral fractures were identified in people aged >65 from 1995 to 2021. Fractures were categorized as ST/FS (n=54,404) or typical femoral fractures (TFFs) (n=281,351) based on ICD-10-codes. ICD-10 codes for ST/FS fractures were utilized as a proxy for identifying AFFs. Exposures were osteoporosis medication, glucocorticoids, and various comorbidities, identified through ICD-10 and/or ATC-codes. Associations were analyzed using a logistic regression model adjusted for age and sex.

**Results:** While both ST/FS and TFFs decreased over time, TFFs decreased significantly more, increasing the proportion of ST/FS to TFFs (P<0.001). Exposures significantly associated with ST/FS fractures were bisphosphonates (OR 1.35, 95% CI 1.31-1.39, P<0.001), denosumab (OR 1.29, 95% CI 1.11-1.50, P=0.001), raloxifene (OR 1.29, 95% CI 1.10-1.55, P=0.003), glucocorticoids (OR 1.03, 95% CI 1.00-1.07, P=0.032), type 1 diabetes (OR 1.11, 95% CI 1.04-1.19 P=0.002), type 2 diabetes (OR 1.06, 95% CI 1.03-1.10, P<0.001), intestinal bowel disease (OR 1.08, 95% CI 1.03-1.13, P=0.002), myeloma (OR 1.76, 95% CI 1.55-2.01, P<0.001), and rheumatoid arthritis (OR 1.31, 95% CI 1.25-1.37, P<0.001).

**Conclusion:** ST/FS fracture incidence has increased relative to TFFs over the past 25 years, with bisphosphonates and several comorbidities, including diabetes and arthritis, identified as significant risk factors. These findings highlight the importance of considering medication use and underlying diseases in AFF risk assessment.

**COP11**

**Incidence of Fragility Fractures in Cancer Patients Treated with Immune-Checkpoint-Inhibitors. A Systematic Review and Meta-analysis of Randomized Controlled Trials.**

*Maria Yavropoulou*[1], *Dimitrios Anastasilakis*[2], *Maria-Iosifina kasdagli*[3], *Chrysoula Giallouri*[4], *Konstantinos Palaiopanos*[5], *Athanasios Anastasilakis*[6], *Athanasios Fountas*[7], *Willem Lems*[8], *Olaf M. Dekkers*[9], *Socrates Papapoulos*[9,10], *Polyzois Makras*[11]
[1]*Medical School- National and Kapodistrian University of Athens- LAIKO University Hospital of Athens, First Department of Propaedeutic and Internal Medicine, ATHENS, Greece*
[2]*Medical School- Aristotle University of Thessaloniki, First Laboratory of Pharmacology-, Thessaloniki, Greece*
[3]*Faculty of Medicine- National and Kapodistrian University of Athens,*
*Department of Hygiene- Epidemiology and Medical Statistics, Athens, Greece*
[4]*National and Kapodistrian University of Athens- School of Medicine-,*
*Joint Rheumatology Program- Clinical Immunology-*
*Rheumatology Unit- 2nd Department of Medicine and Laboratory, Athens, Greece*
[5]*Attica General Hospital of Athens- Greece, Neurology Unit, Athens, Greece*
[6]*424 Military General Hospital- Thessaloniki- Greece.,*
*Department of Endocrinology- 424 Military General Hospital- Thessaloniki- Greece., Thessaloniki, Greece*
[7]*G. Gennimatas- General Hospital of Athens, Department of Endocrinology and Diabetes Center, Athens, Greece*
[8]*Amsterdam UMC- Amsterdam- the Netherlands,*
*Department of Rheumatology- AmsterdamUMC- Amsterdam- the Netherlands, Amsterdam,*

*Netherlands*
*[9]Leiden University Medical Center- Leiden- The Netherlands,*
*Department of Endocrinology and Metabolic Disorders, Leiden, Netherlands*
*[10]251 Hellenic Air Force & VA General Hospital- Athens- Greece, Department of Medical Research-, Athens, Greece*
*[11]251 Hellenic Air Force & VA General Hospital- Athens- Greece,*
*Department of Endocrinology and Diabetes and Department of Medical Research-, Athens, Greece*

**Abstract Text**

**Background:** Immune checkpoint inhibitors (ICIs) have revolutionized cancer treatment but are associated with several endocrine and rheumatic immune-related adverse events (irAEs). Case reports and population-based studies have shown that ICIs may be associated with higher fracture incidence, this but has not been systematically studied.

**Purpose:** To address this issue, we conducted a meta-analysis by pooling individual patient data from randomized-control-trials (RCT) of ICI monotherapy arm versus placebo or chemotherapy.

**Methods:** A systematic review of PubMed, Embase, and Cochrane Library databases was conducted to identify eligible RCTs. The primary outcome was the incidence of fragility fractures. Pathological fractures were excluded.

**Results:** A total of 30 RCTs were identified involving 19,407 patients, of whom 10,076 were ICIs users and 9,331 comprised the control group. ICIs users showed an 18% higher, albeit non-significant, incidence of fragility fractures compared to controls (Pooled odds ratio [OR]: 1.18, 95% confidence interval [CI]: 0.82–1.70). In the subgroup analysis, ICIs users had a higher incidence of fractures compared to chemotherapy (OR: 1.47, 95% CI: 0.93-2.32), but lower compared to placebo (OR: 0.80, 95%CI: 0.44-1.48), both not reaching statistical significance. Among the various factors that were further investigated for a potential effect on the reported fracture incidence in ICIs users, including disease-stage, prior chemotherapy, study-design, and the type of ICI used, none demonstrated a significant impact.

**Discussion:** We found a modest, albeit not significant trend toward increased fracture incidence in ICIs users, in line with the ICI-induced upregulation of the immune-system leading to increased bone resorption. While heightened awareness of potential skeletal effects among oncologists and other healthcare providers is prudent, our analysis does not establish a definitive link between ICIs and increased fracture incidence. Further research is needed to elucidate the relationship between ICIs and skeletal health, and guide prevention and treatment strategies of cancer survivors treated with ICIs.

**COP12**

**Can FRAX Predict Fracture Risk Equitably Across Diverse Racial and Genetic Profiles?**

*<u>Qing Wu</u>[1], Jongyun Jung[2]*
*[1]Ohio State University, Biomedical Informatics, COLUMBUS, United States*

[2]The Ohio State University, Biomedical Informatics, Columbus, United States

**Abstract Text**

**Background / Introduction:** The FRAX is widely used to estimate 10-year fracture probabilities, yet the accuracy of US FRAX across racial, ethnic, and genetic subgroups remains unclear. Disparities in fracture risk predictions may hinder equitable osteoporosis care, particularly for minority populations and individuals with high genetic risk profiles.

**Purpose:** To evaluate the accuracy and calibration of US FRAX predictions, with and without bone mineral density (BMD), across diverse racial, ethnic, and genetic groups.

**Methods:** Data from 27,512 postmenopausal women aged 50–79 in the Women's Health Initiative were analyzed, including 2,640 participants with BMD measurements. Genome-wide polygenic scores (GPS) were calculated using the LDpred algorithm with 103,155 genetic variants from GWAS summary statistics. US FRAX-predicted probabilities for major osteoporotic fractures (MOF) and hip fractures were compared to observed outcomes over a 10-year follow-up. Calibration and hazard ratios (HRs) were assessed using Fine-Gray competing risk models. Sensitivity analyses evaluated FRAX performance without BMD and among estrogen users.

**Results:** US FRAX with BMD significantly overestimated MOF risk in African American women by 72% (95% CI: 64%–90%) and Hispanic women by 48% (95% CI: 13%–69%), compared to Non-Hispanic White women, in multivariate analysis. Calibration analysis demonstrated substantial overestimation of fracture risk in African American women (MOF calibration ratio: 0.41; hip fracture: 0.42) and Hispanic women (MOF: 0.47; hip fracture: 0.74). Miscalibration was also observed across different genetic risk groups. Sensitivity analyses—including FRAX without BMD, hip fractures as the outcome, and estrogen use—confirmed these disparities, highlighting the limitations of US FRAX in diverse populations.

**Conclusion:** US FRAX inadequately accounts for racial, ethnic, and genetic diversity, leading to systemic overestimation of fracture risk for minority populations and miscalibration in high-genetic risk groups. Integrating genetic profiling and hormonal factors into FRAX could enhance prediction accuracy and promote equitable osteoporosis care.

**Concurrent Oral Presentations 2: Oxinflammaging in Bone Pathology**

**COP13**

**Age-Dependent Variation and Regional Bone Mineral Density Influence on Bone Strength in Geriatric Intertrochanteric Fractures: A Finite Element Analysis**

*Yufeng Ge[1], Rui Zhang[2], Ling Wang[3], Yong Huan[4], Shiwen Zhu[1], Xinbao Wu[1]*
*[1]Beijing Jishuitan Hospital- Capital Medical University,*
*Department of Orthopaedics and Traumatology, Beijing, China*
*[2]Chinese Academy of Sciences, Suzhou Institute of Biomedical Engineering and Technology, Suzhou, China*

*[3]Beijing Jishuitan Hospital- Capital Medical University, Department of Radiology, Beijing, China*
*[4]Institute of Mechanics- Chinese Academy of Sciences, State Key Laboratory of Nonlinear Mechanics, Beijing, China*

**Abstract Text**

**Background:** Osteoporosis is a primary cause of geriatric intertrochanteric fractures (GIF), commonly treated with femoral nail anti-rotation (PFNA) fixation. However, the biomechanical influence of regional bone mineral density (BMD) in the proximal femur across age groups remains unclear.

**Purpose:** To investigate the mechanical performance of the proximal femur in different age groups and regions of BMD using finite element analysis (FEA).

**Methods:** Fifteen GIF patients, categorized into five age groups (55, 65, 75, 85, and 95 years; 3 per group), were included. Three-dimensional proximal femur models were reconstructed from preoperative CT scans, and AO/OTA 31-A1.2 fracture models were created. PFNA fixation was subjected to quasi-static loading via FEA until structural failure occurred. Element volume with plastic strain and load-displacement curves were evaluated to assess bone plastic deformation, screw cut-out risk and failure load. Volumetric BMD (vBMD) of peri-blade tract (PB), femoral neck (FN), intertrochanteric part (IT), and total hip (TH) was quantified, and correlations between vBMD and biomechanical parameters were analyzed.

**Results:** Regional vBMDs decreased significantly with age ($P<0.05$). Patients over 85 years showed a notable increase in plastic element volume, despite a gradual reduction in vBMD; at 700N, the volume averaged 50 $mm^3$, compared to zero in younger groups (55-75 years). Stiffness and failure load progressively decreased with age, with models over 85 failing at loads below 5000N. Among the vBMDs, PB vBMD showed the strongest correlation with plastic element volume and stiffness ($-1<r^2<-0.80$, $P<0.01$).

**Conclusion:** Bone strength in GIF patients decreases progressively with advancing age, with a sharp decline after 85 years. Peri-blade vBMD in the femoral head shows a strong association with regional strain, highlighting its potential as a key predictor of screw cut-out risk. These findings offer valuable insights for preoperative assessment and optimization of surgical strategies, particularly for elderly patients over 85 years.

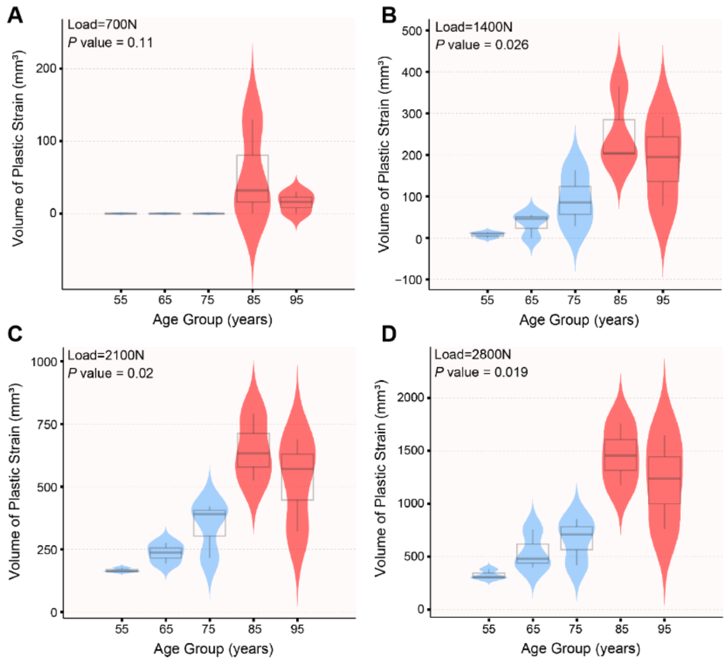

**Figure.** Plastic Strain Element Volume Across Age Groups Under Different Load Conditions. 700N (A), 1400N (B), 2100N (C), 2800N (D)

**COP14**

**Cortical bone loss and fragility precedes amyloid deposition in a triple transgenic mouse model of Alzheimer's disease**

*Giuseppina Storlino*[1], *Francesca Posa*[1], *Manuela Dicarlo*[2], *Patrizia Pignataro*[3], *Stefania Dell'Endice*[1], *Graziana Colaianni*[2], *Silvia Concetta Colucci*[3], *Tommaso Cassano*[4], *Maria Grano*[2], *Giorgio Mori*[1]

[1]*University of Foggia, Department of Clinical and Experimental Medicine, Foggia, Italy*

[2]*University of Bari, Department of Precision and Regenerative Medicine and Ionian Area, Bari, Italy*

[3]*University of Bari, Department of Translational Biomedicine and Neuroscience, Bari, Italy*

[4]*University of Foggia, Department of Medical and Surgical Sciences, Foggia, Italy*

**Abstract Text**

Alzheimer's disease (AD) and osteoporosis often coexist in the elderly. However, the pathophysiological link between these two diseases has been poorly investigated so far.

We examined the skeletal phenotype of a triple transgenic model of AD (3xTg-AD) harboring three mutant genes: β-amyloid precursor protein ($\beta APP_{Swe}$), presenilin-1 ($PS1_{M146V}$), and $tau_{P301L}$. Extracellular Aβ plaques in 3xTg-AD mice are absent in 2-month-old mice and develop incrementally from 6 months of age, with a progressive cognitive decline that worsens significantly as the mice age, showing a specific profile that closely mimics the human AD disease.

To evaluate the skeletal phenotype associated with AD neuropathology, we performed microCT on femurs and tibiae of 2-month-old male non-Tg (n= 9) and 3xTg-AD (n= 9) mice. Our results indicated profound cortical bone loss in 3xTg-AD mice compared with littermates. Specifically, we found significantly lower bone surface (BS), periosteal and endosteal perimeters (Ct.Pm), total (Tt.Ar) and bone (B.Ar.) cross-sectional area. These data were consistent with altered long bone geometry, confirmed by lower polar moment of inertia (pMOI) (-40%), an index of resistance to fracture upon torsional forces. To determine the mechanical properties of long bones, we performed 3-point-bending test, demonstrating that bone strength, load-to-fracture, and stiffness were lower in 3xTg-AD mice than littermates.

In addition, we measured the serum concentration of irisin, whose levels are known to be low in both AD and osteoporosis. Despite the result borders on statistical significance (3xTg-AD vs non-Tg, *p=0.068*), irisin serum levels correlated positively with cortical bone parameters, suggesting that this myokine may represents an early marker of cortical bone fragility in AD.

Overall, our results demonstrate that although plaque development in 2-month-old 3xTg-AD mice is still absent, cortical bone loss occurs before the accumulation of these distinctive pathological lesions, suggesting that skeletal fragility is an early event in the pathogenesis of AD.

**COP15**

**Identification and functional characterization of a novel miRNA candidate as a biomarker and therapeutic target for aging-induced bone loss**

*Devendra Pratap Singh*[1], *Alok Tripathi*[1], *Ankita Paul*[1], *Saurabh Kumar Kaushal*[1], *Megha Dixit*[1], *Divya Singh*[1]
[1]*CSIR - CDRI Central Drug Research Institute, Endocrinology, Lucknow, India*

**Abstract Text**

**Background:** Aging is a complex biological phenomenon that contributes to the progression of various chronic conditions, including osteoporosis, which is characterized by bone loss and fragility fractures.

**Purpose:** This study aimed to investigate the role of miR-668 as a novel regulator of osteoblast function and its potential in mitigating aging-induced bone loss and cellular senescence. Previous studies have implicated miR-668 in aging-related processes, hence strengthening our investigation.

**Methods:** In vitro, miR-668 expression and function were analyzed in primary mouse osteoblast cultures via miC (control), mimic, and inhibitor transfections. qPCR and immunoblotting assessed effects of miR-668 on osteoblast differentiation and osteogenic markers. Target prediction was performed using miRDB and TargetScan, with confirmation via Dual Luciferase Reporter (DLR) assay. Cellular senescence was induced with Doxorubicin (Doxo) to simulate accelerated aging. Senescence markers, SA-β-gal activity, and oxidative stress were evaluated. In vivo, IEC-approved studies involved Balb/C mice in 4 groups: Control, Doxo+miC, Doxo+miR-668, and Doxo+Anti-miR-668. MicroCT was employed to analyze trabecular microarchitecture. Bones & serum were extracted for ex vivo mineralization, histomorphometry, ELISA and protein expression studies.

**Results:** miR-668 mimic enhanced osteoblast differentiation and function by increasing osteogenic markers and targeting Znrf3 (DLR assay ***p ≤ 0.001, w.r.t miC) to activate Wnt signaling. Doxo-treated osteoblasts showed reduced miR-668 and increased Znrf3 expression. miR-668 augmentation mitigated Doxo-induced senescence by reducing p21, p53, SA-β-gal levels (% SA-β-gal+ cells ***p ≤ 0.001, w.r.t miC) and suppressing ROS. Znrf3 overexpression reversed anti-senescence and pro-osteogenic effects of miR-668, confirming its target-specific role. In vivo, Doxo+miR-668 group showed improved trabecular bone (femur) microarchitecture (BV/TV, Tb.Th ***p ≤ 0.001; Tb.N **p ≤ 0.01 w.r.t Doxo+miC), bone strength, and mineralization while maintaining bone turnover markers.

**Conclusion(s):** These findings highlight key role of miR-668 in regulating osteoblast function and mitigating age-related bone loss by targeting Znrf3, thereby underscoring its therapeutic potential.

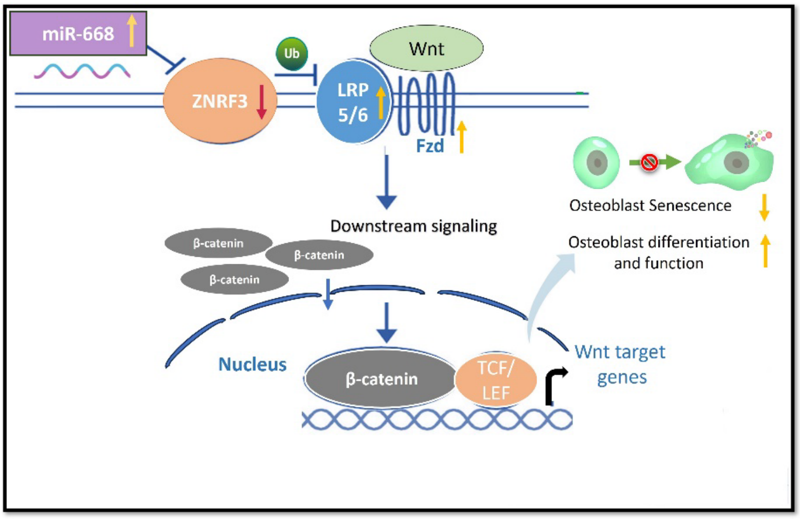

**COP16**

**A transcriptional hub for endochondral ossification and fracture repair.**

*José Valdés-Fernández[1], Miguel Echanove-González de Anleo[1], Tania López-Martínez[1], Purificación Ripalda-Cemboráin[1], Juan Antonio Romero-Torrecilla[1], Emma Muiños-López[1], Froilán Granero-Moltó[1]*
*[1]Clínica Universidad de Navarra, Cell Therapy/Orthopedic Surgery and Traumatology, Pamplona, Spain*

**Abstract Text**

**Background**: After injury bone tissue initiates a reparative response that recovers its structure and function. Failure to initiate, or delaying, this response results in fracture nonunion. The molecular mechanisms that determine fracture nonunion appearance are unknown. We found that the signaling pathways derived from hypoxia and mediated by reactive oxygen species (ROS) homeostasis control *Bmp2* expression and fracture healing initiation, having potential to induce fracture nonunion when disturbed.

**Purpose**: To determine how fracture healing is affected when *Apex1*, a nuclear factor and ROS signaling effector, is silenced.

**Methods**: All animal procedures were approved by our institutional committee for animal use in research. A closed fracture was performed in the tibia of Prrx1Cre;Apex1$^{C/C}$ (Apex1$^{KO}$) and WT mice littermates. The repair process was evaluated along the reparative phase (3-, 6-, 10- 14- and 21-dpf) by histology, immunohistochemistry, transcriptomic analysis (RNA-seq) and microcomputed tomography (µCT).

**Results:** µCT analysis showed that Apex1$^{KO}$ mice presented an impaired endochondral ossification process, with a significant reduced callus volume when compared with their WT littermates (WT, 32.04±10.50 mm$^3$; Apex1$^{KO}$, 14,45±9,35 mm$^3$; p<0.0001), presenting a significative reduction of both soft (WT, 16.63±6.47 mm$^3$; Apex1$^{KO}$, 6.47±5.46 mm$^3$; p<0.0001) and stiff (WT, 15.41±4.68 mm$^3$; Apex1$^{KO}$, 7.97±5.345 mm$^3$; p=0.0006) tissues. At 21-dpf radiographic analysis showed that in Apex1$^{KO}$ mice a persistent fracture line and sclerotic bone were present, defining a nonunion phenotype (Figure 1). Transcriptomic and histologic analysis showed that at 3-dpf periosteal response to injury was impaired in Apex1$^{KO}$ mice, resulting in reduced chondrogenesis and osteogenesis during the reparative phase at 6-, 10- and 14-dpf. In addition, silencing *Apex1* impacts chondrocyte differentiation and accumulation of cartilage intermediate at 21-dpf, that impairs fracture callus vascularization and fracture healing progression.

In conclusion, silencing *Apex1* in mesenchyme progenitors results in a nonunion-like phenotype characterized by delayed fracture healing initiation, and impaired fracture healing progression.

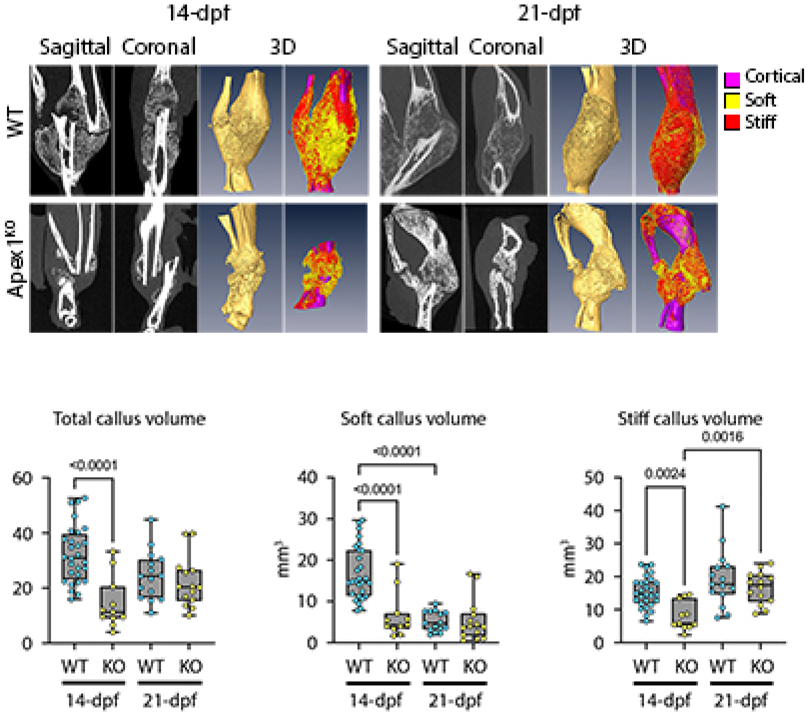

### COP17

### Impact of osteocyte-derived podoplanin on bone homeostasis in the K/BxN serum transfer model of inflammatory arthritis

*Amy J Naylor[1], Rosa Al-Qasemi[2], Gowsh Poologasundarampillai[3], Kim Moeller[2], Denise Beckmann[2],*

*Thomas Pap[2], Chris Buckley[4], Corinna Wehmeyer[2]*
*[1]University of Birmingham, Institute of Inflammation and Ageing, Birmingham, United Kingdom*
*[2]University of Muenster, Institute of Musculoskeletal Medicine, Muenster, Germany*
*[3]University of Birmingham, School of Dentistry, Birmingham, United Kingdom*
*[4]University of Oxford, Kennedy Institute of Rheumatology, Oxford, United Kingdom*

**Abstract Text**

**Background:** Osteocytes are encysted cells found in cavities (lacunae) within bone. They form dendritic extensions to develop a dense sentinel network inside the bone matrix where they modulate bone remodeling through regulation of both osteoclast and osteoblast activity. Immature osteocytes express the transmembrane glycoprotein GP38 known as podoplanin (PDPN) which is important for dendrite elongation. It has been reported that PDPN expression is regulated by inflammatory cytokines and that PDPN facilitates cell-cell communication via gap junction protein connexin-43 at the tips of their dendrites to maintain bone homeostasis.

**Purpose:** The aim of this study was to investigate the impact of cell-specific deletion of PDPN in osteocytes during inflammatory arthritis.

**Methods:** Dmp1 Cre-mice were crossed with $PDPN^{flox/flox}$-mice to generate osteocyte-specific conditional knockout mice. The K/BxN-serum transfer arthritis (K/BxN-ST) was used to study the effect of PDPN deletion on mouse arthritis progression. PDPN, osterix and connexin-43 expression was assessed by immunohistochemistry or qPCR and Western-blot. Dendrite length was evaluated by silver staining and osteoclast number by TRAP. Laboratory and synchrotron X-ray-microCT analysis was used to assess bone density parameters, lacunae and bone erosion.

**Results:** PDPN was highly expressed in osteocytes at sites of bone erosion in the inflamed joints and significantly up-regulated by IL-1beta *in vitro* (3-fold,*p*=0.0022). Interestingly, loss of PDPN in osteocytes led to significantly more bone damage in the KBxN-ST model compared to non-deleted controls (+116%,*p*=0.0411*)* but showed no differences in osteoclast number. Unexpectedly, very low ectopic bone formation (-98%,*p*=0.0033) occurred in hind limbs of PDPN deleted KBxN-ST mice. Additionally, mice with PDPN deletion exhibited decreased osteocyte dendrite length (-32,4%,*p*=0.05), similar lacunae number and size, but reduced connexin-43 expression and significantly fewer osteoblasts (-31.5%,*p*=0.017) in their inflamed joints.

**Conclusion:** PDPN is required for osteocyte dendritic extension. These data indicate that this process is bone-protective during inflammatory arthritis by supporting osteoblast recruitment for bone repair.

**COP18**

**A novel HPLC-MS-MS method for simultaneous measurement of four Vitamin D metabolites reveals Vitamin D metabolism modulation in an animal model of obesity**

*Agnese Giamborino[1], Elena Michelucci[1], Valentina Vitelli[1], Cristina Dettori[1], Chiara Bonelli[1], Leonardo Galfo[1], Beatrice Polini[1], Grazia Chiellini[1], Alessandro Saba[1], Federica Saponaro[1]*

[1]University of Pisa,
Department of Surgical- Medical and Molecular Pathology and Critical Care Medicine, Pisa, Italy

**Abstract Text**

**Introduction:** HypovitaminosisD has been associated with incidence and comorbidity of obesity. The aim of this study is to investigate in an obese animal model how metabolic, inflammatory, and hormonal markers of obesity influence vitamin D metabolism.

**Methods:** Twenty mice (CD1 males) were fed with high-fat diet (HFD); ten with standard diet (SD), vitaminD3 supplemented. 13 weeks later, 1) n=10 HFD continued HFD, 2) n=10 HFD changed to SD, 3) n=10 SD continued SD, for 2 weeks. Analysis was performed on blood samples at basal and at sacrifice; tissues were stored -80°C. A novel HPLC-MS/MS method was developed to simultaneously quantify Vitamin D3, 25OHD, $1,25(OH)_2$, $24,25(OH)_2$ in plasma samples. rtPCR was used to quantify gene expression in muscle and liver tissue.

**Results:** Groups 1 and 2 had similar *baseline* levels of 25(OH) and $1,25(OH)_2$, suggesting the preserved hepatic and renal hydroxylation mechanisms. *After 13 weeks*, HFD mice showed a significantly higher body weight compared to SD group ($p<0.01$); Vitamin D and 25OH levels were significantly lower in the HFD group ($p<0.001$) compared to SD, suggesting impaired hepatic hydroxylation. $1,25(OH)_2$ and $24,25(OH)_2$ did not differ between groups, probably due to renal/extra-renal compensation. After *two more weeks of hypocaloric diet,* Vitamin D and 25OH levels were significantly lower in HFD mice compared to SD ($p<0.001$), and those levels increased in the group 2 after weight loss ($p<0.01$). HMGCoA-R, CYP2R1, CYP27A1, vDR hepatic expression was significantly lower in HFD mice compared to SD ($p<0.001$) and increased in group 2 after weight loss ($p<0.01$), suggesting hepatic metabolism impairment. vDR expression in the skeletal muscle was significantly lower in HFD mice compared to SD ($p<0.001$) and increased in group 2 ($p<0.01$).

**Conclusions:** These findings suggest that vitamin D metabolites are modulated by obesity probably due to impaired hepatic hydroxylation, which is rescued by weight loss.

### Concurrent Oral Presentations 2: Rare Bone Diseases and Beyond

### COP19

### Detrimental effects of immune checkpoint inhibitors (ICIs) on bone health: a narrative review, on behalf of the ECTS Clinical Action Group

*Stella D'oronzo*[1], *Anda Mihela Naciu*[2], *Maria P Yavropoulou*[3], *Athanasios D Anastasilakis*[4], *John J Carey*[5], *Julien Paccou*[6], *Tim Rolvien*[7], *Willem Lems*[8]
[1]LUM University, Medicine and Surgery, Casamassima Bari, Italy
[2]Campus Bio-Medico University, Unit of Endocrinology and Diabetes, Rome, Italy
[3]National and Kapodistrian University of Athens,
Endocrinology Unit- First Department of Propaedeutic and Internal Medicine, Athens, Greece
[4]424 Military General Hospital, Department of Endocrinology, Thessaloniki, Greece
[5]National University of Ireland Galway, Department of Rheumatic Diseases, Galway, Ireland

[6]*University of Lille, Department of Rhematology, Lille, France*
[7]*University Medical Center Hamburg-Eppendorf, Department of Trauma and Orthopaedic Surgery, Hamburg, Germany*
[8]*Amsterdam University Medical Center, Department of Rheumatology, Amsterdam, Netherlands*

**Abstract Text**

**Introduction:** ICIs have revolutionized the treatment of various malignancies, leading to unprecedented improvements in clinical outcomes[1]. Several adverse events (AEs) from ICIs have been documented[2], but the risk of bone adverse events (BAEs) is uncertain. Sporadic reports[3] have described inflammatory cytokine upregulation and osteoporosis during ICI treatment, whose impact is clinically relevant especially once fragility fractures occur.

**Purpose:** reviewing current literature on BAEs during ICI treatment.

**Methods:** Relevant articles, written in English and published up to 2024, were identified in PubMed, Scopus, and WOS databases by using pre-selected keywords. Based on pre-defined inclusion/exclusion criteria, 21 articles were included in the final analysis (3 case series, 1 FDA report and 17 original studies).

We considered BAEs as at least one condition among the following: fractures, osteoporosis, osteopenia, bone pain, altered bone metabolism markers. The studies were categorized into thematic sections based on their focus.

**Results:** We found 1,509 cancer patients in 21 studies developing BAEs during ICI treatment. The described percentage of affected patients was highly variable (range 0.3-88.9%), reflecting the differences in study design, patient enrollment, and BAEs collected. Median age was >55 years, reflecting the greater propensity to both malignancy and osteoporosis in older people. BAEs were reported in patients with various primary solid tumors, in particular lung cancer patients (9 studies). Again, this is difficult to be interpreted, since this could be due to its high incidence. Several other limitations of the studies were highlighted.

**Conclusion(s):** BAEs are common in older patients receiving ICIs but must be interpreted carefully. The attributable risk warrants further investigation to mitigate these risks since diagnostic techniques and anti-osteoprotic drugs are widely available. A research agenda focusing on more high-quality reporting of BAEs from ICIs is warranted.

**References:**[1]Robert, C. Nat Commun 11, 3801(2020);[2]Martins, F. Nat Rev Clin Oncol 16, 563-580(2019);[3]Tang, J. Ther Adv Endocrinol Metab 14, 1-2(2023).

**COP20**

**Mortality and fracture risk in children with osteogenesis imperfecta: results from the French national hospital discharge database.**

*<u>Cécile Philippoteaux</u>[1], Julien Paccou[1], Emmanuel Chazard[2]*
[1]*Lille University Hospital, Rheumatology, Lille, France*

[2]*Lille University Hospital, Public Health, Lille, France*

**Abstract Text**

**Background:** Osteogenesis imperfecta (OI) is a rare genetic disorder characterized by bone fragility, recurrent fractures, and high clinical heterogeneity. Few studies have evaluated OI's burden using Health claim database, with limited focus on mortality.

**Purpose:** To evaluate immediate and long-term mortality in children with OI, fracture and refracture risk.

**Methods:** This historical cohort study included children with an inpatient stay containing an OI diagnosis, using the French national hospital discharge database (2014 to 2022). Children born before 2014 were excluded. Children were classified into newborns (≤1 month), infants (>1 and <24 months), and children (≥2 years) according to age at index stay. Data on stay length, mortality (immediate during index stay, or long-term), and fracture risk were analyzed using descriptive statistics, Kaplan-Meier survival estimates, and Cox models.

**Results:** 658 patients (5,849 stays) were included, with a mean age at index stay of 1.59 years (SD= 2.05, 95%CI [1.44–1.75]) and 52.1% of female. The cohort comprised 30.2% of newborns, 18.1% of infants and 51.7% of children. The mean stay length was 6.96 days (SD= 20.56, CI [5.39; 8.53]), significantly longer for newborns (22.57) and infants (21.81) than children (4.00) (p<0,001). Immediate intra-hospital mortality was 9.1%, highest in newborns (29.1%) compared to infants (14.3%) and children (0.88%) (p<0.001). Median follow-up was 718 days Newborn survival dropped to 68.3% (62.1–75.1) by day 212 (Figure 1). Among the 22.2% of patients hospitalized for fractures at inclusion, femoral fractures accounted for 50.7%; 30.6% of patients experienced refracture, with a median time to refracture of 250 days. The mean fracture rate was 0.31 per year (SD=1.03, 95%CI: [0.23; 0.40]).

**Conclusion:** Newborns face significantly higher immediate and long-term intra-hospital mortality compared to infants and children. About a quarter of the patients were hospitalized at inclusion for a fracture, half of these involving a femoral fracture.

Figure 1 - Survival rate over time (days) by sub-groups

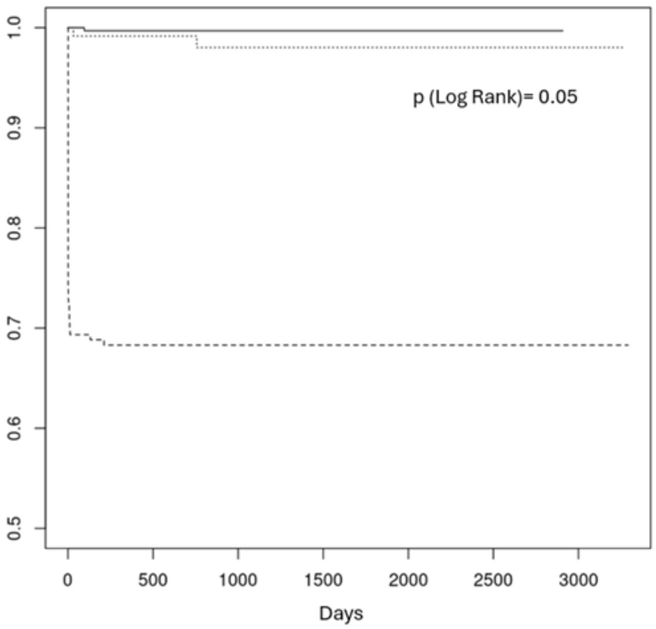

The dashed line represents the newborn group (≤1 month). The dotted line represents the infant group (>1 and <24 months). The plain line represents the children group (≥2 years).

**COP21**

**Characterization of hypophosphatemia in the outpatient setting in a tertiary referral hospital**

*Diana Ovejero*[1], *Natalia Garcia-Giralt*[2], *Xavier Nogués*[1]
[1]*Hospital del Mar Research Institute- Center for Networked Biomedical Research on Fragility and Healthy Aging CIBERFES- ISCIII-, Musculo-Skeletal Research Group, Barcelona, Spain*
[2]*Hospital del Mar Research Institute- Center for Networked Biomedical Research on Fragility and Healthy Aging CIBERFES- ISCIII-, Musculoskeletal Research Group-, Barcelona, Spain*

**Abstract Text**

**Introduction:** Chronic hypophosphatemia is a mineral disorder with a multifactorial origin that can be deleterious but that often goes underrecognized due to its nonspecific symptoms, the lack of routine serum phosphate (Pi) measurements, and low awareness among physicians.

**Purpose:** To analyze the prevalence and causes of chronic hypophosphatemia in our center.

**Materials and Methods:** All Pi measurements from adult patients attended in our hospital from 2019 to 2022 were accessed. Medical records of patients attended in outpatient clinics with Pi <2.4mg/dl were analyzed aiming to identify hypophosphatemia's most probable etiology. Those patients with repeated hypophosphatemia of unclear cause or those that exhibited characteristic manifestations of chronic hypophosphatemia were prospectively evaluated. The study received ethical approval.

**Results:** Out of a total of 86,532 Pi measurements, 2,997 were <2.5 mg/dL (3.4%). Low Pi was detected in 1,071 patients attended in outpatient clinics. The main estimated cause of hypophosphatemia was pharmacological (N=352; 32.9%), ferric carboxymaltose infusions (12.5%) and tenofovir (5%) being the most frequently involved drugs. The cause could not be determined through medical record revision in n=311 (29%), although among these patients, 140 presented secondary hyperparathyroidism due to vitamin D deficiency, and in all cases, hypophosphatemia was occasional. The subsequent causes were kidney transplantation (n=158, 14.8%), malnutrition/malabsorption (n=91, 8.5%), and primary hyperparathyroidism (n=91, 8.5%). No hereditary causes of hypophosphatemia were detected. Nine patients with recurrent hypophosphatemia of unknown cause were prospectively evaluated. Except for one patient diagnosed with oncogenic osteomalacia, hypophosphatemia remained unidentified in the remaining 8 patients despite targeted clinical evaluation. Notably, 2 of these patients were receiving infliximab treatment for ankylosing spondylitis.

**Conclusions:** Chronic hypophosphatemia is an infrequent althoug probably underdiagnosed disorder. Establishing its etiology can be challenging. The most frequently identified cause in our center was pharmacological, with ferric carboxymaltose infusions being particularly prevalent. Infliximab treatment may contribute to hypophosphatemia development.

**COP22**

**Good or Bad Donors: The Role of Cytokines in Bone Allograft Material**

*Louise Hjorth Lind[1], Jacob B. Olesen[2], Dorte Aa. Olsen[3], Line Nielsen[4], Jonna S. Madsen[3], Mikkel Ø. Andersen[4], Kent Søe[1]*
*[1]University of Southern Denmark, Department of Clinical Research, Odense, Denmark*
*[2]Odense University Hospital, Department of Pathology, Odense, Denmark*
*[3]Vejle Hospital, Department of Clinical Immunology and Biochemistry, Vejle, Denmark*
*[4]Lillebaelt Hospital, Spine Surgery and Research- Spine Centre of Southern Denmark, Middelfart, Denmark*

**Abstract Text**

**Background:** Bone allograft transplants are used as graft material in spinal fusion surgery of patients with structural deformities. However, solid bone fusion only occurs in around 30%. One explanation could be "good" and "bad" donors, possibly due to different levels of cytokines and growth factors in the allograft material. Knowledge in this area is sparse, but it would have strong clinical impact if the "good" donor could be identified and preselected upfront, based on a cytokine profile of the allograft material.

**Purpose:** Identify the consequences of different cytokine profiles in bone allograft material on human osteoclasts in cell culture.

**Methods:** Allograft bone (n=29) was stored at -80°C, thawed, minced to a paste, and centrifuged to extract clear "bone liquid". The protein concentration was determined and six concentrations (0.001 to 10 µg/ml) were titrated to human osteoclast cultures during differentiation (7 days) and osteoclastic bone resorption (3 days)) using only 1% FBS. Cytokines etc. were determined using the Olink assay and compared with experimental data and allograft donor characteristics (mean age 67.5 (50-89)), 17 female and 12 male)(ethical approval S-20200210).

**Results:** Osteoclastogenesis (TRAcP activity) and bone resorption (%ES/BS) was investigated (n=29). Increasing IL-6 levels in bone liquid seemed to favour osteoclastogenesis (p=0.09) and resorption (p=0.043), while increasing IL-1α levels seemed to inhibit osteoclastogenesis (p=0.0174) and have no effect on resorption (p=0.88). Increasing age of allograft donors correlated with increased IL-6 (p=0.0198) and decreased IL-1α levels (p=0.0132). Using multiple linear regression, various cytokines were identified, and the following appear to stimulate resorption (CCL3 p=0.006, IFN-gamma p=0.0058, EGF p=0.0091, TIE2 p=0.0173, CSF-1 p=0.0176) but not osteoclastogenesis.

**Conclusion:** Our data clearly suggest that variation in cytokine levels could explain why allograft donors can be either "good" or "bad".

**COP23**

**Automated abdominal aortic calcification extent is associated with muscle composition, strength and sarcopenia: The UK Biobank Imaging Study**

*Abadi Gebre[1], Cassandra Smith[1], James Webster[2], Afsah Saleem[1], Syed Zulqarnain Gilani[1], Gustavo Duque[3], Emma L Duncan[4], Namki Hong[5], Parminder Raina[6], Douglas P Kiel[7], William D Leslie[8], John T Schousboe[9], Yuandan Zhong[10], John P Kemp[10], Richard L Prince[11], Robin M Daly[12], Nicholas C Harvey[13], Joshua R Lewis[1], Marc Sim[1]*

*[1]Edith Cowan University, Nutrition and Health Innovation Research Institute, Joondalup, Australia*

*[2]Oxford University, Applied Health Research Unit- Nuffield Department of Population Health, Oxford, United Kingdom*

*[3]McGill University, Department of Medicine, McGill University, Canada*

*[4]King's College London, Department of Twin Research and Genetic Epidemiology- School of Life Course and Population Sciences, London, United Kingdom*

*[5]Yonsei University College of Medicine, Department of Internal Medicine- Endocrine Research Institute, Seoul, Korea- South*

*[6]McMaster University, McMaster Institute for Research on Aging-, Ontario, Canada*

*[7]Harvard Medical School, Marcus Institute for Ageing Research- Hebrew Senior Life- Department of Medicine- Beth Israel Deaconess Medical Center, Boston, United States*

*[8]University of Manitoba, Department of Radiology- Rady Faculty of Health Sciences, Manitoba, Canada*

*[9]University of Minnesota, Division of Health Policy and Management-, Minneapolis, United States*

*[10]The University of Queensland- Translational Research Institute, Mater Research Institute, Woolloongabba, Australia*

*[11]The University of Western Australia, Medical School, Perth, Australia*

*[12]Deakin University, Institute for Physical Activity and Nutrition, Melbourne, Australia*

*[13]University of Southampton, MRC Lifecourse Epidemiology Centre, Southampton, United Kingdom*

**Abstract Text**

**Background:** Abdominal aortic calcification (AAC) is associated with higher risk for cardiovascular events and can be identified during routine osteoporosis testing on DXA-derived lateral spine images. Our automated machine learning algorithm assessing AAC (ML-AAC24) identifies individuals with higher fall and fracture risk. However, the relationship between ML-AAC24 with muscle composition and strength, strong predictors of fracture risk, remain unclear.

**Purpose:** Examine if greater extent of ML-AAC24 is cross-sectionally associated with poorer measures of muscle composition, strength and sarcopenia in the UK Biobank Imaging Study.

**Methods:** ML-AAC24 extent was categorised as low (<2), moderate (2-5) and high (≥6). Magnetic resonance imaging-derived thigh fat-free muscle volume (FFMV) and muscle fat infiltration (MFI) was available in 33,946 people. Age and/or sex specific cut-points for low FFMV and high MFI were adopted (**Fig**). Muscle strength was assessed using hand grip strength (HGS, n=49,396). Muscle weakness (HGS <27 kg for male and <16 kg for female) and sarcopenia (low height-adjusted appendicular lean mass assessed from DXA and weak HGS, n=35,903) were categorised based on revised European guidelines. Logistic regression examined the associations between ML-AAC24 extent and the aforementioned muscle health measures.

**Results:** Participants mean age was 64.6 ± 7.8 years, 50.8% female. In multivariable-adjusted analysis, participants with moderate and high ML-AAC24 consistently had greater odds for having low FFVM, high MFI, weak HGS and sarcopenia, compared to those with low ML-AAC24 (**Fig**). Results were independent of established cardiovascular disease risk factors. When stratified by sex, results remained consistent across outcomes when comparing high to low ML-AAC24; with the exception of weak HGS in females.

**Conclusion:** ML-AAC24 severity was associated with poorer muscle composition and strength, as well as sarcopenia. These cross-sectional findings suggest that vascular disease has deleterious effects on muscle health. However, results need to be confirmed using longitudinal studies.

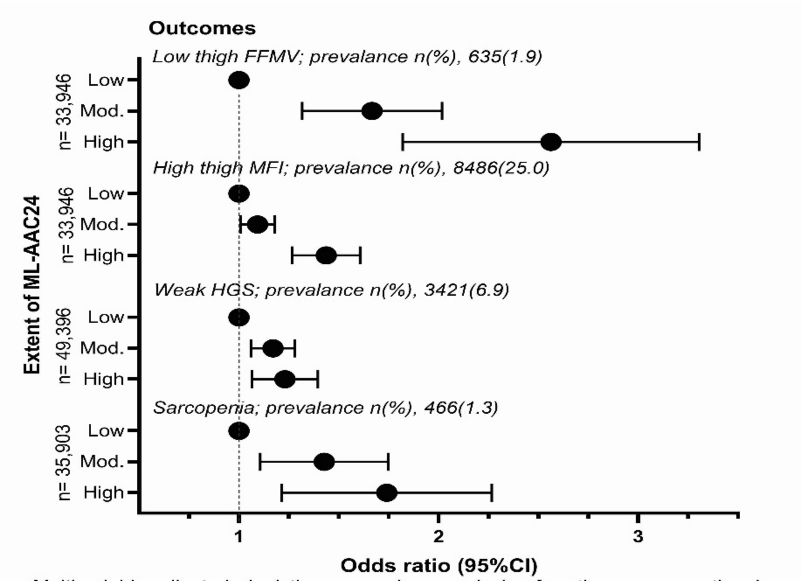

Multivariable-adjusted logistic regression analysis for the cross-sectional association between ML-AAC24 extent and odds of having poorer muscle health measures. Proportion of individuals in low (reference), moderate and high ML-AAC24 was ~79%, 16% and 5%, respectively. Model adjusted for age, BMI, physical activity, smoking status, ethnicity, prevalent diabetes and atherosclerotic cardiovascular disease, geographic region in the UK, deprivation index and imaging year. Fat free muscle volume (FFMV) and muscle fat infiltration (MFI); hand grip strength (HGS); EWGSOP2-defined sarcopenia. Low FFMV adopted age- and sex-specific cut-points; i) 45-59 years: male <9.61 L, female <6.21 L; ii) 60+ years: male <9.01, female <5.93 L. High MFI was based on >75th percentile for male (>7.64%) and female (>8.75%).

**COP24**

**The duration of Type 2 Diabetes Mellitus is an important determinant of bone material properties: the DIABONE Study**

*Delphine Farlay[1], Blandine Merle[1], Elodie Debeire[1], Evelyne Gineyts[1], Francois Duboeuf[1], Roland Chapurlat[1], Romane Roux[2], Tatiana Kochetkova[2], Philippe Zysset[2], Pascale Chavassieux[1], Emmanuelle Vignot[1]*
*[1]Inserm Umr 1033, Université de Lyon, Lyon, France*
*[2]ARTORG Centre, University of Bern, Bern, Switzerland*

**Abstract Text**

**Background:** Type 2 Diabetes Mellitus (T2DM) is a risk factor for fracture in some patients, despite normal or increased BMD. Thus, alteration in bone material properties have been proposed to explain this skeletal fragility.

**Objective :** To investigate the consequence of T2DM on bone material properties.

**Material and Methods:** After ethical approval, 19 T2DM patients (9 men 10 women, 71 ± 8 yrs, BMI: 32± 4) were recruited and matched for sex, age and BMI with 19 controls (CTRL, 72 ± 6 yrs BMI: 32 ± 6). The median duration of diabetes was 11 yrs (range: 2-40 yrs), and patients were treated with metformin, sulphonylureas, DPP-4 inhibitors, GLP-1 agonists or insulin. Both groups of patients had a T-score > -2 and underwent hip or knee arthroplasty. Bone strain Index (BSI) was obtained from DXA images. During surgery, iliac bone biopsies were collected for bone histomorphometry, nonenzymatic crosslink (pentosidine) analysis by HPLC, X-ray microradiography (degree of mineralization of bone, DMB).

**Results:** In T2DM patients, CTX and P1NP were significantly lower compared to CTRL ($p<0.04$). While no significant difference in cortical (Ct) and cancellous (Cn) bone was observed by histomorphometry, the diabetic groups was characterized by a decrease of bone formation and bone resorption parameters. No difference in bone pentosidine concentration was found. Cancellous DMB was found significantly higher in diabetic than in CTRL group ($p=0.0043$). Interestingly, the duration of diabetes was significantly negatively correlated with bone remodeling activity (Cn-BFR/BS, $r'=-0.762$, $p=0.004$), and neck and femoral BSI ($r'=-0.493$ and -0.532, $p<0.05$), but significantly positively correlated with Cn-pentosidine ($r'=0.534$, $p=0.027$), and with Ct and Cn DMB ($r'=0.595$ and 0.501, $p<0.05$).

**Conclusion:** In T2DM patients, the duration of diabetes appears to be a main determinant of modifications of bone material properties.

### Poster Sessions

### Poster Sessions: Biomarkers and biochemical testing

### P001

### Psychiatric Medications use in Patients with Chronic Hypoparathyroidism before and after diagnosis: A nationwide cohort study in Sweden.

*Sigridur Björnsdottir[1], Wafa Kamal[1], Ylva Trolle Lagerros[2], Michael Mannstadt[3], Eystein Husebye[4], Tim Spelman[5], Outi Mäkitie[6], Olle Kämpe[7]*
*[1]Karolinska Institutet, Department of Molecular Medicine and Surgery, Stockholm, Sweden*
*[2]Karolinska Institutet, Department of Medicine, Huddinge, Sweden*
*[3]Massachusetts General Hospital and Harvard Medical School, Endocrine Unit, Boston, Sweden*
*[4]Karolinska Institutet, Department of Medicine, Stockholm, Sweden*
*[5]Karolinska Institutet, Department of Clinical Neuroscience, Stockholm, Sweden*
*[6]Karolinska Institutet, Clinical Genetics, Stockholm, Sweden*
*[7]Karolinska Institutet, Department of Medicine- Solna, Stockholm, Sweden*

**Abstract Text**

**Context:** Patients with chronic hypoparathyroidism (hypoPT) have an increased risk of depression/bipolar disorder and possible anxiety. However, detailed data on psychiatric drug use in this group remains limited.

**Objective:** To assess psychiatric drug prescription pattern in patients with chronic hypoPT before and after diagnosis compared to matched controls.

**Methods:** Using the Swedish national registers, we identified 1,255 patients with chronic hypoPT treated with active vitamin D from 2005 onwards, and 12,550 matched controls. Data on neuropsychiatric drugs (ATC-codes: N05A, N06A, N05B, N05C, N03A) were collected for three years pre- and post-diagnosis of chronic hypoPT, or until December 31, 2020. Drug use was defined as ≥2 dispensations.

**Results**: Adjusted for baseline comorbidities and thyroxin use, patients with chronic hypoPT showed higher post-diagnosis use of psychiatric drugs than the controls. For **Antipsychotics:** Increased use three years post-diagnosis (HR 1.59; 95% CI 1.10–2.30), particularly in non-surgical form of chronic hypoPT. **Anxiolytics**: Higher use during the first-year post-diagnosis (HR 1.31; 95% CI 1.06–1.61), especially in women and non-surgical chronic hypoPT.**Hypnotics/Sedatives**: Increased use in the first two years post-diagnosis (HR 1.26; 95% CI 1.06–1.50), primarily in surgical form of chronic hypoPT**.** **Antiepileptics**: Greater use in the second- and third-years post-diagnosis (HR 1.92; 95% CI 1.42–2.58) in both surgical and non-surgical hypoPT. **Antidepressants**: No difference was found in use after the diagnosis but significantly lower use in surgical form of chronic hypoPT three and one year before diagnosis. No differences were observed in other psychiatric drug prescriptions before diagnosis.

**Conclusion:** In this large Swedish cohort, patients with chronic hypoPT showed increased post-diagnosis use of psychiatric drugs, particularly antipsychotics, anxiolytics, hypnotics/sedatives, and antiepileptics, with variations based on surgical versus non-surgical etiology. These findings highlight the importance of psychiatric evaluation and management in this patient group.

**P003**

**Prevalence of diabetes mellitus among patients with severe carpal tunnel syndrome**

*Maja Velinovic[1], Marko Stojanovic[2], Violeta Bascarevic[3], Predrag Nedeljkovic[3], Milan Stajic[3], Milos Vucetic[3], Zeljko Curcic[3], Andjelka Milic[4], Bojan Petrovic[5], Drasko Vasovic[6], Nikola Bogosavljevic[7], Stanislav Rajkovic[7], Veselin Aleksic[8], Lazar Miceta[9]*
*[1]Institute for Orthopaedics Banjica, Internal Medicine Department, Belgrade, Serbia*
*[2]Clinic for Endocrinology- Diabetes and Metabolic Diseases- University Clinical Center of Serbia- Medical Faculty- University of Belgrade, Department of Neuroendocrinology, Belgrade, Serbia*
*[3]Institute for Orthopaedics Banjica, Department of Plastic and Reconstructive Surgery, Belgrade, Serbia*
*[4]Institute for Orthopaedics Banjica, Department of Laboratory Medicine and Pathobiology, Belgrade, Serbia*
*[5]Institute for Orthopaedics Banjica, Orthopaedic Oncology Department, Belgrade, Serbia*
*[6]Institute for Orthopaedics Banjica, Department of Orthopaedics and Traumatology of the Locomotor System, Belgrade, Serbia*
*[7]Institute for Orthopaedics Banjica- Medical Faculty- University of Belgrade, Orthopaedic Oncology Department, Belgrade, Serbia*
*[8]Institute for Orthopaedics Banjica, Department of Spinal Surgery, Belgrade, Serbia*
*[9]Institute for Orthopaedics Banjica, General Orthopaedic Department, Belgrade, Serbia*

**Abstract Text**

**Background**: Carpal tunnel syndrome (CTS) is the most common entrapment neuropathy. CTS is epidemiologically associated with both type 1 (DM1) and type 2 diabetes mellitus (DM2). Pathophysiological basis for this association are still debated, but mostly attributed to: hyperglycaemia-induced median nerve oedema, the effects of vascular endothelial growth factor and advanced glycation end-products.

**Aim**: The aim of this cross-sectional pilot study was to examine the prevalence and clinical characteristics of DM among patients with advanced CTS.

**Patients and Methods:** Patients with severe degree of CTS (n = 71) requiring surgical treatment were included and analysed for presence, type, duration, and treatment of DM.

**Results:** The cohort consisted of 52 female and 19 male patients, 58.4 ±1.49 years old, with the average BMI of 27.99 ±0.65kg/m$^2$. 11 patients (15.49%) had DM2, treated only with oral medications in 9 and with insulin in 2. One patient had DM1 (1.41%). The average duration of the disease was 10 ± 2 years. According to the average HbA1c 6.20 ±0.25%, patients were well controlled on current treatment. The average duration of CTS symptoms was significantly longer ($p < 0.05$) in patients with DM (6.35 ±2.59) than those without DM (2.56 ±0.50)

**Conclusion:** Results of our pilot study emphasise the prevalent association of diabetes mellitus and carpal tunnel syndrome, additionally associated with the duration of the disease. Prevalently well controlled diabetes rarely requiring additive insulin treatment indicates that other additional pathophysiological mechanisms in CTS pathogenesis need to be addressed in our future research.

**P004**

### Uric acid and bone mineral density in postmenopausal women

*Olga Dobrovolskaya[1], Nikolay Demin[1], Maria Kozyreva[1], Elena Samarkina[2], Natalia Toroptsova[1]*
*[1]V.A. Nasonova Research Institute of Rheumatology, Department of Osteoporosis, Moscow, Russia C.I.S.*
*[2]V.A. Nasonova Research Institute of Rheumatology, Laboratory of Immunology and Molecular Biology of Rheumatic Diseases, Moscow, Russia C.I.S.*

**Abstract Text**

**Objective:** to assess the association of uric acid (UA) level with bone mineral density (BMD) in postmenopausal women.

**Material and methods:** 263 postmenopausal women (median age 62 [56; 67] years) enrolled in the study. Dual-energy X-ray absorptiometry (DXA) to measure BMD of lumbar spine (LS), femoral neck (FN) and total hip (TH) was performed. All patients underwent laboratory examination to assess the level of UA in the blood serum (spectrophotometry; Cobas C311 analyzer). The level of UA>360 µmol/l was taken as hyperuricemia (HU). The study was approved by the local ethics committee.

**Results:** Median UA level was 273.9 [220.0; 326.0] µmol/l, 33 (12.5%) women had HU. A cut-off point (200 mmol/l) was found using ROC analysis. In women with UA <200 µmol/l BMD at any region was significantly less and the frequency of the osteoporosis was more than in those with UA >200 µmol/l. We found correlation between BMD of LS and TH with UA (p=0.001 and p=0.004 respectively). In subgroup analyses according to age in women <60 years old association among BMD of LS and UA was found (p=0.005), in persons 60-70 years old BMD of FN and TH positively correlated with UA (p=0.004 and p=0.002 respectively). There were no significant correlations between BMD and UA in women >70 years old. In the univariate linear regression associations between BMD and UA were confirmed. We didn't find any association between Trabecular Bone Score, 10-year probability of fracture according to FRAX, frequency of previous low-energy fractures and UA.

**Conclusion:** The UA level <200 µmol/l can be used as an additional risk factor of osteoporosis. Postmenopausal women with UA <200 µmol/l need to carry out DXA.

**P005**

**Bone mineral density and biochemical markers in women with rheumatoid arthritis**

*Olga Dobrovolskaya[1], Elena Samarkina[2], Natalia Toroptsova[1]*
[1]*V.A. Nasonova Research Institute of Rheumatology, Department of Osteoporosis, Moscow, Russia C.I.S.*
[2]*V.A. Nasonova Research Institute of Rheumatology,*
*Laboratory of Immunology and Molecular Biology of Rheumatic Diseases, Moscow, Russia C.I.S.*

**Abstract Text**

**Objective:** to study the associations of bone mineral density (BMD) with biochemical markers of blood serum in postmenopausal women with rheumatoid arthritis (RA)

**Material and methods**: 173 women with RA were examined (age 61.0 [56.0; 66.0] year). Bone mineral density (BMD) was measured in all patients at lumbar spine (LS), femur neck (FN) and total hip (TH) by dual energy X-ray absorptiometry. C-reactive protein (CRP), rheumatoid factor, anti-citrullinated protein antibodies (ACPA), parathyroid hormone (PTH), interleukin 6 (IL6), adiponectin, and leptin were determined. The study was approved by the local ethics committee.

**Results:** The linear regression analysis revealed a significant association, independent of age, body mass index (BMI) and post menopause duration, between BMD at any site and PTH (β= -0.22, β= -0.35 and β= -0.30, for LS, FN and TH, respectively), CRP (β= -0.18, β= -0.23 and β= -0.22, for LS, FN and TH, respectively) and leptin (β=0.35, β=0.32 and β=0.42, for LS, FN and TH, respectively). Significant associations were also found between adiponectin and BMD in LS and TH (β= -0.36 and β= -0.28, respectively), ACPA and BMD in FN and TH (β= -0.21, β= -0.24, respectively) and IL6 and BMD in FN (β=0.37).

**Conclusion:** Our study demonstrated that CRP, ACPA, PTH, IL6, adiponectin and leptin were associated with BMD independently of age, BMI and duration of post menopause in women with RA.

**P006**

**Bone mineral density and bone micro-architecture evolution after kidney transplantation: association with early changes in bone turnover biomarkers**

*Olivier Malaise*[1], *François Jouret*[2], *Laurence Seidel*[3], *Clio Ribbens*[1], *Etienne Cavalier*[4], *Pierre Delanaye*[2], *Antoine Bouquegneau*[2]
[1]*CHU Liège, Rheumatology department, Liège, Belgium*
[2]*CHU Liège, Nephrology department, Liège, Belgium*
[3]*CHU Liège, Biostatistics department, Liège, Belgium*
[4]*CHU Liège, Clinical biology department, Liège, Belgium*

**Abstract Text**

**Background / Purpose:** Mineral abnormalities are frequent after kidney transplantation (KTx). This study (1) evaluated areal bone mineral density (aBMD) and cortical and trabecular microarchitecture over 1-year post-KTx and (2) assessed whether bone biomarkers changes at 3 months (3m) predict bone loss at 1 year (1yr).

**Methods:** 75 patients were prospectively enrolled. Bone biomarkers (PTH, BALP, P1NP, TRAcP-5b, CTX, sclerostin) were measured at baseline (D0), 3m and 1y after KTx. DXA assessed aBMD (lumbar spine (LS), hip and radius). HR-pQCT analyzed bone microarchitecture (distal radius and tibia).

**Results:** DXA showed significant LS and femoral neck (FN) aBMD declines between D0 and 3m ($p<0.03$), with partial LS recovery by 1yr. At 1y, 30%, 29% and 50% of patients showed significant LS, FN and total radius BMD loss ($p<0.0006$). HR-pQCT revealed significant declines in total vBMD, trabecular vBMD, and trabecular bone volume fraction (BV/TV) between D0 and 3m ($p<0.002$), that persisted at 1yr ($p<0.003$). Cortical vBMD and thickness were significantly reduced by 1yr ($p<0.0001$).

Bone turnover biomarkers decreased rapidly at 3m and stabilized thereafter. Multivariate analysis identified delta BALP at 3m as independent predictor of LS loss at 1yr (OR: 1.02; $p=0.01$), and delta PTH, CTXs and BLAP at 3m as independent predictors of total radius loss at 1yr (OR:1.005, 1.001 and 1.012 respectively; $p\leq0.04$). Baseline sclerostin predicted cortical vBMD loss (OR:5.651; $p=0.01$), while delta BALP at 3m is an independent predictor of total vBMD loss at 1yr (OR:1.283; $p=0.0009$).

**Conclusion:** Bone loss post-KTx occurs rapidly within 3m and progresses over 1yr, in trabecular and cortical compartments, with more than 30% of patients with decrease exceeding the threshold in the 3 major sites, while HR-pQCT reveals microarchitectural deterioration. Early biomarker changes, especially BLAP, can predict bone loss, emphasizing the importance of biomarker monitoring to identify high-risk patients and optimize bone health interventions.

**P007**

**Bone proteomic analysis in modeled osteoporosis reveals potential new biomarkers**

*Elisavet Ioannidou[1,2], Achilleas Moschos[1,2], Martina Samiotaki[2], Eleni Douni[1,2]*
*[1]Agricultural University of Athens, Department of Biotechnology, Athens, Greece*
*[2]Biomedical Sciences Research Centre "Alexander Fleming", Institute for Bioinnovation, Vari-Athens, Greece*

**Abstract Text**

**Background:** RANKL is a key regulator in osteoclastogenesis and bone resorption. Our lab has established a genetic mouse model of osteoporosis by overexpression of human RANKL in transgenic mice (TgRANKL) for the elucidation of the molecular basis in bone resorption.

**Purpose:** We aimed to identify and validate differentially expressed proteins (DEPs) in the femurs between WT and TgRANKL mice with potential clinical value in osteoporosis.

**Methods:** We isolated proteins from flushed femurs of TgRANKL and wild-type (WT) mice of both sexes (n=4–6 per group and sex), performed proteomics analysis and identified DEPs (|log2FoldChange| > 0.4, FDR < 0.01).

**Results:** We identified a total of 2,200–2,900 proteins per sample, with approximately 50% showing differential expression between TgRANKL and WT femurs. Enrichment analysis of upregulated proteins indicated associations with protein metabolism, translation, endoplasmic reticulum and Golgi. Conversely, downregulated proteins were primarily linked to cellular migration, extracellular matrix organization, skeletal system development, and cell-substrate adhesion. STRING analysis highlighted key hub genes, underscoring their significance in bone resorption. Comparative analysis between TgRANKL males and females identified shared proteins and DEPs, which are being further explored to elucidate mechanisms underlying sex-specificity in osteoporosis.

**Conclusions:** Proteomic analysis of bone samples may serve as a basis for identifying DEPs, revealing new pathogenic mechanisms underlying osteoporosis, and discovering potential novel biomarkers.

*This research work was supported by the Hellenic Foundation for Research and Innovation (HFRI) under the 4th Call for HFRI PhD Fellowships (Fellowship Number: 10704).*

**P008**

**Irisin levels are correlated with age and behavioural performance in Post-surgical HypoPT condition**

*Cristina Dettori[1], Federica Saponaro[1], Agnese Giamborino[1], Leonardo Galfo[1], Chiara Bonelli[1], Grazia Chiellini[1], Beatrice Polini[1], Caterina Ricardi[1], Patrizia Pignataro[2], Silvia Colucci[2], Manuela Dicarlo[3], Graziana Colaianni[3], Maria Grano[3]*
*[1]University of Pisa,*
*Biochemistry Unit- Department of Surgical- Medical and Molecular Pathology and Critical Care, Pisa, Italy*
*[2]University of Bari, Department of Translational Biomedicine and Neuroscience, Bari, Italy*
*[3]University of Bari, Department of Precision and Regenerative Medicine and Ionian Area, Bari, Italy*

**Abstract Text**

**Introduction:** Hypoparathyroidism (HypoPT) is a rare endocrine disease and Post-surgical HypoPT (PS-HypoPT) is the most common cause leading to neuropsychological symptoms. Irisin is a myokine secreted by contracting muscle, which mediates beneficial effects on several targets, including brain. The aim of this study was to evaluate irisin in PS-HypoPT condition.

**Methods:** A surgical rat model of PS-HypoPT was obtained, treated with gluconate-calcium to maintain normocalcemia. A cohort of 40 animals was divided in 4 subgroups: n= 10 PS-HypoPT rats (PTx) 11 weeks of age, n=10 PTx rats 28 weeks of age, n=10 controls shamed-operated (Crl) 11 weeks of age and n=10 Crl 28 weeks of age. All animals underwent serum calcium and irisin measurement and behavioural testing Morris Water Maze (MWM).

**Results:** Serum irisin levels were statistically significantly lower in younger animals compared to elderly (16.5±3.3 vs 13.0± 3.5, p=0.014). In the ANOVA analysis including the 4 groups, we observed that PTx old animals showed the lowest levels of irisin (p=0.04) and the rate of irisin decrease between 11 and 28 weeks of age was significantly higher in PTx animals, compared with Crl (p=0.03). We observed that in MWM test, animals with lower levels of irisin (<14 mg/dl as median cut-off), showed a worse performance at the test: number of entries in the zone of the platform (p=0.04), time spent in the zone of platform (p=0.03), total distance (p=0.02), resting time in the zone of platform (p=0.02). Moreover, PTx animals with lower levels of irisin, showed a higher Escape Latency Time compared to the other groups (p=0.01).

**Conclusions:** We observed that irisin decreases with age and is reduced in PS-HypoPT animals. Moreover, irisin levels are correlated with the worse performance at behavioural testing in the PS-HypoPT group of animals. Further studies are ongoing to understand the role of irisin in PS-HypoPT.

**P009**

**Utility of Bone Turnover Markers and the Impact of Denosumab Cessation and Age in Patients Undergoing Dentoalveolar Surgery**

*<u>Charles Inderjeeth</u>[1], Jessica Devlin[2], Diren Che Inderjeeth[3], Dieter Gebauer[2]*
[1]*Sir Charles Gairdner Osborne Park Health Care Group, Geriatric- Acute and Rehabilitation Medicine, Nedlands, Australia*
[2]*University of Western Australia, Dental Faculty, Perth, Australia*
[3]*Sir Charles Gairdner Osborne Park Health Care Group, Geriatric- Acute and Rehabilitation Medicine, Perth, Australia*

**Abstract Text**

**Background:** Bone antiresorptive medications can increase the risk of medication-related osteonecrosis of the jaw (MRONJ), particularly following invasive dental procedures. Bone turnover markers (BTMs), including C-terminal telopeptide of type 1 collagen (CTX), N-terminal propeptide of type I procollagen (P1NP), and N-terminal telopeptide of type 1 collagen (NTX), are useful for assessing bone metabolism.

**Purpose:** Evaluate the correlations between BTMs and impact of medication cessation and age in patients requiring dentoalveolar surgery.

**Methods:** Statistically powered; prospective cohort of 56 patients on denosumab referred to a maxillofacial surgeon for dentoalveolar surgery. Serum CTX, serum P1NP, and urinary NTX were measured at three time points: before the drug holiday (initial), at the time of surgery (> 6 months after the last denosumab dose; PreOp), and before resuming denosumab (> 3 months post-surgery; PostOp). Data were analyzed to determine correlations between BTMs and the effects of time off medication and age.

**Results:** Age 75.54 years (SD=8.67). 4/56 developed ONJ. Strong positive correlations were between three BTMs; correlation coefficients 0.846 (CTX and P1NP), 0.837 (CTX and NTX); 0.799 (P1NP and NTX). Linear regression models: NTX and P1NP were good predictors of CTX levels. The model using both markers explained 74.1% of the variability in CTX (R-squared=0.741), with an average error (RMSE) of 209.15. Days off medication significantly impact CTX levels ($p<0.001$). Age did not significantly influence BTM levels.

**Conclusion:** CTX, P1NP, and NTX are strongly correlated, and NTX and P1NP can be used to predict CTX levels accurately in patients on denosumab therapy who require dental extractions. The duration off medication significantly affects BTM levels. Age did not affect BTM changes over timepoints. These findings provide valuable insights for managing patients on antiresorptive therapy who require invasive dental procedures and highlight the importance of monitoring BTMs to assess bone metabolism and surgical timing.

Ethics: University of Western Australia

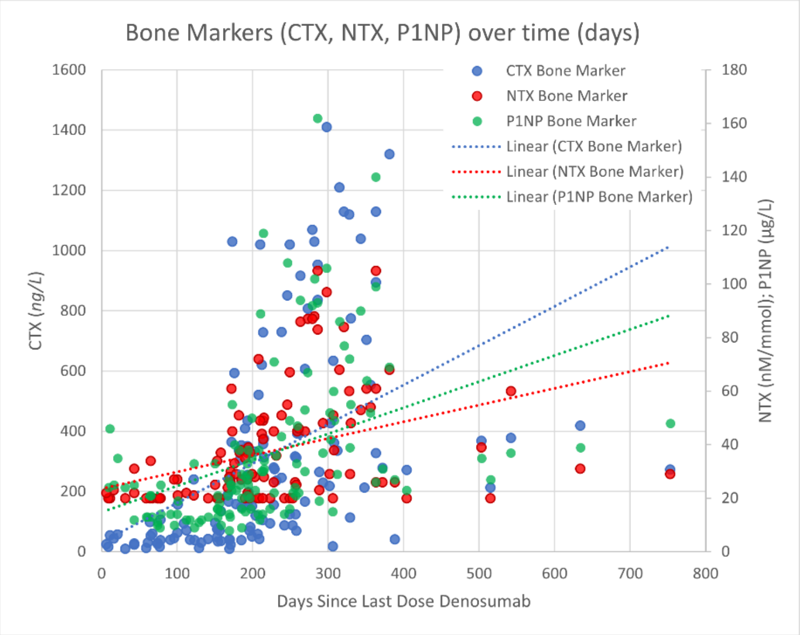

**P010**

**Platelet count and systemic immune-inflammation index as markers of fracture risk**

**postmenopausal women**

_Cecilia Oliveri_[1], _Tonino Midili_[1], _Carmen Guidara_[1], _Evelina Giovinazzo_[1], _Fabio Malacarne_[1], _Concetto Parisi_[1], _Maria Carmela Furci_[1], _Nunziata Morabito_[1], _Giorgio Basile_[1], _Antonino Catalano_[1]
[1]_University of Messina, Clinical and Experimental Medicine, Messina, Italy_

**Abstract Text**

**Background/Introduction**: Recent evidence suggests the involvement of platelets in the mechanisms regulating bone remodeling and healing after fragility fracture.

**Purpose:** To investigate the association with bone mineral density (BMD).

**Methods:** The following variables were evaluated from a complete blood count: platelet count, mean platelet volume (MPV), platelet distribution width (PDW), plateletcrit **(**PCT), percentage of large platelets (P-LCR), platelet-lymphocyte ratio (PLR), and the Systemic immune-inflammation index (SII). Bone mineral density (BMD) was measured using gold-standard dual-energy X-ray absorptiometry (DXA).

**Results:** Participants (n=124, mean age 68.4 ± 9 yr) were stratified into two groups according to the median platelet count; the "lower platelet count group" had a count of 200,000 (174,000 to 226,000), while the "higher platelet count group" had a count of 281,500 (256,500 to 308,500) $mm^2$. A borderline significant difference (p=0.08) was noted for hip fracture risk, with a higher predicted fracture risk in the "higher platelet count group". When analyzing the BMD values evaluated at the lumbar spine and femur, a statistically significant difference was found in lumbar spine BMD between the two groups, with lower BMD in the "higher platelet count group" (p=0.03). Stratifying SII into two groups (higher and lower SII, respectively 950848.6±746097.9 vs 355751.2±88662.6; p <0.0001) a different estimated 10-yr probability of femoral fracture was observed between the group with a higher SII value compared to the group with a lower value (10.5±10 vs 5.2±4.8%; p=0.01). Univariate regression analysis revealed a significant positive correlation between age and PDW (r=0.188, p=0.047), and P-LCR (r=0.208, p=0.03), and a negative association between vitamin D status and P-LCR (r=-0.301, p=0.034).

**Conclusion:** Our data suggest that SII and total platelet may be markers of fracture risk, also due to their reproducibility and cost-effectiveness.

**P011**

**Romosozumab vs. teriparatide: Evaluating early bone mineral density gains at six months in real-world practice from Oswestry, UK.**

_Chadi Rakieh_[1], _Diane Powell_[1], _Tracey Roberts_[1], _Julie Cole_[1], _Shu Ho_[2], _Bernhard Tins_[3], _Claire Mennan_[1]
[1]_Robert Jones and Agnes Hunt Orthopaedic Hospital, Metabolic Bone Disease, Oswestry, United Kingdom_
[2]_Robert Jones and Agnes Hunt Orthopaedic Hospital, Care of the Elderly, Oswestry, United Kingdom_

[3]Robert Jones and Agnes Hunt Orthopaedic Hospital, Radiology, Oswestry, United Kingdom

**Abstract Text**

**Background:** Anabolic therapy is recommended for osteoporosis patients at imminent fracture risk. However, limited data exist on the comparative efficacy and speed of action of different anabolic agents.

**Purpose:** To compare early bone mineral density (BMD) response at the hip and spine between romosozumab (Romo) and teriparatide (Teri) in a real-world setting.

**Methods:** Prospective observational study of postmenopausal women initiated on either Romo or Teri in 2023 as standard of care in a UK metabolic bone service. BMD and Trabecular Bone Score (TBS) were assessed at baseline and six months. Analysis of covariance (ANCOVA) was used to compare percentage change at six months between the treatment groups.

**Results:** Six-month BMD data were available for 68 Romo and 14 Teri patients. Baseline characteristics were well balanced (Table 1). At six months, Romo showed significantly greater BMD gains at the spine (9.6% vs 3.7%, p=0.019) adjusting for baseline BMD and age. BMD changes at the femoral neck (3% vs 3.6%) and total hip (3.3% vs 2.5%), and changes in TBS (1.1% vs -0.6%) were not statistically significant. Figure 1 summarises responses based on the least significant change demonstrating higher response rates in the Romo group.

**Conclusions:** Romosozumab demonstrated significantly greater spine BMD gains at six months compared to teriparatide. This finding may influence anabolic agent selection, suggesting Romo could offer more immediate benefits for patients at imminent risk of vertebral fracture. Larger longitudinal studies including fracture outcomes are needed to validate these results.

Table 1. Baseline characteristics

| | Romosozumab | Teriparatide | p-value |
|---|---|---|---|
| Age (years)† | 74.7(8.5) | 74.3(8.8) | >0.05 |
| L-spine T-score† | -2.6(1.2) | -2.5(1.1) | >0.05 |
| Femoral neck T-score† | -2.8(0.6) | -2.8(0.9) | >0.05 |
| TBS T-score† | -2.8(0.8) | -2.6(0.9) | >0.05 |
| BMI† | 25.1(5.9) | 27(4.9) | >0.05 |
| PINP (ng/ml)† | 54(33) | 47(28) | >0.05 |
| Vertebral fracture (%) | 75% | 65% | >0.05 |
| Hip fracture (%) | 23% | 17% | >0.05 |
| Number of fractures† | 3(2.2) | 3.3(2) | >0.05 |
| Previous osteoporosis therapy (%) | 63% | 82% | >0.05 |

† Mean (SD)

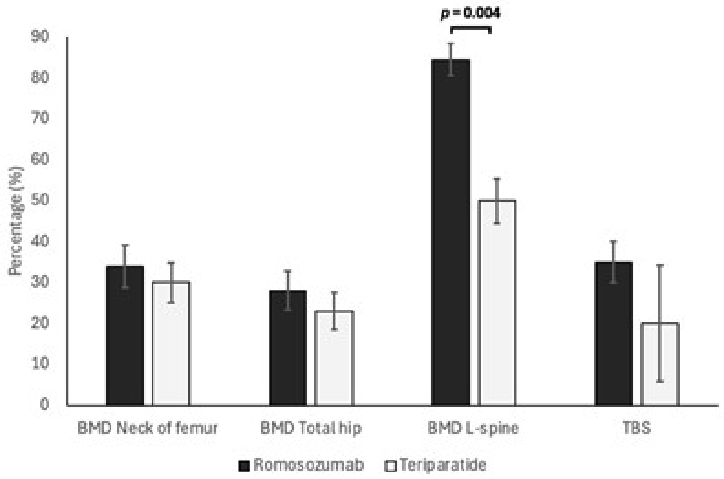

Figure 1. The proportions of patients with a response to Romosozumab or Teriparatide above the least significant change (LSC), defined as 3% at the lumbar spine, 4% at the total hip, 4.5% at the femoral neck (measured by Hologic DXA), and 3.79% for the trabecular bone score (TBS). Error bars represent standard errors.

**Poster Sessions: Bone bioengineering, regeneration and implants**

**P013**

**Impact of ceramic particle size on cellular response and progenitor recruitment in Osteogrow-C bone regeneration therapy**

*<u>Nikola Štoković</u>[1], Natalia Ivanjko[1], Nataša Kovačić[2], Danka Grčević[2], Slobodan Vukičević[1]*
*[1]University of Zagreb School of Medicine, Laboratory for Mineralized Tissues, Zagreb, Croatia*
*[2]University of Zagreb School of Medicine, Laboratory for Molecular Immunology, Zagreb, Croatia*

**Abstract Text**

**BACKGROUND/INTRODUCTION:** Osteogrow-C is a novel therapeutic solution for bone regeneration comprised of rhBMP6 delivered in autologous blood coagulum (ABC) with synthetic ceramics particles.

**PURPOSE:** To explore influence of ceramics particle size on the host cellular response following Osteogrow-C implantation.

**METHODS:** We have conducted rat subcutaneous implant assay study to characterize cellular reaction in early period (day 1 and 3) following implantation of rhBMP6/ABC with ceramic scaffold using flow cytometry. In this experiment we investigated how addition of small (SC,74-420µm) and large (LC,1000-4000µm) ceramic particles to rhBMP6/ABC affects host cellular response. Single cell suspensions were labelled with anti-rat Granulocyte-FITC, anti-rat CD11b-APC, anti-rat CD3-PE, anti-rat CD45RA-PECy7, anti-rat CD45-PE, anti-rat Erythroid Cells-PE, anti-rat CD31, purified anti-rat CD140a, purified anti-rat LepR, anti-rat CD26-FITC, anti-rat CD24-PECy7, anti-rat CD44-AF647, and anti-rat CD90-APCCy7, and in the second step goat anti-rabbit IgG-AF488, or goat anti-rabbit IgG-AF467. Animal study was approved by the National Ethics Committee.

**RESULTS:** Frequencies of myeloid lineage cells among single, live hematopoietic (CD45+) cells were higher in Osteogrow implants containing ceramics (SC/74-420 µm and LC/1000-4000 µm) than without ceramics (C). Frequencies of cells bearing mesenchymal (CD26,CD44), early osteochondroprogenitor (CD90,CD24) and comitted osteochondroprogenitor (CD140a,LepR) markers among single, live, non-hematopoietic (CD45–CD31–Ery–) cells were significantly increased in Osteogrow implants containing small ceramic particles than in Osteogrow implants with large particles or without ceramics.

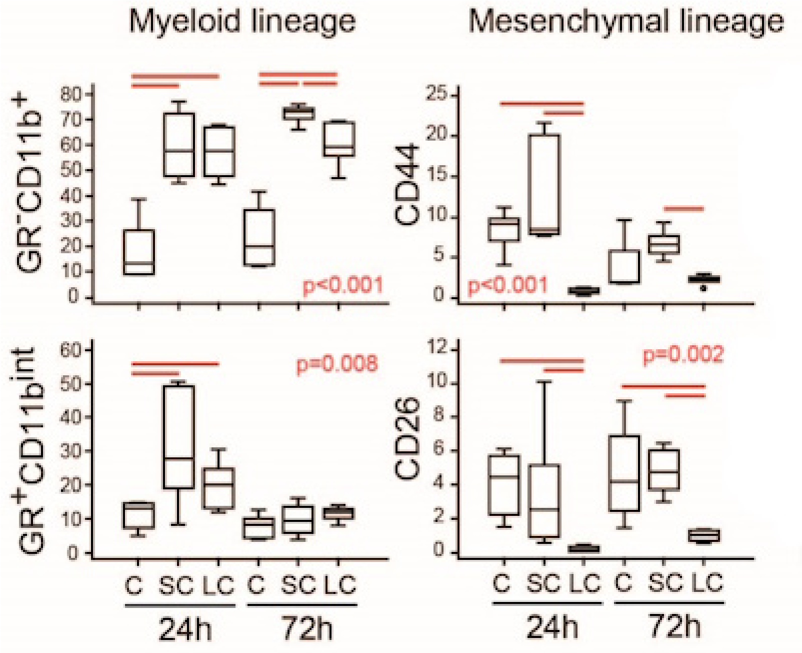

**CONCLUSION:** In conclusion, our study highlights impact of ceramic particle size on cellular responses following Osteogrow-C implantation. Specifically, the inclusion of small ceramic particles in the Osteogrow-C implants resulted in a marked increase in cells expressing mesenchymal and osteochondroprogenitor markers compared to larger ceramic particles or implants without ceramics. These findings suggest that optimizing ceramic particle size within Osteogrow-C could enhance the recruitment and differentiation of progenitor cells, potentially improving its effectiveness for bone regeneration applications.

**P014**

**Influence of different distraction regimes on femoral bone regeneration in a rat model**

*Katrin Bundkirchen*[1]*, Jan Moritz Ramge*[1]*, Stefan Lienenklaus*[2]*, Emmanouil Liodakis*[3]*,*
*Claudia Neunaber*[1]
[1]*Hannover Medical School, Department of Trauma Surgery, Hannover, Germany*

*[2]Hannover Medical School, Institute of Laboratory Animal Science, Hannover, Germany*
*[3]Saarland University, Department of Trauma- Hand and Reconstructive Surgery, Homburg/Saar, Germany*

**Abstract Text**

**Background/Introduction:** Huge bone defects are a major problem in orthopedic and trauma surgery. The "gold standard" in clinics for such defects is the callus distraction with a rate of 1 mm/day. This can be very time consuming and increases the risks for complications, as infections. Therefore, it is necessary further investigate effects of different distraction rates on bone regeneration.

**Purpose:** The aim of the project was the comparison of two new distraction regimes with the standard.

**Methods:** 12 weeks old, male Brown Norway rats received a diaphyseal osteotomy of the femur fixated externally. Seven days after operation, distraction was conducted over 12 days. Group 1 represented the clinical standard with constant distraction (0.75 mm/day) and therefore served as control. In group 2, the first six days distraction was "fast" (1 mm/day) and another six days "slow" (0.5 mm/day). In group 3, "fast" and "slow" distraction rates were inverted. The distracted regenerates were analyzed with µCT to determine bone morphometric parameters, density, and relative volume of hard, soft, and complete callus. Next, bone metabolism markers such as OPG and RANKL will be measured systemically within the plasma via ELISA. Molecular analyses of the regenerate will be performed via qRT-PCR and Western Blot, to investigate potential alterations in Wnt/β-catenin, BMP/SMAD and RANK/RANKL/OPG signaling. Histological analyses will reveal status of callus mineralization, cartilage, vascularization, osteoblasts, -clasts, IL-6 and β-Catenin.

**Results:** All animal experiments were completed successfully without differences between the distraction regimes in physiological parameters (activity, lameness, weight) or bone morphometric parameters. Currently, only the relative volume of the complete callus was significantly increased in group 3 (27,93±6,37%) on day 33 compared to group 1 (18,93±6,37%; p=0.0149).

**Conclusion:** Radiologically, the new regimens showed equally good results as the standard regimen, meaning that histological and molecular analyses could provide promising findings for clinical use.

**P015**

### Modeling Critical Size Defect Healing in Weight-Bearing Bones Using Autologous Bone Grafts in Mice

*Shahar Katz[1], Noam Levaot[1]*
*[1]Ben Gurion university, phisiology, beer sheva, Israel*

**Abstract Text**

Critical size defect (CSD) is a pathological condition in which a fracture gap is too large to heal spontaneously, resulting in persistent non-union. Autologous bone grafts (ABGs) remain the gold standard for treating such defects; however, their application is constrained by donor site morbidity and limited availability. Despite their extensive clinical use since the 19th century, the cellular and molecular mechanisms driving ABG-assisted healing remain poorly characterized.

In this study, we developed a novel mouse model combining osteotomy with plate and screw fixation to explore these mechanisms. For the first time, we utilized freshly harvested crushed bone paste as an autologous graft material to repair weight-bearing bone defects. Defects were either filled with crushed bone paste from GFP-expressing donor mice (graft+) or left empty (graft-).

Radiological analysis demonstrated that all graft- mice exhibited sclerotic atrophic nonunion, whereas the graft+ group showed substantial bone formation, with some cases achieving complete union. Using a CRYO-histology protocol enabling multiple stains on the same cryosection, we gained unprecedented insight into the histomorphometric dynamics of the healing process. The graft+ group exhibited enhanced osteoblastic and osteoclastic activity in the defect area, reduced scar tissue formation, bone hypertrophy around the plate, and overall increased bone regeneration. Notably, GFP signal detection confirmed donor cell survival in the defect zone.

This study represents the first application of crushed bone paste in a mouse model for CSD repair in weight-bearing bones such as the femur. It validates this model as a robust platform for investigating bone graft-assisted healing mechanisms and lays the foundation for developing improved graft substitutes for clinical use.

**P016**

**Advancing Bone Regeneration: Comparative Analysis of BMP-Based Osteoinductive Implants**

*Natalia Ivanjko*[1]*, Nikola Stokovic*[1]*, Marina Milesevic*[1]*, Slobodan Vukicevic*[1]
[1]*School of Medicine University of Zagreb, Laboratory for Mineralized Tissues, Zagreb, Croatia*

**Abstract Text**

**Introduction:** Infuse, a BMP-based osteoinductive device using rhBMP2 on absorbable collagen sponge (ACS), faces limitations due to ACS's compressibility and uncontrolled release, necessitating improved carriers. Osteogrow, utilizing rhBMP6 within autologous blood coagulum (ABC) as a carrier, is promising alternative but lacks sufficient biomechanical properties. A novel Osteogrow formulation (Osteogrow-C), combining rhBMP6/ABC with synthetic ceramic particles (SCP), aims to address these shortcomings.

**Purpose:** This study aims to compare release dynamics and osteoinductive properties of different BMP-osteoinductive implants.

**Materials and Methods:** The release profile of rhBMP2 from ACS and ABC was measured using an ELISA kit. Osteoinductive properties of Infuse (rhBMP2/ACS), Osteogrow (rhBMP6/ABC), and Osteogrow-C (rhBMP6/ABC/SCP) were evaluated in a rat subcutaneous assay. Bone formation was assessed at day 14 and 35 post-implantation using µCT imaging (SkyScan 1076) and histological sections.

**Results:** Cumulative release of rhBMP2 from ACS (85%) was higher than ABC over 72 hours. On day 14, Osteogrow and Osteogrow-C implants were completely ossified, whereas Infuse implants were only partially ossified, with the presence of ACS. Newly formed bone induced by Osteogrow-C was present at the ceramic surfaces, in the pores, and between the particles. By day 35, bone formation was evident in all implants, however, µCT analyses revealed Osteogrow-C's superiority over other formulations in bone volume and trabecular parameters. The structural properties of newly formed bone were similar to day 14, however, the number of adipocytes increased and became the predominant cell population in the bone marrow.

**Conclusions:** ABC demonstrated a sustained release of rhBMP2 over time compared to commercially available ACS. Furthermore, in vivo studies showed that ABC created a permissive environment for rapid bone formation compared to commercially available ACS. Furthermore, we showed that the novel Osteogrow-C formulation was superior to Infuse and Osteogrow, inducing the highest bone volume on observed time points.

**P017**

**Rational engineering of glycosaminoglycan-based dickkopf-1 scavengers to improve bone regeneration**

*Juliane Salbach-Hirsch*[1], *Gloria Ruiz-Gomez*[2], *Jan-Niklas Dürig*[3], *Linda Köhler*[4], *Kanagasabai Balamurugan*[2], *Sandra Rother*[4], *Sophie Heidig*[2], *Stephanie Möller*[5], *Matthias Schnabelrauch*[5], *Giulia Furesi*[1], *Sophie Pählig*[1], *Pedro M Guillem-Gloria*[2], *Christine Hofbauer*[6], *Vera Hintze*[4], *M. Teresa Pisabarro*[2], *Jörg Rademann*[3], *Lorenz C Hofbauer*[1]
[1]*Technische Universität Dresden, Medical Faculty- BoneLab, Dresden, Germany*
[2]*Technische Universität Dresden, BIOTEC-Structural Bioinformatics, Dresden, Germany*
[3]*Freie Universität Berlin, Institute of Pharmacy–Medicinal Chemistry, Berlin, Germany*
[4]*Technische Universität Dresden, Institute of Materials Science- Max Bergmann Center of Biomaterials, Dresden, Germany*
[5]*INNOVENT e.V., Biomaterials Department, Jena, Germany*
[6]*Technische Universität Dresden Medical Center, National Center for Tumor Diseases & University Cancer Center Dresden, Dresden, Germany*

**Abstract Text**

**Background:** The WNT signaling pathway is a central regulator of bone development and regeneration. Functional alterations of WNT ligands and inhibitors are associated with a variety of bone diseases that affect bone fragility and result in a high medical and socioeconomic burden. Hence, this cellular pathway has emerged as a novel target for bone-protective therapies, e.g. in osteoporosis.

**Purpose:** Here we investigated glycosaminoglycan (GAG) recognition by Dickkopf-1 (DKK1), a potent endogenous WNT inhibitor, and the underlying functional implications in order to develop WNT signaling regulators.

**Methods:** In a multidisciplinary approach we applied *in silico* structure-based *de novo* design strategies and molecular dynamics simulations combined with synthetic chemistry and surface plasmon resonance spectroscopy to rationally engineer oligomeric glycosaminoglycan derivatives ($_{RE}$GAG) with improved neutralizing properties for DKK1.

**Results:** *In vitro* Alpl gene expression, Wnt reporter assay and *in vivo* analyses showed that the $_{RE}$GAGs modification translated into increased WNT pathway activity [+20%; p<0.05 and +40%; p<0.05 respectivly] and improved bone regeneration in a mouse calvaria defect model with critical size bone lesions. Importantly, the developed $_{RE}$GAG outperformed their precursor, polymeric high-sulfated hyaluronan (sHA3), in enhancing bone healing up to 50% due to their improved DKK1 binding properties.

**Conclusion:** Thus, rationally engineered GAG variants may represent an innovative strategy to develop novel therapeutic approaches for regenerative medicine.

**P018**

**Novel approach for osteomyelitis treatment: antimicrobial biomimetic hybrid scaffolds to control infection and stimulate bone tissue regeneration**

*Francesca Romana Liberati[1], Sara Bernardoni[2], Valentina Granata[3], Valentina Possetti[1], Elisabetta Campodoni[2], Barbara Bottazzi[1], Alberto Mantovani[1], Cristina Sobacchi[3], Antonio Inforzato[1], Monica Sandri[2]*
*[1]Fondazione Humanitas per la Ricerca, Department of Biomedical Sciences, Rozzano Milan, Italy*
*[2]National Research Council,*
*Institute of Science- Technology- and Sustainability for the Development of Ceramic Materials ISSMC, Faenza, Italy*
*[3]National Research Council, Institute for Genetic and Biomedical Research, Milan, Italy*

**Abstract Text**

**Background:** Osteomyelitis is a bone inflammation caused by bacterial infections, particularly *Staphylococcus aureus* (SA). Treatment involves surgery and antibiotics; however, their efficacy is hampered by many factors[1].

**Purpose**: To develop and test a novel biomimetic and bioresorbable implant with antimicrobial properties.

**Methods:** synthesis of copper-doped hydroxyapatite-collagen (CuHA/Col) scaffolds as described[2], with 0.5,1 or 2% copper ions ($Cu^{++}$) as antimicrobial agent[3]. Colony-forming unit (CFU) counting after SA seeding on scaffolds. MTT assays following MC3T3-E1 contact with scaffolds' conditioned medium (CM) and ALAMAR blue assays on MC3T3-E1 seeded on scaffolds for biocompatibility assessment. All experiments were performed without and with scaffold conditioning (overnight to one week). N≥3 experiments.

**Results**: 1% and 2% CuHA/Col scaffolds had pronounced antimicrobial activity at 24 hours from SA seeding already (1.6±0.0x10$^6$ and 2.0±0.1x10$^6$ CFU/scaffold, respectively, vs control HA/Col 2.2 ±0.6x10$^9$ CFU/scaffold; **A)**; however, the MTT assay indicated reduced cell viability (**B**, e.g. 72h exposure: 1% and 2% CuHA/Col scaffolds 7.3±0.1x10$^3$ and 4.6±0.2x10$^3$ FI/well, respectively, vs control HA/Col 2.0±0.0x10$^5$ FI/well, p<0.001, Two-way ANOVA). This suggests that the $Cu^{++}$ released from the scaffold upon implantation might guarantees disinfection of the site, but not efficient osteoblasts growth.

In the conditioning setting, $Cu^{++}$ release, measured by inductively coupled plasma-optical emission spectrometry (ICP-OES), was rapid in the first 24 hours, slowing afterward, with 85% $Cu^{++}$ remaining after 168 hours. Nevertheless, CuHA/Col scaffolds maintained strong antibacterial effects (**C**). Cells exposed to the scaffolds' CM had similar viability to controls (**D**), regardless of conditioning duration. MC3T3-E1 cells seeded on 96- and 168-hour-conditioned 1% and 2% CuHA/Col scaffolds had comparable viability to controls (**E**), suggesting that initial release of $Cu^{++}$may enhance biocompatibility of doped-scaffolds.

**Conclusions**: This study presents the development of CuHA/Col scaffolds for osteomyelitis treatment that balance antimicrobial efficacy with biocompatibility by optimizing copper ion release to ensure effective infection control and long-term tissue regeneration.

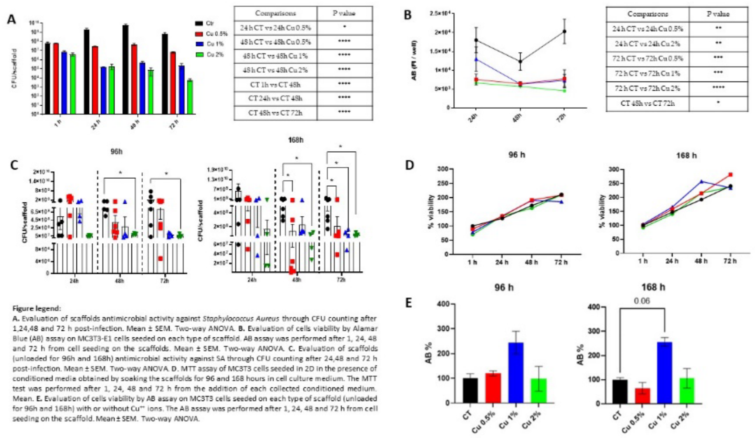

**Figure legend:**
**A.** Evaluation of scaffolds antimicrobial activity against *Staphylococcus Aureus* through CFU counting after 1,24,48 and 72 h post-infection. Mean ± SEM. Two-way ANOVA. **B.** Evaluation of cells viability by Alamar Blue (AB) assay on MC3T3-E1 cells seeded on each type of scaffold. AB assay was performed after 1, 24, 48 and 72 h from cell seeding on the scaffolds. Mean ± SEM. Two-way ANOVA. **C.** Evaluation of scaffolds (unloaded for 96h and 168h) antimicrobial activity against SA through CFU counting after 24,48 and 72 h post-infection. Mean ± SEM. Two-way ANOVA. **D.** MTT assay of MC3T3 cells seeded in 2D in the presence of conditioned media obtained by soaking the scaffolds for 96 and 168 hours in cell culture medium. The MTT test was performed after 1, 24, 48 and 72 h from the addition of each collected conditioned medium. Mean. **E.** Evaluation of cells viability by AB assay on MC3T3 cells seeded on each type of scaffold (unloaded for 96h and 168h) with or without $Cu^{++}$ ions. The AB assay was performed after 1, 24, 48 and 72 h from cell seeding on the scaffold. Mean ± SEM. Two-way ANOVA.

**P019**

**HOBBIT - Highly porous bioinert bone implants for non-union fracture healing**

Regina Brunauer[1]
[1]*LBG Ludwig Boltzmann Institute for Traumatology, Bone, Wien, Austria*

**Abstract Text**

**Background:** Non-union fractures fail to heal on their own. The current gold standard, treatment with autologous bone grafts from the iliac crest, is difficult and often not possible. HOBBIT aimed to develop a novel type of macro-porous implants that are based on high-strength, biocompatible ceramics as a treatment alternative. The goal of this project was to investigate the potential of zirconia ($ZrO_2$) and alumina ($Al_2O_3$) as substrates for minimal-volume high-performance ceramics to serve as load-bearing scaffolds and to examine the bone healing potential of a minimal volume, load-bearing scaffold combined with a gene-activated matrix, using either non-viral gene delivery and mesenchymal stromal cells (MSCs) or extracellular vesicles.

**Methods:** Ceramics were tested for biocompatibility in vitro and for mechanical stability for in vivo application. Load-bearing and regenerative potential of ceramics were evaluated in a rat femur critical size defect model of 5mm. To improve bone regeneration and prevent soft tissue ingrowth silk fibroin tubes were introduced. µCT scans were performed after 3d, 2w, 4w, 6w and 8w for $Al_2O_3$. Hard tissue histology was performed after 8w for $Al_2O_3$ and $ZrO_2$.

**Results:** Both ceramics did not present toxicity in vitro and showed and high mechanical performance. However, $Al_2O_3$ scaffolds did not provide sufficient load-bearing in vivo, resulting in breaking during the 8w observation period. $ZrO_2$ implants facilitated stability in the defect, and soft tissue ingrowth was prevented with silk fibroin tubes to allow bone formation through the whole scaffold.

**Conclusion:** Alterations in design and ceramic material improved stability and regeneration in the rat femur defect significantly over the 8W observation period. However, our results demonstrate the need for further elucidation regarding the introduction of a pro-regenerative matrix within the scaffolds to potentially enhance bone regeneration of patients.

**P020**

**Bone substitute Perossal® modulates bone marrow mesenchymal stem cell immunomodulatory properties**

*Aleksandra Jauković[1], Slavko Mojsilović[2], Tamara Kukolj[2], Hristina Obradović[2], Milena Živanović[2], Sebastian Häusner[3], Lazar Mičeta[4], Duško Spasovski[4], Torsten Blunk[5], Marietta Herrmann[6]*
*[1]Institute for Medical Research- University of Belgrade, Group Hematology and Stem Cells, Belgrade, Serbia*
*[2]Institute for Medical Research- University of Belgrade, Group for Hematology and Stem Cells, Belgrade, Serbia*
*[3]University Hospital of Würzburg- Bernhard-Heine-*
*Center for locomotion research- University of Würzburgg,*
*IZKF Group Tissue Regeneration In Musculoskeletal Diseases, Würzburg, Germany*
*[4]Institute of Orthopedy "Banjica", Institute of Orthopedy "Banjica", Belgrade, Serbia*
*[5]University Hospital Würzburg, Department of Trauma-- Hand-*
*- Plastic- and Reconstructive Surgery Surgery II, Würzburg, Germany*
*[6]University Hospital of Würzburg- Bernhard-Heine-*
*Center for locomotion research- University of Würzburg,*
*IZKF Group Tissue Regeneration In Musculoskeletal Diseases, Würzburg, Germany*

**Abstract Text**

Bone substitutes are commonly used to treat bone defects. Moreover, their combined usage with mesenchymal stromal/stem cells (MSC) improves regeneration of large bone defects increasing osteoinduction. One of the bone substitutes used for treatment of infected bone defects as antibiotic carrier is PerOssal®, composed of nanocrystalline hydroxyapatite and calcium sulfate. Combined usage of PerOssal® with MSC has not been investigated so far. Here we studied *in vitro* whether PerOssal® influences MSC in terms of bone tissue regeneration associated with immunomodulation.

Bone marrow MSC were isolated from femur of patients undergoing hip replacement surgery (Ethical approval I-97/14.3). Cell growth/viability and apoptosis were determined by MTT and flow cytometry (FC).  Immunomodulatory potential of MSC was evaluated using functional *in vitro* assay. Secretome was assessed by FC (multiplex assay) while gene expression by qPCR.

Our results demonstrated that short-term treatment with PerOssal® at all dilutions tested (2000x, 200x, 100x) did not modify MSC growth, while at highest dose it decreased MSC viability by 20% at day 7 (n=5). However, no significant changes in apoptotic MSC percentage were found. Analyses of immunomodulatory potential of PerOssal®-treated MSC showed increased secretion of various inflammatory cytokines after 3 days, including TNFα (p=0.003), IL-17 (p=0.019), IL-23 (p=0.03), IFN-γ (p=0.001), IL-1β (p=0.005) and IL-8 (p=0.023) (n=3) that was also detected at gene level. Since bone regeneration is driven by MSC-immune cell interactions, effects of conditioned media (CM) from PerOssal®-treated MSC on peripheral blood lymphocyte proliferation were tested. Data revealed increased mitogen-activated proliferation of CD4+ lymphocytes by CM of PerOssal®-treated MSC (200x, 100x), while decreased spontaneous proliferation of CD4+ and $CD4^{+}CD25^{+}$ cells.

These findings imply that PerOssal® modulates MSC immunomodulatory properties and secretory profile. Further research will define PerOssal® impact on MSC-immune cells interactions and their importance in bone repair processes.

**Poster Sessions: Bone biomechanics and quality**

**P021**

**Effects of the Amount of Proximal Tibia Resection on the BonenStrength of Prepared Bone Surface: A FEM Study**

*Kwangkyoun Kim*[1]
[1]*Konyang university hospital, Orthopedic surgery, Daejeon, Korea- South*

**Abstract Text**

**Purpose:** The aim of this study is to evaluate the mechanical strength of proximal tibia as resection distance increased from the joint surface.

**Materials and Methods:** We obtained the CT images of twenty knee osteoarthritis patients undergoing total knee arthroplasty. The finite element models were created based on the computed tomography images. The 8-node hexahedron element was made from BIONIXTM (CANTIBio. Co, Suwon, Korea), which is automatic mesh generation software program. The finite element model of the proximal tibia was resected at 6 mm, 8 mm, 10 mm, 12 mm, 15 mm and 18 mm from the lateral joint surface. A 1% strain rate was applied to a model by using HyperMeshTM software (Altair Engineering. Inc, Seattle, USA). The ultimate stress was calculated from the finite element analysis with using ANSYS 9.0 (ANSYS. Inc, Orlando, USA). Results: The mean ultimate stress was 906.84 MPa, 877.22 MPa, 895.93 Mpa, 852.70 MPa, 742.90 Mpa and 585.51 Mpa at the 6 mm, 8 mm, 10 mm, 12 mm, 15 mm and 18 mm resection levels. As compare to the 6 mm resection level, the bone strengths at 15 mm and 18 mm were decreased with statistical significance (15 mm: p=0.005, 18 mm: p=0.000).

**Conclusion:** The ultimate stress was decreased as the resection distance increased from the joint surface. But within a 12 mm resection distance from the lateral condyle articular surface of the tibia, the ultimate stress was not significantly decreased ($p > 0.05$).

**P022**

**Association of Visceral Adiposity with Bone Quality in Different Prediabetes Subtypes: Independent Effects Beyond Insulin Resistance**

*Yongjun Choi*[1], *Ja Young Jeon*[1], *Kyoung Hwa Ha*[1], *Dae Jung Kim*[1]
[1]*Ajou University School of Medicine, Endocrinology and Metabolism, Suwon, Korea- South*

**Abstract Text**

**Background:** The relationship between prediabetes subtypes and bone quality remains unclear, particularly regarding the role of visceral adiposity and insulin resistance.

**Purpose:** We investigated associations between prediabetes subtypes, visceral adiposity, and bone microarchitecture measured by trabecular bone score (TBS).

**Methods:** This cross-sectional study included 1004 males and 1223 postmenopausal females (aged 40-64 years) from the Cardiovascular and Metabolic Diseases Etiology Research Center Cohort Study. Participants underwent whole-body DXA and 75g oral glucose tolerance tests, being classified into normal glucose tolerance (NGT), impaired fasting glucose (IFG), impaired glucose tolerance (IGT), or combined IFG and IGT groups. Visceral adipose tissue (VAT) mass was quantified using DXA. Sex-stratified analyses were performed with adjustments for confounders.

**Results:** The IGT group (1.462, 95% confidence interval (CI):1.442-1.482) showed significantly lower TBS compared to other groups, particularly in men. In men, IGT subjects showed significantly lower TBS than the NGT group (1.491. 95% CI: 1.484-1.497) after adjusting for multiple confounders, including subcutaneous fat (p=0.008). However, this difference was attenuated after additional adjustment for visceral fat (p=0.156), suggesting mediation through visceral adiposity. In postmenopausal women, no significant differences in TBS were observed among prediabetes subtypes, although IGT showed a trend toward lower TBS compared to IFG. The negative association between VAT mass and TBS persisted after controlling for insulin resistance parameters, suggesting independent effects of VAT on bone quality in both men and postmenopausal women ($p<0.001$). The relationship between VAT mass and TBS demonstrated potential non-linear patterns in women, with a threshold effect observed at about 1.0 kg of VAT mass.

**Conclusion:** Our findings demonstrate that IGT is associated with compromised bone quality, primarily mediated through increased visceral adiposity. The robust, independent association between VAT mass and TBS highlights the importance of visceral adiposity assessment in assessing bone quality in prediabetes.

**P023**

**Osteoporosis assessment using cancellous bone density measurement in Hounsfield units in the femoral native CT cross-section**

*Julian Ramin Andresen*[1], *Guido Schröder*[2], *Thomas Haider*[1], *Hans-Christof Schober*[3], *Reimer Andresen*[4]
[1]*Medical University of Vienna, University Clinic for Orthopaedics and Trauma Surgery, Vienna, Austria*
[2]*University Medicine Rostock, Clinic for Trauma- Hand and Reconstructive Surgery, Rostock, Germany*
[3]*Practice for Orthopaedics and Osteology, OrthoCoast, Wolgast, Germany*
[4]*Westküstenklinikum Heide- Academic teaching Hospital of the Universities of Kiel- Lübeck and Hamburg, Institute for Diagnostic and Interventional Radiology / Neuroradiology, Heide, Germany*

**Abstract Text**

**Introduction:** This study aims to investigate whether osteoporosis in the hip can be estimated using density measurements in Hounsfield units (HU) and if quantitative BMD- and T-values can be derived from HU values.

**Methods:** We examined 240 patients (mean age 64.9 ± 13.1 years, BMI 26.8 ± 6.8 kg/m²), including 37 men (mean age 60.0 ± 14.3 years, BMI 28.4 ± 5.7 kg/m²) and 188 women (mean age 65.8 ± 12.7 years, BMI 26.5 ± 7.0 kg/m²). Osteoporosis presence was assessed using computed tomography X-ray absorptiometry (CTXA) for BMD and T-values. Cancellous density in HU was measured in the caput femoris, collum femoris, and pertrochanteric region using circular ROIs in coronal CT sections. The mean values for these regions were used to represent the entire proximal femoral region.

**Results:** A significant ($p < 0.05$) decrease in cancellous bone density was observed with increasing age and decreasing BMI. The median BMD for the entire hip was 0.684 (0.306 - 1.368) mg/cm², and the median HU for the proximal femur was 125.68 (-11.93 - 308.67). A strong correlation ($R^2 = 0.8998$, $p < 0.001$) was found, allowing BMD to be estimated from HU using the formula: $Xctxa_{Hip} = 0.364 + 0.0027 \times HU$. The median T-value was -2.06 (-5.15 - 3.78), with a correlation ($R^2 = 0.9075$, $p < 0.001$) enabling T-values to be calculated from HU using the formula: $Xt_{total} = -4.8717 + 0.0234 \times HU$ A T-value of -2.5 corresponds to an HU value of 101.35. A T-value of -2.5 corresponds to an HU value of 243.2 for the caput femoris, 29.18 for the collum femoris and 23.92 for the pertrochanteric region.

**Conclusion:** HU values can be converted into quantitative BMD- and T-values, providing a reliable method for estimating osteoporosis in the hip.

**P024**

**Bone Quality in Chronic Kidney Disease Patients with and without Diabetes Mellitus**

*Syazrah Salam[1], Sofie Kolibová[2], Lilian Gabel[2], Orla Gallagher[1], Richard Eastell[1], Björn Busse[2], Katharina Jähn-Rickert[2]*
*[1]University of Sheffield, Division of Clinical Medicine, Sheffield, United Kingdom*
*[2]University Medical Center Hamburg-Eppendorf, Department of Osteology and Biomechanics, Hamburg, Germany*

**Abstract Text**

**Background:** Fracture risk is high in patients with advanced chronic kidney disease (CKD) and in patients with diabetes mellitus (DM). Abnormalities in bone mineral density alone have not fully explained the high fracture risk in both disease conditions; suggesting that poor bone quality may play a role in their bone fragility. The effects on bone quality and fracture risk when both diseases co-exist is unknown.

**Purpose:** To assess if DM has differential effects on bone quality in advanced CKD patients.

**Methods:** CKD4-5D patients with (n=11) and without DM (n=22) were recruited. The study was approved by the local research ethics committee. Quantitative bone histomorphometry on trans-iliac bone biopsy samples was performed to determine bone turnover, mineralization, and volume. Bone quality was evaluated using quantitative backscattered electron imaging to assess bone mineral density distribution (BMDD) and 2D morphological analysis of osteocyte lacunae. The osteocyte-lacunae network was visualised and quantified with silver staining on LR white resin sections. Additionally, bone matrix composition was analysed using vibrational Raman spectroscopy to determine the mineral-to-matrix ratio.

**Results:** Trabecular bone volume/tissue volume (BV/TV) was lower in CKD+DM group (17.5 [13.9 – 19.4]% vs. 21.6 [18.7 – 26.1]%, p=0.02). There were no differences in bone formation, resorption and mineralization parameters on bone histomorphometry between the groups. We observed no difference in the regulatory osteocyte network. With respect to mineralization, there were no differences in BMDD. Finally, Raman spectroscopy did not show differences in bone matrix composition apart from a higher phosphate/amide III peak in the cortical bone in CKD+DM (1.42 ± 0.15 vs 1.32 ± 0.11 a.u., p=0.04), which might indicate altered organic matrix organization.

**Conclusion:** There were no differences in bone material properties in advanced CKD patients with and without DM. However, CKD patients with DM had lower trabecular bone volume which may have detrimental effects on their bone health.

**P025**

**Correlation analysis of small-animal DXA for bone parameters and total body, fat and lean mass**

Tiina Kähkönen[1], Yeeun Kim[2], Jungyun Yeu[3], Sunghyun Min[3], Jussi Halleen[1]
[1]OncoBone Ltd, Na, Oulu, Finland
[2]DT&CRO, Efficacy Evaluation Center, Gyeonggi-do, Korea- South
[3]Osteosys, R&D center, Seoul, Korea- South

**Abstract Text**

Dual-energy X-ray absorptiometry (DXA) can be used for analysing bone and body composition in laboratory animals. Unlike commonly used traditional extraction methods such as ash weight and crude fat weight measurements, DXA can be used for longitudinal monitoring of live anesthetized animals during the study.

Purpose of this study was to validate iNSiGHT small-animal DXA in measuring Bone Mineral Content (BMC) and Density (BMD), fat mass (FM) and lean mass (LM) in mice and rats, and to confirm their correlation to traditionally used extraction methods.

Eighteen ICR mice and Sprague-Dawley rats were divided into three weight groups (20 g, 30 g and 40 g for mice, and 100 g, 200 g and 300 g for rats; n=6 in each group). Body weight, bone ash weight, crude fat weight and lean weight were measured using a scale and compared with body weight, total body (TB) BMC, fat mass and lean mass, respectively, measured by iNSiGHT DXA. Femur BMC was measured by iNSiGHT DXA before and after euthanasia and compared to values obtained by µCT.

The obtained DXA values showed strong correlation with actual body weight, fat and lean mass. BMC obtained with DXA showed strong correlation with BMC calculated from µCT analysis and with bone ash weight in both live and euthanized animals. Similar high correlations were observed in both mice and rats, demonstrating that DXA can be used to analyze reliably changes in the above mentioned parameters in commonly used small laboratory animals.

| | *Anesthetized* | | | | | *Euthanized* | | | | |
|---|---|---|---|---|---|---|---|---|---|---|
| **Pearson correlation** | **Body weight** | **FM** | **LM** | **TB BMC** | **Femur BMC** | **Body weight** | **FM** | **LM** | **TB BMC** | **Femur BMC** |
| **Mouse** | 0.9995 | 0.9702 | 0.9964 | 0.9990 | 0.9660 | 0.9926 | 0.9804 | 0.9759 | 0.9945 | 0.9806 |
| **Rat** | 0.9996 | 0.9961 | 0.9999 | 0.9991 | 0.9964 | 0.9996 | 0.9934 | 0.9962 | 0.9982 | 0.9919 |

Table 1. Summary of Pearson correlations between DXA and traditionally used methods.

**P026**

**Deteriorated trabecular micro-architecture in lumbar vertebrae of women with intravenous heroin addiction: potential implication for compromised bone strength**

Jelena Jadzic[1], Nada Tomanovic[2], Aleksa Lekovic[3], Slobodan Nikolic[3], Marija Djuric[1], Danijela Djonic[1]
[1]Faculty of Medicine- University of Belgrade, Center of Bone Biology, Belgrade, Serbia
[2]Faculty of Medicine- University of Belgrade, Institute of Pathology, Belgrade, Serbia

[3]*Faculty of Medicine- University of Belgrade, Institute of Forensic Medicine, Belgrade, Serbia*

**Abstract Text**

**Background:** Previous studies suggested that intravenous heroin addiction (IHA) could be associated with various skeletal conditions, including low bone mineral density. Still, micro-scale bone features were not previously analyzed in women prone to heroin abuse.

**Purpose:** We aimed to conduct a *postmortem* assessment of trabecular and cortical micro-architecture in the anterior mid-transverse portion of the first lumbar vertebral body collected from adult female cadavers with IHA.

**Methods:** A total of 24 one-cm-thick vertebral samples were analyzed using the Skyscan 1172 micro-CT system (Bruker, Belgium). Based on macroscopic stigmata of intravenous heroin abuse and pathohistological analysis of liver tissue, bone samples were divided into the IHA group (n=14, age range: 29-85 years) and the control group (n=10, age range: 28-85 years).

**Results:** As shown in the figure, decline in trabecular bone volume fraction due to lower trabecular thickness and higher trabecular separation was noted in the IHA group compared to controls (Student t-test, $p<0.05$). Despite the declining trend in cortical micro-architectural parameters, we did not note significant differences in the vertebral cortex of women with IHA compared to the control group (Student t-test, $p>0.05$).

**Conclusions:** Our data revealed predominant trabecular micro-architectural impairment in the vertebral body of women prone to heroin abuse, while the cortical bone was not severely affected. Further studies with a more detailed analysis of bone quality features are necessary to fully understand whether heroin abuse contributes to perimenopausal skeletal alterations in women.

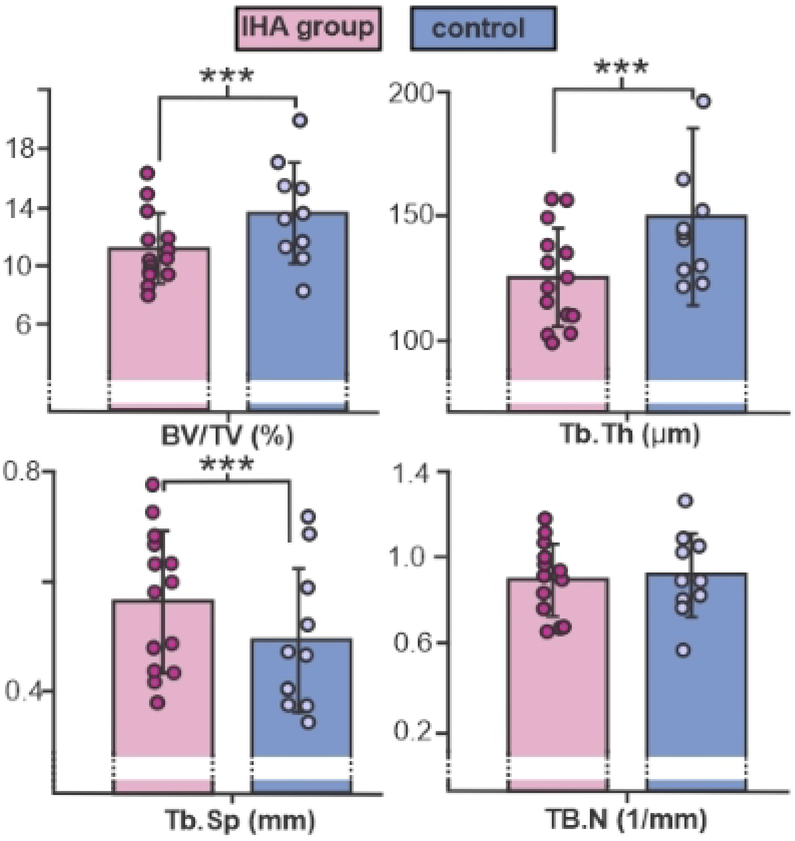

**P027**

**Deteriorated micro-scale vertebral properties in men with class 1 obesity: postmortem bone quality assessment**

*Jelena Jadzic*[1], *Aleksa Lekovic*[2], *Petar Milovanovic*[1], *Slobodan Nikolic*[2], *Bjorn Busse*[3], *Marija Djuric*[1]
[1]*Faculty of Medicine- University of Belgrade, Center of Bone Biology, Belgrade, Serbia*
[2]*Faculty of Medicine- University of Belgrade, Institute of Forensic Medicine, Belgrade, Serbia*
[3]*University Medical Center Hamburg-Eppendorf, Department of Osteology and Biomechanics, Hamburg, Germany*

**Abstract Text**

**Background:** Recent studies suggest that various skeletal sites could be differently affected in individuals with obesity, while their micro-scale bone quality features have not been thoroughly investigated so far.

**Purpose:** We aimed to conduct a postmortem micro-scale bone quality assessment of the anterior mid-transverse portion of the first lumbar vertebral body collected from adult male cadavers with class 1 obesity (body mass index [BMI]>30kg/$m^2$, ) and normal-weight counterparts (BMI between 20 kg/$m^2$ and 25 kg/$m^2$).

**Methods:** With the institutional review board approval, one cm-thick vertebral bone samples were collected during autopsies from 23 men divided into the Ob group (n=10, age: 65±12 years) and age-matched control group (n=13, age: 61±5 years). Micro-computed tomography, Vickers micro-hardness testing, and scanning electron microscopy were done to assess micro-scale bone quality features in these individuals.

**Results:** Our groups did not significantly differ in age, body mass index, and hip bone mineral density (p>0.05). Our data revealed predominant micro-architectural impairment in the vertebral trabecular compartment of the Ob group (decline in trabecular bone fraction [p=0.028] due to lower trabecular thickness [p=0.019] and higher trabecular separation [p=0.035]), while cortical micro-architectural parameters were not significantly affected in these individuals (p>0.05). Despite the declining trend in mechanical properties of cortical and trabecular bone (p=0.069 and p=0.053, respectively), Vickers micro-hardness was not significantly different between the Ob group and controls. Lastly, our data revealed mild alteration in the morphology of the osteocyte lacunar network and Haversian system in the Ob group compared to controls (p<0.05).

**Conclusions:** Trabecular microstructural alterations are a hallmark of skeletal damage in men with class 1 obesity, which could be associated with increased vertebral bone fragility. Further studies with a more detailed bone quality analysis are necessary to understand this topic entirely.

**P028**

**Association between back pain and bone microarchitecture in women with rheumatoid arthritis**

*Maria Kozyreva[1], Nikolay Demin[1], Natalia Toroptsova[1]*
*[1]V.A. Nasonova Research Institute of Rheumatology, osteoporosis, Moscow, Russia C.I.S.*

**Abstract Text**

**Objective:** to assess the bone microarchitecture using the trabecular bone score (TBS) and its association with back pain in women with rheumatoid arthritis (RA).

**Material and methods:**128 women with a confirmed RA were divided into two groups: I - 100 people (mean age 58.8±11.9 years) with back pain according to VAS≥40 mm, II - 28 persons (mean age 57.8±10.6 years) with back pain according to VAS<40 mm. All patients underwent questionnaires, dual-energy X-ray absorptiometry (DXA) of the lumbar spine (Lunar Prodigy Advance, USA). The bone microarchitecture was assessed depending on the TBS level: degraded - TBS≤1.23, partially degraded – TBS 1.23 - <1.31, normal microarchitecture - TBS≥1.31. Lateral vertebra assessment (LVA) was performed. The study was approved by the local ethics committee.

**Results:** low TBS was detected in 29%, partially degraded microarchitecture in 20% and normal microarchitecture in 51% of the examined patients. In group I, the median TBS was 1.31 [1.21; 1.40], and in group II - 1.32 [1.22; 1.43], p>0.05. Low TBS was observed in 28% of patients in group I and in 32% in group II (p>0.05). In group I, vertebral fractures (VF) were diagnosed in 14 (14%) individuals, and in group II – in 1 (3.6%) patient (p>0.05). Among patients with VF, the mean level of back pain was 69.1 [49.5; 77.5] mm, and in women without fractures - 30.5 [20.0; 50.0] mm, p<0.05. Low TBS was significantly more common in patients with VF, and normal bone microarchitecture - in patients without VF (p<0.05).

**Conclusion.** Degraded bone microarchitecture was found in 29% of patients with RA. No association was observed between the TBS and the severity of back pain. Significant differences in back pain were found in patients with VF compared to those without them. Low TBS was significantly more common in patients with VF than in patients without them.

**P029**

**Relationship of bone microarchitecture with clinical and laboratory parameters in women with rheumatoid arthritis**

*Maria Kozyreva[1], Nikolay Demin[1], Oksana Nikitinskaya[1], Natalia Toroptsova[1]*
*[1]V.A. Nasonova Research Institute of Rheumatology, osteoporosis, Moscow, Russia C.I.S.*

**Abstract Text**

**Objective:** to establish the frequency of damaged bone microarchitecture according to trabecular bone score (TBS) and to evaluate the relationship with biochemical parameters in women with rheumatoid arthritis (RA)/

**Material and methods:** 183 women with a confirmed RA (mean age 60.3±10.0 years) were included. Questionnaires, laboratory examination (determination of erythrocyte sedimentation rate (ESR), glucose, total cholesterol, uric acid, creatinine, total protein, parathyroid hormone (PTH), albumin, alkaline phosphatase (ALP), calcium and phosphorus levels); dual-energy X-ray absorptiometry (DXA) of the lumbar spine were performed. TBS calculation of was done using TBS Insight software, version 3.0. The study was approved by the local ethics committee.

**Results:** According to TBS data, microarchitecture was normal in 85 (46.4%) women, partially degraded in 40 (21.9%), and degraded in 58 (31.7%) individuals. Negative associations were found between TBS and age ($r=-0.36$, $p<0.0001$), duration of postmenopausal period ($r=-0.17$, $p<0.05$), PTH level ($r=-0.29$, $p<0.05$), ALP level($r=-0.22$, $p=0.007$), as well as a positive association with phosphorus level ($r=0.29$, $p<0.05$). No associations with other biochemical parameters were found. Univariate linear regression analysis confirmed the relationship between TBS and age ($\beta = -0.36$ $p < 0.0001$), duration of postmenopausal period ($\beta = -0.17$ $p < 0.05$), PTH ($\beta = -0.30$, $p < 0.05$), ALP ($\beta = -0.25$ $p = 0.004$) and phosphorus ($\beta = 0.32$, $p < 0.05$) levels in women with RA.

**Conclusion:** Bone microarchitecture as measured by TBS was inversely associated with age, duration of postmenopausal period, PTH and ALP levels, and directly associated with phosphorus levels.

**P030**

**Prevalence of Sarcopenia in CKD: Insights into the Role of Nutrition and Dialysis**

*Federica Giambò[1], Nadia Edvige Foligno[1], Matteo Brambilla Pisoni[1], Giuseppe Vezzoli[1]*
*[1]IRCCS San Raffaele Hospital- Vita Salute San Raffaele University,*
*Department of Nephrology and Dialysis, Milan, Italy*

**Abstract Text**

**Purpose:** The study aimed to compare the prevalence of sarcopenia, body composition, and nutritional intake between dialysis patients (DP) and those with conservatively managed chronic kidney disease (CKD) in stages IV and V, including patients with End-Stage Renal Disease (ESRD).

**Methods:** A total of 92 dialysis patients (DP: 65 men, 27 women) and 36 conservatively managed patients were included. Sarcopenia was defined using bioimpedance analysis, with the Appendicular Skeletal Muscle Index (ASMI, appendicular muscle mass [kg]/height² [m²]) set at <7 kg/m² for men and <5.5 kg/m² for women. Body composition (BMI, ASMI, lean mass, fat mass) and nutritional intake were assessed, with nutrient intake analyzed using food frequency questionnaires.

**Results:** Among dialysis patients, 12% had a BMI <18.5, 47.8% had a BMI between 18.5–25, 30.4% between 25–30, and 9.8% had a BMI >30. Only 53.5% met the caloric target of 25 kcal/kg/day, and just 17.4% reached the recommended protein intake of 1.2 g/kg/day.

Sarcopenia was significantly more prevalent in dialysis patients (78.2%) compared to conservatively managed patients (52.8%) ($p=0.004$). Within the dialysis group, sarcopenia was more common in men (87.7%) than in women (55.6%) ($p<0.001$). Sarcopenic dialysis patients ($n=72$) had lower ASMI

(5.83±0.8 vs. 6.68±1.1 kg/m², p=0.003), lean mass (15.9±1.6 vs. 17.2±2.3 kg/m², p=0.027), fat mass (6.9±2.7 vs. 10±3.8 kg/m², p=0.002), and BMI (22.8±3.6 vs. 26.9±5.2 kg/m², p=0.001) compared to non-sarcopenic dialysis patients. Sarcopenic dialysis patients also had lower protein intake normalized to caloric intake compared to non-sarcopenic patients (34.4±6.4 vs. 38.8±7.2 g/1000 kcal, p=0.016), while carbohydrate and fat intake did not differ significantly.

**Conclusions:** Sarcopenia is more prevalent and severe in dialysis patients compared to those treated conservatively, particularly among men. This condition is associated with insufficient protein intake relative to caloric intake. Early screening for sarcopenia, alongside targeted nutritional support, is essential to reduce muscle loss and improve outcomes in dialysis patients.

**P031**

**Nanoscale Properties of Bone Tissue Near Lacunae In Women With Long-Term Type 1 Diabetes**

*Wen Qian[1], Maxwyll McConnell[1], Luke Schwaninger[1], Joseph Turner[1], Eleftherios Paschalis[2], Laura Graeff-Armas[3], Sue Bare[4], Joan Lappe[4], Robert Recker[4], Mohammed Akhter[4]*
*[1]University of Nebraska, Mechanical & Materials Engineering, Lincoln, United States*
*[2]Ludwig Boltzmann Institute for Osteology, Materials Science / Organic Matrix, Vienna, Austria*
*[3]University of Nebraska Medical Center, Division of Diabetes- Endocrine & Metabolism, Omaha, United States*
*[4]Creighton University, Osteoporosis Research Center, Omaha, United States*

**Abstract Text**

Bone mineral density (BMD) partially explains the increased fragility risk in patients with Type-1 diabetes. The objective here is to investigate the local intrinsic properties of bone tissue near peri lacunar space in biopsies from postmenopausal women with long term Type 1 diabetes (T1D) (Cases, n=8) and aged-matched non-diabetic controls (Controls, n=4). We hypothesize that bone fragility in T1D is due to alteration in intrinsic material properties in the peri-lacunar regions of bone tissue. Using AFM-nanoIR2 spectra, bone tissue's compositional analysis was performed in iliac crest biopsies from T1D and controls. NanoIR spectra were collected on several lacunae within each biopsy tissue. The bone compositional data was measured at "Lacunae-near" and 'Lacunae-far" i.e., 5 µm and 15 µm away from edge of each lacuna, respectively (**Figure-1**). Spectra were acquired over a range of 1900 to 922 $cm^{-1}$, and automatically averaged for better signal to noise ratio. Peak amplitude normalization was applied to all spectra. Both paired and un-paired t tests were used for near/far lacunae, and for the Cases and controls respectively, to determine differences in bone tissue's compositional variables (Mineral Matrix Area Ratio [MMA], Mineral Matrix Peak Ratio [MMP], Mineral Maturity Area Ratio [MMCA], Mineral Maturity Peak Ratio [MMCP]). While there were no differences in compositional variables in Cases at any region (near or far), the MMCA was higher in near than far regions of lacunae in both controls and combined average values of Cases and controls. Furthermore, both MMA and MMP were higher in controls than Cases. These data present bone tissue's compositions that varies between Control and Cases. Therefore, suggesting that the variation in the intrinsic properties (MMA, MMP) may be responsible for the increased bone fragility in T1Ds.

Additional work/data are needed to understand the deterioration of bone tissue in Type-1 diabetes related skeletal fragility.

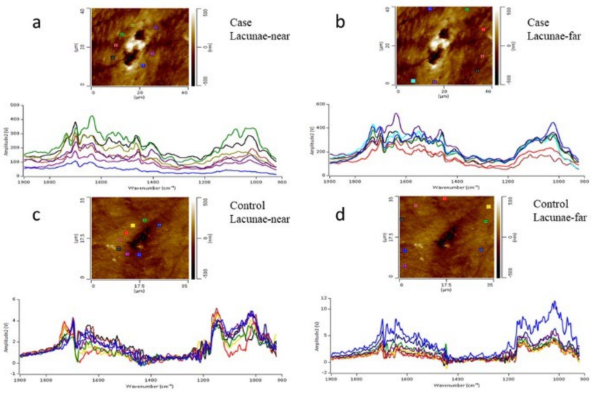

Figure 1: Comparison of localized IR spectrum between "lacunae-near" and "lacunae-far" for Case (a-b) and Control (c-d) samples respectively.

**P032**

**Bone material differences at the cement lines between postmenopausal patients that sustain fragility fractures and age- and BMD-matched controls.**

*Shibarjun Mandal*[1], *Sue Bare*[2], *Robert Recker*[2], *Mohammed Akhter*[2], *Eleftherios Paschalis*[3]
[1]*Ludwig Boltzmann Institute for Osteology, Material Science / Organic Matrix, Vienna, Austria*
[2]*Creighton University, Osteoporosis Research Center, Omaha, United States*
[3]*Ludwig Boltzmann Institute for Osteology, Materials Science / Organic Matrix, Vienna, Austria*

**Abstract Text**

Bone mineral density (BMD) is valuable in establishing fracture risk, yet it does not account for all factors resulting in fragility fracture (Fx). Fx is mostly dependent on bone toughness, which is determined by material properties around interfaces. Cement lines (CL) are a major interface within bone.

We analyzed bone material properties by Raman microspectroscopy in iliac crest biopsies from n=15 postmenopausal patients suffering Fx, and n=15, age- and BMD-matched controls (NoFx). The outcomes determined were mineral / matrix (MM), nanoporosity, and glycosaminoglycan (GAG) and pyridinoline (Pyd) content. Fig. 1a & b are overview pictures of a representative biopsy. Analysis focused in CL related to osteon activity (either inactive: no fluorescent labels evident, or active: fluorescent labels present (Fig. 1c)). Spectra were obtained right on the CL, as well as 2 µm towards the Haversian canal, and 2 µm outside, in interstitial bone (Fig. 1d). For each biopsy 3 active and 3 inactive osteons were considered, the 9 obtained values / osteon type averaged and treated as a single statistical unit. Data were compared by paired t-tests. Significance was assigned to $p<0.05$.

In both osteon types, Fx had higher MM values, while Pyd content was higher in the Fx group in inactive osteons (Fig 1e). To further investigate the source of the difference in MM, we considered the mineral and organic matrix (OM) contents separately. Mineral content was similar in the two groups, while the Fx group had lower OM content in both osteon types (Fig. 1f). This suggests that the mechanical properties in the area analyzed in the Fx group may be akin to the ones in hypermineralized bone.

The data of the present study provide a plausible mechanism behind the discrepancy in Fx occurrence between the two age- and BMD-matched groups of postmenopausal women.

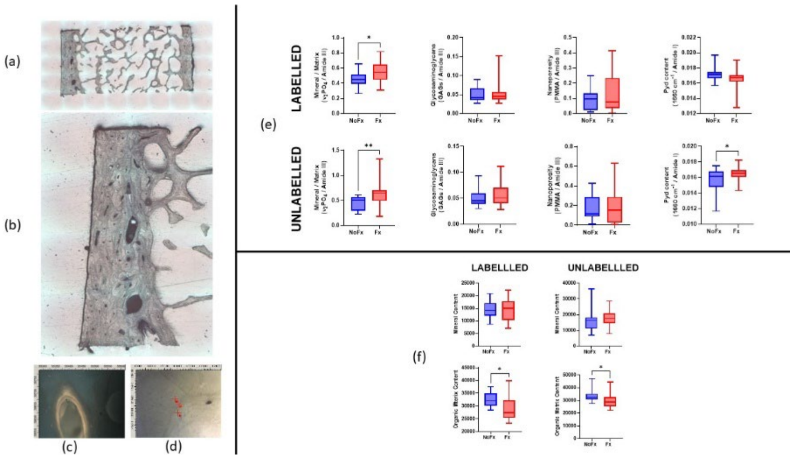

**P033**

**Bone structure parameters in type 1 and 2 diabetes mellitus**

*Ines Foessl*[1], *Kai Ammerer*[1], *Barbara Obermayer-Pietsch*[1], *Felix Aberer*[1]
[1]*Medical University of Graz,*
*Division of Endocrinology and Diabetology- Department of Internal Medicine, Graz, Austria*

**Abstract Text**

**Background:** Type 1 and type 2 diabetes mellitus (T1DM, T2DM) can lead to secondary osteoporosis as a late complication, with differences in fracture for the diabetes subtype. High-resolution peripheral quantitative computed tomography (HRpQCT) promises diagnostic advantages over dual X-ray absorptiometry (DXA).

**Purpose:** The aim of this study is to compare density and structural parameters from HRpQCT and DXA in individuals with T1DM or T2DM.

**Methods:** The prospective, monocentric observational study was approved by the local Ethics Committee and included 25 with T1DM and 17 with T2DM. DXA of the lumbar spine, hip and radius as well as HRpQCT of both distal tibia and radius were compared.

**Results:** No difference in bone mineral density (aBMD) measured by DXA of the radius, hip, lumbar spine, and TBS after adjustment for covariates (BMI, HbA1c, diabetes duration9. Late diabetic complications did not correlate with the DXA parameters. HRpQCT showed no difference between groups. People with late T2DM complications showed a 47% higher cortical porosity of the radius (p=0.022). In T1DM, a higher mean distance between the trabeculae of the tibia (0.72 vs. 0.85

p=0.041) was observed. In accordance with the results of the DXA, HRpQCT parameters of the tibia and radius did not correlate with BMI, diabetes duration or HbA1c in either group.

**Conclusion:** This study showed no differences between T1DM and T2DM in the bone parameters of neither DXA nor HRpQCT. In contrast, previous studies showed alterations of the cortical bone in T2DM and of the trabecular bone in T1DM. A good diabetes management, as in our patients, is likely important to maintain bone health. However, a longer duration of disease and currently poorer blood glucose control had no influence on bone parameters in this study, whereas late diabetic complications were associated with higher cortical porosity in T2DM and trabecular bone in T1DM.

**P034**

**The correlation study between paravertebral muscles and vertebral fracture grading**

*Yandong Liu*[1], *Wenshuang Zhang*[1], *Yi Yuan*[1], *Jian Geng*[1], *Ling Wang*[1], *Xiaoguang Cheng*[1]
[1]*Beijing Jishuitan Hospital, Radiology, Beijing, China*

**Abstract Text**

**Background:** Degeneration of the paravertebral muscles may contribute to osteoporotic vertebral fractures (OVF), but its relationship with OVF severity remains unclear.

**Purpose:** To explore the association between paravertebral muscle parameters and OVF risk.

**Methods:** A total of 3475 Chinese community participants underwent abdominal CT scans, with full-spine CT scout views obtained. OVF in T4-L4 vertebrae were identified and graded using Genant's semi-quantitative (SQ) method, categorizing participants into SQ0, SQ1, and SQ2-3 groups. Muscle parameters at the L3 vertebral level, including paraspinal extensor muscle cross-sectional area (PEMS), density (PEMD), fat area (FS), fat infiltration (FI), and psoas major muscle area (PMMS) and density (PMMD), were measured using Osirix software. Differences in muscle parameters among groups were analyzed, and single- and multi-factor logistic regression models assessed correlations with OVF risk.

**Results:** Among 2992 participants (1109 males, 1883 females), males showed no significant differences in muscle parameters between SQ0 and SQ1 groups, but significant differences in PEMS, PEMD, PMMD, FS, and FI between SQ0 and SQ2-3 groups. In females, all parameters except PMMS differed between SQ0 and SQ1 groups, and all parameters differed between SQ0 and SQ2-3 groups. In males, PEMD (OR: 0.68, 95% CI: 0.47–0.99, p=0.045) was protective against SQ2-3 fractures, while FS (OR: 1.41, 95% CI: 1.1–1.81, p=0.007) and FI (OR: 1.37, 95% CI: 1.07–1.76, p=0.013) were independent risk factors. PMMS (OR: 1.20, 95% CI: 1.01–1.43, p=0.039) and FS (OR: 1.17, 95% CI: 1.01–1.36, p=0.038) were risk factors for SQ1-3 vertebral deformity/fracture in males. In females, PEMD (OR: 0.78, 95% CI: 0.61–0.98, p=0.034) was protective against SQ2-3 fractures.

**Conclusion:** Higher SQ grades correlate with smaller, less dense, and more fat-infiltrated paravertebral muscles. PEMD is protective against SQ2-3 fractures, while FS and FI are risk factors in males. PMMS and FS are additional risk factors for SQ1-3 vertebral deformity/fracture in males.

**P035**

**Impaired composition of organic matrix in early onset osteoporosis**

*Agnes Ostertag*[1], *Bastien Leger*[2], *Eugenie Koumakis*[3], *Patrice Fardellone*[4], *Mylene Zarka*[5], *Thomas Funck-Brentano*[6], *Guillaume Mabilleau*[7], *Martine Cohen-Solal*[8]

[1]*Inserm U 1132 and universite Paris Cite, Hopital Lariboisiere, Paris, France*

[2]*Inserm U1132 Bioscar, Universite Paris Cite, Paris, France*

[3]*Hopital Cochin, Department of Rheumatology, Paris, France*

[4]*Hopital d'Amiens, Department of Rheumatology, Amiens, France*

[5]*Unité U1132 Bioscar and universite Paris Cite, Hopital Lariboisiere, Paris, France*

[6]*Inserm U1132 Bioscar and universite Paris Cite, Hopital Lariboisiere, Paris, France*

[7]*Inserm U1229- RMeS- REGOS, Universite d'Angers, Angers, France*

[8]*Inserm U1132 Bioscar- université Paris Cite, Department of Rare Bone diseases, Paris, France*

**Abstract Text**

**Background/Introduction:** Early onset osteoporosis (EOOP) is a rare form of primary osteoporosis defined by major skeletal fractures or low bone mineral density (BMD) that occur in early age. However, the characteristics of the extracellular matrix that may contribute to bone fragility is unknown.

**Purpose:** We aimed to explore the microarchitecture and bone composition in transiliac bone biopsies (BB) obtained from adult EOOP patients.

**Methods:** We analyzed the EOOP BB collected from 2009 to 2019 that were compared to historical control biopsies. Microarchitecture was measured by µ-CT and bone composition by Raman microspectroscopy and FTIR. The contribution of each component was assessed by Principal Component Analysis.

**Results:** We analyzed 18 BB of EOOP (age 34±8 years, LS BMD Z-score: -2.05±1.04 SD) and compared with 19 BB of age-matched healthy individuals. EOOP and control groups had similar bone volume (BV/TV: 23.3 ± 7.8 vs 24.0 ± 4.9 %, p=0.741). EOOP showed a lower Tb. Sp (540.0±172.5 vs 564.9±54.0 µm, p=0.026) and a higher Tb. connectivity density (13.7±6.5 vs 6.6±1.7 $mm^{-3}$, p<0.001) whilst cortical thickness was lower in EOOP than controls (Ct. Th 625.0±125.8 vs 900.3±429.6 µm, p<0.01).

Trabecular bone revealed higher GAG/Amide3 (0.70±0.08 vs 0.48±0.04, p<0.0001), Hyp/Pro ratio (0.84±0.05 vs 0.71±0.02, p<0.0001), and amount of advanced glycation end products carboxymethyllysine and Pentosidine in EOOP than in controls. EOOP also revealed a lower tissue mineralization compared to controls: $v_1PO_4/CH_2$ (4.7±0.8 vs 7.6±0.8, p<0.0001) and mineral maturity/crystallinity (0.0566±0.0007 vs 0.0571±0.0005, p<0.005). The crystal size index was significantly lower in EOOP than in controls (1.04±0.04 vs 1.16±0.08, p<0.0001). PCA revealed higher contribution of matrix phase rather than microarchitecture at both trabecular and cortical bone.

**Conclusion:** Matrix composition of EOOP is characterized by higher damage of the organic phase and reduced mineralization, that may explain the high risk of fractures.

**P036**

**Comparative microCT analysis of trabecular bone in iliac crest and radius from healthy individuals**

*Elisa Zamengo*[1]*, Stéphane Blouin*[2]*, Andrea Berzlanovich*[3]*, Barbara M. Misof*[2]*, Markus A. Hartmann*[2]
[1]*Ludwig Boltzmann Society,*
*Ludwig Boltzmann Institute of Osteology at Hanusch Hospital of OEGK and AUVA Trauma Centre Meid ling, Vienna, Austria*
[2]*Ludwig Boltzmann Society,*
*Ludwig Boltzmann Institute of Osteology at Hanusch Hospital of OEGK and AUVA Trauma Centre Meid ling, Vienna, Austria*
[3]*Medical University of Vienna, Center for Forensic Medicine, Vienna, Austria*

**Abstract Text**

**Introduction:** Understanding bone microarchitecture is essential for fracture risk assessment and designing patient-specific bone implants. Although transiliac bone biopsy is used in clinical evaluations, its representativeness for bone microarchitecture at other skeletal sites remains unclear.

**Purpose:** This study evaluates trabecular bone microarchitecture differences at the iliac crest and the radial head and age-related variations thereof.

**Methods:** From 12 bone-healthy individuals (two age groups: Group 1: n=6, 60.1±3.9 years, Group 2: n=6, 94.0±0.6 years), bone autopsy samples from iliac crest (n=6/6) and radius (n=4/5) were obtained (ethical approval: EK Nr. 1757/2013). Trabecular bone parameters were computed for the iliac crest biopsy site (cylindric volume with 7 mm diameter) and for the radial head (cubic volume with 5.7 mm edge length) starting from microCT scans (SCANCO 50, resolution: 24 µm, 90kV).

**Results:** Trabecular parameters differed significantly between the two anatomical sites. Compared to the biopsy site the radius had lower bone volume fraction (BV/TV, (5.11±1.31)% vs (20.94±7.49)%, p-value<0.0006) and trabecular thickness (Tb.Th, (123±18)µm vs (232±36)µm, p-value<0.0001) as well as higher trabecular separation (Tb.Sp, (956±101)µm vs (765±121)µm, p-value=0.006), while trabecular number (Tb.N) showed no significant differences ((1.01±0.10)$mm^{-1}$ vs. (0.94±0.09)$mm^{-1}$, p-value=0.122). Age-related differences showed similar trends at both sites, but were less pronounced at the radius: BV/TV and Tb.Th decreased of -25.21% and -7.09% at the radius as well as of -34.13% and -17.02% at the iliac crest in the older study group. Parameters showed no correlations between sites.

**Conclusions:** Our findings in healthy individuals suggest that trabecular bone microarchitecture is sparser and age-related bone loss is less pronounced at the radius compared to the iliac crest. The absence of correlations between outcomes of both sites suggests a limited ability of the transiliac biopsy to predict cancellous bone-microarchitecture at the radius, underlining the importance of gaining information of bone structure at fracture-relevant sites.

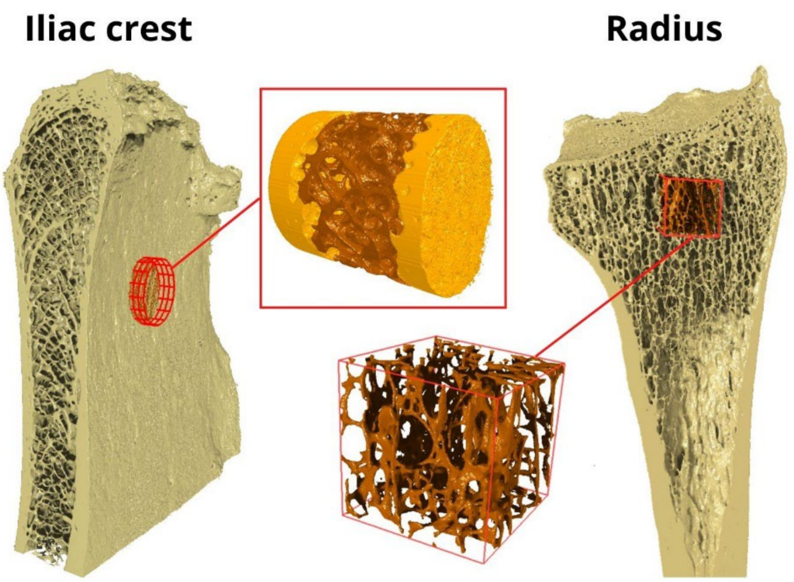

**Figure:** Iliac crest and radius segmentations of bone samples coming from the same donor with a zoom on the volume of interests used to compute trabecular bone parameters.

**P037**

**The Interaction Between Glycosaminoglycans and Pyridinoline Associates with Fragility Fracture Occurrence in Long-Term Type 1 Diabetic Patients**

*Eleftherios P. Paschalis[1], Laura A. Graeff-Armas[2], Sonja Gamsjaeger[1], Sue P. Bare[3], Robert R. Recker[3], Mohammed P. Akhter[3]*
*[1]Ludwig Boltzmann Institute of Osteology, Organic Matrix, Vienna, Austria*
*[2]University of Nebraska Medical Center, Division of Diabetes- Endocrine and Metabolism, Omaha, United States*
*[3]Osteoporosis Research Center, Creighton University, Omaha, United States*

**Abstract Text**

Increased fracture risk in type 1 diabetes patients is not fully captured by bone mineral density outcomes. Although advanced glycation endproducts have been widely implicated in the increased fracture risk in T1D, question whether this is associative rather than causative. Glycosaminoglycans (GAGs) and pyridinoline (Pyd; enzymatic trivalent collagen cross-link) are crucial for collagen fiber organization.

To test the hypothesis that the relationship between GAGs and Pyd is altered in long-term (>10 years) T1D patients sustaining fragility fractures, we analyzed iliac crest biopsies by Raman spectroscopy from two different cohorts (C): C1 5 fracturing T1D Fx, 6 nonfracturing T1D NoFx, and 6 healthy subjects (HC). C2 postmenopausal women; 5 healthy subjects (HC), and 8 age- and BMD-matched postmenopausal, non-fracturing T1D NoFx. In both C1 and C2 the only clinical difference was HbA1c which was elevated in T1D, though no differences were evident between T1D No Fx and T1D Fx groups. For each group, the individual patient GAG and Pyd contents were plotted against each other and compared by non-linear regression followed by F-test to determine whether one curve fits all data.

Comparisons in C1 indicated that the data cannot be described by one curve. Although HC and T1D NoFx data were similar (p = 0.15), T1D Fx data were significantly different compared to either HC or T1D NoFx (p = 0.003 and <0.0001, respectively). In C2, there were no significant differences between HC and T1D NoFx (p = 0.14). When C1 & C2 were considered together, the T1D Fx patients' data were significantly different from either HC or T1D NoFx patients, while no differences were evident between the latter two.

GAGs and Pyd play crucial roles in collagen fibrillogenesis and fibrillar organization. Thus, the plausible suggestion that emerges is that fiber organization is altered in T1D Fx patients.

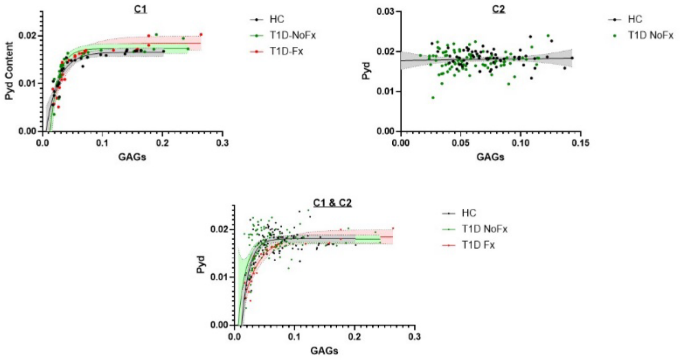

**P038**

**Chemical Evidence of a Bond between the Organic Matrix and Mineral in Human Bone: Insights from X-ray Photoelectron Spectroscopy**

*Dahlia S Eldosoky[1], Ulrich Hansen[2], Richard Abel[1]*
*[1]Imperial College London, Surgery and Cancer, London, United Kingdom*
*[2]Imperial College London, Mechanical Engineering, London, United Kingdom*

**Abstract Text**

Bone is a composite material made up of a collagen matrix reinforced by carbonated hydroxyapatite. The mechanical properties of a composite is heavily dependent on the interface between the matrix and the reinforcing element. However, the nature of the interface is not well understood. In fact, there is no evidence of a chemical bond between the matrix and the mineral. Our question was: what holds the mineral and matrix together? Nitrogen (N) is prevalent in the collagen while calcium ($CA^{2+}$) is abundant in the mineral and these elements have an affinity to chemically bond. Hence, we aimed to determine if N=Ca bonds exist as their presence suggests a tie between the matrix and the mineral. A secondary aim was to investigate if the amount of N=Ca bonds is reduced in bone with low bone mineral density (BMD).

X-ray photoelectron spectroscopy (XPS) was performed on 5 human cadaveric femora (BMD: T-score from -1.0 to -3.0).

The Figure shows the XPS nitrogen spectrum (left) and calcium spectrum (right) of three bone specimens (top, middle and bottom graphs, respectively). In both the nitrogen and the calcium spectrum the results demonstrates a peak (the pink curves) corresponding to the existence of N=Ca bonds. Noteworthy, the intensity of the N=Ca peak drops with lower BMD (note T-scores). Additional testing (not shown) suggests a 4-fold decrease in intensity.

The presence of N=Ca bonds in bone seemed clear, however, the matrix includes nitrogen containing non-collagenous proteins making the source of the nitrogen unclear. The findings also suggests that loss of bone mineral (low BMD) is linked to loss of interfacial bonding.

This study provides the first evidence of a physical bond between the organic component and mineral in bone and indicates that these bonds reduce with BMD suggesting a possible causal reason for poor quality osteoporotic bone.

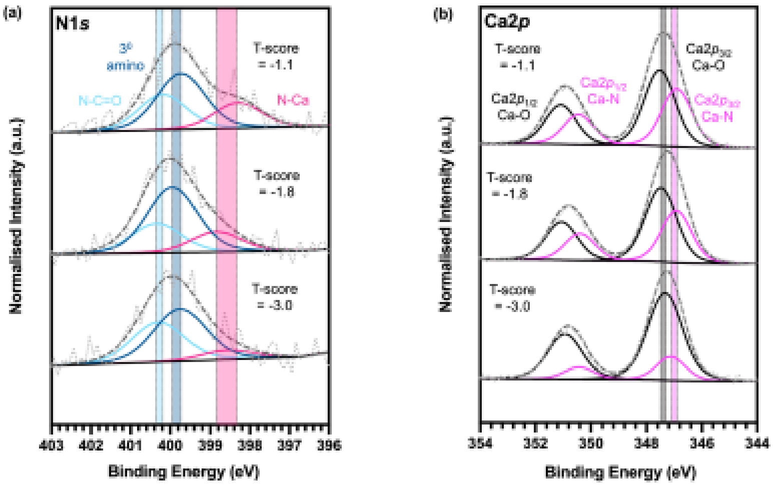

**P039**

**Value of repeat DXA scans: a multisite audit across Three NHS Hospitals in the United Kingdom**

*David Schramm[1], Rafaela Goncalves[2], Ruth Kinyua[3], Jo Rotherham[4], Laura Taylor[5], Simone Edwards[6], Gordon MacDonald[6], Malgosia Magliano[7], Muhammad Kassim Javaid[8]*
*[1]Medical Student, Medical Sciences Division, Oxford, United Kingdom*
*[2]Oxford University Hospitals NHS Trust, Department of Radiology, Oxford, United Kingdom*
*[3]University of Oxford, Department of Radiology, Oxford, United Kingdom*
*[4]Buckinghamshire Healthcare NHS Trust, Department of Rheumatology, Amersham, United Kingdom*
*[5]Department of Rheumatology, Royal Berkshire NHS Trust, Reading, United Kingdom*
*[6]Royal Berkshire NHS Trust, Department of Rheumatology, Reading, United Kingdom*
*[7]Buckinghamshire Healthcare NHS Trust, Department of Rheumatology, Stoke Mandeville, United Kingdom*
*[8]University of Oxford,*

*Nuffield Department of Orthopaedics- Rheumatology and Musculoskeletal Sciences, Oxford, United Kingdom*

**Abstract Text**

**Introduction:** Patients at high fracture risk require access to timely dual-energy x-ray absorptiometry (DXA) scans to access anabolic therapies. Limitations in DXA capacity lead to delays in diagnosis and treatment initiation. DXA is also used to monitor bone density per national and local guidelines.

**Purpose:** To evaluate the appropriateness of repeat DXA scans to increase local DXA capacity.

**Methods**: We evaluated consecutive DXA scans performed in January 2024 at three UK hospitals. Patient demographics, referral details, fracture history and the use of endocrine therapies for breast or prostate cancer were extracted from medical records. We compared the interval between repeat scans to Oxford and ESMO guidelines. Oxford guidelines recommend scan intervals of 5–10 years for bisphosphonates, 4–7 years for zoledronate, and scan only after fractures on denosumab. Non-parametric tests were used to compare differences in intervals using R (version 4.4.2).

**Results**: Of 406 consecutive DXA, 40.6% were repeats, varying 26% to 44% between sites. Among repeats, 4/165 (2.4%) had a new fracture between scans and 39/165 (23.6%) were on cancer-related endocrine therapy. The median interval between scans was 3.88 (IQR 2.35, 5.28) years, with a shorter interval for those on endocrine therapy ($p < 0.001$). Scan intervals varied by anti-osteoporosis treatment (Figure). The mean patient age for repeat scans was 65.1 years (SD: 13.5), and the mean lowest DXA score was -1.82 (95% CI: -1.99, -1.64). Annual BMD changes ranged from 0% (IQR -2.94, 3.4), with the largest decreases in untreated patients and largest increases in those on denosumab. Compared to the guidance, avoidable repeat scans occurred in 49.2% of repeat scans without and 27.5% with endocrine therapy.

**Conclusion**: Adherence to guideline-recommended intervals for repeat DXA scans was suboptimal, with 44.0% of repeat scans deemed avoidable. Standardising the triage of DXA referrals with regular benchmarking could increase DXA capacity for patients at high fracture risk.

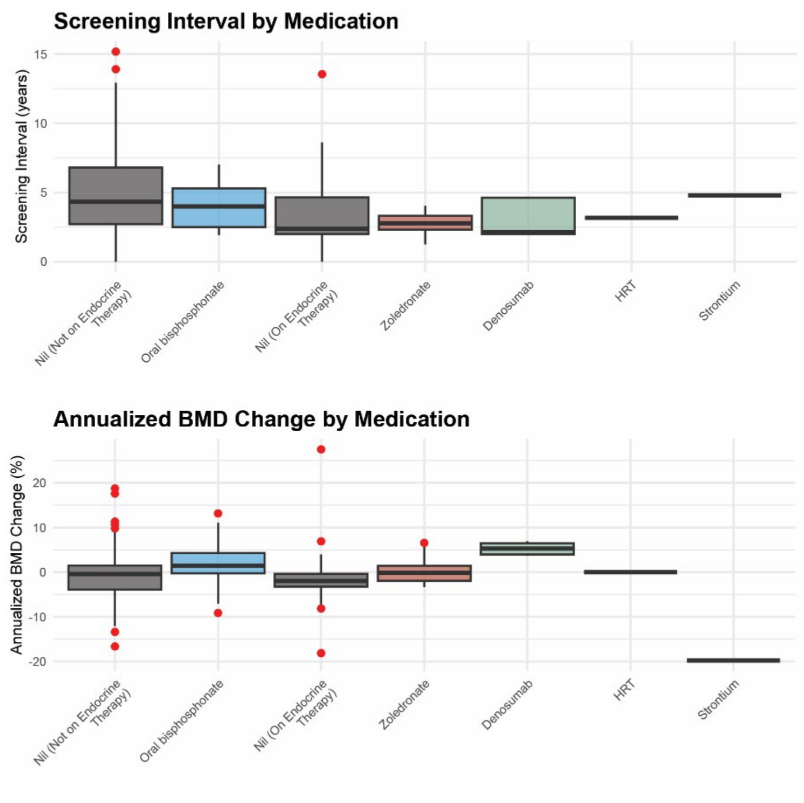

**P040**

**Bone Dynamics: The Cellular Response to Unloading**

*Alamelu Sundaresan*[1], *Vivek Mann*[2]
[1]*Texas Southern University, Biology, 3100 Cleburne Street- Houston- TX 77004, United States*
[2]*Texas Southern University, Biology, 3100 Cleburne Street- Houston TX 77004, United States*

**Abstract Text**

**Introduction/Background**: The human skeletal system comprises bones, cartilage, ligaments, and tendons, with bone serving as a living tissue organized within a mineralized extracellular matrix. It plays mechanical, synthetic, and metabolic roles. Despite its importance, understanding the cellular interactions in bone formation and response to stimuli is challenging due to the absence of suitable in vitro models. Bone grafts are widely used for fracture healing and bone regeneration, with over two million procedures annually, fueling a global market projected to reach USD 3.9 billion by 2025.

**Purpose:** Effective bone grafts or scaffolds require biomaterials mimicking bone's extracellular matrix, osteoprogenitor cells, and growth signals. However, replicating bone's structural and functional properties remains challenging. Bone tissue engineering integrates cells, biocompatible scaffolds, and growth factors but struggles to emulate natural bone remodeling. Microgravity research using devices like NASA's HARV, and RPM offers insights into 3D cell cultures, aiding tissue engineering advancements.

**Methods:** The experiment will use hFOB 1.19 osteoblast cell lines cultured in osteogenic medium to induce osteogenesis. Cells ($10^6$/ml) will be rotated in RCCS HARV at 1-2 rpm initially, increasing to 8-12 rpm. Cultures will last up to 21 days, with medium changes every three days. Constructs will undergo histology, RNA isolation, or mechanical testing.

**Results:** Under unit gravity, hFOB cells grew in adherent, confluent monolayers after 1 to 21 days. In the HARV, cells formed 3D aggregates, increasing in size (3–5 mm by day 21) and number. Histological analysis revealed uniformly distributed cells with external cell layers becoming prominent over time. Hematoxylin–Eosin staining showed abundant osteoblasts and extracellular matrix (ECM) production, while elastin staining identified elastin secretion critical for load-bearing tissues. Sirius Red staining highlighted high collagen expression, the primary organic component of bone ECM.

**Conclusion:** HARV-grown hFOB constructs exhibit tissue-like organization with significant elastin and collagen deposition, emphasizing ECM's structural and biochemical roles.

**P042**

**Dissecting the role of the Dipeptidyl peptidase 3 (DPP3) as a key player in bone biology: in health and disease**

*Emilia Bevacqua[1], Lucca Giacchi[2], Fabio Bedin[3], Daniele Panetta[4], Dario Strina[5], Emanuela Morenghi[6], Gherardo Mazziotti[7], Cristina Sobacchi[8]*
*[1]Humanitas Research Hospital,*
*Bone Metabolism Unit- Laboratory of Traslational Endocrinology and Metabolism- IRCCS Humanitas R esearch Hospital, Rozzano, Italy*
*[2]University of L'Aquila, Department of Biotechnological and Applied Clinical Sciences, L'Aquila, Italy*
*[3]European Institute of Oncology IRCCS, Department of Experimental Oncology at IEO, Milan, Italy*
*[4]National Research Council, CNR Institute of Clinical Physiology, Pisa, Italy*
*[5]Consiglio Nazionale delle Ricerche, Istituto di Ricerca Genetica e Biomedica, Rozzano, Italy*
*[6]IRCCS Humanitas Research Hospital, Biostatistic Unit, Rozzano, Italy*
*[7]IRCCS Humanitas Research Hospital,*
*Endocrinology- Diabetology and Medical Andrology Unit- Metabolic Bone Diseases and Osteoporosis S ection, Rozzano, Italy*
*[8]IRCCS Humanitas Research Hospital,*
*Bone Metabolism Unit- Laboratory of Traslational Endocrinology and Metabolism, Rozzano, Italy*

**Abstract Text**

**Background:** Bone quality encompasses aspects of bone composition and structure that contribute to bone strength independently of BMD[1]. We previously demonstrated bone loss in Dpp3 knock-out ($Dpp3^{-/-}$) owing to enhanced resorption[2], but we didn't investigate other bone quality-related factors.

**Purpose:** To define the role of DPP3 in bone composition and quality in pathophysiological conditions.

**Methods:** Animal experimentation approval: n.165/2023-PR. Proteomic analysis of flushed bone of 6-month-old untreated male WT and Dpp3$^{-/-}$ mice through untargeted high-resolution mass-spectrometry (n=4), as described[3]. Protein extracts prepared as described[4].
Treatment: Western Diet (45% fat) or chow (control, CT) for 12 weeks after weaning of male and female WT and Dpp3$^{-/-}$ mice (<5n<10). GTT and ITT after 0,6 and 12 weeks of treatment following standard protocols. Serological analyses at necropsy, e.g. AGE, Leptin, RANKL, OPG, P1NP (ELISA assays); Dpp3 enzymatic activity[2]. Cyclic-nanoindentation on femurs for bone biomechanical testing according to standard protocol (Force=4N;10 indentation cycles).

**Results:** Proteomic analysis detected 920 proteins in all samples, 90 of them were significantly up-regulated in Dpp3$^{-/-}$ vs WT mice (**A**), including proteins involved in matrix remodelling, lipid metabolism and oxidative stress (**B**). HFD treatment reduced glucose tolerance in WT and Dpp3$^{-/-}$ mice (*p*<0.001;**C**) and lowered insulin sensibility (in males only). In the serum, AGEs significantly increased in HFD vs CT Dpp3$^{-/-}$ females (*p*<0.01;**D**); leptin increased in HFD WT and Dpp3$^{-/-}$ males and in HFD females (*p*<0.001;**E**); Dpp3 activity decreased only in HFD-fed females (*p*<0.05;**F**). Parameters assessed by nanoindentation were mainly affected by the diet (**G**); RANKL was significantly increased in male HFD Dpp3$^{-/-}$ vs CT Dpp3$^{-/-}$ and HFD WT mice (p>0.001); OPG and P1NP did not change (**H-J**).

**Conclusion:** DPP3 loss affected bone composition; candidate proteins will be validated by Western blot analysis. In our experimental setting, HFD elicited sex-dependent phenotypes, only partially dependent on DPP3. Further evaluations are ongoing.

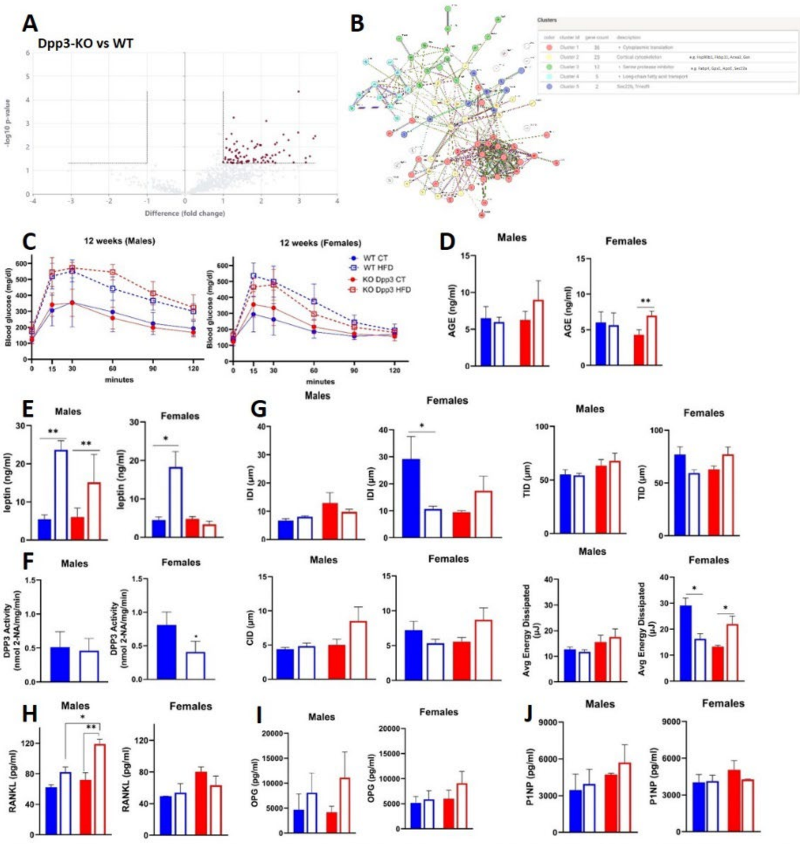

**Figure 1**. **A** Volcano plot of proteomic analysis indicated proteins up-regulated in KO-Dpp3 mice vs WT. **B** Enrichment proteomic analysis by STRING. **C** GTT after 12 weeks of treatment. Two-way ANOVA statistical analysis for AUC (Males CT WT vs HFD WT *p*<0.001), (Males CT KO Dpp3 vs HFD KO Dpp3 *p*<0.001), and after 120 min (Males CT WT vs HFD WT *p*=0.0146), (Males CT KO Dpp3 vs HFD KO Dpp3 *p* =0.0006). **D-E** AGE and Leptin serum levels respectively, One way ANOVA (5<n<8). **F** Dpp3 enzymatic activity, Mann Withney (3<n<6). **G** c-nanoindentation and related parameters: IDI, TID, CID and Average energy dissipated, Mann Withney, 5<n<10. **H-J** Rankl, OPG, and P1NP serum levels, Mann Withney (3<n<6).

**P043**

**Parathyroidectomy in Male Sprague-Dawley Rats Impairs Bone Microstructure and Material Properties**

*Coralie Noel*[1], *Guillaume Mabilleau*[1]
[1]*University of Angers, Inserm UMR_S 1229 RMeS - REGOS Team, Angers, France*

**Abstract Text**

**Background/Introduction**: Hypoparathyroidism (HPT) is a rare bone disorder characterized by low or absent plasma PTH levels and hypocalcemia. About 75% of HPT cases follow neck surgery and are associated with neuromuscular disorders, calcinosis, kidney stones, chronic kidney disease, and cardiovascular issues. Skeletal manifestations include increased risk of spine and radius fractures, despite higher bone mass.

**Purpose**: We hypothesized that bone fragility in HPT arises from altered bone material properties.

**Methods**: All procedures were carried out in accordance with the European Union Directive 2010/63/EU for animal experiments and were approved by the regional ethical committee for animal use (authorization 32915-2021090114386646v4). A rat model of HPT was developed using male Sprague-Dawley rats. Parathyroidectomy (PTX) was performed on 8 rats (PTX group) at 10 weeks by a trained surgeon. Eight sham-operated rats matched for age and sex served as controls. All animals received calcein injections (10 mg/kg) 10 and 2 days before sacrifice. Bone microarchitecture, biomechanics, and material properties were analyzed. Mann-Whitney tests were used, with p-values <0.1 considered significant.

**Results**: PTX resulted in lower plasma PTH, hypocalcemia, and hyperphosphatemia, consistent with HPT. Three-point bending tests showed reduced ultimate force (-13%, p=0.05) and yield load (-21%, p=0.081) in the appendicular skeleton, and reduced ultimate force (-21%, p=0.035) and yield force (-25%, p=0.051) in the axial skeleton. Appendicular cortical bone parameters decreased, including Tt.Ar (-8%, p=0.029), Ma.Ar (-12%, p=0.081), and Ct.Ar (-6%, p=0.020). Trabecular bone mineral density and microstructure remained unchanged. In the PTX group, carbonate/phosphate ratio (-35%, p=0.008) and collagen maturity (-15%, p=0.073) were reduced, while hydroxyproline content and nanoporosity (-62%, p=0.051) increased.

**Conclusion**: Parathyroidectomy and hypoparathyroidism impair cortical bone structure and material properties, contributing to bone fragility in rats. Further studies are needed to assess whether similar effects occur in humans.

**P044**

**Human cortical bone ECM proteome: the fingerprint of bone quality**

*Tatiana Kochetkova*[1,2], *Michael Stumpe*[3], *Jörn Dengjel*[3], *Jonathan Avaro*[4], *Bruno Silva*[4], *Antonia Neels*[4],

*Andreas Borgschulte[5], Markus S. Hanke[6], Johann Michler[2], Philippe Zysset[1], Jakob Schwiedrzik[2,7]*
*[1]University of Bern, ARTORG Center for Biomedical Engineering Research, Bern, Switzerland*
*[2]Empa - Swiss Federal Laboratories for Materials Science and Technology,*
*Laboratory for Mechanics of Materials & Nanostructures, Thun, Switzerland*
*[3]University of Fribourg, Metabolomics and Proteomics Platform- Department of Biology, Fribourg, Switzerland*
*[4]Empa - Swiss Federal Laboratories for Materials Science and Technology, Center for X-ray Analytics, St. Gallen, Switzerland*
*[5]Empa - Swiss Federal Laboratories for Materials Science and Technology,*
*Chemical Energy Carriers and Vehicle Systems Laboratory, Dübendorf, Switzerland*
*[6]University Hospital of Bern, Department of Orthopedic Surgery, Bern, Switzerland*
*[7]Empa - Swiss Federal Laboratories for Materials Science and Technology,*
*Laboratory for High Performance Ceramics, Dübendorf, Switzerland*

**Abstract Text**

**Introduction:** Emerging experimental evidence suggests that bone extracellular matrix (ECM)-level elastic and yield properties are remarkably constant in aging patients and remain unrelated to low-trauma fracture. It is, however, debatable whether the same is true for refined bone ECM composition that may influence crack initiation and propagation.

**Purpose:** Assessing cortical bone ECM composition in aging patients with and without the occurrence of femoral neck fracture to highlight potential compositional markers of bone quality.

**Methods:** Cortical bone samples of femoral neck inferomedial regions were collected during the total hip arthroplasty of 38 patients (45-89 y.o.) suffering from coxarthrosis (no fracture) or hip fracture, in accordance with the ethical approval 2018-01815 (Cantonal Ethics Commission of Bern, Switzerland). Liquid chromatography-mass spectrometry (LC-MS)-based proteomic analysis was complemented by Raman spectroscopy and FTIR to assess ECM chemical composition variations. On a subset of samples (N=18, 62-89 y.o.) SAXS/WAXS measurements were used to characterize the mineral fraction.

**Results:** From the complementary Raman spectroscopy and FTIR measurements, only mineral to matrix ratio demonstrated a marginal increase in fracture patients ($p=0.04$), no significant differences in the ECM mineral characteristics were observed for the fractured patients. Mineral platelet length and width were positively correlated with the mineral to matrix ratio ($p<0.004$). The overall ECM chemical composition and mineral morphology were constant with patient's age. The in-depth proteome analysis revealed overexpression of the collagen trimers, specifically COL16A1 in fracture patients. Moreover, principal component analysis of the ECM proteome revealed a subtle distinction between patients with and without the occurrence of femoral neck fracture (Fig. 1), suggesting potentially relevant differences at the proteomic level.

**Conclusion:** In contrast to spectroscopically-derived compositional characteristics, ECM proteome measured via tandem LC-MS highlights potential markers of bone fragility. Despite the low number of patients, the proposed multimodal characterization approach yields new perspectives for organo-mineral bone matrix assessment.

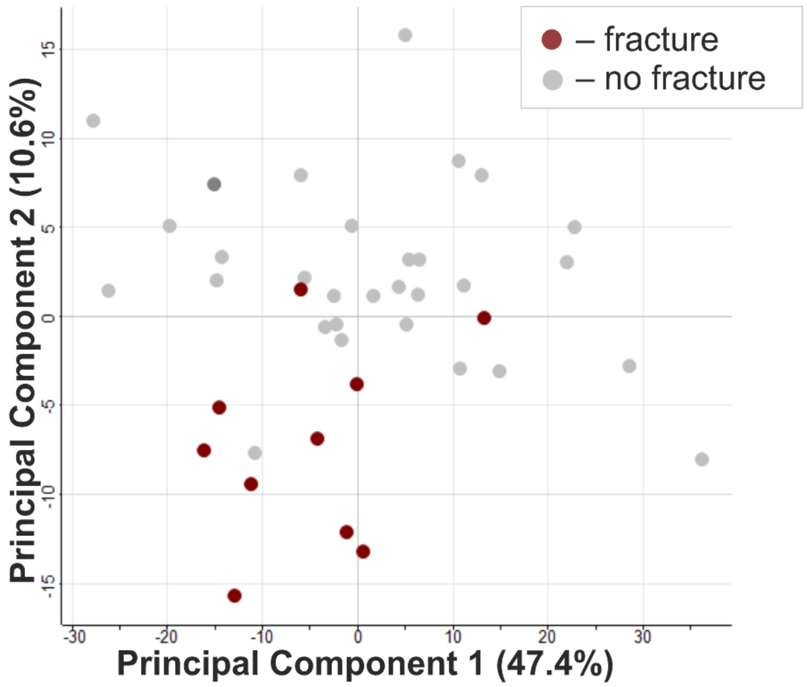

**P045**

**High-throughput modeling predicts electrostatic contributions to collagen contraction driven by osmotic pressure**

*James Rowe[1], Peter Fratzl[2], Daniele Dini[1], Richard Abel[3], Harrison Nicholas[4], Ulrich Hansen[1]*
*[1]Imperial College London, Mechanical Engineering, London, United Kingdom*
*[2]Max Planck Institute of Colloids and Interfaces, Biomaterials, Potsdam, Germany*
*[3]Imperial College London, Surgery and Cancer, London, United Kingdom*
*[4]Imperial College London, Chemistry, London, United Kingdom*

**Abstract Text**

Water plays a crucial role in collagen-based biomaterials, including bone. Recent experiments have shown that increases in osmotic pressure induce collagen fibril contraction, potentially toughening bone by pre-stressing the mineral phase. However, until now, the mechanism driving this phenomenon has not been understood. This work employs molecular dynamics simulations of collagen-mimetic peptides to characterise and understand the response of diverse collagen sequences. Our simulations reveal that charge interactions are critical in controlling the osmotic pressure response. As osmotic pressure increases, screening between charged residues diminishes, leading to localised contractions. Crucially, significant contraction is only observed with specific inter-residue distances, resulting in highly localised contractions within the full type-I collagen sequence. Specifically, we find that contraction is maximised when there is a 1-5 or 1-6 relationship between positively and negatively charged amino acid sidechains. This work provides a foundation for understanding the role of water and electrostatic interactions in the mechanical behaviour of collagen-based materials, with implications for bone health, tissue engineering, and the development of novel biomaterials. Future studies will investigate how post-translational modifications associated with ageing and disease may alter these charge groups and their impact on collagen and bone function.

| Type of sequence | Mean (SD) contraction from 2.5 to 0.1 water molecules per amino acid |
|---|---|
| No charged side chains (N=10) | -0.9% (0.7%) |
| 1-2, 1-3 or 1-4 relationship between oppositely charged side chains (N=8) | -0.1% (1.0%) |
| 1-5 or 1-6 relationship between oppositely charged side chains (N=5) | 6.2% (1.3%) |

Mean contraction of collagen-mimetic peptides of different sequences. It can be seen that sequences with a 1-5 or 1-6 relationship between the charged groups show significantly more contraction. A negative contraction indicates that the sequence expands upon drying.

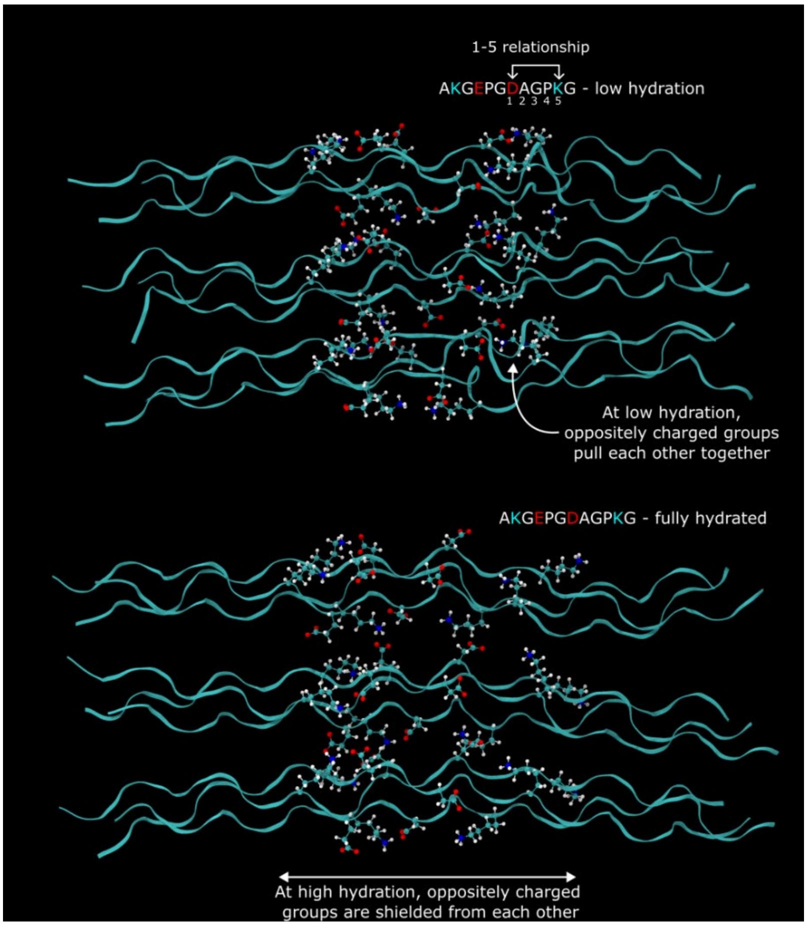

Images of a structure containing a 1-5 relationship between charged groups, showing how attraction between the charged groups leads to local contractions of the backbone.

**P046**

### Associations of ultrasound attenuation and backscatter coefficients in cortical bone with intracortical pore morphology

_Kay Raum_[1]

[1]*Charité-Universitätsmedizin Berlin, Center for Biomedicine, Berlin, Germany*

### Abstract Text

The attenuation and backscatter coefficients α(f) and BSC(f) measured in cortical bone have previously shown predictive value in distinguishing fragility fractures. The aim of this study is to investigate the sensitivity of α(f) and BSC(f) to alterations of the intracortical pore diameter distribution Ct.Po.DmD and tissue absorption $\alpha_{abs}$ in a well-controlled in-silico study.

A parametric numerical simulation study (N=272) utilized a 2D finite-difference time-domain (FDTD) code to model cortical bone plates with randomly distributed circular pores. To mimic a heterogeneous pore size distribution, an erosion procedure was applied, gradually increasing pore sizes from the outer to the inner boundaries. While material properties remained constant (c11=c22=23.7 GPa, c12=9.5 GPa, c66=6.6 GPa, ρ=1.93 kg/m$^3$), thickness, $\alpha_{abs}$, and Ct.PoDm.D were varied. The model simulated in-vivo conditions, positioning the plate 20 mm below a linear transmitter array with 16 elements (element size = 0.3 mm) emitting a 6-MHz center frequency, 80% bandwidth pulse focused at a depth of 21 mm. α(f) and BSC(f) derived from in-silico data were compared concerning changes in Ct.PoDmD and tissue absorption and associations with pore morphology and absorption were derived by means of PLS regression analysis.

The mean BSC was associated with pore density and tissue absorption (adj. $R^2$ = 0.49, RMSE = 0.83 dB). Shape and amplitude of BSC(f) were determined by the gradient of the pore size distribution and pore density, respectively. Scattering at pores resulted in a positive slope of α(f), which was not significantly impacted by pore size distribution and pore density (Fig. 1). Increasing tissue absorption resulted in an increase of measured attenuation. The mean attenuation was determined by pore density and tissue absorption (adj. $R^2$ = 0.90, RMSE = 0.22 dB/mm).

Cortical backscatter and attenuation coefficients exhibit distinct sensitivities associated with unbalanced remodeling, reduced turnover, and viscoelastic matrix properties.

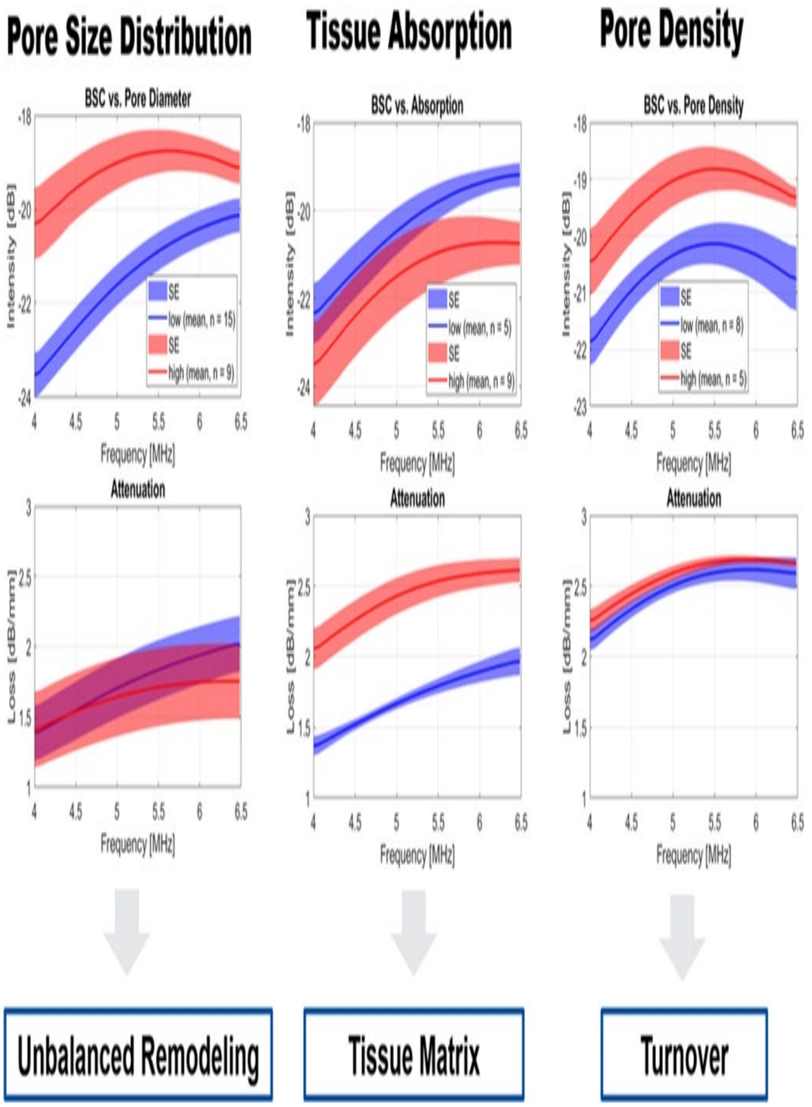

**P047**

**Multiscale investigation of bone quality at the interface formed by biodegradable magnesium implants using 2D qBEI imaging and 3D micro-CT**

*Sven Schimek*[1], *Amel Manel Batouche*[2], *Imke Fiedler*[2], *Katharina Jähn-Rickert*[2], *Björn Busse*[2], *D.C. Florian Wieland*[1]
[1]*Helmholtz-Zentrum hereon, Institute of metallic biomaterials, Geesthacht, Germany*
[2]*University Medical Center Hamburg-Eppendorf, Department of Osteology and Biomechanics, Hamburg, Germany*

**Abstract Text**

**Introduction:** The majority of implants used for fracture treatment are made from non-degradable materials like titanium alloys, which remain in the body unless surgically removed. This presents significant challenges, especially in children, as these implants cannot adapt to growing bones, often necessitating secondary surgeries with associated risks and extended recovery periods. Additionally, the stiffness disparity between titanium alloys and bone can lead to stress shielding, which can trigger enhanced bone resorption and, thus, deteriorates the mechanical properties. Magnesium (Mg) alloys offer an innovative alternative due to their biodegradability and mechanical properties resembling those of cortical bone, making them promising for osteoimplants. However, understanding the mutual interaction of the degradation process of Mg implants with the tissue remodeling remains challenging.

**Purpose:** This study investigates the influence of Mg-based implants on bone mechanical properties and ossification processes. It aims to analyze the multiscale impact of Mg alloys on bone quality, focusing on early-stage healing using both 2D and 3D methods.

**Methods:** Tibiae of rats were implanted with Mg alloys and titanium alloys which served as control. The rats were sacrificed after 3, 7, 14, 28, and 90 days. 2D quantitative backscattered electron microscopy (qBEI) was employed to examine bone-implant interfaces, focusing on bone area fraction, mineral density distribution, and the density of osteocyte lacunae near the implant (Fig. 1). Synchrotron micro-CT measurements were utilized to provide detailed assessments of bone volume fraction (BV/TV) and the spatial organization of lacunae.

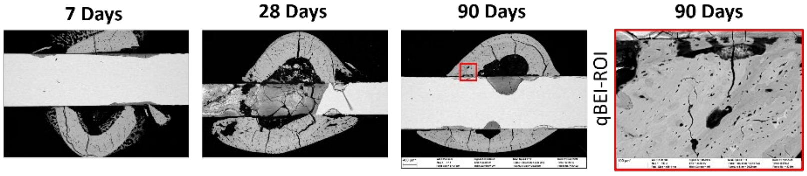

*Figure 1: Overview of the samples at the three time points (7, 28, and 90 days). The region of interest (ROI) in red highlights the iimplant-bone interface.*

**Results:** The results show that Mg implants stimulated bone turnover, resulting in lower mineralization and calcium heterogeneity, likely due to insufficient time for bone maturation. Both 2D and 3D analyses revealed decreased lacunar density and BV/TV for Mg implants compared to titanium.

**Conclusion:** The advanced multiscale approach demonstrates efficacy, revealing significant differences in bone quality parameters between titanium and magnesium implants during the early stage of healing.

**Poster Sessions: Bone development/growth and fracture repair**

**P050**

**Analysis for compression rate progression in osteoporotic vertebral compression fracture**

*Jaewan Soh[1], Jae Chul Lee[2], Hyun-Junl Kim[1]*
*[1]Hanyang University Guri Hospital- College of Medicine, Orthopaedic Surgery, Guri, Korea- South*
*[2]Soonchunhyang University Hospital- College of Medicine, Orthopaedic Surgery, Seoul, Korea- South*

**Abstract Text**

**Background:** Osteoporotic vertebral compression fractures (OVCF) treatment aims to reduce pain and prevent further compression, but compression often progresses during conservative treatment. However, studies on factors affecting compression rate progression and the timing when compression progresses the most are insufficient.

**Purpose:** To identify factors affecting compression rate progression in osteoporotic vertebral compression fractures (OVCF) and determine when this progression occurs most significantly.

**Methods:** Sixty-one patients with OVCF, followed for more than six months, were included. Factors such as age, gender, fracture level, bone mineral density (BMD), body mass index (BMI), osteoporosis medication, initial compression rate, fracture location, and pelvic incidence were assessed. Compression rates were measured initially and at 1, 2, 3, and 6 months. The correlation between these factors and compression rate progression of more than 30% was analyzed using univariate and multivariable Cox regression analysis. Compression rate progression over time was evaluated using a repeated measures general linear model, and clinical outcomes were assessed via paired t-test.

**Results:** Univariate analysis showed that an initial compression rate of less than 30% was significantly associated with progression($p=0.001$). Multivariable Cox regression confirmed this association($p=0.015$, Hazard ratio=3.103, 95% Confidence Interval 1.249-7.710). The most significant compression rate progression occurred within the first month of follow-up($p=0.0006$). At the final follow-up, both VAS for back pain($p=0.034$) and ODI($p=0.025$) were significantly higher in the compression rate progression group.

**Conclusions:** An initial compression rate of less than 30% was associated with a higher correlation of compression rate progression, particularly within the first month after injury. Compression rate progression was correlated with poorer clinical outcomes in terms of pain and quality of life.

**P051**

**Deciphering the impact of age on cortical bone remodelling**

*<u>Mai Ibrahim</u>[1], Martin Schicht[2], Flora Weber[1,3], Anika Grüneboom[1,3], Ulrike Steffen[1]*
*[1]Universitätsklinikum Erlangen and Friedrich-Alexander University Erlangen-Nürnberg FAU, Department of Internal Medicine 3 - Rheumatology and Immunology, Erlangen, Germany*
*[2]Friedrich-Alexander University Erlangen-Nürnberg, Department of Functional and Clinical Anatomy, Erlangen, Germany*
*[3]Leibniz-Institut für Analytische Wissenschaften-ISAS-e.V, Department of Biopsectroscopy, Dortmund, Germany*

**Abstract Text**

**Background:** Two models of bone remodelling have been previously reported in which trabecular bone is remodelled on the surface and cortical bone in the haversian canal. A recent study described directed modelling of cortical bone with bone formation at the endosteum and bone resorption at the periosteum (endo-t-p) in postnatal mice with growing bones (PMID: 39119717). However, it is unknown whether the trans-pairing of osteoclasts and osteoblasts is maintained during cortical bone remodelling with age.

**Purpose:** Investigate the effect of aging on the cortical bone remodelling.

Methods: In the present study, we investigated cortical bone remodelling in mice of different age (4,

8, 15 and 26 weeks) by serial intraperitoneal injection of calcein and alizarin red and by immunostaining and electron microscopy of osteoblasts, osteoclasts and osteocytes.

**Results:** Our results showed directed bone remodelling in long bones from the endosteum to the periosteum at all ages. Bone turnover speed was strongly side dependent and highest at the posterior side of the tibia. We observed a significant decrease in bone turnover rate with age with 10.77±2.3

µm/day in 4-week-old and 3.24±0.9 µm/day in 15-week-old mice ($p<0.0001$). Osteoclasts were strongly enriched at the periosteum (9.667±3.14) compared to the endosteum (0.1667±0.4; $p<0.0001$).

Conversely, bone forming osteoblasts were mainly observed at the endosteum. To check the distribution of the different osteocyte maturation stages, we stained for Podoplanin (early marker)

and dentin matrix protein 1 (DMP1-mid stage marker). Podoplanin positive osteocytes were located towards the endosteum (14.5% positivity compared to 2.86% positivity at the periosteum; $p<0.01$). On the other hand, DMP1 positive cells distributed more towards the periosteum (23.95% positivity)

compared to endosteum (11.57% positivity; p.0.0081).

**Conclusion:** Based on our findings, we conclude that directed endo-t-p cortical remodelling of long bones continues in the older age and that it is not limited to developmental stages.

**P052**

**Sacral Insufficiency Fractures in Postmenopausal Women with Low Back and Buttock Pain: Prevalence and Risk Factor Analysis in 752 Patients**

*Ye-Soo Park[1], Hyun-Jun Kim[2], Jaewan Soh[3]*
*[1]Guri Hospital- Hanyang University College of Medicine, Department of Orthopaedic Surgery, Guri city- Gyunggi-do, Korea- South*
*[2]Guri Hospital- Hanyang University College of Medicine, Department of Orthopaedic Surgery, Guri city, Korea- South*
*[3]Guri Hospital- Hanyang University College of Medicine, Department of Orthopaedic Surgery, Guri,*

*Korea- South*

**Abstract Text**

**Background/Introduction:** Sacral insufficiency fractures (SIF) are often overlooked as a cause of low back and buttock pain. However, their prevalence and risk factors have been poorly reassessed in aging populations. This study aimed to evaluate the prevalence and identify the risk factors of SIF in postmenopausal women within an aging population.

**Methods:** A retrospective study included 752 postmenopausal women who presented with vague low back or buttock pain between January 2021 and March 2024. All patients underwent bone scans with single-photon emission computed tomography (SPECT)/CT. Demographic, laboratory, and radiological findings were compared between the SIF and non-SIF groups. Risk factors were analyzed using logistic regression, and the serum vitamin D cutoff value was determined via ROC analysis.

**Results:** Among 752 patients (mean age: 74.4 ± 8.8 years), 48 (6.4%) were diagnosed with SIF. Univariate analysis identified age ≥ 80 years, cognitive disorders, compression fractures ≥ 3, osteoporosis, and lower vitamin D levels as significant risk factors. Multivariate analysis confirmed age ≥ 80 years (OR: 3.736 p = 0.043), osteoporosis (OR: 2.220, p = 0.029), and low vitamin D levels (OR: 0.913, p < 0.001) as independent risk factor. Vitamin D levels ≤ 20.8 ng/mL was the cutoff for low vitamin D (AUC of 76.7). Multiple risk factors increased SIF prevalence: 1.7% with no risk factors, 3.7% with one, 11.0% with two, and 31.7% with three (p < 0.001).

**Conclusion:** In an aging society, the incidence of SIF is 6.4%. Key risk factors include age ≥ 80 years, osteoporosis, and low serum vitamin D levels (≤ 20.8 ng/mL). Managing osteoporosis and maintaining serum vitamin D above this threshold are crucial. SIF should be considered in patients with vague low back or buttock pain, especially with risk factors.

**P053**

**Osteoblast-specific rank in developing zebrafish skeletal structures**

*Arianna Gomez[1], Joyce Tang[1], Priscilla Boatemaa[1], Edith Gardiner[1], Ron Kwon[1]*
*[1]University of Washington, Orthopaedic Surgery and Sports Medicine, Seattle, United States*

**Abstract Text**

RANK is a central regulator of osteoclastogenesis which has long been thought to lack expression in stromal cells or osteoblasts and therefore have no direct role in bone formation. Recently, mice with conditional knock-out of Rank in mesenchyme-derived cells have been shown to have elevated rates of bone formation. This osteoanabolic effect was attributed to RANK expression in bone mesenchymal stem cells (BMSCs), where it inhibits osteogenic differentiation. However, a direct role for RANK in regulating bone formation remains highly controversial due to a lack of studies identifying RANK+ osteoprogenitors *in vivo*. Here, we defined the expression of *tnfrsf11a* (*rank*), whose human ortholog encodes RANK, in the developing zebrafish skeleton. Analysis of a scRNA-seq atlas of developing zebrafish revealed that *rank* is differentially expressed in osteoblasts compared to >150

cell types. HCR RNA-FISH revealed expression of *rank, sp7* (osterix, an osteoblast-specific transcription factor), and *col27a1a* (a marker of developing bone and cartilage) localized to the developing operculum at 3-, 5-, 8-, and 10-days post fertilization (dpf). The overlap of *rank* expression with *sp7*, in concert with the fact that osteoclasts are not detectable at these time points, suggest that *rank* expression is in the osteoblast lineage. Moreover, *rank* co-localized with *sp7* rather than *runx2a/b*, suggesting that *rank* is expressed in committed osteoprogenitors. We also identified *rank* expression in the ceratobranchials, hyomandibula, cleithrum, and branchiostegal rays, indicating that *rank* is expressed in bones undergoing dermal and endo/perichondral ossification. We conclude that *rank* is highly and specifically expressed in osteoblastic cells in zebrafish, functionally serving as an osteoblastic biomarker during skeletal development. These findings broaden our understanding of RANK expression in osteoblasts in vertebrates and highlight the potential of an ancient role of RANK in bone formation, which could have implications on the ability to target RANK for anabolic therapies.

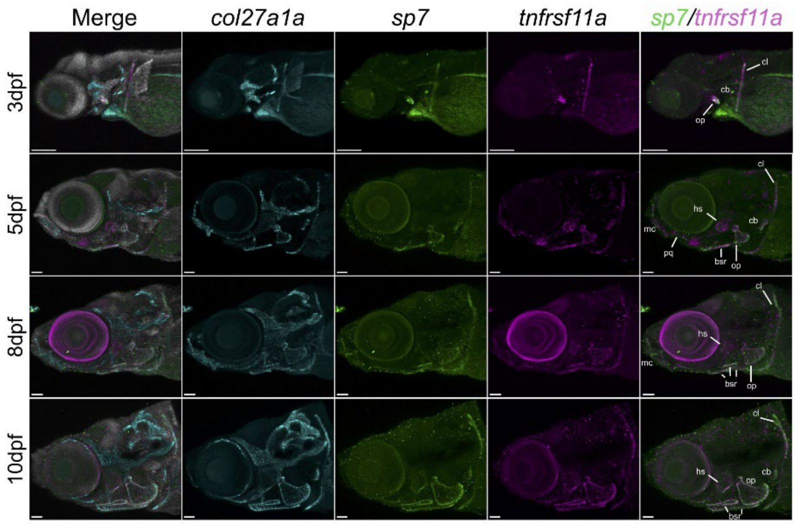

Fig 1. Expression of *rank* in developing craniofacial structures. Images of HCR RNA-FISH stained zebrafish from 3 to 10 days post fertilization (dpf). Structures that co-express *tnfrsf11a* (*rank*) and *sp7* (*osterix*) are labeled: branchiosteigal rays (bsr), ceratobranchials (cb), cleithrum (cl), hyosymplectic (hs), Meckel's cartilage (mc), operculum (op), and palatoquadrate (pq). Scale bars are 50µm

**P054**

### Age- and sex-dependent role of $\beta_2$-adrenoceptor-signaling in chondrocytes for long bone growth and bone metabolism

*Melanie Rebecca Kuhn*[1,2], *Melanie Haffner-Luntzer*[1], *Sandra Dieterich*[1], *Katarina Teresa Acker*[1], *Anita Ignatius*[1], *Stefan Oskar Reber*[3], *Miriam Eva Angelica Tschaffon-Müller*[1]
[1]*Institute of Orthopedic Research and Biomechanics, University Medical Center Ulm, Ulm, Germany*
[2]*Department of Orthopedic Trauma, University Medical Center Ulm, Ulm, Germany*
[3]*Laboratory for Molecular Psychosomatics - Department of Psychosomatic Medicine and Psychothera py, University Medical Center Ulm, Ulm, Germany*

**Abstract Text**

**Background/Introduction:** Adrenergic signaling influences bone metabolism and fracture healing, with catecholamines impairing endochondral ossification during these processes. The $\beta_2$-adrenoceptor ($\beta_2$-AR) is crucial in mediating the effects of catecholamines on bone. Systemic or osteoblast-specific $\beta_2$-AR knockout mice display increased bone mass.

**Purpose:** This study examined the age- and sex-dependent effects of a chondrocyte-specific $\beta_2$-AR knockout on long bone growth and bone metabolism in mice.

**Methods:** Male and female $Adrb2^{Col2a1\text{-}Cre}$ mice with a specific $\beta_2$-AR knockout in chondrocytes were compared to their $Cre^-$ littermates ($Adrb2^{fl/fl}$). At 11-12 and 40 weeks of age, femur lengths and growth plates were investigated. Bone metabolism of femora was assessed using histology and µCT. Statistics: t-test/Mann-Whitney-U-Test. p<0.05. n=4-6. Ethical approval by local government.

**Results:** 11-12-week-old male and female $Adrb2^{Col2a1\text{-}Cre}$ mice exhibited shorter femora than the controls (males: 14.9 vs 15.8mm, p<0.0001; females: 14.2 vs 14.8mm, p=0.021). In contrast to females, males also showed an enlarged hypertrophic zone (31.9 vs 25.2µm, p=0.009) and more hypertrophic chondrocytes (35.1 vs 23.2%, p=0.031) in the growth plate. 40-week-old male and female $Adrb2^{Col2a1\text{-}Cre}$ mice both displayed a decreased relative bone volume (Fig.1A,B) and less osteoblasts (Fig.1C,D). This osteopenic phenotype was not observed in 11-12-week-old mice.

**Conclusion:** Our findings indicate an age- and sex-dependent role of $\beta_2$-AR-signaling in chondrocytes for long bone growth and bone metabolism. At juvenile age, bone growth is disturbed in knockout mice, with a greater effect in males than in females. Only at higher ages, an additional osteopenic phenotype is seen regardless of sex. The impaired bone growth is very likely due to an accelerated transition of proliferative to hypertrophic chondrocytes. Osteopenic bone phenotype in elderly mice could be due to changes in chondrocyte-derived-osteoblasts, which are important for secondary bone formation in long bones. Further research is needed to clarify the mechanisms and how sex hormones might influence the phenotype.

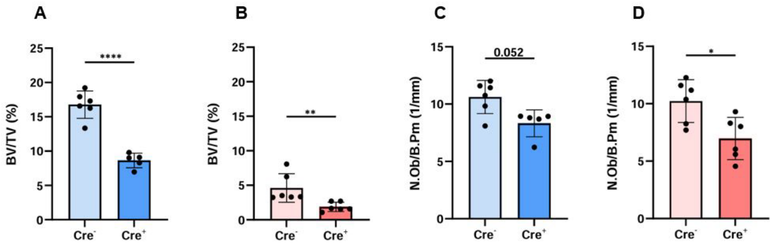

**Figure 1:** Bone metabolism in 40-week-old $Adrb2^{Col2a1\text{-}Cre}$ mice ($Cre^+$) and $Adrb2^{fl/fl}$ control mice ($Cre^-$). **A,B)** Bone volume per tissue volume (BV/TV) and **C,D)** number of osteoblasts per bone perimeter (N.Ob/B.Pm) in femora of males (blue) and females (red). *p<0.05. **p<0.01. ****p<0.0001. n=5-6.

### P055

### Impaired neoangiogenesis causes reduction of ossification at the chondro-osseous interface in Syndecan-1 deficient mice

*<u>Melanie Timmen</u>[1], Christian Arras[2], Nils Roters[1], Daniel Kronenberg[1], Seyedehelnaz Enderami[1], M. Gabriele Bixel[3], Ralf H. Adams[3], Richard Stange[4]*

[1]*Institute of Musculoskeletal Medicine- University of Muenster,*
*Department of Regenerative Musculoskeletal Medicine, Muenster, Germany*
[2]*University Medical Centre Hamburg- Eppendorf,*
*Department of Trauma- Hand and Reconstructive Surgery, Hamburg, Germany*
[3]*Max Planck Institute for Molecular Biomedicine, Department of Tissue Morphogenesis, Muenster, Germany*
[4]*University Hospital Muenster- University of Muenster,*
*Department of Regenerative Musculoskeletal Medicine, Muenster, Germany*

**Abstract Text**

**Background:** Neoangiogenesis drives the replacement of mineralized cartilage by trabecular bone during bone growth regulated by molecules like e.g. VEGF, OPG and RANKL. The Heparan-sulfate-proteoglycan Syndecan-1 (Sdc1) while interacting with VEGF and OPG, plays a role in the communication of osteoclasts and osteoblasts and in the development of blood vessels. Syndecan-1-deficient bones show reduced blood vessel invasion in chondro-osseous interfaces like bone epiphysis and fracture callus in vivo and impaired microvessel formation in vitro.

**Purpose:** To understand the consequences of impaired vessel invasion in Sdc1 deficient bones we analysed the ossification near the chondro-osseous borders in native bones and fracture callus in mice.

**Methods:** Native femora of WT (n=11) and Sdc1-/- (n=13) mice were used for bone analysis. Additionally, female mice (WT n=6-14, Sdc1-/- n=6-8, per time point) underwent midshaft femur fracture intramedullary stabilized (Ethic-app: 81-02.04.2019.A164). Bony structures were analysed using microCT and histology (Two-Photon-microscopy) with the focus at the chondro-osseous interface. Vascularisation was visualised using IF staining.

**Results:** Sdc1-deficient mice showed a significant decreased number of vessel buds (15buds/mm) at the chondro-osseous border (growth plate) compared to WT (25buds/mm). After fracture, a decreased number of vessel buds invading at the borderline of cartilage to bone in the callus were measured in Sdc1-deficient callus tissue. Areas of reduced blood vessel invasion showed reduced collagen fibers pointing to a reduction in ossification. Quantification of this region revealed a significant reduction (Sdc1-/-:34%, WT:52%) of early bone formation in Syndecan-1-deficient tissue.

**Conclusion:** Syndecan-1 has a significant impact on neoangiogenesis at the chondro-osseous border in bone as well as during bone healing. Impaired vessel invasion led to a reduction in formation of trabecular bone due to less collagen fiber formation and ossification. Understanding the regulatory part of Syndecan-1 may reveal a new target to treat bone formation disorders like osteoporosis or fracture healing disturbances.

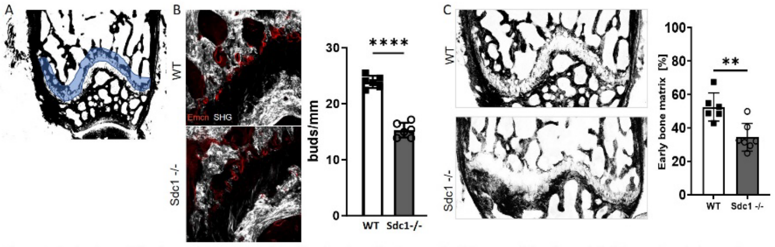

Figure 1: Reduction of blood vessel invasion and ossification in native bones of wild type and Syndecan-1 deficient mice. A Area of interest (blue) at the chondro-ossous interface of a femur aged 4 month. B: Neoangiogenesis starts ossification process by vessel buds invasion into area of hypertrophic chondrocytes. Syndecan-1 deficiency impairs vessel invasion process. C: Formation of ossified trabecular bone at the interface of cartilage to bone which is reduced in Syndecan-1 deficient bones.

**P056**

**Elucidating the Impact of the ACAN p.N178Y Mutation on Growth Plate Function and Bone Development**

*Chen Abramovitch Dahan*[1], *Sveta Katchkovsky*[2], *Ateret Levi*[2], *Aviv Moyal*[2], *Noam Levaot*[2]
[1]*Ben Gurion University of the Negev, Biology and physiology of the cell, Beer Sheva, Israel*
[2]*Ben Gurion University of the Negev, Biology and physiology of the cell, Beer Shave, Israel*

**Abstract Text**

Aggrecan (ACAN), a key proteoglycan in the cartilage extracellular matrix, plays a critical role in growth plate function and bone elongation. Homozygous ACAN mutations in mice severely disrupt these processes, leading to tightly packed chondrocytes with minimal intervening matrix. These disruptions correlate with advanced bone age, premature growth cessation, and early epiphyseal fusion observed in individuals with ACAN mutations. A specific mutation in the N-terminus, p.Asn178Tyr (p.N178Y), was identified in children presenting with marked short stature; however, the causative effect and underlying mechanisms of this mutation remain poorly understood.To investigate the impact of the ACAN p.N178Y mutation on growth and elucidate its pathological mechanisms, we generated a knock-in (KI) mouse model harboring this mutation. Body weight and length were monitored weekly in wild-type (WT) and KI mice for 12 weeks. While WT females demonstrated continuous body elongation throughout this period, homozygous p.N178Y mice exhibited growth arrest around six weeks, resulting in significantly shorter body and tibial lengths. Histological analysis of six-week-old mice revealed a pronounced reduction in the hypertrophic zone (HZ) area and thickness within the growth plates of p.N178Y mutants.These findings represent the first demonstration of a genomic model that replicates the human phenotype, highlighting a critical difference in growth plate organization between WT and KI mice. The disrupted growth plate structure may underlie the impaired skeletal development observed in both mice and humans with the p.N178Y mutation. This model establishes a foundation for future studies to explore the pathological mechanisms driving growth retardation caused by ACAN p.N178Y and potentially other aggrecan mutations in humans.

**P057**

**TSG-6 promotes critical-sized bone repair and modulates osteogenesis**

*Verena Fischer*[1], *Oliver Kueppers*[1], *Mubashir Ahmad*[1], *Jasmin Buelow*[1], *Melanie Haffner-Luntzer*[1], *Karin Scharffetter-Kochanek*[2], *Anita Ignatius*[1]
[1]*University Medical Center Ulm, Institute of Orthopedic Research and Biomechanics, Ulm, Germany*
[2]*University Medical Center Ulm, Department of Dermatology and Allergic Diseases, Ulm, Germany*

**Abstract Text**

Critical-sized bone defects are associated with impaired regeneration and non-union, requiring new osteoanabolic therapies. TNF-stimulated gene 6 (TSG-6), an anti-inflammatory and pro-regenerative protein secreted by mesenchymal stem cells (MSCs), promotes wound healing. Here, we investigated whether TSG-6 stimulates bone repair *in vivo* and regulates osteogenesis *in vitro*.

Male C57BL/6J mice (12-weeks-old) received a 1.5 mm femoral critical-sized defect, which was treated with 50 µg recombinant human TSG-6 delivered in a type-1 collagen gel. Empty defects and collagen gel served as controls. Bone repair was assessed on d35 using µCT analysis and histomorphometry, while the local immune response was analysed on d3 by flow cytometry (n=8/group). *In vitro*, TSG-6 was knocked down in primary murine MSCs using siRNA, and osteogenesis was analysed by ALP-staining, qPCR, and RNA-Seq on d6 (n=4/group). Statistics: Student's t-test or ANOVA/Fisher's LSD ($p<0.05$).

*In vivo*, rhTSG-6 significantly enhanced bone repair on d35, as evidenced by increased bone volume (empty: 0.4 $mm^3$; collagen gel: 0.6 $mm^3$; rhTSG-6: 1.2 $mm^3$), cortical bridging (empty: 13%; collagen gel: 37.5%; rhTSG-6: 75% healed, respectively), and osteoblast numbers, while osteoclasts were reduced compared to both control groups. On d3, rhTSG-6-treated mice exhibited more M2-macrophages in the hematoma compared to empty ($p=0.001$) and collagen gel ($p=0.038$) controls. *In vitro*, TSG-6-KO impaired osteogenic differentiation of murine MSCs, as indicated by reduced expression of osteogenic marker genes (*Alpl, Bglap, Sp7*) and diminished mineralization ($p=0.0001$). Metascape analysis revealed the extracellular matrix organization pathway as the most prominently affected, with matrix metalloproteinase-13 (*Mmp-13*) identified as the most highly regulated gene in response to TSG-6-KO.

Our results showed that TSG-6 improves bone repair in a pre-clinical model and positively regulates osteogenesis *in vitro* by regulating matrix mineralization and modulating *Mmp-13* gene expression. These findings imply that TSG-6 treatment could be a new therapeutic approach to enhance bone repair in clinical settings.

**P058**

### (-)-epicatechin is an effective treatment to correct both defective bone growth and repair in the Hypochondroplasia mouse model

*<u>Charline Vilpreux</u>[1], Anne Morice[2], Amélie de La Seiglière[2], Geneviève Baujat[3], Konstantina Grossios[4], Kostas Kaloulis[4], Laurence Legeai-Mallet[1]*
*[1]Imagine Institut University Paris Cité, Inserm U1163, Paris, France*
*[2]Imagine Institut, Inserm U1163, Paris, France*
*[3]Hôpital Necker-Enfants, Département de Génétique, Paris, France*
*[4]Epicure Biotechnologies, EPFL Innovation Park, Lausanne, Switzerland*

**Abstract Text**

**Introduction:** Hypochondroplasia (HCH) is a rare autosomal dominant skeletal condition characterized by disproportionate short stature with short arms and legs, macrocephaly, relatively normal facies, and, variable degree of learning difficulties.. HCH is caused by a dominant gain-offunction mutations in the fibroblast growth factor receptor 3 (*FGFR3)* gene, most notably the N540K mutation. Currently, there is no specific treatment for HCH. Previously, we demonstrated that strict control of *FGFR3* signaling by (-)-epicatechin provides a rationale for developing it as therapy for *FGFR3*-related disorders.

**Objectives:** The objectives of this study were to determine 1) the effect of (-)-epicatechin in *Fgfr3*$^{N534K/+}$mice (HCH mice) on bone growth and 2) on mandibular bone repair. (-)- epicatechin, was administered by daily sub-cutaneous injection (s.c.) from day 1 and for 31 days, at 0.1mg/ kg /day to HCH mice and compared to vehicle HCH/wild type littermates. The non-stabilized fractures were performed at 6 weeks of age on the right mandible in HCH mice (three months of age). At day 0 *post* fracture, subcutaneous injections were performed with (-)-epicatechin (0.3 mg/kg) three times/week, during 28 days.

**Results/Conclusion:** (-)-epicatechin administered to HCH mice statistically increased the length of femur +6.2% (p= 0.0020), tibia +3.7% (p=0.0021), humerus +4.1% (p=0.0052), ulna +3.0% (p=0.0136), and lumbar segments L4/L6 +7.5% (p=0.0489) (Figure). (-)-epicatechin improved bone repair of the mandible with a +7.88% (p=0.0348) increase in BV/TV value of calluses and -33.54% (p=0.0017) reduction in callus volume at D28 *post* fracture in HCH mice. Interestingly, no pseudarthrosis was observed in mandibular calluses of HCH-treated mice.

Here, we concluded that that daily treatment with s.c. (-)-epicatechin improved the bone growth deficit and rescued the defect in mandibular endochondral bone repair in HCH mice. Altogether, these results provide a rationale to use of (-)-epicatechin for improving bone growth for children with HCH.

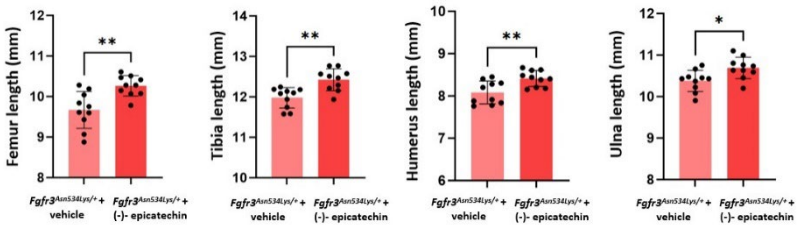

Figure: Improvement of the long bones length with (-)-epicatechin treatment.

**P060**

**The Impact of Hormone Replacement Therapy on Bone Mineral Density after Hematopoietic Stem Cell Transplantation**

*Jung Yoon Park[1], Park Hyoung Moo[2], Song Jae-Yen[1]*
*[1]Seoul St. Mary's Hospital- College of Medicine- The Catholic University of Korea- Seoul- Korea., Department of Obstetrics and Gynecology, Seoul, Korea- South*
*[2]Menopause Clinic Grace Women's Hospital, bDepartment of Obstetrics and Gynecology, Goyang, Korea- South*

**Abstract Text**

**Objectives:** This study aimed to evaluate the effects of hormone replacement therapy (HRT) on bone mineral density (BMD) in young women who have undergone allogeneic hematopoietic stem cell transplantation (HSCT).

**Methods:** This retrospective cohort included 230 female patients with premature ovarian insufficiency (POI) who underwent allogeneic HSCT between April 2009 and April 2016 at Seoul St. Mary's Hospital in Seoul, Korea. Inclusion criteria included adult patients who were age 40 years or younger at the time of transplantation and were followed for at least 3 years after HSCT.

**Results:** At the first and second years after HRT initiation, the HRT group showed a significant increase in lumbar spine BMD compared to the non-HRT group. Furthermore, the BMD of the lumbar spine significantly increased after 1 and 2 years of HRT significantly. The BMD of the femoral neck and total hip also exhibited a significant percentage increase from baseline after 2 years of HRT. These findings were consistent, regardless of the presence of GVHD or steroid exposure. Subgroup analysis revealed that initiating HRT within 12 months after HSCT yielded the greatest increase in lumbar spine BMD after 2 years of therapy.

**Conclusion:** Hormone replacement therapy demonstrated significant positive effects on bone mineral density in young women who underwent allogeneic HSCT. The improvements in lumbar spine, femoral neck, and total hip BMD were observed even in the presence of GVHD or steroid exposure. Initiating HRT within 12 months post-HSCT appears to be associated with the greatest increase in lumbar spine BMD after 2 years. These findings highlight the potential of HRT as a therapeutic intervention to mitigate bone loss and improve bone health in this population.

**P061**

**Body Composition and Bone Mineral Density in Postmenopausal Women with Advanced Knee Osteoarthritis Undergoing Surgical Treatment**

*Jung Yoon Park*[1], *Park Hyoung Moo*[2], *Kim Mee-Ran*[1]
[1]*Seoul St. Mary's Hospital- College of Medicine- The Catholic University of Korea- Seoul- Korea., Department of Obstetrics and Gynecology, Seoul, Korea- South*
[2]*Menopause Clinic Grace Women's Hospital, Department of Obstetrics and Gynecology, Goyang, Korea- South*

**Abstract Text**

**Objectives:** This study aimed to demonstrate the bone mineral density (BMD) and body composition in postmenopausal women with knee osteoarthritis (OA) who underwent surgical treatment, such as total knee arthroplasty, osteotomy, or meniscectomy.

**Methods:** A total of 254 women with OA aged 50 years who underwent surgical treatment were enrolled in this study. We evaluated obesity-related factors, muscle components, and BMD using dual-energy X-ray absorptiometry.

**Results:** No significant differences were noted in the BMD of the hip joint between the symptomatic side of the leg with knee OA and the contralateral side. However, when comparing the BMD of each component, the results indicated a significantly higher BMD in the obesity group based on body mass index (BMI). When defining sarcopenic obesity (SO) using various indicators of obesity (BMI, the estimated visceral adipose tissue area, android/gynoid ratio, and total body fat percentage), the prevalence of SO in the OA group who underwent surgical treatment ranged from 22.0% to 49.6%.

**Conclusions:** This study investigated obesity-related factors in patients with advanced knee OA who underwent surgery, revealing a high prevalence of overweight/obese individuals, the presence of SO, and a complex relationship between obesity, body composition, and bone density, highlighting the potential protective effects of weight-bearing on bone health while exploring the impact of sarcopenia on bone density differences in the context of OA. Depending on various definitions of obesity, diverse proportions of SO in patients with OA have been observed, and further detailed research is required to understand its impact on the condition.

**P062**

**Five-Year Sales trend for osteoporosis medication in Korea: A market share analysis from 2018-2023**
**Running head: Trends of Osteoporosis treatment in Korea**

*Hyoung-Moo Park*[1]*, Jung Yoon Park*[2]*, KIm Mee-Ran*[2]
[1]*Menopause Clinic Grace Women's Hospital, Department of Obstetrics and Gynecology, Goyang-si, Korea- South*
[2]*Seoul St. Mary's Hospital- College of Medicine- The Catholic University of Korea- Seoul- Korea., Department of Obstetrics and Gynecology, Seoul, Korea- South*

**Abstract Text**

**Objective**s: Osteoporosis is the most common chronic disease that occurs after menopause in women. The purpose of this study was to investigate the current status of use of osteoporosis drugs in Korea by figuring out the size of osteoporosis drugs sold in Korea from 2018 to 2023.

**Methods:** Data Based on Intercontinental Marketing Services (IMS) for 5 years between 2018 to 2023, the sales amount of osteoporosis drugs was calculated. Specific pharmacologic drugs were analyzed included bisphosphonates, raloxifene, parathyroid hormone, denosumab and others. In November 2019, Romosozumab was newly approved by the Ministry of Food and Drug Safety for use as a treatment for osteoporosis and was included in this analysis.

**Results:** The total osteoporosis drug market sales in 2023 was $284 million, a 63% increase compared to $174 million in 2018. In an evaluation of the annual growth rates as of 2023, romosozumab demonstrated a significant increase of 59% relative to the previous year, whereas denosumab exhibited a growth rate of 31%. Until 2022, bisphosphonates represented the largest proportion of osteoporosis treatments; however, as of 2023, denosumab has become the leading treatment in terms of sales.

**Conclusion:** The analysis of osteoporosis drug sales in Korea from 2018 to 2023 demonstrates significant market trends. Total sales surged to $284 million in 2023, a remarkable 63% increase from 2018. Romosozumab, approved in November 2019, experienced a notable 59% sales growth, while

Denosumab, with a growth rate of 31%, emerged as the leading treatment by 2023, surpassing bisphosphonates. These findings reflect the evolving landscape of osteoporosis management in Korea, emphasizing the adoption of newer therapeutic options.

**P063**

**Zebrafish as a Model for Craniosynostosis: Generating Loss-of-Function Mutants in Genes Regulating Calvarial Formation**

*Lital Ashtamkar Matok*[1], *Paola Nicoletti*[2], *Ramon Birnbaum*[3], *Inga Peter*[2], *David Karasik*[1]
[1]*Bar-Ilan University, The Azrieli Faculty of Medicine, Zefat, Israel*
[2]*The Icahn School of Medicine at Mount Sinai, Medicine, New York, United States*
[3]*Ben-Gurion University, Department of Life Sciences, Be'er Sheva, Israel*

**Abstract Text**

**Background**: Intramembranous ossification, characterized by the condensation of mesenchymal cells, underlies the formation of the skull bones. Disruptions in this process can result in congenital disorders such as craniosynostosis (CS) and craniofacial malformations. A recent GWAS has identified the distal-less homeobox 5/6 (*DLX5*/*DLX6*) locus on 7q21.3 to be associated with non-syndromic CS. Based on the evolutionary conservation, we hypothesized that zebrafish could serve as a valuable model to investigate the impact of *dlx5a* and *dlx6a* loss-of-function on cranial vault development.

**Methods:** Stable knockout (KO) lines for *dlx5a*, *dlx6a*, and *dlx5a6a* double knockout were generated using CRISPR-Cas9 technology and progressed for 3 generations. Cranial suture development was monitored in each stable KO genotype every 3-5 days from 45 to 60 days post-fertilization (dpf) by in vivo calcein staining; standard length and weight were measured. At 60 dpf, fish were fixed, stained with 1% silver nitrate, and scanned using micro-CT (µCT).

**Results**: *dlx5a -/-* mutants exhibited delayed sutural development evidenced by both calcein staining and µCT analysis compared to wildtype, with 50% displaying an abnormal suture phenotype; demonstrated significantly reduced survival rates (1-10 dpf) and lower weight at 60 dpf. *dlx6a -/-* mutants exhibited significantly reduced survival (1-10 dpf), body length and weight (60 dpf). Additionally, *dlx6a -/-* mutants displayed delayed suture development. In *dlx5a6a* --/-- double KOs, sutural development was further compromised compared to wildtype sibling (*dlx5a6a* ++/++) and single KOs. These *dlx5a6a* --/-- mutants exhibited significantly reduced standard body length, weight, and survival rates. Notably, 70% of double KOs displayed severe neurocranial and viscerocranial deformities (Figure 1) vs. 0% to 33.7% in single KO lines.

In conclusion, KO of candidate CS genes DLX5 and or DLX6 results in abnormal development, characterized by either reduced body size and or delayed cranial vault development in surviving fish.

**Figure.1 Craniofacial deformities of *dlx5a6a* --\-- zebrafish** (left: calcein in-vivo, right: µCT image after staining with 1% silver nitrate)
A) 60 dpf: Parasphenoid shown in red bar, dentary-basihyal angle in blue. B) Cranial sutures of WT and double KO.

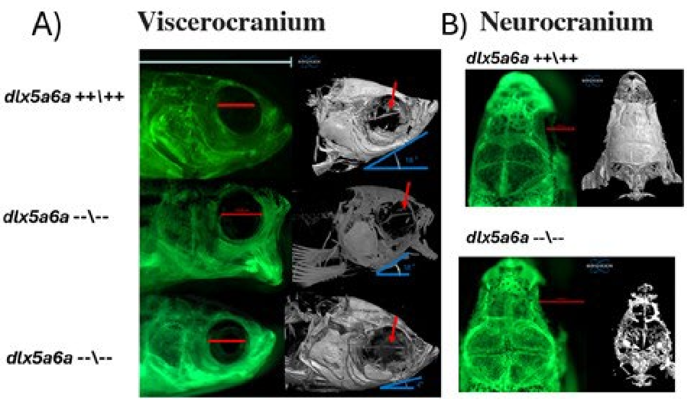

**P064**

**Effects of hypoxia-mimicking compounds on the energy metabolism of bone marrow stromal cells during osteogenic differentiation**

*Yudong Zhao[1], Marijke Koedam[1], Johannes P.T.M. van Leeuwen[1], Bram C.J. van der Eerden[1]*
*[1]Erasmus University Medical Center, Internal Medicine, Rotterdam, Netherlands*

**Abstract Text**

**Background:** Hypoxia plays a crucial role in the early stages of fracture healing, significantly affecting bone marrow stromal cells (BMSCs). Simulating hypoxia in vitro is essential for studying cellular responses, including metabolic effects. While physical hypoxia can be replicated using specialized equipment like hypoxia chambers, hypoxia-simulating compounds offer a cost-effective, stable and accessible alternative.

**Purpose:** This study aims to compare the responses of exposing BMSCs to 3% oxygen versus three hypoxia-simulating compounds: dimethyloxalylglycine (DMOG), deferoxamine (DFO), and cobalt chloride (CoCl2).

**Methods:** We applied compound concentrations ranging from 0.16 µM to 500 µM to osteogenic BMSCs, alongside 3% oxygen treatment. Cell counts, protein and alkaline phosphatase assays were conducted to evaluate BMSC viability and differentiation. qPCR analysis assessed hypoxia and osteogenic markers, while Flash-Seq studies focused on differentially expressed genes (DEG)-mediated identification of hypoxia-related cellular pathways. Seahorse mitochondrial stress tests were conducted to assess metabolic effects by the treatments.

**Results:** Our findings indicate that osteogenically differentiated BMSCs respond similarly to hypoxia-inducing compounds versus 3% oxygen as shown by dose-dependent upregulation of hypoxia markers and downregulation of osteogenic markers ($p < 0.05$). Cell counts showed no significant differences across treatments compared to controls. All three compounds strongly inhibit osteogenic differentiation as showing by ALP activity. Mitochondrial stress tests revealed distinct EC50 values, with DMOG being the most potent. Metabolic reprogramming induced by the hypoxic compounds inhibited mitochondrial respiration and enhancing glycolysis. KEGG enrichment analysis confirmed that DEGs regulated by all compounds were significantly associated with hypoxia-related pathways ($p < 0.05$).

**Conclusions:** Our results show that all three hypoxia-mimicking compounds closely resemble the effects of 3% oxygen on osteogenic BMSCs, providing possibilities towards using these as alternatives to overcome physical hypoxia limitations in in vitro studies.

**P065**

**Mandibular cortical thickness is associated with skull mineral density in adolescents**

*Tarsila Figueiredo*[1], *Vid Prijatelj*[2], *Olja Grgic*[1], *Carolina Medina-Gomez*[2], *Eppo Wolvius*[1], *Lea Kragt*[1], *Fernando Rivadeneira*[2]
[1]*Erasmus Medical Center,*
*Department of Oral and Maxillofacial Surgery- Special Dental Care and Orthodontics, Rotterdam, Netherlands*
[2]*Erasmus Medical Center, Department of Internal Medicine, Rotterdam, Netherlands*

**Abstract Text**

**Background**: Maximizing bone mineral density (BMD) before peak bone mass is reached is an effective way to prevent osteoporosis later in life. The assessment of mandibular cortical thickness from dental panoramic radiographs (DPRs) has been proposed as a tool for assessing low BMD. Skull BMD (SK-BMD) is highly heritable and efficiently captures the genetic variation at other clinically-relevant skeletal sites.

**Purpose**: The aim of this study was to analyze the relationship between mandibular cortical thickness and SK-BMD in adolescents. The association established in an observational setting was assessed using the Mendelian Randomization (MR) framework underlying polygenic risk scores (PRS).

**Methods**: This multiethnic population-based study included 2672 children (1268 boys; 47.5%) aged 13 years from the Generation R Study. SK-BMD was derived from dual-energy X-ray absorptiometry (DXA) total body scans and instrumented using a weighted PRS. Mandibular cortical thickness from DPRs was quantified with the mental index (MI) and the superior panoramic mandibular index (sPMI). The association between SK-BMD and mandibular cortical thickness was analyzed with a linear regression corrected for age, BMI, sex, ethnicity and puberty. Participants of genetically-determined European ancestry (N=2118) were allocated along the wPRS distribution. The characteristics of participants allocated to top and bottom quintiles were compared using a MR framework.

**Results**: Each standard deviation (SD) increase of SK-BMD was associated (p-value$<2x10^{-16}$) with 0.11 increase of sPMI and 0.23 MI in fully adjusted models. When the characteristics of the participants were examined stratified across the extreme quintiles of the SK-BMD wPRS distribution, SK-BMD (pvalue<0.001), sPMI (p-value=0.032), and MI (p-value<0.001) significantly differ between extremes while all other parameters were randomized.

**Conclusion**: Our findings suggest that mandibular cortical thickness parameters measured from DPRs constitute unconfounded assessments of skull BMD and may serve for the evaluation bone health status in adolescents from the general population.

**P066**

**Loss-of-Function Mutations in the ALPL Gene Presenting with Low Serum Concentrations of Total Alkaline Phosphatase**

*Sanjay Kumar Bhadada*[1], *Vandana Dhiman*[2]
[1]*PGIMER- Chandigarh, Endocrinology, Chandigarh, India*
[2]*PGIMER, Endocrinology, Chandigarh, India*

**Abstract Text**

**Introduction:** Hypophosphatasia (HPP) is a rare inherited disorder characterized by bone pains and low circulating concentrations of total alkaline phosphatase (ALP) caused by mutations in ALPL. Severe HPP presents in childhood but milder forms can present in adulthood. The prevalence and clinical features of adult HPP are poorly defined. The aim of this study was to evaluate the prevalence and clinical significance of low serum total alkaline phosphatase (ALP) levels in a clinic-based population of adult osteoporotic patients.

**Methodology:** We screened for patients with low ALP in Indian cohort of two families patients and performed mutation screening of ALPL in those with low ALP (≤40 U/L). These individuals were age matched clinic controls with a normal ALP.

**Result:** We find out serum low ALP and ALPL mutations in 6/13 individuals from patients. We identified mutation on heterozygous, missense mutation in exon 5 at position 311 (c.311a >g;p.104 Asn > Ser) of ALPL gene in the three members and other family present mutation heterozygous, missense mutation in exon 5 at position (c.455g>a, p.Arg152His ) of ALPL gene in the three members with low ALP and had potentially pathogenic variants in ALPL.

**Discussion & Conclusion:** Seven patients with low ALP, including 6 with ALPL mutations.We conclude that heterozygous loss-of-function mutations in ALPL are common in osteoporosis patients with low ALP.

**P069**

**Investigating the effect of aging on the mechano-sensitivity of bone regeneration in a mouse model of premature aging.**

*Neashan Mathavan*[1], *Graeme Paul*[1], *Sara Lindenmann*[1], *Stefanie Wissmann*[1],

*Francisco Correia Marques[1], Dilara Yilmaz[1], Gisela A. Kuhn[1], Esther Wehrle[2], Ralph Müller[1]*
*[1]ETH Zürich, Institute for Biomechanics, Zürich, Switzerland*
*[2]AO Research Institute Davos, Bone Biology Focus Area, Davos Platz, Switzerland*

**Abstract Text**

**Background/Introduction:** The mechanical environment is of critical importance to the regenerative response in bone. However, no consensus exists on the effect of aging on the mechano-sensitivity of bone regeneration. Moreover, our understanding of sexual dimorphism in mechanobiological mechanisms remains limited. $PolgA^{D257A/D257A}$ (PolgA) mice - a mouse model of premature aging - exhibit the premature onset of clinically-relevant musculoskeletal aging characteristics including frailty, osteosarcopenia and impaired fracture healing with age.

**Purpose:** To investigate the mechano-sensitivity of the regenerative response in aged mice of both sexes.

**Methods:** 35-week-old PolgA mice (n=15 female, n=12 male) received a mid-diaphyseal femoral osteotomy stabilized with an external fixator (license: ZH229/19, ZH033/2022). Time-lapsed *in vivo* micro-CT imaging was performed weekly at the fracture site (weeks 0-7; 10.5µm resolution). At weeks 3-7, mice were subdivided into Loaded or Control groups and received either cyclic loading (2-16N, 10Hz, 3000cycles; 3x/week) or sham-loading.

**Results:** Cyclic loading induced significantly enhanced osteogenic responses in aged mice of both sexes at weeks 5-7 (Fig 1). At week 7, BV/TV was 47.3 ±12.7% vs 91.0 ±19.2% in aged female mice ($p<0.001$) and 33.0 ±6.4% vs 78.2 ±16.3% in aged male mice ($p<0.001$) (Fig 1[A][B]). Mechanically-enhanced osteogenic responses were driven by a sustained rate of bone formation (BFR) and a diminished rate of bone resorption (BRR) between weeks 5-7 (Fig 1[C][D]). Between weeks 6-7, BFR was 0.5 ±0.2 %/day vs 1.8 ±1.0 %/day in aged female mice ($p<0.05$) and 0.5 ±0.2 %/day vs 1.4 ±0.3 %/day in aged male mice ($p<0.001$). Conversely, BRR between weeks 6-7 was 1.0 ±0.2 %/day vs 0.4 ±0.4 %/day in aged female mice ($p<0.01$) and 1.1 ±0.7 %/day vs 0.3 ±0.2 %/day in aged male mice ($p<0.05$).

**Conclusion(s):** The regenerative response in bone exhibits mechano-sensitivity with aging across both sexes in a mouse model of premature aging.

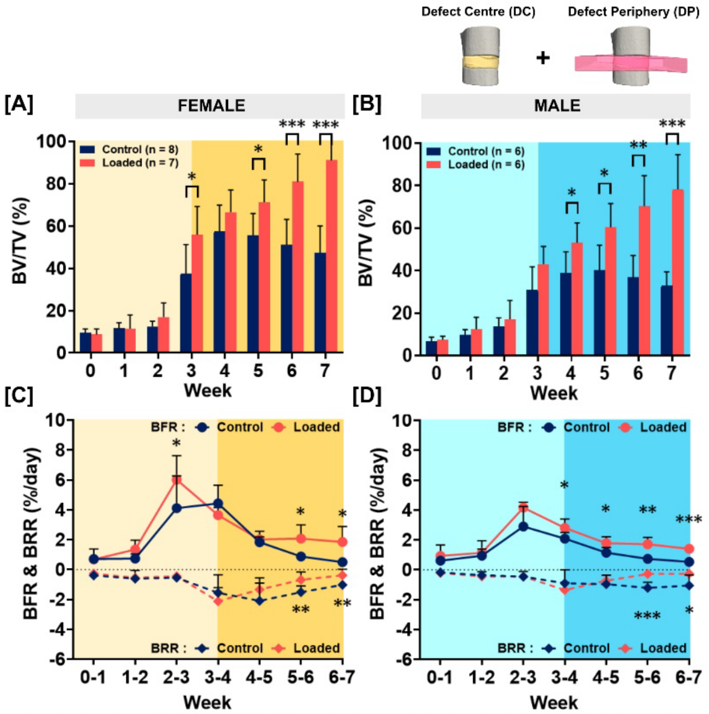

**Fig 1.** CT-derived bone morphometric parameters (BV/TV, bone formation rate BFR, bone resorption rate BRR) determined at each weekly time-point. The region of interest analyzed is the combined defect centre (DC) and defect periphery (DP) regions (visulized at the top right of the figure). Statistical significance ($p < 0.05$) between loaded vs control groups at each weekly time-point was assessed using two-way ANOVA with Geisser-Greenhouse and Bonferroni corrections.

### P070

### Quantitative Multicolored Deep Imaging of Human Bones Reveals a Composite Osteo-Sinusoidal Niche for Mesenchymal Stromal Cells

*Andrei Chagin*[1], *Nelson Chu*[1], *Ostap Dregval*[1], *Yu-Wei Chang*[2], *Giovanni Volpe*[2]
[1]*The University of Gothenburg, Medicine, Gothenburg, Sweden*
[2]*The University of Gothenburg, Physics, Gothenburg, Sweden*

### Abstract Text

Human bone marrow mesenchymal stromal/stem cells (BM-MSCs) are widely utilized in clinical trials and tissue engineering; however, their native microenvironment remains poorly understood. Here, we introduced a tissue-clearing protocol for human bones and integrated it with simultaneous mRNA and protein detection. Using this protocol, named DeepBone, we spatially mapped BM-MSCs relative to key bone microenvironment components, including human blood capillaries, adipocytes, sinusoids, and bony trabeculae. Quantitative analysis revealed that BM-MSCs are predominantly localized near vasculature in both young and aged bones but near the bone surface only in young samples. Moreover, we identified a unique microenvironment for BM-MCs in young bone, characterized by sinusoids coiled around trabeculae and enriched by ADGRL+ R-type vessels. These findings provide critical insights into the native niches of BM-MSCs, offering a foundation for the development of tissue-engineering strategies that mimic their physiological context.

**Poster Sessions: Cancer and bone**

**P071**

**Osteocyte-Driven Mechanisms in Melanoma Bone Metastasis**

*Yewei Jia[1], Fulin Zhang[1], Xianyi Meng[1], Georg Schett[1], Aline Bozec[1]*
*[1]FAU university clinic, Department of medicine clinic 3, Erlangen, Germany*

**Abstract Text**

Bone metastasis significantly worsens prognosis in melanoma, yet the role of osteocytes in promoting or restraining tumor progression within the bone environment has remained largely unexplored. In this study, we aimed to elucidate the mechanisms by which osteocytes contribute to melanoma-driven bone metastasis, identifying distinct yet complementary pathways that exacerbate tumor spread and bone destruction.

Our first key finding revealed that melanoma-stimulated osteocytes release the chemokine CXCL5, which binds to the CXCR2 receptor on melanoma cells, thus promoting their migration and invasion into bone tissue. Through in vitro and in vivo models, we demonstrated that blocking the CXCL5-CXCR2 axis—by downregulating CXCL5 in osteocytes or CXCR2 in melanoma cells—significantly reduced metastasis and osteolytic lesions, highlighting this signaling pathway as a potential therapeutic target to inhibit tumor spread within bone (Jia Y *et al* cancer letters 2024).

In parallel, our research identified a novel, non-canonical ferroptosis pathway within osteocytes induced by melanoma metastasis, mediated by HIF1α and HMOX1. This pathway contributes to osteocyte ferroptosis through autophagy-dependent degradation of ferritin, leading to iron overload and lipid peroxidation. Inhibiting HMOX1 with Znpp preserved osteocyte viability and bone structure, substantially mitigating the osteolytic effects associated with melanoma progression in bone (Jia Y *et al* Bone Research accepted).

Our findings establish a dual role for osteocytes in melanoma bone metastasis, where they actively promote tumor progression through both the CXCL5-CXCR2 signaling and HIF1α-HMOX1-driven ferroptosis pathways. These insights underscore the importance of targeting osteocyte-mediated mechanisms in the development of therapeutic strategies to counteract melanoma bone metastasis and improve patient outcomes.

**P072**

**FGF-7 in Tumor-Induced Osteomalacia. Clinic-pathologic study of a patient with a sino-nasal Phosphaturic Mesenchymal Tumor**

*Luciano Colangelo[1], Francesca Romana Di Ciommo[1], Biagio Palmisano[2], Rossana Scrivo[1], Marco Della Monaca[3], Jessica Pepe[1], Cristiana Cipriani[1], Cristiano Alessandri[1], Valentino Valentini[3], Salvatore Minisola[1], Mara Riminucci[2], Fabrizio Conti[1], Alessandro Corsi[2]*
*[1]Sapienza University of Rome,*

*Department of Clinical- Internal- Anesthesiologic and Cardiovascular Sciences, Rome, Italy*
*[2]Sapienza University of Rome, Department of Molecular Medicine, Rome, Italy*
*[3]Sapienza University of Rome, Department of Odontostomatological Science and Maxillo-Facial Surgery, Rome, Italy*

**Abstract Text**

Tumor-induced osteomalacia (TIO) is an ultrarare paraneoplastic syndrome due to the overproduction of Fibroblast Growth Factor 23 (FGF-23). Although other phosphate-regulating substances (i.e., "phosphatonins") have been demonstrated to be expressed in tumors associated with TIO, very limited information is available about their circulating levels and clinical significance.

Here, we report a patient with a long-standing history of low serum phosphorus values due to a sino-nasal Phosphaturic Mesenchymal Tumor (PMT), in which co-expression of *FGF-23* and *FGF-7* was detected in the neoplastic tissue and their serum levels determined before and after surgical tumor excision. The serum level of FGF-23 was increased (312 pg/mL) before surgery and promptly normalized 24 hours after tumor excision. In contrast, the value of FGF-7 was high (202.6 pg/mL) before surgery, fell at 24 hours after surgery (108.6 pg/mL), increased again after one week (252.0 pg/mL) and finally normalized (33.40 pg/mL) one year later, six months after normalization of phosphoremia.

This is the first sino-nasal PMT in which expression of *FGF-7* was observed and the first case of TIO in which the serum levels of FGF-7 were monitored during the course of the disease (i.e., before and after tumor excision). The co-expression of "phosphatonins" in addition to FGF-23 might suggest a synergistic/additive phosphaturic effect. However, further studies are needed to define the mechanisms underlying the different post-surgical time-dependent reduction in the serum levels of FGF-23 and FGF-7 and the role of FGF-7 in patients with TIO, especially after surgery.

**P073**

**Therapeutic Potential of Caffeic Acid Phenethyl Ester (CAPE) in Inducing Apoptosis in Osteosarcoma Cells**

*Ana Lígia Pagnan[1], Adriano Pessoa[1], Cintia Tokuhara[2], Flávia Iano[3], Maylla Oliveira[1], Laura Ribeiro[1], Vanessa Fakhoury[1], Gabriela Oliveira[1], Mariana Sanches[4], Kelly Inacio[5], Valdecir Ximenes[6], Rodrigo Oliveira[1]*
*[1]Faculdade de Odontologia de Bauru - Universidade de São Paulo, Ciências Biológicas, Bauru, Brazil*
*[2]Sanford Children's Health Research Center, Human Genetics Program, San Diego, United States*
*[3]Faculdade de Odontologia de Araçatuba - UNESP, Preventive and Restorative Dentistry Division of Pediatric Dentistry, Araçatuba, Brazil*
*[4]Hospital for Rehabilitation of Craniofacial Anomalies, Pediatric, Bauru, Brazil*
*[5]Universite de Montreal, Institute for Research in Immunology and Cancer, Montreal, Canada*
*[6]Faculdade de Ciências - UNESP/Bauru, Química, Bauru, Brazil*

**Abstract Text**

Apoptosis is essential for eliminating non-essential, infected, or neoplastic cells, maintaining cellular cycle balance and homeostasis. Loss of this control can increase the resistance of neoplastic cells, induce angiogenesis, and elevate the capacity for cell invasion and metastasis. Osteosarcoma is a rare cancer characterized by malignant tumors in long bones, causing local pain and structural bone changes. Although osteosarcoma treatment has improved over the decades, resulting in increased patient survival, new therapeutic strategies are still needed. In this context, natural compounds have shown promising capabilities in modulating neoplastic processes, offering new therapeutic perspectives. One of the main active compounds in propolis, caffeic acid phenethyl ester (CAPE), has demonstrated antioxidant, anti-inflammatory, antibacterial, and anticarcinogenic properties in various studies. Studies with osteosarcoma cells (UMR-106) revealed that CAPE selectively targets tumor cells, reducing cell viability, decreasing migratory activity, and attenuating reactive oxygen species production. These effects may be related to CAPE's ability to induce apoptosis and cause acute mitochondrial damage. The activation of the intrinsic apoptotic pathway by CAPE occurs through the activation of effector proteins such as BAX and BAK, which increase mitochondrial permeability, resulting in the release of pro-apoptotic substances into the cytoplasm. Pharmacological modulation of BAX has been widely studied, especially regarding the ability of tumor cells to evade apoptosis through the overexpression of anti-apoptotic inhibitors. To investigate CAPE's influence on apoptotic pathways, a real-time quantitative PCR assay was conducted to verify the expression of targets such as caspases 3, 6, 9, BAX, and BAK. The results showed significance ($p<0.05$) for both concentrations ($IC_{25}$ and $IC_{50}$) in BAX expression after CAPE treatment in UMR-106 cells. The increased expression of BAK after CAPE treatment indicates the compound's ability to induce apoptosis in tumor cells, suggesting its potential as an adjuvant in osteosarcoma treatment.

**P074**

**Time-Lapsed In Vivo Micro-Computed Tomography Analysis of the Tibial Growth Plate in a Mouse Model of Multiple Myeloma**

*Sara Lindenmann[1], Neashan Mathavan[1], Friederike A. Schulte[1], Hayley M. Sabol[2], Jesus Delgado-Calle[2], Ralph Müller[1]*
*[1]ETH Zurich, Institute for Biomechanics, Zurich, Switzerland*
*[2]University of Arkansas for Medical Sciences, Department of Physiology and Cell Biology, Little Rock- AR, United States*

**Abstract Text**

**Introduction:** The study of multiple myeloma (MM) using micro-computed tomography (micro-CT) provides valuable insights into the destructive nature of the disease in bone. Previous studies have investigated the disease-defining osteolytic bone lesions or diffuse osteoporosis with compression fracture. When studying MM in young immature mice, changes at the growth plate should also be considered.

**Purpose:** This in vivo study investigated static and dynamic morphometry at the tibial growth plate to gain more insight into the remodeling activity during MM.

**Methods:** *In vivo* time-lapsed tibia images (voxel size: 10.5 µm) of male NSG mice of a control (Saline) and multiple myeloma vehicle (MMV) without and with sclerostin antibody (Scl-ab) treatment were investigated at week 1 and 3. Static and dynamic morphometric parameters like bone volume fraction (BV/TV), bone formation and resorption rate (BFR/BRR) were analyzed within two different metaphyseal masks, where one included the growth plate (GP) and the other one was set 525 µm below the GP to reflect secondary trabecular bone only.

**Results:** All groups showed higher BV/TV (> 10%) in the GP mask. In the GP mask, an increase of 12.13% in BV/TV was found when comparing week 1 with week 3 in MMV, while in the trabecular mask a decrease of BV/TV (-0.70%) was measured. The other two groups only showed moderate increases in BV/TV (Saline: 1.55%, Scl-Ab: 0.34%) in the GP mask. The increases or decreases of BV/TV were also reflected in higher, respectively lower BFR than BRR. All results are illustrated in Fig. 1.

**Conclusion:** Time-lapsed *in vivo* micro-CT enables quantitative and visual investigation of the involvement of the GP in the destructive process of MM and the favorable treatment of Scl-Ab. The data suggest a coincident destruction of metaphyseal trabeculae together with the formation of new bone in the growth plate region.

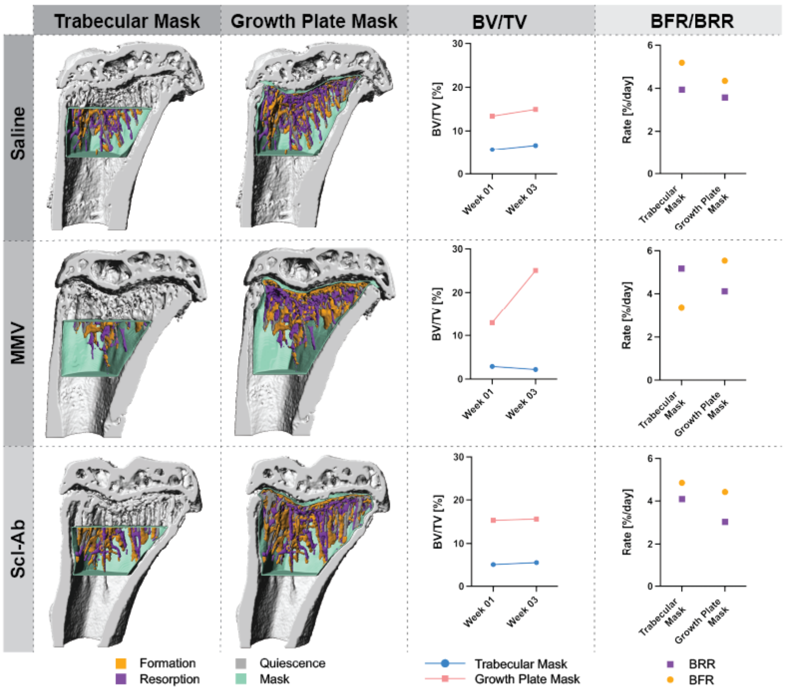

**Figure 1:** Visualisation of the successful gaussian filtering, registration and thresholding approach on *in vivo* time-lapsed micro-computed tomography (micro-CT) images to investigate static and dynamic morphometric parameters. Plots of the morphometric parameters are shown in the right two columns: bone volume fraction (BV/TV) and bone formation and resorption rates (BFR/BRR) for the two different masks applied to the three samples: Top row: Saline; Middle row: Multiple myeloma vehicle (MMV); Bottom row: Sclerostin antibody treatment (Scl-Ab).

**P075**

**Optical-Photothermal Infrared (O-PTIR) Microspectroscopy for Simultaneous Submicron Infrared and Raman Analysis of Calcified Materials**

*Miriam Unger[1], Carolin Borbeck[2]*

*[1]Photothermal Spectroscopy Corp GmbH, Applications, Muelheim an der Ruhr, Germany*

[2]Photothermal Spectroscopy Corp. GmbH, Applications, Muelheim an der Ruhr, Germany

**Abstract Text**

**Background:** Calcifications serve vital roles in both health and disease, acting as biomarkers for breast cancer and as structural components in bone and teeth. Traditional analytical methods, such as Fourier Transform Infrared (FTIR) and Raman spectroscopy, are often constrained by limitations in spatial resolution, sensitivity to water interference, or the need for complex sample preparation. O-PTIR spectroscopy overcomes these limitations by enabling label-free, contact-free chemical imaging with unprecedented submicron spatial resolution.

**Methods:** O-PTIR combines photothermal excitation using a tunable infrared laser and detection through visible light scattering, enabling infrared absorption spectra to be obtained with high spatial precision. Simultaneous Raman spectra are captured using the same optical path, allowing complementary molecular information to be acquired at the same submicron resolution without sample contact. Samples of breast cancer calcifications, bone, and teeth were analyzed to assess mineral content, matrix composition, and heterogeneity.

**Results:** The technique successfully mapped carbonate and phosphate content variations in breast cancer microcalcifications, revealing compositional heterogeneity correlated with pathological states. In bone and dental samples, O-PTIR provided detailed insight into mineral phase distribution, protein matrix organization, and hydration status. The simultaneous collection of Raman spectra enriched the analysis by highlighting molecular features not captured by infrared alone.

**Conclusion:** O-PTIR spectroscopy represents a paradigm shift in the molecular imaging of calcified materials. Its ability to perform non-contact, simultaneous submicron infrared and Raman analysis with high chemical specificity positions it as a powerful tool for advancing biomedical research, diagnostics, and biomaterials development.

**P076**

**PTH-related peptide (PTHrP)–mediated hypercalcemia in Malignancy: A multi-center case series based on Clinical Data Warehouse (CDW)**

*Kihyun Baek*[1], *Jinyoung Kim*[2]
[1]*Yeouido St. Mary's Hospital- College of Medicine- The Catholic University of Korea, Division of Endocrinology and Metabolism- Department of Internal Medicine, Seoul, Korea- South*
[2]*Yeouido St. Mary's Hospital- College of Medicine- The Catholic University of Korea, Department of Internal Medicine- Division of Endocrinology, Seoul, Korea- South*

**Abstract Text**

**Background** PTHrP is one of the important mediators in malignancy-associated hypercalcemia, but reports on the underlying causes are limited.

**Methods** This study is a retrospective case series based on a data platform for research purposes created based on electronic medical records from eight hospitals affiliated the Catholic University of

Korea. Through screening of laboratory results conducted from 2013 to 2022, cases in which hypercalcemia and PTHrP were simultaneously elevated among patients over 20 years of age were reviewed. Patients in whom malignancy was not confirmed were excluded.

**Results** Of the 292 patients studied, 85% had solid cancer and 15% had hematologic cancer. The most common cancer types were lung cancer 30% (n=87), head and neck cancer 10% (n=29), multiple myeloma 8% (n=24), esophageal cancer 7% (n=21), and hepatocellular carcinoma 6% (n=17). Among various cancer types, clear cell sarcoma, aplastic anemia, and hemophagocytic lymphohistiocytosis were carcinomas that had not been identified in previous reports. The median serum calcium level (corrected albumin) was 12.5 mg/dL [interquartile range, 11.7-13.8 mg/dL], and the median PTHrP level was 6.05 pmol/L [3.58-11.43 pmol/L]. The median age of this cohort was 66 years [59-75years], and the survival time after confirmation of hypercalcemia was 46 days [36-61 days].

**Conclusion** PTH-related peptide (PTHrP)–mediated hypercalcemia can occur with the progression of various type of cancers, and is associated with poor prognosis.

**P077**

**Assessment of bone loss and bone remodeling in premenopausal women with breast cancer during treatment with GnRH agonists and aromatase inhibitors: Cohort B-ABLE**

*<u>Natalia Garcia-Giralt</u>[1], Diana Ovejero[2], Sonia Servitja[3], Tamara Martos[4], Anna Ribes[5], Irene Petit[6], Xavier Nogues[7]*
[1]*CIBER on Frailty and Healthy Ageing, Hospital del Mar Research Institute, Barcelona, Spain*
[2]*Hospital del Mar Research Institute, Musculoskeletal research grup, Barcelona, Spain*
[3]*Hospital del Mar Research Institute- CIBERONC, Cancer Research Program, Barcelona, Spain*
[4]*Hospital del Mar Research Institute, Cancer Research Program, Barcelona, Spain*
[5]*Hospital del Mar Research Institute, Musculoskeletal research group, Barcelona, Spain*
[6]*Hospital del Mar, Internal Medicine Department, Barcelona, Spain*
[7]*Hospital del Mar- CIBERFES, Internal Medicine Department, Barcelona, Spain*

**Abstract Text**

**Introduction:** Ovarian function suppression (OS) combined with aromatase inhibitor (AI) therapy is the treatment of choice for premenopausal women with estrogen receptor-positive breast cancer. While effective in improving disease-free survival, this regimen is associated with estrogen deprivation-related adverse effects such as osteoporosis and arthralgia, impacting quality of life.

**Purpose:** To evaluate changes in bone mineral density (BMD), bone turnover markers and pain due to arthralgia during OS+AI therapy.

**Methods:** Prospective data were collected from women receiving OS+AI, in addition to vitamin D supplements, without osteoporosis. The primary outcomes were the relative annual changes in BMD measured by DXA in lumbar spine (LS), femoral neck (FN), and total hip (TH) and changes in bone turnover markers compared to baseline for up to 2 years of therapy. Arthralgia assessed via a visual analogue scale (VAS) was also registered. Statistical analysis was performed using mixed models for repeated measures in SPSSv3. Study was approved by the local Ethical Committee.

**Results:** Thirty-nine patients initiated OS+AI therapy (mean age: 46.67±4.5 years). Four patients who had not completed a year of therapy and one receiving alendronate were excluded from the analyses related to bone parameters. At 2 years, average bone loss was (mean[95%CI]) -9.52%[-10.71;-8.32] in LS, -8.34%[-9.92;-6.77] in FN, and -6.3%[-7.62;-4.94] in TH (p<0.001). Significant increases in P1NP, CTX and bone specific Alkaline Phosphatase were observed, especially during the first year of therapy (+33.46%[21.12;45.8], +0.21%[0.12;0.31], and +7.48%[5.54;9.41], respectively), which tended to normalize slightly during the second year, although still remained significantly elevated compared to baseline. Similarly, arthralgia measured by VAS increased almost 2 points during the first year and 0.85 the second year (p<0.001).

**Conclusions:** OS+AI therapy over two years leads to significant bone loss with increased bone turnover, and arthralgia development. These adverse effects warrant monitoring and interventions to mitigate long-term risks.

**P078**

**Dickkopf 1 (DKK1) mediates stress-induced cytokine responses in prostate and breast cancer**

*Sophie Pählig*[1], *Andy Göbel*[1], *Tilman D. Rachner*[1]
[1]*Hospital of the Dresden University of Technology, Department of Medicine III, Dresden, Germany*

**Abstract Text**

Dickkopf-1 (DKK1) is an inhibitor of canonical Wnt signaling. In homeostasis, DKK1 acts as a regulator of bone metabolism and embryonal development. Moreover, DKK1-overproduction is a hallmark of numerous pathologies including cancer. Tumor cells encounter with different types of stressors, including proteostatic stress, hypoxia, nutrient deprivation, or cancer treatments. A typical reaction is the inflammatory response. We hypothesize that DKK1 acts as a mediator of the inflammatory response to stress and that high DKK1 expression is an adaptive mechanism of intrinsically stressed cancer cells. The increased inflammatory response could further promote tumor growth and metastasis.

Human prostate cancer cells (PC3) and breast cancer cells (MDA-MB-231) were treated with different stressors (e.g. LPS and Thapsigargin) and MAPK inhibitors. Gene silencing was performed using siRNA and stable shRNA clones. Gene expression was assessed by real-time PCR. Protein expression was analyzed by immunoblotting.

We confirmed a strong *DKK1* mRNA and protein expression in all cell lines. Moreover, cytokine mRNA expression of *IL1b, IL6* and *IL8* significantly correlates with *DKK1* expression in all tested cell lines.

Treatment of cells with different stressors fostered *IL1b*, *IL6* and *IL8* expression (up to 15-fold), while knockdown of *DKK1* prevented the pro-inflammatory response, pointing to a mechanistic link between *DKK1* and cytokine expression. Combined treatment of PC3 cells with different MAPK inhibitors showed, that the cells required p38 signaling to mount DKK1 response upon stress.

Injection of DKK1-deficient and corresponding control PC3 cells in a zebrafish xenograft model uncovered the role of DKK1 for early metastasis, since the former ones showed 40% less tumor burden.

Our data unravel DKK1 as a regulator of stress induced cytokine response, playing a role in early metastasis. Additional experiments are needed to dissect the role of DKK1 in tumor cell biology and as a potential target in cancer treatment.

**P079**

**Contribution of mesenchymal stromal cells to the metastatic bone niche of breast cancer patients: involvement of alkaline phosphatase**

*Drenka Trivanovic*[1], *Nikola Bogosavljević*[2,3], *Milica Rajković*[1], *Marko Vujačić*[2,3], *Nemanja Jovanović*[2,3], *Tamara Kukolj*[1], *Marija Ćazić*[4], *Slavko Mojsilović*[1], *Aleksandra Jauković*[1]
[1]*Institute for Medical Research University of Belgrade, Group for Hematology and Stem Cells, Belgrade, Serbia*
[2]*Institute for Orthopedy Banjica, Surgery, Belgrade, Serbia*
[3]*Faculty of Medicine University of Belgrade, Orthopedic Surgery, Belgrade, Serbia*
[4]*University Children's Hospital, Department of Hematology and Oncology, Belgrade, Serbia*

**Abstract Text**

Breast cancer (BC) is one of the most frequent cancers in women and the skeleton is a common metastatic site for disseminated BC cells (BCCs). Ours as well as the results of others showed that the mesenchymal stromal cells (MSCs), acquire phenotype of cancer-associated cells, supporting BCC survival at the primary BC site. It is known that bone marrow MSCs participate in organization and evolution of pre-metastatic and metastatic niches for disseminated BCCs. Here we explored a mechanistic background of MSC engagement in BC bone metastatic niche.

Cells were isolated from femurs from metastatic BC patients (BC-MSCs, n=12) and women with hip arthrosis (non-BC-MSCs, n=12, control) undergoing arthroplasty (Ethical approvals: I-97/14.3 and I-97/24.2). To recapitulate BCC invasion into marrow, MCF-7 cells were used, and cellular assays were conducted in hypoxia (3% $O_2$). Cell phenotype was determined by flow cytometry, immunofluorescent microscopy and qPCR. Statistical significance was estimated by non-parametric tests.

Our results indicated no significant difference in proliferation rates nor Ki67 expression between non-BC-MCS and BC-MCSs. BC-MSCs showed significantly higher alkaline phosphatase (ALP) activity than non-BC-MSCs (n=7, p=0.031) and assessment of osteogenic potential showed preserved calcification levels in BC-MSC. Although we observed lower capacity for lipid droplet enlargement in BC-MSCs than in non-BC-MSCs, BC-MSCs were not completely deprived of adipogenic potential. When compared to BC-MSCs, non-BC-MSCs significantly increased adherence of MCF-7 cells. In addition, conditioned media from both cell populations upregulated bulk and single-cell colony forming capacity and expression of SSEA4, OCT4, Nanog and SOX2 in MCF-7 cells. The presence of ALP activity inhibitor abolished these effects.

These results revealed that distinct states of MSCs can support different aspects of BCC metastatic behavior. Further investigation will define the molecular signature of pre-metastatic and metastatic BC-educated MSCs to empower the knowledge on BC and management of skeletal pathologies in metastatic BC.

**P080**

**RANKL promotes melanoma-induced metastasis in the TgRANKL mouse model**

*Christos Chrysovalantis Syrigos[1,2], Evi Gkikopoulou[1,2], Martina Rauner[3], Eleni Douni[1,2]*
*[1]Agricultural University of Athens, Department of Biotechnology, Athens, Greece*
*[2]B.S.R.C. "Alexander Fleming", Institute for Bioinnovation, Vari, Greece*
*[3]TU Dresden,*
*Division of Endocrinology- Diabetes and Bone Diseases- Department of Medicine III- and Center for He althy Aging- University Medical Center, Dresden, Germany*

**Abstract Text**

**Background:** Melanoma, the most aggressive form of skin cancer, progresses rapidly and exhibits a high propensity for metastasis to various tissues, including bones. Receptor activator of nuclear factor-κB ligand (RANKL) plays a central role in osteoclastogenesis and bone resorption. Additionally, RANKL has been linked to the capacity of cancer cells to migrate and expand within the bone microenvironment. However, the potential involvement of RANKL in melanoma metastasis remains incompletely understood.

**Purpose:** We explored the metastatic potential of melanoma cells in osteoporotic TgRANKL mice overexpressing the human RANKL gene and evaluated the impact of denosumab, a monoclonal antibody targeting human RANKL, in mitigating metastases.

**Methods:** We systemically injected a range of B16F10 mouse melanoma cells into the caudal tail artery of syngeneic TgRANKL and wild-type (WT) mice of both sexes (ethical-approved). Over a period of three weeks, we monitored body weight and grip strength. Macroscopic examination during necropsy revealed melanoma metastases in various tissues, which were further confirmed by histological analysis.

**Results:** Our findings demonstrated a greater metastatic burden of melanoma cells in multiple tissues of TgRANKL mice compared to WT mice, including the hindlimbs, spinal cord, intestine, lungs, calvarial bones, and brain. Specifically, bone metastases were associated with increased bone loss and metastasis progression in both male and female TgRANKL mice relative to their WT littermates. Prophylactic administration of denosumab improved the overall health status of TgRANKL mice and significantly reduced the incidence of metastases in various tissues, including bone.

**Conclusions:** Collectively, our results demonstrate that RANKL overexpression in TgRANKL mice enhanced the metastatic potential of melanoma cells, particularly in the bone microenvironment, while prophylactic administration of denosumab demonstrated promising therapeutic effects, reducing metastasis in both bone and other tissues.

*The research work was supported by the Hellenic Foundation for Research and Innovation (HFRI) under the 5th Call for HFRI PhD Fellowships (Fellowship Number: 19290).*

**P081**

**Role of RANKL in Prostate Cancer-Induced Bone Metastases and the Therapeutic Potential of Denosumab in the TgRANKL mouse model**

*Christos Chrysovalantis Syrigos[1,2], Anna Peramatzi[1], Evi Gkikopoulou[1,2], Melina Dragolia[2], Vasileios Ntafis[2], Martina Rauner[3], Eleni Douni[1,2]*
*[1]Agricultural University of Athens, Department of Biotechnology, Athens, Greece*
*[2]B.S.R.C. "Alexander Fleming", Institute for Bioinnovation, Vari, Greece*
*[3]TU Dresden,*
*Division of Endocrinology- Diabetes and Bone Diseases- Department of Medicine III- and Center for Healthy Aging- University Medical Center, Dresden, Germany*

**Abstract Text**

**Background:** Bone metastases are highly prevalent in patients with advanced prostate cancer, with bones being the most frequent site of prostate-driven metastasis. Receptor activator of nuclear factor-κB ligand (RANKL) plays a pivotal role in prostate cancer metastasis to bone by promoting osteoclastogenesis. Denosumab, a monoclonal antibody that inhibits RANKL activity, has been employed as a therapeutic strategy to reduce skeletal-related events associated with bone metastases.

**Purpose:** In this study, we examined the metastatic potential of prostate cancer cells in the bones of osteoporotic TgRANKL male mice, which overexpress the human RANKL gene. Additionally, we investigated the impact of RANKL inhibition on bone metastases through denosumab administration.

**Methods:** To assess metastatic bone lesions, $10^5$-$2x10^5$ RM1-BM mouse prostate cancer cells, recognized for their bone-metastatic potential, were injected systemically into the caudal tail artery of syngeneic TgRANKL and wild-type (WT) male mice (ethical-approved). These cells were stably transduced with the firefly luciferase gene, allowing metastatic progression to be monitored over three weeks using *in vivo* bioluminescence imaging. Histological analysis was performed to evaluate metastatic bone lesions.

**Results:** *In vivo* bioluminescence imaging revealed enhanced bone metastasis progression in the hindlimbs of TgRANKL mice compared to their WT littermates, most of whom remained metastasis-free. These findings were confirmed through histological analysis. Subsequently, we investigated the potential of denosumab as a prophylactic treatment to prevent bone metastasis. Both imaging and histological analyses indicated that while denosumab did not entirely block bone metastasis, it effectively reduced its occurrence in the hindlimbs of TgRANKL mice and prevented osteolysis.

**Conclusions:** Our findings demonstrate that TgRANKL mice exhibit an increased susceptibility to prostate cancer-induced bone metastasis compared to their WT counterparts, while RANKL inhibition significantly reduced its incidence and mitigated osteolysis.

*The research work was supported by the Hellenic Foundation for Research and Innovation (HFRI) under the 5th Call for HFRI PhD Fellowships (Fellowship Number:19290).*

**P083**

**Bortezomib sensitizes breast cancer and bone metastasis cell lines to NK-mediated killing by restoring NKG2DLs expression on membrane surface**

*Marta Sofia Gomarasca[1], Paola Maroni[1], Chiara Verdelli[1], Barbara Bassani[2], Maria Teresa Palano[2], Laura Gerosa[1], Martina Faraldi[1], Antonino Bruno[2,3], Giovanni Lombardi[1,4]*
*[1]IRCCS Ospedale Galeazzi-Sant'Ambrogio,*
*Laboratory of Experimental Biochemistry and Advanced Diagnostic, Milan, Italy*
*[2]IRCCS MultiMedica, Laboratory of Innate Immunity, Milan, Italy*
*[3]University of Insubria, Department of Biotechnology and Life Sciences, Varese, Italy*
*[4]Poznań University of Physical Education, Department of Athletics- Strength and Conditioning, Poznań, Poland*

**Abstract Text**

Bone metastases dramatically worsen breast cancer (BC) prognosis and reduce overall survival. Natural killer (NK) cells can detect and eliminate malignant cells through the NKG2D receptor-mediated recognition of the ligands (NKG2DLs) expressed by cancer cells. The proteasome inhibitor bortezomib has demonstrated to restore cell surface expression of the NKG2DLs in different cancers; however, little is known of its effect on BC cells or on bone metastasis (BCbm) cells. Here, we demonstrate that bortezomib increases the expression of the NKG2DLs MICA/B and ULBP2 on BC and BCbm cell surface to restore NK-mediated immune response.

We investigated expression and cellular localization of MICA/B and ULBP2 in tissue samples of different tumor stages and, *in vitro*, in BC cells with different invasive potential (MDA-231, MCF7) and BCbm cells (1833) by immunohistochemistry, immunofluorescence, WB, and flow cytometry. Cells were treated with 10nM bortezomib for 24h or left untreated as control.

The different intracellular distribution of MICA/B and ULBP2 in tissue sections reflected a marked co-localization with Golgi in invasive and migrating MDA-231 and 1833 cells, and, at a lesser extent, in less invasive MCF7 cells. Upon treatment with bortezomib the plasma membrane localization of MICA/B was increased in 1833 and MDA-231 (1.7- and 1.8-fold, respectively, $p<0.05$), while ULBP2 was increased in MCF7 (2.2 fold, $p<0.01$). Additionally, bortezomib increases NK-mediated killing with higher sensitivity for more invasive 1833 and MDA-231 cells.

Invasive and migrating BC cells retain NKG2DLs into Golgi avoiding immune recognition. Upon bortezomib treatment, cell surface expression of MICA/B and ULBP2 is restored, enhancing cell sensitization to NK-mediated recognition, especially in the most invasive cells. These results suggest a relevant mechanism of action to improve immune surveillance especially in more aggressive BC and in BCbm, to be exploited as a combined treatment to enhance anti-tumor response.

**P084**

**Deciphering the role of miR-26a-5p, miR-27a-3p, and miR-30e-5p on prostate cancer homing to bone in human disease**

*Ulrike Németh[1], Kati Erdmann[2], Susanne Füssel[2], Daniela Aust[3], Martina Rauner[4], Lorenz C. Hofbauer[5], Stefanie Conrad[4]*
*[1]Technische Universität Dresden, Department of Medicine III- Institute of Pathology, Dresden, Germany*
*[2]Technische Universität Dresden, Department of Urology, Dresden, Germany*
*[3]Technische Universität Dresden,*
*Institute of Pathology- Tumour- and Normal Tissue Bank of the University Cancer Center UCC, Dresden, Germany*
*[4]Technische Universität Dresden, Department of Medicine III, Dresden, Germany*
*[5]Technische Universität Dresden, Department of Medicine III- Center for Healthy Aging, Dresden, Germany*

**Abstract Text**

Prostate cancer (PCa) frequently leads to bone metastasis. Recent studies suggest a significant role of microRNAs (miRNAs) to control the formation of bone metastases in different cancer types. However, how miRNAs mediate the interactions between PCa cells and the bone microenvironment remains unclear. Here, we assessed the expression levels of miR-26a-5p, miR-27a-3p, and miR-30e-5p in human serum and urine samples to further assess their potential diagnostic value.

Human serum samples from patients with benign prostatic hyperplasia (BPH, n=14), primary PCa (n=14) and PCa-induced bone metastases (n=14) collected at the Departments of Urology and Medicine III at the Technische Universität Dresden were analyzed for miR-26a-5p, miR-27a-3p, and miR-30e-5p expression. The GEO2R (Gene Expression Omnibus) database was used to analyze the series GSE86474 on the "Urine miRNA transcriptome of prostate cancer patients" for the miRNAs of interest in urine samples from PCa patients. The cohort consists of nine control, 49 low-risk, and 50 high-risk PCa patients.

Analysis of miR-26a-5p, miR-27a-3p, and miR-30e-5p in human serum samples showed the highest expression levels in primary PCa patients and lower levels in the serum of patients with BPH and PCa metastasis [$P<0.05$, $P<0.01$, $P<0.001$]. Furthermore, miR-26a-5p expression levels were also significantly lower in serum of patients with metastasis compared to BPH [$P<0.05$]. Expression analysis of miRNAs in urinary cell sediments using GEO2R study showed no difference for miR-26a-5p between these groups. However, miR-27a-3p was significantly lower in low- and high-risk PCa samples compared to controls [$P<0.001$]. In contrast, miR-30e-5p showed a significantly higher level in the urinary cell sediments of low-risk PCa patients [$P<0.05$] compared to the control. Our results show distinct miRNA patterns in the serum and urine samples of PCa patients. However, matching sample pairs, which are currently collected, will enable more accurate comparisons between the groups in a larger cohort.

**P085**

**A case report of heterotopic ossification with somatic GNAS mutation as a cause of dramatic misdiagnosis: progressive ossificans heteroplasia versus malignancy**

*Irina Nikishina*[1], *Svetlana Arsenyeva*[1], *Valeria Matkava*[1], *Alesya Firsa*[1], *Garik Sagoyan*[2]
[1]*V.A. Nasonova Scientific Research Institute of Rheumatology, Paediatric, Moscow, Russia C.I.S.*
[2]*N.N. Blokhin National Medical Research Centre of Oncology, Pediatric oncology, Moscow, Russia C.I.S.*

**Abstract Text**

**Introduction**: Diseases associated with GNAS mutation have variety clinical manifestations. One of them is ultra-rare progressive ossificans heteroplasia (POH) presented with formation of heterotrophic ossification (HO) in soft tissues. Sometimes POH may be misdiagnosis of other diseases such as trauma,paralysis,cancer.

**Purpose**: to describe a clinical case of misdiagnosed malignant peripheral nerve sheath tumor(MPNST).

**Methods:** Boy 13 y.o. suffered from the tingling and pain in the left foot and tibia for a year. Painful formation appeared later on the plantar of the left foot and caused gait disturbance. MRI/ultrasound detected neoplasm lasted from plantar to tibia.Diagnosis of calcifying aponeurotic fibroma was proposed after first biopsy. X-Ray and CT found multiple calcinosis and ossificates.Repeated biopsy and histological examination revealed only bone tissue formation. Presence of left extremity asymmetrical reticular HO reminded hereditary condition: POH was suggested. Molecular genetic testing of histological sample verified somatic mutation of GNAS gene and patient was directed to geneticist. Genetic analyses in a patient and his parents didn`t confirm the GNAS mutation.Because of these manipulations patient lost about two years.

**Results**. Finally, patient was consulted by rheumatologists.We identified huge neoplasm (8x5 cm) on the left foot with local reaction and vascularization. At first sight we realized that these clinical features didn’t correspond to POH despite the verified somatic GNAS mutation.Patient was consulted by oncologist again, lung CT detected multiple metastatic lesions. Morphological examination verified MPNST G3.Lower-leg amputation was done, many chemotherapy protocols were performed without positive results.Inspite of all efforts the child died.

**Conclusion**: HO is a new challenge for oncologists,geneticists,rheumatologists. It's important to establish the type of HO for correct management strategy.Delay of correct diagnosis and treatment lead to dramatic final.Paradoxically, it can be assumed that it'd been better for this patient if at the beginning he hadn't met doctor who was informed about such a rare disease as POH.

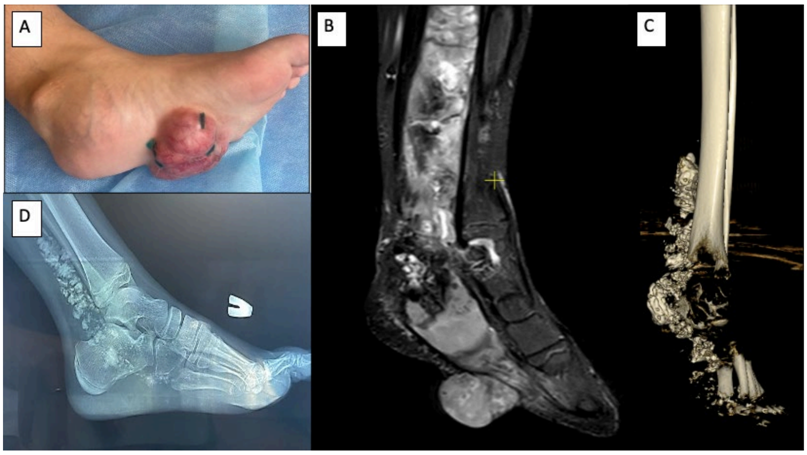

**P087**

**Tenosynovial Giant Cell Tumour-derived fibroblasts retain the imprint of increased capacity of forming large multinucleated giant cells.**

*Teun De Vries*[1], *Julia Ide*[1], *Willem H. Schreuder*[2], *Daisy Picavet*[3], *Marjolijn Gilijamse*[2], *Jolanda Hogervorst*[4], *Ton Schoenmaker*[1]

[1]*Academic Centre for Dentistry Amsterdam- University of Amsterdam and Vrije Universiteit Amsterdam, Periodontology, Amsterdam, Netherlands*

[2]*Amsterdam University Medical Centre, Oral and Maxillofacial Surgery and Oral Pathology, Amsterdam, Netherlands*

[3]*Amsterdam University Medical Centre, Medical Biology, Amsterdam, Netherlands*

[4]*Academic Centre for Dentistry Amsterdam- University of Amsterdam and Vrije Universiteit Amsterdam, Oral Cell Biology, Amsterdam, Netherlands*

**Abstract Text**

**Background/Introduction:** Tenosynovial Giant Cell Tumours (TGCT) are rare lesions of the joints with both inflammatory and neoplastic features, and with a high heterogenic profile. Clinically, these tumours manifest themselves with resorption of bone in situ, being one of the key pathological features of TGCT.However, the tumour's aberrant osteoclastogenesis leading to the formation of highly multinucleated giant cells (GCs) remains unclear.

**Purpose:** This study examines osteoclast differentiation steered by fibroblast-like cells derived from a rare case of bone-destructive TGCT with excessive bone resorption at the base of the skull. We hypothesize that TGCT-derived fibroblasts maintain an imprint for accelerated osteoclast formation.

Methods.
To test this, TGCT-derived fibroblasts or control alveolar bone (AB)-derived cells were co-cultured with PBMCs in osteoclastogenesis assays. Formation of osteoclast-like cells was higher in TGCT-cocultures, where the osteoclast-like cells were highly multinucleated with 15x more prevailant with >10 nuclei ($p<0.01$) in TGCT. TGCT secreted more M-CSF before ($p<0.001$) and during co-culture ($p<0.05$). The levels of expression of osteoclastogenic markers M-CSF (3x, $p<0.01$) and RANKL (3x , $p<0.01$) and osteoclast fusion protein DC-STAMP (3x , $p<0.01$)were higher in TGCT-PBMC co-cultures. The osteogenic potential of the tumour-derived cells was assessed through ALP activity and Alizarin Red staining and showed similar differentiation potential as controls. Our main findings indicate increased osteoclastogenic activity in the fibroblasts derived from the TGCT. These results contribute to the understanding of TGCT pathology and support the potential use of M-CSF inhibitors in treating these tumours.

**Poster Sessions: Osteoblasts and bone formation**

**P087a**

**Stanniocalcin 1 and 1,25(OH)2D3 cooperatively regulate osteoblast-dependent bone mineralization**

*Jungha Kim[1], Kabsun Kim[1], Wonbong Lim[1,2]*
*[1]School of Medicine- Chosun University, Lab. of Orthopaedic Surgery, GwangJu, Korea- South*
*[2]Chonnam National University, Regional Leading Research Center, Yeosu, Korea- South*

**Abstract Text**

Stanniocalcin 1 (STC1) is a calcium- and phosphate-regulating hormone that is expressed in all tissues, including bone tissues, and is involved in calcium and phosphate homeostasis. Previously, STC1 expression was found to be increased by 1,25-dihydroxyvitamin $D_3$ [1,25$(OH)_2$Vitamin-$D_3$] administration in renal proximal tubular cells. In this study, we determined whether STC1 directly regulates osteoblast differentiation or reciprocally controls the effects of 1,25$(OH)_2D_3$ on osteoblasts to contribute to bone homeostasis. We found that STC1 inhibited osteoblast differentiation *in vitro* and bone morphogenetic protein 2 (BMP2)-induced ectopic bone formation *in vivo*. Moreover, 1,25$(OH)_2D_3$ increased STC1 expression through direct binding to the *Stc1* promoter of vitamin D receptor (VDR). STC1 activated the 1,25$(OH)_2D_3$–VDR signaling pathway through the upregulation of VDR expression mediated by the inhibition of Akt phosphorylation in osteoblasts. STC1 further enhanced the roles of 1,25$(OH)_2D_3$ in the secretion of receptor activator of nuclear factor-κB ligand (RANKL) and inhibition of osteoblast differentiation by exhibiting a positive correlation with 1,25$(OH)_2D_3$. The long bone phenotype of transgenic mice overexpressing STC1 specifically in osteoblasts was not significantly different from that of wild-type mice. However, compared with wild-type mice, 1,25$(OH)_2D_3$ administration significantly decreased bone mass in STC1 transgenic mice. Collectively, these results suggest that STC1 negatively regulates osteoblast differentiation and bone formation; however, the inhibitory effect of STC1 on osteoblasts is transient and could be compensated under normal conditions. Nevertheless, the synergistic effect of STC1 and 1,25$(OH)_2D_3$ through 1,25$(OH)_2D_3$ administration may reduce bone mass by inhibiting osteoblast differentiation.

**P088**

**Extracellular Phosphate Sensing by Osteoblasts: Regulation of Matrix Mineralization and Phosphatase Expression through FGFR1 and ERK1/2 Signaling Pathways**

_Soher Jayash_[1], *Thomas Duff*[1], *Qaisar Tanveer*[1], *Colin Farquharson*[1]
[1]*University of Edinburgh, The Roslin Institute, Edinburgh, United Kingdom*

**Abstract Text**

**Background/Introduction:** Systemic and local mechanisms are in place to tightly regulate phosphate (Pi) provision for skeletal biomineralization. The contribution of extracellular Pi versus locally generated Pi to the mineralization process remains obscure and osteoblasts may sense extracellular Pi to modulate phosphatase activity and fine-tune the biomineralization process

**Purpose:** This study examined the ability of osteoblasts to sense and respond to extracellular Pi to regulate osteoblast expression of tissue-nonspecific alkaline phosphatase (TNAP) and PHOSPHO1.

**Methods:** Human primary osteoblasts were cultured with varying Pi concentrations (0, 1 mM, 3 mM, and 5 mM). Matrix mineralization was assessed using alizarin red staining, while the expression of PHOSPHO1, ALPL, SLC20A1/PiT1, and SLC20A2/PiT2 was evaluated via RT-qPCR and immunoblotting. Intracellular signaling pathways were analyzed using immunoblotting with antibodies targeting total and phosphorylated ERK1/2, AKT, p38, and JNK1/2.

**Results:** Extracellular Pi downregulated the expression of PHOSPHO1 and TNAP at both mRNA and protein levels. Increasing Pi concentrations also reduced the mRNA expression of the type III Na-Pi co-transporters, PiT-1 and PiT-2 and selectively enhanced ERK1/2 phosphorylation, with no significant effects on AKT, JNK1/2, or p38 signaling pathways. Inhibition of PiT-1 and PiT-2 by Foscarnet or MEK1/2 by UO126 abolished the downregulation of PHOSPHO1 and ALPL expression by extracellular Pi. Moreover, extracellular Pi phosphorylated FRS2α (downstream of FGFR signalling) and this activation was abolished by Foscarnet. Also, blocking FGFR signaling by PD173074 inhibited the phosphorylation of ERK1/2 and prevented the decrease in ALPL and PHOSPHO1 expression by extracellular Pi. Finally, the promotion of osteoblast matrix mineralization by extracellular Pi was dependent upon type III Na-Pi co-transporters and FGFR signalling.

**Conclusion(s):** The interplay between FGFR1 and Pi transporters suggests a nuanced mechanism by which osteoblasts sense and respond to extracellular Pi. This understanding advances our knowledge of the molecular control of physiological bone mineralization by osteoblasts.

**P089**

**Expression of Wnt1 induces immediate bone formation with site-specific patterns in mice**

_Kim Wagner_[1], *Wenbo Zhao*[1], *Julia Luther*[1], *Michael Amling*[1], *Thorsten Schinke*[1], *Timur Alexander Yorgan*[1]
[1]*University Medical Center Hamburg-Eppendorf, Department of Osteology and Biomechanics,*

*Hamburg, Germany*

**Abstract Text**

**Introduction:** The ligand Wnt1 is a highly relevant regulator of bone mass. Pathogenic variants of WNT1 can cause early-onset osteoporosis and osteogenesis imperfecta type XV in humans. Importantly, induction of Wnt1 via a transgenic system in mice has been demonstrated to cause a rapid and substantial increase in bone mass, highlighting the enormous osteoanabolic potential of Wnt1. However, the precise nature of this osteoanabolic response has not been thoroughly investigated.

**Purpose:** The purpose of this study was to analyze the early tissue-level osteoanabolic response of bones upon Wnt1-induction.

**Methods:** STOCK-Tg(tetO-*Wnt1*,-luc)TWNTLach-Tg(Col1a1-tTa)139Niss/Uke mice were used to induce osteoblast-specific Wnt1-expression by doxycycline removal for 3, 5 and 7 days prior to sacrifice at the age of 6 weeks. Vertebral bodies and tibiae were collected for histological analysis. Ethical approvals were present.

**Results:** After 3 days of Wnt1-induction, a significant increase of osteoblasts was observed in vertebral bodies [86.62 vs. 51.94 $mm^{-1}$, p<0.0001], followed by a continuous increase of bone volume after 5 and 7 days of induction [d5: 29.89, d7: 32.82 vs. d0: 21.61 %, p<0.01]. Likewise, an increase of bone mass was observed in tibiae after 7 days of induction. Here, a striking aggregation of cells interspersed with partially mineralized extracellular matrix was observed even after 3 days of Wnt1-induction in the proximal metaphyseal medullary cavity. Although initially reminiscent of sclerosis, these aggregates were locations of apparent de novo bone formation, as they increased in size and developed mineralized trabecular structures after longer Wnt1-induction.

**Conclusion:** Induction of Wnt1 signaling causes an immediate tissue level response with increased osteoblast numbers and bone formation. The specific nature of this response was found to be divergent at different skeletal sites, as in vertebral bodies mostly typical bone formation was observed, whereas there were regions of apparent de novo bone formation in tibiae.

**P091**

**The interplay between GPR39 and TGF-β signaling in the Regulation of collagen transcription in osteoblasts**

<u>*Gal Gozlan*</u>*[1], Biplab Chatterjee[2], Chen Dahan Abramovitz[3], Noam Levaot[4]*
*[1]Ben Gurion University, Biology and physiology of the cell, Beer Sheva, Israel*
*[2]Ben Gurion, biology and physiology of the cell, Atlanta, United States*
*[3]Ben Gurion University, biology and physiology of the cell, Meitar, Israel*
*[4]Ben Gurion University, Biology and physiology of the cell, lehavim, Israel*

**Abstract Text**

Bone quality and structural integrity rely on a delicate balance between bone formation and resorption, processes orchestrated by a complex network of signaling pathways. Among these, GPR39, a zinc-sensing G-protein-coupled receptor, has emerged as a modulator of bone homeostasis, influencing osteoblast differentiation, extracellular matrix (ECM) composition, and mineralization. Here we show that GPR39 control collagen synthesis through regulation of collagen I alpha 2 (col1a2) transcription. We show that acute stimulation of osteoblasts with zinc activates col1a2 transcription in a GPR39 dependent manner. Furthermore, GPR39 is known to activate $G_q\alpha$ signaling downstream of zinc exposure. We show that col1a2 induction by zinc is blocked by a specific $G_q$ inhibitor. TGF-β signaling is a known regulator of bone ECM composition and regulates col1a2 transcription in osteoblasts. We show that TGF-β induction of col1a2 transcription in osteoblasts depends on GPR39. Moreover, TGF-β fails to induce col1a2 transcription in the presence of a specific $G_q$ inhibitor. Taken together our data suggests a crosstalk between GPR39 and TGF-β in regulation of collagen transcription. These findings have broad implications for the regulation of bone quality in health and disease.

**P092**

**B cells regulate bone remodeling in a Fra-2/AP-1 dependent manner**

*Flora Nguemedjio Foko Takile[1], Sylvia Thiele[2], Martina Rauner[2], Georg Schett[1], Aline Bozec[1], Bettina Groetsch[1]*
*[1]Universitätsklinikum Erlangen, Department of Medicine 3 - Rheumatology and Immunology, Erlangen, Germany*
*[2]Uniklinikum Dresden, Medizinische Klinik III - Bone Lab, Dresden, Germany*

**Abstract Text**

Bone and immune cell interactions and their regulatory mechanisms are central topics of osteoimmunology. However, the impact of immune cells especially B cells in bone remodeling remains poorly described. Remarkably, the AP-1 transcription factor Fra2 is an important regulator of skeletogenesis and early B cell development. Nevertheless, it is still unknown if B cell-specific expression of Fra2 can control bone turnover. Notably, ChIP sequencing in WT B220+ bone marrow B cells identified binding sites for Fra2 on the Dkk1 promoter.
We hypothesize that Fra2 deletion in B cells, downregulates Dkk1 secretion, a soluble inhibitor of Wnt/β-catenin signaling, leading to increased ß-catenin signaling in osteoprogenitor cells that promote osteoblast proliferation and differentiation.
The bone homeostatic state of mice with B cell-specific (mb1cre) deletion of Fra2 (*Fra2ΔBcell*) was first analyzed compared to littermate controls. *Fra2ΔBcell* mice showed an increased bone volume (BV/TV: 0.064 vs. 0.101%; p=0.035) due to increased osteoblast numbers (N. OB/B.Pm: 2.758 vs. 7.724 1/mm; p=0.013). In vitro, *Fra2ΔBcell* B220+ bone marrow B cell conditioned media significantly increased MSC proliferation and differentiation into osteoblasts compared to WT conditioned medium (0.234% vs. 0.275%; p=0.018). Further analyses revealed a substantial reduction in Dkk1 within the *Fra2ΔBcell* conditioned medium compared to wt control (10200 vs. 3506-pixel intensity; p=0.02).
Therefore, we generated mice with B cell-specific (mb1cre) deletion of Dkk1 (*Dkk1ΔBcell*). In line with our previous *Fra2ΔBcell* data, *Dkk1ΔBcell* mice showed an increased BV/TV (0.22 vs. 0.268%; p=0.07) due to increased N.Ob/B.Pm (0.749 vs. 2.543 1/mm; p=0.001) when compared to controls. Moreover, *Dkk1ΔBcell* conditioned medium significantly increased MSC proliferation and differentiation (953.76 vs. 1262.3 cells/cm²; p=0.026).
Altogether, our data depict Fra2 in B cells as a central actor in Wnt/ß-catenin signaling to orchestrate osteoblast differentiation and thereby bone formation. Our goal is to understand the molecular mechanisms underlying the role of B cells in bone loss-related diseases.

**P093**

**Diet-Induced Bone Adiposity Implications: Beta-Oxidation Pathway in Adipogenesis-Osteoblastogenesis Imbalance**

*Ana Alonso-Pérez*[1], *María Guillán-Fresco*[1], *María Piñeiro-Ramil*[1], *Miriam López-Fagúndez*[1], *Andrés Pazos-Pérez*[1], *Verónica López*[1], *Claudio Pirozzi*[2], *Alberto A. Jorge-Mora*[1], *Rodolfo Gómez*[1]
[1]*Institute IDIS, Musculoskeletal Pathology Group, Santiago de Compostela, Spain*
[2]*University of Naples Federico II, Department of Pharmacy, Napoli, Italy*

**Abstract Text**

**Background:** Adipogenesis-osteoblastogenesis balance rupture is observed in multiple bone diseases. Here, signalling pathways webwork to integrate the information from the environment and stimuli. Transcription factors (TFx) play a key role in this process controlling the adipogenic-osteoblastogenic equilibrium. In previous works, we have identified a node of TFx (Node) involved in osteoblast-adipocyte balance control, related to ageing, obesity, and bone adiposity physiopathological situations. Notwithstanding, the pathways by which this Node exerts its effect are yet not known.

**Objective:** To identify the potential mechanisms by which the Node modulates the osteoblastogenesis-adipogenesis balance.

**Methods:** Bones from a mice model of Diet-Induced Obesity (DIO) were characterised through image, RT-qPCR, and proteomic techniques. Following, the expression of the beta-oxidation pathway genes altered in DIO mice bones was measured by RT-qPCR in mice mesenchymal stem cells (C3H10T1/2) differentiated to osteoblast and adipocyte, where the Node was silenced by siRNA. The expression of the same genes was equally measured in human bone samples and correlated with the Node expression and the Body Mass Index (BMI). Ethical requirements were met in these studies.

**Results:** DIO mice bones showed increased trabecular-cortical bone relation (p<0.05), higher adipogenic marker genes and Node expression, evidencing low bone quality. In these bones, proteomic analysis depicted a beta-oxidation pathway enrichment. In Node silenced-C3H10T1/2 differentiated to osteoblast the expression of the beta-oxidation genes was slightly diminished. This effect was significant (p<0.05) for almost all genes when the cells underwent adipogenesis. In human bone samples, the expression of the Node strongly correlated (p<0.05) with the BMI and the expression of the beta-oxidation genes studied.

**Conclusions:** The TFx node related to the harmful effects of bad diet and obesity in bone through adipogenesis-osteoblastogenesis balance disruption is closely related to beta-oxidation pathway. Hence, beta-oxidation emerges as a potential new therapeutic target for bone alterations linked to bone adiposity.

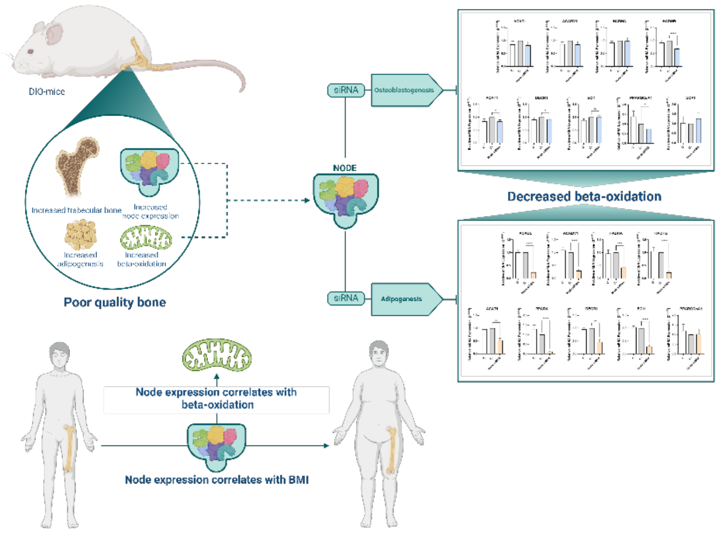

**P094**

### Regulation of bone mass by AXL receptor tyrosine kinase

*<u>Mubashir Ahmad</u>[1,2], Christoph Kölbl[2], Irfana Jan[1,3], Iris Baum[2], Ann-Kristin Dorn[1], Ulrike Kelp[1], Dilay Yilmaz[1], Alessa Wagner[1], Maja Vujic Spasic[1], Benjamin Thilo Krüger[2], Sooyeon Lee[1], Anita Ignatius[2], Jan Tuckermann[1]*

*[1]Institute of Comparative Molecular Endocrinology, Ulm University, Ulm, Germany*

*[2]Institute of Orthopaedic Research and Biomechanics, Ulm University, Ulm, Germany*

*[3]Institute of Immunology, Ulm University, Ulm, Germany*

### Abstract Text

**Introduction:** Osteoporosis is characterized by reduced bone mass and compromised bone microarchitecture, leading to a significantly increased risk of fractures, particularly in older individuals. The most widely available small-molecule therapies for osteoporosis are antiresorptive agents. While these treatments help reduce fracture risk by preserving bone mass, they do not improve bone quality. Identifying novel molecular regulators of osteoblast activity, such as those revealed through RNAi screening of druggable targets, offer promising new therapeutic avenues.

**Purpose:** This study leverages RNAi screening to identify molecular regulators of osteoblast differentiation, with a particular emphasis on Axl as a potential therapeutic target.

**Methods:** We utilized RNAi screening, Alkaline phosphatase (Alp) and Alizarin red S staining, qPCR, Micro-computer tomography (µCT), Histomorphometry, Western blotting, RNAseq, and statistical analysis.

**Results:** We conducted an unbiased high-throughput RNAi screen in primary murine calvarial osteoblasts, using Alp activity as a functional readout. This approach identified *Axl* as a novel and potent suppressor of osteoblast differentiation. *In vitro*, siRNA-mediated knockdown of *Axl* or pharmacological inhibition with the specific inhibitor BGB324 increased Alp activity and mineralization by 5.0- and 1.5-fold, and 5.6- and 2.7-fold, respectively. Moreover, µCT analysis of femurs from 13-week-old female BALB/c mice treated with BGB324 demonstrated a significant increase in bone volume fraction (BV/TV), attributed to an elevated bone formation rate *in vivo* (Fig. 1A). Expression profiling revealed that *Axl* depletion upregulated *Isg15* expression (Fig. 1B-C). Further analysis revealed that knockdown of *Isg15*impaired osteoblast differentiation (Fig. 1D), indicating that *Axl* regulates osteoblast differentiation through the modulation of *Isg15* expression.

**Conclusion:** Our research highlights a previously unknown role of *Axl* in regulating osteoblast differentiation and bone mass, suggesting it as a potential target for osteo-anabolic therapies to treat osteoporosis and other bone disorders.

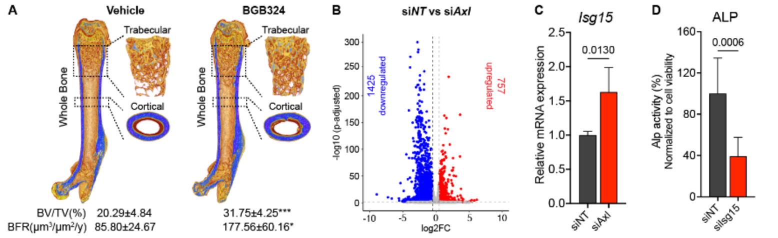

**Fig. 1: Inhibition of Axl with BGB324 increases bone mass via modulation of *Isg15* expression.** (A) Representative µCT images of femurs from mice treated with either vehicle or BGB324 for 2 weeks, highlighling trabecular parameter such as percent bone volume fraction - BV/TV (%) (n=7/group), and bone formation rate - BFR ((µm³/µm²/y) (n=4-5/group). (B) Volcano plot illustrating differentially expressed genes (DEGs) following siRNA-mediated knockdown of *Axl*. (C) *Isg15* expression levels after siRNA-mediated Axl knockdown, measured 8 days post-transfection (n=4/group). (D) Cellular Alp staining after siRNA-mediated knockdown of *Isg15* assessed 8 days post-transfection. Statistical differences between the two groups were determined using one-way ANOVA, followed by Tukey's test.

**P095**

**Effect of teriparatide treatment on amino acid metabolism during osteoblast differentiation**

*Mithra Sophie Gruber*[1], *Franziska Haas*[1], *Viktoria Klingerstorff*[1], *Marco Balasso*[1], *Sabine Pailer*[1], *Sigrid Simonetti*[1], *Markus Herrmann*[1], *Giovanny Rodriguez-Blanco*[1], *Nerea Alonso*[1]
[1]*Medical University of Graz, Clinical Institute for Medical and Chemical Laboratory Diagnostics, Graz, Austria*

**Abstract Text**

Genome-wide association analysis identified rs6430612 associated with poorer teriparatide response in patients with osteoporosis. This SNP is an eQTL for aspartate aminoacyl-tRNA synthetase (DARS). It belongs to a multicomplex regulating amino acid incorporation during protein biosynthesis. Amino acids can also play a signalling role in the regulation of cell survival, growth, and development. We aim to investigate the role of teriparatide treatment in amino acid metabolism in osteoblasts. MC3T3-E1 osteoblast-like cell line was treated with 50nM TPTD for 24h at both undifferentiated stage and after 10 days of osteoblastic differentiation (n=3). An omics-scale metabolomics method using HILIC chromatography was used to profile intracellular polar metabolites. Global proteomics was performed on cell pellets using in-solution trypsin digestion and liquid chromatography coupled to a Bruker TIMS-TOF mass spectrometer operated in DIA-PASEF mode. Skyline, DIA-NN, and Perseus software were used for downstream analysis. Non-parametric statistical analysis was performed in

GraphPad Prism v10. False discovery rate (1%) was applied for multiple testing correction (q value). Eighteen amino acids were significant at least in two replicates and up to 540 proteins were differentially expressed after TPTD treatment. A trend in reduced DARS expression was found in osteoblasts after Teriparatide (FC=0.89). Aspartate levels were increased during osteoblast differentiation (fold change (FC) $_{range}$=1.5-1.7, $q_{range}$ =0.02-0.001). Teriparatide also increased the abundance of 16 amino acid metabolites ($FC_{range}$=1.2-4.8, $q_{range}$=0.04-0.005) and reduced citrulline levels (FC=0.3, q=0.005) in differentiated osteoblasts, whilst it downregulated six aminoacyl-tRNA synthetases ($FC_{range}$=0.7-0.8, $q_{range}$=0.04-0.01). Treatment regulated the expression of proteins associated with extracellular matrix organisation (n=19, q=$3.4x10^{-8}$), cell cycle (n=12, q=$5.8x10^{-6}$), IGF transport (n=10, q=$3.9x10^{-5}$), and signalling by Receptor Tyrosine Kinases (n=14, q=0.007) pathways. Our results suggest that teriparatide increases the availability of some free amino acids in osteoblasts, thus promoting their role as signalling molecules for pathways associated with cell differentiation and bone matrix formation.

**P096**

**Is Omega 3 good or bad for bone development: lessons from cells and fishes**

*Gabrielle N Dudek[1], Rajashekar Donaka[1], David Karasik[2], Cheryl L Ackert-Bicknell[1]*
*[1]University of Colorado, Department of Orthopedics, Aurora, United States*
*[2]Bar-Ilan University, Azrieli Faculty of Medicine, Safed, Israel*

**Abstract Text**

Several omega 3 poly-unsaturated fatty acids (ω3 PUFAs), including Alpha-linolenic acid (ALA), are essential nutrients in mammals. They are perceived to have many health benefits and are widely used as nutritional supplements. No Tolerable Upper Intake Levels exist but the US Institute for Medicine notes that high doses of ω3 PUFAs may have serious adverse effects. The impact of high doses of ω3 PUFAs on the developing skeleton are not known. We treated hFOB cells (a human fetal osteoblast cell line) for 10 days during osteoblasts maturation with 0 (vehicle), 10, 20 and 30 uM ALA. In the 30 uM ALA treated cells, we saw suppressed mineralization that was not observed at any other dose. While still viable, cell proliferation was suppressed at 20 uM of ALA exposure, with no effect on mineralization. In parallel, zebrafish were fed diet supplemented with ALA in doses ranging from 26 g ALA/1000g diet ("baseline" levels in unmodified diet) to 130g ALA/1000g diet from 5-7 days post fertilization (dpf). Any dose above 52g ALA/1000g diet (2X baseline) resulted in larval death. To assess skeletal formation, zebrafish were fed either the baseline or the 2X dose from 5-7 dpf and were imaged at 7 (larvae) and 28 dpf (juveniles). At 7 dpf, the 2X exposed larvae had smaller parasphenoid bones ($p<0.0001$; baseline: 9.54±2.10% vs. 2X: 2.89±1.97% alizarin red staining per ROI). Larvae exposed to the 2X dose from 5-7 dpf and then switched to baseline diet had reduced standard length at 28 dpf (p=0.001; baseline: 4.73±0.44mm vs. 2X: 4.30±0.47mm) and smaller calcified skeletal structures as determined by microCT (Figure 1). Our results suggest that ω3 PUFA over-exposure affects osteoblast mineralization and delays skeletal development. Future studies will determine the mechanism by which ω3 PUFAs affect skeletal development and the effect on the osteoblast.

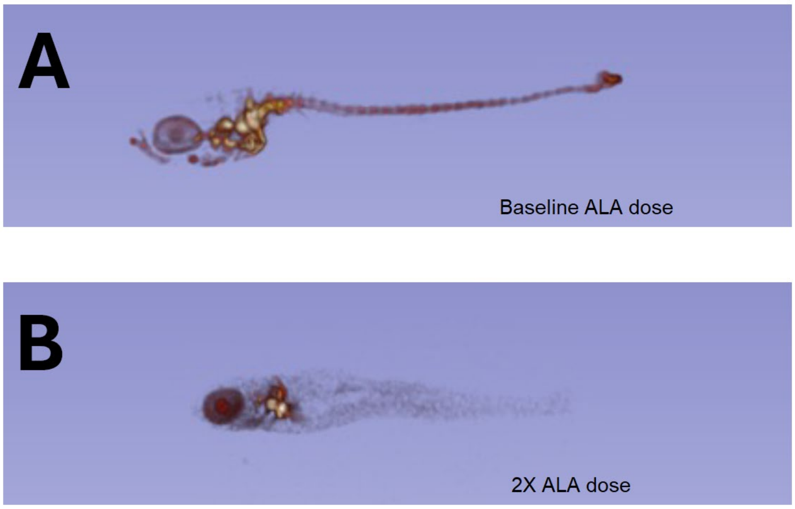

Fig 1. microCT of 28 dpf zebrafish

**P097**

**Cereblon regulates osteoblast differentiation and functions through potassium and calcium channels in mouse primary cultured osteoblasts**

*Yu-Mi Yang*[1], *Dong Min Shin*[1]
[1]*Yonsei University College Of Dentistry, Department of Oral Biology, Seoul, Korea- South*

**Abstract Text**

**Backgroud:** Cereblon (CRBN) is a multifunctional protein located in the cytoplasm, nucleus, and peripheral membranes of various tissues. CRBN is closely related to the proliferation and metabolism of normal cells as well as tumor cells and is a direct target of immunomodulatory drugs. It can lead to several diseases including cardiovascular disease, obesity, diabetes, fatty liver, and neurodegenerative diseases by abnormal cellular metabolism of CRBN. However, the role and the molecular mechanism of CRBN in differentiation of bone cells remain unknown.

**Purpose:** In this study, we investigated the role of CRBN in osteogenic differentiation using CRBN knock-out (KO) mice.

**Methods:** In vivo animal studies, mice of wild-type (WT) and CRBN KO (8-10 weeks old) were analyzed the morphology and mass levels of the bones using micro-CT and histopathological sections. In vitro, isolated osteoblast precursor cells from male murine long bones through a process of extended collagenase digestions were differentiated. Osteoblast differentiation and function were assessed by alizarin red staining and gene expression analysis (qRT-PCR).

**Results:** The deletion of *CRBN* gene was markedly increased the bone density of the femur (~1.36-fold), especially in male CRBN KO mice (~1.52-fold). We found that CRBN KO cells enhanced potency of osteoblastic differentiation compared to WT mice using methods of alkaline phosphatase and alizarin red staining. We also identified that the mRNA expression of osteogenic genes significantly regulated by treatments of blockers of $Ca^{2+}$ and $K^{+}$ channels during osteoblast differentiation of CRBN KO cells compared to WT cells.

**Conclusion:** These results suggest that CRBN plays a critical role for osteoblastogenesis in bone remodeling.

**P098**

**Prior treatment with sequences of RANKL inhibitor and bisphosphonates modifies the tissue-level response of vertebral cancellous and cortical bone to sclerostin antibody in ovariectomised rats**

*Agathe Bedard[1], Denise Dwyer[2], Melanie Felx[1], Martin Guillot[1], Amy Bourdeau[3],*
*Marina Stolina[3], Jen Timoshnako[4], Zhigang Yu[5], Mary Oates[5], Rogely Waite Boyce[3], Serge Ferrari[6]*
*[1]Charles River Laboratories, Montreal ULC, Senneville, Canada*
*[2]Amgen Inc, Cardiometabolic Disorders, Thousand Oaks, United States*
*[3]Amgen Inc, Translational Safety and Bioanalytical Sciences, Thousand Oaks, United States*
*[4]UCB, Bone Health Mission, Slough, United Kingdom*
*[5]Amgen Inc, Global Development, Thousand Oaks, United States*
*[6]Geneva University Hospital, Division of Bone Diseases, Geneva, Switzerland*

**Abstract Text**

**Background**: Prior antiresorptives can reduce net gain of BMD with romosozumab.

**Purpose:** To explore how various preceding treatment sequences with a bisphosphonate (BP) and/or OPG-Fc (RANKL inhibitor) affect early, tissue-level response to sclerostin antibody (Scl-Ab) in vertebral cancellous (Cn) and cortical bone in ovariectomised rats.

**Methods**: Following ethical approval, rats were administered various sequences of vehicle, OPG-Fc, and zoledronic acid (ZOL) or alendronate in Cycle 1 (0–12 weeks) or Cycle 2 (12–18 weeks) before Scl-Ab in Cycle 3 (18–24 weeks) (**Fig 1A**). DXA was performed at baseline and post cycles. Assessments included: bone turnover markers (BTMs) throughout, mineralising surface/bone surface and modelling-based bone formation (MBF; Weeks 18, 20 and 24) and remodelling-based bone formation (RBF; Weeks 20 and 24) on Cn and endocortical surfaces.

**Results**: In Group 1, anticipated responses in BTMs, BMD (**Fig 1B, C**), and dynamic histomorphometry (**Fig 1D, E**) were observed after 2 (Week 20) and 6 weeks (Week 24) of Scl-Ab. At Week 24, Scl-Ab-induced bone mass accrual was blunted in all rats with preceding antiresorptive sequences (ARS). In Groups 2, 4 and 5 (direct transition from OPG-Fc to Scl-Ab), suppression of bone-forming surfaces (BFS) was marked at Week 20, with limited MBF. In Group 3, MBF was only modestly reduced at Week 20. By Week 24, groups with preceding ARS were similar in BFS, but relative contribution of MBF and RBF differed. Group 5, which included a treatment-free period preceding Scl-Ab, demonstrated reduced MBF and increased RBF, hence opposing effects on bone mass accrual, whereas in Group 3, RBF increased slightly. In Groups 2 and 4, only MBF was present.

**Conclusion**: All prior ARS blunted Scl-Ab bone-formation effects. BP exposure improved subsequent Scl-Ab BMD response. Interposing ZOL between OPG-Fc and Scl-Ab improved early BFS response compared with direct transition from OPG-Fc.

**Funding:** UCB and Amgen Inc.

**Figure:** Effect on the response to Scl-Ab in ovariectomised rats administered 2 cycles of various sequences of Veh, OPG-Fc, and a BP (ZOL or Aln) before receiving Scl-Ab in Cycle 3. A) study schema; B and C) percentage change in BMD; and E and F) percentage remodelling and modelling active bone formation referent to total bone surface.

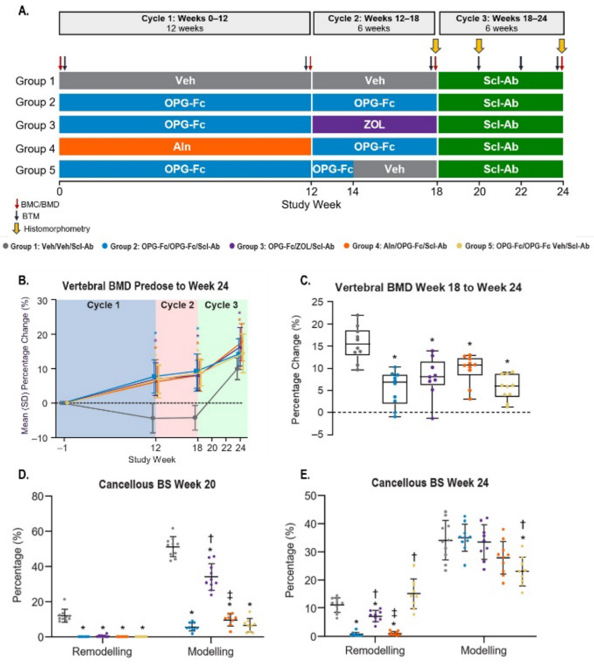

*P ≤ 0.05, significantly different from Group 1 (Veh/Veh/Scl-Ab). †P ≤ 0.05, Group 3 (OPG-Fc/ZOL/Scl-Ab) or Group 5 (OPG-Fc/OPG-Fc-Veh/Scl-Ab) significantly different from Group 2 (OPG-Fc/OPG-Fc/Scl-Ab). ‡P ≤ 0.05, Group 4 (Aln/OPG-Fc/Scl-Ab) significantly different from Group 3 (OPG-Fc/ZOL/Scl-Ab). Aln, alendronate; BMD, bone mineral density; BTM, bone turnover marker; BS, bone surface; Scl-Ab, sclerostin antibody; Veh, vehicle; ZOL, zoledronic acid.

### P099

### FasL impact on extracellular tooth and bone matrix factors Tgfb3 and Tnc

*Adela Kratochvilova[1], Lucia Kytkova[1,2], Martina Zapletalova[1], Matous Jedlicka[1,3], Barbora Sedlackova[1,3], Reinhard Gruber[4], Eva Matalova[1]*
*[1]Institute of Animal Physiology and Genetics- Czech Academy of Sciences, Laboratory of Odontogenesis and Osteogenesis, Brno, Czech Republic*
*[2]Masaryk University, Department of Biology, Brno, Czech Republic*
*[3]Masaryk University, Department of Experimental Biology, Brno, Czech Republic*
*[4]Medical University Vienna, University Clinic of Dentistry, Vienna, Austria*

### Abstract Text

**Introduction**: Fas ligand (FasL) in the bone is well known in osteoblast-induced osteoclast apoptosis but the bone phenotype of FasL deficient (gld) mice indicates also non-apoptotic engagement of the ligand. The impact of FasL on the tooth supporting alveolar bone was reported under healthy and induced inflammatory conditions recently. Alongside, FasL was shown to be required for osseous healing in the extraction socket.

**Methods:** To provide a complex insight into the impact of FasL deficiency on extracellular matrix (ECM) formation, gld derived precursors were differentiated into osteoblastic cells and compared with controls based on RNA Sequencing (Lexogen) approach.

**Results:** The analysis revealed statistically significant changes in expression of more than 500 genes associated with the ECM pathways. Notably, 15 % of the factors were localized directly in the ECM. Among the altered genes, Tgfb3 and Tnc were detected, both known as targets of the transcription factor Prrx1. To follow this link, IDG-SW3 osteoblasts were challenged by soluble FasL. The results displayed a vice versa effect on expression of Tgb3 as well as Tnc compared to the FasL deficient cells. Additionally, Prrx1 silencing was performed and the results indicated involvement of Prrx1 as a possible intermediate step in the effect of FasL on Tgfb3 and Tnc.

**Conclusion:** In this investigation, FasL was demonstrated to impact expression of Tgfb3 and Tnc, factors participating in tooth and bone mineralization. The findings further support the increasing evidence about the non-apoptotic functions of FasL in tooth-bone homeostasis. Notably, FasL in IDG-SW3 cells dramatically decreased expression of sclerostin, a therapeutic target in osteoporosis as well as regenerative dentistry. In this case, one possible link may be Mef2c, the research is under way.

**Acknowledgement:** The research runs under the project GACR 23-06660S (Czech Science Foundation).

**P100**

**Spatial transcriptional profiling of osteoprogenitors proximate to osteoclasts in human bone remodeling**

*<u>Xenia Borggaard</u>[1], Malene Hykkelbjerg Nielsen[1], Chin-Hsin Huang[1], Kristine Tribler Edwardsen[1], Sam Khac Dinh[1], Julia F Charles[2], Christina Møller Andreasen[1], Thomas Levin Andersen[1]*
*[1]University of Southern Denmark, Molecular Bone Histology Lab- Dept. of Clinical Research, Odense, Denmark*
*[2]University of Southern Denmark, Molecular Bone Histology Lab- Dept. of Clinical Research, Boston, United States*

**Abstract Text**

Crosstalk between bone cells is essential in bone remodeling. Reversal cells (Rv.Cs) are osteoprogenitors near osteoclasts (OCs), which may play a central and overlooked role in this process. In human trabecular bone, Rv.Cs express early osteoblastic markers and collagenases like MMP13, indicating a catabolic function in cleaning eroded surfaces from OC debris. They also express receptors for coupling factors, suggesting a pro-formative role. This study aims to further characterize Rv.Cs and their partnership with OCs in human intracortical bone remodeling.

Decalcified paraffin-embedded cortical bone samples from adolescents (aged 12-15 years) were analyzed using GeoMX$^{TM}$ digital spatial profiling (GeoMx). With TRAcP/CD34 immunofluorescence staining guiding the identification of Rv.Cs, osteoblasts, and OCs for selection of ROIs. Further sections were stained for MMP13, or RANKL using in situ hybridization (ISH) combined with TRAcP immunostaining.

GeoMx analysis included 12 active and 4 inactive remodeling events. As expected, osteoblasts expressed genes like IBSP, COL1A1, and BGLAP, while OCs expressed MMP9, ACP5, TCIRG1, and CTSK. Furthermore, GeoMx identified differentially expressed genes (DEGs) between Rv.Cs and osteoblasts included IGFBP5, TNC, BGLAP, and MMP13, while DEGs between Rv.Cs and OCs included BGN, FN1, and SPARC. Histological analysis of MMP13 expression in 7596 cells from 6 bone samples identified 16% MMP13-positive cells. Rv.Cs on eroded surfaces (n=1154) had significantly higher MMP13 expression than mature osteoblasts (n=743, p<0.0001) and bone lining cells (n=428, p<0.0001). Rv.Cs near OCs (<25 µm) had higher MMP13 expression than those further away (>25 µm). RANKL expression, evaluated by ISH and TRAcP immunostaining, showed a similar pattern with higher expression near OCs. Co-expression of MMP13 and RANKL aligns with published scRNA-seq data from mice and warrants further evaluation in humans.

GeoMx is a promising method for investigating Rv.Cs, and additional samples may reveal more about this cell population.

**P101**

**The role of nuclear lamins A/C in bone development**

*Stéphane Blouin[1,2], Nadja Fratzl-Zelman[1,2], Markus Hartmann[1,2], Claudia Hufnagel[3], Phaedra Messmer[1], Silvia Spitzer[3], Wayne Cabral[4], Francis Collins[4], Mike Erdos[4], John Dunlop[5], Andreas Roschger[5], Barbara Schamberger[5,6], Maria Eriksson[7], Charlotte Strandgren[7,8], Daniel Whisenant[7], Thomas Dechat[2,3]*

*[1]Ludwig Boltzmann Institute of Osteology at Hanusch Hospital of OEGK and AUVA Trauma Centre Mei dling, UKH Meidling, Vienna, Austria*

*[2]Vienna Bone and Growth Center, Ludwig Boltzmann Institute of Osteology, Vienna, Austria*

*[3]Ludwig Boltzmann Institute of Osteology at Hanusch Hospital of OEGK and AUVA Trauma Centre Mei dling, 1st Medical Department- Hanusch Hospital, Vienna, Austria*

*[4]National Human Genome Research Institute- National Institutes of Health, Molecular Genetics Section- Center for Precision Health Research, Bethesda, United States*

*[5]Paris-Lodron University of Salzburg, Department of the Chemistry and Physics of Materials, Salzburg, Austria*

*[6]Heidelberg University, Institute for Molecular Systems Engineering and Advanced Materials, Heidelberg, Germany*

*[7]Karolinska Institute, Department of Biosciences and Nutrition, Huddinge, Sweden*

*[8]Karolinska Institute, Department of Oncology-pathology, Solna, Sweden*

**Abstract Text**

Nuclear lamins, intermediate filament proteins, are the main components of the nuclear lamina, a filamentous meshwork underlying the inner nuclear membrane. They are classified into A- and B-types. The major A-types, lamins A and C (LAC), are involved in many cellular functions including transcription, differentiation and mechanotransduction. Mutations in LAC have been linked to various human diseases termed laminopathies. Amongst those, the premature aging disease Hutchinson-Gilford Progeria Syndrome (HGPS) represents also a bone phenotype amongst other pathologies.

To study the role of LAC in bone development we investigated 1) the effect of LAC deficiency (LAC-ko) on extracellular matrix (ECM) organization and on the formation of twisted plywood-like tissue on curved surfaces using murine pre-osteoblast MC3T3-E1 cells and 2) bone mineralization and growth plate organization in the *Lmna*$^{G609G/G609G}$ HGPS mouse model.

Wildtype (WT) and LAC-ko MC3T3-E1 cells were grown on scaffolds harboring a constant mean curvature and induced for osteogenic differentiation up to 32 days. Cells were analyzed at various days post induction using immunofluorescence (confocal and light-sheet) and second harmonic generation microscopy. While WT cells changed their orientation from a right-handed to a left-handed pattern during growth and differentiation, the LAC-ko cells maintained the original right-handed chiral pattern. In addition, the ECM formed by the LAC-ko cells appears looser compared to the WT ECM.

Quantitative backscattered electron imaging of femurs and humeri revealed similar mineral content between WT and *Lmna*$^{G609G/G609G}$ mice. The osteocyte lacunae features were also similar. However, more empty lacunae were found in *Lmna*$^{G609G/G609G}$ bones. Bone histomorphometry and histology demonstrated decreased bone volume per tissue volume, as well as growth plate dysplasia with thinner unmineralized resting and proliferative zones in the *Lmna*$^{G609G/G609G}$ mice versus WT. Our data suggest that LAC is more involved in establishing a correct ECM and growth plate than in bone mineralization.

**P102**

**STAT3 hyperactivation induced in the adult murine skeleton in osteoblasts and osteocytes causes a region-specific increase in cortical porosity**

*<u>Natalie Koh</u>[1], Narelle McGregor[1], Natalie Wee[2], Christa Maes[3], Natalie Sims[1]*
*[1]St. Vincent's Institute of Medical Research, Bone Cell Biology and Disease, Fitzroy, Australia*
*[2]St. Vincent's Insitute of Medical Research, Bone Cell Biology and Disease, Fitzroy, Australia*
*[3]Laboratory of Skeletal Cell Biology and Physiology SCEBP- Skeletal Biology and Engineering Research Center, Department of Development and Regeneration, Leuven, Belgium*

**Abstract Text**

Although cortical porosity increases with ageing, the signals responsible are unknown. Previous work showed that the juvenile murine skeleton was highly porous when STAT3 signalling was stimulated in osteoblasts and osteocytes by constitutive targeted deletion of the suppressor SOCS3 (Suppressor of Cytokine Signalling 3). We sought to determine whether delaying SOCS3 deletion and STAT3

hyperactivation in osteoblasts and osteocytes until adulthood induces cortical porosity in consolidated cortical bone.

We generated tamoxifen-dependent SOCS3-deficient mice with an Ai9.tdTomato fluorescent tag to localise recombination (*iDMPCre⁺.Ai9. Socs3*$^{ff}$). Bone-specific *Socs3* recombination was induced by 4x 20mg/kg doses of tamoxifen between 12 and 14 weeks of age in female *iDMPCre⁺.Ai9. Socs3*$^{f/f}$ mice and confirmed by PCR. By cryo-histology, ~100% of trabecular osteocytes were tdTomato+. In cortical bone, Cre-mediated recombination was region-specific: ~80% of osteocytes in the metaphysis were tdTomato+, but only ~50% were tdTomato+ in the diaphysis.

Longitudinal micro-CT analysis at 16, 20, and 26 weeks of age detected 5-fold greater cortical porosity, restricted to the metaphysis, in tamoxifen-treated *Socs3*-deficient mice compared to tamoxifen-treated controls (*iDMPCre⁺.Ai9*). By 32-weeks, greater porosity (~2.4-fold) also emerged at the diaphysis. Additionally, at all timepoints, the proportion of low-density cortical bone was significantly greater (~20%) in *Socs3*-deficient mice compared to controls, but only at the metaphysis. TRAP-staining and double calcein labelling revealed greater intracortical and endocortical resorption and formation in *Socs3*-deficient mice. Ploton silver staining showed that osteocytes in *Socs3*-deficient mice were not lamellar, but were haphazardly arranged, in rounder lacunae, and with more dendritic processes.

These data indicate that induced SOCS3 deletion in osteoblasts and osteocytes in consolidated cortical bone increases cortical remodelling and porosity. This commences in the less mature metaphysis and over time, extends to the more mature diaphysis. This suggests that the age-related increase in cortical porosity and bone fragility may arise due to STAT3 hyperactivation.

**ND-P003**

**Hypergastrinemia and Bone Fractures: Elucidating the Effects of Proton Pump Inhibitors**

*<u>Aine Pears</u>[1], Malcolm Boyce[2], Joanna Seiffert[2], Alan J. Stewart[3], Gerry McLachlan[1], Colin Farquharson[1]*
*[1]University of Edinburgh, The Roslin Institute, Edinburgh- Midlothian, United Kingdom*
*[2]Trio Medicines Ltd, Trio Medicines Ltd, London, United Kingdom*
*[3]St Andrews, School of Medicine, St Andrews- Fife, United Kingdom*

**Abstract Text**

Proton pump inhibitors (PPIs) are amongst the most prescribed medicines in the world with more than 70 million prescriptions issued during 2023 in the UK alone. The safety records of PPIs are generally favourable but epidemiological studies suggest an increase in fracture risk associated with chronic PPI use. The underlying mechanisms of this association have not yet been elucidated. The most commonly accepted explanation is that PPIs cause calcium malabsorption, leading to hyperparathyroidism and increased osteoclastic bone resorption. However, these data are inconsistent and often disputed. In this study, alternative mechanisms for the increased fracture risk were explored, with the overall aim of ascertaining whether this may be mediated by hypergastrinemia; a consequence of chronic PPI use.

It is unknown if osteoblasts express gastrin receptors. Therefore, as an initial step in this investigation, primary human osteoblasts were evaluated for functional gastrin receptors. Primary human osteoblasts were analysed by RT-qPCR, western blot, and immunocytochemistry, revealing the presence of the gastrin/cholecystokinin-2 (CCK-2) receptor. Furthermore, the functionality of the receptor was assessed, where gastrin induced phosphorylation of ERK 1/2 and JNK but not p38 or AKT, suggesting gastrin utilises the MAPK intracellular signalling pathway. Gastrin treatment reduced the expression of genes associated with osteoblast differentiation and mineralisation; collagen type 1 (*COL1A1*), $p<0.01$; osteopontin (*SPP1*), $p<0.001$; osteocalcin (*BGLAP*), $p<0.05$; PHOSPHO1, $p<0.001$ and osteonectin (*SPARC*), $p<0.05$. Additionally, gastrin inhibited proliferation (0.1 – 10nM, $p<0.05$) and reduced alizarin red staining (0.01 – 10nM, $p<0.01$). In contrast, alkaline phosphatase activity was increased by gastrin (control media: 53.6 µmol/L/min), 10µM gastrin; 87.7 µmol/L/min), $p<0.001$) and inversely correlated with matrix mineralisation.

These data establish the presence and functionality of osteoblast gastrin receptors raising the possibility that the elevated gastrin levels that occur as a consequence of PPI use may act directly on osteoblasts leading to deficits in bone formation.

**Poster Sessions: Osteoclasts and bone resorption**

**P104**

**Spontaneously circulating hematopoietic stem/progenitor cells from TCIRG1-defective osteopetrosis patients at single cell resolution: implication for gene therapy**

*Valentina Capo*[1,2], *Erika Zonari*[1], *Fabio Bedin*[1], *Valeria Iannello*[1,3], *Matteo Barcella*[1], *Alessandra Zecchillo*[1,4], *Elena Draghici*[1], *Elena Rizzoli*[1], *Beatrice Martini*[1], *Robert Chiesa*[5], *Alper Gezdirici*[6], *Ivan Merelli*[1,7], *Matteo Maria Naldini*[1], *Bernhard Gentner*[1,8], *Anna Villa*[1,2]

*[1]IRCCS San Raffaele Scientific Institute, San Raffaele Telethon Institute for Gene Therapy SR-Tiget, Milan, Italy*

*[2]Consiglio Nazionale delle Ricerche CNR, Milan Unit- Institute of Genetic and Biomedical Research IRGB, Milan, Italy*

*[3]Vita-Salute San Raffaele University, Molecular Medicine, Milan, Italy*

*[4]University of Milano Bicocca, Translational and Molecular Medicine DIMET, Milan, Italy*

*[5]Great Ormond Street Hospital, Department of Bone Marrow Transplant, London, United Kingdom*

*[6]University of Health Sciences Basaksehir Cam and Sakura State Hospital, Department of Medical Genetics, Istanbul, Turkey*

*[7]Consiglio Nazionale delle Ricerche CNR, Institute for Biomedical Technologies ITB, Segrate, Italy*

*[8]University of Lausanne UNIL and Lausanne University Hospital CHUV, Ludwig Institute for Cancer Research and Department of Oncology, Lausanne, Switzerland*

**Abstract Text**

**Introduction:** CD34+ hematopoietic stem/progenitor cells (HSPCs) recirculate at low levels in the peripheral blood for immune-surveillance and hematopoietic homeostasis. Patients affected by *TCIRG1*-defective autosomal recessive osteopetrosis (ARO), characterized by defective osteoclasts, show high levels of spontaneously circulating HSPCs, due to their high bone density. The standard of care is allogeneic hematopoietic stem cell (HSC) transplantation, while lentiviral vector (LV) gene therapy (GT) represents a valuable alternative. However, GT must rely on the manipulation of circulating HSPCs (cHSPCs), the only source available in ARO.

**Purpose:** Here we studied if the prolonged circulation outside bone marrow (BM) hematopoietic niches impacts on ARO cHSPCs stemness and fitness, at steady state and after *ex vivo* GT and UM171-based expansion.

**Methods:** HSPCs from 3 ARO patients were analysed by single-cell RNA sequencing and transplanted in NSG mice.

**Results:** We identified 17 clusters, encompassing primitive HSPCs and committed progenitors. At steady-state, ARO cCD34+ cells contain HSCs and multipotent progenitors (MPPS) similarly to healthy cord blood CD34+ cells, with lower content of megakaryocyte/erythroid-biased MPPs in favour of lympho/myeloid primed progenitors. These results suggest that in ARO, the lack of adequate BM niches induces circulation of populations normally retained within the BM. Following UM171 expansion, we observed the sustained presence of HSCs and early progenitors, which aligns with the long-term engraftment and repopulation capacity of the expanded product seen in NSG mice (with human engraftment in BM 1.5%-33.4%). UM171-expansion also resulted in the increase of myeloid progenitors, that will contribute to the differentiation of gene-corrected osteoclasts after GT. No skewing of subset composition was observed after LV transduction.

**Conclusion:** We identified distinct characteristics of ARO HSPCs, supporting their use for LV gene therapy. Differential gene expression analyses will offer valuable insights to optimize GT and may reveal new pathways involved in regulating CD34+ cell egress from hematopoietic niches.

**P105**

**Activin-A has dual roles in osteoclast formation and foreign body giant cell differentiation**

*Ton Schoenmaker[1], Elina Kylmäoja[2], Sami Kauppinen[3], Mikko Finnilä[3], Petri Lehenkari[2], Juha Tuukkanen[2], Teun Vries de[1]*
*[1]ACTA, Department of periodontology, Amsterdam, Netherlands*
*[2]University of Oulu, Department of Anatomy and Cell Biology, Oulu, Finland*
*[3]University of Oulu, Faculty of Medicine, Oulu, Finland*

**Abstract Text**

**Background:** Osteoclasts and foreign body giant cells (FBGCs) are both multinucleated cells derived from the same monocyte precursor, but they have distinct functions. Osteoclasts are specialized cells responsible for bone resorption while FBGCs form in response to foreign material in tissues, participating in immune reactions and inflammation, but not in bone remodeling. Activin-A belongs to the transforming growth factor-beta superfamily. It plays diverse roles in the body, including regulation of inflammation, and immune regulation. Recently, we have shown that Activin A induces fewer, but larger and more active osteoclasts.The effect of Activin-A on the differentiation of FBGCs is not known. Both osteoclasts and FBGCs are crucial in the context of bone implants because osteoclasts are responsible for bone resorption and remodeling, which is essential for the integration of the implant. FBGCs on the other hand, play a role in the immune response to the foreign material, influencing the body's reaction and long-term success of the implant.

**Purpose:** To investigate the effect of Activin A on the differentiation of osteoclasts and FBGCs from human CD14-positive monocytes.

**Methods:** Number of multinucleated cells and cell area were measured after 14 and 21 days and QPCR was performed after 7, 14 and 21 days of culturing.

**Results:** Activin A induced significantly fewer , but larger osteoclasts. In contrast, Activin A did not have an effect on FBGC number, but decreased the cell area in FBGC cultures.

qPCR showed that Activin-A seems to upregulate the expression of several genes such as TRAcP, CIITA, OLR1, RHOBTB1 and ALK4, mainly in osteoclasts.

**Conclusion:** These results show that Activin-A has a different effect on osteoclasts compared to FBGCs, despite the similarities between these two cell types. This difference could be caused by a difference in the expression in the canonical Activin-A receptor ALK4.

**P107**

**Alendronates effect on osteoclasts in a rat model of cortical bone remodeling**

*Birgitte Villadsen*[1,2]*, Simone Nielsen*[1]*, Pankaj Shitole*[3]*, Joseph Wallace*[4]*, Malene Nielsen*[2,5]*, Frank Ko*[3]*, Kyle Anderson*[3]*, Bryan Dulion*[3]*, Ryan Ross*[3]*, Thomas Andersen*[1,2,5,6]
[1]*University of Southern Denmark, Molecular Bone Histology Lab- Dept. of Clinical Research, Odense, Denmark*
[2]*University of Southern Denmark, Danish Spatial Imaging Consortium DanSIC, Odense, Denmark*
[3]*Rush University, Dept. of Anatomy and Cell Biology, Chicago, United States*
[4]*Purdue University, Weldon School of Biomedical Engineering, West Lafayette, United States*
[5]*Odense University Hospital, Dept. of Pathology, Odense, Denmark*
[6]*Aarhus University, Dept. of Forensic Medicine, Aarhus, Denmark*

**Abstract Text**

**Background:** Atypical femoral fractures (AFFs) are a rare but severe side effect of long-term bisphosphonate (BP) treatment. Despite the low prevalence of AFFs, public awareness has significantly reduced adherence to osteoporosis medications. The mechanisms driving BP-associated AFFs remain unclear, with several proposed mechanisms including impaired bone remodeling, altered bone matrix maturation, and the accumulation of tissue microdamage. A critical barrier to understanding how BPs negatively affect skeletal integrity is the difficulty in separating the individual contributions of altered remodeling kinetics (suppressed resorption and formation) from bone matrix maturation. We have addressed this barrier using a recently validated rat model of induced cortical remodeling to investigate the mechanisms and treatments.

**Purpose:** To examine the cellular effect of bisphosphonates on the remodeling process in a rat model of cortical remodeling.

**Methods:** 11-12-weeks-old Lactating Sprague Dawley rats (n=7-8 per group) were treated with a low calcium diet (0.01% calcium) and either the BP alendronate (ALN) (28 µg/kg twice weekly), raloxifene (1 mg/kg 5 days per week), or vehicle (twice weekly). The dams were sacrificed after 7 days, and femurs were fixed and stored in 4%? paraformaldehyde. The mid- and distal-diaphysis bone structure was analyzed by micro-CT, while the proximal-diaphysis was analyzed by dynamic and static molecular histomorphometry. The molecular histomorphometry combined Masson-Trichrome staining with multiplex *in situ* hybridization of the markers *Ctsk*, *Mmp13*, and *Col1a1*.

**Results:** After 7 days of bisphosphonate treatment, osteoclasts were larger with more nuclei, though their number was unchanged. ALN treatment increased eroded pores, while the abundance of reversal cells and osteoblasts were unaffected. Both eroded and formative pores were abundant, indicating a rapid ongoing resorption and formation.

**Conclusion:** Bisphosphonates already affect osteoclast size and fusion after just 7 days in rats, while osteoblastic cells seem unaffected under the investigated conditions.

**Acknowledgement:** This research was supported by the NIH

**P108**

**A Novel Single Cell Approach for In-Depth Characterization of Osteoclast Resorption Dynamics in Health and Disease**

*Antonio Maccataio*[1], *Georg Schett*[1,2], *Ulrike Steffen*[1]
[1]*Universitätsklinikum Erlangen, Department of Internal Medicine 3 - Rheumatology and Immunology, Erlangen, Germany*
[2]*Friedrich-Alexander-Universität Erlangen-Nürnberg, Faculty of Medicine, Erlangen-Nürnberg, Germany*

**Abstract Text**

**Background:** Bone erosions are typical features of various arthritic diseases, including RA. Therefore, Osteoclasts (OCs) have become a main therapeutic target to prevent long-term bone loss. Exact mechanisms remain unclear, so that it is still controversially debated, whether this increased bone loss is caused by enhanced OC precursor numbers, OC differentiation, or OC activation. But studying OC activity remains challenging, as existing assays only measure OC resorption dependent on OC size and numbers.

**Purpose:** Utilizing a novel single cell approach to investigate OC resorption dynamics of RA patients and healthy controls independent from the limitations of standard osteoclast resorption assays.

**Methods:** Blood monocytes from RA patients and healthy controls were differentiated to OCs and seeded onto calcein-incorporated calcium phosphate-coated (calcein-CaP) plates. OC resorption was recorded using a time-lapse fluorescent microscope. Subsequently, OCs were stained with Hoechst and imaged for nuclei quantification. Using the fluorescence signal of the calcein-CaP coating, the resorption speed was determined, and in combination with the nuclei number, the resorptive capacity (resorption speed/nuclei) of each individual OC was calculated. The resorptive capacity was obtained at different RANKL concentrations to evaluate the mean RANKL dependency (resorptive capacity/Δ[RANKL]).

**Results:** OCs from 43.75% of all RA patients displayed a significantly increased resorptive capacity in comparison to healthy controls (0.0707±0.0071 vs. 0.0456±0.0037 $\mu m^2$/min/nucleus; p=0.0012). This increase in OC resorptive capacity was dependent on joint pain (sensitivity: 100%; selectivity: 77.78%; p=0.0032). OCs from RA Patients with increased resorptive capacity also displayed a significantly decreased RANKL dependency compared to healthy controls ($1.584 \pm 0.2871 \times 10^{-3}$ vs. $4.333 \pm 1.361 \times 10^{-3}$ $\mu m^2$/min/nucleus/ng RANKL; p=0.0218)

**Conclusion:** These findings imply, that OCs derived from RA patients experiencing arthralgia, exhibit an increased resorptive capacity and decreased RANKL dependency compared to OCs from healthy donors. This intrinsic change likely contributes to the accelerated bone loss in RA patients.

**P109**

**Osteoclast Progenitor Gene Signatures Reveal Insights into Trench based Resorption influenced by the Bone Marrow Microenvironment**

*Anne Sofie Ølund[1,2], <u>Neha Sharma</u>[1,2,3], Lars Grøntved[4], Jonas Heilskov Graversen[3], Megan Weivoda[5], Kent Søe[1,2]*

*[1]The University Of Southern Denmark, Dept. of Clinical Research, Odense, Denmark*
*[2]Odense University Hospital, Dept. of Pathology, Odense, Denmark*
*[3]The University of Southern Denmark, Department of Molecular Medicine, Odense, Denmark*
*[4]The University Of Southern Denmark, Dept. of Biochemistry and Molecular Biology, Odense, Denmark*
*[5]Mayo Clinic, Dept. of Hematology, Rochester, United States*

**Abstract Text**

**Background:** Osteoclasts exhibit functional heterogeneity. Studies have shown that osteoclastic bone resorption can be classified into two modes: pit (intermittent) and trench (aggressive resorption). Pit-forming osteoclasts are stationary, while trench-forming osteoclasts move during resorption. Age, post-menopausal status, and bone formation levels of the donor influences the likelihood of osteoclast progenitors to differentiate into trench-forming osteoclasts. How is this possible? Are CD14+ cells already different when harvested for osteoclast differentiation?

**Purpose:** We investigated whether differences in the gene expression profile of C14+ cells from healthy donors can predict the trench resorption potential of resulting osteoclasts, and how such a difference in the CD14+ cells may come about.

**Methods:** Study 1, in vitro, examined osteoclasts generated from CD14+ monocytes isolated from healthy female blood donors (n=10), assessing function through fusion, TRAcP activity, and bone resorption assays. Study 2: RANK+/CD14+ cells were isolated from bone marrow aspirates (healthy women, n=52). RNAseq was performed on CD14+ cells from both studies. Olink protein analyses were conducted on bone marrow aspirates.

**Results:** Study 1: 6 genes correlated negatively (ranked gene expression) with osteoclast number (R=-0.9199, P=0.0002). 12 genes correlated negatively with nuclei/osteoclast (R=-0.9377, P<0.0001). 10 genes correlated positively with % trench surface/bone surface (R=0.9582, P<0.0001). No correlations were observed for pit surface. Study 2: The expression level of 10 genes found to correlate with trenches in Study 1 was analysed in CD14+ cells of Study 2 and correlated with the Olink proteomic profile. This identified 21 proteins, of which 7—CCL4 (R=0.6105, P=0.01), VEGFD (R=0.5605, P=0.02), CCL5 (R=0.54, P=0.03), IL8 (R=0.55, P=0.03), OPN (R=0.54, P=0.03), CXCL1 (R=0.52, P=0.04), and LAPTGFbeta (R=0.52, P=0.04)—were identified as SASP factors linked to biological aging and frailty.

**Conclusion:** The bone marrow microenvironment shapes osteoclast progenitors, where SASP factors also exert their influence. This altered population likely leads to the trench based aggressive form of resorption on bone.

**ND-P004**

**Biomaterial substrate stiffness and mineralization synergistically regulate osteoclast maturation and resorptive activity**

*Micol Bressan*[1], *Tess van Alphen*[1], *Annelieke Wentzel*[1], *Sandra Hofmann*[1], *Miguel Castilho*[1]
[1]*Eindhoven University of Technology, Department of Biomedical engineering, Eindhoven, Netherlands*

**Abstract Text**

**Intro:** Bone is dynamically remodeled by osteoclasts (OCs), whose osteoclastogenesis and activity are influenced by substrate stiffness, especially impacting cell fusion and migration. Additionally, the mineralized bone extracellular matrix further modulates OC behavior, promoting sealing zone formation on the surface and podosome belt formation on non-mineralized substrates [Blangy et al. 2020]. However, the specific interplay between substrate stiffness and mineralization in regulating OC maturation remains unclear.

**Purpose:** Here, we investigate the effects of substrate stiffness and mineralization on osteoclast maturation and resorption in-vitro using 2D biomaterial films, with a focus on sealing zone assembly and resorption pit formation.

**Methodology:** Collagen type 1 and silk fibroin 2D films were casted and subsequently mineralized using a polymer-induced liquid precursor method [De Wildt et al., 2022]. Film mineralization was confirmed via µCT, while stiffness was evaluated using unconfined compression. Human monocytes (hMCs) were isolated from peripheral blood mononuclear cells with purity confirmed by flow cytometry, and seeded on the films. Over 16 days, apoptosis, resorption, and morphology were analyzed using LDH and TRAP assays, scanning electron microscopy (SEM), and fluorescent cell staining.

**Results:** Mineralized silk fibroin (mSF) exhibited approximately three times the stiffness of its non-mineralized counterpart (nmSF) (161±44 kPa vs. 40±19 kPa). In contrast, mineralized collagen (mCOL)

films had a much lower stiffness (0.24±0.11 kPa). Confocal and SEM imaging confirmed the presence of TRAP+ osteoclasts on stiffer substrates (mSF), with sealing zone formation and resorption pits (Fig1A). Despite harbouring TRAP+ cells, nmSF exhibited podosome belts. In contrast, mCOL mainly supported TRAP- mono-nucleated cells (Fig1B).

**Conclusions:** These findings suggest that both stiffness and mineral presence are crucial for OC maturation. The low stiffness of mCOL inhibits OC fusion and maturation, further underscoring stiffness as a key determinant in osteoclast differentiation and function, providing important insights for the design of osteoclastogenesis-supportive biomaterials.

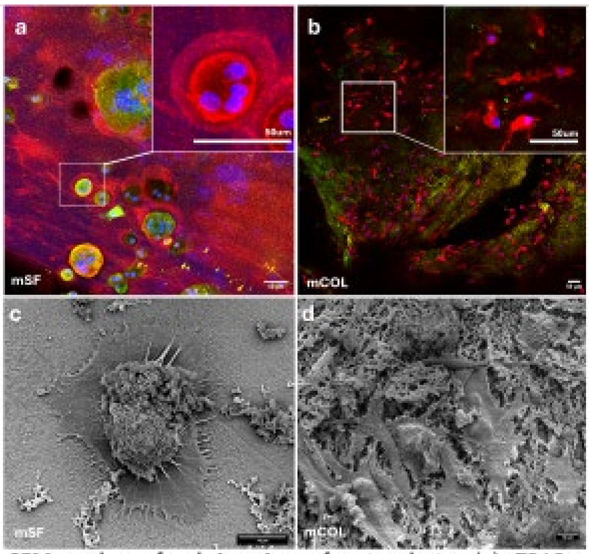

**SEM and confocal imaging of osteoclasts.** (a) TRAP+ osteoclast on mSF films with focus on sealing zone formation; (b) Mononuclear TRAP- cells on mCOL films [Nuclei are stained with Blue, Actin with Red, TRAP with Yellow and CTSK with Green]; (c) Osteoclast-like cell on mSF films; (d) cells on mCOL films. Abbreviations: mSF – Mineralized silk fibroin; nmSF – Non-mineralized silk fibroin; mCOL – Mineralized collagen; TRAP – Tartrate-resistant acid phosphatase; CTSK – Cathepsin K.

**ND-P005**

**Comparative phenotyping of osteoclast progenitors in immune-mediated inflammatory diseases: rheumatoid arthritis and diabetes mellitus type 1**

*Sara Aničić[1], Marta Radošević[1], Maša Filipović[1], Darja Flegar[1], Ozana Jakšić[1], Tomislav Kelava[1], Nataša Kovačić[2], Alan Šućur[1], Danka Grčević[1]*
*[1]University of Zagreb School of Medicine,*
*Department of Physiology and Immunology- Croatian Institute for Brain Research, Zagreb, Croatia*
*[2]University of Zagreb School of Medicine,*
*Department of Anatomy- Croatian Institute for Brain Research, Zagreb, Croatia*

**Abstract Text**

**Background:** Immune-mediated inflammatory diseases (IMIDs) comprise heterogenous disorders characterized by deregulated immune response, inflammation in target tissues and systemic adverse effects, including changes in bone integrity. IMID-associated immune system malfunctions may directly affect bone cell functions (i.e. in rheumatoid arthritis, RA) or induce systemic deregulation of bone metabolism (i.e. in diabetes mellitus type 1, T1D). However, mechanisms of osteoporosis associated with clinically diverse IMIDs are still not fully explored.

**Purpose:** We aimed to compare T1D-specific and RA-specific subsets of highly potent osteoclast progenitors (OCPs) in circulation and identify specific chemotactic signals for their bone homing and activation.

**Methods:** Upon Ethical approval, peripheral blood samples were obtained from controls as well as RA and T1D patients and subjected to gradient separation to collect peripheral blood mononuclear cells (PBMCs). Immunophenotyping of PBMCs was performed for monocyte markers and chemokine receptors. Sorted OCPs were differentiated in osteoclastogenic medium supplemented with RANKL and M-CSF and analyzed by TRAP staining. Systemic resorptive activity was assessed by serum TRAcP5b ELISA.

**Results:** We combined several monocyte markers to identify peripheral subset of human OCPs within the population of classical monocytes (CD45+CD3-CD19-CD56-CD15-CD11b+CD14+CD16lo), which highly expressed chemokine receptors (CCR2 and CXCR2), suggesting their ability to migrate toward chemokine gradient. This subset was expanded in both RA and T1D (17.3% in RA, 21,2% in T1D vs 9,6% in control), with efficient maturation into multinuclear TRAP+ osteoclasts in vitro and high serum TRAP activity. In addition, significant proportion of OCPs expressed Fc-receptor CD64 and adhesion molecule CD38, with superior differentiation potential compared to the corresponding negative population. Interestingly, OCPs exhibit higher expression of CD64 in RA, while they show increased expression of CD38 in T1D.

**Conclusions:** Identification of distinct OCP subsets may contribute to the understanding of IMID-associated osteoporosis and identify possible therapeutic targets for osteoclast suppression. The work was supported by the project HRZZ-IP-2022-10-2285.

### Poster Sessions: Osteocytes and mechanobiology

**P111**

### Effects of age and of bone mineral content on the osteocyte lacuno-canalicular network in human iliac crest cortical bone

*Chloe Jones[1], Stéphane Blouin[1], Raguin Emeline[2], Richard Weinkamer[2], Andrea Berzlanovich[3], Peter Fratzl[2], Markus A. Hartmann[1]*

*[1]Ludwig Boltzmann Institute of Osteology, Material Science, Vienna, Austria*

*[2]Max Planck Institute of Colloids and Interfaces, Biomaterials, Potsdam, Germany*

*[3]Centre of Forensic Science- Medical University of Vienna, Unit of Forensic Gerontology, Vienna, Austria*

**Abstract Text**

**Introduction:** The osteocyte lacuno-canalicular network (OLCN) houses osteocyte cells within lacunar pores embedded in the mineralised bone matrix, interconnected by a network of canaliculi. The cellular network allows for communication throughout the tissue and orchestrates bone remodelling, essential for the maintenance of mineralised bone. We examined the age-dependent relation between bone mineral content and OLCN structure, using confocal laser scanning microscopy to image rhodamine-stained networks.

**Methods:** We investigated iliac crest autopsy samples from bone-healthy female donors in two age groups: 'middle' (n=6, age 60.1 ± 3.94 years) and 'old' (n=6, age 94.0 ± 0.63 years) (Ethics Committee, Medical University of Vienna: EK 1757/2013). 12 cortical osteons per sample of high (n=6) and low (n=6) mineral content were imaged using quantitative backscattered electron imaging. The rhodamine-stained OLCN was imaged using confocal microscopy to a depth of ~50µm, then analysed in 3D using the Tool for Imaging and Network Analysis (TINA) to calculate the canalicular density (Can.Dn µm/$\mu m^3$). In 25% of osteons, confocal images revealed network defects appearing as patches of stain and engorged canaliculi, which were further investigated in 3D using high resolution focussed ion beam scanning electron microscopy (FIB-SEM).

**Results:** Two-way ANOVA results show no significant age-dependence of the canalicular density, whereas mineralisation is shown to significantly impact Can.Dn (P=0.0235) as well as volume fraction of patches (P=0.002). Pearson correlation analyses revealed Can.Dn to be negatively correlated with average osteon mineral content (wt% Ca, r = -0.3285, P=0.0003). FIB-SEM analyses revealed rhodamine patches to exhibit a higher number of canaliculi with increased diameter.

**Discussion and conclusion:** These results show lower canalicular density in osteons with higher mineral content, but no dependence on donor age. Network defects were found in regions with diffuse rhodamine stain, possibly indicating a defective network where stain is able to escape from the LCN.

**P112**

**A novel 3D perfusion bioreactor model for studying osteocyte mechanobiology in myeloma bone disease**

*Wen-Hui Cheng[1], Wyonna Rindt[1], Melanie Krug[2], Azeem Muhammad[1], Shuntaro Yamada[3], Martin Kuric[2], Ellen Leich[4], Ana Rita Pereira[5], Marietta Hermann[5], Jan Hansmann[6], Camilla Mussoni[7], Philipp Stahlhut[7], Taufiq Ahmad[7], Yassin Mohammed[3], Kamal Mustafa[3], Regina Ebert[8], Franziska Jundt[1]*

*[1]University Hospital Würzburg, Department of Internal Medicine II, Würzburg, Germany*

*[2]Orthopedic Clinic König-Ludwig-Haus- University of Würzburg, Department of Musculoskeletal Tissue Regeneration, Würzburg, Germany*

*[3]University of Bergen, Centre of Translational Oral Research TOR-*

*Tissue Engineering Group- Department of Clinical Dentistry, Bergen, Norway*
[4]*University of Würzburg, Institute of Pathology, Würzburg, Germany*
[5]*University Hospital Würzburg, IZKF Group Tissue Regeneration in Musculoskeletal Diseases, Würzburg, Germany*
[6]*Fraunhofer Institute for Silicate Research ISC, Translational Center Regenerative Therapies, Würzburg, Germany*
[7]*Institute of Functional Materials and Biofabrication IFB- and Bavarian Polymer Institute BPI- University of Würzburg, Department for Functional Materials in Medicine and Dentistry, Würzburg, Germany*
[8]*Orthopedic Clinic König-Ludwig-Haus- University of Würzburg- Würzburg, Department of Musculoskeletal Tissue Regeneration, Würzburg, Germany*

**Abstract Text**

**Background/Introduction:** Malignant plasma cells (myeloma cells) in the bone marrow cause osteolytic bone disease, also known as myeloma bone disease (MBD). They disrupt normal bone remodeling, leading to increased osteoclast activity and reduced osteoblast function, and interact with osteocytes, stimulating tumor progression and bone destruction.

**Purpose:** To study mechanobiology of osteocytes in MBD, we established a biomimetic three-dimensional (3D) bone model applying perfusion and cyclic compression that mimics fluid shear stress and substrate strain in a bone-like environment.

**Methods:** Poly(L-lactide-co-trimethylene carbonate) (LTMC) was used for fabricating porous scaffolds in which the mouse-derived osteocytic IDG-SW3 cells were seeded and differentiated into mature osteocytes. We evaluated the proliferation and differentiation properties of the osteocytes under physiological fluid flow for 21 days and introduced conditioned medium of murine myeloma cells (MOPC315.BM cells) to mature osteocytes. The mechanoresponse was assessed with FBJ osteosarcoma oncogene (Fos) and prostaglandin-endoperoxide synthase 2 (Ptgs2) under perfusion and dynamic compressive loading at 1 Hz with 5 % strain.

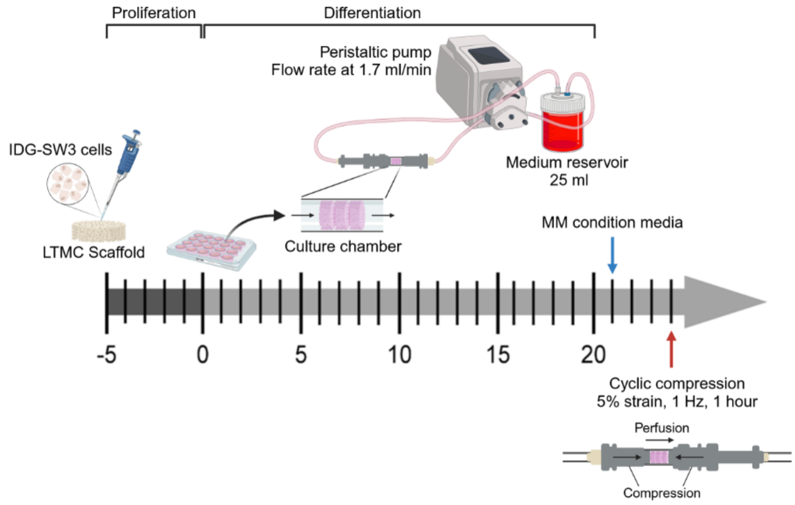

**Results:** Homogeneous distribution and high cell density of IDG-SW3 cells could be observed on the LTMC scaffolds. The osteocyte differentiation marker alkaline phosphatase (Alpl), dentin matrix acidic phosphoprotein 1 (Dmp1), and sclerostin (Sost) were significantly upregulated ($p \leq 0.05$) after 7 days under fluid flow conditions at 1.7 ml/min, indicating rapid and efficient osteocyte maturation.

Quantitative PCR revealed expression changes of cytokines and extracellular matrix proteins in osteocytes incubated with fresh and myeloma-conditioned media. In addition, compressive loading of osteocytes regulated expression of these genes over time, indicating that biophysical stimuli alter bone turnover markers in osteocytes in this model.

**Conclusion(s):** Together, we successfully developed a 3D biomimetic platform that recapitulates *in vivo* mechanical cues, such as fluid flow within the lacuno-canalicular network, to evaluate osteocyte mechanobiology in the content of myeloma bone disease *in vitro*.

**P113**

**In silico micro-multiphysics agent-based modeling: linking mechanoregulation and cytokine dynamics in a mouse model of osteoporosis**

*Friederike Schulte[1], Laurin Schaller[1], Jack J. Kendall[1], Céline Sonnenschein[1], Nuria Balbás[1], Neashan Mathavan[1], Gisela Kuhn[1], Ralph Müller[1]*
*[1]Institute for Biomechanics, ETH Zurich, Zurich, Switzerland*

**Abstract Text**

**Introduction:** Bone tissue adapts to mechanical demands through mechanoregulation, a process mediated by molecular signals that govern bone formation and resorption. Key regulators expressed by osteocytes are the receptor activator of nuclear factor kappa-B ligand (RANKL), which promotes bone resorption, and sclerostin, an inhibitor of formation. In postmenopausal osteoporosis, estrogen deficiency disrupts bone remodeling, but the interplay with mechanical signals, RANKL, and sclerostin remains poorly understood. *In silico* micro-multiphysics agent-based (micro-MPA) models can provide insights into the mechanoregulation of molecular signals, which may be important when considering combining physical and drug therapies for osteoporosis.

**Purpose:** To investigate the role of mechanical signals in regulating sclerostin and RANKL cytokine concentrations under estrogen depletion and mechanical loading using *in silico* micro-MPA modeling.

**Methods:** Baseline images of micro-computed tomography scans of the sixth caudal vertebra of ovariectomized mice were used as input to a micro-MPA model[1]. Five weeks of estrogen deficiency were simulated by lower estrogen activity, and further four weeks of mechanical (8N) or sham (SHM) loading[2]. Trabecular bone volume fraction (BV/TV) was matched with *in vivo* values (8N/SHM: n=10/8). Strain energy density (SED) in formation, quiescence and resorption volumes was analyzed in relation to sclerostin and RANKL concentrations.

**Results:** BV/TV decreased by 19% following estrogen deficiency for 5 weeks (0.5% deviation from mean experimental values), increased further 2% in the SHM-group, and 23% in the 8N-group (Fig. 1a, $p<0.05$). Sclerostin and RANKL concentrations in resorption and quiescence volumes decreased exponentially with increasing SED in both groups, with low concentrations in formation volumes (Fig. 1b-d).

**Conclusion:** These results indicate that mechanics controls the downregulation of RANKL in estrogen deficiency to avoid bone resorption of highly loaded areas, and downregulation of sclerostin to support subsequent bone formation in these areas. Such findings may inform combination therapies for osteoporosis.

[1]Boaretti et al. 2023, [2]Lambers et al. 2011

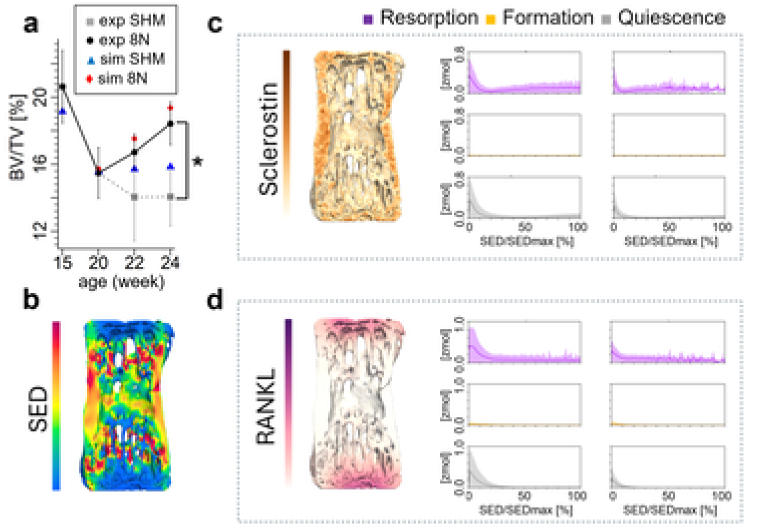

**Fig. 1:** **(a)** *In vivo* and *in silico* bone volume fraction (BV/TV) after estrogen depletion in sham (SHM, n=8) and mechanical loading (8N, n=10) groups, *p<0.05 **(b)** strain energy density (SED), **(c)** sclerostin concentrations per SED%-bin in remodeled areas **(d)** RANKL concentrations per SED%-bin in remodeled areas.

**Poster Sessions: Chondrocytes and cartilage**

**P114**

**Deep Learning-Based Automated Scoring of Osteoarthritis Severity in Mouse Cartilage**

*Kahyon Park[1], Gyuseok Lee[1], jehwang ryu[1]*
*[1]Chonnam National University, School of Dentistry, Gwangju, Korea- South*

**Abstract Text**

**Objective:** This study aims to develop and validate a deep learning model to objectively detect and classify osteoarthritis (OA) symptoms in a mouse model, automating the scoring of histological images based on OARSI criteria.

**Methods:** We acquired Safranin-O stained images of cartilage from mice with OA induced by surgical destabilization of the medial meniscus. For preprocessing, the horizontal rotation of the cartilage was learned using the VGG16 regression model, and partial cartilage incision was learned using YOLO-v7. We applied the learned weight values to the original images to create a Cartilage-Rotation-Crop data set. This data set was then used to train and compare the classification performance of three convolutional neural network models: VGG16, ResNet50, and Xception.

**Results:** Initially, 544 mouse cartilage images were classified into 6 OARSI grades, but the accuracy was significantly low, prompting a sequential error correction strategy. First, the number of specimens was increased to 2788 images and reclassified into more detailed grades. To address the issue of misidentifying non-cartilaginous regions, an algorithm was employed that sequentially that rotates the images to a horizontal position and then crops only the joint part. This approach significantly improved the accuracy of cartilage degradation scoring with our proposed algorithm. Among the various deep learning network models used, the VGG16 model demonstrated the best performance.

**Conclusion:** These results demonstrate that when using our proposed algorithm, deep learning can provide more objective and reliable results for the classification of OARSI grades, underscoring the potential of an automated OA assessment system for mouse cartilage.

**P116**

**Loss of Bmp3 decreases articular cartilage volume in mice**

*Igor Erjavec*[1], *Matej Nekić*[2], *Rumenović Viktorija*[2], *Vukičević Slobodan*[2]
[1]*Center for Translational and Clinical Research- School of Medicine- Zagreb- Croatia, Laboratory for Mineralized Tissue- Department of Anatomy, Zagreb, Croatia*
[2]*Center for Translational and Clinical Research- School of Medicine- Zagreb- Croatia, Laboratory for Mineralized Tissue, Zagreb, Croatia*

**Abstract Text**

**Introduction:** BMP3 is a member of the bone morphogenetic family of proteins with antagonistic actions towards other BMPs. The effect of BMP3 on endochondral bone and cartilage formation have not been sufficiently explored.

**Purpose:** We sought to explore the effect of Bmp3 on articular cartilage and investigate potential use of microCT in cartilage tissue research.

**Methods**: In the study C57BL/6Ntac mice of both sexes were used, where *LacZ* gene was inserted into the first exon the *Bmp3 gene*, rendering it non-functional (Bmp3$^{-/-}$ mice). Femur was cleaned of muscle tissue and fixed in 70% ethanol for 2 days after which it was stained for 3 days in 1% phosphotungstic acid (PTA). Stained bones were scanned using 1076 microCT and cartilage was manually delineated from subchondral bone. After scanning, the bones were decalcified, paraffin embedded and cut to 5 µm slices. The slices were stained using H&E staining for qualitative cartilage assessment.

**Results:** Using PTA for tissue contrasting enabled the visualization of articular cartilage by microCT. As evident on images, the thickness of cartilage was lower in Bmp3$^{-/-}$ mice, while the analysis of cartilage volume showed significant decrease in cartilage volume (CV) in Bmp3$^{-/-}$ mice. Qualitative visualization by histology and H&E staining corroborated the findings and further solidified the use of contrast-enhanced microCT for cartilage tissue research.

**Conclusion:** The lack of Bmp3 causes decrease in articular cartilage thickness and volume, as Bmp3-/- mice had less femoral articular cartilage on both condyles. MicroCT can be used for cartilage research, especially for volumetric analyses, as it enables the quantification of the whole cartilage.

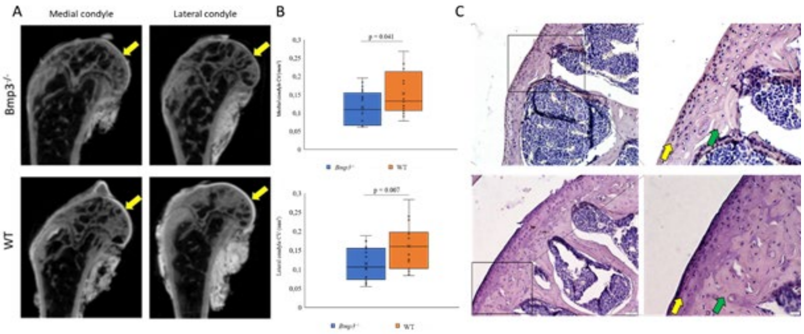

Figure 1. Analysis of articular cartilage in Bmp3$^{-/-}$ and WT mice. MicroCT visualization of contrasted cartilage (A). Cartilage volume on the femoral medial and lateral condyle in WT and Bmp3$^{-/-}$ mice (B). H&E staining of the cartilage at 10x and 40x magnification (C).

**P117**

**Immunobased profiling of knee osteoarthritis patients to predict response to regenerative treatment with autologous platelet-rich plasma injection**

*Valentina Granata*[1], *Antonio Tonutti*[2], *Veronica Marrella*[3], *Rita Ragusa*[2], *Chiara Camisaschi*[4], *Simone Puccio*[5], *Emanuela Morenghi*[6], *Marzia Monferini*[2], *Di Matteo Berardo*[7], *Carlo Selmi*[2], *Cristina Sobacchi*[1], *Angela Ceribelli*[2]

*[1]IRCCS Humanitas Research Hospital,*
*Bone Metabolism Unit- Laboratory of Translational Endocrinology and Metabolism, Rozzano, Italy*
*[2]IRCCS Humanitas Research Hospital, Rheumatology and Clinical Immunology, Rozzano, Italy*
*[3]IRCCS Humanitas Research Hospital, Immunity and Immunodeficiency Lab, Rozzano, Italy*
*[4]IRCCS Humanitas Research Hospital, Flow Cytometry Core, Rozzano, Italy*
*[5]IRCCS Humanitas Research Hospital, Laboratory of Translational Immunology, Rozzano, Italy*
*[6]IRCCS Humanitas Research Hospital, Biostatistics Unit, Rozzano, Italy*
*[7]IRCCS Humanitas Research Hospital, Division of Orthopedics, Rozzano, Italy*

**Abstract Text**

**Background**: Osteoarthritis (OA) is a degenerative musculoskeletal disease causing chronic disability and elevated social costs worldwide. Its multifactorial origin is responsible for the existence of different OA phenotypes and at present no biomarker can predict individual response to treatments such as autologous platelet-rich plasma (PRP) injection[1].

**Purpose**: To define the peripheral blood profile of knee OA patients before PRP injection able to predict the response to this regenerative treatment, based on the analysis of serological and cellular biomarkers.

**Methods**: patients: men/women, aged >40 - <75 years; knee OA grade 2-3, BMI <40. Analyses on peripheral blood samples prior to PRP injection (nSTRIDE): multiparametric FACS analysis of the main adaptive and innate immune cell populations (CD3, CD4, CD8, Treg, CD19, CD14, CD56; central and effector memory cells), followed by unsupervised cluster analysis[2]; expression (qPCR, ELISA) and functional analysis (enzymatic assays) of inflammatory (Wnt and IL-1 pathway; PTX3) and antioxidant (DPP3/Keap1/Nrf2) pathways, selected based on the literature[1]. Patient follow-up until 12 months after treatment. Response to treatment defined based on WOMAC questionnaires and established clinical criteria. Statistical analysis: Mann Whitney test.

**Results**: We enrolled 86 OA patients (30 male, 56 female); 54 patients (45 responders and 9 non-responders) were eligible for the mid-term analysis. The two subgroups did not differ as for age, sex, duration of symptoms prior to enrollment, previous conservative therapy, or surgery. Among the measured parameters, responders had higher serum ascorbic acid and 4-HNE ($p<0.01$); higher expression of *IL1R1, TNFa* and *IL6* ($p<0.05$), *Myd88* and *RANKL* ($p<0.01$) and *Sfrp3* and *IL1R2* ($p=0.06$); lower $CD8^{+}$ cells, which were mainly naïve ($p<0.05$). Unsupervised cluster analysis is ongoing.

**Conclusions**: Mid-term analysis suggested that in our cohort early response to PRP treatment was associated with features of higher oxinflammation before treatment. The study will be completed when all patients reach the 12-month follow-up.

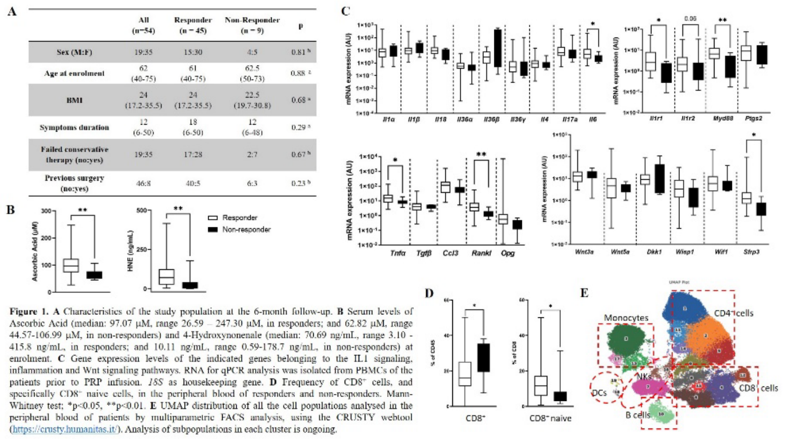

|  | All (n=54) | Responder (n = 45) | Non-Responder (n = 9) | p |
|---|---|---|---|---|
| Sex (M:F) | 19:35 | 15:30 | 4:5 | 0.81 b |
| Age at enrolment | 62 (40-75) | 61 (40-75) | 62.5 (50-73) | 0.88 a |
| BMI | 24 (17.2-35.5) | 24 (17.2-35.5) | 22.5 (19.7-30.8) | 0.68 a |
| Symptoms duration | 12 (6-50) | 18 (6-50) | 12 (6-48) | 0.29 a |
| Failed conservative therapy (no:yes) | 19:35 | 17:28 | 2:7 | 0.67 b |
| Previous surgery (no:yes) | 46:8 | 40:5 | 6:3 | 0.23 b |

**Figure 1. A** Characteristics of the study population at the 6-month follow-up. **B** Serum levels of Ascorbic Acid (median: 97.07 µM, range 26.59 – 247.30 µM, in responders; and 62.82 µM, range 44.57-106.99 µM, in non-responders) and 4-Hydroxynonenale (median: 70.69 ng/mL, range 3.10 - 415.8 ng/mL, in responders; and 10.11 ng/mL, range 0.59-178.7 ng/mL, in non-responders) at enrolment. **C** Gene expression levels of the indicated genes belonging to the IL1 signaling, inflammation and Wnt signaling pathways. RNA for qPCR analysis was isolated from PBMCs of the patients prior to PRP infusion. *18S* as housekeeping gene. **D** Frequency of $CD8^{+}$ cells, and specifically $CD8^{+}$ naive cells, in the peripheral blood of responders and non-responders. Mann-Whitney test; $*p<0.05$, $**p<0.01$. **E** UMAP distribution of all the cell populations analysed in the peripheral blood of patients by multiparametric FACS analysis, using the CRUSTY webtool (https://crusty.humanitas.it/). Analysis of subpopulations in each cluster is ongoing.

### P118

### Deciphering early-onset hip osteoarthritis in COL2A1-related spondyloepiphyseal dysplasia and its corresponding mouse model

*Assil-Ramin Alimy[1], Maximilian Möhlmann[1], Ana Ocokoljic[1], Haoyan Pan[1], Johannes Keller[1], Thorsten Schinke[2], Frank Timo Beil[1], Ralf Oheim[2], Tim Rolvien[1]*
*[1]University Medical Center Hamburg-Eppendorf, Department of Trauma and Orthopaedic Surgery, Hamburg, Germany*
*[2]University Medical Center Hamburg-Eppendorf, Department of Osteology and Biomechanics, Hamburg, Germany*

### Abstract Text

**Background:** Spondyloepiphyseal dysplasia congenita (SEDC) is a rare genetic condition caused by variants in the COL2A1 gene. SEDC is characterized by disproportionate dwarfism, low bone mass, and early-onset osteoarthritis (EOOA). Despite the impact on patients' quality of life, the underlying pathophysiological mechanisms of EOOA in SEDC remain unclear.

**Purpose:** This study aimed to decipher the osteochondral hip phenotype of a patient with SEDC and the corresponding mouse model (*Col2a1*$^{+/d}$).

**Methods:** Bilateral femoral heads were obtained from a patient with SEDC during total hip arthroplasty and compared to femoral heads from primary osteoarthritis (OA) and controls (each n=10). A multi-scale high-resolution approach was applied. Targeted proteomic analysis of synovial fluid and qRT-PCR of the osteochondral unit were performed. Finally, hip joints of *Col2a1*$^{+/d}$ mice at 4, 12, and 52 weeks of age were analyzed and compared with wild-type controls.

**Results:** SEDC samples exhibited reduced subchondral bone mass (BV/TV (%); 16.8 vs. 30.8, p=0.046) and substantial articular cartilage degeneration (Cg.Th (mm); 0.33 vs. 1.7, p=0.003) compared to controls, as well as an increased osteoid volume compared to primary OA and controls (1A, 1B). Furthermore, a tendency towards decreased interleukin-6 and osteoprotegerin was detected in the synovial fluid of the SEDC patient. Interestingly, COL2A1 expression was increased in the osteochondral unit of SEDC, suggesting a compensatory mechanism. The EOOA phenotype was recapitulated in *Col2a1*$^{+/d}$ mice, showing impaired cartilage integrity at 12 and 52 weeks (OARSI score; 2.8 vs. 0.5, p=0.017) and delayed ossification of the femoral head epiphysis at 12 weeks (1C, 1D).

**Conclusion:** Our data illustrate that impaired subchondral bone homeostasis with mineralization defects in the femoral head may contribute to EOOA in SEDC. The *Col2a1*$^{+/d}$ mouse model, which demonstrated delayed ossification of the femoral head, further supports this finding, providing an explanatory basis for the distinct EOOA phenotype observed in patients.

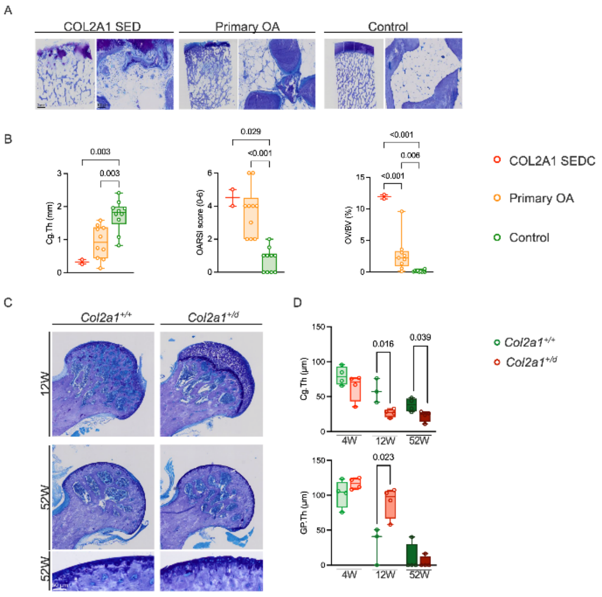

Fig. 1: Unique osteochondral phenotype in a patient with SEDC and *Col2a1*$^{+/d}$ mice.
A) Representative toluidine blue–stained sections of undecalcified femoral heads from a patient with SEDC, primary OA, and controls. B) Histomorphometric quantification of articular cartilage thickness, articular cartilage integrity via the OARSI score, and osteoid volume per bone volume. C) Representative Toluidine blue–stained sections of undecalcified femoral heads from *Col2a1*$^{+/+}$ and *Col2a1*$^{+/d}$ mice at 12 and 52 weeks of age. D) Histomorphometric quantification of articular cartilage thickness and growth plate thickness in *Col2a1*$^{+/+}$ and *Col2a1*$^{+/d}$ mice at 4, 12, and 52 weeks. Abbreviations: SEDC: Spondyloepiphyseal dysplasia congenita, OA: Osteoarthritis.

**Poster Sessions: Endocrine factors**

**P119**

**Endocrine Functions of Bone Are Suppressed by Orally Administered Glucose Regardless of Bodyweight**

*Niki Jalava*[1], *Terhi J. Heino*[1], *Kirsi A. Virtanen*[2], *Pirjo Nuutila*[2], *Kaisa K. Ivaska*[1]
[1]*University of Turku, Institute of Biomedicine, Turku, Finland*
[2]*Turku University Hospital, Turku PET Centre, Turku, Finland*

**Abstract Text**

Bone is recognized as a target for diabetic complications. However, the impact of diabetic conditions on bone metabolism is less understood. Bones participate in systemic energy metabolism by communicating with other organs via secreted factors called osteokines. While it is well established that orally-administered glucose rapidly reduces bone resorption, the effects on osteokines remain unknown. Further, it is not known whether the response of bone to oral glucose is altered in obesity.

In this study, we compared the circulating levels of C-terminal telopeptide of collagen I (β-CTX-I), total osteocalcin (OC), sclerostin, fibroblast growth-factor 23 (FGF23), uncarboxylated OC, and lipocalin 2 (LCN2) at baseline and after a 2-hr oral glucose tolerance test (OGTT, 75g) in volunteers with normal weight (BMI<25kg/m^2, n=22) and obesity (BMI>29kg/m^2, n=27) (age=21–54yr, M/F=11/38).

At baseline, higher uncarboxylated OC/total OC ratio (p=0.012) was found in obesity, possibly due to lower OC levels (p=0.07). OGTT resulted in a significant decrease in bone resorption, median (IQR) decrease in β-CTX-I was 42% (31–53; p<0.0001) in both groups. We observed a modest but statistically significant decrease in the osteokine levels. OC decreased by 11% (8–15; p<0.0001) and FGF23 by 14% (1–25; p=0.007) in both groups, while uncarboxylated OC decreased by 1.2% (0.62–2.0; p<0.0001) and sclerostin by 7.8% (-5.7–15; p=0.032) only in obesity. The differences in responses to OGTT between the groups were not statistically significant. However, we observed a tendency for a less pronounced effect on β-CTX-I levels (p=0.052) and a greater effect on OC levels (p=0.051) in obesity compared to normal weight.

Our study shows that osteokines respond rapidly to oral glucose regardless of bodyweight. Our data suggests that orally-administered glucose affects not only bone turnover but can also suppress bone endocrine functions. Furthermore, obesity may influence the effect of oral glucose on β-CTX-I and OC.

**P120**

**Serum osteocalcin is associated with residual β-cell function in newly diagnosed type 1 diabetes**

*Cecilia Oliveri*[1], *Pietro Furfaro*[1], *Adele Di Giovanni*[1], *Simone Crescimone*[1], *Nunziata Morabito*[1], *Mariella Valenzise*[1], *Antonino Catalano*[1]
[1]*University of Messina, Clinical and Experimental Medicine, Messina, Italy*

**Abstract Text**

**Background/Introduction:** Osteocalcin (OC) is a non-collagenous protein synthetized by osteoblasts that plays a role in bone metabolism. In vitro studies have suggested an association between OC levels, glucose metabolism and β-cell function.

**Purpose:** This study investigated circulating OC levels in children and adolescents at the onset of type 1 diabetes (T1D) and explored their association with glucose control, residual β-cell function, and severity of T1D presentation.

**Methods:** Newly diagnosed T1D patients were recruited at our tertiary-care Diabetes Centre. Anamnestic, anthropometric, clinical and biochemical data at T1D diagnosis were collected. Basal and stimulated C-peptide concentrations were evaluated. Surrogate serum markers of bone remodelling were measured: i.e. osteocalcin (OC), alkaline phosphatase (ALP) and C-terminal telopeptide of type 1 collagen (CTX). Vitamin D status by measuring 25(OH)D and parathormone (PTH) levels were also assessed.

**Results:** 29 individuals with newly diagnosed T1D were recruited with a mean age of 8.4 ± 3.7 yrs. An association between OC and stimulated C-peptide ($r=0.53$; $p=0.02$), and between PTH and serum $HCO_3^-$ ($r=0.544$; $p=0.02$) was found. No other correlations between bone remodelling markers and T1D clinical variables were observed.

**Conclusions:** Our data showed for the first time a positive correlation between OC levels and residual β-cell function in children and adolescents at T1D presentation. Further longitudinal studies focusing on OC levels are needed to improve knowledge regarding the complex interaction between bone and glucose metabolisms in T1D.

**P121**

**Bone remodeling is regulated by the amplitude and timing of glucocorticoid rhythm**

*<u>Annelies Smit</u>[1], Jan Kroon[2], Sander Kooijman[2], Maaike Schilperoort[3], Salwa Afkir[2], Bram van der Eerden[4], Marijke Koedam[4], Onno Meijer[2], Elizabeth Winter[5]*
*[1]Leids Universitair Medisch Centrum, Department of Medicine- Division of Endocrinology & Einthoven Laboratory, Leiden, Netherlands*
*[2]Leiden University Medical Center, Department of Medicine- Division of Endocrinology & Einthoven Laboratory, Leiden, Netherlands*
*[3]Columbia University Irving Medical Center, Department of Medicine, New York, United States*
*[4]Erasmus Medical Center, Department of Internal Medicine, Rotterdam, Netherlands*
*[5]Leiden University Medical Center, Department of Medicine- Division of Endocrinology- Einthoven Laboratory & Center for Bone quality, Leiden, Netherlands*

**Abstract Text**

**Background:** Synthetic glucocorticoid(GC)-use increases fracture risk. Aside from GC excess, the loss of circadian GC rhythmicity may also contribute, as flattening the natural GC rhythm induces osteoporosis in mice. This study explored whether reinstating circadian GC rhythmicity prevents osteoporosis.

**Methods:** We implanted 7.5% corticosterone (CORT) pellets in female C57Bl/6J mice. This blunts the natural circadian CORT rhythm, without elevating CORT exposure. To evaluate the importance of GC troughs and peaks, CORT-pelleted mice received injections with glucocorticoid receptor (GR) antagonist RU486 timed at the natural GC trough (PM) or CORT timed at the natural GC peak (AM) for one week. To evaluate whether timing of the GC trough is important, CORT-pelleted mice received daily AM or PM RU486 injections for 7 weeks. We analyzed plasma CORT and bone formation marker P1NP levels by ELISA, and bone microarchitecture by micro-CT.

**Results:** One week of flattened CORT rhythm reduced plasma P1NP levels, as compared to vehicle (-38% p<0.01). Reinstating a trough in GR signaling rescued P1NP levels (+2% ns), whereas reinstating a GC peak did not (-57% p<0.001). After 7 weeks, RU486-injected mice showed a serum P1NP peak in the morning (AM injection) or evening (PM injection), together suggesting that bone formation is under the control of the GC trough. 7 weeks of flattened CORT rhythm reduced cortical bone thickness (-13% p<0.001) and trabecular bone volume, as compared to vehicle (-32% p<0.05), as shown before (Schilperoort *Aging cell* 2021). RU486 injection at either timepoint rescued cortical thinning (AM -2% ns; PM -1% ns). AM RU486 injections preserved trabecular bone volume (+3% ns), while PM injections did not (-31% p<0.05), demonstrating that timing of the GC trough is relevant.

**Conclusion:** Reinstating a well-timed trough in GR signaling prevents osteoporosis in mice. Thus, maintaining a GC trough in GC treated subjects may be a promising strategy to prevent osteoporosis.

### Poster Sessions: Energy metabolism, fat, diabetes, nutrition

### P122

### Advanced Glycation End Products (AGEs) are not associated with Bone Mineral Density longitudinally: The Rotterdam Study

*Mengyuan Cai[1], Jinluan Chen[1], Jiawei Li[1], Katerina Trajanoska[1], Evert van Velsen[1], Carola Zillikens[1]*
*[1]Erasmus MC, Internal Medicine, Rotterdam, Netherlands*

**Abstract Text**

**Background** Advanced glycation end products (AGEs), formed through non-enzymatic glycation of e.g., proteins in collagen have been associated with prevalent fractures, but their relation with bone mineral density (BMD) and trabecular bone score (TBS) is unclear.

**Purpose** This study aims to assess the association of AGEs measured in skin with longitudinal BMD and TBS.

**Methods** In the Rotterdam Study (RS), skin AGEs were assessed as skin autofluorescence (SAF) using the AGE Reader®. Total body (TB) and femoral neck (FN) and lumbar spine (LS) BMD were assessed using dual-energy X-ray absorptiometry (DXA). SAF was analyzed in relation to baseline and follow-up BMD and TBS, employing a linear mixed effects model with interaction analysis examined for sex, prevalent type 2 diabetes mellitus (T2DM) and chronic kidney disease (CKD).

**Results** Analyses between SAF and TB BMD were performed in 2553 participants (mean age 68 years, 56.2% women, mean follow-up time 4.9 years), and between SAF and LS, FN BMD and TBS in 851 participants (mean age 58.0 years, 55.1% women, mean follow-up 5.6 years). SAF was not associated with BMD nor with changes in BMD nor with TBS. Interactions were observed of SAF with sex and T2DM for the associations with TB and FN BMD. Subgroup analysis revealed no significant associations between SAF and repeated TB BMD stratified by sex or diabetes status.

**Conclusion** We observed no longitudinal association between SAF and BMD at multiple sites or TBS, consistent with our earlier findings that associations of SAF with prevalent fractures were not explained by BMD or TBS. Other aspects of bone quality or muscle characteristics including fall risk may be involved.

**P123**

**Longitudinal changes in body composition in older adults with changes in body mass index: a 5-year follow-up study**

*Yi Yuan[1], Wenshuang Zhang[1], Yandong Liu[1], Ling Wang[1], Xiaoguang Cheng[1]*
*[1]Peking University Fourth School of Clinical Medicine- Beijing Jishuitan Hospital, Department of Radiology, Beijing, China*

**Abstract Text**

**Background:** Multiple changes in body composition occur during ageing, including increased total body fat mass, infiltration of adipose tissue into skeletal muscles, and a shift from subcutaneous adipose area to visceral adipose area.

**Purpose:** This study sought to identify any differences in longitudinal changes in body composition in older adults associated with changes in body mass index (BMI).

**Materials and methods:** 120 participants performed quantitative CT scans of the lumbar spine, hip, and mid-thigh at baseline and after 5 years. Changes in muscle cross-sectional area and muscle density and adipose area were evaluated. Changes in body composition were analyzed according to whether BMI increased or decreased.

**Results:** Most of the parameters for muscle size and muscle density decreased, adipose area increased, but liver fat content decreased in both groups in both sexes. In women, only paraspinals muscle density (baseline: 30.1 HU vs. 33.0 HU, P=0.02; 5-year follow-up: 25.5 HU vs. 29.2 HU, P<0.01) was lower and the percent changes of extensors and abductors muscle density (extensors: -15.0% vs. -9.2%, P<0.01; abductors: -6.9% vs. -2.4%, P<0.01) were greater in the increased BMI group than that in the decreased BMI group. In men, muscle size of psoas major, external rotators, adductors, flexors and abductors were greater (all P<0.01) and the percent changes of psoas major muscle size (the percent changes of psoas major muscle size: -7.3% vs. -13.6%, P<0.01) were lower in the increased

BMI group than that in the decreased BMI group. The percent changes of adipose area showed no differences between two groups in both sexes.

**Conclusion:** The changes of most body composition showed little differences according to the changes in BMI, but muscle density decreased greater in elder with BMI increasing, suggesting that routine monitoring of muscle quality should be taken into account and of value in this population.

**P124**

**Is diabetes-induced bone disease associated with bone microangiopathy? Comparison of bone and vascular damage in type 1 and type 2 diabetes mouse models.**

*Marie Helene Lafage Proust*[1], *Solène Viallon*[1], *Dalia Ibarissen*[1], *Bernard Roche*[1], *Hubert Marotte*[1]
[1]*Inserm U1059, Université J Monnet, Saint-Etienne, France*

**Abstract Text**

Both Type 1 (T1D) and Type 2 (T2D) diabetes increase fracture risk and induce microangiopathy. With vascularization playing a critical role in bone remodeling and despite the complex pathophysiology of diabetic bone fragility, we hypothesized that diabetic bone disease may involve bone vascular damage.

**Methods:** For T1D experiment, 8-week-old male C57Bl/6 mice received saline (CT1, n=24) or streptozotocin (STZ, 180 mg/kg, n=34). For T2D experiment, 4-week-old mice were fed a standard diet (CT2, n=18) or a high-fat diet (HFD, n=26) with STZ injections (60 and 40 mg/kg) 8 and 11 weeks later. Longitudinal bone parameters were assessed at the femur (µCT) at baseline (t0) and 6 months (M6; T1D and T2D) or 12 months (M12; T2D). Blood pressure, bone perfusion (Laser Doppler), tibia histomorphometry, and femoral vascular immunohistochemistry (endomucin, Edm, leptin receptor, LepR) were analyzed. Retinal microangiopathy was evaluated via lectin labeling.

**Results:** In T1D, mice were hyperglycemic (493 vs 184 mg/dL), gained less weight (5% vs 33%), and lost 25% of trabecular and cortical bone by M6 (no loss in CT1). Perfusion declined by 30%, and retinopathy was present. T2D hyperglycemic obese mice showed no trabecular gain at M6 (+25% in CT2), while cortical thickening was similar. By M12, T2D exhibited bone loss at both envelopes, reduced bone perfusion, and retinopathy. Both diabetes decreased bone formation, with bone marrow vessel density unchanged. However, coverage by LepR+ pericytes of bone marrow Edm+ microvessels was elevated in both types of diabetes compared to CT.

Overall, there are functional alterations of bone vessels in T1D with concurrent systemic microangiopathy, eventually observed in DT2 in mice at 12 months. T1D and T2D affected differently in terms of kinetics and amplitude the cortical and trabecular envelopes and decreased bone formation. The diabetes-induced increase in pericytes vascular coverage is in line with previously reported hematopoietic stem cell "mobilopathy".

**P126**

**The Effect of Western Diet on The Transcriptome of Bone Marrow and White Adipose Tissue in Rats**

*Nicko Widjaja[1], Niki Jalava[1], Kaisa K. Ivaska[1]*
*[1]University of Turku, Institute of Biomedicine, Turku, Finland*

**Abstract Text**

Bone marrow adipose tissue (BMAT) expands in metabolic disorders including obesity and diabetes, potentially at the expense of bone formation. BMAT is now recognised as a functional fat depot distinct from extramedullary white adipose tissue (WAT). However, little is known on the effect of energy-rich diet on BMAT at a molecular level. Here we characterised the transcriptome of BMAT and gonadal WAT in rats fed with western diet (WD).

Eight-week-old male Sprague-Dawley rats were fed *ad libitum* either WD (42%kJ from fat, 1.25% cholesterol) or chow diet (18%kJ from fat, 0% cholesterol) for 12 weeks. Adipocyte morphology was analysed from histological sections of gonadal WAT and humeral BMAT. We performed bulk RNA-sequencing analysis of BMAT isolated from tibial and femoral bone marrow and gWAT. Humeral microarchitecture and bone mineral density were measured with microCT.

WD significantly increased body weight (p=0.02), whole-body fat percentage (p=0.009) and trabecular separation (p=0.001), and reduced trabecular number (p=0.002) compared to the chow group. WD significantly increased the median adipocyte size in both gonadal WAT (p=0.023) and proximal humeral BMAT (p=0.041). RNAseq showed that WD was associated with enrichment of inflammation-related pathways in BMAT and fatty acid biosynthesis pathways in gWAT when compared to their respective chow groups. Analysis on the PPAR signalling pathway revealed a different response to WD in BMAT and gWAT. In BMAT, pathways related to fatty acid transport were upregulated by WD. In gWAT, pathways related to lipogenesis were upregulated.

In summary, we observed enrichment in inflammation-related pathways in BMAT in response to WD. Contrastingly in gWAT, the most enriched pathways were related to fatty acid biosynthesis. WD significantly increased the median size of both bone marrow and gonadal adipocytes. Overall, this might suggest that adipocyte expansion may be more related to fatty acid uptake in BMAT and lipogenesis in gWAT.

**P128**

**Indices of low bone turnover in males with type 2 diabetes**

*Jakob Starup-Linde[1], Helena Bardenfleth[2], Ellen Hauge[3], Niklas Rye Jørgensen[4], Torben Harsløf[2], Bente Langdahl[2]*
*[1]Aarhus University Hospital, Department of Endocrinology, Aarhus N, Denmark*
*[2]Aarhus University Hospital, Department of Endocrinology, Aarhus, Denmark*
*[3]Aarhus University Hospital, Department of Rheumatology, Aarhus, Denmark*
*[4]Rigshospitalet, Department of Clinical Biochemistry, Copenhagen, Denmark*

**Abstract Text**

**Background:** Type 2 diabetes (T2D) is associated with an increased risk of fracture and a low level of circulating bone turnover markers.

**Purpose:** To investigate if the low bone turnover in T2D can be demonstrated in bone tissue and blood and bone marrow serum (BMS) when compared to males without T2D.

**Methods:** A cross-sectional study comprising 26 Caucasian males with T2D and 26 age and matched controls without T2D. We collected serum, BMS, and bone tissue and performed whole body and regional dual energy x-ray absorptiometry. Bone tissue samples were obtained from the iliac crest ad modum Jamshidi and were evaluated by histomorphometry. Descriptive statistics were used to analyse the data. We defined low bone formations as PINP < the median P1NP (33.8ug/l) of 50 year old Caucasian men.

**Results:** The males with and without T2D were comparable on all parameters except T2D status and glycated hemoglobin levels. S- b-CTX-I (224 ng/l vs. 315,p=0.02), S-PINP (43.2 ug/l vs. 52.5,p=0.04), S-osteocalcin (15.0 ug/l vs. 18.4,p=0.03). BMS b-CTX-I, BMS-PINP, and BMS-osteocalcin) were significantly lower in the T2D group compared to the controls (p<0.05 for all), but no differences was observed in the histomorphometry-derived parameters. Low bone formation (n=12) was mainly found in men with T2D (p<0.05) and in these men osteoid surface, mineralised surface, mineral apposition rate, and bone formation rate were lower (p<0.05 for all) compared to men with normal bone formation

**Conclusion:** We found that circulating b-CTX-I, PINP, and osteocalcin are significantly lower in males with T2D compared to males without T2D. Surprisingly, the histomorphometry derived parameters were not different between the group, however a subset of participants comprising 1/3 of men with T2D had low bone formation and displayed very low bone turnover based on both bone turnover markers and bone histomorphometry.

**P129**

**Title: A Pilot Study on the Impact of Obesity and Weight loss on Bone Microarchitecture in Chinese Adolescents**

*Joanna Tung[1], Tony Nelson[2], Kexin Sun[2], Dorothy Chan[2], Connie Hui[3], Vivian Hung[4], Tsz-ping Lam[4]*
*[1]Hong Kong Children's Hospital, Paediatrics and Adolescent Medicine, Hong Kong, Hong Kong*
*[2]The Chinese University of Hong Kong, Paediatrics and Adolescent Medicine, Hong Kong, Hong Kong*
*[3]The Polytechnic University of Hong Kong, Food Science and Nutrition, Hong Kong, Hong Kong*
*[4]The Chinese University of Hong Kong, Orthopaedic and Tramatology, Hong Kong, Hong Kong*

**Abstract Text**

**Introduction**: The impact of excess adiposity and weight loss on bone health in adolescents with obesity is controversial, possibly due to the limitations of dual energy x-ray absorptiometry (DXA) in assessing growing children.

**Purpose**: This study aimed to assess the bone microarchitecture of adolescents with obesity at baseline and after a 16-week intensive weight loss program using high-resolution peripheral quantitative computed tomography (HR-pQCT).

**Methods**: This research was a sub-study of a randomized controlled trial (RCT) comparing a ketogenic diet to a lifestyle modification program for adolescents with obesity. Post-pubertal Chinese adolescents aged 14-19 years were recruited, excluding those with type II diabetes or other secondary causes of obesity. Bone microarchitectural parameters were measured at the non-dominant distal radius using HR-pQCT at baseline and after 16 weeks.

**Results**: Forty-four adolescents (median age: 15.0 years, 28 males) completed the baseline assessment. Compared to 44 age- and sex-matched healthy controls, adolescents with obesity exhibited significantly higher total volumetric bone mineral density (vBMD), cortical vBMD, and cortical thickness, while trabecular vBMD showed no significant difference (Table 1).

Thirty-nine participants (64% male, median age: 15.2 years) completed the 16-week RCT, with a significant decrease in median BMI from 31.6 kg/m² to 30.9 kg/m² ($p < 0.001$). When comparing weight loss groups, those losing ≥3% had a lower median increase in total vBMD (2.75 vs. 14.6 mg HA/cm³, $p = 0.028$) but no significant changes in other parameters.

**Conclusion**: This study highlights the complex relationship between obesity, weight loss, and bone microarchitecture in adolescents. While higher total and cortical vBMD were observed at baseline in adolescents with obesity, significant changes emerged after the 16-week intervention, particularly among those achieving greater weight loss. Further research is essential to understand the implications of weight management strategies on bone health in this vulnerable population.

Table 1. Bone microarchitectural parameters between the obesity group vs. healthy controls [value expressed as median (IQR)]

| | Obesity (n=44) | Control (n=44) | p value |
|---|---|---|---|
| **Age (years)** | 15.1 (14.2-16.4) | 15.4 (14.0-16.1) | 0.442 |
| **BMI (kg/m2)** | 31.9 (29.39–35.6) | 20.2 (18.41–23.1) | <0.001 |
| **Total vBMD [mg HA/cm³]** | 380.8 (320.6–418.7) | 301.7 (237.8–351.4) | <0.05 |
| **Trabecular vBMD [mg HA/cm³]** | 164.7 (137.9–187.1) | 159.7 (144.3–177.8) | 0.816 |
| **No. of trabeculae [mm]** | 1.42 (1.30–1.51) | 1.63 (1.49–1.82) | <0.001 |

**P131**

### Extended bile acid annotation identifies novel species associated with bone health in a rat model of high fat diet-induced obesity

*Marco Balasso*[1], *Gunter Almer*[1], *Donatella Semeraro*[1], *Mithra Sophie Gruber*[1], *Romana Penker*[1], *Sabine Pailer*[1], *Hans Jürgen Gruber*[1], *Giovanny Rodriguez-Blanco*[1], *Markus Herrmann*[1], *Nerea Alonso*[1]
[1]*Medical University of Graz, Clinical Institute for Medical and Chemical Laboratory Diagnostics, Graz, Austria*

### Abstract Text

Bile acids (BAs) alteration in high fat diet (HFD)-induced obesity in rodents is associated with bone loss. We aim to better characterize the bile acid pool including newly discovered microbiallyconjugated (MCBAs), sulfated (s-), and glucuronidated (g-) species, and their association to bone health in a HFD-induced obesity rat model. Sprague Dawley female rats (n=20) were fed with HFD or chow control diet for ten months. Bone structure was assessed at study end by microCT and controlled by weight. Untargeted bile acid analysis was performed in serum, stool, urine, and liver by reversed-phase chromatography and Orbitrap mass spectrometry using DDA acquisition. This study was approved by the National Ethics Committee. Total fecal bacteria was assessed by 16S analysis. Mann-Whitney test and Spearman correlation were performed in GraphPad Prism v10. Microbiome-BA correlation was performed in MicrobiomeAnalyst software. Twelve MCBAs were identified in stool; five g-bile acids, and fifteen s-bile acids were detected in serum, urine, and/or liver. Eight MCBA were higher in stool of HFD animals compared with controls (fold change>2.2, $p_{range}$=0.049-0.0006), and were negatively correlated with cortical thickness ($r_{range}$=-0.541- -0.767, $p_{range}$=0.03-0.0008). Levels of g-BAs were 10 times lower in serum of HFD animals compared to controls (p=0.0003). g-BAs positively correlated with cortical bone ($r_{range}$=0.697-0.823, $p_{range}$=0.0006-8.4x$10^{-6}$). Five s-BAs were reduced in serum of HFD animals compared to controls ($range_{reduction}$=81.7-97.4%, $p_{range}$=0.001-0.0003). s-BAs positively correlated with structural parameters of cortical bone ($r_{range}$=0.677-0.821, $p_{range}$=0.0007-9.14x$10^{-6}$). Similar results were found for urine and liver. Microbiome analysis showed a link between BAs and musculoskeletal-associated bacteria, namely Arg-CDCA with *Alistipes* (r=0.595, p=0.01), s-BAs with *Turicibacter* ($r_{range}$=0.770-0.828, p=0.01) and g-BAs with *Eubacterium fissicatena* and *Colidextribacter* ($r_{range}$=0.680-0.733, p=0.01). Our results suggest that recently described BAs, like MCBAs, could play a role in maintaining cortical bone health. Besides, obesity-driven alterations could cause dysregulation of BA detoxification mechanisms coupled with bone-protective microbiome dysbiosis.

**P132**

**Increased bone inflammation in type 2 diabetes and obesity correlates with Wnt signaling downregulation and reduced bone strength**

*Giulia Leanza[1], Malak Faraj[1], Viola Viola[1], Flavia Tramontana[1], Niccolò Pellegrini[1], Rocky Strollo[2], Gianluca Vadalà[3], Roberto Civitelli[4], Rocco Papalia[3], Nicola Napoli[1]*
*[1]Campus Bio-Medico University, Unit of Endocrinology and Diabetes, Roma, Italy*
*[2]San Raffaele Open University,*
*Department for the Promotion of the Human Science and Quality of Life, Roma, Italy*
*[3]Fondazione Policlinico Campus Bio-Medico of Rome, Orthopedic surgery, Roma, Italy*
*[4]Washington University in St Louis, Bone and Mineral Diseases, St Louis, United States*

**Abstract Text**

Type 2 diabetes (T2D) and obesity (OB) are associated with chronic inflammation and increased fracture risk. In vitro studies showed that inflammation inhibits bone formation by increasing Wnt canonical pathway inhibitors. However, the impact of inflammation on Wnt pathway regulation and bone quality in T2D and OB remains unclear. We studied 63 postmenopausal women undergoing hip replacement for osteoarthritis. Among these women, 19 had T2D and OB (HbA1c 6.8±0.79%; BMI 29.9±5.2 kg/m2), 17 had OB but they were normoglycemic (BMI 32.5±5.4 kg/m2), and 27 served as controls (BMI 23.1±5.5 kg/m2). Serum inflammatory cytokines revealed higher TNF-α (p=0.0084) and lower adiponectin (p=0.0402) in T2D, and higher IL-6 (p=0.0003) levels in OB vs controls. Gene expression analysis of trabecular bone showed increased TNF-α (p=0.0019) and SFRP5 (p=0.0084) in T2D vs controls. IL-10 was lower in T2D (p=0.0285) and OB (p=0.0324), while adiponectin (ADIPOQ) was lower in T2D (p=0.0041) vs controls. Interestingly, the Wnt inhibitor SOST was higher in T2D (p<0.0001) and OB (p<0.0001). Conversely, WNT10B mRNA levels were lower in T2D (p=0.0071) and in OB (p=0.0196), while LEF-1 were lower in T2D (p=0.0009) vs controls. WNT5A (p=0.0025) and GSK3β (p=0.0003) mRNA levels were higher T2D vs controls. Importantly, TNF-α mRNA levels positively correlated with SOST (r=0.5121, p=0.0002), WNT5A (r=0.3227, p=0.0396) and GSK3β (r=0.3789, p=0.0146) mRNA levels, but negatively correlated with WNT10B (r=0.3844, p=0.0188) and LEF-1(r=-0.3310, p=0.0322) mRNA levels. Conversely, IL-10 was negatively correlated with SOST mRNA levels (r=0.3100, p=0.0457). ADIPOQ was negatively correlated with SOST (r=-0.3864, p=0.0105) and WNT5A (r=-0.3025, p=0.0515) mRNA levels. Finally, serum levels of TNF-α (r=-0.3473, p=0.0352) and IL-6 (r=-0.3777, p=0.0302) negatively correlated with Young's Modulus. These findings suggest that increased inflammation in bone of subjects with T2D and obesity is negatively associated with Wnt pathway and bone strength, shedding light on pathophysiology of bone impairment in metabolic diseases.

**P133**

**High glucose and palmitic acid affect osteoblast mineralization by modulating mitochondria function and ROS production via the DPP3/Nrf2 pathway**

*Giuliana La Rosa*[1,2]*, Concetta Sozio*[1,2]*, Carmela Onda*[1,2]*, Fabiano Cimmino*[1,3]*, Rita De Mare*[2]*,*
*Cristina Sobacchi*[4]*, Maria Pina Mollica*[3]*, Mariarosaria Santillo*[1,2]*, Sabrina Diano*[2,5]*, Ciro Menale*[2]
[1]*A.O.U. Policlinico "Federico II", Public Health- Pharmacoutilization and Dermatology- UOC Hygiene, Naples, Italy*
[2]*University of Naples Federico II, Clinical Medicine and Surgery, Naples, Italy*
[3]*University of Naples Federico II, Biology, Naples, Italy*
[4]*IRCCS Humanitas Research Hospital,*
*Bone Metabolism Unit- Laboratory of Translational Endocrinology and Metabolism, Milano, Italy*
[5]*Columbia University- Irving Medical Center, Institute of Human Nutrition, New York, United States*

**Abstract Text**

**BACKGROUND:** High glucose (HG) and high lipids affect osteoblast (OB) function and metabolism inducing cellular dysfunction. Mitochondria can sense fuel availability to regulate cellular adaptation. The Dipeptidyl peptidase-3 (DPP3) is involved in bone metabolism regulating the antioxidant-Nrf2 pathway and can play a role in OB dysfunction under nutrient excess.

**PURPOSE:** To study the effects of HG and high palmitic acid (PA) in OB and the role of the DPP3/Nrf2 axis.

**METHODS**: MC3T3-E1 cells treated with normal glucose (NG, 5.5mM), HG (25mM) and HG+PA (100µM) were used to evaluate mineralization, mitochondria dynamics and ROS production by fluorescence. Molecular analysis was conducted by IF, WB and qPCR; oxygen consumption rate (OCR)

was assessed by Seahorse.

**RESULTS**: We found that HG and HG+PA treatments decreased mineralization in OB compared to NG (HG, -1.7-fold; HG+PA, -2.0-fold; p<0.001), supported by a reduction of osteogenic markers. Compared to NG, HG and HG+PA exposure reduced mitochondria density (-3.0 and -4.0-fold, respectively; p<0.01) and coverage (-1.2 and -1.9-fold, respectively; p<0.001), with mitochondria appearing more rounded in shape, suggesting the induction of a fission process (mito.n/area: NG, 0.36; HG, 0.79; HG+PA, 0.75; p<0.01 vs NG). The fission-regulator DRP1 protein expression was modulated supporting fission processes. Maximal mitochondrial respiration was elevated in HG and HG+PA conditions (OCR pmol/min/cell n: NG, 109; HG, 152; HG+PA 190; p<0.01 vs NG), and mRNA level of lipid utilization molecules was increased in HG+PA vs NG (*Cpt1α*: 1.8-fold; *Cpt2*: 9.0-fold; *Acadl*: 2.0-fold; p<0.01). ROS production was augmented by 2.0-fold (p<0.001) in HG and by 3.7-fold (p<0.01) in HG+PA leading to oxidative stress. DPP3 and Nrf2 pathway-downstream molecules were reduced in HG and even further in HG+PA treatments (HG+PA-fold density: DPP3, -2.5; HO-1, -6.6; SOD1, -2.8; p<0.05 vs NG).

**CONCLUSIONS**: OB function is affected by nutrient-induced oxidative stress and mitochondria imbalance impairing DPP3/Nrf2 antioxidant pathway.

**P134**

**Novel Dietary Regimen to Study Diabetic Osteopathy Induced by a High-Fat Diet**

*<u>Maria Lucia Schiavone</u>[1], Marta Filibian[2], Roberta Gioa[2], Anda Mihaela Naciu[3], Giovanna Trinchese[4], Gaia Tabacco[3], Ciro Menale[5], Andrea Palermo[3]*
*[1]IRCCS Humanitas, Endocr Cell Molec, Milano, Italy*
*[2]University of Pavia, Magnetic Resonance Imaging and X-ray Micro Tomography Laboratory- Center for Advanced Equipment, Pavia, Italy*
*[3]Campus Bio-Medico University of Rome, Unit of Endocrinology and Diabetes- Department of Medicine, Rome, Italy*
*[4]University of Naples "Federico II", Department of Biology, Naples, Italy*
*[5]University of Naples "Federico II", Clinical Medicine and Surgery, Naples, Italy*

**Abstract Text**

**Background**: Diabetes represent an important public health issue and the specific influence on bone health is often underestimated(1).

**Aim**: We aim to establish a murine model of type 2 diabetes (T2D) induced-osteopathy through a customized high-fat diet (HFD) 45% where fat is mainly of plant origin, at variance with the most common Western diet; the same was for the control diet (CD, 10% kcal). Moreover, we aim to investigate the mechanism of 'oxinflammation' in relation to bone fragility, focusing on DPP3 (Dipeptidyl peptidase 3) as a potential key factor(2,3).

**Methods**: 3-month-old Wild Type (WT) and DPP3 knockout (KO) animals (animal experimentation protocol n.7/23) ) were divided based on sex and randomized prior to the 12-week treatment. Glucose tolerance test (GTT) was performed at baseline, at six and twelve weeks, following standard protocols. Analyses at necropsy: serological evaluations (particularly, DPP3 activity), histological and immunohistochemical analyses (Nrf2, 4HNE) on decalcified bone, densitometric and biomechanical evaluations.

**Results**: We designed a diet for our purpose (Fig.1A). In male mice, HFD administration caused significant changes in weight (WT CD vs. WT HFD, p<0.0001; WT HFD vs. KO HFD, p<0.0001, Two-way ANOVA; Fig.1B) and glucose tolerance (T120 WT CD vs. WT HFD, p=0.0119; KO CD vs. KO HFD, p=0.0181, two way Anova; Fig.1C) after twelve weeks and decreased DPP3 enzyme activity (WT CTR vs. WT HFD, p = 0.0040, Mann-Whitney; Fig.1D). Immunohistochemical analysis for 4HNE and Nrf2 suggested increase in WT e KO males post-diet (Fig.1E). No differences in glucose metabolism were found in female mice (Fig.1F); however, modulation of DPP3 activity was observed (Fig.1H).

**Conclusion**: The selected diet induces changes in glucose metabolism in male WT and KO animals, alongside modulation of DPP3 and responses to oxidative stress. Preliminary evaluation on females exhibits a milder phenotype. The study is ongoing to perform all the investigations planned.

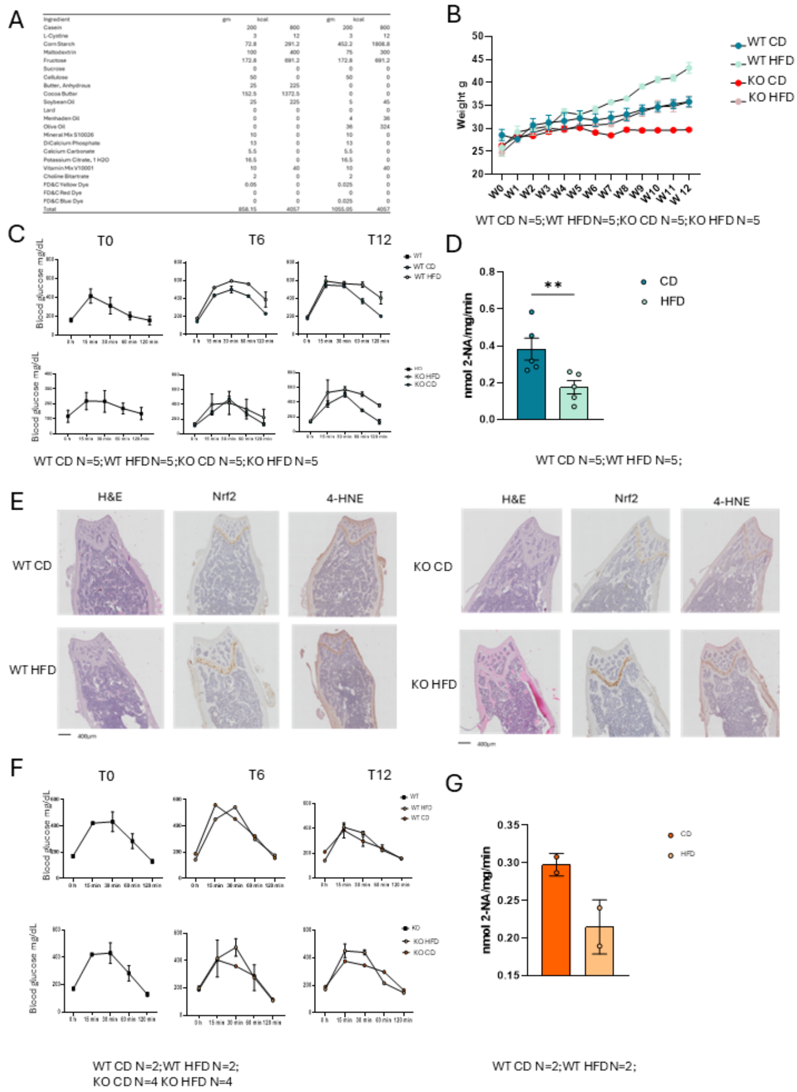

.

**P135**

### Cardiomyocyte-Specific Deletion of Fibroblast Growth Factor 23 in a Left Ventricular Hypertrophy Model Uncovers Its Critical Role in Cardiac Energy Metabolism

*<u>Arezou Lari</u>[1], Nejla Latic[2], Na Sun[3], Achim Buck[3], Ana Zupcic[2], Martin Hofer[4], Wenhan Chang[5], Axel K. Walch[3], Alexander Grabner[6], Reinhold G. Erben[1]*

*[1]Ludwig Boltzmann Institute of Osteology, Ludwig Boltzmann Institute of Osteology, Vienna, Austria*

*[2]University of Veterinary Medicine, Department of Biomedical Sciences, Vienna, Austria*

*[3]German Research Center for Health and Environment, Research Unit Analytical Pathology, Neuherberg, Germany*

*[4]University of Veterinary Medicine, Genomics Core Facility- VetCore-, Vienna, Austria*

*[5]University of California, San Francisco VA Medical Center- Department of Medicine, San Francisco, United States*

[6]Duke University Medical Center, Department of Medicine, Durham, United States

**Abstract Text**

Left ventricular hypertrophy (LVH) is associated with increased cardiac expression of fibroblast growth factor (FGF23) in mice and men. To further elucidate the role of cardiac FGF23 in LVH, we specifically ablated Fgf23 in cardiomyocytes, and employed transverse aortic constriction (TAC) to induce LVH by pressure overload. The TAC-induced increase in cardiac *Fgf23* transcription was blunted in cardiomyocyte-specific Fgf23 knock-out (Fgf23CKO) mice. LVH developed independently of cardiac Fgf23, but Fgf23CKO TAC mice were characterized by ameliorated hypertension, more pronounced eccentric hypertrophy, and a distinct reduction of cardiac fibrosis, relative to $Fgf23^{fl/fl}$ TAC controls. Spatial metabolomics in heart cryosections of Fgf23CKO and $Fgf23^{fl/fl}$ TAC mice revealed that lack of local Fgf23 secretion reduced intracellular glucose abundance and lowered cardiac energy charge. In agreement with these findings, spatial transcriptomics showed a downregulation of GLUT1 and GLUT4, key glucose transporters, as well as of hexokinase, the enzyme responsible for converting glucose to glucose-6-phosphate.
Notably, lack of cardiac FGF23 resulted in the upregulation of AMPK, SIRT1, and PGC1α gene expression in hearts of Fgf23CKO TAC mice, mimicking the cardioprotective mechanisms seen with sodium glucose transporter-2 (SGLT2) inhibitors in humans. These findings suggest that lack of cardiac FGF23 signaling promotes cardiac health by improving energy balance through AMPK activation, reducing oxidative stress via SIRT1, and enhancing mitochondrial biogenesis via PGC1α.
Our findings highlight FGF23 as a critical regulator of glucose and energy metabolism in the hypertrophic heart and may have important therapeutic implications for conditions such as LVH and heart failure.

**P136**

**Visceral and bone marrow adipose tissues impair bone quality in pre- and postmenopausal women and men with obesity: utility of DXA and MRI**

*Vit Zikan[1], Ivan Jr Raška[2], Andrej Bocán[3], Monika Wágnerová[3], Dana Michalská[2], Radka Taxová Braunerová[4], Pavla Kalousková[4], Michaela Ferenčáková[5], Michaela Tencerova[5], Lukáš Lambert[6]*
[1]*General University Hospital and Faculty of Medicine 1- Charles University, Internal Medicine 3, Prague, Czech Republic*
[2]*General University Hospital- Faculty of Medicine 1- Charles University, Internal Medicine 3, Prague, Czech Republic*
[3]*General University Hospital- Faculty of Medicine 1- Charles University, Department of Radiology, Prague, Czech Republic*
[4]*Institute of Endocrinology, Obesity Management Center, Prague, Czech Republic*
[5]*Institute of Physiology of the Czech Academy of Sciences, Laboratory of Molecular Physiology of Bone, Prague, Czech Republic*
[6]*University Hospital Motol and Faculty of Medicine 2- Charles University, Department of Radiology, Prague, Czech Republic*

**Abstract Text**

Obesity, namely visceral adiposity (VAT) associated with insulin resistance (IR) is responsible for other metabolic comorbidities including fractures. Besides VAT, also bone marrow adipose tissue (BMAT) is associated with poor bone quality and higher risk of vertebral fractures. The present cross-sectional study was designed to evaluate the validity of dual X-ray absorptiometry (DXA) compared with magnetic resonance imaging (MRI) for estimating VAT in pre- and postmenopausal women and men with obesity and controls (N= 66). Moreover, relationships between bone mineral density (BMD) and trabecular bone score (TBS) and adipose indices [VAT, BMAT, Liver Fat Fraction (LFF) and paraspinal muscle fat fraction (PSMFF)], as well as HOMA-IR and plasma osteocalcin were assessed. Correlation between MRI and DXA measured VAT was 0.90 ($p < 0.0001$). The obese groups showed significantly higher HOMA-IR and lower circulatory levels of osteocalcin ($p<0.05$). Considering all subjects, osteocalcin levels negatively associated with VAT and LFF ($p<0.05$). HOMA-IR positively associated with VAT ($R^2$=0.32 and 0.33, MRI and DXA, resp., $p<0.001$) and LFF ($R^2$=0.2; $p < 0.01$). TBS was positively associated with LS BMD ($R^2$ =0.325, $p<0.001$) and negatively associated with VAT ($R^2$ = 0.13 and 0.18; $p < 0.01$), BMAT ($R^2$ = 0.26; $p<0.001$) and PSMFF ($R^2$ = 0.40; $p<0.001$). BMAT was positively related with VAT ($R^2$ = 0.11; $p<0.01$). Taken together, our data showed that VAT measurement by DXA are well-correlated with MRI in subjects with obesity. IR and AT dysfunction, including high VAT and BMAT, may be active players in bone deterioration, as suggested by lower TBS in obese subjects. However, further studies are needed to investigate the underlying mechanisms.

This study was supported by the MH CZ (NU23-01-00125) and by the MH CZ—DRO (General University Hospital in Prague—00064165) and Cooperation Program, research area "Metabolic Diseases" and RVO: 86652036 and EFSD/NovoNordisk Foundation Future Leaders Award (NNF20SA0066174)

**P137**

**Increased frequency of osteoclast progenitors and enhanced osteoclast activity in streptozotocin induced type 1 diabetes mouse model**

*Marta Radošević[1], Danka Grčević[1], Ivo Kalajzic[2], Nataša Kovačić[3], Tomislav Kelava[1], Sanja Novak[4], Alan Šućur[1], Darja Flegar[1], Maša Filipović[1], Sara Aničić[1], Pavao Planinić[5], Ivo Krešić[5], Ozana Jakšić[1]*
*[1]University of Zagreb School of Medicine, Department of Physiology and Immunology, Zagreb, Croatia*
*[2]UConn Health, Center for Regenerative Medicine and Skeletal Development, Conneticut, United States*
*[3]University of Zagreb School of Medicine, Department of Anatomy, Zagreb, Croatia*
*[4]University of Arkansas for Medical Sciences, Department of Physiology and Cell Biology, Little Rocks, United States*
*[5]University of Mostar School of Medicine, Department of Physiology, Mostar, Bosnia - Herzegovina*

**Abstract Text**

**Background:** Type 1 diabetes mellitus (T1D) arises from the autoimmune destruction of insulin-producing pancreatic beta-cells, resulting in chronic hyperglycaemia and widespread organ complications. Although T1D has been associated with bone loss, mechanisms underlying increased osteoclast-driven bone resorption remain unclear. Recent studies associate inflammatory cytokines, chemokines and advanced glycation end-products with increased osteoclast activity.

**Purpose:** Our study aimed to investigate osteoclast progenitor (OCP) populations and their bone-resorbing potential in the context of T1D.

**Methods:** Upon Ethical approval, T1D was induced by streptozotocin administration (STZ, 50 mg/kg for 5 days) to male C57BL/6 mice (9-11 weeks old). Bone marrow (BM) and spleen (SPL) phenotyping was performed using flow-cytometry. Sorted OCPs were stimulated with osteoclastogenic factors (M-CSF/RANKL) to promote in vitro differentiation of tartrate-resistant acid phosphatase (TRAP)-expressing osteoclasts. Bone resorption was assessed by micro-CT and serum markers (TRAP, CTX-I) 3 weeks and 2 months following STZ administration compared to controls (CTRL).

**Results:** STZ-treated mice exhibited severe hyperglycaemia (T1D 16.0 (10.0-21.6) mmol/L vs CTRL 8.7 (7.6-9.6) mmol/L, $p<0.05$) and weight loss. In parallel, T1D mice showed an increased frequency of peripheral (SPL, $CD45^{+}CD3^{-}B220^{-}NK1.1^{-}Ly6G^{-}CD11b^{+}CD115^{+}$) and medullar (BM, $CD45^{+}CD3^{-}B220^{-}NK1.1^{-}Ly6G^{-}CD11b^{lo}CD115^{+}$) OCPs, with enhanced expression of chemokine receptors CCR2 and CX3CR1. Sorted OCPs from T1D mice generated higher number of multinuclear $TRAP^{+}$ osteoclasts in vitro. Finally, 2 months following STZ administration T1D mice showed significantly increased serum TRAP5b levels (T1D 37.4 (32.9-55.4) U/L vs CTRL 29.9 (27.2-39.7) U/L, $p<0.05$) and slightly reduced femoral cortical bone volume (BV/TV T1D 49.7 (48.4-50.9)% vs CTRL 51.1 (50.8-51.5)%, $p<0.05$), with no difference in serum CTX-I levels.

**Conclusions:** Our findings suggest expansion of OCPs in T1D, with the enhanced differentiation capacity. Future experiments will focus on understanding the mechanisms driving OCP stimulation and their increased recruitment to bone surfaces. The work was supported by projects HRZZ-IP-2022-10-2285 and DOK-NPOO-2023-10-4220.

**ND-P006**

**Bone Biopsy Microstructure and In Vitro Metabolic Profile of Bone Cells are Preserved in a Cohort of Adults with Well-Controlled Childhood-Onset Type 1 Diabetes**

*Sebastian Zanner[1,2], Maibritt Egeskov Tornbjerg[1,2], Inge Gerlach Brandt[3], Charlotte Ejersted[4], Paula Fernandez-Guerra[1,2], Moustapha Kassem[1,2], Eva Maria Wölfel[1,5], Morten Frost[1,2]*
*[1]Department of Endocrinology, Odense University Hospital, Odense, Denmark*
*[2]Molecular Endocrinology Unit, Clinical Research- University of Southern Denmark, Odense, Denmark*
*[3]Steno Diabetes Center Nordjylland, Steno Diabetes Center Nordjylland, Aalborg, Denmark*
*[4]Steno Diabetes Center Odense, Steno Diabetes Center Odense, Odense, Denmark*
*[5]Molecular Endocrinology Unit, Clinical Research- University Southern of Denmark, Odense, Denmark*

**Abstract Text**

**Introduction:** Patients with type 1 diabetes (T1D) have a lower BMD and an elevated fracture risk. Additionally, bone turnover is lower in T1D. The underlying mechanisms causing these abnormalities remain to be explored.

**Purpose:** We hypothesized that impairments in the metabolic program, including lower glycolytic rate and mitochondrial respiration, of bone cells from T1D patients contribute to the skeletal phenotype.

**Methods:** Human bone marrow mesenchymal stromal cells (hMSCs) and monocytes were isolated from 20 participants (12 male, 8 female) with childhood onset T1D along with 9 age-, sex-, and BMI-matched controls (6 male, 3 female). HMSCs and monocytes were in vitro differentiated towards osteoblasts for 7 days and towards osteoclasts for 9 days to determine the differentiation potential in normoglycemia and in (clinically relevant) hyperglycemia (15 mmol/L). Extracellular flux assays (Seahorse) were performed on hMSCs, monocytes, osteoblastic, and osteoclastic cells to assess mitochondrial function and glycolysis. MicroCT and dynamic histomorphometry were performed on Iliac crest bone biopsies to evaluate the relationship between bone cell metabolism and microstructure.

**Results:** Participants with T1D had a median age of 46 years (29.7 – 57.3) and a median disease duration of 32.7 years (16.4 – 46.6). HbA1C was 54.2 ± 2.8 mmol/mol. The differentiation capacity of hMSCs and monocytes from T1D cases did not differ compared to controls in normoglycemia and hyperglycemia. Moreover, the overall metabolic profile of hMSCs, monocytes, and osteoblastic cells was not different in mitochondrial function and glycolysis in T1D cases. Osteoclastic cells showed a higher ($p = 0.007$) spare respiratory capacity in T1D (2.43±0.88) compared to controls (1.58±0.54). Iliac crest bone microstructure and dynamic remodeling showed no differences between T1D and controls.

**Conclusion:** The preserved metabolic profile of monocytes, hMSCs, and osteoblastic cells from patients with long-standing and well-controlled T1D suggests that impairments in the metabolic program are not a cause of skeletal fragility in T1D.

**Poster Sessions: Genetics and epigenetics**

**P138**

**Decisive gene strategy on osteoarthritis: a comprehensive whole-literature based approach for conclusive gene targets**

*Sui-Lung Su*[1]

[1]*National Defense Medical Center, School of Public Health, Taipei, Taiwan R.O.C.*

**Abstract Text**

**Background:** Previous meta-analyses only examined the association between single or several gene polymorphisms and osteoarthritis (OA), whereas no studies have concluded that there are existing all gene loci that associate with OA.

**Purpose:** To assess whether a definite conclusion of the association between the gene loci and OA can be drawn.

**Methods:** Decisive gene strategy (DGS), a literature-based approach, was used to search PubMed, Embase, and Cochrane databases for all meta-analyses that associated gene polymorphisms and OA. Trial Sequential Analysis (TSA) examined the sufficiency of the cumulative sample size. Finally, we assessed the importance of gene loci in OA based on whether there were enough sample sizes and the heterogeneity of the literatures with I2 value.

**Results:** After excluding 179 irrelevant publications, 80 meta-analysis papers were recruited. Among Caucasians, SMAD3 rs12901499 (OR = 1.20, 95% CI: 1.12-1.29) was a risk factor with validation of sufficient sample sizes through TSA model. Among Asians, there were 3 gene loci risk factors with validation of sufficient sample sizes through TSA model: ESR1 rs2228480, SMAD3 rs12901499, and MMP-1 rs1799750 (OR = 1.35, 95% CI: 1.08-1.69; OR = 1.34, 95% CI: 1.07-1.69; OR = 1.43, 95% CI: 1.18-1.74, respectively). Besides, 3 gene loci, DVWA rs7639618, GDF5 rs143383, and VDR rs7975232 (OR = 0.78, 95% CI: 0.67-0.90; OR = 0.74, 95% CI: 0.67-0.81; OR = 0.56, 95% CI: 0.35-0.90, respectively) were identified as protective factors through TSA model.

**Conclusions:** We used DGS to identify conclusive gene loci associated with OA. These findings provide implications of precision medicine in OA and may potentially advance genetic therapy.

**P140**

**Variants in PDK4, TNR and FN1 genes identified following WGS in a Family with Low Bone Mass and Fractures**

*<u>Chanelle Cilia</u>[1], Francesca Borg Carbott[1], Donald Friggieri[1], Josanne Vassallo[2], Melissa M Formosa[1]*
*[1]University of Malta, Faculty of Health Sciences- Department of Applied Biomedical Science, Msida, Malta*
*[2]University of Malta, Faculty of Medicine and Surgery- Department of Medicine, Msida, Malta*

**Abstract Text**

**Introduction:** Osteoporosis is a silent skeletal disease with a strong genetic background. The study aimed to identify causal gene variants in a family with osteoporosis and fragility fractures.

**Methods:** A three-generation pedigree of seven relatives (aged 27 to 87) was recruited of whom five had osteoporosis or low bone mass, including three with a fragility fracture history. The proband had a femoral neck T-score of -2.6. Whole genome sequencing was performed on five individuals using the Illumina NovaSeq6000 platform. Data was analysed by comprehensive stepwise filtering strategies and multiple *in silico* tools to evaluate the potential causality of the shortlisted variants which were subsequently validated by Sanger sequencing.

**Results:** Gene variant filtering following a dominant inheritance pattern shortlisted three missense variants having an alternative allele frequency of ≤1% in Non-Finnish Europeans from gnomAD, classified as 'variant of uncertain significance' by the ACMG/AMP guidelines, and predicted to be deleterious by multiple *in silico* software. *PDK4* c.878A>T (p.Asp293Val; rs913394387) is located in the histidine kinase domain that is involved in ATP binding and protein phosphorylation, playing an important role in downregulation of sclerostin. *In silico* protein modelling predicted the amino acid change to be structurally damaging. *TNR* c.4486C>T (p.Pro173Arg; rs778130882) is found in between two domains; the coiled coil structure and EGF-like 1 domain and causes protein structural damage due to expansion of surface cavity. *FN1* c.518C>G (p.Arg1496Trp; rs139078629) is present in fibronectin type III domain which is critical for cell migration and adhesion via integrin binding and necessary to support the assembly and organisation of the extracellular matrix.

**Conclusion:** Findings suggest that the shortlisted variants, alone or in combination, may contribute to osteoporosis and fragility fractures, which nonetheless require further functional validation. The coding proteins interact through TNF-α signalling which promotes bone resorption and mediates inflammatory responses underlying bone disease.

**P141**

**WGS Identifies IGF1R as a possible pleiotropic gene underlying Osteoporosis and Diabetes in Malta**

*Marichela Schembri*[1], *Donald Friggieri*[1], *Nikolai P Pace*[2], *Josanne Vassallo*[3], *Melissa M Formosa*[1]
[1]*University of Malta, Department of Applied Biomedical Science- Faculty of Health Sciences, Msida, Malta*
[2]*University of Malta, Department of Anatomy- Faculty of Medicine and Surgery, Msida, Malta*
[3]*University of Malta, Department of Medicine- Faculty of Medicine and Surgery, Msida, Malta*

**Abstract Text**

**INTRODUCTION:** Osteoporosis is a metabolic disorder characterised by low bone mass, quality and strength, and increased fragility. The aim of this study was to identify genetic factors underlying bone mass determination that could potentially be pleiotropic.

**METHODS:** DNA from 100 unrelated Maltese postmenopausal women with high or low bone mineral density (BMD) forming part of the Malta Osteoporotic Fracture Study (MOFS; N=1,045) were subjected to whole genome sequencing on the Illumina NovaSeq6000 platform. An in-house bioinformatics pipeline was used to align raw sequencing read to Genome assembly GrCh38/hg38, call and annotate variants. A stepwise filtering strategy was applied to curate and shortlist variants predicted to be deleterious which were subsequently genotyped in the rest of MOFS and the Maltese Diabetes study (N=320).

**RESULTS:** A novel nonsense variant *IGF1R* c.1722G>A (p.Trp574Ter) was identified in a 79-year-old female with osteoporosis at the FN (T-score: -2.5), having a BMI of 24.3kg/m$^2$ (weight: 50.2kg, height: 144cm) and who had recently developed type 2 diabetes (T2D). The conserved variant is located in the Fibronectin type-III-1 domain involved in the alignment and stability of the receptor. The variant is classified as 'pathogenic' by the ACMG/AMP guidelines (evidence codes PVS1, PM2 and PP3) and has strong *in silico* predictions of possible impact on protein structure and function favouring pathogenicity. The monomorphic variant is absent in both local study collections and gnomAD.

**CONCLUSION:** Our findings suggest that the *IGF1R* c.1722G>A variant might be implicated in the pathogenesis of both osteoporosis and T2D. IGF1R is a transmembrane tyrosine kinase receptor that plays a key role in growth and development through its activation of intracellular signalling pathways. The identified variant may possibly explain the short stature in our patient. Further testing in a larger collection could potentially lead to a better understanding of the variant's association with bone and glucose metabolism.

**Poster Sessions: Inflammation, arthritis and other joint diseases**

**P142**

**Frequency and risk factors of low bone mineral density in young patients with systemic sclerosis**

*Arina Sorokina[1], Natalya Toroptsova[1], Olga Dobrovolskaya[1], Nikolay Demin[1]*
*[1]V.A. Nasonova Research Institute of Rheumatology, Osteoporosis Laboratory, Moscow, Russia C.I.S.*

**Abstract Text**

**Background:** Systemic sclerosis (SSc) is a chronic inflammatory disease and therefore factors associated with SSc may increase the risk of osteoporosis

**Purpose**: to evaluate the frequency of low bone mineral density (BMD) and factors affecting it among young patients with SSc

**Methods:** The study included 84 patients with SSc according to the criteria of ACR/EULAR 2013: 68 (81.0 %) fertile women and 16 (19.0 %) men under the age of 50 (median age - 37.0 [32.0; 44.0] years). BMD was measured at lumbar spine, femoral neck and total hip by dual energy X-ray absorptiometry (DXA). Low BMD was diagnosed if the Z-score was less than -2.0 SD. To identify the factors associated with low BMD, a logistic regression analysis was performed.

**Results**: 17 (20.2 %) patients had low BMD, including 14 (20.5%) women and 3 (18.8 %) man. Univariate logistic regression revealed that low BMD was associated with duration of SSc (OR 1.12; 95% CI [1.02; 1.23]; p = 0.013), joint contractures (OR 3.44; 95% CI [1,05; 11,29], p = 0,039), duration of glucocorticoids (GCs) taking (OR 1.18; 95% CI [1.06; 1.32], p = 0.003) and cumulative dose of GCs (OR 1.07; 95% CI [1.03; 1.12], p = 0.002), body mass index (BMI) < 25 $kg/m^2$ (OR 6.85; 95% CI [1.41; 33.29], p = 0.017). Multivariate analysis demonstrated the association between low BMD and BMI < 25 $kg/m^2$ (OR 6.68; 95% CI [1.27; 35.17], p = 0.025), duration of SSc (OR 1,13 95% CI [1,02; 1,26], p = 0,018) in young patients with SSc.

**Conclusion(s):** 20.2% of young patients with SSc had low BMD, which was associated with BMI and disease duration.

**P143**

**The frequency of osteoporosis and factors associated with it in patients with systemic sclerosis**

*Arina Sorokina[1], Natalya Toroptsova[1], Nikolay Demin[1]*
*[1]V.A. Nasonova Research Institute of Rheumatology, Osteoporosis Laboratory, Moscow, Russia C.I.S.*

**Abstract Text**

**Background:** Osteoarticular involvement in systemic sclerosis (SSc) is frequent and varied. Data are scarce on the prevalence and risk factors of osteoporosis (OP).

**Purpose:** To evaluate the frequency of OP and related factors in patients with SSc.

**Methods:** 200 patients with SSc (median age 52.0 [40.0; 63.0] years) were included: 160 (80.0%) women and 40 (20.0%) men**.** BMD was measured by DXA. In postmenopausal women and men ≥ 50 years OP was diagnosed with T- score at any region ≤ -2.5 SD, in fertile women and men < 50 years - with Z-score <-2.0 SD. To identify the factors associated with OP, a logistic regression analysis was performed.

**Results:** OP was detected in 62 (31.0%) persons. In univariate analysis the presence of OP was associated with age, duration of SSc, duration of proton pump inhibitor use, joint contractures, acroosteolysis, low diffusing capacity of the lung for carbon monoxide, interstitial lung disease, glucocorticoids (GCs) use > 3 months, GCs cumulative dose, current dose of GCs, duration of GCs taking, anti-topoisomerase I positivity, body mass index (BMI) < 24 kg/m$^2$, female gender. Multivariate analysis confirmed association between OP and BMI < 24 kg/m$^2$ (OR 2.86 95% CI [1.49; 5.49], p = 0.001), age (OR 1,05; 95% CI [1,02; 1,07], p < 0.001), duration of SSc (OR 1.07; 95% CI [1.02; 1.11]; p = 0.004), joint contractures (OR 2,08; 95% CI [1,03; 4,19], p = 0,040) and anti-topoisomerase I positivity (OR 2,23; 95% CI [1,13; 4,39], p = 0,019).

**Conclusion(s):** 31% of examined patients with SSc had OP. BMI < 24 kg/m$^2$, age, duration of SSc, joint contractures and anti-topoisomerase I positivity increased the risk of OP.

**P144**

**Frequency and risk factors of low-energy fractures in patients with SSc**

*Arina Sorokina[1], Natalya Toroptsova[1], Olga Dobrovolskaya[1]*
*[1]V.A. Nasonova Research Institute of Rheumatology, Osteoporosis Laboratory, Moscow, Russia C.I.S.*

**Abstract Text**

**Background:** Systemic sclerosis (SSc) is recognized as a chronic infammatory disease and several SSc-associated factors may increase the risk of osteoporosis and fractures

**Purpose:** to study the frequency and factors associated with of fragility fractures in patients with SSc

**Methods:** 200 patients (80,0% women; median age - 52.0 [40.0; 63.0] years) with SSc according to the criteria of ACR/EULAR 2013 were included. The history of low-energy nonvertebral fractures and vertebral fractures according to spine X-ray morphometry was assessed. To identify the factors associated with fragility fractures, a logistic regression analysis was performed.

**Results:** 42 (21%) patients had fragility fractures: 18 (9.0%) - vertebral and 24 (12.0%) - nonvertebral fractures. Univariate analysis revealed the association between fragility fractures and age, duration of SSc, modifed Rodnan skin score, glucocorticoids (GCs) use > 3 months, duration of GCs use, cumulative dose of GCs, glomerular filtration rate, body mass index (BMI) < 23 kg/m$^2$, bone mineral density of lumbar spine. Multivariate analysis confirmed the association between fragility fractures and age (OR 1.08; 95% CI [1.03; 1.14], p <0.001), modifed Rodnan skin score (OR 1.12; 95% CI [1.01; 1.24], p = 0.025) and GCs use > 3 months (OR 5.05; 95% CI [1.31; 19.37], p = 0.017).

**Conclusion(s):** We found out that 21% of SSc patients had a history of fragility fractures. The risk of fragility fractures was increased with age, modifed Rodnan skin score and GCs use > 3 months.

**P145**

**Body composition in postmenopausal women with rheumatoid arthritis**

*Olga Dobrovolskaya[1], Nikolay Demin[1], Natalia Toroptsova[1]*
*[1]V.A. Nasonova Research Institute of Rheumatology, Department of Osteoporosis, Moscow, Russia C.I.S.*

**Abstract Text**

**Objective:** to determine the body composition of postmenopausal women with rheumatoid arthritis (RA).

**Material and methods:** The study included 177 women with RA, median age was 62 [56; 67] years, duration of postmenopause - 12 [6; 18] years, duration of RA - 8 [4; 14] years. Body composition and bone mineral density (BMD) were determined by dual-energy x-ray absorptiometry using «Discovery A» (Hologic, USA). Osteoporosis (OP) was diagnosed as a decrease in T-score <-2.5 SD in lumbar spine, femoral neck or total hip. Sarcopenia was determined when appendicular mass index (AMI) was <5.5 kg/m$^2$, osteosarcopenia: T-score was <-1.0 SD, AMI was <5.5 kg/m$^2$, sarcopenic obesity: normal BMD, AMI <5.5 kg/m$^2$ and total fat >35% (overfat) and osteosarcopenic obesity - T-score <-1.0 SD, AMI <5.5 kg/m$^2$ and total fat >35%. The study was approved by the local ethics committee.

**Results:** Disease activity score in 28 joints-erythrocyte sedimentation rate was 5.17 [4.40; 5.86] points for the group. The median body mass index (BMI) was 26.2 [23.5; 30.3] kg/m$^2$. 96 (54.2%) patients were constantly taking oral glucocorticoids, and 55 (31.1%) people used biological therapy. The mean appendicular muscle mass and AMI were 16.8±3.0 kg and 6.5±1.1 kg/m$^2$, respectively. AMI <5.5 kg/m$^2$ was detected in 36 (20.3%) patients. 131 (74.0%) women had overfat, among them 35 (26.7%) had normal BMI. Isolated OP was found in 13 (7.3%) patients, OP with overfat - in 36 (20.3%) persons. 13 (7.3%) and 18 (10.2%) patients had osteosarcopenia and osteosarcopenic obesity, respectively.

Only 1 woman had sarcopenic obesity, and no cases of isolated sarcopenia were detected. Isolated obesity had 76 (42.3%) women. Only 8 (4.5%) patients had normal body composition.

**Conclusion:** about 95% postmenopausal women with RA had abnormal body composition. The most frequency phenotype was isolated obesity. Normal body composition had only 4.5% patients with RA.

**P146**

**The frequency and factors associated with sarcopenia in postmenopausal women with rheumatoid arthritis**

*Olga Dobrovolskaya[1], Alexey Feklistov[1], Nikolay Demin[1], Maria Kozyreva[1], Natalia Toroptsova[1]*
*[1]V.A. Nasonova Research Institute of Rheumatology, Department of Osteoporosis, Moscow, Russia C.I.S.*

**Abstract Text**

**Aim:** to evaluate the frequency of sarcopenia (SP) and factors associated with it in postmenopausal women with rheumatoid arthritis (RA).

**Material and methods**: 104 postmenopausal women (age 60.5 [51.1; 66.0] years) with confirmed RA (ACR/EULAR, 2010) were enrolled. Nutritional status was assessed using a MNA (Mini Nutricial Assessment) questionnaire. All patients underwent handgrip strength by mechanical dynamometer for muscle strength determing. A laboratory examination and dual-energy X-ray absorptiometry were conducted. Osteoporosis (OP) was diagnosed according to WHO criteria, and sarcopenia (SP) was diagnosed according to the criteria EWGSOP2. The study was approved by the local ethics committee.

**Results**. SP was diagnosed in 33 (31.7%) women with RA. Persons with and without SP (SP+ and SP- respectively) did not differ in age and RA duration (p>0.05). Normal body mass index (BMI) was found in 72.7% SP+ patients and in 28.2% SP- (p<0.001). Women SP+ had bone mineral density (BMD) lower than those SP- both in the lumbar spine (LS) and in the hip. OP was diagnosed in 18 (54.5%) patients SP+ and in 17 (23.9%) women SP- (p=0.002).

AMM correlated with BMI (r=0.61, p<0.001), BMD in L1-L4 (r=0.32, p<0.001), BMD in femur neck (r=0.53, p<0.001), BMD in total hip (r=0.44, p<0.001). Negative correlations were found with C-reactive protein (r=-0.18, p=0.036). AMM was not correlated with age. In univariate logistic regression analyses SP was associated with BMI<25 kg/m$^2$ (OR 8.13; 95%CI (3.30-20.01), p<0.001), OP (OR 4.01; 95%CI 1.78-9.03), p<0.001), and nutritional by MNA<24 points (OR 2.82; 95%CI (1.16-6.83), p=0.022).

**Conclusion**: 1/3 of postmenopausal women with RA had confirmed SP. Factors associated with SP were BMI<25 kg/m$^2$, presence of OP and reduced nutritional status.

**P147**

**Osteoporosis, sarcopenia, osteosarcopenia and the risk of falls and fractures in patients with**

**rheumatoid arthritis**

*Olga Dobrovolskaya[1], Nikolay Demin[1], Alexey Feklistov[1], Maria Kozyreva[1], Natalia Toroptsova[1]*
*[1]V.A. Nasonova Research Institute of Rheumatology, Department of Osteoporosis, Moscow, Russia C.I.S.*

**Abstract Text**

**Aim**: to assess the frequency of osteoporosis (OP), sarcopenia (SP), osteosarcopenia (OSP) and risk of falls and fractures in women with rheumatoid arthritis (RA).

**Material and methods**: 102 postmenopausal women (mean age 60.1±9.2 years), with confirmed RA (median duration 8.0 [4.0; 15.0] years) were enrolled. The study was approved by the local ethics committee. Using the FRAX tool, 10-year probability of major osteoporotic fracture was estimated. Dual energy X-ray absorptiometry (DXA) was performed to evaluate body composition and bone mineral density. Falls and low-energy fractures were recorded during a 3-year prospective study. Univariant logistic regression was used to identify risk of falls and fractures.

**Results.** 20 (19.6%) patients had OP, 10 (9.8%) - SP and 13 (12.7%) – OSP. According to FRAX, 75.0% of patients with OP, 40.0% - with SP and 92.3% - with OSP had a high risk of fractures versus 33.9% of women with normal body composition (NBC) ($p<0.001$). The frequency of falls during the year before the study in patients with OP, SP, OSP was 20.0%, 40.0, 61.5% respectively and in women with NBC - 22.4% ($p<0.05$), during the prospective follow-up, the frequency of falls was 15.0%, 50.0%, 53.8% and 10.3%, respectively ($p<0.001$). During the follow-up, 5 (4.9%) persons had fractures. The frequency of fractures did not differ between the groups. SP and OSP increased the risk of falls compared with women with NBC (OR 9.3 (2.53-40.99), p =0.001 and OR 13.25 (2.81-64.48), p=0.001, respectively).

**Conclusion.** In postmenopausal women with RA 42.2% had OP, SP or OSP, among them 72.1% had a high 10-year probability of fractures. OSP increased the risk of falls by 13 times; SP – by 9 times in women with RA. We did not found relationship between the presence of abnormal body composition and fractures. Further research is needed involving more people with SP/OSP.

**P148**

**Distinct Pathomechanism of Temporomandibular Joint Involvement in Rheumatoid Arthritis: Insights from the hTNFtg Arthritis Model**

*Darja Andreev[1], Pauline Porschitz[1], Daniela Weidner[2], Georg Schett[2], Lina Gölz[3], Aline Bozec[2]*
*[1]Technische Universität TU Dresden, Center for Regenerative Therapies Dresden CRTD, Dresden, Germany*
*[2]Friedrich-Alexander-Universität FAU Erlangen-Nürnberg and Universitätsklinikum Erlangen UKER, Department of Medicine 3 - Rheumatology and Immunology, Erlangen, Germany*
*[3]Friedrich-Alexander-Universität FAU Erlangen-Nürnberg and Universitätsklinikum Erlangen UKER, Dental Clinic 3 – Department of Orthodontics and Orofacial Orthopedics, Erlangen, Germany*

**Abstract Text**

Rheumatoid arthritis (RA) and other inflammatory arthritides are chronic conditions characterized by joint inflammation and bone erosion. While smaller joints are commonly affected early in RA, larger joints become involved as the disease progresses. Notably, the temporomandibular joint (TMJ) is affected in over 50% of RA patients, leading to severe jaw pain and functional impairment of the stomatognathic system. Despite its prevalence, TMJ involvement is often underestimated, and its cellular and molecular mechanisms remain poorly understood. Due to the unique biological and functional properties of the TMJ, inflammatory pathways observed in other joints such as the well-studied ankle joint may not directly apply to the TMJ.

This study aimed to identify a reliable inflammatory arthritis model to investigate TMJ-specific pathomechanisms. The human TNF-α transgenic mice (hTNFtg) model was shown to effectively replicate TMJ pathology observed in arthritic patients, including inflammation, cartilage degradation, and severe bone loss. These changes were driven by increased osteoclast activity and upregulation of osteoclast-associated genes. Comparative analysis with the ankle joint revealed distinct patterns of immune cell infiltration and cytokine expression in the TMJ, particularly involving T cell recruitment. Bulk RNA sequencing confirmed widespread inflammation in both joints of hTNFtg mice compared to controls. However, the TMJ displayed unique transcriptomic signatures, with increased expression of genes linked to energy dysregulation and bone resorption-related enzymes, such as cathepsin K and tartrate-resistant acid phosphatase. In contrast, the ankle joint exhibited higher levels of immune cell and pro-inflammatory cytokine-related gene expression.

These findings highlight the TMJ as a distinct anatomical site with heightened susceptibility to arthritis-related damage. Our data underscore the importance of joint-specific research to develop tailored therapeutic strategies for effective management of TMJ involvement in RA patients.

**P149**

**Association of alkaline phosphatase with inflammatory signature of bone marrow mesenchymal stromal cells of hip osteoarthritis patients**

*<u>Drenka Trivanovic</u>[1], Marko Vujačić[2,3], Slavko Mojsilović[1], Milica Rajković[1], Tamara Kukolj[1], Dragana Aleksandrović[1], Ivana Okić Djordjević[1], Nikola Bogosavljević[2,3], Srdja Janković[4], Zoran Baščarević[2,3], Aleksandra Jauković[1]*
*[1]Institute for Medical Research University of Belgrade, Group for Hematology and Stem Cells, Belgrade, Serbia*
*[2]Institute for Orthopedy Banjica, Surgery, Belgrade, Serbia*
*[3]Faculty of Medicine University of Belgrade, Orthopedic Surgery, Belgrade, Serbia*
*[4]University Children's Hospital, Department of Hematology and Oncology, Belgrade, Serbia*

**Abstract Text**

The endogenous ecto-enzyme tissue non-specific alkaline phosphatase (TNAP) participates in skeletal tissue mineralization, as well as ATP degradation and production of adenosine which significantly attenuates inflammatory conditions. However, the implication of TNAP in osteoarthritis (OA) pathology and progression is not clear. Here, we explored the association of TNAP activity and the *ALPL* gene encoding TNAP expression with the inflammatory profile of mesenchymal stromal cells (MSCs) isolated from hip OA patients.

Bone marrow MSCs were isolated from acetabular spongiosa (aMSCs) and femoral metaphysis (fMSCs) of matched patients undergoing hip replacement surgery (n=7, Ethical approval I-97/14.3). To silent *ALPL* gene expression, siRNA duplex oligonucleotides was applied, while non-targeting control siRNA was used as negative control. Secretome, protein and gene expression were assessed by flow cytometry (multiplex assay), immunofluorescence microscopy, and qPCR. Statistical significance was estimated by non-parametric tests.

Our results show that aMSCs secrete higher amounts of pro-inflammatory IL-1β, IFN-α2, IFN-γ, IL-6, IL23 and IL-12p70, as well as anti-inflammatory IL-10 in comparison with fMSCs (n=6). This was followed by stronger expression of NFκB protein in aMSCs than in fMSCs. On the other side, we found reduced TNAP activity and *ALPL* gene expression associated with lower adipogenic potential of aMSC when compared to fMSCs. Pharmacological inhibition of NFκB increased TNAP activity and adipogenesis of aMSCs. Downregulation of *ALPL* gene led to a significant elevation of inflammatory IL-1β (p=0.002), IFN-γ (p=0.041), IL-23 (p=0.045), IL-10 (p=0.043) and IL-12p70 (p=0.036) in fMSCs. In addition, *ALPL*-silencing led to the downregulation of *PPAR-γ* and *IL-6* gene expression in fMSCs.

The obtained results indicate an important role of TNAP molecule in the regulation of inflammatory states of MSCs, where *ALPL* deficiency is associated with increased inflammatory cytokine production. Further establishing of *ALPL* gene knockout cultures will improve the investigation of TNAP roles in the inflammatory phenotype of OA patients.

**P150**

**Locoregional Immune Response in Thoracic and Abdominal Aortic Aneurysms**

*Viola Niemann*[1], *Nicolas Stumpe*[2], *Ulrich Flögel*[2], *Maria Grandoch*[1]
[1]*Universitätsklinikum Düsseldorf, Institute for Translational Pharmacology, Düsseldorf, Germany*
[2]*Universitätsklinikum Düsseldorf, Institute for Molecular Cardiology, Düsseldorf, Germany*

**Abstract Text**

**Background:** Abdominal and thoracic aortic aneurysms (AAA, TAA) differ in their hemodynamic properties, the composition of the extracellular matrix of the vascular wall and the inflammatory response, characterized by an increased haematopoietic activity. Since vertebral bodies are haematopoietically active organs and are located close to aortic aneurysms, the question arises as to whether individual vertebrae in close proximity to developing aneurysms are particularly haematopoietically active or whether all vertebrae react in a similar way.

**Purpose:** Aim of the current study was to analyse the locoregional haematopoietic responses in different bone marrow niches during aneurysm development.

**Methods:** For studying TAA and AAA the model of combined application of angiotensin II (Ang-II, 1000 ng/kg/min) and β-Aminopropionitril (BAPN, 0,1 % in drinking water) was used in male 8-12 week old C57BL/6J mice. Aneurysms were investigated using magnetic resonance imaging (MRI), ultrasound, histological and flow cytometric analysis of bone marrow and aorta, and enzyme-linked immunosorbent assay for detection of stromal cell derived factor (SDF) 1.

**Results:** Treatment with Ang-II/BAPN resulted in TAA in 30 % of the treated mice, AAA were observed in 20 % and TAA and AAA developed in 42 %. Flow cytometric analysis revealed increased numbers of common myeloid and granulocyte-macrophage progenitor cells, as well as more mature neutrophils, lymphocytes and myeloid cells in vertebral bodies in close proximity to developing aneurysms. This locoregional activation was confirmed by 19F MRI, which detected immune responses in co-localised vertebral bodies and aortic sections. In line, histological staining confirmed more mononuclear cells in the vascular niche of these locally activated vertebral bodies. An altered extracellular matrix and increased levels of SDF1 in vertebral bodies close to aneurysms promote immune cell migration from the endosteal to the vascular niche.

**Conclusion:** Our results underscore the critical role of locoregional hematopoiesis occuring during the development of thoracic and abdominal aortic aneurysms.

**P151**

**Decreased bone matrix mineralization and increased size of osteocyte lacunae sections in patients with axial spondyloarthritis (axSpA)**

*Barbara Misof[1], Stéphane Blouin[1], Markus Hartmann[1], Rafaela Ceron[2], Marcelo Pinheiro[3], Carolina Moreira[2], Natalia Machado[4]*
*[1]Ludwig Boltzmann Institute of Osteology at Hanusch Hospital of OEGK and AUVA Trauma Centre Mei dling, 1st Med. Dept. Hanusch Hospital, Vienna, Austria*
*[2]Hospital de Clinicas da Universidade Federal do Paraná SEMPR,*
*Academic Research Center Pro Renal Institute- Endocrine Division, Curitiba, Brazil*
*[3]Universidade Federal de São Paulo UNIFESP/ EPM, Rheumatology Division, São Paulo-SP, Brazil*
*[4]Universidade Federal do Paraná, Rheumatology Division, Curitiba, Brazil*

**Abstract Text**

**Background:** Osteoporosis is frequently observed in patients with axial spondyloarthritis (axSpA), however, the pathophysiology thereof is unclear.

**Purpose:** We aimed for information about bone mineralization density distribution (BMDD) and osteocyte lacunae sections (OLS) in axSpA.

**Methods:** Transiliac biopsy samples from n=21 men with axSpA (age 46±6 years; duration of disease 2-40 years, on average 18±12 years [mean ± SD]) were measured for BMDD and OLS in cancellous (Cn.) and cortical (Ct.) bone compartments separately based on quantitative backscattered electron imaging and compared to previously obtained normative BMDD-REF and OLS-REF data. Furthermore, we analyzed correlations of our outcomes with clinical disease duration and activity score.

**Results:** The Cn.BMDD of the patients was shifted towards lower calcium concentrations. The average degree of mineralization Cn.CaMean was decreased by -2.3% ($p<0.05$), while the heterogeneity of mineralization and the percentage of lowly mineralized bone areas were increased (Cn.CaWidth +9%, p=0.02 and CaLow +43%, p=0.008, respectively) compared to BMDD-REF. Ct.BMDD was not altered. Of note, one sample presented with osteomalacia. Cn.OLS-area and Ct.OLS-area were both increased (+10% and +30%, respectively, both p<0.001) and the Cn.OLS-density was decreased (-11.5%, p<0.001) compared to OLS-REF. Neither BMDD- nor OLS-parameters were correlated with disease duration or activity score.

**Conclusions:** The bone specimens of the patients with SpA showed decreased bone matrix mineralization, which contributes to the previously reported decreased bone mass (by DXA). The observed decrease in mineral content of the bone matrix cannot be attributed to lower bone tissue age, as the studied cohort did not present with increased bone formation based on histology as reported previously. Together with altered osteocyte lacunae size, our findings might indicate some osteocyte functional abnormalities related to an altered bone matrix mineralization, which however needs to be further studied in patients with axSpA.

**P152**

**Serotype-Specific Direct and Indirect Effects of E. coli Lipopolysaccharides on Joint Cells Inflammation and Metabolism**

*Ana Alonso-Pérez[1], María Piñeiro-Ramil[1], Verónica López-López[1], María Guillán-Fresco[1], Andrés Pazos-Pérez[1], Miriam López-Fagúndez[1], Reetta Satokari[2], Kaisa Hiippala[2], Alberto Jorge-Mora[1], Rodolfo Gómez[1]*
*[1]Health Research Institute of Santiago de Compostela IDIS, Musculoskeletal Pathology Group, Santiago de Compostela, Spain*
*[2]University of Helsinki, Human Microbiome Research, Helsinki, Finland*

**Abstract Text**

**Background:** Dysbiosis in gut microbiota composition is increasingly recognized as a key factor in the pathogenesis of various diseases. This condition facilitates the release of bacterial lipopolysaccharides (LPS) into circulation, leading to metabolic endotoxaemia, a potential driver of chronic inflammation and disease progression. However, although various bacterial species have been linked to rheumatic diseases, the effects of specific LPS serotypes on joint tissues remain unexplored.

**Purpose:** This study aimed to compare the effects of LPS from different *E. coli* serotypes on the immune and metabolic responses of chondrocytes and other joint cell types.

**Methods:** ATDC5 chondrocytes and SW-982 synoviocytes were exposed to 100 ng/ml of different LPS serotypes for 48 hours. Furthermore, SW-982 cells were pre-treated with LPS O26:B6 for 72 hours, and the resulting conditioned medium was used to treat ATDC5 cells. The expression levels of genes related to inflammation, chemotaxis, and metabolism were assessed using real-time PCR.

**Results:** LPS from serotype O26:B6 demonstrated the strongest inflammatory, pro-catabolic, and anti-anabolic effects, whereas LPS O55:B5 exhibited the weakest (Figure 1A). Similarly, LPS O26:B6

triggered strong inflammatory and chemotactic responses in synoviocytes, while LPS O55:B5 had no significant effects (Figure 1B). In addition, conditioned medium from LPS O26:B6-treated synoviocytes elicited significant inflammatory and catabolic responses in chondrocytes, including enhanced expression of the matrix-degrading enzyme ADAMTS compared to direct LPS O26:B6 treatment (Figure 1C).

**Conclusions:** LPS from different *E. coli* serotypes exhibit distinct inflammatory and pro-catabolic potentials, which may help explain the inconsistent associations between specific bacterial species and rheumatic diseases observed in some studies. These discrepancies could arise from serotype-specific effects. Furthermore, synoviocyte-derived factors induced by LPS may contribute to joint inflammation and cartilage degradation, suggesting an indirect mechanism in endotoxaemia-associated chronic inflammation. This implies that LPS may not need to directly reach the articular cavity to exert its pathological effects.

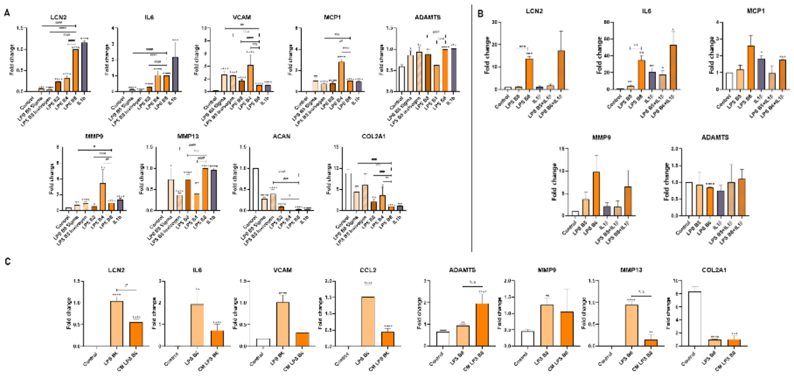

**P153**

**Identification of subchondral bone endotypes in hip osteoarthritis**

*Assil-Ramin Alimy[1], Maximilian Möhlmann[1], Julius Rosenfeld[1], Eva Goedecke[1], Frank Timo Beil[1], Jeroen Geurts[2], Tim Rolvien[1]*
*[1]University Medical Center Hamburg-Eppendorf, Department of Trauma and Orthopaedic Surgery, Hamburg, Germany*
*[2]University Hospital Lausanne and University of Lausanne CHUV-UNIL, Department of Musculoskeletal Medicine- Rheumatology, Lausanne, Switzerland*

**Abstract Text**

**Background:** The hip is one of the anatomical sites most commonly affected by osteoarthritis (OA). While pathomechanistic aspects of hip OA are still largely unclear, current treatments focus on symptom relief without addressing patient-specific characteristics.

**Purpose:** This study aimed to identify distinct endotypes based on subchondral bone properties in hip OA to provide a basis for individualized treatment strategies.

**Methods:** Femoral heads were collected during total hip arthroplasty (n=38) and underwent a detailed histological assessment. Additionally, backscattered electron imaging was performed to investigate mineralization and osteocyte lacunar properties. Principal component analysis was conducted to reduce the dimensionality of the data, and hierarchical clustering was performed to group the specimens according to their similarity.

**Results:** Cluster analysis revealed four distinct groups based on their osteochondral characteristics. Despite no differences in BMD T-scores of the hip (p=0.568), clusters I and II were histologically characterized by low subchondral bone mass, in contrast to the high bone mass phenotype of cluster IV. Additionally, osteoblast and osteoid indices progressively increased across the clusters, with the highest amount in clusters III and IV. Remarkably, cluster IV was notable for its low mineralization and higher osteocyte lacunar density (N.Ot.Lc/B.Ar ($mm^{-2}$); cluster IV: 381 vs. cluster I: 187 p<0.001; vs. cluster II: 224, p<0.001; vs. cluster III: 253, p<0.001). Additionally, reduced bone marrow adipocyte size (a surrogate for lipolysis) was observed in clusters III and IV and was also associated with increased subchondral bone formation in the overall cohort ($R^2$=0.557, p<0.001).

**Conclusion:** Our data identify four distinct subchondral bone endotypes with unique profiles of microstructure, mineralization, and cellular activity, reflecting varying disease activity and treatment needs, such as osteoanabolic treatment in clusters I and II and antiresorptive treatment in clusters III and IV. Furthermore, our results suggest a previously unrecognized role of lipolysis in modulating hip OA endotypes.

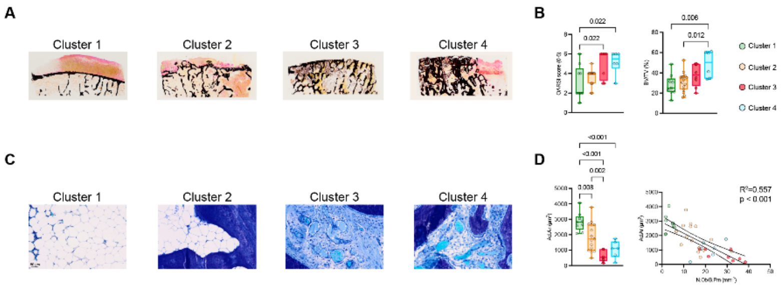

A) Representative von Kossa-stained sections of osteochondral specimens from patients with hip OA in each cluster. B) Histological evaluation of OARSI scores (left) and bone volume quantification (right). C) Representative toluidine blue-stained sections of bone marrow across clusters. D) Quantification of bone marrow adipocyte size (left) and its association with osteoblast density (right).

**P154**

**Single center experience of the treatment of calcinosis associated with juvenile dermatomyositis**

*<u>Irina Nikishina</u>[1], Olga Kostareva[1], Svetlana Arsenyeva[1], Valeria Matkava[1]*
*[1]V.A. Nasonova Scientific Research Institute of Rheumatology, Paediatric, Moscow, Russia C.I.S.*

**Abstract Text**

**Introduction**: Calcinosis is a feared and difficult to manage complication of Juvenile Dermatomyositis (JDM).Biologics and JAK-inhibitors become promising options especially in refractory cases with complications although there are no special controlled studies and registered indications regarding their use.

**Purpose**:To describe real world data of long term managing of JDM pts with calcinosis.

**Methods**:Single center data of 67 pts(27/40% male,40/60% female) with Juvenile Idiopathic Inflammatory Myopathies(JIIM) who were observed in our clinic since 2020 to 2024 years.

**Results**:Our cohort includes 51 pts with JDM and 16 pts with overlap syndromes.Calcinosis was observed in 17/25%(11-JDM,5–overlap syndrome).The median age at onset for this category of pts was 6.92 y.o.(min-10mo; max-12y.o.).The median disease duration before establishing diagnosis–8 mo(min–1;max–88).17/100% pts had myopathic syndrome,14/88%-typical skin lesions,16/94% suffered from arthritis, the lung involvement-in 4/23%.Laboratory findings included positive ANA in 13/76%, level of CK was elevated in 7/41%. 17/100% pts received glucocorticoids and methotrexate(MTX),4 of them combined with hydroxychloroquine,2-mycophenolate mofetil,IVIG-11/64% pts.Due to refractory course of JIIM 9/17/53% were treated by Biologics and JAK-inhibitors(among them 1/9/11% pts with overlap syndrome).Spectrum of therapy included abatacept (4 pts–in 1-st line, 1–in 2-nd),rituximab (2 pts–in 1-st line),tofacitinib (2 pts–in 1-st line, 2–in 2-nd),upadacitinib(1 pt–in 3-rd line after inefficacy of abatacept and tofacitinib).Under the therapy we observed improvement of muscle force,relieve of myopathic syndrome,decrease of calcinosis in all cases.We observed a significant regression of calcifications in one patient under the upadacitinib after failures in the use of abatacept and tofacitinib sequentially, which was confirmed by the low-dose whole-body CT with dynamics after 1 year.

**Conclusion**:Despite the "off label" status of Biologics ang JAK-inhibitors for JIIM (JDM including),we satisfied with their good effectiveness and favorable safety profile for pts with calcinosis.The most impressive thing in our experience was the observation of a significant regression of calcifications in 16 y.o. boy as a result of the use of upadacitinib.

**P156**

**Hormone replacement therapy and the risk of knee and hip replacement: Insights from the OsteoLaus cohort study**

*Jeroen Geurts[1], Seda Aydogdu[1], Cinja Koller[1], Elena Gonzalez Rodriguez[2], Didier Hans[2], Olivier Lamy[2], Thomas Hügle[1], Enisa Shevroja[2]*
*[1]Lausanne University Hospital, Rheumatology, Lausanne, Switzerland*
*[2]Lausanne University Hospital, Interdisciplinary Center for Bone Diseases, Lausanne, Switzerland*

**Abstract Text**

**Background:** The potential benefit of hormone replacement therapy (HRT) on the risk of incident joint replacement, remains a debated topic due to mixed evidence from both interventional and cross-sectional radiographic studies.

**Purpose:** In this study we assessed the association of HRT status and duration with the incidence of total knee (TKR) or hip replacement (THR) among post-menopausal women.

**Methods:** We conducted a population-based cohort study utilizing data from the longitudinal OsteoLaus study, which included five consecutive visits conducted at regular intervals over a 10-year period between 2010 and 2022. We included participants who had a minimum follow-up duration of 7.5 years (*n*=1026). The presence of TKR & THR was evaluated using DXA images obtained during each visit. Cases with joint replacement at baseline or following hip fracture were excluded (*n*=87). Cox proportional hazard models estimated the hazard ratio (HR) and 95% CIs of incident TKR or THR relative risks associated with use (never, former, current) and duration of self-reported HRT use. Results were adjusted for multiple covariates (age, BMI, femoral BMD and osteoporotic drug use at baseline, comorbidities, ΔBMI, ΔBMD and HRT duration after baseline).

**Results:** Participants with incident TKR (*n*=63) had significantly higher baseline age (66.4±6.2 vs. 63.1±7.2 years), BMI (29.2±4.8 vs. 25.2±4.3 kg/m²), and fBMD (0.76±0.10 vs. 0.73±0.11 g/cm²) compared to controls (*n*=908). Participants with incident THR (*n*=55) demonstrated higher baseline age (66.6±5.7 years) and fBMD (0.77±0.14 g/cm²). Current HRT use at baseline (*n*=256) was not significantly associated with incident TKR (HR 1.56[0.84–2.90]) or THR (HR 1.45[0.74–2.85]). However, HRT duration at baseline was associated with incident TKR (HR 1.06[1.01–1.12]) but not with incident THR (HR 1.04[0.97–1.10]). No differences in HRT starting age were observed.

**Conclusion:** In conclusion, longer HRT duration may increase TKR risk, potentially through hormonal effects on joint inflammation or bone remodeling. No association was found with THR, suggesting differing pathophysiological mechanisms.

### Poster Sessions: Mammalian and non-mammalian models, including preclinical imaging

### P157

### The effect of alendronate on the life span and bone properties of the short-lived turquoise killifish Nothobranchius furzeri

*Maria Butylina[1], Michael Kothmayer[2], Katharina Wahl-Figlash[1], Katharina Gelles[1], Oliver Pusch[2], Peter Pietschmann[1]*
*[1]Medical University of Vienna MUW, Center for Pathophysiology- Infektiology and Immunology, Vienna, Austria*
*[2]Medical University of Vienna MUW, Center of Anatomy and Cell Biology, Vienna, Austria*

**Abstract Text**

**Introduction:** Nitrogen containing bisphosphonates (e.g. alendronate) effectively inhibit osteoclastic bone resorption and are considered as first-line treatment of osteoporosis and Paget's disease. Additionally, several studies suggest a beneficial effect on mortality that goes beyond life-extending effects attributed to the well-established fracture risk reduction. However, the mechanisms of the mortality reducing effect of bisphosphonates are unclear. The turquoise killifish (*Nothobranchius furzeri*), an established organism of accelerated ageing, exhibits an extremely short life cycle and thus could represent a suitable model for survival studies.

**Purpose:** The aim of this study was to test the effect of alendronate on the life span and bone properties of *Nothobranchius furzeri*.

**Methods:** 75 female *Nothobranchius furzeri* were divided at the age of 16 weeks to a treatment (10 µM alendronate, n=37) and a control (n=38) group and monitored for survival. Vertebral bodies of deceased fish of both groups were examined by micro-computed tomography (µ-CT).

**Results:** Comparing the median survival of both groups, the alendronate group showed significantly higher values (18.6 weeks vs. 17.8 weeks; p = 0.0324). Unexpectedly, µ-CT evaluation of the $15^{th}$ vertebral body showed significantly higher values for bone (+2%; p= 0.0013) and tissue mineral density (+3%; p= 0.0159) in the control group. Comparing the age of the micro-CT investigated subpopulation, the alendronate group had significantly higher survival rates (19.3 weeks vs. 17.6 weeks; p= 0.0217).

**Conclusion:** Our results indicate that alendronate has a positive effect on the survival of *Nothobranchius furzeri* and alters bone mineralization. These findings can be taken into consideration for future studies of mechanisms contributing to a potential mortality reducing effect of bisphosphonates.

**Funding:** This study was supported by the Austrian Science Fund (FWF) (P35209).

**Ethical approval:** This project was approved by the intramural Committee for Animal Experimentation and Austrian Federal Ministry of Education, Science and Research (BMBWF) (2022 - 0.238.324).

**ND-P008**

**Exploring the effects of the enterokine FGF19 on the skeleton in preclinical models of osteosarcopenia associated with aging and chronic disuse**

<u>*Maura Strigini*</u>[1]
[1]*Université Jean Monnet Saint-Étienne- Mines Saint Etienne- INSERM, SANBIOSE- U1059, Oullins, France*

**Abstract Text**

**Introduction:** Osteosarcopenia, the coexistence of osteopenia/osteoporosis and sarcopenia, represents a significant health concern in the elderly and in bedridden individuals, with an increased risk of falls and fractures. The ileum-produced Fibroblast Growth Factor 19 (FGF19) was recently shown to prevent muscle weakness in murine models.

**Purpose:** We investigated the therapeutic potential of FGF19 in mitigating bone and muscle deterioration in aged mice and mice subjected to mechanical unloading (chronic disuse) of posterior legs.

**Methods:** 22-month old male mice were injected with FGF19 (0.1mg/kg) or vehicle daily for 3 weeks (n=32); 4-month old male mice were subjected to hindlimb unloading (HU) or ground control conditions and similarly injected with FGF19 or vehicle (ethical approval CECAPP_LS_2019_005;

38620-2022082500391402). Bone phenotypes was assessed by microCT at 10.5 µm isotropic cubic resolution, histomorphometry and RTqPCR on mRNA extracted from femurs.

**Results:** In aged mice, microCT analysis showed a clear increase in the cross-sectional area of the femur distal metaphysis of treated mice (median: Vehicle: 3.4 $mm^2$; FGF19: 3.7 $mm^2$, P=0.006), confirmed by 2D slice by slice analysis, and significantly increased polar Moment Of Inertia (pMOI), an indicator of bone strength and torsional rigidity ($p<0.001$). FGF19 also increases cortical porosity and decreases cortical thickness, and leaves other skeletal sites and trabecular bone unaffected, leaving open the question of the overall biomechanical impact. Histomorphometry on femur sections shows no effect on osteoblasts and a non-significant decrease in osteoclast number (p=0.06). In mechanically unloaded mice, that showed the expected decrease in cortical thickness at diaphysis ($p<0.01$) and increased porosity ($p<0.05$) FGF19 treatment prevented such changes.

**Conclusions:**

FGF19 has modest, yet potentially beneficial effects on the skeleton, that are site specific and model-specific.

**Poster Sessions: Muscle, physical activity and bone**

**P158**

**Reference values and cut-points for trunk myopenia and myosteatosis in Chinese adults: a nationwide multi-center study**

*<u>Wenshuang Zhang</u>[1,2], Liu Yandong[2], Yuan Yi[1,2], Geng Jian[2], Cheng Zitong[2], Hu Bo[2], Wang Ling[2], Cheng Xiaoguang[1,2]*
*[1]Peking University Fourth School of Clinical Medicine, Department of Radiology, Beijing, China*
*[2]Beijing Jishuitan Hospital- Capital Medical University, Department of Radiology, Beijing, China*

**Abstract Text**

**Background:** CT-based trunk muscle measurements are emerging as potential markers for sarcopenia, but the cut-points for myopenia and myosteatosis have not been established in the Chinese adult population. Understanding age- and sex-specific reference values is essential to define these conditions accurately.

**Purpose:** To develop sex- and age-specific reference values and cut-points for trunk muscle area and density, and to investigate the age-related changes in trunk muscle characteristics in a large cohort of Chinese adults.Methods:4016 community-dwelling individuals aged 21~80yrs from 12 distinct regions in China were recruited between 2013 and 2017. Trunk muscle area and density were measured from abdominal CT images at the L1 and L3 levels using OsiriX software. Reference values for muscle area and density were calculated using lambda-mu-sigma(LMS) method, and sex- and age-specific percentile charts were developed(Figure1). Cut-points for myopenia and myosteatosis were established using the mean minus two standard deviations(M-2SD) method for participants aged 21~40 years.

**Results:** An age-related decline in both trunk muscle area and density was observed. From 25to75yrs, the annual reductions in muscle area were -0.45% at L1 and-0.55% at L3 for men,and -0.33% at L1 and-0.41% at L3 for women.Annual reductions in muscle density were -0.38%at L1 and -0.35%at L3 for men, and -0.55%at L1 and -0.52%at L3 for women. Accelerated muscle loss was observed in women at menopause and in older men. The cut-points for trunk skeletal muscle area, density, and index were as follows: L1—96.0$cm^2$, 29.8HU, and 32.5$cm^2/m^2$ for men, and 63.1$cm^2$, 26.4HU, and 24.5$cm^2/m^2$ for women; L3—122.3$cm^2$, 31.7HU, and 41.3$cm^2/m^2$ for men, and 75.4$cm^2$, 27.5HU, and 29.4$cm^2/m^2$ for women.

**Conclusion:** This study establishes sex- and age-specific reference values and cut-points for trunk muscle area and density at the L1 and L3 levels in the Chinese adult population. These findings will aid future research on myopenia and myosteatosis and contribute to the definition of trunk muscle sarcopenia.

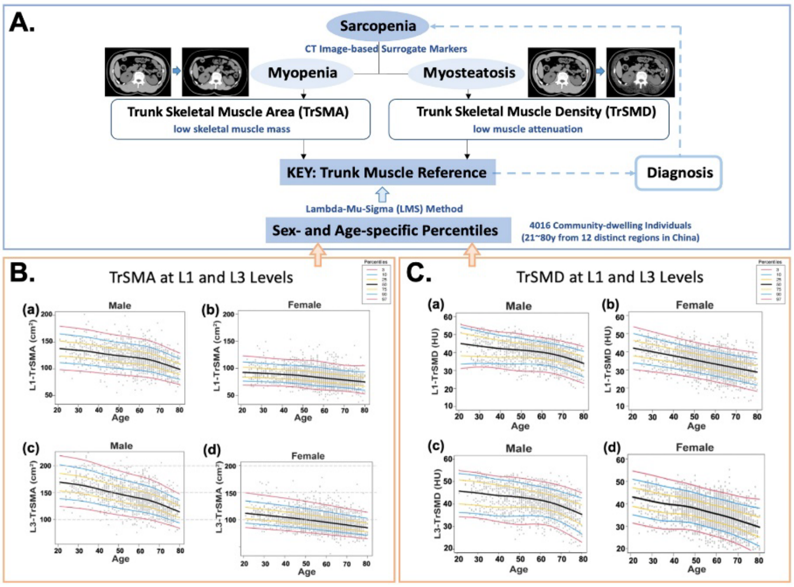

**Figure 1**: **A**. Schematic of the study design. **B**. Percentile charts of L1-TrSMA (a, b) and L3-TrSMA (c, d) in males and females aged 21 to 80 years. **C**. Percentile charts of L1-TrSMD (a, b) and L3-TrSMD (c, d) in males and females aged 21 to 80 years. TrSMA, trunk skeletal muscle area. TrSMD, trunk skeletal muscle density.

**P159**

**Associations of Hip and Thigh Muscle Size and Density with Proximal Femur Bone Mineral Density in Older Hip Fracture Patients**

*Wenshuang Zhang[1,2], Ge Yufeng[2], Liu Yandong[2], Geng Jian[2], Zhang Qingyu[1,2], Wang Ling[2], Cheng Xiaoguang[1,2]*

*[1]Peking University Fourth School of Clinical Medicine, Department of Radiology, Beijing, China*

*[2]Beijing Jishuitan Hospital- Capital Medical University, Department of Radiology, Beijing, China*

**Abstract Text**

**Background**: Muscle weakness and fragile bones are major factors leading to hip fractures in older adults.The forces generated by muscle contractions play a role in shaping bone strength and driving changes in bone structure and mass.However,the connection between muscle size and density and trabecular bone mineral density(BMD) in proximal femur remains insufficiently understood,especially in individuals with hip fractures.

**Purpose**: This study investigated associations of hip and thigh muscle size and density with trabecular BMD of proximal femur subregions in older hip fracture patients.

**Methods** :A total of 479 hip fracture patients aged ≥65 years underwent hip quantitative computed tomography(QCT) between 2018 and2019.Patients using walking aids before the fracture were excluded to ensure representativeness of the unaffected hip side.Trabecular areal BMD(aBMD) of the femoral neck(FN), trochanter(TR), intertrochanter(IT), and total hip(TH) was measured using Mindways QCTPro software.Cross-sectional area (CSA) and density of the gluteus medius/minimus (G.Med/Min) and gluteus maximus (G.Max) were measured using OsiriX software with a threshold of -29HU to differentiate muscle from fat. Measurements were taken from the unaffected side. Pearson correlation coefficients and scatterplots were used to analyze relationships between muscle parameters and BMD.

**Results**: The study included 388 participants (280 females, 108 males), with a mean age of 77.5±7.4 years. The strongest correlation was observed between average muscle size and TR-aBMD ($r2$=0.60, 95%CI:0.53–0.66, $p$<0.001). Muscle size parameters (e.g., thigh CSA: $r2$=0.56 with FN-aBMD, $r2$=0.54 with IT-aBMD) consistently showed stronger correlations with BMD compared to muscle density (e.g., thigh density: $r2$=0.39 with FN-aBMD, $r2$=0.32 with IT-aBMD, all $p$ <0.001). Correlation analysis revealed linear trends between muscle size and BMD, with the strongest association observed for TR-aBMD(Figure1).

**Conclusion**: In older hip fracture patients, muscle size is more strongly associated with proximal femur trabecular BMD than muscle density, with the strongest association at TR-aBMD. These findings highlight the importance of preserving muscle mass for bone health and hip fracture management.

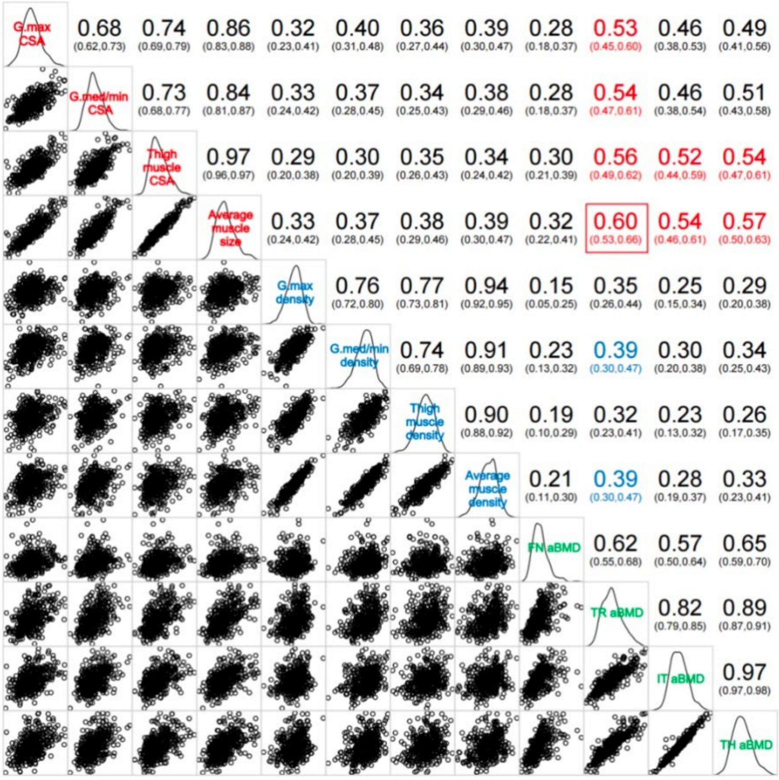

**Figure 1**. Correlation matrix of hip and thigh muscle size and density with proximal femur bone mineral density (BMD). Pearson correlation coefficients were used to evaluate the relationships between muscle parameters and BMD, with all p-values < 0.001. Average muscle size was calculated as the mean of G.Max CSA, G.Med/Min CSA, and thigh muscle CSA, while average muscle density was defined as the mean of G.Max density, G.Med/Min density, and thigh muscle density.

**P160**

**Dynamic Phases and Non-Linear Progression of Disuse Muscle Atrophy During Acute Immobilization After Acetabular Fracture: Insights From Opportunistic CT in a Real-World Study**

*<u>Yufeng Ge</u>[1], Ling Wang[2], Minghui Yang[1], Klaus Engelke[3], Xinbao Wu[1]*
*[1]Beijing Jishuitan Hospital- Capital Medical University,*
*Department of Orthopaedics and Traumatology, Beijing, China*
*[2]Beijing Jishuitan Hospital- Capital Medical University, Department of Radiology, Beijing, China*
*[3]University of Erlangen, Institute of Medical Physics, Erlangen, Germany*

**Abstract Text**

**Background:** Disuse muscle atrophy (DMA) caused by immobilization is a well-known phenomenon in fracture patients. However, the specific dose-response relationship between the duration of immobilization and the progression rate of DMA remains poorly defined.

**Purpose:** To explore the time-dependent progression of DMA during acute immobilization in patients with acetabular fractures using opportunistic CT imaging.

**Methods:** Data from 380 acetabular fractures patients (mean age: 48.1 ± 15 years, 75.5% male) at a trauma center in Beijing, China were retrospectively analyzed. Two opportunistic hip CT scans of each patient were utilized: the first within three days post-injury and the second within 30 days during bed rest. Upper-thigh muscle cross-sectional area (CSA) was measured, and DMA was calculated as the percentage change (($2^{nd}$ - $1^{st}$) / $1^{st}$×%). Generalized additive models were applied to investigate the nonlinear relationship between immobilization duration and DMA, with automatic inflection points detection.

**Results:** Prolonged immobilization was significantly associated with increased DMA (B= -1.01, *P*< 0.001). A nonlinear, inverted S-shaped relationship with three phases-onset, acceleration, and deceleration-was identified, with inflection points around day 5 and day 15 (likelihood ratio test *P* < 0.001). Before day 5, the rate of DMA was not significant (B= -0.26, *P*= 0.494). From day 5 to day 15, the DMA rate increased sharply (B= -1.64, *P*< 0.001). After day 15, the rate slowed (B= -0.74, *P*= 0.004). Subgroup analysis revealed that patients with high-volume injuries or multiple traumas experienced significantly faster DMA (*P* for interaction= 0.017 and 0.004, respectively).

**Conclusion:** The progression of DMA in acetabular fracture patients follows a distinct "onset, acceleration, and deceleration" nonlinear pattern, with rapid atrophy starting after the first week of immobilization. These findings are critical for guiding strategies to preserve muscle mass in prolonged immobilization, particularly in patients with multiple traumas.

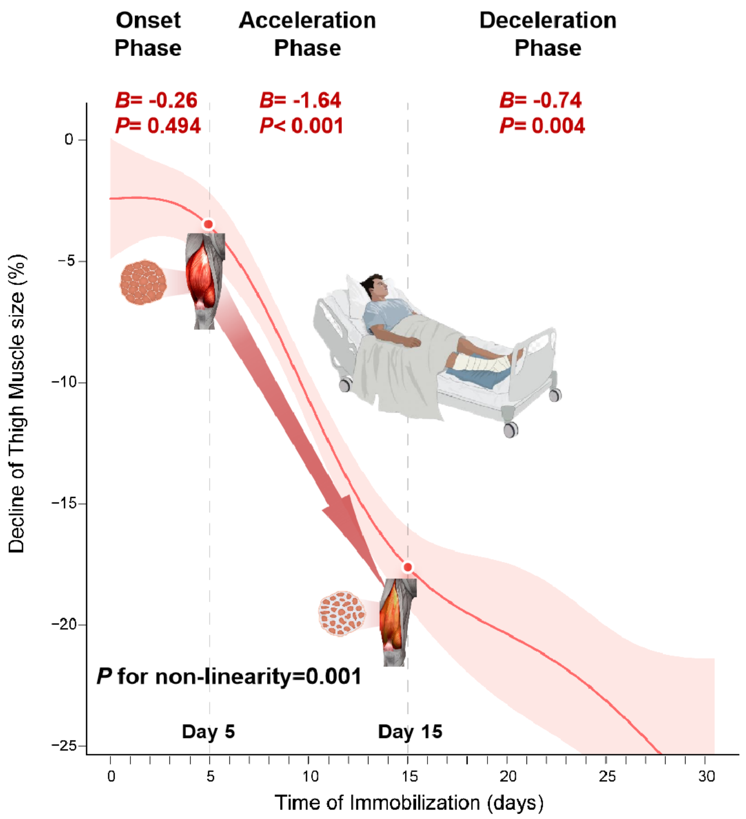

**Figure.** Onset-Acceleration-Deceleration Progression of Disuse Muscle Atrophy Over Immobilization Duration After Adjustment for Age, Sex, and BMI

**P161**

**Quantitative evaluation of cervical muscles in patients with degenerative cervical myelopathy using DTI metrics**

*Bo Hu[1], Jiuheng Li[2], Yandong Liu[1], Wenshuang Zhang[1], Yi Yuan[1], Jian Geng[1], Zitong Cheng[1], Qingyu Zhang[1], Xiaoguang Cheng[1], Xiao Han[2], Ling Wang[1]*
*[1]Beijing Jishuitan Hospital- Capital Medical University, Department of Radiology, Beijing, China*
*[2]Beijing Jishuitan Hospital- Capital Medical University, Department of Spine Surgery, Beijing, China*

**Abstract Text**

**Background:** DTI is increasingly being applied to quantitative muscle measurements. In DCM patients, DTI of the deep paraspinal cervical muscles can reveal the microscopic structure of the muscles, but research on its relationship with clinical function has been largely overlooked.The objective of this research is to assess the DTI parameters of deep cervical muscles in individuals diagnosed with degenerative cervical myelopathy (DCM) before and after surgery at different follow-up stages, to reflect the changes in paraspinal muscles, and to analyze the correlation between these imaging findings, clinical manifestations, and functional assessment scores.

**Methods**: This study involved 30 patients with DCM who underwent cervical laminoplasty. MRI DTI imaging and clinical assessments were performed preoperatively as well as at 3-month and 6-month postoperative intervals. DTI parameters of cervical paraspinal muscles (multifidus and semispinalis cervicis) were measured at the C3/4, C4/5, and C5/6 intervertebral discs levels, and average values were calculated. Preoperative and postoperative changes in DTI parameters were analyzed using the Friedman test. Spearman correlation analysis was conducted to assess the relationship between preoperative cervical paraspinal muscle DTI measurements and changes in modified Japanese Orthopaedic Association (mJOA) scores and Visual Analog Scale (VAS) at 3-month and 6-month follow-up.

**Results**: Preoperative paraspinal muscle FA (*P*=0.045) correlated with VAS recovery at 3-month and 6-month follow-up (*P*=0.010, *P*=0.008), and mJOA recovery rate at 6-month follow-up (*P*=0.045). Linear regression analysis showed that higher preoperative paraspinal muscle FA was a significant predictor of decreased VAS scores at 3-month and 6-month follow-up (*P*=0.024, *P*=0.053) and increased mJOA scores at 6-month follow-up (*P*=0.028).

**Conclusion**: In DCM patients, changes in DTI parameters from preoperative to postoperative follow-up stages can indicate paraspinal muscle recovery after cervical surgery. Preoperative paraspinal muscle FA is associated with better postoperative spinal cord function and pain recovery. DTI

examination before surgery can predict patient recovery outcomes.

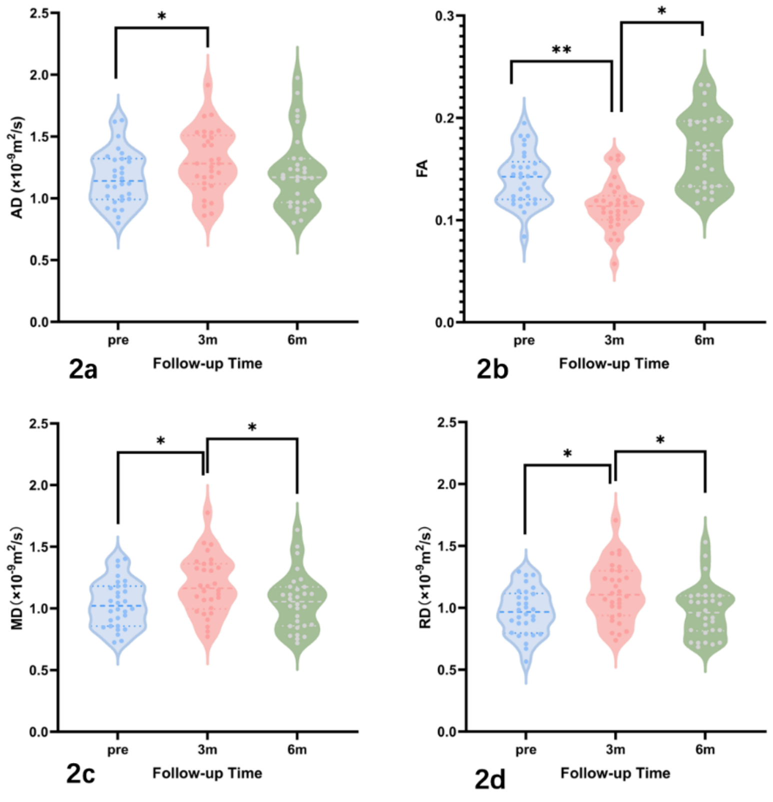

**P162**

**Sarcopenic phenotype and physical performance in rheumatoid arthritis patients treated by biologic disease-modifying anti-rheumatic drugs**

*Olga Dobrovolskaya[1], Maria Kozyreva[1], Alexey Feklistov[1], Natalia Toroptsova[1]*

*[1]V.A. Nasonova Research Institute of Rheumatology, Department of Osteoporosis, Moscow, Russia C.I.S.*

**Abstract Text**

**Aim:** to evaluate appendicular lean mass, muscle strength and physical performance in rheumatoid arthritis (RA) women treated by biologic disease-modifying anti-rheumatic drugs (bDMARDs).

**Materials and methods.** The study included 63 women (mean age 60.3±8.9 years) with confirmed RA treated with bDMARDs (bDMARDs +) and control group (117 age-matched RA patients who had never previously treated with BDMARDs). Clinical and laboratory examination, evaluation of body composition using dual-energy X-ray absorptiometry and tests to assess muscle strength and physical performance were carried out. The sarcopenic phenotype was diagnosed with an appendicular lean mass of less than 15 kg or an appendicular lean mass index of less than 5.5 kg/m$^2$. The study was approved by the local ethics committee.

**Results.** The frequency of sarcopenic phenotype among RA patients was 23.3%: in group bDMARDs + – 27.0%, in the control – 21.4% (p>0.05). There were no differences among the groups based on the results of muscle strength assessment tests, however, patients bDMARDs + performed significantly better the short physical performance battery (SPPB) and had a higher gait speed (p=0.016 and p=0.002, respectively). Univariate linear regression analysis confirmed the relationship of the bDMARDs presence with the physical performance according to the results of SPPB (b*=0.24; p=0.018) and gait speed (b*=0.28; p=0.006).

**Conclusion.** The frequency of sarcopenic phenotype among patients with RA was 23.3% and was comparable in those who received bDMARDs and without it. There were no differences in muscle strength depending on the presence of bDMARDs, but the physical performance was significantly better in women bDMARDs +.

**P163**

### Characterizing the molecular signatures of an in vitro model of muscle senescence: implications for sarcopenia and frailty

*Amir Mohammad Malvandi[1], Laura Gerosa[1], Valerio Leoni[2], Simone Cristoni[3], Giuseppe Banfi[1,4], Giovanni Lombardi[1,5]*
*[1]IRCCS Ospedale Galeazzi-Sant'Ambrogio, Laboratory of Experimental Biochemistry and Advanced Diagnostics, Milano, Italy*
*[2]University of Milano-Bicocca- Azienda Socio Sanitaria Territoriale Della Brianza- ASST-Brianza- Desio Hospital, Department of Laboratory Medicine, Desio, Italy*
*[3]ISB-Ion Source & Biotechnologies Srl, n.a., Bresso, Italy*
*[4]Vita-Salute San Raffaele University, Faculty of Medicine and Surgery, Milano, Italy*
*[5]Poznan University of Physical Education, Department of Athletics- Strength and Conditioning, Poznan, Poland*

**Abstract Text**

**Background:** During aging the accumulation of senescent cells could contribute to a variety of tissue dysfunctions, particularly in the musculoskeletal system. The integrity of bone and muscle function relies on complex molecular interactions, and disruptions in one component affect the other. However, the related pathological mechanisms, specifically those linking cellular senescence to musculoskeletal decline, remain poorly understood.

**Purpose:** This study aimed to further characterize the *in vitro* murine model of muscle senescence and aging that we developed at both stages of myoblast and differentiated myotubes.

**Methods and Results:** We performed untargeted high resolution mass spectrometry to identify pivotal molecular trends in senescent myoblasts. Key findings include significant shifts in eicosapentaenoic acid (EPA) metabolism, mitochondrial functional dynamics, and different fatty acid metabolic pathways. Reduced mitochondrial fission 1 protein levels and altered mitochondrial morphology observed by western blot and immunofluorescence analyses support the mitochondrial dynamics impairment observed in senescent myoblasts. Further quantitative target analysis using GS-MS method, confirmed significant changes in very long fatty acids (VLFAs) and cholesterol metabolites, as the final output of enriched pathways in both senescent myoblasts and derived sarcopenic-like myotubes. A significant reduction in many VLFAs, with an increase in C24 and C26 fatty acids and the increase in oxysterol markers of oxidative stress (7bOHC and 7KC) suggested peroxisome activation. Moreover, the reduced levels of desmosterol and lathosterol in senescent myoblasts suggest a disruption in cholesterol synthesis. In sarcopenic-like myotubes, derived from senescent myoblasts, the decrease in desmosterol coupled with the increase in lathosterol may indicate dysregulation of cholesterol synthesis, leading to the accumulation of sterol intermediates that could further stress the cell.

**Conclusion:** This study reveals a distinct molecular signature of senescent muscle cells, providing a valuable in vitro model to explore therapeutic targets for age-related musculoskeletal impairments and identify biomarkers linked to sarcopenia and frailty.

**P164**

**Senescent muscle cells and their secretory factors: implications for bone dysfunctions in aging**

*Laura Gerosa*[1], *Chiara Verdelli*[1], *Amir Mohammad Malvandi*[1], *Maria Emanuela Orlando*[1], *Giuseppe Banfi*[1,2], *Giovanni Lombardi*[1,3]

*[1]IRCCS Ospedale Galeazzi Sant'Ambrogio,*
*Laboratory of Experimental Biochemistry and Advanced Diagnostics, Milan, Italy*
*[2]Vita-Salute San Raffaele Univeristy, Faculty of Medicine and Surgery, Milan, Italy*
*[3]Poznan University of Physical Education, Department of Athletics- Strength and Conditioning, Milan, Italy*

**Abstract Text**

Beside the mechanical coupling, bone-skeletal muscle functional unit relies on endocrine-like interactions: any disruption in one component can affect the other. However, the pathophysiological mechanisms related to the entire functional unit are poorly defined. Aging impacts the musculoskeletal apparatus, with senescent cells accumulating and contributing to the dysfunctional phenotype, particularly through the senescence-associated secretory phenotype (SASP), which affects other tissues.

This study investigates the molecular pathways linking muscle cell senescence to bone impairment by analyzing SASP composition, focusing on soluble factors, extracellular vesicles (EVs) and EVs miRNA content.

We used an *in vitro* model of murine sarcopenic-like myotubes derived from senescent myoblasts that we recently developed. Supernatants from these sarcopenic-like myotubes are enriched in myostatin (+50.4% *p= 0.0226, one-way ANOVA Tukey's multiple comparisons test) and RANTES/CCL5 (+77.30% *p= 0.0452, unpaired t-test), while showing a reduction in osteoprotegerin (-27.90% **p= 0.0021, one-way ANOVA Tukey's multiple comparisons test). Nanoparticle tracking analysis reveal that sarcopenic-EVs are in lower amount and of greater average size compared to control. Analyses of sarcopenic-EVs miRNA indicate that miR-27a-3p and miR-206-3p content differs significantly to control (miR27a-3p control= 2.327, sarcopenic= 0.3821 *p= 0.0315; miR206-3p control= 5.398, sarcopenic= 2.036 *p= 0.0445, unpaired t-test). Further, preliminary experiments demonstrate that the exposure to conditioned medium (CM) from myoblasts and myotubes differentially altered the expression of periostin, osteopontin, and alkaline phosphatase (Alp) in MC3T3-E1 pre-osteoblasts during differentiation. Specifically, long-term treatment with CM from sarcopenic-like myotubes downregulated osteopontin and Alp, while upregulated periostin and modifyed ALP enzyme activity in mature osteoblasts.

This study provides insights into the SASP-associated secretome composition from sarcopenic-like myotubes and its potential effects on bone cells. Specifically, CM treatment revealed that factors released from sarcopenic-like myotubes impair osteoblast function. Further research is needed to elucidate the molecular interactions between muscle and bone, which may lead to new therapeutic approaches.

**P165**

**Correlating Diffusion Tensor Imaging Parameters with Lumbar Paraspinal Muscle Fat Infiltration: Implications for Sarcopenia Diagnosis**

*Wenshuang Zhang[1,2], Hu Bo[2], Liu Yandong[2], Yuan Yi[1,2], Geng Jian[2], Cheng Zitong[3], Zhang Qingyu[1,2], Wang Ling[2], Cheng Xiaoguang[2]*
*[1]Peking University Fourth School of Clinical Medicine, Department of Radiology, Beijing, China*
*[2]Beijing Jishuitan Hospital- Capital Medical University, Department of Radiology, Beijing, China*
*[3]Beijing Jishuitan Hospital- Capital Medical University, Departmrnt of Radiology, Beijing, China*

**Abstract Text**

**Background:** Aging leads to increased paraspinal muscle sarcopenia, with fat infiltration being a key marker. While diffusion tensor imaging(DTI) is widely used for tissue analysis, its role in paraspinal muscle disorders is underexplored. This study investigates the correlation between DTI parameters and fat infiltration, aiming to identify potential biomarkers for sarcopenia.

**Purpose:** To evaluate the relationship between DTI-derived parameters and proton density fat fraction(PDFF) values in lumbar paraspinal muscles, and to determine whether DTI can be utilized to assess muscle degeneration and fat infiltration.

**Methods:** Sixteen community volunteers(5males, 11females; mean age 45.2±13.4years; BMI 24.2±3.5kg/m$^2$)with chronic lumbar discomfort participated.MRI scans were performed using a 3T MR system, including T1-vibe, Q-dixon,and DTI sequences. Regions of interest(ROIs) were manually delineated on both sides of the paravertebral muscles at the L5/S1 and L4/5 levels, totaling 64 ROIs. DTI protocol included:TR/TE=3900/47ms, 20 diffusion directions, 2-fold acceleration.Post-processing of data was conducted using FSL, ITK-SNAP, and DSI Studio software. Spearman's correlation and scatterplots were used to assess the relationship between DTI parameters(FA, MD, AD, RD)and PDFF values.A p-value of<0.05 was considered statistically significant.

**Results:** This study successfully demonstrated the feasibility of employing DTI for fiber tracking within the lumbar paraspinal muscles. The three-dimensional visualization of fiber tracking(Figure1) vividly showcased the fibrous structure of the paraspinal muscles, offering clear and notable insights.An analysis of the 64 ROIs yielded histograms illustrating the distribution of PDFF, FA, MD, AD,and RD values(Figure2).Additionally,scatterplots unveiled significant positive correlations between FA values and PDFF, MD, AD,and RD values(all with p-values<0.01), as detailed in Figure3.

**Conclusion:** This study demonstrates the potential of DTI to quantify lumbar paraspinal muscle degeneration and fat infiltration, with implications for sarcopenia diagnosis. The significant correlations between DTI parameters and PDFF support DTI's utility as a non-invasive imaging modality to monitor sarcopenia progression. Further studies with larger sample sizes are necessary to validate these findings and explore their clinical applications.

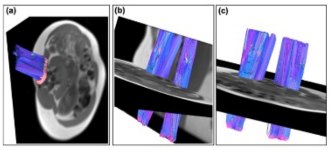

**Figure 1:** DTI 3D Tractography of Paraspinal Muscles. (a) Illustrates the muscle fiber tracking of one side of the paraspinal muscles, demonstrating the detailed fibrous architecture. (b) and (c) Display bilateral paraspinal muscle fiber tracking, showcasing comparative structural visualization across both sides.

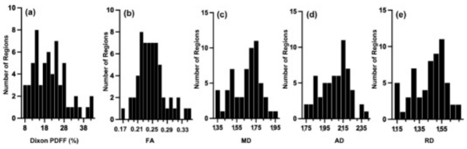

**Figure 2:** Histograms show measurements of (a) Q-Dixon proton density fat fraction (PDFF), (b) fractional anisotropy (FA), (c) mean diffusivity (MD), (d) axial diffusivity (AD), and (e) radial diffusivity (RD).

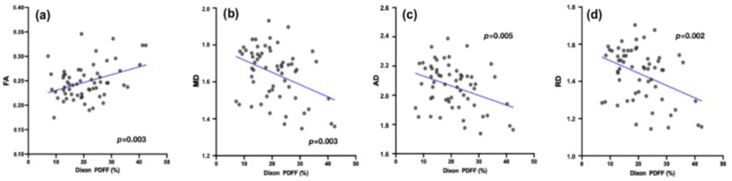

**Figure 3:** Scatterplots of Q-Dixon proton density fat fraction (PDFF) against (a) fractional anisotropy (FA), (b) mean diffusivity (MD), (c) axial diffusivity (AD), and (d) radial diffusivity (RD) respectively.

**P166**

**Site-specific associations between age and bone strength indicators in an archaeological sample may relate to childhood motor development**

*Alex Ireland[1], Antony Colombo[2], Charlotte Henderson[1]*
*[1]Manchester Metropolitan University, Department of Life Sciences, Manchester, United Kingdom*
*[2]École Pratique des Hautes Études - PSL, Maison de l'Archéologie, Bordeaux, France*

**Abstract Text**

The onset of independent walking in infancy results in substantial loading of the lower limb bones during a period of rapid growth. Previous work has shown that age of independent walking is associated with large gains in lower limb bone mass, and that these advantages persist across childhood and adulthood. Whilst contemporary motor development is well characterised, little is known about typical ages of motor milestone attainment in past populations.

Therefore we examined bone properties in 37 individuals aged from 0-4 years from a skeletal collection from the North West of England from the seventh to twelfth centuries. Peripheral quantitative computed tomography scans of the radius, femur and tibia were taken at 5% increments along each bone from 15-85% distal-proximal length. Associations between age estimated from dental mineralisation and long bone growth, and bone properties were examined using LOESS local regression.

For femur and tibia bone properties including cortical area and thickness, values decreased from birth to one year of age, thereafter increasing. For the radius, a similar pattern was evident but with an earlier inflexion point at six months. Whilst differences in cortical bone mineral density were also evident from the same timepoints, the trajectories varied substantially between each bone. There was little evidence of site-dependency of these associations between regions of the same bone.

Decreases in bone strength indicators identified in the first year of life in this archaeological sample may relate to reduced post-natal bone loading during physical activity. Conversely, increases in the same indicators at six months (radius) and twelve months (tibia and femur) may relate to attainment of independent crawling and walking respectively. Study of bone properties in infants may inform on child-rearing practices, and on developmental of related changes in culture. Future work should validate these findings in more modern populations with detailed information on motor milestones.

**P167**

**Differential bile acids profiles in individuals with and without osteosarcopenia**

*Marco Balasso[1], Zoltan Mezei[2], Markus Herrmann[1], Harjit Bhattoa[2], Nerea Alonso[1], Giovanny Rodriguez-Blanco[1]*
*[1]Medical University of Graz, Clinical Institute for Medical and Chemical Laboratory Diagnostics, Graz, Austria*
*[2]University of Debrecen, Department of Laboratory Medicine - Faculty of Medicine, Debrecen, Hungary*

**Abstract Text**

Bile Acids (BAs) are associated with both muscle and bone health, but their role in osteosarcopenia is unknown. We aim to investigate the bile acid pool in fasting serum samples from 95 females >50 years of age with available clinical information on bone and muscle health. Twenty-three individuals were diagnosed with osteosarcopenia. The study was approved by the local ethics committee. We used a targeted approach using a reverse phase LC-MS/MS method to quantify fifteen analytes including free, taurine- and glycine-conjugated BAs. An unbiased/extended approach including Orbitrap technology and data dependent acquisition was used to identify sulphated and glucuronidated conjugations. We used mzMine 4 software (feature extraction) and the GNPS platform (annotation based on public fragmentation spectral libraries). Non-parametric statistical tests were performed in GraphPad Prism v10.

Patients with osteosarcopenia presented lower concentrations of primary bile acids (PBAs) (2.3[1.3-4.4] vs 1.4[0.5-3.2] uM, p = 0.0098), than those without. PBA concentrations correlated with sarcopenia quality of life score, Activin A, skeletal muscle mass ($r_{range}$=0.239-0.389, $p_{range}$=0.02-9.6x10$^{-}$

$^{5}$), P1NP and osteocalcin ($r_{range}$=0.204-0.233, $p_{range}$=0.047-0.02). PBAs negatively correlated with the severity of sarcopenia ($r_{range}$=-0.207-0.284, $p_{range}$=0.04-0.005) and phosphate levels (r=-0.226, p = 0.03). ROC analysis showed that levels <1.5 µM of PBAs best identify patients with osteosarcopenia (AUC=0.690, p=0.006, Youden Index=0.37). Patients with sarcopenia and osteosarcopenia had elevated glucuronidated-UDCA (1.5 fold change, p=0.01) and sulfated bile acids (fold change range=1.7-11, $p_{range}$=0.01-1.8x10$^{-4}$) compared to controls. They correlate positively with age, P3NP, SARC-F questionnaire and severity of sarcopenia ($r_{range}$=0.208-0.358, $p_{range}$=0.04-3.9x10-4). Sulfated BAs negatively correlated with hand grip score and gait score, CTx and BSAP ($r_{range}$=-0.207--0.261, $p_{range}$= 0.04-0.01).

Low BA levels in patients with osteosarcopenia might be due to an increased catabolism via sulfation and glucuronidation processes, which adversely impacts muscle health. Our results suggest a potential role of BAs as markers for osteosarcopenia.

**P170**

**Calibration of CT muscle density: from CT values to physical density**

*<u>Klaus Engelke</u>[1], Jonathan Bamassel[2], Fjola Johannesdottir[3], Oliver Chaudry[4], Ling Wang[5]*
*[1]Friedrich-Alexander-University FAU Erlangen-Nürnberg,*
*University Hospital- Department of Medicine 3, Erlangen, Germany*
*[2]Friedrich Alexander University FAU of Erlangen-Nürnberg,*
*University Hospital- Department of Medicine 3, Erlangen, Germany*
*[3]Harvard Medical School,*
*Center for Advanced Orthopedic Studies- Beth Israel Deaconess Medical Center, Boston, United States*
*[4]Friedrich Alexander University FAU Erlangen-Nürnberg, University Hospital- Institute of Radiology, Erlangen, Germany*
*[5]Jishuitan Hospital, Department of Radiology, Beijing, China*

**Abstract Text**

Currently, CT muscle density is reported as CT value in Hounsfield units (HU). CT values vary linearly with density and muscle density varies linearly with fat infiltration; thus muscle density can be calibrated to mg/cm$^3$ and fat infiltration can be obtained by a linear calibration of CT density to fat fraction. These calibrations require two absolute CT values, one for 'muscle tissue' and one for fat. Muscle tissue ideally would represent fat free contractile tissue, however such a material is difficult to manufacture. Thus the use of 'standard' muscle and adipose tissues as specified by the International Commission on Radiation Units (ICRU reports 44 and 46) was evaluated.

An electron density phantom (EDP, QRM, Moehrendorf, Germany) containing inserts with these two materials was measured on four different CT scanners (table) on top of a bone density calibration (BDC) phantom from QRM at 80 to 140 kV (100 mAs, pitch of 1, 3 mm slice thickness, medium kernels). The BDC water insert was used to correct for potential offsets of the routine scanner calibration. CT values of ICRU muscle were largely independent of scanner and kV, thus an HU value of 50 for using ICRU muscle as standard could be used as reference muscle value. Remaining accuracy errors of 5 HU resulted in errors of about 10% for ICRU muscle density. Despite regular CT calibration, a water offset correction is important for accurate muscle density. kV related CT value variations of ICRU adipose tissue (tissue) were much larger than for ICRU muscle and therefore the use of CT values determined in subcutaneous fat of the scan under investigation may be used as fat reference instead.

| | 80 kV | | 140 kV | |
|---|---|---|---|---|
| **Scanner** | $CT_0$[HU] | $CT_1$[HU] | $CT_0$[HU] | $CT_1$[HU] |
| **ICT-SP** | 47.1 / -92.9 | 45.7 / -94.2 | 44.4 / -66.9 | 45.3 / -66.0 |
| **X.cite** | 46.4 / -102.0 | 47.3 / -101.2 | 45.2 / -72.0 | 45.2 / -71.8 |
| **go.Top** | 46.6 / -101.1 | 48.2 / -99.6 | 44.9 / -71.3 | 44.4 / -72.1 |
| **Force** | 51.8 / -93.7 | 49.4 / -96.0 | 45.8 / -70.0 | 44.4 / -71.4 |
| **Mean (SD)** | 48.0 (2.6) / -97.4 (4.8) | 48.0 (1.6) / -97.8 (3.2) | 45.1 (0.6) / -70.1 (2.3) | 44.8 (0.5)* / -70.3 (2.9)* |
| | 100 kV | | 120 kV | |
| **Force** | 49.3 / -80.1 | 46.5 / -83.7 | 47.1 / -74.1 | 45.5 / -75.7 |

CT values [HU] for ICRU muscle tissue / subcutaneous adipose tissue (SAT) for 80 and 140 kV. CT values before ($CT_0$) and after ($CT_1$) water offset correction. ICT-SP is a Philips scanner, X.cite, go.Top and Force are Siemens scanners. * significant difference compared to $CT_1$ at 80 kV

**P171**

**Risk of sarcopenia in postmenopausal women with vertebral fractures**

*Anna Musiienko[1], Nataliia Grygorieva[1], Nataliia Zaverukha[1], Alina Iniushyna[1], Daryna Kurylo[1]*
*[1]D.F. Chebotarev Institute of Gerontology of the National Academy of Medical Sciences of Ukraine, Department of Clinical Physiology and pathology of the musculoskeletal system, Kyiv, Ukraine*

**Abstract Text**

**Background:** Sarcopenia is a significant age-related disease contributing to disability, reduced quality of life, and mortality, especially in older patients with osteoporosis. Vertebral fractures (VFs), a common osteoporotic complication, cause back pain, diminished quality of life and longevity, and an increased risk of further fractures.

**Objective:** The study aimed to assess the risk of sarcopenia in postmenopausal women with previous VFs.

**Materials and Methods:** A retrospective analysis was conducted on data from 500 females aged 60+ (mean age 70.2±6.7 years), including 374 without any previous fractures and 126 with confirmed VFs. Patients with VFs had significantly lower weight, height, and body mass index compared to those without fractures. The risk of sarcopenia was evaluated using the SARC-F questionnaire, hand grip strength and sit-to-stand test measurement, and DXA indices based on EWGSOP2 criteria.

**Results:** Patients with VFs had significantly higher SARC-F scores (4.0 [1.0–5.0] vs. 2.0 [0.0–4.0], Z=3.7; p=0.0003), indicating a greater risk of sarcopenia. SARC-F correlated with hand grip strength in both groups (p<0.00001) and with the sit-to-stand test in individuals without fractures (p=0.000008) but not for subjects with VFs (p=0.18). Patients with VFs reported more difficulties for the following: strength (lifting and carrying 10 pounds): 25.4 vs. 42.0%, respectively; assistance in walking (across a room): 47.6 vs 61.5%; rising from a chair: 34.9 vs. 50.8%; climbing stairs (10 steps): 36.5 vs 49.5%; and falls in the past year: 64.3 vs. 71.7%, respectively. A high risk of sarcopenia (SARC-F≥4) was identified in 54.8% of VF patients and 37.7% of subjects without fractures. Sarcopenia was confirmed in 20% of VF patients and 8.8% of persons without fractures.

**Conclusion:** Patients with VFS exhibit a higher risk of sarcopenia compared to subjects without fractures. These findings highlight the need for integrated strategies to prevent bone and muscle loss in the elderly.

**P172**

**Role of the sympathetic nervous system (SNS) in a model of bone loss by immobilization in mice**

*<u>Hélène Libouban</u>[1], Quentin Massiquot[2], Marie Veziano[1]*
*[1]University of Angers, RMeS- REGOS, ANGERS, France*
*[2]University of Angers, Sfr Icat, Angers, France*

**Abstract Text**

**Background:** Long term immobilization induces a severe muscle wasting and bone loss. A physical and biochemical link between the two organs exists. The biochemical synchronization between bone and muscle is influenced by different factors of which nervous system network (both central and peripheral). The role of sympathetic nervous system in disuse osteoporosis model has been poorly investigated.

**Purpose:** Understand the influence of the sympathetic nervous system using a β2AR pathway antagonist and agonist on muscle atrophy and bone strength in a BTX immobilization model.

**Methods:** 24 female mice were spread into 3 groups. At day 0, all mice received Botulinum toxin (BTX) injection in the quadriceps to induce paralysis of the right hindlimb. Mice received daily intraperitoneal injection of physiological serum (Control-BTX group) or clenbuterol β2AR (adrenergic receptor) agonist solution at a dose of 2mg/kg (Clenbuterol-BTX group) or propranolol β2AR antagonist (propranolol-BTX group) solution at a dose of 2 mg/kg. Mice were sacrificed at 21days post-BTX.

Bone strength and quality were measured on femur by three points-binding, microCT, histomorphometry and Raman microscopy. Histology was performed on quadriceps. A one-way ANOVA was used to determined significant differences between groups.

**Results:** In control-BTX group, ultimate load and yield load were reduced (resp. 11,03%, $p<0.05$ and 28,2%, $p<0.05$) compared to the left side control femur. Neither propranolol nor clenbuterol had any effect on the two biomechanical parameters altered by BTX injection. Raman microscopy revealed an alteration of collagen maturity in control-BTX group which was significantly improved by propranolol (*p*<0.05).

**Conclusions:** The use of propranolol as an antagonist had no effect on the decrease in bone strength induced by BTX. The use of clenbuterol as a β2AR agonist confirmed the anabolic protective effect of the β2AR pathway on muscle atrophy but failed to compensate for the inhibitory effect of this agonist on bone formation.

**P173**

**Pilot study of bone turnover markers, diet, and exercise in elite female ballet dancers**

*Richard L Abel*[1]
[1]*Imperial, Surgery and Cancer, London, United Kingdom*

**Abstract Text**

**Introduction:** Elite ballet dancers face high rates of bone stress injuries despite engaging in substantial weight-bearing exercise. The paradoxical relationship between intense training and bone health suggests complex underlying mechanisms that are not fully understood.

**Purpose:** This pilot study investigated bone remodelling patterns in professional ballet dancers by examining the relationship between bone turnover markers, exercise intensity, and hormonal profiles compared to controls.

**Methods:** Five professional female ballet dancers from the Royal Ballet Company and six age-matched female controls participated in an 8-week study. Bone turnover was assessed through urinary NTX and serum P1NP markers. Oestrogen metabolism was evaluated via 2OH/16OH metabolite ratios. Dietary intake was tracked using 72-hour diaries, while exercise was monitored through 2-week activity logs.

**Results:** Dancers exhibited significantly higher bone resorption to formation ratios (NTX/P1NP) compared to controls ($p < 0.050$) (Fig.1) and elevated oestrogen metabolite ratios (2OH/16OH) ($p < 0.010$). These differences occurred despite similar dietary profiles between groups in fat ($p = 0.874$), carbohydrate ($p = 0.501$), and protein intake ($p = 0.099$). Dancers showed substantially higher weekly exercise hours (46.50 ±38.75 vs 14.75 ±11.75 hours/week, $p < 0.001$) and lower BMI (18.36 ±1.35 vs 20.77 ±3.66, $p = 0.020$).

**Conclusions:** This pilot study reveals that elite ballet dancers exhibit an imbalance in bone remodelling, favouring resorption over formation despite similar nutritional intake to controls. The combination of elevated NTX/P1NP ratios and altered oestrogen metabolism suggests a potential mechanism for increased bone stress injury risk. These findings support the development of bone turnover marker screening protocols for early identification of at-risk dancers.

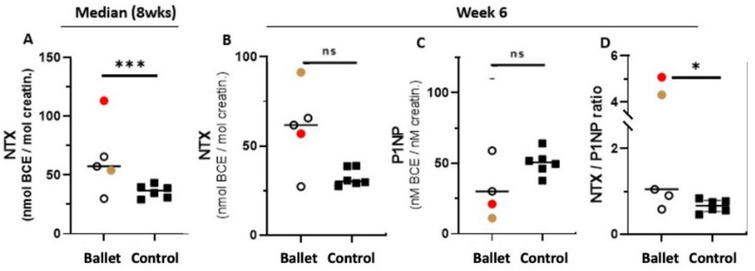

**Figure 1: Comparison of bone turnover markers between ballet dancers and controls.** NTX levels were measured over 8 weeks, while NTX/P1NP ratios were assessed at week 6. *p < 0.050, **p < 0.010, ***p < 0.001.

**ND-P010**

### The Association of Three-Dimensional CT-Derived Quantitative Parameters of Hip Muscle with Hip Refracture Risk in Older Women: A nnU-Net Deep Learning Approach

*Wenshuang Zhang*[1], *Wenhui Wang*[2], *Yajun Liu*[3], *Xiaoguang Cheng*[4], *Ling Wang*[4]
[1]*Beijing Jishuitan Hospital- Capital Medical University, Department of Radiology, Beijing, China*
[2]*Institute of High Energy Physics- Chinese Academy of Sciences, Beijing Synchrotron Radiation Facility, Beijing, China*
[3]*Beijing Jishuitan Hospital- Capital Medical University, Departmrnt of Spine Surgery, Beijing, China*
[4]*Beijing Jishuitan Hospital- Capital Medical University, Departmrnt of Radiology, Beijing, China*

### Abstract Text

**Background/Introduction:** Elderly patients face high mortality rates from secondary hip fractures, often due to falls linked to muscle function. Identifying muscle function biomarkers via imaging to predict refracture risk is crucial, but current methods based on single-plane imaging do not address the relationship between three-dimensional muscle parameters and refracture risk.

**Purpose:** This study develops a nnU-Net deep learning model for automatic three-dimensional segmentation of hip muscle in CT images to measure muscle volume and density, and explores the association between these parameters and refracture risk.

**Methods:** 447 elderly women (mean age:74.95±9.48years) with initial low-energy hip fractures were included, and 285 patients were prospectively followed up. A nnU-Net model was developed using 60 CT scans (40 for training, 10 for validation, and 10 for testing). Model accuracy was evaluated by comparing it with manual segmentation. Combined with threshold segmentation, four spatial parameters were quantified: total hip muscle volume(THMV), pure muscle volume(PHMV), total muscle density(THMD), and pure muscle density(PHMD). Cox regression analysis was used to explore the relationship between these parameters and refracture risk, comparing three-dimensional measurements with traditional single-plane methods.

**Results:** The median follow-up was 4.5years, with 206 patients(35 refractures,153 no refractures,18 deaths). The nnU-Net model achieved high accuracy(Dice coefficient=0.969), with strong correlation to manual segmentation($R^2$>0.997, *p*<0.001) and Bland-Altman data points within 95% limits of agreement. Multivariable Cox regression identified two high-risk segments: 15–17%(proximal, near the third sacral vertebra) and 39–41%(mid-to-proximal, higher than the top of the greater trochanter).Notably, the 39%–41% segment of PHMV(all HR=1.74, all *p*≤0.004), THMV(HR:1.62-1.63,all *p*=0.01), and the 15%–17% segment of PHMD(HR:1.53-1.63, all *p*≤0.03) demonstrated significant associations with refracture risk. These parameters showed stronger associations with refracture risk than traditional single-plane gluteus muscle density(HR=1.44,*p*=0.03).

**Conclusion(s):** The nnU-Net model enables efficient, accurate automatic segmentation of hip muscle CT images, with specific muscle parameters serving as sensitive predictors of refracture risk, outperforming traditional single-plane methods.

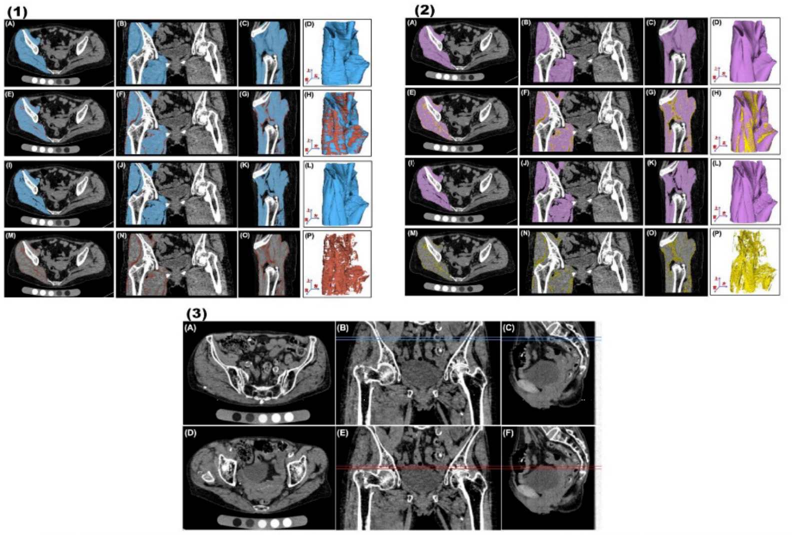

(1) Manual segmentation of hip muscle: (A – D), Total hip muscle by manual segmentation; (E – H), Threshold segmentation showing pure muscle (blue) and infiltrated fat (red); (I – L), Thresholded pure muscle (blue); (M – P), Thresholded infiltrated fat (red).
(2) nnU-Net automatic segmentation. (A – D), Total hip muscle segmented by nnU-Net; (E – H), Threshold segmentation showing pure muscle (purple) and infiltrated fat (yellow); (I – L), Thresholded pure muscle (purple); (M – P), Thresholded infiltrated fat (yellow).
(3) Anatomical locations of high-risk normalized segments: Axial, coronal, and sagittal views of the normalized (A – C), 15 – 17% segment (between blue lines) and (D – F), 39 – 41% segment (between red lines).

**ND-P012**

**Gain in Paraspinal Muscle Mass from High BMI Disappears in Older Adults**

*Wenkai Wu[1], Fangfang Duan[2], Renxian Wang[3], Dingding Wang[3], Anyi Guo[3], Ling Wang[4], Yajun Liu[1]*
*[1]Beijing Jishuitan Hospital- Capital Medical University, Department of Spine Surgery, Beijing, China*
*[2]Beijing Jishuitan Hospital- Capital Medical University, Clinical Epidemiology Research Center-, Beijing, China*
*[3]Beijing Research Institute of Traumatology and Orthopaedics- Beijing Jishuitan Hospital, JST sarcopenia Research Centre, Beijing, China*
*[4]Beijing Jishuitan Hospital- Capital Medical University, Department of Radiology, Beijing, China*

**Abstract Text**

**Objectives:** This study aimed to investigate the effects of body mass index (BMI) and central obesity (CO) on paraspinal muscle aging in Chinese community populations.

**Methods:** In this cross-sectional study, abdominal CT scans were collected and analyzed from 4,305 community-dwelling adults in China. Psoas and posterior paraspinal muscle index (PMI and PSMI) and density (PMD and PSMD) at the L3 level were measured. Multiple linear regressions and one-way ANOVA were performed for statistical analysis.

**Results:** Multiple linear regression analysis indicated that the paraspinal muscle index (PSMI and PMI) was primarily influenced by sex (β=–0.391 and –0.599, p<0.001) and BMI (β=0.442 and 0.371, p<0.001), followed by age and waist circumference (WC). In contrast, muscle density (PSMD and PMD) was mainly associated with sex (β=–0.405 and –0.317, p<0.001) and age (β=–0.409 and –0.429, p<0.001), slightly influenced by WC and BMI (Table 1).

**Conclusions:** This study identified sex and BMI as key determinants of paraspinal muscle mass, with BMI having a greater impact on muscle quantity than age. In contrast, muscle density was primarily influenced by sex and age. Notably, the muscle gain asssociated with higher BMI diminishes in older adults (Figure 1), highlighting the need to adjust for BMI in muscle mass analysis. The strong correlation between paraspinal muscle density and age suggests that muscle density may serve as a reliable marker of muscle aging.

Table 1. Results of multiple linear regression analysis for the impact of age, BMI, and WC on paraspinal muscle parameters

| **Outcome variable** | **Standardized beta coefficient** | | | |
|---|---|---|---|---|
| | **Sex** | **Age** | **BMI** | **WC** |
| **L3-PSMI** | -0.391$^{**}$ | -0.198$^{**}$ | 0.442$^{**}$ | -0.169$^{**}$ |
| **L3-PMI** | -0.599$^{**}$ | -0.090$^{**}$ | 0.371$^{**}$ | -0.192$^{**}$ |
| **L3-PSMD** | -0.405$^{**}$ | -0.409$^{**}$ | -0.005$^{\#}$ | -0.235$^{**}$ |
| **L3-PMD** | -0.317$^{**}$ | -0.429$^{**}$ | 0.050$^{*}$ | -0.170$^{**}$ |

Notes： **: P<0.001; *: P<0.05; #: P>0.05.

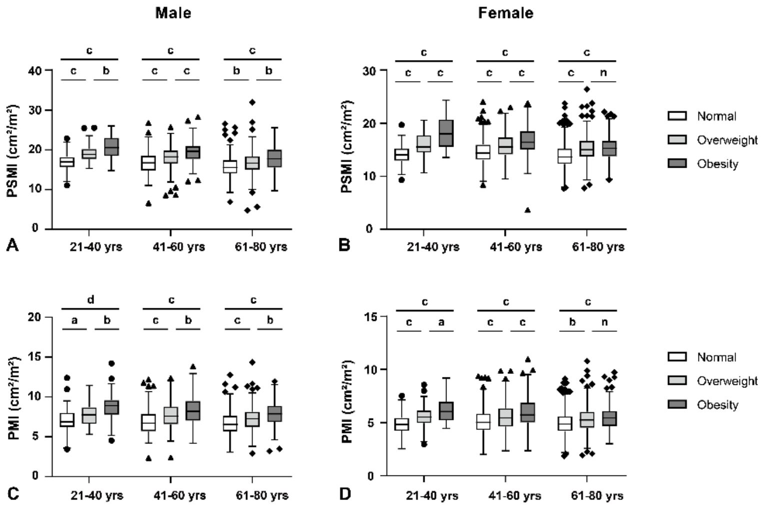

Figure 1. Distribution of L3-PSMI (A, B) and L3-PMI (C, D) across different age groups in Chinese adults.
Notes: a, P<0.05; b, P<0.01; c, P<0.0001; n, P>0.05.

**ND-P013**

**A novel fragility hip fracture risk calculator based on a mechanistic and stochastic modelling approach**

*<u>Christina Wapp</u>[1], Yvan Gugler[1], Paula Cameron[1,2], Alice Dudle[1], Daniela Frauchiger[3,4], Maria Papageorgiou[5], Emmanuel Biver[5], Serge Ferrari[5], Kurt Lippuner[3], Philippe Zysset[1]*
*[1]University of Bern, ARTORG Center for Biomedical Engineering Research, Bern, Switzerland*
*[2]ETH Zurich, Department of Mechanical and Process Engineering, Zurich, Switzerland*
*[3]Inselspital Bern University Hospital, Department of Osteoporosis, Bern, Switzerland*
*[4]University of Bern, ARTORG Center for Biomedical Engineering Researcdh, Bern, Switzerland*
*[5]Geneva University Hospitals and Faculty of Medicine, Division of Bone Diseases, Geneva, Switzerland*

**Abstract Text**

Fragility fractures of the hip in older adults are a major burden for the individual and society, as they result in increased morbidity, mortality and substantial health care expenditure. Most hip fractures are caused by falls resulting in an impact on the hip, inducing a force that exceeds the femoral bone strength. The risk of fracture depends on the rate of falling and the fall-induced impact force in relation to femoral bone strength. This work presents the framework of a novel personalised fragility hip fracture risk calculator based on a stochastic and mechanistic modelling approach, integrating the stochastic aspects of a fall together with a 1D dynamic mechanical model computing the impact force in the hip to calculate a one-year absolute fracture risk. The required input variables require solely anthropometric and densitometric data and can be refined using QCT images. Thus, the calculator does not depend explicitly but implicitly on epidemiological risk factors such as age, sex or ethnicity. A sensitivity analysis assessing the influence of the model's variables on the fracture risk was conducted, confirming that the fall rate, the trochanteric soft tissue thickness and the bone strength are the dominating factors influencing the risk of fragility fractures of the hip. Furthermore, output variables such as the predicted impact velocity and impact force aligned well with experimental data. Thus, the model is able to reflect observations from empirical data, indicating that it can capture the intrinsic aspects that affect the risk of fragility hip fractures.

**ND-P015**

**Prevalence of Sarcopenia and Its Association with Metabolic and Clinical Parameters in Patients with Type 2 Diabetes Mellitus**

*Subasri Bhuvaneswari Mohankumar*[1], *Vijayalakshmi Devarashetty*[1], *Geetha Aanantharamaiah*[1]
[1]*Bangalore medical college, Pharmacology, Bangalore, India*

**Abstract Text**

**Background:** Type 2 diabetes mellitus (T2D) is associated with an increased risk of sarcopenia. This study aims to assess the prevalence of sarcopenia and its association with clinical-metabolic parameters.

**Methodology:** Patients with T2D (≥1 year) aged >18 years were classified into three groups based on AWGS criteria: Group A (no sarcopenia), Group B (possible sarcopenia), and Group C (sarcopenia). Hand grip strength and skeletal muscle mass were assessed using a dynamometer and Bioelectrical Impedance Analysis, respectively

**Results:** mong 167 T2D patients, 52.1% were in group A, 32.9% in group B, and 15.0% in group C. Age, BMI, smoking, and alcohol intake were comparable across groups. Sarcopenia was associated with longer diabetes duration, higher HbA1c, larger abdominal circumference, hypertension, dyslipidemia, and diabetic kidney disease. Metabolic age and total body fat were significantly higher in sarcopenic patients (Table 1).

**Conclusion:** Sarcopenia was common in T2DM, linked to longer diabetes duration, poor glycaemic control, and higher cardiometabolic risk. Early identification and targeted interventions are essential to improve muscle health and metabolic outcomes in diabetes.

| Parameter | Total (n=167) | Group 1 (n=87) | Group 2 (n=55) | Group 3 (n=25) | p-value |
|---|---|---|---|---|---|
| **Age(years)** | 49.67±8.22 | 48.39±8.78 | 50.78±7.38 | 51.68±7.45 | 0.157 |
| **Sex(Male)** | 97(58.1%) | 38(43.7%) | 43(78.2%) | 16(64.0%) | <0.000 |
| **BMI** | 26.54±3.30 | 26.67±3.41 | 26.18±3.38 | 26.88± 2.76 | 0.1 |
| **Duration of DM (years)** | 7.61 ± 5.12 | 6.63 ± 4.46 | 6.33 ± 3.56 | 13.84 ± 5.84 | <0.001#$ |
| **HbA1c (%)** | 8.38 ± 1.77 | 7.47 ± 1.00 | 9.03 ± 1.65 | 10.14 ± 2.14 | <0.001*$ |

| Parameter | Total (n=167) | Group 1 (n=87) | Group 2 (n=55) | Group 3 (n=25) | p-value |
|---|---|---|---|---|---|
| **Abdominal Circumference (cm)** | 98.00±10.57 | 96.73±9.25 | 95.73±12.09 | 107.14±5.54 | <0.001#$ |
| **Hypertension** | 77(46.1%) | 28(32.2%) | 35(63.6%) | 14(56.0%) | 0.001 |
| **Dyslipidemia** | 78(46.7%) | 29(33.3%) | 27(49.1%) | 22(88.0%) | <0.001 |
| **Exercise** | 75(44.9%) | 53(60.9%) | 21(38.2%) | 1(4.0%) | <0.001 |
| **DKD** | 5(3.0%) | 0(0.0%) | 1(1.8%) | 4(16.0%) | <0.001 |
| **Duration of Exercise (min/day)** | 0(0,30) | 30 (0,30) | 0 (0,30) | 0 (0,0) | <0.001*#$ |
| **Total Body Fat (%)** | 33.05 ± 7.20 | 32.59 ± 7.28 | 30.27 ± 5.96 | 40.73 ± 3.04 | <0.001*# |
| **Metabolic age** | 57.04±10.63 | 55.86±11.83 | 56.16±9.65 | 62.96±5.44 | 0.005*# |

Group AvsB;#Group AvsC;$Group BvsC

**Poster Sessions: Osteoporosis: evaluation and imaging**

**P174**

**Comparison of Cancellous Bone Density from C3 to L5 in 11 Body Donors: CT Analysis versus Micro-CT Analysis**

*Guido Schröder[1], Estelle Akl[2], Justus Hillebrand[2], Andreas Goetz[3], Thomas Mittlmeier[4], Steffi S.I. Falk[4], Laura Hiepe[5], Julian Ramin Andresen[6], Reimer Andresen[7], Aenne Glass[8], Hans-Christof Schober[9]*
*[1]Sana Hospital Bad Doberan- Academic Teaching Hospital of the University of Rostock, Clinic for Orthopaedics and Trauma Surgery, Hohenfelde, Germany*
*[2]Institute for Diagnostic and Interventional Radiology- Pediatric and Neuroradiology, Rostock University Medical Center, Rostock, Germany*
*[3]Institute for Biomedical Technology, Rostock University Medical Center, Rostock, Germany*
*[4]Department of Trauma Surgery- Hand and Reconstructive Surgery, Rostock University Medical Center, Rostock, Germany*
*[5]Institute of Anatomy, Rostock University Medical Center, Rostock, Germany*
*[6]Clinical Department of Trauma Surgery, University Department of Orthopedics and Trauma Surgery, Vienna, Austria*
*[7]Institute of Diagnostic and Interventional Radiology / Neuroradiology, Westkuestenklinikum Heide- Academic Teaching Hospital of the Universities of Kiel- Luebeck and Ham burg, Heide, Germany*
*[8]Institute for Biostatistics and Informatics in Medicine and Ageing Research,*

*Rostock University Medical Center, Rostock, Germany*
[9]*Medical Office of Orthopedics and Osteology, OrthoCoast, Wolgast, Germany*

**Abstract Text**

**Introduction:** Comparative studies on Hounsfield units (HU) and bone volume fraction (BVF %) for the demonstration of cancellous bone density in the entire spine and in the various intravertebral regions are rare.

**Purpose:** This study aimed to measure HU in different spinal segments and planes (sagittal, axial, coronary), relate these measurements to micro-CT findings, and evaluate their significance regarding bone loss and fracture risk.

**Methods:** The spines of 11 body donors were analyzed using high-resolution spiral CT and micro-CT. Vertebral deformities were classified based on sagittal reformations. Cancellous bone density from C3 to L5 was measured in HU across all 242 vertebral bodies. For this purpose, a manually positioned ROI was established in mid-vertebral cancellous bone in the axial, sagittal and coronary planes.

A total of 726 specimens were extracted from prepared vertebrae from three quadrants (QI: right-sided edge, QII: central, QIII: left-sided edge) and analyzed with a micro-CT device. A general linear model was employed for repeated measurements, adjusting for age and body mass index.

**Results:** All spines exhibited osteoporotic characteristics. Both imaging techniques indicated a significant decrease in cancellous bone density from C3 to L5 ($p \leq 0.018$). Gender ($p = 0.001$) and fracture status ($p = 0.002$) influenced bone density, with men showing higher values and fewer fractures. CT measurements in the axial plane revealed higher density values compared to sagittal or coronary planes. The CT profiles along the spine were consistent across quadrants, while micro-CT results varied.

**Conclusion(s):** The study confirmed a craniocaudal reduction in bone density with varying clarity between the two methods. The differences in cancellous bone density based on gender, fracture status, and sectional planes highlight the need for improved understanding of each method's advantages for osteoporosis diagnosis. Future research should focus on establishing standard values for clinical applications in osteoporosis prevention and treatment.

**P175**

**Volumetric bone mineral density and bone microarchitecture are associated with joint ultrasound activity in rheumatoid arthritis**

*Olivier Malaise*[1], *Charline Rinkin*[1], *Caroline Gérard*[1], *Florane Chauveheid*[1], *Clio Ribbens*[1]
[1]*CHU Liège, Rheumatology, Liège, Belgium*

**Abstract Text**

**INTRODUCTION:** Osteoporosis is an important co-morbidity in rheumatoid arthritis (RA) that will lead to fractures and excess mortality. The objective of this work is to determine whether deteriorated areal / volumetric bone mineral density and bone micro-architecture are associated with higher disease activity, defined by joint ultrasound, which is more sensitive than clinical examination to detect synovitis.

**METHODS:** RA activity was studied in 61 patients, with evaluation by joint ultrasound (hands and wrists). Bone analyses included bone densitometry and peripheral bone micro-CT scan (HR-pQCT, tibia and distal radius), allowing to study bone structure in more detail (bone geometry, bone density and micro-architecture) and to distinguish trabecular and cortical areas.

**RESULTS:** Ultrasound activity of RA (number of joints with synovitis) is associated with lower bone density (studied with the peripheral bone micro-CT) (trabecular volumetric bone density, p=0.0072).

1. Ultrasound activity of RA (synovitis count, number of Doppler-positive joints, presence of tenosynovitis) is also associated with lower bone microarchitecture (trabecular bone percentage p=0.0031; span thickness p=0.026; cortical porosity p<0.001; cortical pore diameter p=0.015).
2. We studied the specific correlation between the distal radius area studied in HR-pQCT and the corresponding wrist ultrasound activity, to investigate a more local influence. The presence of ultrasound synovitis is associated with a lower trabecular volume density (p=0.012), a lower number of spans (p=0.046) and an increased cortical porosity (p=0.019).
3. Regarding the areal bone density, we observe an association only between T-score < -1 on the total hip and the number of ultrasound synovitis (p=0.0083).

**DISCUSSION:** HR-pQCT parameters, with lower bone volume density and altered microarchitecture. (organization of the spans, cortical porosity…) are associated with RA activity. Patients with RA ultrasound activity are more likely to have impaired bone quantity and quality and their bone health should be assessed as a priority.

**P177**

**Research on the Usefulness of Artificial Intelligence Osteoporosis Prediction Apps for Postmenopausal Women**

*Young Jun Won*[1], *Yu-mi Kim*[2], *Woo-ri Kim*[3]
[1]*Catholic Kwandong University College of Medicine Interantional St. Mary's Hospital, Department of Endocrinology and Metabolism, INCHEON, Korea- South*
[2]*International St. Mary's Hospital- Catholic Kwandong University College of Medicine-, Department of Endocrinology and Metabolism, INCHEON- South Korea, Korea- South*
[3]*International St. Mary's Hospital- Catholic Kwandong University College of Medicine-, Research Institute of Medical Data and Artificial Inteligence, INCHEON- South Korea, Korea- South*

**Abstract Text**

**Background/Introduction:** We previously conducted research using artificial intelligence (AI) to predict osteoporosis in postmenopausal women using data from the Korea National Health and Nutrition Examination Survey (KNHANES). This study developed a program to predict osteoporosis based on 20 features in 1,431 postmenopausal women.

**Purpose:** This study investigated the accuracy, precision, and reproducibility of this program in postmenopausal women using an existing application. We also assessed user satisfaction with the application.

**Methods:** 517 postmenopausal women participated in an application-based questionnaire to predict osteoporosis, with consent. We investigated 15 features identifiable through existing research questionnaires.

Model performance was evaluated using sensitivity, specificity, accuracy, and reproducibility, calculated using a confusion matrix. Deep learning models (Random Forest, AdaBoost, Gradient Boost) were trained and used for prediction, incorporating previous data. The application's ease of use was also assessed.

**Results:** Feature importance analysis and recursive feature elimination with cross-validation identified 10 key features from the initial 15 variables: age, age at menopause, weight, height, waist circumference, education level, peak height during youth, quality of life score, weekly muscle exercise frequency, and use of hormone replacement therapy (HRT) after menopause.

Using existing models, osteoporosis prediction achieved an accuracy of 0.84, precision of 0.87, and reproducibility of 0.91. Incorporating the new data into the existing model, the AdaBoost deep learning model achieved an accuracy of 0.896, precision of 0.672, recall of 0.608, and an area under curve(AUC) of 0.877.

A user survey on the application's ease of use showed 92.9% satisfaction, 4.8% dissatisfaction, and 2.3% uncertainty.

**Conclusion(s):** The application can aid in predicting osteoporosis in postmenopausal women. The results suggest that a bone mineral density (BMD) test should be considered if the application indicates a risk of osteoporosis, prior to definitive diagnosis following menopause.

**P179**

**Three-Dimensional HR-pQCT Bone-Image Synthesis of the distal radius with Generative Adversarial Networks**

*Gerald Degenhart[1], Christoph Angermann[2], Johannes Bereiter-Payr[1], Kerstin Stock[3], Markus Haltmeier[2]*
*[1]Medical University innsbruck, University Clinic for Radiology- Core Facility MicroCT, Innsbruck, Austria*
*[2]Universität Innsbruck, Department of Mathematics, Innsbruck, Austria*
*[3]Medical University innsbruck, Department of Orthopedics and Traumatology, Innsbruck, Austria*

**Abstract Text**

**Introduction and purpose:** The further development of the post processing of high resolution peripheral quantitative computed tomography (HR-pQCT) for bone health analysis is important to improve the quality of diagnosis in bonehealt. Limiting factors are the availability and the privacy concerns of patient data. Generally in the field of medical imaging, the problems of data availability and privacy hinder the rapid implementation of deep learning-based solutions in clinical routine. Generative models have great potential in medicine, as they allow the development of data-driven models on a much larger scale. This work demonstrates that three-dimensional generative adversarial networks (GANs) can be efficiently trained to generate high-resolution medical volumes with finely detailed architectures which enable development and testing of new methods and algorithm and patient independent data.

**Methods:** A dataset of a diverse cohort (Mean Age 53.2 years, youngest/oldest patient 18/91 60% female 40% male different bone qualities from healthy to severe osteoporosis) of HR-pQCT examinations obtained at the Core Facility MicroCT of the Medical University Innsbruck was used to train different GAN architectures (3D-ProGAN, 3D-StyleGAN) to obtain virtually generated volumetric image stacks of the distal radius. These images where then segmented for a binarised bone image and presented to experts for comparison. Further quality measures (Frechet Inception Distance (FID)) were applied and compared to the expert evaluation.

**Results and conclusion:** GAN networks could be trained which show no visible differences in the eye of the expert. The GANs are able to generate a high variability of realistic contours and bone qualities of the distal radius. Further we could show that the quality of the image could not be calculated by the FID and only the expert opinion is reliable. Using the expert opinion to select the most realistic images and calculating the relevant bone morphometrics new training and testing datasets can be obtained.

**P181**

### Improving vertebral fracture reporting frequency on CT scans; targeted audit and educational intervention or Hawthorne effect? The ADOPT study

*Jack Boylan[1], Jane Turton[1], Daniel Chappell[2], Jane Threlkeld[3], Madeleine Sampson[4], Cass Chisholm[3], Rachel Eckert[5], Elizabeth Curtis[4], Opinder Sahota[6], Kenneth Poole[2], Rafael Pinedo-Villanueva[7], Michael Stone[1], Kassim Javaid[7]*

*[1]Cardiff and Vale University Health Board, Bone Research Unit, Cardiff, United Kingdom*

*[2]NIHR Cambridge Biomedical Research Centre, Department of Medicine, Cambridge, United Kingdom*

*[3]Bradford Teaching Hospitals NHS Foundation Trust, Radiology, Bradford, United Kingdom*

*[4]University Hospitals Southampton NHS Foundation Trust, Radiology, Southampton, United Kingdom*

*[5]Oxford University Hospitals NHS Foundation Trust, Fracture Liaison Service, Oxford, United Kingdom*

*[6]Nottingham University hospitals NHS Trust, Department of Medicine for Older People, Nottingham, United Kingdom*

*[7]University of Oxford, Nuffield department of orthopaedics- rheumatology and musculoskeletal sciences, Oxford, United Kingdom*

**Abstract Text**

**Background:** 70% of osteoporotic Vertebral fractures (VF) remain undiagnosed. The ADOPT study is evaluating the patient and economic outcomes of VF detection on CT scans using artificial intelligence (AI) enabled Fracture Liaison Service identification.

The Hawthorne effect refers to individuals modifying their behaviour in response to their awareness of being observed; in this context, our radiology colleagues were aware of the ongoing ADOPT study.

**Purpose:** To evaluate reporting frequency of VF by radiologists on pre-existing CT scans before and after project initiation.

**Methods:** CT scans of adults aged 50 and over were analysed by Nanox-AI HealthVCF and then re-read by physicians with experience in identifying VF using the grade 2/3 semi-quantitative Genant score. VF identified by physicians were compared to VF reported by radiologists. CT scans from 2017 (pre-project) were analysed with AI and the results discussed in 2022 with our radiology colleagues with the objectives of the ADOPT study. Two subsequent re-reading of CT scans were conducted in October 2023 and October 2024. Proportions of concordance from AI flagged scans were compared using Chi-squared tests.

**Results:** 301 total AI analysed scans from 2017 were flagged by the AI. Physicians identified 42 VF (14.0%) of which 17 VF (41%) were identified by radiologists. 983 flagged scans from 2023 contained 68 (6.9%) VF with 55 (81%) identified by radiologists ($p<0.001$ compared with 2017). In 2024, 1201 flagged scans had 86 (7.2%) VF with 65 (76%) identified by radiologists, with no significant difference from 2023 ($p=0.6$).

**Conclusion:** Radiologists' true-positive reporting frequency increased significantly from before the AI project initiation and was maintained in the following year. This could be related to the educational presentation we provided, however increased reporting may be secondary to ongoing awareness of ADOPT. Of note, the hospital now has a musculoskeletal radiology lead aiming to improve VF reporting.

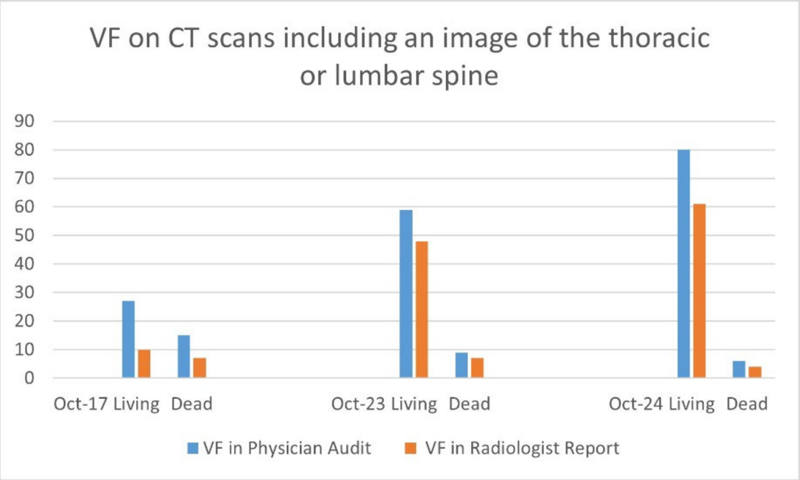

**P182**

### Estimating the impact of poor quality DXA scanning on patient care: A presentation on behalf of the ECTS Clinical Action Group.

*John Carey[1], Willem F Lems[2], Athanasios Anastasilakis[3], Maria P Yavropoulou[4], Julien Paccou[5], Anda M Naciu[6], Tim Rolvien[7], Stella D'Oronzo[8]*
*[1]University of Galway, School of Medicine, Galway, Ireland*
*[2]Amsterdam University Medical Center, Rheumatology, Amsterdam, Netherlands*
*[3]424 Military General Hospital-, Endocrinology, Thessaloniki, Greece*
*[4]First Department of Propaedeutic and Internal Medicine- Medical School- National and Kapodistrian University of Athens, Endocrinology, Athens, Greece*
*[5]University of Lille, Rheumatology, Lille, France*
*[6]Campus Bio-Medico University, Endocrinology and Diabetes, Rome, Italy*
*[7]University Medical Center Hamburg-Eppendorf, Trauma and Orthopaedic Surgery, Hamburg, Germany*
*[8]Section of Internal Medicine and Clinical Oncology- University of Bari Aldo Moro, Biomedical Sciences and Human Oncology-, Bari, Italy*

**Abstract Text**

**Background**: DXA scanning is widely used in the assessment of patients at risk of fracture, and the management of osteoporosis. DXA access receives considerable recognition, but attention to the quality of DXA services does not. Published reports suggest poor quality DXA harms patients, while experts suggest up to 50% of DXA scans have 1 or more errors. However, details of the impact these errors have on patient care is not well characterized. **Purpose**: To estimate the impact of poor quality DXA on the quality and cost of patient care.

**Methods**: We conducted a scoping literature review in Medline (Ovid) to estimate the most common errors and their impact on patient care from 2016 to 2023.

**Results**: 264 articles were identified in the initial search. Although several articles detailed the type and frequency of error, none evaluated their impact on post-DXA patient outcome or cost of care. To address this knowledge gap, we established a model using 6 patient cases which highlight frequently encountered clinical scenarios resulting from poor quality DXA. These highlight the impact on patients and enable estimations of the cost of subsequent care. Direct individual patient costs ranged from €1,488 to €41,392. If these events occurred in 1% of DXA scans, our data suggest the cost of poor quality care would be similar to the annual cost of providing DXA services nationally. If 10% of DXA scans resulted in such care, the costs would be significantly higher. In this presentation we will show the potential financial impact across several European countries on behalf of the ECTS clinical practice group.

**Conclusion**: Poor quality DXA has significant adverse effects on patient care and is very costly. DXA quality requires greater attention including education, audit and implementation research to incorporate best practice and improve standards in DXA services.

**ND-P016**

### Comparative Study of Phantom-less Quantitative CT and Phantom-based Quantitative CT in the Diagnosis of Osteoporosis

*Zi Tong Cheng*[1], *yandong liu*[2], *jian geng*[2], *Ling Wang*[2], *Wenshuang Zhang*[2], *Yi yuan*[2], *Bo Hu*[2], *Bowen zhang*[2], *kangkang ma*[2], *Fengyun zhou*[2], *Qingyu zhang*[2], *Dong yan*[2]

[1]*Beijing Jishuitan Hospital- Capital Medical University- National Center for Orthopaedics, Department of Radiology, beijing, China*

[2]*Beijing Jishuitan Hospital- Capital Medical University- National Center for Orthopaedics, radiology, beijing, China*

**Abstract Text**

**Background:** Osteoporosis is a disease characterized by low bone mass and microarchitectural deterioration of bone tissue, often leading to fragility fractures. Accurate measurement of bone mineral density (BMD) is crucial for the diagnosis of osteoporosis and prediction of fracture risk. Currently, PB-QCT is considered the gold standard for BMD measurement. However, the clinical value of PL-QCT, an alternative method without a phantom, remains controversial.

**Purpose:** Using phantom-based QCT (PB-QCT) as the gold standard, compare the accuracy of bone mineral density (BMD) measurement and osteoporosis diagnosis with phantom-less QCT (PL-QCT), and assess whether PL-QCT can replace PB-QCT. Secondly, to compare the differences between muscle and fat as the reference tissue for PL-QCT.

**Methods:** BMD measurements and osteoporosis diagnosis were compared in 298 middle-aged and elderly individuals (137 in the fracture group and 161 in the control group). They were recruited from patients undergoing QCT scans due to lumbar fractures and from community populations participating in low-dose CT lung cancer screening. BMD and osteoporosis variables were obtained using PB-QCT with QCT Pro (Mindways Software) and PL-QCT techniques with muscle and fat as reference tissues.

**Results:** PL-QCT measurements using fat as the calibration tissue exhibited higher correlation with PB-QCT (r=0.86–0.96 in males and r=0.91–0.96 in females) compared to those using muscle (r=0.51–0.85 in males and r=0.19–0.78 in females), with narrower limits of agreement. ROC curve analysis revealed that PL-QCT using fat as the calibration tissue performed better in diagnosing osteoporosis than using muscle. Although the average differences between BMD measurements using the fat and muscle references compared to PB-QCT were small, there was a large scatter in individual results.

**Conclusions:** These data showed that muscle as a reference tissue was inferior compared with fat for PL-QCT BMD measurements. This finding suggests that PL-QCT technology should not replace PB-QCT technology for the diagnosis of osteoporosis.

**Poster Sessions: Osteoporosis: pathophysiology and epidemiology**

**P183**

**Lycopene positively regulates osteoclast differentiation in RAW264.7 murine macrophage cells and osteoblast mineralization in Saos-2 cells**

*Cynthia Sithole[1], Trevor Nyakudya[1], Abe Kasonga[1]*
*[1]University of Pretoria, Physiology, Pretoria, South Africa*

**Abstract Text**

**Introduction:** Bone diseases, particularly osteoporosis, represent a significant global health challenge, accounting for the majority of fractures annually. Osteoporosis is characterized by decreased bone mineral density and an increased risk of fractures. Maintaining bone health requires a balance between the activities of bone-resorbing osteoclasts and bone-forming osteoblasts. Lycopene, a potent carotenoid, has been recognized for its potential preventive properties against bone-related disorders. This study aimed to examine the effects of lycopene on osteoclast and osteoblast differentiation *in vitro.*

**Methods:** Cell viability following lycopene treatment in RAW264.7 murine macrophage and Saos-2 cells was evaluated using resazurin assay. The effects of lycopene on osteoclast differentiation in RAW264.7 cells was assessed using tartrate-resistant acid phosphatase (TRAP) staining and TRAP activity. Osteoclastogenesis was induced by the receptor activator of nuclear factor-κB (NF-κB) ligand (RANKL) (15 ng/mL). The effects of lycopene on osteoblast mineralization were investigated using alizarin red staining, with osteoblastogenesis induced by media containing 50 mM ascorbic acid, 1 mM dexamethasone and 10 mM β-glycerophosphate. Osteoblastic gene (*Runx2*) expression was tested using quantitative polymerase chain reaction (qPCR).

**Results:** Lycopene exhibited no cytotoxic effects on RAW264.7 murine macrophage and Saos-2 cell viability. Lycopene significantly inhibited ($p < 0.0001$) osteoclast differentiation and reduced TRAP activity compared to cells treated with RANKL only ($p < 0.001$). Lycopene significantly increased osteoblast mineralization after day 14 and 21 ($p < 0.0001$) and upregulated *Runx2* gene expression after day 14 compared to cells treated with osteogenic media only ($p < 0.05$).

**Conclusions:** These preliminary findings demonstrate that lycopene inhibits osteoclast differentiation in a dose-dependent manner while enhancing osteoblast mineralization through increasing *Runx2* gene expression. Our findings suggest that lycopene may be beneficial for maintaining bone health. Further *in vivo* studies on the effects of lycopene on bone density are necessary to elucidate its potential benefits for bone health.

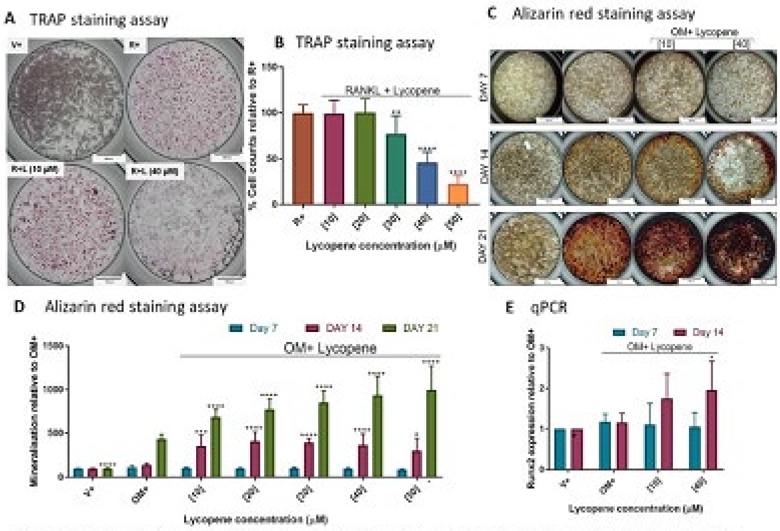

**Figure 1. A)** Lycopene decreased osteoclast formation with increasing concentrations. Scale bar = 500 µm. **B)** Lycopene (30-50 µM) significantly decreased the percentage cell counts relative to RANKL (R+) only treated cells. **C)** At 14 and 21 days, intensified red stained calcium granules were observed. Scale bar = 500 µm. **D)** Lycopene (10-50 µM) significantly increased osteoblast mineralization after day 14 and 21 compared to osteogenic media (OM+) only treated cells. **E)** Lycopene significantly increased *Runx2* gene expression after day 14 compared to OM+ cells.

**P184**

### Major osteoporotic fracture trends in Denmark 1997-2021

*Annika Kvist*[1]*, Vincent P. Visker*[2,3]*, Johanna H.M. Driessen*[2,3]*, Peter Vestergaard*[1,4,5]
[1]*Region North Denmark, Steno Diabetes Center North Denmark, Aalborg, Denmark*
[2]*Maastricht University Medical Center, Department of Pharmacy and Toxicology, Maastricht, Netherlands*
[3]*Cardiovascular Research Institute Maastricht CARIM- Maastricht University, Department of Clinical Pharmacy, Maastricht, Netherlands*
[4]*Aalborg University Hospital, Department of Clinical Medicine, Aalborg, Denmark*
[5]*Aalborg University Hospital, Department of Endocrionology, Aalborg, Denmark*

**Abstract Text**

**Introduction:** Fractures are a burden to the persons sustaining them, and to society. It has been estimated that 4.3 million fragility fractures occur each year in Europe with a cost of 56 billion euro. Fragility fractures have several risk factors, and with changes in population characteristics over time, fracture IRs are also likely to change over time. The aim was to investigate changes over time in fracture incidence rates in Denmark from 1997 to 2021.

**Methods:** We identified persons aged 18+ with a fracture in 1997-2021 in Denmark. Annual fracture incidence rates per 10,000 person years with corresponding 95% confidence intervals were calculated. Poisson regressions were estimated to test if the changes over time in fracture IRs were statistically significant.

**Results:** We identified 1,478,808 persons with fracture(s) in 1997-2021. 45.7% of the population were males, and the median (IQR) age was 55 (35-72) years. Clinical vertebral fracture IRs increased for men and women, while hip fracture IRs decreased for women but remained stable for men. Humerus and forearm fractures were stable for both men and women, but with more fluctuation for women than men.

**Conclusion:** The decrease of hip fracture IRs in women is interesting and important, although there remains a gap between men and women for both hip, humerus, and forearm fractures.

**P185**

**Metabolomics profiling of bone fragility in older women – the MICROBONE study**

*Pawel Szulc*[1]*, Karim Chikh*[2]*, Oriane Vitalis*[2]*, Roland Chapurlat*[3]

[1]*INSERM UMR 1033- University of Lyon- Hospices Civils de Lyon, Epidemiology of Osteoporosis, Lyon, France*

[2]*CARMEN INSERM U1060- INRA U1397- University of Lyon- Hospices Civils de Lyon, Laboratory of Biochemistry and Molecular Biology, Lyon, France*

[3]*INSERM UMR 1033- University of Lyon- Hospices Civils de Lyon, Department of Rheumatology, Lyon, France*

**Abstract Text**

**Background:** Fragility fractures are a major health problem in older women, but mechanisms underlying low bone mineral density (BMD) and poor bone strength are only partly studied. Interestingly, small molecules, including lipids, may regulate bone metabolism.

**Purpose:** To identify metabolites associated with low BMD or prior fragility fracture in older women by metabolomics profiling.

**Methods:** Four groups of 20 women aged >60 each were examined:

A (reference): BMD T-score>–1.5 at the lumbar spine, total hip and femoral neck (all three), no prior fracture;

B: BMD T>–1.5 and prior fragility fractures (vertebral, peripheral);

C: osteoporosis (T-<–2.5, at least one site) without fracture;

D: osteoporosis and prior fragility fractures.

Mass spectrometry–based multi-targeted metabolomics was performed on serum samples. All analytes were screened using Kruskal-Wallis test and Benjamini-Hochberg false discovery rate procedure. Selected metabolites were evaluated by analysis of covariance adjusted for age, BMI, smoking and physical activity.

**Results:** Women with osteoporosis (C&D) had significantly higher concentrations of four glycosylceramides, six phosphatidylcholines, and two phosphatidylethanolamines, but lower concentrations of glutamate, leucine and isoleucine than women with T-score>–1.5 (A&B) ($p<0.05$).

In the women with fractures (B&D) concentrations of eight lysophosphatidylethanolamines, three phosphatidylglycerols and six phosphatidylinositols were higher vs. women without fracture (A&C) ($p<0.05$ to $<0.001$). Certain ceramides, five phosphatidylcholines and three lysophosphatidylethanolamines were higher in the Group D vs. A ($p<0.01$ to $<0.005$).

Women with osteoporosis and/or fractures (B&C&D) had higher concentrations of eleven phosphatidylglycerols, two lysophosphatidylethanolamines, and three phosphatidylinositols vs. Group A ($p<0.05$ to $<0.001$).

Concentrations of eleven triglycerides and of two diacylglycerols were significantly higher ($p<0.05$) in the Group B (fracture despite normal BMD) vs. Group C (low BMD, but no fracture).

**Conclusion:** Metabolomics profiling may help identify new metabolic pathways determining bone mass and strength in older women (particularly at the level of the metabolism of sphingolipids and glycerophospholipids) and contribute to the development of new markers of the fracture risk.

**P186**

**FGF23 and bone microstructure in βIVSII-654 thalassemia mice: A study across adulthood and middle age**

*Kannikar Wongdee[1], Ratchaneevan Aeimlapa[2], Pathnaree Wattano[3], Saovaros Svasti[4], Narattaphol Charoenphandhu[2]*
*[1]Burapha University, Faculty of Allied Health Sciences, Chonburi, Thailand*
*[2]Mahidol University, Department of Physiology- Faculty of Science, Bangkok, Thailand*
*[3]Mahidol University, Center of Calcium and Bone Research COCAB- Faculty of Science, Bangkok, Thailand*
*[4]Mahidol University, Institute of Molecular Biosciences, Nakhon Pathom, Thailand*

**Abstract Text**

Thalassemia not only induces anemia and iron overload but is also associated with iron-induced aberrant bone turnover and calcium and phosphorus homeostasis dysregulation. However, the microstructural changes occurring during adulthood and middle age in $+/\beta^{IVSII-654}$ mutant mice remain unclear. It is hypothesized that the levels of hormones—including FGF23, which plays roles in bone and iron metabolism—are altered under thalassemic conditions. Here we aimed to investigate the microstructural bone changes and the levels of iron- and calcium-regulating hormones in mature adults (16-week-old) and middle-aged (28-week-old) wild-type (WT) and $+/\beta^{IVSII-654}$ mice. Female C57BL/6 WT and hemizygous $\beta^{IVSII-654}$ (Thal) mice were used in this study. Each strain was divided into two groups, i.e., mature and middle-aged adults. The tibiae were analyzed for bone microstructure using *in vivo* ultrahigh-resolution µCT. Blood samples were collected to analyze complete blood count (CBC), as well as iron- and calcium-regulating hormones. This study was approved by Animal Ethics Committee of Faculty of Science, Mahidol University. The CBC analysis confirmed that Thal mice exhibited anemia and anisopoikilocytosis. Cortical parameters revealed no significant differences in cross-sectional area or thickness of the cortical envelope between WT and Thal mice in both age groups. At the trabecular sites, the trabecular thickness and connectivity tended to be lower in the

Thal group. Serum ferritin levels in 28-week-old Thal mice were elevated by approximately twofold. Hormonal analysis revealed that intact FGF23 levels were significantly higher in Thal mice compared to WT mice, whereas $1,25(OH)_2D_3$ levels were apparently similar. In conclusion, from adulthood to middle age, Thal mice were able to maintain the cortical envelope structure despite having erythrocyte breakdown and anemia. In this context, the elevated FGF23 level might act as a protective factor since it has been reported to exert hypoferremic actions that possibly alleviate iron toxicity on bone.

**P187**

**The impact of hip fracture on survival, disability, pain and health-related quality of life in Zimbabwe: a prospective cohort study**

*Celia Gregson[1], Mohamad Nasser[1], Anya Burton[1], Hannah Wilson[1], Tadios Manyanga[2], Prudance Mushayavanhu[3], Munyardardzi Ndekwere[4], Joseph Chipanga[2], Samuel Hawley[1], Simon Graham[5], James Masters[5], Kate A Ward[6], Matthew Costa[5], Rashida A Ferrand[7]*

*[1]University of Bristol, Musculoskeletal Research Unit, Bristol, United Kingdom*

*[2]Biomedical Research and Training Institute, The Health Research Unit Zimbabwe, Harare, Zimbabwe*

*[3]Sally Mugabe Central Hospital, Department of Surgery, Harare, Zimbabwe*

*[4]Midlands State University, Department of Surgery, Gweru, Zimbabwe*

*[5]University of Oxford, Oxford Trauma and Emergency Care- Nuffield Department of Orthopaedics, Oxford, United Kingdom*

*[6]University of Southampton, MRC Lifecourse Epidemiology Centre, Southampton, United Kingdom*

*[7]London School of Hygiene and Tropical Medicine, Clinical Research Department, London, United Kingdom*

**Abstract Text**

**Background:** Fragility fracture risk is increasing as longevity increases in Africa.

**Purpose:** We assessed one-year health outcomes following hip fracture in older adults in Zimbabwe.

**Methods:** A cohort of adults ≥40 years with hip fracture, presenting to hospitals in Harare (2 public; 5 private) (2021-22) were followed-up for one-year. Operative management, survival, health-related quality of life (HRQoL) utilities from EQ-5D-5L, disability (WHODAS) and pain were quantified. Associations between patient characteristics and odds of operative management were assessed.

**Results:** Of the 196 hip fracture patients (49% female and median age 74·0 years [IQR 63·0-83·0]), 86·2% were fragility fractures. 173 (88·3%) were managed in the public hospitals, where just 96 (55·5%) received operative hip fixation; all 23 (11·7%) managed in private facilities received an operation. After one-year, 54 (28·2%) had died (40·3% of those aged ≥70 years, and 8·3% of those <70 years). In public hospitals, 40% of non-operated patients died, versus 19·4% of those operated.

By 30-days the mean HRQoL utility score had fallen by 64·1% from 0·81[95%CI 0·79,0·82] pre-fracture to 0·29[95%CI 0·25,0·33]. Minimal recovery was seen after 120-days. Larger reductions occurred in those age ≥70 years (Figure-1). Post-fracture disability was almost universal, with only 2% disabilityfree by 365-days, when 97% still reported hip injury-related pain, with 94% experiencing pain interfering with walking, and 80% with sleep. Higher household income was associated with higher odds (OR=3.05 [95%CI 1.54,6.05]), and malnutrition with lower odds (OR=0·31 [95%CI 0·14,0·65]), of being operated.

**Conclusions:** Substantial health inequities in the provision of hip fracture care have been identified by these unique data. Following hip fracture in Zimbabwe, quality-of-life and survival fall substantially, more so than seen after stroke in other regions, demanding implementation of guidelines to standardise care and improve operative capacity to manage the predicted rise in fractures.

**Figure-1:** HRQoL prior to hip fracture and over 12 months

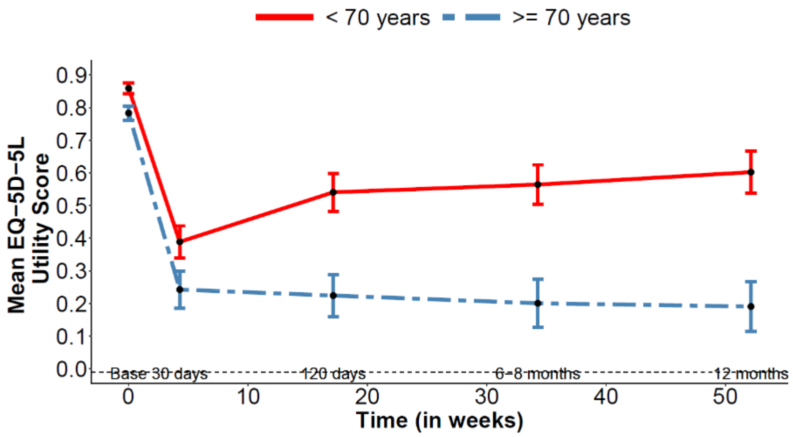

**P188**

**Inhibition of osteoclastogenesis and multinucleated osteoclast formation through modulation of DPP-4, Piezo1 and TRPV6**

*Narattaphol Charoenphandhu*[1], *Ratchaneevan Aeimlapa*[2], *Jiraporn Panmanee*[3], *Kannikar Wongdee*[4]

[1]*Mahidol University, Center of Calcium and Bone Research COCAB- Faculty of Science, Bangkok, Thailand*

[2]*Mahidol University, Department of Physiology- Faculty of Science, Bangkok, Thailand*

[3]*Mahidol University, Institute of Molecular Biosciences, Nakhon Pathom, Thailand*

[4]*Burapha University, Faculty of Allied Health Sciences, Chonburi, Thailand*

**Abstract Text**

Osteoclastogenesis and the fusion of mononuclear precursor cells rely on the functions of various membrane proteins, such as RANK, CSF-1R/CD115 (M-CSF receptor), and calcitonin receptor. Besides cytokine receptors, certain proteins may have specific functions—for instance, the cleavage of local chemokines/cytokines, mechanoreception, and regulation of cellular calcium metabolism (e.g., DPP-4, Piezo1 and TRPV6, respectively)—crucial for osteoclastogenesis and osteoclast-mediated bone resorption. Therefore, modulators of these three proteins are hypothesized to suppress osteoclastogenesis and could potentially be used as antiresorptive agents. Herein, we aimed to investigate whether DPP-4, Piezo1, and TRPV6 were expressed in primary mouse osteoclasts, and whether inhibition of these membrane proteins could suppress multinucleated osteoclast formation. This study has been approved by the Animal Ethics Committee of the Faculty of Science, Mahidol University. Long bones were collected from C57BL/6 mice for primary osteoclast culture. Successful multinucleated osteoclast culture was evaluated optically using Tomocube holotomography, based on the refractive index of cellular components. Mononuclear precursor cells or osteoclasts were treated with omarigliptin (DPP-4 inhibitor), Yoda1 (Piezo1 inhibitor), and a newly synthesized TRPV6 inhibitor. Multiple-group comparisons were performed using one-way ANOVA. Primary mouse osteoclasts were found to express DPP-4, Piezo1, and TRPV6. Molecular docking and molecular dynamics analyses indicated strong interactions between the tested small molecules and target proteins, including TRPV6. Under normal conditions, the cell volume of osteoclasts was positively correlated with the number of nuclei, as determined by holotomography. TRAP staining revealed that omarigliptin, Yoda1, and TRPV6 inhibitors reduced the number of multinucleated osteoclasts, indicating the suppression of osteoclastogenesis even in the presence of RANKL. In conclusion, osteoclastogenesis and multinucleated osteoclast formation were dependent on the enzyme DPP-4, the mechanoreceptor Piezo1, and the calcium channel TRPV6. Thus, inhibitors of these membrane proteins may serve as therapeutic antiresorptive drugs for osteoporosis or other diseases with accelerated bone resorption.

**P189**

**Investigation of the bone phenotype of CD274 knockout mice**

*Claudia Neunaber[1], Hadi El Assaad[2], Stefan Lienenklaus[3], Marina Komrakova[4], Katrin Bundkirchen[1]*
*[1]Hannover Medical School, Department of Trauma Surgery, Hannover, Germany*
*[2]University of Leipzig Medical Center, Department of Orthopaedic- Trauma and Plastic Surgery, Leipzig, Germany*
*[3]Hannover Medical School, Institute of Laboratory Animal Science, Hannover, Germany*
*[4]University Medical Center Göttingen,*
*Department of Trauma Surgery- Orthopaedics and Plastic Surgery, Göttingen, Germany*

**Abstract Text**

**Background/Introduction:** Osteoporosis is a serious bone defect in the elderly population, who suffer from fractures due to the altered microarchitecture of the osteoporotic bone. Previous studies in 8 week old mice suggest that Programmed Death Ligand 1 (gene name Cd274) may play an important role in osteoporosis by exerting an inhibitory effect on the disease, thereby maintaining better bone density.

**Purpose:** The aim of this study was to evaluate the bone phenotype of adult Cd274 knockout (KO) mice compared to wild-type mice.

**Methods:** Femora, tibiae and lumbar vertebrae were collected from Cd274 KO and wild-type mice (males/females, aged 26-34 weeks). MicroCT scans assessed density and morphometry of bones. Via biomechanical 3-point bending test the bones' ability to resist external stress was determined. The organic and inorganic bone composition was evaluated by ashing analysis. Histology was performed to analyze  bone cells. The unpaired T-test with a significance level of $p<0.05$ was used for statistical analysis.

**Results**: Compared to controls, Cd274 KO long bones showed in microCT scans an increase in cortical volume (WT 3.37 $mm^3$ vs. KO 3.92 $mm^3$, $p<0.001$), and a higher stiffness (WT: 51.37 N/mm vs. KO 82.41 N/mm, p=0.0006) in biomechanical testing. Histology revealed a lower amount of osteoclasts in Cd274 animals (WT 33 cells vs. KO 26 cells, p=0.44) as well as a higher amount of osteocytes (866 WT vs. 1102 KO) compared to wild-types.  Ashing showed a higher anorganic amount in CD274 KO compared to wild-types (WT: 35.69% vs. KO: 40.08%, p=0.038). Similar results were seen for lumbar vertebrae.

**Conclusion**: The findings showed that Cd274 KO mice exhibit higher bone density and increased resistance to external forces. Thus, PDL1 may contribute to the loss of cortical and trabecular bone, leading to lower bone density and quality, contradicting previous assumptions of its inhibitory role in osteoporosis.

**P191**

**Predicting Imminent Risk of Major Osteoporotic Fractures through Unsupervised Cox Analysis of Medication Exposure Windows in Adults without Osteoporosis**

*Marlene Rietz[1,2], Sören Möller[3], Jan Brønd[4], Katrine Hass Rubin[3], Bo Abrahamsen[3,5]*
*[1]Department of Clinical Research- University of Southern Denmark, Research Unit OPEN-, Odense, Denmark*
*[2]Karolinska Institutet, Division of Clinical Physiology- Department of Laboratory Medicine, Huddinge, Sweden*
*[3]Affiliation 1, as above, Odense, Denmark*
*[4]Centre of Research in Childhood Health- University of Southern Denmark,*
*Research Unit for Exercise Epidemiology- Department of Sports Science and Clinical Biomechanics, Odense, Denmark*
*[5]Holbæk Hospital and University of Southern Denmark, Medicine, Holbæk, Denmark*

**Abstract Text**

**Background:** Medications can affect skeletal strength and the risk of falls and fractures. The date of last redemption (DLR) of a medication may help differentiate associations as short- or long-lasting, or simply as a marker of chronic disease.

**Purpose:** To evaluate the potential of using simple cox-proportional hazards analysis to identify key medications relevant for  case finding for imminent fracture risk and in screening already marketed medications for potential to reduce fracture risk.

**Methods:** This analysis included 2,468,247 individuals age 45 or over. We used national prescription data to identify the DLR of any medication, predicting fractures in 2022. The DLR was divided into three windows of recency (<1y, 2-5y and 6-15y). Cox- analysis, adjusted for age and sex, were performed for medications with >1 available DLR and case in each exposure window, at significance level of $P_{adj} < 0.001$.

**Results:** We present a Volcano plot for recent medication use (<1y prior to outcome ascertainment) demonstrating the estimated HR ($log_{10}[HR]$) on the x-axis and statistical significance ($log_{10}[P_{adj}]$) along the y-axis, (Fig 1A). ATC codes (level 4) with significant positive associations with fracture risk are labelled as points with call-outs in the scatterplot. For example, N02AA (morphine and other natural opium alkaloids) demonstrates a highly significant positive association with limited risk increase (HR 2.1, $N_{exp}$=115,174), while A10BG (glitazones) has a high HR but low prevalence (HR 6.6, $N_{exp}$=95). Next, a heatmap for medications with >1 statistically significant association shown across time windows demonstrates unique temporal patterns (Fig 1B). Using hierarchical clustering, medications with similar DLR dynamics were grouped.

**Conclusion:** This approach may be useful in identifying less commonly used prescription medications that are associated with increases, or more rarely, decreases in imminent fracture risk. Resulting resources may help distinguish between transient (e.g. reversible bone loss or immediate fall risk) or long-term associations (e.g. disease proxy).

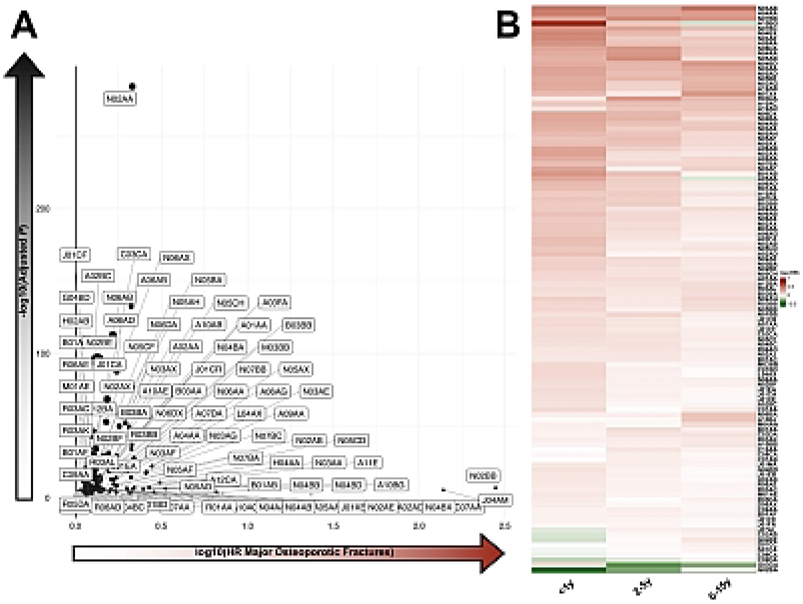

**P192**

**Fracture and cancer history of Atypical Femoral Fracture cases – Are non-bisphosphonates users more prone to non-classical osteoporotic fractures or cases of cancer?**

*Kasper Westphal Leth*[1], *Benjamin Bakke Hansen*[1], *Rick Dell*[2], *Bo Fan*[3], *Anne Grethe Jurik*[4], *Jens Brøndum Frøkjær*[5], *Mikael Ploug Boesen*[6], *Christopher D. Smith*[7], *Eric Vittinghoff*[8], *Katrine Hass Rubin*[1], *Douglas C. Bauer*[8,9], *Dennis M. Black*[8], *Bo Abrahamsen*[7,10]
[1]*Research Unit OPEN,*
*Department of Clinical Research - University of Southern Denmark and Odense University Hospital,*

*Odense, Denmark*
*[2]Kaiser Permanente Southern California, Kaiser Permanente Southern California, Downey, United States*
*[3]University of California, Department of Radiology and Biomedical Imaging, San Francisco, United States*
*[4]Aarhus University, Department of Radiology, Aarhus, Denmark*
*[5]Aalborg University Hospital, Departments of Radiology and Clinical Medicine, Aalborg, Denmark*
*[6]Copenhagen University Hospital, Department of Radiology, Bispebjerg and Frederiksberg, Denmark*
*[7]University of Southern Denmark, Department of Public Health, Odense, Denmark*
*[8]University of California, Department of Epidemiology & Biostatistics, San Francisco, United States*
*[9]University of California, Department of Medicine, San Francisco, United States*
*[10]Research Unit OPEN,*
*Department of Clinical Research - University of Southern Denmark - Odense University Hospital and H olbæk Hospital, Odense, Denmark*

**Abstract Text**

**Background/Introduction:** We hypothesized that underlying rare bone diseases or cancer might be more common among individuals who did not use bisphosphonates but still had an Atypical Femoral Fracture (AFF).

**Purpose:** To examine whether people with AFF who are not using bisphosphonate (non-BP-users) have a history of more non-classical osteoporotic fractures or cases of cancer compared with those who had AFF while using bisphosphonate (BP-users).

**Methods:** Danish descriptive study with radiology review of AFF in the period 2010 to 2014; all 189 AFF in the country were included (Women: 149, Men: 40). Non-classical osteoporotic fractures were defined as non-Major Osteoporotic Fractures (non-MOF), excluding fractures of the face, fingers, and toes, categorised as single-site or multiple-sites where two non-MOF had to be in different locations. Fracture and cancer history were recorded prior to the AFF. P-values were calculated using the chi-squared test.

**Results:** Fracture and cancer history is shown in Table 1. No statistically significant difference was found in fracture history between the two groups. However, a significant difference in cancer history was found for the non-BP-users (37.3% vs. 21.5%, p=0.02). This difference was primarily due to more cases of lung cancer. However, most were cancer-free, with no metastases suspected based on X-ray adjudication.

| **Fracture history (non-MOF)** | AFF (Non-BP-users) n=59 | AFF (BP-users) n=130 | **p-value** |
|---|---|---|---|
| Prior, single site | 6 (10.2%) | 28 (21.5%) | 0.06 |
| Prior, multiple sites | 3 (5.1%) | 10 (7.7%) | 0.51 |

**Cancer history**

| | | | |
|---|---|---|---|
| Lung, breast, prostate or colorectal | 15 (25.4%) | 16 (12.3%) | 0.02 |
| Other | 7 (11.9%) | 12 (9.2 %) | 0.58 |
| Total number of participants with cancer | 22 (37.3%) | 28 (21.5%) | 0.02 |

**Conclusion:** The AFFs in the study population cannot be explained by rare bone diseases, but there is a slight overrepresentation of cancer patients in the non-BP-users compared to the BP-users.

Funded by the National Institute of Health (US) R01AR082562

**P193**

**Identifying the Fracture Threshold in Glucocorticoid Induced Osteoporosis by means of a Regional BMD Database Linked to National Pharmacy and Fracture Data**

*Benjamin Bakke Hansen[1], Line Møller Nanque[1], Catharina Vind Nielsen[2,3], Anne Pernille Hermann[2], Katrine Hass Rubin[1], Bo Abrahamsen[1,4]*
*[1]Research Unit OPEN - Odense University Hospital,*
*Department of Clinical Research - University of Southern Denmark, Odense, Denmark*
*[2]Department of Endocrinology - Odense University Hospital,*
*Department of Clinical Research - University of Southern Denmark, Odense, Denmark*
*[3]Department of Diabetes and Endocrinology,*
*Esbjerg Sygehus - University Hospital of Southern Denmark, Esbjerg, Denmark*
*[4]Department of Medicine 1, Holbæk Hospital, Holbæk, Denmark*

**Abstract Text**

**Background:** Systemic glucocorticoid (GC) treatment has profound direct and indirect skeletal effects but guidelines do not agree on the appropriate intervention threshold. Fractures are expected to occur at a different (elevated) bone mineral density (BMD) threshold following GC treatment but this has not been firmly established. In 1992, Melton et al suggested defining a fracture threshold in osteoporosis as the 90$^{th}$ percentile of BMD observed in fracture cases but to our knowledge this has never been tested in a GC setting.

**Purpose:** Estimate the BMD treshold following GC treatment in adult men and women

**Methods:** Nested analysis of BMD and recent Major Osteoporotic Fracture (MOF) events in a regional BMD database (40,562 men and women) who had not been treated with osteoporosis medications. Data was enriched with national register data on hospital events and pharmacy records.

**Results:** In women, the fracture threshold (see table 1 for g/cm2 values) was elevated by only 0.14 T-score units for the hip and 0.11 units at the spine. In men the threshold was lowered by 0.68 units at the hip with no difference at the spine.

**Table 1. Fracture thresholds (90th percentile of BMD and 95% CI))**

| BMD Site | GC Exposure | No GC Exposure |
|---|---|---|
| Spine BMD (all) N=3,836 | 1.112 (1.095-1.129) | 1.103 (1.096-1.111) |
| Total Hip BMD (all) N=4,487 | 0.937 (0.922-0.951) | 0.949 (0.943-0.956) |
| Spine BMD (Women) N=2,852 | 1.087* (1.069-1.106) | 1.075 (1.067-1.084) |
| Total Hip BMD (Women) N=3,337 | 0.924* (0.908-0.941) | 0.907 (0.900-0.914) |
| Spine BMD (Men) N=984 | 1.172 (1.133-1.211) | 1.170 (1.154-1.186) |
| Total Hip BMD (Men) N=1,150 | 0.967* (0.938-0.997) | 1.050 (1.033-1.067) |

**p < 0.05*

**Conclusion:** The BMD threshold for MOF is slightly elevated in women but not men following systemic GC exposure. The apparent gender discrepancy may be influenced by a smaller sample of men. The proposed approach of using the 90th percentile for BMD in fracture cases to derive a fracture threshold has limitations which will be discussed.

**P194**

**Bone mineral density and hand grip strength in postmenopausal females with forearm fractures**

*Ivan Stets[1], Anna Musiienko[1], Nataliia Grygorieva[1]*
*[1]D.F. Chebotarev Institute of Gerontology of the National Academy of Medical Sciences of Ukraine, Department of Clinical Physiology and pathology of the musculoskeletal system, Kyiv, Ukraine*

**Abstract Text**

**Backgrounds:** Forearm fractures (FFs) are an essential type of osteoporotic fracture that increases the risk of future low-energy fractures and can lead to temporary or permanent disability. Previous studies demonstrated poor bone health in this category, but knowledge about their long-term functional capabilities is limited.

**Objective**: The study aimed to evaluate bone mineral density (BMD) and physical function (hand grip strength) in postmenopausal women depending on previous FFs.

**Material and Methods:** 150 postmenopausal women with an average age of 66.9±8.5 years old were examined and divided into two groups. The first included 75 females without any history of fractures, while the second group comprised 75 women with previous FFs. The average age of FFs was 57.1±10.1 years, with a mean duration since fracture of 10 years. BMD and T and Z indices of the lumbar spine, hip, femoral neck, and radius were measured using dual-energy X-ray absorptiometry (Hologic Discovery WI). Hand grip strength was assessed using a spring hand dynamometer for both hands.

**Results:** The results indicated that subjects did not differ by age. However, women with FFs had significantly lower body mass, body mass index, earlier age of menopause and more extended postmenopausal period compared to persons without fractures. BMD indices at the lumbar spine, hip, femoral neck, and radius were significantly lower in women with FFs. While no differences in BMD were observed between the fractured and non-fractured forearms (0.535±0.089, and 0.538±0.090 g/cm$^2$, respectively, 0.622±0.060 g/cm$^2$ for the first group), hand grip strength on the fractured side was significantly lower compared to the uninjured side (respectively 16.0 [12.0-20.0] and 18.0 [15.0-23.0] kg, p<0.001).

**Conclusion:** Women with previous FFs exhibited poorer BMD and reduced muscle strength in the fractured arm, even years after the injury. These findings emphasize the long-term impact of FFs on musculoskeletal health in postmenopausal women.

**P195**

**Patients with periprosthetic femoral fractures are frail elderly patients who are commonly diagnosed with osteoporosis**

*Victoria Houel*[1]
[1]*University Hospital of Lille, Nord, Lille, France*

**Abstract Text**

**Introduction:** To compare patients presenting with periprosthetic femoral fractures (PFF) to patients with native hip fractures with a special focus on bone mineral density (BMD) measurements, in order to reinforce the hypothesis that PFF are osteoporotic fractures.

**Methods:** A retrospective, single-centre, observational study of all patients aged ≥ 50 years with low-energy PFF identified at the Lille University Hospital from January 1, 2016, to December 31, 2022, was conducted. The PFF group was compared to a control group of patients with native hip fractures hospitalized during the same period. To compare the T-scores data, we used a linear mixed model that considered a predefined adjustment for age, sex, and BMI. Adjusted means ± standard error of the mean (SEM) are derived from the mixed model.

**Results:** Among 71 patients with PFF (78.9% female, median (IQR) age 81 (72–88) years), osteoarthritis (57.8%) was the primary indication for hip surgery. Compared with the control group (n=117), patients in the PFF group were significantly older (p=0.002), had a significantly greater BMI (p=0.043), and had a higher risk of multiple falls (54.3% vs. 26.1%, p<0.001). The risk factors for osteoporosis included a greater frequency of previous low-energy fractures (69.0% vs. 44.0%, p<0.001) and an increased prescription of anti-osteoporosis medications (26.8% vs. 11.1%, p=0.006) in patients with PFF. Adjusted T-scores differed between the two groups at the lumbar spine (mean adjusted ± SEM, -0.5 ± 0.2 (PFF group) vs. -1.2 ± 0.2 (control group), p = 0.008) but not at the femoral neck or at the total hip.

**Conclusion:** Low-energy PFF should be considered as an osteoporotic fracture and treated accordingly.

**P196**

**The first comparison of hip fracture incidence across 4 countries in Africa**

*Celia Gregson[1,2], Hannah Wilson[1], Anya Burton[1], Momodou Jallow[3], Bintou Trawelly[4], Lucy Gates[5], Tadios Manyanga[2], James Masters[6], Rashida Ferrand[2,7], Matt Costa[6], Kebba Marenah[4], Kate Ward[3,5]*
*[1]University of Bristol, Musculoskeletal Research Unit, Bristol, United Kingdom*
*[2]Biomedical Research and Training Institute, The Health Research Unit Zimbabwe, Harare, Zimbabwe*
*[3]MRC Unit The Gambia @ London School of Hygiene and Tropical Medicine, Nutrition, Banjul, Gambia*
*[4]Edward Francis Small Teaching Hospital, Department of Orthopaedics & Trauma, Banjul, Gambia*
*[5]University of Southampton, MRC Lifecourse Epidemiology Centre- Human Development and Health, Southampton, United Kingdom*
*[6]University of Oxford,*
*Oxford Trauma and Emergency Care- Nuffield Department of Orthopaedics- Rheumatology and Musculoskeletal Science, Oxford, United Kingdom*
*[7]London School of Hygiene and Tropical Medicine, Clinical Research Department, London, United Kingdom*

**Abstract Text**

**Introduction:** Longevity in African populations is increasing which will inevitably increase fragility fracture risk; deprivation and malnutrition are common. There are few prospective hip-fracture incidence data in the continent, none in West Africa.

**Purpose:** To determine age and sex specific hip fracture incidence rates in adults age ≥40 years in The Gambia, West Africa and compare with those from other African countries.

**Methods:** All hip fracture cases in adults aged ≥40 years, presenting to a hospital or traditional bone setter (TBS) in the study area over 2-years, were identified. Age- and sex- specific hip fracture incidence per 100,000 person-years were estimated using the 2024 Gambian Population census. Incidence estimates were compared between The Gambia, Zimbabwe[1], South Africa[2] and Botswana[3]. Future hip fracture numbers were estimated to 2054 using UN population projections.

**Results:** Over 2-years, 238 (67% female) patients, mean(SD) age 71.3(12.5) years, presented to hospital (197[82.8%]) or TBS (37[15.5%]). Most reported fragility fractures (217[91.2%]). Delayed presentation was common (70[29.4%]). Incidence rates in The Gambia were 28.0 and 56.2/100,000 person-years for men and women respectively. Hip fractures numbers are predicted to nearly quadruple, from 175 in 2024, to 656 in 2054.

Age-standardised hip fracture incidence rates were broadly comparable between The Gambia, Zimbabwe, Botswana, and the Black South African population (Figure-1). All countries see higher incidence in men than women until age >50 years. The highest incidence rates in women age 60-74 years were in The Gambia, and in women age >75 years in Zimbabwe.

**Conclusions:** These are the first hip fracture incidence data from West Africa. Fragility fractures in Gambian adults were common, indicative of age-associated osteoporosis. Across the 4 countries, hip fracture cases are predicted to increase two-four-fold over coming decades. Healthcare systems now need to pivot to provide fracture services for ageing populations.

[1]Wilson 2025; [2]Dela 2020; [3]Kebaetse

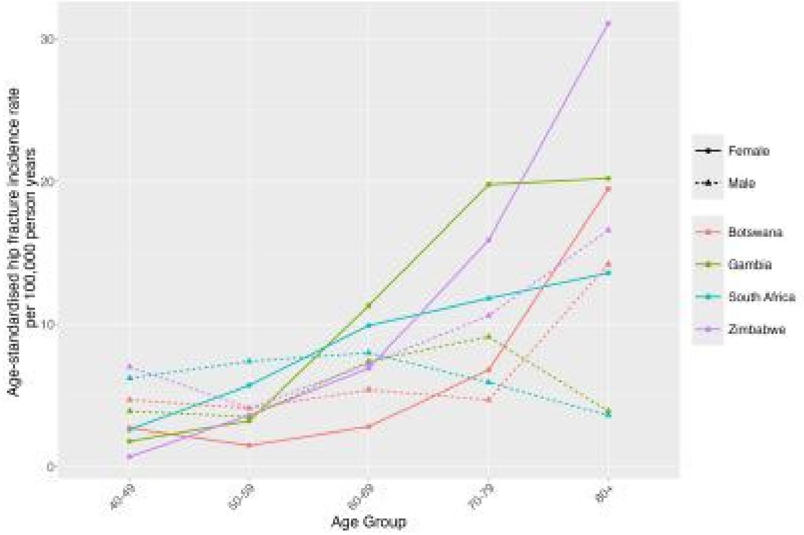

2021

**P197**

**Lipid Profile Alterations in the Mandible of a Rat Model with Ovariectomy-induced Osteoporosis**

*Maxime Bedez*[1], *Cécile Olejnik*[1], *Nicolas Bertheaume*[1], *Alexandrine During*[1]
[1]*University of Lille, MABLab ULR4490, Lille, France*

**Abstract Text**

**Background:** Postmenopausal osteoporosis correlates with increased marrow adiposity and altered lipid profiles, suggesting lipotoxic effects on bone. The mandible, an unpaired bone with mixed embryological origin, shows natural resistance to osteoporosis. We hypothesize that osteoporosis affects the bone lipid profile differently depending on its anatomical location.

**Purpose:** With the support of MSDAVenir, this project, which is part of the LIPIDOS initiative, specifically focuses on analyzing the lipid profile of the mandible in an ovariectomy-induced rat model.

**Methods:** Sprague-Dawley rats (6 months) underwent ovariectomy (n=10). At 9 months post-surgery, mandibles and tibiae were harvested and frozen. Lipids were extracted from mineralized tissue and fatty acids analyzed via HPLC.

**Results:** As expected, fat content was lower is mandibles compared to tibiae. At the individual level, no significant difference was observed between mandibles and tibiae for 16:0 (24.3 % vs 25.3 %, p = 0.10). However, 18:0 and 20:4 n-6 levels were higher in mandibles (9.6 % vs 5.2 %, p < 0.001 and 7.8% vs 2.0%, p < 0.001, respectively), while 16:1 n-7 and 18:1 n-7 were lower (5.4 % vs 6.4 %, p = 0.001 and 24.2 % vs 30.2 %, p < 0.001, respectively). Additionally, the ratios 16:1/16:0 and 18:1/18:0, markers of delta-9 desaturase activity, were notably lower in mandibles (0.2 vs 0.3, p = 0.014 and 2.6 vs 6.8, p < 0.001, respectively).

**Conclusions:** Delta-9 desaturase higher ratios was previously associated with osteoporotic status in femur of rats (During, 2020, *Calcif Tissue Int*). Mandibles exhibit lower delta-9 desaturase ratios, and lower inflammatory 20:4 n-6 levels, indicating reduced inflammatory response, while stable 16:0 underscores its structural role. These results suggest that the mandible's resistance to osteoporosis may be partially explained by adaptations in its lipid profile.

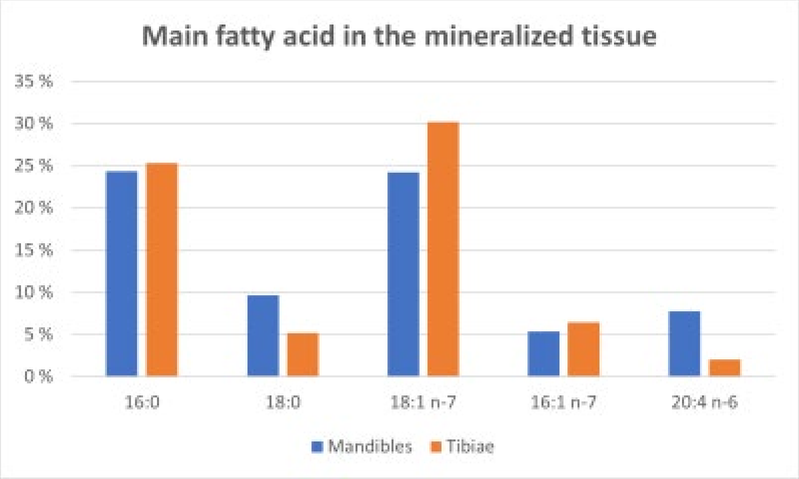

Main fatty acids of the ovariectomized rats in mandibles (n=10) and tibiae (n=10).

**ND-P017**

**Fall incidence and risk factors in a hospital: a descriptive analysis from 2020-2024**

*Martina Behanova*[1,2], *Gabriele Rametsteiner-Ritzinger*[3], *Michaela Fischer*[3], *Brunhilde Flicker*[3], *Anton Sokhan*[1,2], *Jochen Zwerina*[1], *Roland Kocijan*[1,2]
[1]*Ludwig Boltzmann Institute of Osteology at Hanusch Hospital of OEGK and AUVA Trauma Centre Mei dling, 1st Med. Dept. Hanusch Hospital, Vienna, Austria*
[2]*Sigmund Freud University Vienna- School of Medicine, Metabolic Bone Diseases Unit, Vienna, Austria*

[3]Hanusch Hospital of OEGK, Hanusch Hospital of OEGK, Vienna, Austria

**Abstract Text**

**Introduction:** A high risk of falling is considered an imminent risk factor for osteoporotic fractures. While fractures in the elderly are commonly fall-related, this connection is often missing in statistical data. In hospitals, falls are thoroughly documented, offering insights into fall patterns and risk factors for fractures.

**Purpose:** To analyze the incidence, patterns, and factors associated with falls in a university teaching hospital in Vienna over a five-year period.

**Methods:** We conducted a retrospective analysis of falls documented from 2020 to 2024. Data collected included patient demographics (age group, sex) and fall-related information (cause, circumstances, timing, location, and resulting injuries). Cumulative incidence was calculated as the number of falls divided by the number of inpatients.

**Results:** The average cumulative incidence of falls was 1.65% (range: 1.24-2.03%). Males experienced falls more frequently than females, with male representation ranging from 50.6% in 2024 to 60.3% in 2022. The 80-89 age group was most affected, accounting for 37.2% of all falls on average. Patient rooms were the predominant location (72.2%), and every fourth fall (25.2%) resulted in injury. Falls occurred during night shifts in 33% of cases and most commonly during position changes, particularly when standing up or sitting down (31.9%). The factors most frequently associated with falls were physical weakness (20.9%), impaired mobility (18.2%), and a history of falling within the previous six months (17.9%).

**Conclusion:** Our findings identified specific high-risk circumstances and patient characteristics associated with falls in the hospital setting. Attention should be directed toward older patients and prevention strategies should focus on nighttime supervision, assistance with position changes, and interventions for patients with mobility limitations and fall history.

**Poster Sessions: Osteoporosis: treatment**

**P198**

**Long term treatment with denosumab in patients with celiac disease and osteoporosis: a pilot study**

*Jessica Pepe[1], Luciano Colangelo[1], Rachele Santori[1], Marco Occhiuto[1], Daniele Diacinti[1], Evaristo Ettore[1], Giovambattista Desideri[1], Salvatore Minisola[1], Cristiana Cipriani[1]*
*[1]Sapienza University, Department of clinical- internal- anesthesiology and cardiovascular sciences, Rome, Italy*

**Abstract Text**

**Background:** Patients with celiac disease have lower bone mineral density (BMD) and higher incidence of fractures compared to age- and sex-matched controls. BMD improvement with gluten-free diet (GFD), which is the mainstream therapy for celiac disease, has been shown by several studies, but data on the efficacy of pharmacological treatment of osteoporosis are scant in this population.

**Purpose:** To study the long-term effect of denosumab on BMD in celiac patients with osteoporosis on GFD, compared to non-celiac osteoporotic patients.

**Methods:** Fifteen celiac patients with osteoporosis and control subjects of the same age, sex, BMI, and fragility fractures were enrolled. At baseline, each patient underwent biochemical tests, spine X-ray, DXA measurements and the Charlson Comorbidity Index (CCI) was computed. At each visit (every 2 years ±6 months), with a follow-up of four years, any adverse events or new clinical fractures, DXA measurements and CCI were recorded.

**Results:** The mean age of the patients was 73.66±11.67 years and the mean BMI was 23.78±3.77 $kg/m^2$ . All patients followed a GFD and had no symptoms or biochemical findings of malabsorption. In celiac patients a statistically significant delta increase in total hip T-score of 0. 15±0.18 compared to baseline was observed (ANOVA $p < 0.05$), while in the control group of 0.09±0.10 (ANOVA $p<0.05$), with no difference between groups. New fractures occurred in the celiac group in five patients during the follow-up, and in two patients in the control group ($p=0.38$). No adverse events occurred during follow-up. Baseline hemoglobin value in the celiac patients was positively associated with the delta increase of T-score total hip after 2 years of denosumab treatment but not in the control group ($r= 0.64$, $p<0.05$).

**Conclusion:** In celiac patients with osteoporosis on GFD, denosumab, up to four years of follow-up, increased hip BMD without adverse events.

**P199**

**Combined treatment of romosozumab and whole body vibration in an animal model of postmenopausal osteoporosis**

*<u>Marina Komrakova</u>[1], Lennart Jansen[1], Wolfgang Lehmann[1], Kai Oliver Böker[1], Arndt Friedrich Schilling[1], Daniel Bernd Hoffmann[1]*
*[1]University Medical Center Goettingen, Trauma Surgery- Orthopaedics and Plastic Surgery, Goettingen, Germany*

**Abstract Text**

**Introduction:** Sclerostin is produced by bone cells and acts as a negative regulator of bone formation through the Wnt/β-catenin signaling pathway. Romosozumab (Evenity®), an anti-sclerostin monoclonal antibody (ROMO), is approved for the treatment of severe osteoporosis in postmenopausal women. Whole body vibration (WBV) is known to benefit musculoskeletal tissue and reduce plasma sclerostin levels.

**Purpose:** The effect of sclerostin inhibition by combined ROMO and WBV treatments on bone was investigated.

**Methods:** The animal study was approved prior to the study. Six-month old female rats were ovariectomized (OVX) or sham operated (SHAM). After 8 weeks, rats were divided into groups (n=15/group): 1) SHAM, 2) OVX, 3) OVX+ROMO treated s.c. with ROMO (25 mg/kg body weight, 1x/week), 4) OVX+WBV vibrated 2x/day, for 15 min, 7x/week, 5) OVX+ROMO+WBV underwent combination therapy. Groups 1, 2 and 4 received vehicle injections. After 8 weeks, bone samples were analyzed by microcomputed tomography, biomechanical and ashing analyses. Sclerostin protein was measured using ELISA.

**Results:** ROMO alone improved all biomechanical and structural bone parameters in OVX rats. WBV alone did not change bone quantity and quality. When WBV was combined with ROMO, the effect was stronger than single treatments, further improving biomechanical properties, increasing bone density and serum osteocalcin. In ROMO treated rats, the amount of sclerostin in serum, muscle, brain, and bone was 200, 160, 16, and 4 times higher, respectively, than in the other groups. There were no cross-reactions of ROMO drug itself in ELISA.

**Conclusions:** ROMO+WBV had a favorable synergistic effect on bone parameters and can be recommended for osteoporosis treatment. ROMO effect on bone was confirmed, whereas WBV alone was less effective than expected. The high endogenous levels of sclerostin upon ROMO treatment should be further investigated to elucidate possible side effects of ROMO. This could be a reason for the known rebound effect in patients after discontinuation of ROMO therapy.

**P200**

**Impact of orthogeriatric care models in Belgium: A population-based data registry study**

*Sigrid Janssens[1], Mieke Deschodt[1,2], An Sermon[3,4], Johan Flamaing[1,5], Marian Dejaeger[1,5]*
*[1]KU Leuven, Public Health and Primary Care, Leuven, Belgium*
*[2]University Hospital Leuven, Centre of Nursing, Leuven, Belgium*
*[3]KU Leuven, Development and Regeneration, Leuven, Belgium*
*[4]University Hospital Leuven, Trauma, Leuven, Belgium*
*[5]University Hospital Leuven, Geriatric Medicine, Leuven, Belgium*

**Abstract Text**

**Background/introduction:** Orthogeriatric co-management has demonstrated improved patient outcomes. However, there is no conclusive evidence on the optimal organization of orthogeriatric care.

**Purpose:** The study aimed to compare he effectiveness of different types of orthogeriatric care in Belgian hospitals.

**Methods:** This data registry study included patients aged 75 years and over, admitted with a hip, femur, pelvic, forearm, humerus, or vertebral fracture between Jan 1-Dec 31, 2019. A survey was sent to all 95 Belgian hospitals to map the care models: (1) admission on surgical ward or (2) geriatric with consultation on request, (3) admission on surgical or (4) geriatric ward with systematic consultation, or admission on an integrated (5) surgical or (6) geriatric ward. The primary outcome was 1-year mortality; secondary outcomes included 30-day and 90-day mortality, length of stay (LOS), emergency department (ED) visits and unplanned readmissions within 30 and 90 days, and 1-year institutionalization.

**Results:** A total of 8302 patients of the 84 participating hospitals were included. The overall one-year mortality was 14.5% and did not differ between the six care models (p=0.524). In-hospital mortality, 30-day mortality, 90-day mortality, one-year institutionalization, and 90-day unplanned readmissions did not differ. Patients in model 5 had a lower rate of ED visits within 30 days than those in model 3 (5.9% vs. 11.5%; p=0.025) and also had a lower rate of ED visits within 90 days compared to model 1 (3.8% vs. 5.2%; p=0.008) and model 3 (3.8% vs. 7.6%; p=0.008). Patients in model 6 had the highest median LOS (17 (IQR=26) days), while patients in model 5 had the lowest median LOS (11 (IQR=11) days) (p<0.001).

**Conclusion:** The study found no significant differences in mortality and institutionalization between the orthogeriatric care models. However, an integrated surgical ward showed a lower rate of ED visits and shorter LOS, suggesting potential benefits.

**P201**

**Baricitinib for the treatment of age-related osteoporosis**

*<u>Katharina Gelles</u>[1], Vincent Kurz[1], Anastasia Meshcheryakova[1], Maria Butylina[1], Katharina Wahl-Figlash[1], Peter Pietschmann[1]*
*[1]Medical University of Vienna MUW, Center for Pathophysiology- Infectiology and Immunology, Vienna, Austria*

**Abstract Text**

**Background:** Proinflammatory cytokines are involved in the pathogenesis of age-related osteoporosis. Baricitinib, a Janus Kinase (JAK) inhibitor, mediates cytokine signaling and is used for the treatment of inflammatory diseases such as rheumatoid arthritis.

**Purpose:** We hypothesized that baricitinib will improve bone properties in the senescence accelerated mouse 8 (SAMP8), an established model of age-related osteoporosis.

**Methods:** At the age of eight months 60 female SAMP8 mice were randomized to baricitinib or placebo group. After a treatment period of six weeks twice daily by gavage, bones were assessed by µCT and histomorphometry. Additionally, flow cytometry (FACS) was performed for the assessment of intracellular cytokine production of spleen cells.

**Results:** Comparing the intracellular production of $TNFa^+$ and $IL21^+$ in T cells of both groups, the baricitinib group showed significantly lower cytokine levels ($CD4^+$: p=0.089; $CD8^+$: p=0.0043). Additionally, levels of $IL6^+$and $IL21^+$ were significantly higher in the placebo group ($CD4^+$: p=0.0061;

CD8$^+$: p=0.0134). µCT evaluation of the BV/TV in the vertebral body and the femur showed no significant difference between group. In contrast, results of bone histomorphometry exhibited a significantly higher BV/TV (+21.8%; p=0.0221) compared to the placebo group.

**Conclusion:** Our current results demonstrate, an anti-inflammatory impact of baricitinib in immune cells and a favorable effect of the compound on bone properties assessed by bone histomorphometry. In contrast, parameters determined by µCT did not differ in the baricitinib and placebo group.

**Keywords** osteoporosis, baricitinib, SAMP8, histomorphometry, micro-computed tomography

This project was supported by the Austrian Science Fund (P35262).

This project was approved by the intramural Committee for Animal Experimentation and Austrian Federal Ministry of Education, Science and Research (BMBWF) (2022 - 0.238.324).

**P202**

**Romosozumab and denosumab combination therapy in postmenopausal osteoporosis**

*Giovanni Adami*[1], *Francesco Pollastri*[1], *Angelo Fassio*[1], *Ombretta Viapiana*[1], *Davide Gatti*[1], *Maurizio Rossini*[1]

[1]*University of Verona, Rheumatology Unit, Verona, Italy*

**Abstract Text**

**Background:** Transitioning from long-term denosumab to PTH-analogs or romosozumab may expose patients to a rebound phenomenon. Adding romosozumab to denosumab may offer a therapeutic alternative for postmenopausal women who sustain fractures while on denosumab.

**Objectives:** This study aimed to evaluate the effects of combining romosozumab with ongoing denosumab therapy in postmenopausal osteoporosis.

**Methods:** We conducted a combined 12-month prospective and 24-month retrospective observational study. Postmenopausal women were divided into three cohorts:

1. Treatment-naive patients initiating romosozumab (Naïve→Romo; M-24 to M+12),
2. Patients on denosumab who added romosozumab (Dmab→Dmab+Romo; M-24 to M+12),
3. Patients continuing denosumab (Dmab→Dmab; M-24 to M+12).

Bone mineral density (BMD) and bone turnover markers (BTMs; CTX and P1NP) were measured at key time points. A mixed model for repeated measures (MMRM) was used to assess percentage and absolute changes in BMD and BTMs, with treatment sequence, time, treatment-by-time interaction, and baseline BMD as fixed effects and patients as random effects.

**Results:** A total of 139 patients participated: 90 were Naïve→Romo, 25 were Dmab→Dmab+Romo, and 24 were Dmab→Dmab. At lumbar spine M+12, the Dmab→Dmab+Romo group showed a trend toward greater BMD increases compared to the Dmab→Dmab group (+3.2%, SE 4.4; p = 0.074), as did the Naïve→Romo group compared to Dmab→Dmab (+4.4%, SE 3.7; p = 0.064). No significant differences were observed at the femoral neck or total hip. P1NP levels significantly differed at M+3 between the Dmab→Dmab and Dmab→Dmab+Romo groups (+24.4 ng/mL, SE 12.6; p = 0.033), with a trend toward significance at M+6 (+13.4 ng/mL, SE 7.7; p = 0.057).

**Conclusion:** Romosozumab retains anabolic potential when combined with denosumab, supporting its use in patients with severe osteoporosis inadequately responding to long-term denosumab.

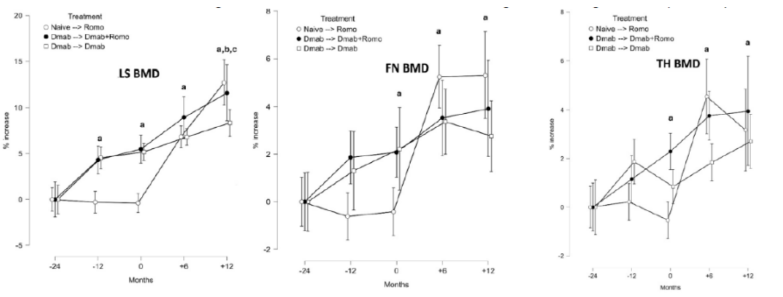

*a, p value <0.05 vs baseline; b, p value 0.074 for Dmab→Dmab vs Dmab→Dmab+Romo; c, p value 0.064 for Dmab→Dmab vs Naive→Romo. Between-groups differences were tested with a mixed model for repeated measures (MMRM) using Satterthwaite and restricted maximum likelihood method with treatment sequence, time, treatment-by-time interaction, and baseline bone mineral density (BMD) as fixed effects and with patients as random effect. LS, lumbar spine; FN, femoral neck; TH, total hip*

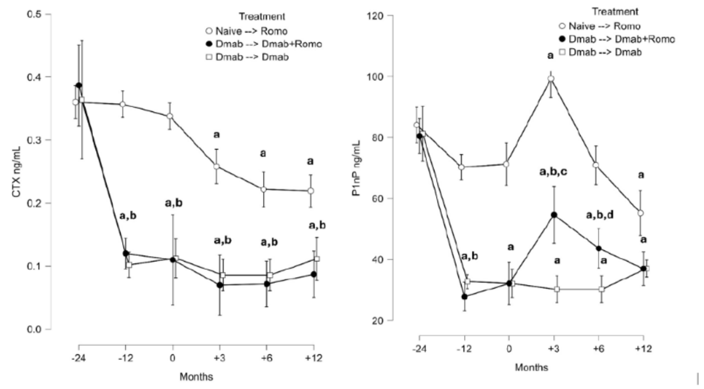

*a, p value <0.05 vs baseline; b, p value <0.05 for Dmab→Dmab and Dmab→Dmab+Romo vs Naïve→Romo; c, p value 0.033 for Dmab→Dmab vs Dmab→Dmab+Romo; d, p 0.057 for Dmab→Dmab vs Dmab→Dmab+Romo. Between-groups differences were tested with a mixed model for repeated measures (MMRM) using Satterthwaite and restricted maximum likelihood method with treatment sequence, time, treatment-by-time interaction, and baseline bone mineral density (BMD) as fixed effects and with patients as random effect. CTX, C-terminal telopeptide of type I collagen; P1NP, Procollagen I Intact N-Terminal Peptide*

**P203**

**Effectiveness of romosozumab on bone mineral density surrogate threshold effect: results from the ROMosozumab Early experience in female patients with severe Osteoporosis (ROMEO study)**

*Giovanni Adami[1], Bartezaghi Marta[2], Davide Gatti[1], Ombretta Viapiana[1], Rachele Fornari[2], Maurizio Rossini[1]*
*[1]University of Verona, Rheumatology Unit, Verona, Italy*
*[2]UCB Pharma, Ucb Italy, Milan, Italy*

**Abstract Text**

**Background:** Romosozumab (ROMO), a sclerostin inhibitor, has been approved in Italy for treating postmenopausal osteoporosis (OP) in women at high fracture risk.

**Objectives:** To evaluate the effectiveness of ROMO in a cohort of Italian postmenopausal women at high fracture risk.

**Methods:** We retrospectively analyzed prospectively collected data from postmenopausal women with severe OP treated with ROMO at the Verona center. Bone mineral density (BMD) was assessed at baseline, month 6, and month 12. Bone turnover markers (BTMs) and calcium-phosphate metabolism were measured at baseline and months 3, 6, and 12. The surrogate threshold effect (STE) for BMD, defined as the change needed to predict fracture risk reduction with 95% confidence, was evaluated based on the FNIH-ASBMR SABRE Project criteria.

**Results:** The study included 133 postmenopausal women (mean age 72.5 ± 9.5 years). Seventy-nine patients completed 6 months of follow-up, and 39 completed 12 months. A subgroup of 91 patients had minimal prior bisphosphonate exposure (MEB). Significant BMD increases were observed at all sites at 6 and 12 months, particularly in the MEB group: femoral neck (+3.9% and +6.5%), total hip (+3.4% and +3.7%), and lumbar spine (+7.5% and +10.9%). Over 50% of patients reached the STE for fracture risk reduction by 6 months, with higher proportions in the MEB group. Among patients with baseline lumbar spine T-scores ≤-2.5, 18.2% and 32.0% reached a T-score >-2.5 at months 6 and 12, respectively. P1NP levels increased significantly at months 3 (+76.2%) and 6 (+34.6%), while CTX levels decreased (-2.6% and -26.6%). BTMs returned to normal ranges by month 12.

**Conclusion:** In this real-world cohort of Italian postmenopausal women at high fracture risk, over half achieved the STE for minimal fracture risk reduction, particularly those with minimal bisphosphonate exposure. Additionally, one-third reached osteopenic BMD levels after ROMO treatment.

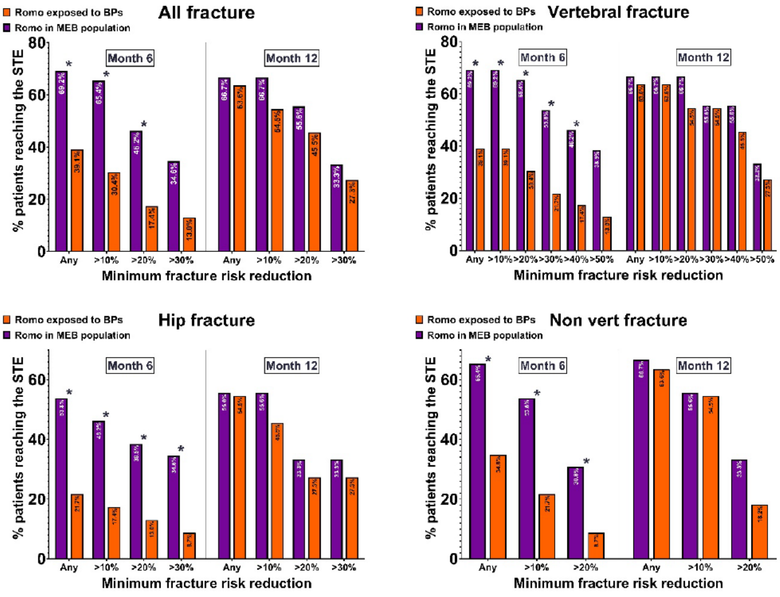

**P204**

**A Comparison of Osteoporosis Intervention Thresholds: A Data-Driven Analysis**

*Lan Yang[1], Attracta Brennan[2], Bryan Whelan[1], Damian Gonzalez Garza[1], Erjiang Erjiang E[3], Tingyan Wang[4], Rebecca Egan[5], Kelly Gorham[5], Fiona Heaney[5], Catherine Armstrong[5], Guadalupe Morote Ibarrola[5], Amina Gsel[5], Ming Yu[6], Mary Dempsey[7], John Carey[1]*

[1]*University of Galway, School of Medicine, Galway, Ireland*
[2]*University of Galway, School of Computer Science, Galway, Ireland*
[3]*Guangxi Minzu University, School of Management, Nanning, China*
[4]*University of Oxford, Nuffield Department of Medicine, Oxford, United Kingdom*
[5]*University Hospital Galway, Department of Rheumatology, Galway, Ireland*
[6]*Tsinghua University, Department of Industrial Engineering, Beijing, China*
[7]*University of Galway, School of Engineering, Galway, Ireland*

**Abstract Text**

**Background**: Clinical guidelines recommend considering osteoporosis medication for people with a diagnosis of osteoporosis, a major osteoporotic fracture, taking chronic glucocorticoids and those at high risk of fracture. Many eligible patients are not treated so identifying the most appropriate intervention criteria for osteoporosis treatment is critical to optimise care and close the osteoporosis treatment gap.

**Purpose**: To compare agreement between different osteoporosis treatment intervention thresholds.

**Methods**: A previously published, validated subset of The DXA-HIP cohort was used to compare five intervention thresholds: 1) a Major Osteoporotic Fracture (MOF); 2) central DXA T-score ≤ -2.5; 3) chronic glucocorticoid therapy; 4) Irish FRAX® with BMD MOF ≥ 20% or Hip fracture (HF) ≥ 3%; and 5) Irish FRAX® without BMD MOF ≥ 20% or HF ≥ 3%. Agreement between thresholds was compared using Cohen's kappa coefficient, and a Venn diagram to demonstrate overlap between thresholds.

**Results**: 1,254 adults aged 40–90 years were included: 290 men and 964 women, mean age 69 years, mean lowest T-score: -1.4, FRAX® with BMD MOF: 13%, HF: 4%.

220 (18%) did not meet any treatment criteria, 1025 (82%) met 1 or more, and 9 (<1%) met all. There was considerable variation in the proportion deemed eligible for treatment across the different criteria: 48% prior MOF, 13% T-score ≤ -2.5, 20% glucocorticoid therapy, 46% for FRAX® with BMD, and 66% for FRAX® without BMD.

A Venn diagram (shown below) reveals considerable variability and overlap across different thresholds. with notable disagreements, particularly involving those on glucocorticoid therapy.

Agreement ranged from very poor (kappa: - 0.15) to moderate (kappa: 0.48).

**Conclusions**: There is considerable heterogeneity and inconsistency between various intervention thresholds for osteoporosis treatment, which could explain differences in treatment rates. This underscores the need for harmonisation of intervention thresholds to close the osteoporosis treatment gap.

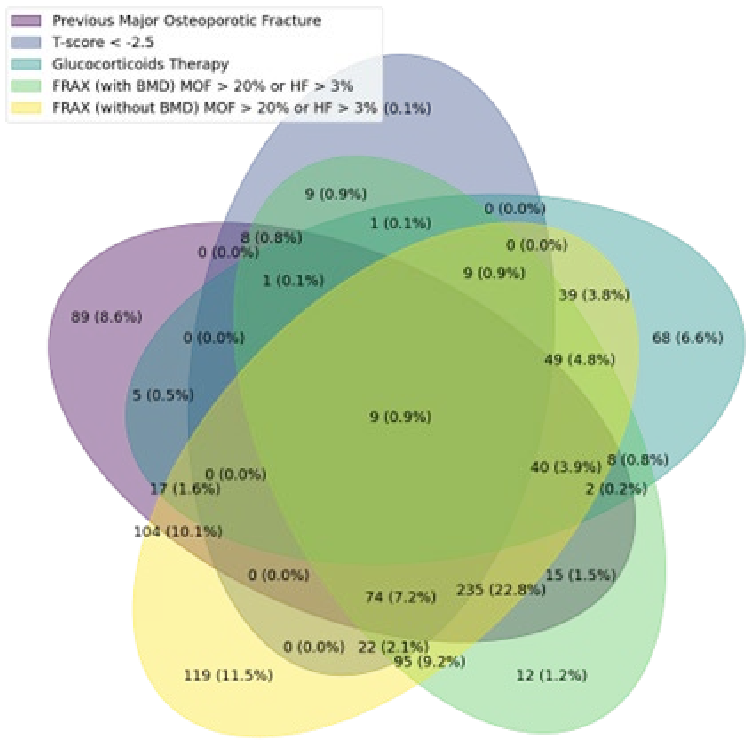

**P205**

**One Year of Treatment with Denosumab or Zoledronate in Postmenopausal Women with Osteoporosis and Primary Hyperparathyroidism**

*Katarina Mlekus Kozamernik*[1], *Luka Lezaic*[2], *Marko Hocevar*[3], *Tomaz Kocjan*[1]
[1]*UMC Ljubljana, Department of Endocrinology- Diabetes and Metabolic Diseases, Ljubljana, Slovenia*
[2]*UMC Ljubljana, Department of Nuclear Medicine, Ljubljana, Slovenia*
[3]*Institute of Oncology, Surgery, Ljubljana, Slovenia*

**Abstract Text**

**Background:** Optimal medical treatment of postmenopausal women with osteoporosis and primary hyperparathyroidism (PHPT) who refuse surgery has not been determined.

**Purpose:** We aimed to compare the effects of one year of treatment with denosumab or zoledronate in this population.

**Methods:** Forty postmenopausal women with osteoporosis and PHPT (aged 73.0 (7.8 SD) years, 22.6 (10.0) years post-menopause, BMI 27.77 (5.1) kg/m$^2$) were randomized to either zoledronate 5 mg iv annually (ZOL group) or denosumab 60 mg sc every six months (DMAB group) (ClinicalTrials.gov NCT04085419; study has ethical approval). Serum calcium levels after 3 (3M), 6 (6M), and 12 months

(12M), as well as BMD at four standard sites and trabecular bone score (TBS) 12 months post-therapy initiation, were measured and compared between the two groups.

**Results:** There was a statistically significant decrease of 3M serum calcium in both groups (ΔDMAB -0.07 mmol/L; p=0.045, ΔZOL -0.08 mmol/L; p<0.01). In the ZOL group, serum calcium remained low (median 6M 2.66 (0.51) and 12M 2.67 (0.44) mmol/L), while it slowly increased in the DMAB group (median 6M 2.69 (0.73) and 12M 2.75 (1.26) mmol/L). After one year of treatment, LS BMD was significantly higher in both groups (ΔDMAB +0.038 g/cm$^2$; p<0.001 and ΔZOL +0.040 g/cm$^2$; p=0.003). TH BMD was significantly higher only in the DMAB group (ΔDMAB TH +0.036; p=0.006), while FN and 1/3R BMD did not change in any group. Altogether, patients had significantly higher TBS (ΔTBS +0.066; p=0.016). The two groups had no statistically significant differences in any of the observed parameters.

**Conclusion:** One year of ZOL or DMAB in postmenopausal women with osteoporosis and PHPT yielded similar beneficial effects on serum calcium, BMD, and TBS.

**P206**

**Bone mineral density response to romosozumab in older postmenopausal women: a prospective observational study**

*<u>Michaël Laurent</u>[1], Désirée Coppens[2], Jolan Dupont[2], Kurt De Vlam[3], Marian Dejaeger[2], Evelien Gielen[2]*
*[1]Imelda Hospital, Geriatrics, Bonheiden, Belgium*
*[2]University Hospitals Leuven, Geriatrics, Leuven, Belgium*
*[3]University Hospitals Leuven, Rheumatology, Leuven, Belgium*

**Abstract Text**

**Background:** Romosozumab is approved in the European Union to treat severe osteoporosis in postmenopausal women at high risk of fracture. Randomized trials with this medication have included older adults, but specific data on response to therapy in geriatric patients are lacking. We have previously shown that bone mineral density (BMD) changes associated with teriparatide use were greater in younger patients with lower baseline BMD.

**Purpose:** To examine bone mineral density (BMD) changes and adverse events in a real-world cohort of older postmenopausal women treated with romosozumab. We hypothesized that younger, treatment-naive patients or those with lower baseline BMD might experience greater BMD improvements.

**Methods:** Retrospective and prospective observational study in routine clinical practice at two Belgian centres. The study was approved by the Ethics Committee in both hospitals, and participants provided written informed consent.

**Results:** We included 104 postmenopausal women (mean [standard deviation] age 76 [10] years) with a recent fracture (29 hip, 51 vertebra, 38 other), a mean T-score of -2.1 at the lumbar spine and total hip, and a mean T-score of -2.7 at the femoral neck. BMD increased +10.6% at the total spine,

+2.0% at the hip, and +1.7% at the femoral neck. Pearson correlation showed that BMD changes were associated with baseline BMD or T-score at the spine (-0.310, p=0.008) or total hip (-0.340, p=0.039). Prior treatment with other osteoporosis drugs was associated with lower BMD gains at the lumbar spine (-0.252, p=0.030). There was no significant association of BMD response with age. Twelve patients reported side effects (mainly injection-site related). There were six fractures, one myocardial infarction, one stroke and four deaths, mainly in frail older patients.

**Conclusions:** Our data support the use of romosozumab in older postmenopausal women in routine geriatric practice. Further research is required to optimize the use of anabolic agents in the oldest old.

**P207**

**Post-discharge impact of nurse-led orthogeriatric co-management on patient outcomes and secondary fracture prevention: An observational pre-post study**

*Sigrid Janssens[1], Mieke Deschodt[1,2], An Sermon[3,4], Katleen Fagard[1,5], Marie Cerulus[1,2], Heidi Cosyns[5], Johan Flamaing[1,5], Marian Dejaeger[1,5]*
*[1]KU Leuven, Public Health and primary care, Leuven, Belgium*
*[2]University Hospital Leuven, Centre of Nursing, Leuven, Belgium*
*[3]KU Leuven, Development and Regeneration, Leuven, Belgium*
*[4]University Hospital Leuven, Traumatology, Leuven, Belgium*
*[5]University Hospital Leuven, Geriatric Medicine, Leuven, Belgium*

**Abstract Text**

**Background:** Fracture patients are at imminent risk of additional fractures. Orthogeriatric care might reduce the osteoporosis treatment gap and improve patient outcomes in hip fracture patients.

**Purpose:** This study assessed whether nurse-led orthogeriatric co-management on a surgical ward improves post-discharge outcomes and secondary fracture prevention in patients ≥75 years hospitalized with a major osteoporotic fracture (hip, pelvis, shoulder, wrist, spine).

**Methods:** This observational study took place at the trauma department of the University Hospital Leuven. We evaluated 108 patients before and after the implementation of a nurse-led orthogeriatric co-management program, G-COMAN. G-COMAN included proactive geriatric care, comprehensive geriatric evaluation, multidisciplinary interventions, and systematic follow-up. Outcomes at 1, 3, 6, and 12 months post-surgery included mobility status, functional status, instrumental activities of daily living status, nutritional status, quality of life, return to pre-fracture living situation, unplanned readmissions, mortality, and secondary fracture prevention measures.

**Results:** The intervention group showed an worser mean Parker mobility score at one month (3.8 vs. 3.1; p=0.036). There were no significant differences in other patient outcomes. Intervention patients received more calcium/vitamin D supplements at 1 month (70.1% vs. 82.4%; p=0.042) and had more lab investigations for secondary osteoporosis (24.1% vs. 50.0%; p<0.001). Referral to fracture liaison service (FLS) was similar for both groups (70.4% vs 77.8%; p=0.214), actual presence at FLS was 37%

in both groups due to cancellations. Non-hip fracture intervention patients had fewer 30-day unplanned readmissions (20.7% vs. 3.4%; p=0.044) and were more frequently referred to a fracture liaison service at 12 months (56.7% vs. 93.5%; p=0.001).

**Conclusion**: While the nurse-led orthogeriatric co-management intervention did not improve overall post-discharge outcomes, it strengthened secondary fracture prevention efforts across fracture types especially also in non-hip fracture patients. The findings also highlight the importance for patient education on osteoporosis.

**P208**

**The Effect of Acetylcholinesterase Inhibitors on Dementia, Falls, Fractures and Mortality in a Geriatric Cohort: A 4-Year Follow-Up Study**

*Charles Inderjeeth*[1], *Diren Che Inderjeeth*[2], *Maxine Isbel*[3], *Sneha Bharadwaj*[4], *Dani Kostova*[3]
*[1]Sir Charles Gardner Hospital, Geriatric- Acute and Rehabilitation Medicine, Nedlands, Australia*
*[2]Sir Charles Gairdner Osborne Park Health Care Group, Geriatric- Acute and Rehabilitation Medicine, Perth, Australia*
*[3]Sir Charles Gairdner Osborne Park Health Care Group, Geriatric- Acute and Rehabilitation Medicine, Nedlands, Australia*
*[4]Fremantle Hospital, Geriatric Medicine, Fremantle, Australia*

**Abstract Text**

**Background:** We reported that in vitro and in vivo mouse models suggest that antidementia medications acetylcholinesterase inhibitors (AChEI) improve bone health through its impact on inhibiting osteoclasts and a potential anabolic effect on osteoblasts (Li, Xu, Inderjeeth et al, Journal Cellular Physiology 2023). In this study we a looked at the impact of AChEI in fracture risk in patients with dementia.

**Methods:** Memory Clinic prospective cohort followed up annually for 4 years. The main outcomes assessed were, incidence of new fracture and death. Data was entered into SPSS with analysis of frequency and comparison of groups using Chi-Square Test. Main analysis was demographics at baseline, diagnosis of dementia, prevalence of baseline osteoporosis and fracture, falls risk, dementia medications, osteoporosis medications and follow up incidence of fracture and mortality.

**Results:** 744 patients were followed up. At baseline fifty-nine percent were female; mean age 81 years (SD6.8).Twenty one percent had prior hip fracture Fifty six percent had dementia and 113 (15.2%) were on AChEI. During 4 years of annual follow up 137(18%) reported new fractures. Overall mortality rate was 267/744 (36%). Patients with dementia were more likely to die (180/415 vs 87/329; p <0.001) with a trend to more fractures (73/415 vs 64/329; p = 0.078). Patients on dementia medications were less likely to fracture 15/113 vs 122/631 (p = 0.029). Propensity matched AChEI cohort 4 year fracture risk OR was 0.7249 (95% CI: 0.4267 to 1.2314; p = 0.2340; z statistic = 1.190; Chi Square = 1.425).

**Conclusions:** Patients with dementia have a higher risk of recurrent fractures and mortality. Treating dementia with acetylcholinesterase inhibitors may reduce the risk of all fractures. This clinical finding supports our recently published in vitro and in vivo mouse model confirming the benefit of cholinesterase on bone histology, biology and biomarkers.

**Ethics:** SCGHOPH Ethics Committee

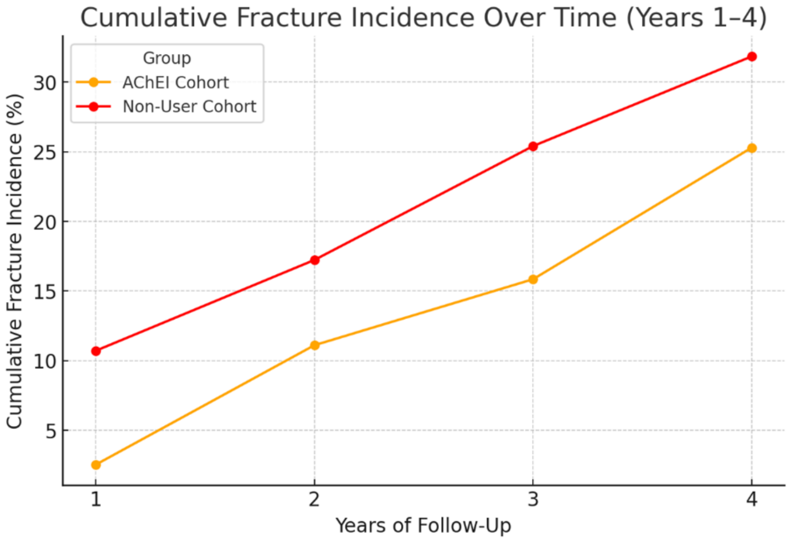

**P210**

**Phase 3 study comparing the efficacy and safety of proposed biosimilar RGB-14-P with denosumab in postmenopausal women with osteoporosis: results from the transition (switch) phase**

*Serge Ferrari[1], Lothar Seefried[2], Dénes Páll[3], Ombretta Viapiana[4], Jan Rosa[5], Jerzy Supronik[6], Rodina Nestorova Licheva[7], Joachim Kiefer[8], Norbert Jeszenői[8], Károly Horvát-Karajz[8], Enikő Jókai[8], István Takács[9]*

*[1]Geneva University Hospital, Division of Bone Diseases, Geneva, Switzerland*

*[2]University of Würzburg, Musculoskeletal Center Würzburg, Würzburg, Germany*

*[3]University of Debrecen, Department of Medical Clinical Pharmacology, Debrecen, Hungary*

*[4]University and Azienda Ospedaliera Universitaria Integrata of Verona, Rheumatology Unit, Department of Medicine, Verona, Italy*

*[5]Osteoporosis and Metabolic Bone Disease Center Affidea, Prague, Czech Republic*

*[6]NZOZ Centrum Medyczne Artur Racewicz, Bialystok, Poland*

*[7]Rheumatology Center St.Irina, Sofia, Bulgaria*

*[8]Gedeon Richter Plc, Budapest, Hungary*

*[9]Semmelweis University, Department of Internal Medicine and Oncology, Faculty of Medicine, Budapest, Hungary*

**Abstract Text**

**Background:** Denosumab is indicated for osteoporosis treatment, acting by blocking the nuclear factor kappa-B ligand (RANKL) receptor activator. RGB-14-P is a proposed denosumab biosimilar.

**Purpose:** To assess the clinical equivalence in efficacy and compare the pharmacodynamics (PD), safety and immunogenicity of RGB-14-P and reference denosumab (RD) when switching from RD to RGB-14-P in postmenopausal women with osteoporosis (EudraCT 2020-006017-38).

**Methods:** Participants were randomised 1:1 to receive subcutaneous RGB-14-P or RD 60 mg every 6 months, on Day 1 and at Week (W) 26. At W52, a subset of participants on RD were re-randomised 1:1 to receive RGB-14-P or RD 60mg and followed to W78. Here we report outcomes up to W78 (W52 results reported previously).

**Results:** Of the 473 participants randomised at W0, 188 participants were re-randomised at W52 (continued RGB-14-P, n=63; continued RD, n=63; switched RD-to-RBG-14-P, n=62). Following the bone mineral density (BMD) gains by W52, additional gains in lumbar spine (LS) (**Figure**), hip, and femoral neck (FN) BMD were observed with continuous RGB-14-P or RD to W78. Following switch from RD to RBG-14-P, there were no notable differences in LS, hip, or FN BMD compared with previous RD or continuing RGB-14-P. Incidences of new fragility fractures were comparable. Changes from baseline in serum PD markers (serum C-telopeptide of type I collagen, and procollagen type I N-terminal propeptide) were maintained. Switch did not induce a discernible immunological reaction, and there were no clinically meaningful differences between groups in safety (participants with ≥1 treatment-emergent adverse event: RGB-14-P: 47.6%; RD to RGB-14-P: 40.3%; RD 39.7%).

**Conclusion:** Treatment response to RGB-14-P or RD was maintained to W78; switch from RD to RBG-14-P had no discernible impact on efficacy, PD, immunogenicity, or safety.

Funding: Gedeon Richter; medical writing by Timothy Davies, PhD, of Avalere Health; funded by Gedeon Richter.

**Figure.** Mean %CfB (95% CI) in Lumbar Spine BMD in the FAS[a]

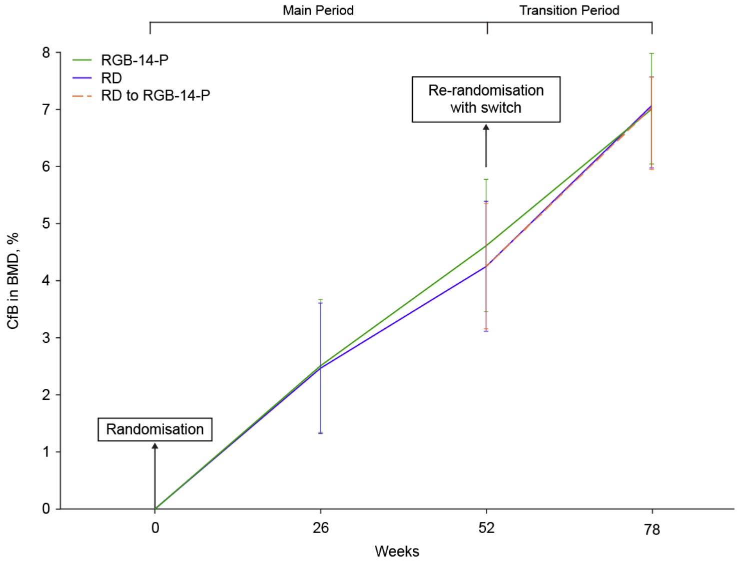

BMD, bone mineral density; CfB, change from baseline; CI, confidence interval; FAS, full analysis set; RD, reference denosumab.
[a]Mixed model for repeated measures with observed %CfB in lumbar spine BMD as the dependent variable; covariates are treatment arm (RGB-14-P and RD) and stratification factors at randomisation.

**P211**

**Mortality and Cardiovascular events in patients with severe Osteoporosis Treated with Romosozumab Followed by Antiresorptive Therapy: A Comparative Analysis with Teriparatide in a Single-Centre Study**

*Pollie Ravji[1], Elizabeth Orhadje[2], Nikita Bendstrup-Charlton[2], Kathryn Berg[2], Stuart Ralston[2], Barbara Hauser[1]*
*[1]NHS Lothian, Rheumatology Department, Edinburgh, United Kingdom*
*[2]Institute of Genetics and Cancer,*
*Bone and Musculoskeletal Research Group at University of Edinburgh, Edinburgh, United Kingdom*

**Abstract Text**

**Background:** Romosozumab (ROMO) is an osteoporosis treatment for patients at high fracture risk. Concerns regarding its cardiovascular (CV) safety were raised in the ARCH trial. Current SIGN guidelines recommend anabolic treatment for postmenopausal women with severe spinal OP.

**Purpose:** We studied serious adverse events (SAE) in patients started on ROMO followed by a 1 year of antiresorptive treatment (ART) versus those on a 24-month course of Teriparatide (TPTD).

**Methods:** Retrospective single-center analysis of postmenopausal women initiating either 12 months of ROMO + 12 months ART or 24 months of TPTD (February 2021–September 2024). Cardiovascular risk was assessed with QRISK3®. SAE were assessed through electronic record review and primary care contact.

**Results:** ROMO patients (n=100) were older (72.9 ± 9.1 vs.67.8 ± 8.3 years, p<.001), had lower BMI (23.5 ± 5.9 vs.25.6 ± 5.4 kg/m²,p=.004), higher baseline fracture risk (30.9 ± 12.4% vs.22.6 ± 10.1%, p<.001), and greater QRISK3 score (21.5 ± 12.3% vs.13.8 ± 10.1%, p<.001) than TPTD patients (n=107). In the ROMO group, there were 14 deaths (14%) over two years, including 11 during ROMO treatment. These involved 3 cardiac ischaemic and 1 cerebrovascular event. A further 5 patients (5%) experienced nonfatal CV events. In the TPTD group, 5 deaths (4.6%) and 4 CV events (3.7%) occurred. Although deaths were numerically greater in the ROMO group, treatment type was not identified as an independent risk for death in a multivariable logistic regression model; instead, this identified QRISK3 as independent predictor of mortality (OR 1.05,95% CI 1.01-1.09) when adjusting for treatment type, BMI and MOP fracture risk.

**Conclusion:** We found 9.1% of women with severe postmenopausal osteoporosis died within two years of initiating anabolic therapy and that this was associated with QRISK3 score but not treatment type. Further research is indicated to target anabolic therapy to patients most likely to experience clinical benefit.

**P212**

**Impact of AI-enabled vertebral fracture (VF) identification on Fracture Liaison Service (FLS) key performance indicators and treatment recommendations.**

Franz - NDORMS- Clemano[1], Janine Connor[2], Jane Threlkeld[3], Cassandra Chisholm[3], Daniel Chappell[4], Madhavi Vindlacheruvu[5], Emma Gerety[6], Jack Boylan[7], Jane Turton[7], Rachel Eckert[8], Michael Stone[7], Ken Poole[4], Opinder Sahota[9], Rafael Pinedo-Villanueva[1], Muhammad Kassim Javaid[10]
[1]University of Oxford,
Nuffield Department of Orthopaedics- Rheumatology and Musculoskeletal Sciences, Oxford, United Kingdom
[2]Bradford Teaching Hospitals NHS Foundation Trust, Fracture Liaison Service, Bradford, United Kingdom
[3]Bradford Teaching Hospitals NHS Foundation Trust, Radiology, Bradford, United Kingdom
[4]NIHR Cambridge Biomedical Research Centre, Department of Medicine, Cambridge, United Kingdom
[5]Cambridge University Hospitals NHS Foundation Trust, Orthogeriatrics, Cambridge, United Kingdom
[6]Cambridge University Hospitals NHS FT- Cambridge- UK, Department of Radiology, Cambridge, United Kingdom
[7]University Hospital Llandough- Cardiff and Vale University Health Board,
Bone Research Unit or Metabolic Bone Health Department, Cardiff, United Kingdom
[8]University of Oxford, Fracture Liaison Service, Oxford, United Kingdom
[9]Nottingham University Hospitals NHS Trust, Department of Medicine for Older People, Nottingham, United Kingdom
[10]University of Oxford, NDORMS, Headington, United Kingdom

**Abstract Text**

**Background:** VF identification remains challenging for FLSs. While AI models identify adults with vertebral fractures from existing CT scans, the impact on wider FLS performance has not been evaluated.

**Purpose:** To compare the KPIs of FLSs before and after introduction of AI-enabled VF identification in the NHS setting.

**Methods:** The Nanox-AI HealthVCF AI model was implemented in 3 FLSs to identify potential vertebral fractures from existing CT scans with additional funding for FLS administrators and nurses. The KPIs for identification, assessment, treatment recommendation and follow-up were compared before (Jan to Nov 2023) and after (Jan to Nov 2024) AI implementation using the FLS-Database of England and Wales. Each AI-FLS was supported to deliver patient co-produced service improvement. Up to 4 control FLSs for each AI-FLSs were identified, matching on spine fracture identification KPI in 2023 to account for secular changes. Differences in proportions were compared using two-proportion z-tests.

**Results:** Comparing pre- and post-AI, overall spine fracture identification increased from 25.1% to 55.4%, $p<0.001$, with no change in non-spine identification (28.9% vs 28.8%, $p=0.89$). For spine fracture patients, KPIs significantly worsened for time to assessment and DXA while falls assessment and treatment initiation by first follow-up improved (Figure). There were significant differences between AI-FLS sites. For non-spine fracture patients, KPIs for falls assessment slightly worsened while treatment recommendation and initiation by first follow-up significantly improved ($p<0.001$). In the control sites, there was a modest improvement in VF identification (28.6 vs 31.3%, $p=0.007$) and time to assessment, with no difference in treatment initiation by follow-up

**Conclusion:** AI implementation significantly improved VF identification, as expected, but worsened time to assessment despite additional FLS staff. There was an unexpected improvement in some KPIs for non-spine fracture patients, suggesting AI implementation benefited wider FLS performance and the importance of embedding service improvement when implementing AI.

**Figure**

Change in KPI achievement pre and post implementation of AI-enabled vertebral fracture identification in the FLS setting

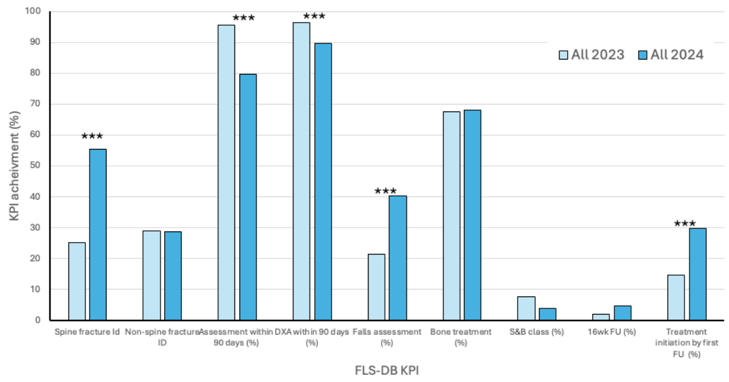

Legend: All= all active AI-FLSs
*p<0.05, **p<0.01, ***p<0.0001

### P213

### Clincal criteria for selecting patients identified using AI for Fracture Liaison Service assessment.

*Muhammad Kassim Javaid*[1], *Janine Connor*[2], *Jane Threlkeld*[3], *Daniel Chappell*[4], *Kenneth Poole*[4], *Jack Boylan*[5], *Jane Turton*[5], *Michael Stone*[5], *Elizabeth Gadd*[6], *Rachel Eckert*[7], *Tiago Santos*[8], *Mark Baxter*[9], *Charlotte Toogood*[10], *Elizabeth Curtis*[11], *Opinder Sahota*[12]

[1]*University of Oxford, NDORMS, Headington, United Kingdom*

[2]*Fracture Liaison Service, Bradford Teaching Hospitals NHS Foundation Trust, Bradford, United Kingdom*

[3]*Bradford Teaching Hospitals NHS Foundation Trust, Radiology, Bradford, United Kingdom*

[4]*NIHR Cambridge Biomedical Research Centre, Department of Medicine, Cambridge, United Kingdom*

[5]*University Hospital Llandough- Cardiff and Vale University Health Board, Bone Research Unit or Metabolic Bone Health Department, Cardiff, United Kingdom*

[6]*University Hospital Llandough- Cardiff and Vale University Health Board, Fracture Liaison Service, Cardiff, United Kingdom*

[7]*Oxford University Hospitals NHS Foundation Trust, Fracture Liaison Service, Oxford, United Kingdom*

[8]*University Hospital Southampton NHS Foundation Trust, Medicine for Older People, Southampton, United Kingdom*

[9]*University Hospital Southampton NHS Foundation Trust, Medicine for Older Peopl, Southampton, United Kingdom*

[10]*University Hospital Southampton NHS Foundation Trust, Fracture Liaison Service, Southampton, United Kingdom*

[11]*University of Southampton, MRC Lifecourse Epidemiology Centre, Southampton, United Kingdom*

*[12]Nottingham University Hospitals NHS Trust, Department of Medicine for Older People, Nottingham, United Kingdom*

**Abstract Text**

**Background:** As AI models are introduced into clinical practice, identifying patients who should not be prioritised for FLS assessment will determine the AI impact

**Purpose:** To describe the variation in clinical exclusion factors for patients identified with vertebral fracture using AI.

**Methods:** Six UK public hospitals that were targeted to use AI in their FLS pathway completed a survey describing local referral rules for patients identified by the AI.

**Results:** From the hospitals involved, there was a significant variation in the criteria for excluding patients (Table). In particular, site-to-site differences occurred in terms of cancer and renal status (table).

**Conclusion:** As AI technologies are introduced into routine clinical care, there is variation in which patients are referred for FLS assessment which may impact the value of the AI implementation. Further evidence is needed to inform guidelines for specific subgroups of patients who would not be suitable for FLS referral to ensure equitable access and benefit from AI.

**Table:** Clinical Criteria applied by different NHS hospitals in the UK to prioritise patients for FLS assessment

| Criteria | Hospital A | Hospital B | Hospital C | Hospital D | Hospital E | Hospital F |
|---|---|---|---|---|---|---|
| Teaching hospital | No | Yes | Yes | Yes | Yes | Yes |
| Exclusion Criteria: | | | | | | |
| Age | Nil | Nil | Over 75 years old and only one VF, automatically referred for primary care management | Nil | Nil | NIl |
| Cancer | Nil | Any Cancer | Cancer if Palliative / hospice care | Nil | New diagnosis, active oncology surveillance, metastatic disease | Nil |
| Renal | Nil | CKD 4,5 or Dialysis | Nil | Nil | CKD 4, 5 or dialysis | CKD 5 or dialysis |

| | | | | | | |
|---|---|---|---|---|---|---|
| Previous secondary care engagement | DXA in last 2 years | Nil | Bone clinic appointment/DXA in last year | Nil | Nil | Nil |
| Other | Nil | Terminal stage of chronic disease, life expectancy less than 12 months, Significant daily activity dependency | Palliative care | Nil | Terminal stage of chronic disease, life expectancy less than 12 months, Significant daily activity dependency | Palliative care |

**P214**

**Organizational predictors of higher Fracture Liaison Service (FLS) performance in England and Wales: The Radiate study**

*Franz - NDORMS- Clemeno[1], Maggie Fielding[2], Karen Whitehead[3], Rosie Dickinson[4], Antony Johansen[5], Celia Gregson[6], Rafael Pinedo-Villanueva[1], Muhammad Kassim Javaid[7]*
*[1]University of Oxford, Nuffield Department of Orthopaedics- Rheumatology and Musculoskeletal Sciences, Oxford, United Kingdom*
*[2]Royal Osteoporosis Society, Patient engagement, Bath, United Kingdom*
*[3]Pregnancy Associated Osteoporosis, Patient Engagement, Bath, United Kingdom*
*[4]Royal College of Physicians, The Fragility Fracture and Falls Audit Programme, London, United Kingdom*
*[5]Cardiff University, School of Medicine, Cardiff, United Kingdom*
*[6]University of Bristol, Musculoskeletal Research Unit, Bristol, United Kingdom*
*[7]University of Oxford, NDORMS, Headington, United Kingdom*

**Abstract Text**

**Background:** While FLSs are designed to deliver secondary fracture prevention, significant differences exist between services.

**Purpose:** To describe the organisational predictors of FLS performance as measured by key performance indicators (KPIs).

**Methods:** 2017, 2020 and 2022 KPI data from the FLS-DB of England and Wales facilities and clinical audit were analysed to identify potential "high-performance" FLSs using K-means clustering. The optimal number of clusters was determined using the NbClust package. The organisational characteristics of the FLSs were compared between the clusters by bootstrap logistic models. The organisational characteristics included the fracture types identified, time to assessment, treatment recommendations, monitoring methods, total work time equivalent (WTE) spent by different staff members within the FLS per week and the estimated caseload. Within cluster associations were tested using Pearson correlations.

**Results:** 61 FLSs were identified in 2017. The optimal number of clusters was two with significant differences in KPI for DXA with 90 days, proportion recommended treatment, strength and balance testing and follow-up measures. 20/28 (71%) FLSs remained high performing from 2017-22, and 23/24 (96%) FLSs remained low performing. Of the organisational parameters, monitoring by an FLS coordinator predicted higher performance, and the delegation of monitoring to primary care physicians predicted low performance. While in 2017, the total nurse WTE predicted the number of patients submitted in the high but not low-performing clusters, there was no difference by 2022 (Figure).

**Conclusion:** FLS cluster assignment was relatively stable before and after the COVID-19 pandemic. How FLSs delegate monitoring predicted better overall FLS KPI performance. While there was little relationship between staff numbers and cases submitted in the low-performing FLSs in 2017, in 2022, both clusters demonstrated a significant relationship, suggesting improvement in case identification since implementing the FLS-DB.

Figure: Relationship between FLS nurse whole-time equivalents and submitted caseload in 2017 and 2022 by cluster type.

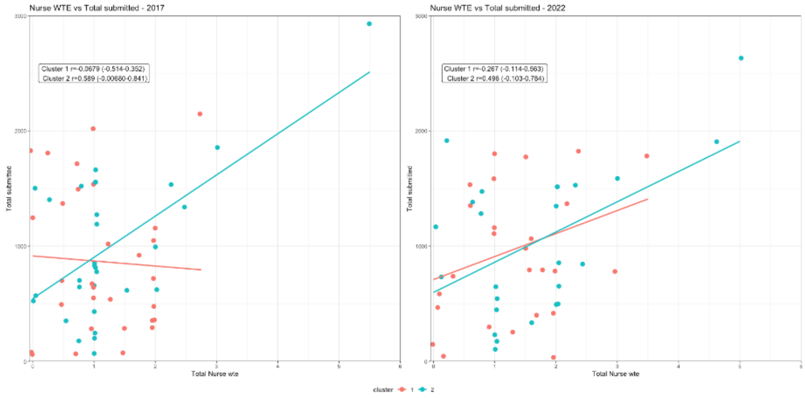

**P215**

**Real-world efficacy analysis of 12-month Romosozumab followed by 12-month Antiresorptive Treatment compared to 24-month Teriparatide**

*<u>Elizabeth Orhadje</u>[1], Pollie Ravji[2], Nikita Bendstrup-Charlton[1], Kathryn Berg[1], Stuart H Ralston[1], Barbara Hauser[2]*
*[1]Institute of Genetics and Cancer,*
*Bone and Musculoskeletal Research Group at University of Edinburgh, Edinburgh, United Kingdom*
*[2]NHS Lothian, Rheumatology Department, Edinburgh, United Kingdom*

**Abstract Text**

**Background:** Romosozumab (ROMO) and Teriparatide (TPTD) are established anabolic osteoporosis treatments for patients at very high fracture risk, with proven efficacy in reducing vertebral fractures[1,2]. However, no direct comparisons exist to guide treatment selection.

**Purpose:** This study aimed to evaluate bone mineral density (BMD) responses at 12 and 24 months in patients treated with ROMO followed by antiresorptive treatment (ART) versus TPTD.
**Methods:** A retrospective single-centre analysis included patients treated with ROMO for 12 months followed by ART or TPTD for 24 months between February 2021 and September 2024. Data were extracted from electronic health records.

**Results:** The ROMO group (n=111) was older (73.1 ± 9.0 vs.68.1 ± 8.1 years,p<.001) and had a lower BMI (23.4 ± 5.9 vs.25.6 ± 5.4 kg/m²,p<.001) than the TPTD group (n=107). More TPTD patients were treatment-naïve (65.1% vs.45.8%,p=.006). Baseline lumbar spine BMD was comparable (T-score: -3.86 ± 1.0), but femoral neck BMD was lower in the ROMO group (-3.15 ± 0.68 vs.-2.75 ± 0.76; p<.001). Most patients had a 1 yr scan however due to various reasons (frailty, death, clinical decision) only 41 ROMO patients and 68 TPTD patients had a 2 yr DEXA scan.

ROMO resulted in greater lumbar spine BMD gains at 12 months (p=.004), but no differences were observed between groups at 24 months (Fig1A). Femoral neck BMD gains were superior in the ROMO group at both time points (p=.034)(Fig1B). Treatment-naïve patients demonstrated significantly greater increases in lumbar spine BMD at 24 months (16.9 ± 10.0% vs.10.6 ± 8.2%, p=.001) compared to pre-treated patients, regardless of the treatment type

**Conclusion:** ROMO patients had more severe osteoporosis at baseline. ROMO demonstrated faster lumbar spine BMD improvement but similar results to TPTD at 24 months. Femoral neck BMD gains were greater with ROMO. Prior antiresorptive therapy blunted lumbar spine BMD responses, highlighting the importance of treatment sequencing.

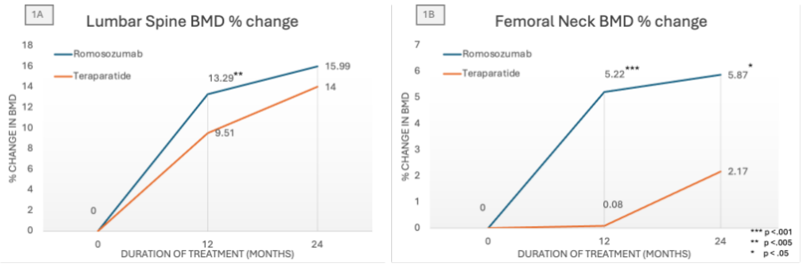

### P217

### The Osteoporosis Treatment Gap in Ireland: Reality or Ecologic Fallacy?

*Lan Yang[1], Brennan Attracta[2], Bryan Whelan[1], Damian Gonzalez Garza[1], Erjiang Erjiang E[3], Tingyan Wang[4], Rebecca Egan[5], Kelly Gorham[5], Fiona Heaney[5], Catherine Armstrong[5], Guadalupe Morote Ibarrola[5], Amina Gsel[5], Ming Yu[6], Mary Dempsey[7], John Carey[1]*
[1]*University of Galway, School of Medicine, Galway, Ireland*
[2]*University of Galway, School of Computer Science, Galway, Ireland*
[3]*Guangxi Minzu University, School of Management, Nanning, China*
[4]*University of Oxford, Nuffield Department of Medicine, Oxford, United Kingdom*

[5]University Hospital Galway, Department of Rheumatology, Galway, Ireland
[6]Tsinghua University, Department of Industrial Engineering, Beijing, China
[7]University of Galway, School of Engineering, Galway, Ireland

**Abstract Text**

**Objective:** The osteoporosis treatment gap reflects the difference between those who should be treated and those who are. An European report suggests this is 32% in Ireland; our experience suggests otherwise. We investigated the treatment gap in Irish men and women.

**Materials and Methods:** We investigated treatment rates among patients in a previously described West of Ireland cohort: The DXA HIP Project, following ethical approval. Development, validation and clinical features of this cohort are published. We compared calcium and vitamin D and osteoporosis medication (bisphosphonates, denosumab, oestrogen, raloxifene and teriparatide) treatment in 2 groups: those referred for a DXA scan, and those who had a DXA scan. Subjects were divided into 3 risk groups based on treatment indications: low risk (none), moderate risk (at least one) and high risk (2 or more).

**Results:** 978 (28%) of patients in the referral group and 2090 in the post scan group had data available for analysis. In the referral group 271 had a prior fracture, 707 did not. Calcium and vitamin D was prescribed for 48% of the former and 45% of the latter, while 30% of the former and 35% of the latter were prescribed osteoporosis medication. The majority of the post DXA group were prescribed Calcium and vitamin D, irrespective of risk, shown in table below. In contrast, the majority of at risk men and women were not prescribed osteoporosis medication. Of particular concern fewer than 1 in 3 men and 1 in 2 women deemed high risk were prescribed appropriate therapy.

**Conclusions:** The real osteoporosis treatment gap in Ireland remains unacceptably large. The number of unnecessary treatment among those at low risk has created a false (ecologic fallacy) that this number is quite small. Work is needed to understand and improve the reasons behind ineffective prescribing.

| Variable | | Low Risk | Intermediate Risk | High Risk |
|---|---|---|---|---|
| Calcium and Vitamin D | Men | 60% | 48% | 58% |
| | Women | 53% | 48% | 70% |
| Osteoporosis Medication | Men | 21% | 21% | 31% |
| | Women | 16% | 28% | 46% |
| Both | Men | 17% | 16% | 22% |
| | Women | 14% | 23% | 37% |

**P219**

### Safety of teriparatide treatment of osteoradionecrosis of the jaw: A Scoping Review, on behalf of the ECTS Clinical Action Group

*John Carey[1], Rola Alwaheidi[2], Willem F Lems[3], Athanasios D Anastasilakis[4], Maria P Yavropoulou[5], Julien Paccou[6], Anda M Naciu[7], Tim Rolvien[8], Stella D'Oronzo[9]*
[1]University of Galway, School of Medicine, Galway, Ireland

[2]University of Galway- Ireland, Medicine, Galway, Ireland
[3]Amersterdam University Medical Centre, Rheumatology, Amsterdam, Netherlands
[4]Military General Hospital, Endocrinology, Thessaloniki, Greece
[5]First Department of Propaedeutic and Internal Medicine- Medical School- National and Kapodistrian University of Athens-, Endocrinology Unit, Athens, Greece
[6]University of Lille, Rheumatology, Lille, France
[7]Campus Bio-Medico University, Endocrinology and Diabetes, Rome, Italy
[8]University Medical Center Hamburg-Eppendorf, Trauma and Orthopaedic Surgery, Hamburg, Germany
[9]Section of Internal Medicine and Clinical Oncology- University of Bari Aldo Moro, Biomedical Sciences and Human Oncology-, Bari, Italy

**Abstract Text**

**Background**: Osteoradionecrosis of the jaw is a significant comorbidity among cancer patients, particularly adults who receive radiotherapy for head and neck cancer. Treatment is complex, while morbidity and mortality are high. Osteoanabolic therapy, such as teriparatide, has shown promise for radiation-induced bone damage in experimental studies and osteonecrosis. However, teriparatide use is restricted in cancer patients due to concerns about osteosarcoma risk, particularly among people with previous radiotherapy. The FDA removed this restriction in November 2020 despite limited data on treating patients with prior radiotherapy. **Purpose**: To assess the safety of teriparatide treatment of osteoradionecrosis in cancer patients.

**Methods**: We conducted a scoping literature review in Medline (Ovid) and Embase from inception to November 2024 of studies using teriparatide treatment of osteoradionecrosis in cancer patients. Eligible studies included full-text publications in English treating people, including RCTs, observational studies and case reports.

**Results**: 308 articles were identified in the initial search, 31 underwent full-text review. Only one article met our inclusion criteria detailing two case reports: a 53-year-old woman and an 83-year-old man. Both were diagnosed with tongue cancer and received radiotherapy: 60 Gy and 61.2 Gy, respectively. Teriparatide therapy for 4 to 6 months was used to manage refractory osteoradionecrosis (ORN). Reported follow-up duration was 15 and 27 months. Both cases demonstrated significant clinical improvements, including symptom relief, closure of the orocutaneous fistula, increased radiopacity, and regeneration of bone defects. Metastatic squamous cell carcinoma (SCC) lesions were detected in one patient at 1 and 10 months following teriparatide therapy, successfully treated with locoregional interventions.

**Conclusion**: Short-term treatment with teriparatide therapy in patients with severe, refractory osteoradionecrosis appears promising. However, the safety of teriparatide in this population remains uncertain due to the limited number of published reports.

**P220**

**Evaluating the safety of teriparatide treatment following radiotherapy. A Scoping Review, on behalf of the ECTS Clinical Action Group.**

John Carey[1], Rola Alwaheidi[2], Willem F Lems[3], Athanasios D Anastasilakis[4], Maria P Yavropoulou[5], Julien Paccou[6], Anda M Naciu[7], Tim Rolvien[8], Stella D'Oronzo[9]
[1]University of Galway, School of Medicine, Galway, Ireland
[2]University of Galway, Medicine, Galway, Ireland
[3]Amsterdam University Medical Center-, Rheumatology, Amsterdam, Netherlands
[4]Military General Hospital-, Endocrinology, Thessaloniki, Greece
[5]Department of Propaedeutic and Internal Medicine- Medical School- National and Kapodistrian University of Athens, Endocrinology, Athens, Greece
[6]University of Lille, Rheumatology, Lille, France
[7]Campus Bio-Medico University, Endocrinology and Diabetes, Rome, Italy
[8]University Medical Center Hamburg-Eppendorf, Trauma and Orthopaedic Surgery-, Hamburg, Germany
[9]Section of Internal Medicine and Clinical Oncology- University of Bari Aldo Moro, Biomedical Sciences and Human Oncology-, Bari, Italy

**Abstract Text**

**Background**: Osteoporotic fractures (OF) are a significant comorbidity among cancer patients, particularly adults with reproductive cancers receiving hormonal therapy and radiotherapy. Antiresorptive therapies are widely used in at risk patients, but some will experience OF. Osteoanabolic therapy, such as teriparatide, is more effective than antiresorptive treatments for patients with severe osteoporosis. However, teriparatide use is restricted in cancer populations in Europe due to concern about an osteosarcoma risk, particularly among people with previous radiotherapy. The FDA removed this restriction in November 2020 despite limited data on treating patients with prior radiotherapy. **Purpose**: To assess the safety of teriparatide treatment of osteoporosis in cancer patients with a history of radiotherapy.

**Methods**: We conducted a scoping literature search in Medline (Ovid) and Embase from inception to November 2024 of studies using teriparatide treatment of osteoporosis in cancer patients treated with radiotherapy. Eligible studies include full-text publications of people, in English, including RCTs, observational studies and case reports.

**Results**: We identified 819 articles in the initial search; 76 underwent full text review. Only 3 met our inclusion criteria: 2 case reports and 1 case series of 6 patients. 5 subjects were female, mean age: 65 years, including cervical, prostate, lymphoma, breast and skin cancer.,6 patients completed 18 to 24 months of therapy. Dose and duration of radiation was not specified in all cases.

Follow-up duration was up to 4.7 years. BMD improved and no incident fractures were reported. One patient was diagnosed with osteosarcoma 2 months after commencing teriparatide; another was diagnosed with vaginal cancer after completing teriparatide treatment. **Conclusion**: Our literature search found favorable responses in BMD and no incident fractures. However, the safety of teriparatide in this population remains uncertain due to the limited number of publications addressing this issue.

**ND-P018**

**Impact of Drug Holidays and Their Duration on MRONJ Risk Reduction in Patients Undergoing Invasive Dental Procedures**

*Jin-Woo Kim[1], Junghyun Park[1], Sunghye Kong[2], Hyojung Lee[3]*
*[1]Ewha Womans University, Oral and Maxillofacial Surgery, Seoul, Korea- South*
*[2]Seoul National University, Endocrinology, Seoul, Korea- South*
*[3]Seoul National University, Periodontics, Seoul, Korea- South*

**Abstract Text**

Medication-related osteonecrosis of the jaw (MRONJ) is a significant concern for patients undergoing invasive dental procedures after bisphosphonate use. However, robust evidence regarding the optimal duration of a drug discontinuation before dental procedures remains limited. This nationwide cohort study using the Korean National Health Information Database investigated the impact of the time since the last dose (TSL) —commonly referred to as a 'drug holiday'— of intravenous (IV) bisphosphonate on MRONJ incidence in osteoporosis patients. We analyzed 152,299 individuals aged 65 or older, diagnosed with osteoporosis between 2009 and 2020, who received IV bisphosphonates. Participants were categorized based on the TSL. The primary outcome was MRONJ occurrence. Results showed that TSL exceeding 90 days significantly reduced MRONJ risk, with hazard ratios (HRs) of 0.57 for 91–180 days, 0.45 for 181–365 days, and 0.43 for >365 days, compared to ≤90 days. Notably, only durations exceeding 365 days showed significant risk reduction for zoledronate users, whereas ibandronate users consistently exhibited risk reduction across all time categories. These findings underscore the need for personalized MRONJ prevention strategies based on the specific IV bisphosphonate used.

**ND-P019**

**Risk of bisphosphonate treatment for osteoporosis in patients undergoing invasive dental procedures**

*Roxana Novac[1], Marius Novac[2]*
*[1]Emergency Hospital Moinesti, endocrinology, Bacau, Romania*
*[2]Clinica Dr. Novac, Dentistry, Bacau, Romania*

**Abstract Text**

As we well known there has been a concern regarding the performance of invasive dental procedures in patients undergoing bisphosphonates in the fact that association with bisphosphonate-related osteonecrosis of the jaws (BRONJ) following invasive dental procedures poses a significant challenge.

**Purpose:** The aim of this study was to detect the incidence of bisphosphonate-related osteonecrosis of the jaws (BRONJ) in association with osteoporosis between 2014-2024 in a single center dental clinic. The aim of this retrospective study was to examine the outcome of patients referred to a dedicated clinic for dental extractions, dental implants, parodontotic procedures while they were either oral or intra-venous (IV) bisphosphonates (BPs). The following parameters were assessed: mode of BP administration, indication for BP prescription, incidence of BRONJ, concomitant risk factors for development of bisphosphonate-related osteonecrosis of the jaws (BRONJ) and demographic details.

**Results:** On a period of almost 10 years the clinic had 140 patients diagnosed with osteoporosis and taking treatment with bisphosphonates, 110 were prescribed oral and 30 iv BPs, women 92,14% (129), 8,57 % (12) had also a history of corticosteroid treatment for more than 3 months, and only 15,71% (22) of them had undergone treatment for more than 4 years. We also looked in other comorbidities like diabetes, anticoagulant therapy, parodonthosis. We didn't identify any case of BRONJ during this period. Each of the patients had been closely monitored for about 6 weeks after the dental procedure or until complete restoration of the incision. We also stopped the treatment the week before the intervention and until complete healing, collaborating with the prescribing doctor of our patients.

**Conclusion:** Using all the precaution measures (two-plan sutures, before-intervention cleaning, keeping a high dental hygiene, glycemic control, adjustment of anticoagulant therapy) and close collaboration with the patients' referrals can be the key to minimize the risk of BRONJ.

**Poster Sessions: Other diseases of bone and mineral metabolism**

**P221**

### Proteomic identification of natural inhibitors of calcium oxalate renal calculi in the urine of non-stone/healthy subjects

*Piyaporn Rattananinsruang[1], Paleerath Peerapen[1], Visith Thongboonkerd[1]*
*[1]Siriraj Hospital- Mahidol University, Medical Proteomics Unit- Research Department, Bangkok, Thailand*

**Abstract Text**

**Background/Introduction:** With the increasing number of renal stone patients, awareness has been raised to control the rise of disease prevalence.

**Purpose:** Our purpose was to identify natural inhibitors of calcium oxalate (CaOx) stones in the urine of non-stone/healthy subjects that could serve as targets for disease prevention.

**Methods:** Urinary proteins were fractionated by diethylaminoethyl (DEAE) anion exchange followed by GigaCap Q-650 M column chromatography. The fractionated protein samples (Q1-Q9) were then assayed toward CaOx-inhibitory activities. The potent inhibitory fractions were characterized by tandem mass spectrometry.

**Results:** Q3, Q4, Q5 and Q6 provided the most potent inhibitory activities against CaOx crystals. NanoLC-ESI-Qq-TOF MS/MS identified 49-78 proteins from each of these fractions. From all identified proteins, 26 were found in all these 4 inhibitory fractions, and 38 have been reported with lower urine levels in CaOx stone patients than in non-stone/healthy subjects. Interestingly, 5 of these proteins (vitronectin, trefoil factor 1, prothrombin, protein AMBP and ceruloplasmin) are the known inhibitors supported by experimental evidence from previous reports.

**Conclusions:** We have identified a large number of natural inhibitors of CaOx stones from normal human urine after fractionation of urinary proteins followed by crystal assays and tandem mass spectrometry. These potential CaOx stone inhibitors may serve as the targets to prevent CaOx renal stones.

**P222**

**Pathogen-associated molecular patterns enhance neurogenic heterotopic ossifications after spinal-cord injury**

*Selwin G Samuel[1], Hsu-Wen Tseng[1], Dorothée Girard[2], Bastien Rival[2], Valérie Barbier[1], Marjorie Salga[3], Whitney Fleming[1], François Genêt[3], Sébastien Banzet[2], Kylie A Alexander[1], Jean Pierre Levesque[1]*
*[1]Mater Research Institute - The University of Queensland, Chronic Diseases, Woolloongabba, Australia*
*[2]INSERM, Institut de Recherche Biomédicale des Armées, Clamart, France*
*[3]Assistance Publique-Hôpitaux de Paris, Hôpital Raymond Poincaré, Garches, France*

**Abstract Text**

**Background:** Neurogenic heterotopic ossification (NHO) is a complication of spinal cord injury (SCI) which manifest as heterotopic bones in periarticular muscles. The mechanisms leading to NHO are largely unknown and treatment remains surgical resection. Retrospective studies report have shown higher NHO prevalence in patients with infections. We previously established a functional association between gram-negative bacterial infections and NHO development and demonstrated that the pathogen associated molecular pattern (PAMP) lipopolysaccharide exacerbated NHO development in mice.

**Purpose:** Further evaluate if fungal, viral, and other bacterial PAMPs also exacerbate NHO development in mice.

**Method:** Using our model of NHO following SCI, where mice undergo a SCI together with a muscle injury induced by an intramuscular injection of cardiotoxin, we administered purified PAMPs post-surgery that mimic fungal (Zymosan), viral (Poly I:C and Gardiquimod) and bacterial infections (Pam2CSK4, Pam3CSK4, CpG oligodeoxynucleotide and lipoteichoic acid) and measured NHO bone volumes by microCT.

**Results:** NHO volumes were quantified at 7 and 21 days post-surgery demonstrate that fungal, viral and bacterial PAMPs all significantly exacerbated NHO development in a dose dependent manner (Fig1A-C $p<0.05$, $p<0.01$, $p<0.001$). These PAMPs also enhanced calcium mineralisation of mouse muscle fibro-adipogenic progenitors (FAPs), the NHO cell of origin, via macrophages, as conditioned media from bone marrow derived macrophages (BMDMs) stimulated with PAMPs, significantly increased FAP calcium mineralisation when added into FAP osteogenic cultures (Fig1D $p< 0.0001$).

Finally, we found that macrophages, and in some cases muscle FAPs, expressed the receptors for these PAMPs suggesting that the effects of PAMPs on NHO could have contributions from both myeloid and mesenchymal cells in muscle.

**Conclusion:** This study highlights the necessity for infection vigilance and prophylaxis to reduce incidence of NHO in victims of SCI or TBI.

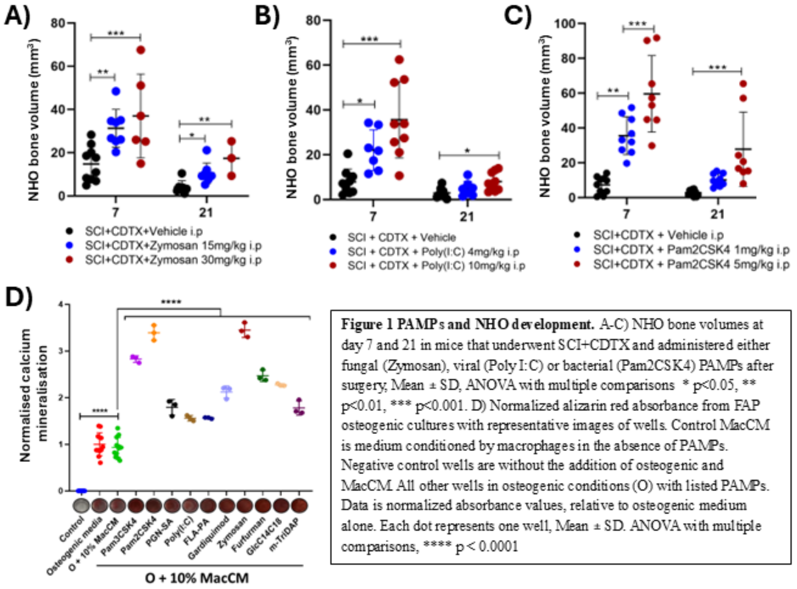

**Figure 1 PAMPs and NHO development.** A-C) NHO bone volumes at day 7 and 21 in mice that underwent SCI+CDTX and administered either fungal (Zymosan), viral (Poly I:C) or bacterial (Pam2CSK4) PAMPs after surgery, Mean ± SD, ANOVA with multiple comparisons * $p<0.05$, ** $p<0.01$, *** $p<0.001$. D) Normalized alizarin red absorbance from FAP osteogenic cultures with representative images of wells. Control MacCM is medium conditioned by macrophages in the absence of PAMPs. Negative control wells are without the addition of osteogenic and MacCM. All other wells in osteogenic conditions (O) with listed PAMPs. Data is normalized absorbance values, relative to osteogenic medium alone. Each dot represents one well, Mean ± SD. ANOVA with multiple comparisons, **** $p < 0.0001$

### P223

### Exploratory study on bone microarchitecture in post-mortem specimens of various skeletal sites from dialysis-dependent patients

*Matthias Fuchs[1], Johannes Werzowa[1], Marija Bojic[1], Barbara Misof[2], Stéphane Blouin[2], Marlis Huber[3], Alexander Nader[3], Markus Hartmann[2], Jochen Zwerina[2]*

*[1]1st Medical Department, Hanusch Hospital, Vienna, Austria*

*[2]Ludwig Boltzmann Institute of Osteology, Hanusch Hospital and AUVA Trauma Centre Meidling, Vienna, Austria*

*[3]Department of Pathology, Hanusch Hospital, Vienna, Austria*

### Abstract Text

**Background:** Bone fragility is high in dialysis patients. Clinical practice guidelines recommend transiliac bone biopsies in patients with severe chronic kidney disease (CKD), which might provide important information about the type of renal osteodystrophy and for treatment decision, however, its diagnostic power for bone properties of other, more fragile skeletal sites is unknown.

**Purpose:** We compared bone microarchitecture among various skeletal sites.

**Methods:** The trabecular and cortical bone microarchitecture of iliac crest, femur and lumbar vertebrae was analyzed in 21 patients with dialysis-dependent CKD (aged 79 [69; 81.50] years, median [IQR]) using micro-computed tomography (SCANCO µCT50). Iliac crest outcomes were compared to data from a healthy reference group (REF, aged 79.5 [66.25; 84.75] years). Additionally, within the CKD cohort, cortical and trabecular bone microarchitecture of the iliac crest were compared pairwise and correlated to that of the femur and vertebrae, respectively.

**Results:** Compared to REF, the CKD group exhibited significantly lower iliac crest bone volume (BV/TV -44%, $p<0.001$) and trabecular thickness (Tb.Th -43%, $p<0.001$), while trabecular number (Tb.N) or separation (Tb.Sp) were not different. Cortical porosity (Ct.Po.V/TV +298%) and pore number (Ct.Po.N +86%) were elevated, while separation thereof (Ct.Po.Sp -15%) was reduced (all $p<0.001$) compared to REF. Within CKD patients, trabecular outcomes of iliac crest and vertebrae were not different, and Tb.Th of these sites was positively correlated (R=0.67, $p<0.001$). Ct.Po.V/TV (+230%, $p<0.001$) and pore diameter (Ct.Po.Dm +27%, $p<0.05$) were found larger, and Ct.Po.Sp (-12%, $p<0.05$) lower in the iliac crest than in the femur.

**Conclusion**: Our findings indicate that dialysis-dependent patients exhibit compromised bone microarchitecture at the iliac crest. While trabecular microarchitecture of the iliac crest appears predictive for that of the vertebrae, differences in cortical microarchitecture might limit iliac crest bone as a potential marker for fracture risk assessment at cortical bone dominated sites such as the femur.

**P224**

### BMP4 exacerbates the chondrogenic differentiation potential of human fibro-adipogenic progenitors implicated in neurogenic heterotopic ossifications

*Juliette Fernandez[1], Draï Maëva[1], Sébastien Banzet[2], Dorothée Girard[3]*
*[1]Institut de Recherche Biomédicale des Armées IRBA, Unité Thérapie Cellulaire et Tissulaire U.TCT, Clamart, France*
*[2]Centre de Transfusion Sanguine des Armées CTSA, Recherche et Innovations, Clamart, France*
*[3]Institut de Recherche Biomédicale des Armées IRBA, Unité Traumatologie de Guerre U.TG, Clamart, France*

### Abstract Text

Neurogenic heterotopic ossifications (NHO) are intra-muscular ectopic bone formations that develop following central nervous system (CNS) damage such as spinal cord injury (SCI). NHOs occur in the vicinity of major joints and induce ankylosis, pain, nervous and vascular compressions, ultimately impairing patient recovery. The combination of a severe CNS lesion and macrophages-mediated inflammation deeply alters muscle microenvironnement and stimulates the chondro-osteogenic differentiation of fibro-adipogenic progenitors (FAPs). Evidence of circulating BMP4$^+$ monocytes following SCI has been reported. In this study, we aimed to investigate the effect of BMP4 on the *in vitro* chondrogenic differentiation of human FAPs.

Human PDGFRα$^+$CD56$^-$ FAPs were isolated from muscle surrounding NHO and sorted using flow cytometry. $2{,}5.10^5$ FAPs were cultured as spheroids in TGF-β3 chondrogenic differentiation medium or control medium supplemented with increasing concentration of BMP4 (0, 25, 50 to 100 ng/ml). After 21 days, spheroids were collected for Safranin O/Fast Green staining, RT-qPCR analysis of chondrogenic genes expression and immunohistochemistry staining of Sox9, Aggrecan and Collagen 2.

Our results show that the addition of BMP4 induces a strong positive Safranin O staining suggesting a production of proteoglycans and the establishment of cartilaginous-like matrix during FAPs chondrogenic differentiation. BMP4 also stimulates the gene expression of key chondrogenic transcription factors *SOX9*, *SOX5*, *SOX6* and increases the gene expression of extracellular matrix proteins *COL2A1*, *COL10A1* and *ACAN*. Finally, we observed an increased Sox9, Collagen 2 and Aggrecan protein expression in FAPs spheroids cultured with BMP4 further confirming its stimulatory effect.

This study confirms that FAPs implicated in NHOs have the capacity to differentiate into chondrocytes *in vitro* and provide first evidence that BMP4 can exacerbate their chondrogenic differentiation potential.

**P225**

**Bone mineral density and bone turnover markers in a pilot cohort of chronic hypoparathyroidism patients**

*Marko Stojanovic[1,2], Sandra Pekic[1,2], Dragana Miljic[1,2], Mirjana Doknic[1,2], Zvezdana Jemuovic[1], Ivana Cekic[1], Ivan Jevtic[1], Marina Nikolic-Djurovic[1,2]*
*[1]Clinic for Endocrinology- Diabetes and Metabolic Diseases- University Clinical Center of Serbia, Department of Neuroendocrinology, Belgrade, Serbia*
*[2]Medical Faculty- University of Belgrade, Department of Internal Medicine, Belgrade, Serbia*

**Abstract Text**

**Background:** Hypoparathyroidism (HypoPTH) is a rare congenital or acquired condition hallmarked by hypocalcaemia, hyperphosphatemia and lack of active vitamin D (AVD). Conventional treatment is limited by risk of renal and ectopic calcifications. PTH and AVD are instrumental in bone remodelling. Fracture risk in HypoPTH may be elevated despite bone mineral density (BMD) often described as normal or increased.

**Purpose:** This cross-sectional pilot study was aimed at assessing the relevance of bone mineral density and bone turnover markers analysis in patients with hypoparathyroidism.

**Patients and Methods:** We analysed 9 patients with surgical (n=6) or non-surgical (n=3) HypoPTH lasting for 11.33±3.18 years and treated with tolerable AVD doses and minimal necessary calcium supplementation. Analysis included 7 females and 2 males, 38.33±6.32 years old with high-normal BMI of 26.60±3.14 kg/m$^2$. BMD was analysed by DXA at L1-L4 spine, femoral neck (FN) and mid-distal radius (MDR) and expressed as Z-score (Zsc) of BMD. Serum bone turnover markers analysis included fasting morning osteocalcin (OC), ALP and beta-cross-laps (BCL). Serum marker values were expressed as index denominated by the upper limit of normal age and gender reference values (ULN).

**Results:** The average DXA Zsc were +0.69± 0.43 (at L1-L4); +0.94± 0.45 (at FN); and -0.8±0.55 (at MDR). Three patients (33.33%) qualified by DXA as having osteopenia, and none as having osteoporosis. None reported previous pathological fractures. Average OC was 0.44±0.03 ULN. Average BCL was 0.60±0.13 ULN. Average ALP was 0.66± 0.13 ULN.

**Conclusion:** In our investigated pilot cohort of patients with chronic hypoparathyroidism treated with conventional therapy, cortical bone mineral density (assessed at MDR) was borderline low and bone formation (assessed by serum osteocalcin value) was lower than mid-normal age and gender reference range. This supports the relevance for further follow-up of bone health parameters in our HypoPTH patients.

**Keywords:** PTH, DXA, osteocalcin, BCL, ALP

**P226**

**Diagnostic agreement of bone histomorphometry for a diagnosis of renal osteodystrophy**

*Hanne Skou Jørgensen[1,2], Marie-Helene Lafage-Proust[3], Fabiana Gouveia Alves[4], Ana Carina Ferreira[5], Anibal Ferreira[5], Maude C Gerbaix[6], Heikki Kröger[7], Christa Maes[8], Elena Nefyodova[8], Kenneth Poole[4], Linda Skingle[4], Xiao Yu Tong[7], Andrea Trombetti[9], Pieter Evenepoel[10,11], Nathalie Bravenboer[12]*
*[1]Aarhus University, Department of Clinical Medicine, Aarhus, Denmark*
*[2]Aalborg University Hospital, Department of Nephrology, Aalborg, Denmark*
*[3]INSERM U1059 SAINBIOSE Université Jean Monnet-, Service de Rhumatologie, Saint-Etienne, France*
*[4]University of Cambridge- Addenbrooke's Hospital, NIHR Cambridge Biomedical Research Centre & Department of Medicine, Cambridge, United Kingdom*
*[5]Hospital Curry Cabral, Department of Nephrology, Lisbon, Portugal*
*[6]Geneva University Hospitals, Service of Bone Diseases- Department of Medicine, Geneva, Switzerland*
*[7]University of Eastern Finland, Kuopio Musculoskeletal Research Unit KMRU, Kuopio, Finland*
*[8]KU Leuven, Laboratory of Skeletal Cell Biology and Physiology SCEBP- Skeletal Biology and Engineering Research Center SBE- Department of Development and Regeneration, Leuven, Belgium*
*[9]Geneva University Hospitals and Faculty of Medicine, Service of Bone Diseases- Department of Medicine-, Geneva, Switzerland*
*[10]Katholieke Universiteit Leuven, Department of Microbiology- Immunology and Transplantation- Nephrology and Renal Transplantation Research Group, Leuven, Belgium*
*[11]University Hospitals Leuven, Department of Medicine- Division of Nephrology, Leuven, Belgium*
*[12]Amsterdam University Medical Centers, Laboratory Science, Amsterdam, Netherlands*

**Abstract Text**

**Introduction:** An iliac bone biopsy remains the gold standard for evaluating renal osteodystrophy (ROD). Lack of standardization in procedure, sample handling, and reporting hinders collaboration. This study assessed diagnostic agreement across laboratories performing histomorphometry for ROD evaluation.

**Methods:** Seven-millimeter transiliac biopsy slices from five cases were scanned for analysis by six laboratories. Parameters included histomorphometry, TMV classification, and clinical diagnosis. Agreement was analyzed using Kappa statistics and intraclass correlation coefficients (ICC).

**Results:** Static parameter agreement varied widely. High agreement was observed for bone area (ICC 0.91), osteoid perimeter (ICC 0.84) and osteoid area (ICC 0.95). For dynamic parameters, agreement was high for mineralizing surface (ICC 0.87) and bone formation rate (ICC 0.74), but poor for mineral apposition rate (ICC -0.13), adjusted apposition rate (ICC 0.13) and mineralization lag time (ICC 0.07). Agreement for TMV classification was substantial for bone turnover (Kappa 0.77), poor for bone mineralization (Kappa 0.14), and moderate for bone volume (Kappa 0.59). Clinical diagnoses showed high consistency across laboratories (Table 1).

**Conclusions:** Despite inter-reader variability in bone histomorphometry, clinical ROD diagnoses were consistent. Key variability sources were measurement methods, cortical bone evaluation, reference values, and mineralization disorder definitions.

*Table 1: Clinical diagnosis of ROD*

| **Lab/** Case | A | B | C | D | E |
|---|---|---|---|---|---|
| **1** | Hyperparathyroid bone disease | Normal, increased bone turnover | Hyperparathyroid bone disease | Low turnover osteoporosis | Low turnover osteoporosis |
| **2** | Normal, increased bone turnover | High bone turnover | Hyperparathyroid bone disease | Low turnover osteoporosis | Osteoporosis** |
| **3** | Mixed ROD | Hyperparathyroid bone disease | Hyperparathyroid bone disease/ Mixed ROD | Adynamic bone disease | Osteoporosis |
| **4** | Mild hyper-parathyroid bone disease | Normal bone | Osteoporosis, increased bone turnover | Low turnover osteoporosis | Osteoporosis/ Adynamic bone |
| **5** | High turnover | Normal bone | Osteoporosis, increased bone turnover | Low turnover osteoporosis | Low turnover osteoporosis |
| **6** | Mixed ROD | Normal, increased bone turnover | High bone turnover | Low bone turnover | Osteoporosis** |

ROD, renal osteodystrophy; **additional cortical turnover noted.

**P227**

**Hypophosphatemia as a determinant of Persistent Primary Hyperparathyroidism**

*Jayaditya Ghosh[1], Liza Das[2], Durairaj Arjunan[1], Poonam Kumari[1], Puneet Bhardwaj[1], Sanjay*

*K. Bhadada*[1]
*[1]Post Graduate Institute of Medical Education and Research, Endocrinology, Chandigarh, India*
*[2]Post Graduate Institute of Medical Education and Research, Endocrinology- Telemedicine, Chandigarh, India*

**Abstract Text**

**Background/Introduction**: Primary hyperparathyroidism (PHPT) is characterized by excessive secretion of parathyroid hormone (PTH), leading to hypercalcemia and hypophosphatemia. Hypophosphatemia exacerbates PTH's effects on bone and renal health, yet its role in the severity and management of PHPT is not well-defined. This study aimed to evaluate the impact of phosphate levels on clinical presentation, biochemical severity, and outcomes following parathyroidectomy in PHPT patients.

**Methodology** A retrospective analysis was conducted on 446 PHPT patients diagnosed over 10 years at a tertiary care hospital in North India. Patients were classified based on serum phosphate levels: normal phosphate (≥2.5 mg/dL) and hypophosphatemia (<2.5 mg/dL), further subdivided into mild (2–2.5 mg/dL) and moderate (1–2 mg/dL). Clinical symptoms, biochemical parameters, and post-surgical outcomes, including persistent disease (elevated serum calcium at 3 months), were analyzed.

**Results** Hypophosphatemia was present in 58.2% of patients, predominantly mild (69.7%). Patients with hypophosphatemia had more frequent bone pain (70.5% vs. 58.4%, p=0.026) and higher corrected calcium (12.1mg/dl(11.23-12.95) vs. 11.4mg/dl (11-12.5), p<0.001) and PTH levels (271pg/ml (162.5-664.5) vs. 204 pg/mL(121.55-534.42), p=0.013). Persistent disease was more common in the hypophosphatemia group (11.5% vs. 8.0%, p=0.037). Persistent hypophosphatemia at 3 months also associated with persistent disease. (23.4% vs. 12.93%, p=0.035).

**Conclusion** Low phosphate levels are associated with greater disease severity in PHPT, correlating with bone pain, elevated calcium and PTH, and higher rates of persistent disease. Incorporating phosphate levels into diagnostic and prognostic evaluations may improve disease management and outcomes.

**P228**

**Bone metabolism of heterotopic ossification is primarily influenced by time after trauma, independent of anatomic location**

*Simon von Kroge*[1,2], *Gesine Eis-Janzyk*[1], *Anke Baranowsky*[1], *Assil-Ramin Alimy*[1], *Frank-Timo Beil*[1], *Karl-Heinz Frosch*[1], *Konrad Mader*[1], *Johannes Keller*[1], *Tim Rolvien*[1]
*[1]University Medical Center Hamburg-Eppendorf, Department of Trauma and Orthopaedic Surgery, Hamburg, Germany*
*[2]University Medical Center Hamburg-Eppendorf, Department of Osteology and Biomechanics, Hamburg, Germany*

**Abstract Text**

**Background:** Heterotopic ossification (HO) is a common complication after trauma, such as fractures or surgical interventions. Although several risk factors and prophylactic measures have been established, HO growth and metabolic activity over time are largely unknown.

**Purpose:** This study aimed to identify specific characteristics of HO metabolism and time-dependent activity shared at two of the most prevalent sites of HO formation. Specifically, we addressed bone modeling and remodeling processes, microstructure, and mineralization in HO after elbow trauma and total hip arthroplasty.

**Methods:** We analyzed central and peripheral regions of 36 HO specimens (elbow/hip: 11/25) by performing cellular histomorphometry and quantitative backscattered electron imaging. Data were compared with iliac crest samples of healthy donors (n=11) and analyzed based on the time after trauma.

**Results:** Elbow and hip HO exhibited high proportions of woven bone (Fig. A,B), as well as endochondral ossification (C) and remodeling (D). Ongoing ossification was frequently observed along the HO rim, and a high osteoid volume (elbow: 6.67±4.54 vs. hip: 3.17±3.02 vs. control: 0.97±1.02, $p<0.001$), number of osteoblasts (18.8±11.4 vs. 18.0±12.9 vs. 7.5±4.9, $p=0.02$) and osteocytes (448±123 vs. 158±56 vs. 219±48, $p<0.001$) were evident, particularly in elbow HO (E). While elbow specimens were obtained at earlier stages after trauma, most characteristics were comparable to hip HO when matching for time after trauma (p-values in brackets). Ongoing ossification did not significantly change with time after trauma. However, the woven bone proportion and osteocyte density declined, particularly in the first three years. The mean calcium content increased inversely (F).

**Conclusion:** HO in both elbow and hip is characterized by immature, woven bone that is metabolically highly active, especially within the first three years after trauma. Although ongoing ossification was apparent several years after trauma, reduced metabolic activity and signs of bone maturation indicate that the risk of recurrence after resection might be reduced at later stages of HO development.

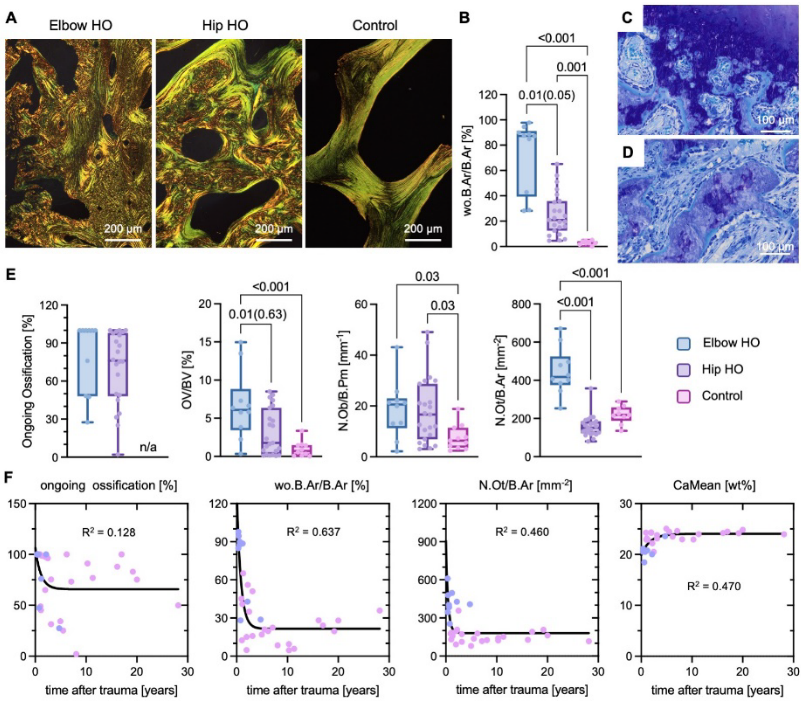

(A) Representative circularly polarized light microscopy images of picrosirius-red stained histological sections. (B) Elbow HO presented with higher proportions of woven bone (wo.B/B.Ar) than hip HO (time-matched p-value in brackets) and iliac crest controls, while the latter also presented with less woven bone than hip HO. (C) Zones of endochondral ossification were frequently apparent in toluidine-blue stained sections of elbow and hip HO. (D) Central regions were characterized by a high degree of remodeling. (E) Peripheral regions of HO showed large proportions of ongoing ossification, while central regions indicated high osteoid volume (OV/BV), a high number of osteoblasts per bone perimeter (N.Ob/B.Pm), and a high density of osteocytes (N.Ot/B.Ar). (F) Non-linear regression models showed only slight correlations of ongoing ossification with the time after trauma but strong correlations of woven bone percentage, osteocyte density, and the mean calcium content (CaMean).

**P231**

**Changes in bone microarchitecture 6 months after a first kidney transplant**

*<u>Cyrille Confavreux</u>[1], Charlène Levi[2], Angélique Denis[3], Aurélie Méry de Montigny[3], Pascal Roy[3], Christophe Mariat[4], Marie-Hélène Lafage-Proust[5], Tiphanie Ginhoux[6], Emmanuel Morelon[2], Aurélie Portefaix[6], Justine Bacchetta[7]*

*[1]INSERM U1033 LYOS -Université de Lyon - Hospices Civils de Lyon, Rheumatology, Pierre Bénite, France*

*[2]Hospices Civils de Lyon, Nephrology, Lyon, France*

*[3]Hospices Civils de Lyon, Statistics, Lyon, France*

*[4]CHU de Saint-Etienne, Nephrology, Saint-Etienne, France*

*[5]CHU de St Etienne, Rheumatology, St Etienne, France*

*[6]Hospices Civils de Lyon - INSERM CIC, EPICIME-CIC 1407 de Lyon- Service de Pharmacotoxicologie, Lyon, France*

*[7]Hospices Civils de Lyon - INSERM LYOS UMR 1033,*
*Centre de Référence des Maladies Rénales Rares- Centre de Référence des Maladies Rares du Calcium et du Phosphore- Filières Maladies Rares ORKiD- OSCAR et ERK-Net, Lyon, France*

**Abstract Text**

**Introduction**: Transplantation improves survival and quality of life compared with dialysis, but bone disorders frequently persist. Thus, even with cortisone-sparing strategies, transplant patients continue to have an increased risk of fracture. Our work aims at assessing early changes of bone microarchitecture after renal transplantation.

**Patients:** We conducted a prospective study in two French centers. Eligible patients were adults with severe renal failure treated by transplantation. Bone assessment was performed at 1 and 6 months post-transplant, using DXA and high-resolution peripheral quantitative CT (HR-pQCT XtremeCTScanco®) of the tibia and wrist.

**Results:** Between 2016 and 2019, 114 patients (56% males) were included split into 93 (82%) renal transplant alone and 21 (18%) kidney-pancreas. The mean (±SD) age was 50.6 ± 14.1 years. The three leading causes of renal failure were diabetes (35; 31%), glomerulopathy (26; 23%) and polycystic kidney disease (22; 19%). Most patients were on haemodialysis (85; 87%) or peritoneal dialysis (13; 13%) for (median [Q1 ;Q3]) 2.0 years [1.0 ; 3.6]. Sixteen (14%) patients were not on dialysis. No patient had suffered any fragility fracture or received bone treatment. There was no change in densitometry. Thirty-five patients (32%) were osteoporotic at inclusion.
At the tibia, 6 months after transplantation, there was a loss of BV/TV (12.0±3.9% vs 11.9±3.7; $p<0.05$), and a loss of volume density (vBMD) in cortical (857.9±61.3 mg/cm³ vs 853.5±61.9; $p<0.0001$) and trabecular bone (143.4±46.7 mg/cm³ vs 142.2±44.3; $p<0.0001$). Cortical thickness was also reduced (1.09±0.29 mm vs 1.07±0.27; $p<0.0001$) but not trabecular parameters. Bone loss was similar at radius. The older the patient, the greater the bone loss, but there was no difference between the sexes or the type of transplantation.

**Conclusion**: Our study shows an early impairment of bone microarchitecture after transplantation despite the improvement in renal function. It highlights the importance of bone management in renal transplant patients.

**P232**

**Iron pronounces heterotopic ossification**

*Sven Spangenberg[1], Lorenz C. Hofbauer[2], Martina Rauner[1], Ulrike Baschant[1]*
*[1]Medical Faculty- TU Dresden, Department of Medicine III and Center for Healthy Aging, Dresden, Germany*
*[2]Medical Faculty TU Dresden, Department of Medicine III and Center for Healthy Aging, Dresden, Germany*

**Abstract Text**

**Background:** Heterotopic ossification (HO) is bone formation at abnormal anatomical sites, developing a sequence of three the stages, inflammation, chondrogenesis and osteogenesis. Bone morphogenetic protein (BMP) signaling plays a critical role in the pathogenesis of HO and has also emerged as an essential regulator of iron homeostasis. We aimed to investigate how dysregulated iron metabolism impacts on HO development.

**Methods:** Wild type mice were fed a low (<10 ppm Fe), normal (200 ppm Fe) or high (20,000 ppm Fe) iron diet (HID) for 8 weeks starting from weaning on. HO was then induced by injection of recombinant BMP-2 into the M. tibialis anterior. Ectopic bone formation in the muscle was assessed at day 14 by µCT analysis. At day 3, muscle and HO tissue was analyzed by flow cytometry, proteome analysis and qPCR. Human samples were obtained from 18 patients, 9 from individuals with HO and 9 from patients who underwent hip replacement surgery, with the femoral head serving as a control.

**Results:** HID in WT mice led to a 2.6-fold increase in HO ($p<0.05$), while low iron diet led increased HO formation by 65% ($p<0.05$) compared to normal iron diet. Notably higher iron levels were measured in HO tissue of mice on HID, but also in patients with advanced HO +21%; +24%, $p<0.05$).

Analyzing the early HO stages revealed an increase in local oxidative stress as well as in inflammatory cell recruitment, especially of classical macrophages in mice on HID (2-fold, $p>0.01$). Along these lines, we observed a significant upregulation of pro-inflammatory cytokines and platelet-associated growth factors (e.g. PD-ECGF, 3.7-fold, $p>0.01$). Anti-inflammatory treatment with itaconate or dexamethasone prevented iron-induced aggravation of HO (-50% and -70%, $p<0.01$).

**Conclusions:** Our findings highlight the role of iron homeostasis in HO and, thereby, offer possibilities for therapeutic interventions using anti-inflammatory or iron-chelating agents.

**P233**

**The Impact of Immunosuppressive Strategies and Patient Characteristics on Change in Bone Mineral Density in Kidney Transplant Recipients: ALLEGRO trial**

*Annemieke Jansen[1], Renate de Jongh[1]*
*[1]Amsterdam UMC, Endocrinology, Amsterdam, Netherlands*

**Abstract Text**

**Background/introduction:** Immunosuppressive regimens impact bone mineral density (BMD) in kidney transplant recipients, contributing to increased fracture risk. Limited evidence exists on effects of immunosuppressive minimization strategies on BMD post-transplantation.

**Purpose:** To determine differences in post-transplantation BMD between immunosuppressive strategies and effect modification of age and sex thereon.

**Methods:** This is a post-hoc analysis of the ALLEGRO trial, a randomized, multicenter, open-label study involving de novo kidney transplant patients, comparing early steroid withdrawal (ESW) and tacrolimus minimization (TM) with standard immunosuppression (ST). The primary endpoint was 1-year post-transplantation change in lumbar spine (LS) and total hip (TH) BMD, measured by dual-energy X-ray absorptiometry. Interaction effects of sex and age were assessed.

**Results:** In 165 patients, 1-year post-transplantation LS BMD changed by +0.0085 g/cm² (+0.85%), -0.0084 g/cm² (-0.83%) and -0.0305 g/cm² (-3.10%) in ESW, TM and ST group, respectively ($p=0.004$ for ESW vs ST and $p=0.062$ for TM vs ST). Interaction analyses demonstrated a significant interaction for sex ($p=0.045$), but not for age ($p=0.214$). In men (N=105), changes were +0.0109 g/cm² (+1.06%), -

0.0329 g/cm² (-3.05%) and -0.0179 g/cm² (-1.77%) in the ESW, TM and ST groups, respectively (p=0.040 for ESW vs ST and p=0.807 for TM vs ST). In women (N=60), changes were +0.0027 g/cm² (+0.29%), +0.0203 g/cm² (+2.18%) and -0.0546 g/cm² (-5.82%) in ESW, TM and ST groups, respectively (p=0.063 for ESW vs ST and p=0.006 for TM vs ST). Changes in TH BMD did not differ between different treatment strategies.

**Conclusion:** This post-hoc, hypothesis-generating study suggests that ESW and TM affect LS BMD differently by sex, with both strategies more favorable than ST. Further research is needed to confirm the influence of sex and age on the effects of post-transplant immunosuppressive regimens on bone outcomes.

**P234**

### Chronic Nonbacterial Osteomyelitis (CNO) Presenting with Thoracic Outlet Obstruction and Upper Arm DVT

*Nina Thomson[1], Jack Leese[1], Barbara Hauser[1]*
*[1]NHS Lothian, Rheumatology Department, Edinburgh, United Kingdom*

### Abstract Text

Chronic Nonbacterial Osteomyelitis (CNO) is a rare autoinflammatory bone disorder characterized by sterile bone inflammation, leading to pain, swelling, and potential bone expansion and deformities 1. Often misdiagnosed, the average diagnostic delay is five years, contributing to disease progression and complications2. Expert consensus guidelines1 provide a framework for diagnosis and management, but research is needed to enhance early detection and prevent bone deformities.

This report describes a 40-year-old male with a decade-long history of palmoplantar pustulosis, sternoclavicular discomfort, and back stiffness. Despite the combination of dermatological and musculoskeletal symptoms, the condition was undiagnosed and inadequately managed with occasional NSAIDs and topical treatments. Progressive disease led to clavicular cortical expansion, subclavian vein compression, and upper arm deep vein thrombosis (DVT). CT imaging confirmed clavicular sclerosis and cortical thickening consistent with CNO. Persistent inflammation (CRP: 30 mg/L) supported the diagnosis. Earlier recognition and treatment could possibly have prevented complications, including thoracic outlet obstruction and thrombotic events.

This case underscores the severe consequences of delayed diagnosis and insufficient treatment in CNO, such as vascular compression, bone overgrowth, and DVT. It highlights the need for increased clinical awareness of CNO to improve early detection and outcomes. Further research should explore whether bisphosphonates (BP) or tumor necrosis factor (TNF) inhibitors can prevent pathological bone expansion in CNO.

References:

1.Winter EM, Dekkers OM, Andreasen CM, et al. Expert consensus recommendations for the diagnosis and treatment of chronic non-bacterial osteitis (CNO) in adults. *Ann Rheum Dis*. Published online November 28, 2024.

2. Leerling AT, Dekkers OM, Appelman-Dijkstra NM, Winter EM. Clinical and therapeutic diversity in adult chronic nonbacterial osteomyelitis (CNO) of the sternocostoclavicular region: a meta-analysis. *Rheumatology (Oxford)*. 2023;62(2):512-522

**P235**

**Citrate reduces Calcium Protein Particle toxicity and prevents vascular calcification**

*Guido De La Roij[1], Jeroen de Baaij[1], Joost Hoenderop[1]*
*[1]Radboudumc, Medical Biosciences, Nijmegen, Netherlands*

**Abstract Text**

**Background/Introduction:** Chronic Kidney Disease is a major risk factor for cardiovascular diseases, partly due to arterial stiffening caused by calcium phosphate deposition. This results from an imbalance between phosphate and calcium, leading to the formation of secondary calcium protein particles (CPP, see figure), which are closely associated with vascular calcification (VC). Preventing VC is critical as treatment becomes challenging once established; therefore, we target CPPs during the early stages of kidney failure.

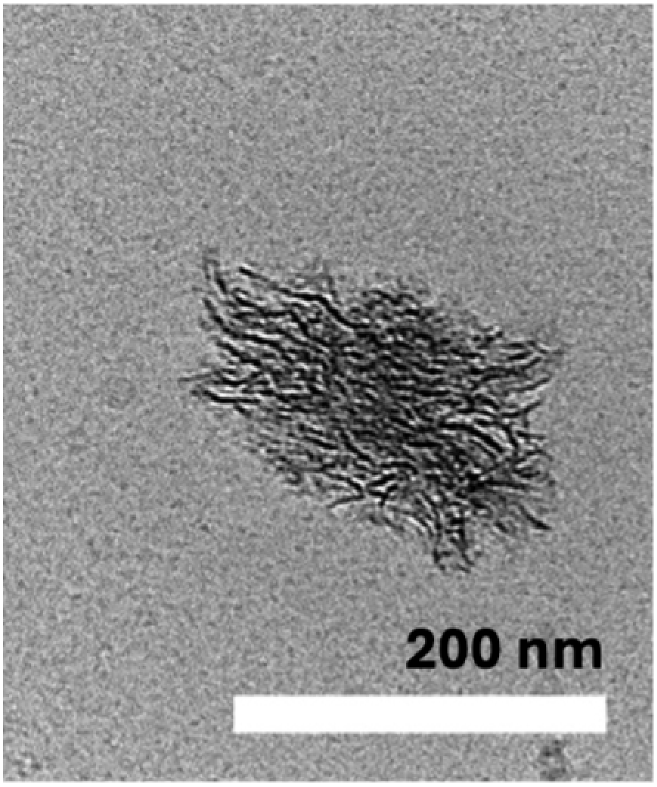

**Purpose:** We aim to evaluate the potential of citrate supplementation to prevent in-vitro vascular calcification by modulating CPPs.

**Methods:** Calcium protein assays were conducted to quantify calcification. Electron Microscopy, Raman spectroscopy, Dynamic Light Scattering, Electron Diffraction, and Mass Spectrometry were employed to characterize the particles' morphology, mineral composition, and protein content.

**Results:** $CPP_{1000\ \mu M\ citrate}$ led to a significant reduction of VC by 0.88 (SD ± 0.057, $p < 0.0001$) compared to $CPP_{STD}$. CryoTEM showed that the particles were morphologically similar. DLS showed smaller diameters for $CPP_{1000\ \mu M\ citrate}$ particles (216.1 nm; range 214.4–219.1 nm) versus $CPP_{STD}$ (236.2 nm; range 228.1–242.5 nm) with a p-value of 0.0022. ED showed that the crystal remained the same for the different conditions. Raman analysis showed a higher mineral protein ratio for $CPP_{1000\ \mu M\ citrate}$ which was confirmed by macro analysis (p = 0.0028). MS revealed 18 proteins uniquely present in $CPP_{STD}$ such as APOA1, APOA2, and APOE.

**Conclusion(s)** Citrate effectively modulates CPPs by lowering their calcification toxicity, slowing their maturation, decreasing the diameter, and increasing the mineral to protein ratio. Despite morphological similarities, citrate-treated particles exhibit significant differences in protein composition, which may underlie their reduced toxicity. These findings highlight the potential of citrate as a therapeutic strategy for preventing VC.

**ND-P020**

**Fracture Risk and Complications in Patients with Combined Primary Hyperparathyroidism and Type 2 Diabetes: A Nationwide Cohort Study**

*Emma Bakhauge[1], Stinne Dueholm[1], Vitus Bollerslev[1], Annika Vestergaard Kvist[1],*
*Peter Vestergaard[1], Nicklas Rasmussen[2]*
*[1]Aalborg University Hospital- Denmark, Steno Diabetes Center North Denmark, Aalborg, Denmark*
*[2]Aalborg University Hospital,*
*Steno Diabetes Center North Denmark and the Danish Diabetes and Endocrine Academy, Aalborg, Denmark*

**Abstract Text**

**Background & Objective:** The coexistence of type 2 diabetes (T2DM) and primary hyperparathyroidism (PHPT) may lead to a compounded disease burden. This study aimed to assess the risk of fractures, mortality, major adverse cardiovascular events (MACE), renal manifestations, nephrolithiasis, depression, and parathyroidectomy (PTX) in individuals diagnosed with both conditions.

**Methods:** A retrospective cohort study was conducted using data from the Danish National Patient Registry between 1996 and 2020. Individuals aged 50–90 years were included and categorized into four groups: controls(n=7,317), T2DM(n=6,721), PHPT(n=1,945), and T2DM+PHPT(n=2,105). Participants were followed from diagnosis or prescription of relevant medications until the study endpoint. The primary outcome was the incidence of fractures, while secondary outcomes included all-cause mortality, MACE, renal manifestations, nephrolithiasis, depression, and PTX. Cox proportional hazards models and competing risk regression models were applied to estimate hazard ratios (HRs) and subhazard ratios adjusting for age, sex, and Charlson Comorbidity Index.

**Results:** Fracture risk was lower in the T2DM (HR 0.82, 95% CI 0.76–0.89, $p<0.001$) and T2DM+PHPT groups (HR 0.73, 95% CI 0.64–0.82, $p<0.001$) compared to controls. The T2DM+PHPT group exhibited the highest mortality risk (HR 1.20, 95% CI 1.10–1.32, $p<0.001$). MACE risk was significantly increased in T2DM (HR 1.12, 95% CI 1.02–1.22, $p=0.02$) but not in T2DM+PHPT. Renal complications were significantly elevated in PHPT (HR 2.50, 95% CI 2.05–3.05, $p<0.001$) and T2DM+PHPT (HR 2.20, 95% CI 1.82–2.66, $p<0.001$). Depression risk was increased in PHPT (HR 1.33, 95% CI 1.04–1.70, $p=0.02$) and non-significantly in T2DM+PHPT (HR 1.22, 95% CI 0.94–1.59, $p=0.14$). Parathyroidectomy rates were nearly doubled in the T2DM+PHPT group(HR 2.75, 95% CI 2.36–3.20, $p<0.001$) compared to PHPT alone.

**Conclusion:** No synergistic effect on fracture risk was observed in individuals with both T2DM and PHPT, but the combination was associated with significantly increased mortality. Additionally, risks of cardiovascular, renal, psychiatric complications, and parathyroidectomy were elevated, underscoring the need for targeted clinical management.

**ND-P021**

**Not just another adenoma: the diagnostic challenges of atypical parathyroid lesions**

*Alexandru Florescu*[1], *Teodora-Cristina Vintilă*[2], *Viviana Adam*[3], *Andra Berigoi*[2], *Irina Pușcașu*[2]
[1]*Saint Spiridon County Hospital, Endocrinology, Iasi, Romania*
[2]*Saint Spiridon County Hospital, Endocrinology, Iași, Romania*
[3]*Grigore T Popa University of Medicine and Pharmacy, Endocrinology, Iasi, Romania*

**Abstract Text**

Atypical parathyroid adenomas are rare tumors that pose diagnostic challenges due to their overlapping histopathological features with benign parathyroid adenomas and parathyroid carcinoma. Clinically, these tumors cause symptoms of primary hyperparathyroidism and hypercalcemia. Although imaging techniques can detect them, a definitive diagnosis is established through histopathological assessment.

We report the case of a 74 year old male, with a history of prostate cancer, whose routine CT scan incidentally identified a lesion posterior to the left thyroid lobe alongside multiple pelvic osteolytic bone metastases. Of note, the patient was completely asymptomatic. Endocrinological tests showed normal thyroid function, hypercalcemia(Ca=13,3 mg/dl), hyperparathyroidism(1741 pg/ml), low-normal vitamin D (20,59 ng/ml) and hypercalciuria (367,2 mg/24h). Neck ultrasound revealed a well defined, hypoechoic, highly vascularized mass(3,22x4,9x2,86cm) behind the left thyroid lobe, raising suspicion of a parathyroid lesion. BMD-DXA revealed severe osteoporosis in the distal forearm( T score= 5,9) and decreased bone mineral density in the lumbar spine and femoral neck.Despite hydratation, hypercalcemia persisted, prompting treatment with intravenous ibandronic acid . Sestamibi scintigraphy confirmed a large hyperfunctioning parathyroid lesion on the left side, along with a suspicious thyroid nodule. Additionally, a bone scan identified suspicious bone lesions in the skull and skeleton. One month later, the patient underwent parathyroidectomy and thyroidectomy, and the histopathological report confirmed an atypical parathyroid adenoma. A bone biopsy was recommended, but has not yet been performed.

This case highlights the diagnostic challenge of hypercalcemia in a patient with a history of malignancy. Although the presence of osteolytic bone lesions initially suggested metastatic prostate cancer, distinguishing between malignancy and parathyroid bone disease proved more difficult than expected. This distinction is crucial, since parathyroid bone disease may improve postoperatively, while metastatic disease would persist or progress. Close follow-up and monitoring are essential to reach a correct diagnosis and ensure patient well-being in the postoperative period.

**ND-P023**

**Stac3 Deficiency, Lipid Overload, and Reduced BMD: A Zebrafish Model for Native American Myopathy.**

*Lital Ashtamkar Matok*[1]*, Rajashekar Donaka*[2]*, David Karasik*[1]
[1]*Bar-Ilan University, The Azrieli Faculty of Medicine, Zefat, Israel*
[2]*Program for Musculoskeletal Research- University of Colorado- Aurora, Department of Orthopedics, Colorado, United States*

**Abstract Text**

**Background:** The STAC3 gene is implicated in muscle weakness and elevated lipid levels in Native American Myopathy (NAM) patients. Leveraging the evolutionary conservation of STAC3, we used zebrafish as a valuable in vivo model for studying rare genetic diseases. Of late, we showed that stac3-/- zebrafish model faithfully reflects human phenotypes of NAM, including increased neutral lipids, structural and functional defects in skeletal muscle, and early mortality. This effective research model allows us to further investigate the consequences of STAC3 loss-of-function on bone mineral density (BMD). We aimed to explore the complex effect of STAC3 on muscle function, lipid metabolism, and adult BMD.

**Methods:** stac3 knockout (KO) and heterozygote lines were established using CRISPR-Cas9 technology and propagated for three generations. Standard length, weight and body mass index measurements were recorded. At 11 months post-fertilization (mpf), zebrafish were fixed in 3.7% paraformaldehyde overnight at room temperature and subsequently scanned using micro-computed tomography (micro-CT) (SKYSCAN 1172; Bruker, Kontich, Belgium). BMD was calculated using standard methods.

**Results:** Since homozygotes for stac3-/- die as larvae, heterozygous (stac3+/-) siblings were utilized for BMD and body composition analyses. No significant differences were observed in either total body weight or body length between stac3+/- and wild-type (+/+) siblings at 11 mpf. However, BMD analysis revealed a significantly lower BMD of stac3+/- heterozygotes compared to their stac3+/+ counterparts ($p = 0.00005$), while bone and whole-body morphology seems unaffected.

In conclusion, while the precise mechanisms connecting low BMD, elevated lipid levels and skeletal morphology in NAM patients remain to be fully elucidated, and several potential pathways are under consideration, our zebrafish model suggests a link between reduced BMD and the partial loss of STAC3 gene. Further research is warranted to validate these findings and explore the specific molecular mechanisms involved.

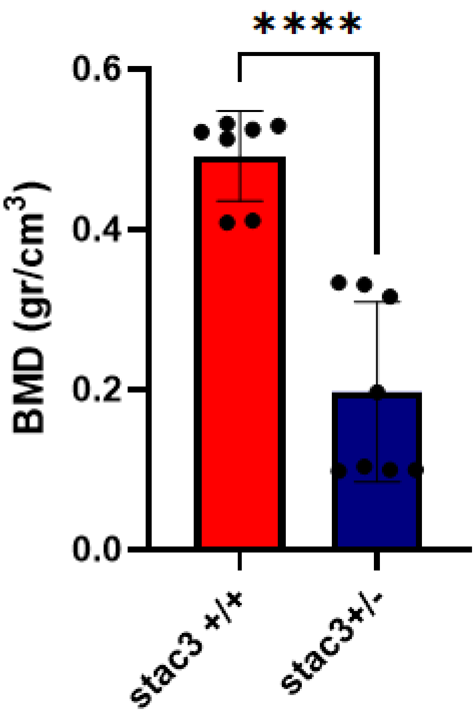

**ND-P024**

**Observational Cohort Study of Fracture Rates and Fracture Related Outcomes in Dialysis Patients.**

*Nadim Beruni*[1], *Grahame Elder*[2]
[1]*Westmead Hospital, Renal Medicine Department, Westmead, Australia*
[2]*Westmead Hospital, Renal Medicine, Westmead, Australia*

**Abstract Text**

**Background:** Chronic kidney disease (CKD) is associated with an elevated risk of fractures, particularly in patients undergoing haemodialysis, due to complex disturbances of bone and mineral metabolism.

**Purpose:** This observational cohort study aimed to establish baseline fracture rates and outcomes among haemodialysis patients within the Western Sydney Local Health District (WSLHD).

**Methods:** Data were collected from January 2022 to July 2024 for patients on home or satellite haemodialysis from WSLHD electronic medical records. Data extracted included patient demographics, baseline biochemistry, dialysis characteristics, and patient relevant outcomes including fracture characteristics and fracture related outcomes. Data were analyzed using IBM SPSS (Version 29.0).

**Results:** Of 549 patients, 153 (27.9%) experienced fractures, equivalent to 56.1 fractures per 1000 patient-years. Hip and long bone fractures were the most common, and 77% of fracture events required hospitalization. Patients with fractures suffered higher mortality (34% vs. 22.5%, p=0.006), had higher rates of dialysis withdrawal (10.5% vs. 4.6%, p=0.01) and were more likely to be discharged to residential aged care facilities (OR 17.34, p<0.01). Despite the high fracture burden, bone mineral density (BMD) assessment and management with antiresorptive, anabolic and hormonal therapies used for osteoporosis did not differ between those patients who did or did not fracture.

**Conclusions:** Patients on haemodialysis have high rates of fracture and greater rates of hospitalization and mortality. Following a fracture these patients do not receive targeted investigations or management that might assist in reducing fracture risk. Consideration should be given to the development of fracture prevention strategies and the instigation of multidisciplinary Fracture Liaison Services for patients with CKD on dialysis.

**ND-P025**

**Radiologic prevalence of Paget's disease of bone in Campania, Southern Italy**

*Veronica Abate[1], Anita Vergatti[1], Luigi Gennari[2], Daniela Merlotti[2], Guido Cavati[3], Filippo Pirrotta[3], Gianpaolo De Filippo[4], Giuseppina Annunziata[5], Luisa Mangoni di Santo Stefano[5], Domenico Rendina[1]*
*[1]Federico II University, Department of Clinical Medicine and Surgery, Naples, Italy*
*[2]Azienda Ospedaliera Universitaria Senese, Department of medical sciences, Siena, Italy*
*[3]Azienda Ospedaliera Universitaria Senese, Department of Medical Science, Siena, Italy*
*[4]Hôpital Robert Debré, Assistance Publique-Hôpitaux de Paris-, Paris, France*
*[5]Azienda Ospedaliera Santa Maria della Pietà, Department of Radiology, Naples, Italy*

**Abstract Text**

**Background:** Paget's disease of bone (PDB) is a focal disorder of bone turnover, characterized by pathognomonic radiological deformities, affecting one (monostotic PDB) or more skeletal sites (polyostotic PDB). Previous studies demonstrate that patients from Campania, Southern Italy, showed a higher PDB clinical severity. Since the PDB prevalence in Campania is unknown, we performed a radiographic survey in three Campania Hospitals.

**Methods:** All X-ray exams showing the entire pelvis, the sacrum, the lumbar vertebrae, and the femoral heads were reviewed for the PDB presence. The exams were collected in three Campania hospitals, A.O.R.N. Moscati, Avellino, A.O.R. N. Rummo, Benevento, and A.O. Santa Maria della Pietà, Nola, Naples, from 01/01/2024 to 12/31/2024.

**Results:** Overall, we analysed 2347 X-ray exams of as many patients aged > 40 years [males 1063; mean age 66.7±9.9 years]. Of them, 24 [males 12; 50.0%, mean age 68.1±11.6 years] were affected by PDB located in the pelvis. The crude radiographic prevalence of pelvic PDB was 1.00%. Based on reported prevalence of pelvic PDB in patients from Campania (68%), the estimated overall prevalence of PDB in Campania was 1.47%.

**Conclusion:** Based on crude radiological prevalence of pelvic PDB in patients from Campania (1.00%), the estimate prevalence of PDB in this geographical area characterized by increased PDB clinical severity is 1.47%.

**Poster Sessions: Paediatric bone diseases**

**P236**

**Epidemiology of Brachydactyly Type A3 in China: A Nationwide Multi-center Population-based Study Among Children Aged 3 to 17 Years**

*Wenshuang Zhang[1,2], Geng Jian[2], Hu Bo[2], Yuan Yi[2], Liu Yandong[2], Wang Ling[2], Cheng Xiaoguang[2]*
*[1]Peking University Fourth School of Clinical Medicine, Department of Radiology, Beijing, China*
*[2]Beijing Jishuitan Hospital- Capital Medical University, Departmrnt of Radiology, Beijing, China*

**Abstract Text**

**Background:** Brachydactyly Type A3 (BDA3) is a congenital condition characterized by shortening of the middle phalanx of the fifth digit. While understanding its epidemiology is important for identifying genetic and environmental risk factors, large-scale studies on BDA3 in populations like China are limited.

**Objective:** This study aimed to provide nationwide epidemiological data on the prevalence of BDA3 and its associated epiphyseal abnormalities among Chinese children aged 3 to 17 years, with a focus on gender, regional, and urban-rural differences.

**Methods:** The study sample was drawn from the "Large-sample Investigation on Skeletal Maturation in 3 to 18-Year-Old Children in China" (ClinicalTrials.gov ID: NCT04197557), a subset of the "National Nutrition and Health Systematic Survey for Children Aged 0-18" (ChiCTR1900022825). Using multi-stage stratified random sampling, 17850 children (8856 boys and 8994 girls) from 14 provinces were selected. Non-dominant hand-wrist radiographs were obtained using a portable X-ray device. A retrospective analysis identified BDA3 and associated epiphyseal abnormalities, with prevalence rates calculated and compared across demographics.

**Results:** Of the 17850 radiographs analyzed, the overall prevalence of BDA3 was 10.0%, with a higher prevalence in girls (12.9% vs 7.1%, P<0.001). The northern region had a higher prevalence than the southern region (11.5% vs.8.6%, P<0.001), while no significant difference was found between urban and rural areas (10.3% vs. 9.8%, P=0.32). Two BDA3 phenotypes were identified: brachymesophalangia-5 with cone-epiphysis of mid-5 (BMP5 with CM5) (3.6%) and brachymesophalangia-5 alone (BMP5 alone)(6.4%). Both phenotypes showed a higher prevalence in girls(BMP5 with CM5: 4.4% vs.2.8%, BMP5 alone: 8.6% vs.4.3%; P<0.001 for both). Regional and urban-rural differences in phenotype prevalence were also noted.

**Conclusions:** This nationwide study is the first to provide comprehensive epidemiological data on the prevalence of BDA3 and related epiphyseal abnormalities in Chinese children. Significant gender and regional differences in prevalence highlight the need for further studies into the genetic and environmental factors contributing to BDA3.

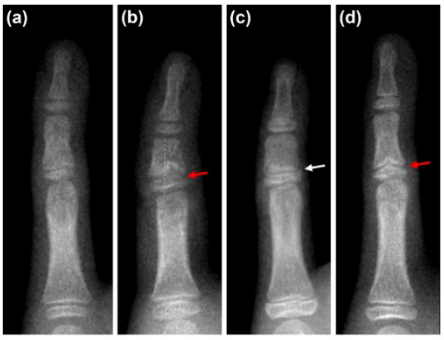

**Figure 1**. Diagrams of normal, BDA3 (BMP5 with CM5, BMP5 alone), and CM5 alone, and measurement of the length of the middle phalanx V. (a) Normal middle phalanx V. (b) BMP5 with CM5: shortened middle phalanx V with a conical epiphysis (red arrow). The white dashed line indicates the measurement of the middle phalanx length. (c) BMP5 alone: isolated shortening of the middle phalanx with a normal epiphysis (white arrow). (d) CM5 alone: conical epiphysis of the middle phalanx V (red arrow) without shortening. BMP5 with CM5, brachymesophalangia-5 with cone-epiphysis of mid-5; BMP5 alone, brachymesophalangia-5 alone; CM5 alone, cone-epiphysis of mid-5 alone; phalanx V, phalanx of the fifth finger.

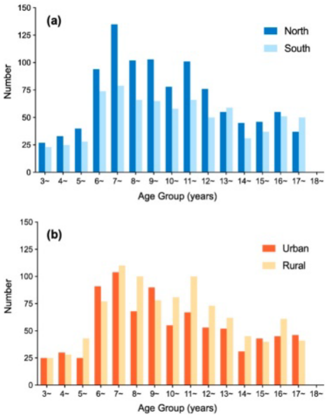

**Figure 2**. Number of BDA3 across different age groups in northern and southern regions, urban and rural areas. BDA3, brachydactyly type A3.

**P237**

**Sanjad Sakati syndrome and craniosynostosis: a case series**

*Noor Bawahab*[1], *Abdulmajeed Alsubaihin*[1]

[1]*King Saud University Medical City, Pediatric Endocrine, Riyadh, Saudi Arabia*

**Abstract Text**

Sanjad Sakati syndrome (SSS) is a rare syndrome first described in Saudi Arabia in the year of 1988. The features that are reported in this syndrome include congenital hypoparathyroidism, intrauterine growth restriction ( IUGR ) with poor growth later on. Moreover, developmental delay, learning disabilities and characteristic facial features.

Upon our observation we noticed that four cases( Table.1 ) were found to have an additional skeletal feature, manifested as craniosynostosis. All the patients have disclosed dysmorphic features in the form of low set ears, triangular face, hypertelorism,depressed nasal bridge, micrognathia, abnormal head shape ( Scaphocephaly ) , poor dentition, small hands and feet.

Moreover, it was observed that they have poor growth parameter including height, weight and head circumference all below the third centile.

Herein, we report those cases, as far as we know our report is the first one that recognized the association of craniosynostosis with Sanjad Sakati syndrome. It is necessary to identify and prevent further complications such as neuro-ophthalmological and neuro-cognitive defects consequently.

We believe that as many more reports on this rare syndrome are being published, we will get more knowledge of the associations related to it.

| Case No. | Age | Gender | CT brain |
|---|---|---|---|
| 1 | 8 year | Female | Sagittal craniosynostosis, Bilateral cerebral and cerebellar calcifications |
| 2 | 4 year | Female | Premature closure of the sagittal suture. Supratentorial ventriculomegaly. Crowding of the foramen magnum with kinking and compression upon the cervicomedullary junction. |
| 3 | 3 year | Male | Premature closure of the sagittal suture. |
| 4 | 9 year | Female | Bilateral subcortical and basal ganglia symmetric calcifications.Evidence of premature closure of sagittal suture. |

Table 1. Demographic and CT brain finding.

**P238**

**Lower Limb Deformities in Rickets: A Systematic Review**

*Anahita Mayr[1,2], Gabriel T. Mindler[3,4], Adalbert Raimann[1,2]*
[1]*Medical University of Vienna, Department of Pediatrics and Adolescent Medicine, Vienna, Austria*
[2]*Vienna Bone and Growth Center, Department of Pediatrics, Vienna, Austria*
[3]*Orthopedic Hospital Speising, Department of Pediatric Orthopaedics, Vienna, Austria*
[4]*Vienna Bone and Growth Center, Department of Orthopedics, Vienna, Austria*

**Abstract Text**

**Background**: Lower limb deformities significantly impact patients with rickets, yet structured data on this symptom in hypophosphatemic (HPR) and calcipenic rickets (CR) remains limited.

**Purpose:** This review aims to present available data and identify literature gaps regarding lower limb deformities in HPR (e.g., XLH) and CR patients.
Methods: A PubMed search using "rickets" AND "deformity" was conducted through 08/2023.

**Results**: Analysis of 126 studies included 2924 patients with rickets-associated leg deformities. Varus deformities were equally prevalent in HPR and CR, while valgus and windswept deformities were more common in CR. Pathologic femoral torsion (n=70), procurvatum (n=85), and recurvatum (n=6) were exclusively reported in HPR. Sagittal tibial deformities were more prevalent in HPR (7 studies). Only 54.8% of studies reported basic laboratory assessments (total alkaline phosphatase, serum phosphate and serum calcium), 17.5% assessed ≥1 radiographic/functional parameter for deformity quantification. In total 5.6% (7/126) included both minimal radiological and laboratory assessments, all in HPR patients with no case series including CR fullfilling these criteria.

**Conclusion(s):** Current data on acquired and inherited forms are limited and heterogeneous, often lacking minimal standards for interpretation. Only 5.6% of studies document lower limb deformities using standardized basic radiological and laboratory parameters, with a strong focus on HPR. Future efforts for prevention and optimal treatment of deformities associated with both acquired and inherited types of rickets would benefit from minimal quality criteria for deformity assessment. Multidisciplinary research approaches are needed to address the complex nature of these conditions.

**ND-P026**

**Sanjad Sakati syndrome and craniosynostosis: a case series**

*Noor Bawahab[1], Abdulmajeed Alsubaihin[1]*
*[1]King Saud University Medical City, Pediatric Endocrine, Riyadh, Saudi Arabia*

**Abstract Text**

Sanjad Sakati Syndrome (SSS) is a rare genetic disorder first described in Saudi Arabia in 1988. It is caused by mutations in the TBCE (Tubulin Folding Cofactor E) gene, which plays a crucial role in microtubule assembly, cytoskeletal organization, and skeletal development. Clinically, SSS is characterized by congenital hypoparathyroidism, intrauterine growth restriction (IUGR), and postnatal growth failure. Additionally, patients commonly exhibit developmental delay, learning disabilities, and distinct facial dysmorphism.

In our study, we observed that four cases presented with an additional skeletal abnormality—craniosynostosis. Given that TBCE is essential for osteoblast function and bone remodeling, its mutation may contribute to abnormal skull formation and premature suture fusion. To the best of our knowledge, this is the first report recognizing an association between craniosynostosis and Sanjad Sakati Syndrome. Early identification of this feature is essential to prevent potential complications, including neuro-ophthalmological and neurocognitive impairments.

As more cases of this rare syndrome are documented, our understanding of its clinical spectrum and associated features will continue to expand, aiding in improved diagnosis and management.

| Case no. | Age | Gender | Average Calcium mmol/L | Average phosphate mmol/L | Average ALP Unit/L | Average PTH pmol/L | CT brain |
|---|---|---|---|---|---|---|---|
| 1 | 8 years | Female | 2.065 | 1.66 | 135.21 | 0.283 | Sagittal craniosynostosis, Bilateral cerebral and cerebellar calcifications |

| | | | | | | | |
|---|---|---|---|---|---|---|---|
| 2 | 4 years | Female | 1.91 | 2.35 | 227 | 0.15 | Premature closure of the sagittal suture. |
| 3 | 3 years | Male | 2.29 | 3.21 | 231.8 | 0.36 | Premature closure of the sagittal suture |
| 4 | 9 years | Female | 1.99 | 3.45 | 204.55 | 0.18 | Premature closure of the sagittal suture |

**Poster Sessions: Rare bone diseases**

**P239**

**Safety and efficacy of garetosmab in patients with fibrodysplasia ossificans progressiva: Design of the phase 3, randomised, placebo-controlled OPTIMA trial**

*Stephen Dimartino[1], Susan Rhee[2], Robert Pordy[3], Meijian Zhou[4], Stefanie Hectors[5], Robert J. Sanchez[6], Richard DelGizzi[7], Dinko Gonzalez Trotter[5], Richard Keen[8]*
*[1]Regeneron Pharmaceuticals- Inc., General Medicine, Tarrytown, United States*
*[2]Regeneron Pharmaceuticals- Inc., Clinical Sciences – Internal Medicine, Tarrytown, United States*
*[3]Regeneron Pharmaceuticals- Inc., Global Development, Tarrytown, United States*
*[4]Regeneron Pharmaceuticals- Inc., Biostatistics, Tarrytown, United States*
*[5]Regeneron Pharmaceuticals- Inc., Precision Medicine and Imaging, Tarrytown, United States*
*[6]Regeneron Pharmaceuticals- Inc., Health Economics & Outcomes Research, Tarrytown, United States*
*[7]Regeneron Pharmaceuticals- Inc., Global Patient Safety, Tarrytown, United States*
*[8]Centre for Metabolic Bone Disease Royal National Orthopaedic Hospital NHS Trust, Rheumatology, London, United Kingdom*

**Abstract Text**

**Introduction.** Fibrodysplasia ossificans progressiva (FOP), an ultra-rare, severely disabling disease, is caused by heterozygous missense mutations in the gene encoding activin A receptor type 1 (*ACVR1*). The disease is characterised by progressive multi-focal heterotopic ossification (HO) of extra skeletal tissue. We describe the design of the ongoing phase 3 OPTIMA clinical trial (NCT05394116) evaluating the safety and efficacy of low-dose or high-dose garetosmab, a fully human activin A monoclonal antibody, in patients with FOP.

**Methods.** In this phase 3, multinational, randomised, three-arm, parallel-group, placebo-controlled trial, the targeted enrolment is 66 patients with active FOP. The primary endpoint is the number of new HO lesions and will be assessed by CT after the 56-week double-blind treatment period. The study design is illustrated in the **Figure**.

**Results.** Key primary and secondary endpoints of OPTIMA are summarised (**Table**).

**Conclusion**. This study will assess the safety and efficacy of garetosmab in patients with active FOP.

**Figure. Study flow**

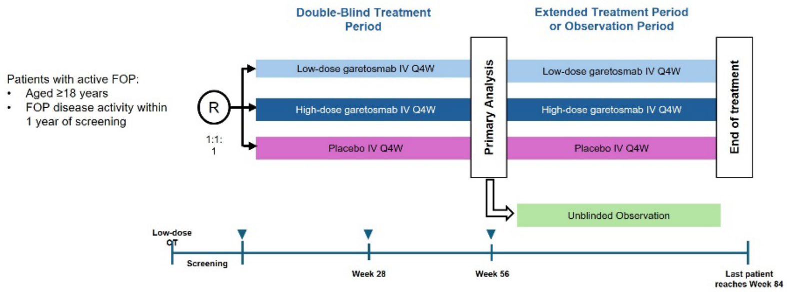

At the end of the double-blind treatment period, patients can choose to either extend the double-blind treatment until the last global patient completes Week 84 or discontinue double-blind treatment and enter the observation period.
*ACVR1*, gene encoding activin A receptor, type I; FOP, fibrodysplasia ossificans progressiva; IV, intravenous; R, randomization; Q4W, every 4 weeks,

**Table. Key primary and secondary endpoints**

| |
|---|
| **Primary efficacy endpoint**<br>• Number of new HO lesions at W56 |
| **Primary safety endpoint**<br>• Incidence and severity of treatment-emergent adverse events of special interest from baseline to W56 |
| **Secondary efficacy endpoints**<br>***Assessed at W56:***<br>• Occurrence of new HO lesions (Yes/No)<br>• Total volume of new HO lesions<br>***Assessed through W56:***<br>• Number of clinician-assessed flare-ups<br>• Occurrence of clinician-assessed flare-ups (Yes/No)<br>• Number of patient-reported flare-ups<br>• Occurrence of patient-reported flare-ups (Yes/No)<br>***Change from baseline to W56:***<br>• Joint function assessment by physician using Cumulative Analogue Joint Involvement Scale<br>• Pulmonary function as assessed by spirometry<br>• Disease severity as assessed by the Patient Global Impression of Severity, the Patient's Global Impression of Change, and the Clinician's Global Impression of Change |

W, week.

### P240

### Potential predictors of clinical and biochemical response to burosumab treatment in adult XLH patients

*Gaetano Paride Arcidiacono*[1], *Marco Onofrio Torres*[1], *Valentina Camozzi*[2], *Cristina Eller-Vainicher*[3], *Giuseppe Vezzoli*[4], *Maria Luisa Brandi*[5], *Gemma Marcucci*[6], *Antonio Aversa*[7], *Gaetana Cerbone*[8], *Maria Michela D'Alessandro*[9], *Giuseppe Girasole*[10], *Corrado Vitale*[11], *Paolo Simioni*[1], *Stefania Sella*[1], *Sandro Giannini*[1]

[1]*University of Padua, Department of Medicine - Clinica Medica 1, Padua, Italy*
[2]*Azienda Ospedale-Università di Padova, UOC Endocrinologia, Padua, Italy*
[3]*Fondazione Ca' Granda IRCCS Ospedale Maggiore Policlinico, UOC Endocrinologia, Milano, Italy*
[4]*IRCCS- Ospedale San Raffaele e Università Vita Salute San Raffaele, Unità di Nefrologia e Dialisi, Milano, Italy*
[5]*Fondazione, Firmo, Firenze, Italy*
[6]*AOU Careggi di Firenze, SOD Malattie del Metabolismo Minerale e Osseo, Firenze, Italy*
[7]*Università Magna Graecia di Catanzaro, Dipartimento di Medicina Sperimentale e Clinica, Catanzaro, Italy*
[8]*AORN San Giuseppe Moscati, UOS Genetica Medica, Avellino, Italy*
[9]*ARNAS Civico, UO Nefrologia Pediatrica, Palermo, Italy*
[10]*Azienda Sanitaria Locale 3 - Ospedale La Colletta, SC Reumatologia, Arenzano, Italy*
[11]*AO Ordine Mauriziano, SC Nefrologia e Dialisi, Torino, Italy*

### Abstract Text

**Background:** X-linked hypophosphatemia (XLH) is a genetic disorder characterized by elevated levels of fibroblast growth factor 23 (FGF23), leading to chronic renal phosphate wasting and hypophosphatemia, causing skeletal and extraskeletal abnormalities. Burosumab, a human monoclonal antibody inhibiting FGF23, effectively corrects hypophosphatemia and improves the disease's clinical course. This study assessed burosumab's efficacy in a real-world setting and identified potential predictors of treatment response.

**Methods:** Twenty-seven adult XLH patients from an Italian multicenter cohort were treated with burosumab for up to 24 weeks; laboratory tests were evaluated at midpoints and endpoints (i.e., 14 and 28 days) of the dosing interval. Of these, eleven patients were followed up for 48 weeks, including both laboratory assessments and patient-reported outcomes.

**Results:** We enrolled 27 XLH patients (mean age 42 years; 48% female). After the initiation of burosumab, median serum phosphate levels increased from a baseline of 1.5 mg/dL (IQR 1.3–1.8) to 2.0 mg/dL (IQR 1.7–2.4) ($p<0.05$) and remained higher than baseline at the midpoints of the dosing interval for up to 24 weeks of observation. Higher baseline serum phosphate levels predicted higher midpoint levels ($p<0.05$), whereas higher baseline PTH ($p<0.05$) and FGF23 ($p<0.001$) were associated with lower phosphate levels at midpoints. In a subgroup of 11 patients observed for 48 weeks, there were significant improvements in patient-reported outcomes in all patients. However, improvements in WOMAC Pain ($r=0.94$, $p=0.02$) and BPI Worst Pain ($r=0.98$, $p<0.001$) were positively correlated with increase in serum phosphate levels at week 48.

**Conclusion:** In a real-world setting, burosumab effectively increased serum phosphate levels and improved clinical outcomes, with greater improvements observed in patients who experienced a more substantial increase in phosphate levels. Baseline serum phosphate, PTH, and FGF23 were significant predictors of phosphate response. Identifying these predictors may help tailor treatment strategies and optimize patient management.

**P241**

### Hungry bone syndrome after parathyroidectomy in a patient with X-linked hypophosphatemia and chronic secondary hyperparathyroidism.

*<u>Stavros Stergianos</u>[1,2], Carolina Nylén[2,3], Kristina Linder Ekberg[1], Sigridur Björnsdottir[2]*
*[1]Karolinska University Hospital, Department of Endocrinology, Stockholm, Sweden*
*[2]Karolinska Institutet, Department of Molecular Medicine and Surgery, Stockholm, Sweden*
*[3]Karolinska University Hospital, Department of Breast and Endocrine Surgery, Stockholm, Sweden*

**Abstract Text**

**Background:** X-linked hypophosphatemia (XLH) is characterized by phosphate wasting and persistent hypophosphatemia. Patients on conventional treatment with active vitamin D and phosphate supplementation are at risk of complications, such as secondary hyperparathyroidism (HPT). This case illustrates the progression from secondary to tertiary HPT in a patient with XLH, highlighting the complexities of perioperative and postoperative management.

**Case Presentation:** A man with XLH, born in 1967, had elevated parathyroid hormone (PTH) levels (13 pmol/L; reference: 1.6–6.0 pmol/L) and two potentially pathological parathyroid glands in 2017. Parathyroidectomy was deferred due to normal urinary calcium and bone turnover markers. In 2020, the patient was diagnosed with nephrocalcinosis. Adherence to vitamin D and phosphate supplementation was inconsistent.

By January 2024, his serum phosphate levels declined to 0,42 mmol/L (reference range: 0.75–1.5 mmol/L). Treatment with alfacalcidol (0.25 µg daily) and phosphate supplementation improved phosphate levels to 0.49 mmol/L, but PTH levels continued to rise (12–16 pmol/L), and ionized calcium levels remained at the upper normal range (1.27–1.32 mmol/L; reference range: 1.15–1.33 mmol/L. By June 2024, worsening phosphate levels (0.25 mmol/L) required intravenous phosphate therapy.

In July 2024, a total parathyroidectomy was performed, and part of the right inferior gland reimplanted into the right forearm. The histopathology report indicated adenomas in all parathyroid glands.

Postoperatively, the patient developed hungry bone syndrome, requiring high doses of alfacalcidol (2.25 µg/day) and calcium (2 g/day), with gradual dose tapering.

**Discussion/Conclusion:** This case highlights the challenges in the management of XLH, particularly concerning the progression from secondary to tertiary HPT. Key challenges include decision-making regarding the timing and extent of surgical intervention and addressing postoperative metabolic complexities such as hungry bone syndrome. Individualized, multidisciplinary approaches are essential for optimizing outcomes in such patients.

**P242**

**A rare case with tumor-induced osteomalacia and inoperable sinonasal glomangiopericytoma that responded to burosumab treatment.**

*Symeon Tournis[1], Michail Krikelis[1], Sousana Gazi[2], Maria Giannopoulou[2], Vasileios Lainis[2], Olga Katsouli[2], Alexia Mpalanika[3], Dimitrios Stergios Evangelopoulos[1], Chronopoulos Efstathios[1]*
*[1]School of Medicine- University of Athens,*
*Laboratory for Research of the Musculoskeletal System LRMS, Athens, Greece*
*[2]KAT General Hospital, Department of Rheumatology, Athens, Greece*
*[3]General Hospital Asklepieio Voulas, Department of Radiology, Athens, Greece*

**Abstract Text**

**Background/Introduction:** Tumor-induced osteomalacia (TIO) is a rare paraneoplastic syndrome characterised by hypophosphataemia, renal phosphate wasting, inadequate bone mineralization, insufficiency fractures and muscle weakness. The primary pathophysiological event is the overexpression of fibroblast-growth-factor 23 (FGF23) by tumors mostly of mesenchymal origin.

**Purpose:** We present a rare case of TIO with an inoperable sinonasal tumor that eventually responded to treatment with burosumab to highlight the efficacy of burosumab in difficult-to-treat cases.

**Case description:** A 73 yrs female patient with abnormal gait, muscle weakness and diffuse musculoskeletal pain (symptom duration of 10 years).

Initial laboratory findings indicated hypophosphataemia [P=1.4 mg/dl (2.3-4.7 mg/dl)] and phosphaturia (TRP 39%) with increased FGF23 [FGF23=391 kRU/l, (26-110 kRU/l)].

The octreoscan revealed a suspect midline lesion situated between the pituitary gland and the cranial orbits and cranial MRI was suggestive of a pathological proliferation (2.5 cm*2 cm) of the ethmoid and frontal sinus with secondary sinusitis. Only partial excision was possible and the biopsy showed a glomagiopericytoma (hemagiopericytoma of sinonasal type) with increased expression of FGF23. Initially, the patient was treated with phosphate salts and alfacalcidol, which partially ameliorated her symptoms. However, due to constantly increasing doses she developed hypercalcemia with nephrocalcinosis, which led to the addition of cinacalcet. BMD progressively deteriorated (-10% in a period of 4 years). As such, she was started on burosumab 0.5mg/kg/m with a rapid normalization of her laboratory values [P=3.3 mg/dl (2.3-4.7 mg/dl); TRP=81%]. Remarkably, her bone mineral density increased by 11% at the lumbar spine and by 7% in the total hip after only six months of burosumab treatment.

**Conclusion:** Burosumab is an effective treatment in TIO patients with inoperable tumors who do not tolerate or who deteriorate despite conventional treatment.

**P243**

**Endogenous Glucocorticoids Promote Neurogenic Heterotopic Ossification After Spinal Cord Injury**

*Kylie Alexander[1], Hsu-Wen Tseng[1], Dorothée Girard[2,3], Hong Wa Lao[4], Jacobus Ungerer[5], Brett McWhinney[5], Valerie Barbier[1], Selwin Samuel[1], Whitney Fleming[1], Marjorie Salga[6], François Genêt[6,7], Marc Ruitenberg[4], Sébastien Banzet[2,3], Jean-Pierre Levesque[1]*
*[1]Mater Research, The University of Queensland, Brisbane, Australia*
*[2]Institut de Recherche, Biomédicale des Armées, Clamart, France*
*[3]Inserm UMR-MD U1197, SToRM, Clamart, France*
*[4]School of Biomedical Sciences- Faculty of Medicine, The University of Queensland, Brisbane, Australia*
*[5]Pathology Queensland, Royal Brisbane and Women's Hospital, Brisbane, Australia*
*[6]UPOH Unité Péri Opératoire du Handicap- Perioperative Disability Unit- Physical and Rehabilitation Medicine department, Hôpital Raymond-Poincaré- Assistance Publique – Hôpitaux de Paris AP-HP, Garches, France*
*[7]Université Versailles Saint-Quentin-en-Yvelines UVSQ,*
*UFR Simone Veil - Santé- END: ICAP- INSERM U1179, Montigny-le-Bretonneux, France*

**Abstract Text**

**Background:** Neurogenic heterotopic ossifications (NHOs) are periarticular ectopic bones that develop after central nervous system insults such as spinal cord injuries (SCI). NHOs are debilitating, causing joint ankylosis, with the only therapy being surgical resection. How a central nervous system injury triggers heterotopic bone formation remains unknown, precluding the use of targeted therapies.

**Purpose and Method:** To elucidate NHO pathophysiology we utilized our model of NHO where mice undergo SCI followed by a muscle injury induced by an intramuscular injection of cardiotoxin, where NHO only develops in injured muscles after SCI.

**Results:** We demonstrate that SCI triggered an acute glucocorticoid (GC) (corticosterone) spike, which was causal as mice treated with corticosterone or the glucocorticoid receptor (GR) agonist dexamethasone, developed heterotopic ossifications in injured muscles in the absence of SCI (Fig1A $p=0.0146$). RNA sequencing of whole injured muscles from mice revealed that dexamethasone treatment upregulated the expression of osteoinductive and osteogenic differentiation genes. Interestingly, dexamethasone treatment in mice with SCI and muscle injury further increased NHO development compared to saline treated controls (Fig1B 10.81mm$^3$ vs 24.38 mm$^3$ $p=0.0015$).

The central role for GR signaling in driving NHO was further demonstrated in mice with a tamoxifen-inducible post-natal deletion for the GR gene (Nr3c1$^{flox/flox}$|Rosa26$^{CreERT2}$), which did not develop NHO compared to GR wildtype CreERT2 controls (Fig1C 41.51mm$^3$ vs 0.05mm$^3$ $p<0.0001$). Furthermore, administration of clinical GR antagonists for the first 48 hours after surgery, significantly inhibited NHO development, whereas side-by-side comparative treatment with indomethacin had no significant effect (Fig1D vehicle 2.8mm$^3$ vs mifepristone 0.23mm$^3$ vs indomethacin 6.99mm$^3$ $p=0.027$). Finally, we demonstrate that GR antagonist treatment does not negatively impact SCI lesion volume in a mouse model of contusive SCI (Fig1E).

**Conclusion:** This study identifies endogenous GCs driving pathological NHO in injured muscles after SCI and GR antagonists may be of prophylactic use to prevent NHO.

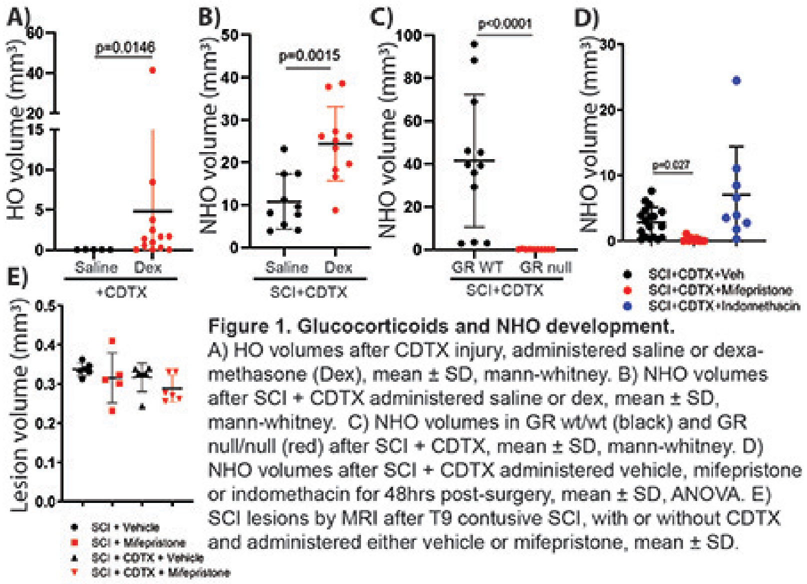

**Figure 1. Glucocorticoids and NHO development.** A) HO volumes after CDTX injury, administered saline or dexamethasone (Dex), mean ± SD, mann-whitney. B) NHO volumes after SCI + CDTX administered saline or dex, mean ± SD, mann-whitney. C) NHO volumes in GR wt/wt (black) and GR null/null (red) after SCI + CDTX, mean ± SD, mann-whitney. D) NHO volumes after SCI + CDTX administered vehicle, mifepristone or indomethacin for 48hrs post-surgery, mean ± SD, ANOVA. E) SCI lesions by MRI after T9 contusive SCI, with or without CDTX and administered either vehicle or mifepristone, mean ± SD.

**P244**

**Bone marrow adiposity: an underlying mechanism of the development of cystic fibrosis-related bone diseases**

*Laurine Hamon*[1]*, Claire Dumortier*[1,2]*, Léa Thoraval*[1]*, Johan Sergheraert*[1,3]*, Olivier Piot*[4]*, Emilie Buache*[4]*, Nicolas Goffin*[4]*, Christophe Chauveau*[5]*, Sophie Gangloff*[1]*, Denise Al Alam*[2]*, Marie-Laure Jourdain*[1,3]*, Frédéric Velard*[1]

[1]*Université Reims Champagne-Ardenne, UR BIOS « Biomatériaux et Inflammation en site Osseux », Reims, France*

[2]*The Lundquist Institute, Harbor-UCLA Medical Center, Torrance, United States*

[3]*CHU Reims, Service de Médecine Bucco-Dentaire, Reims, France*

[4]*Université Reims Champagne-Ardenne, UR BioSpeCT "BioSpectroscopie Translationnelle", Reims, France*

[5]*Université Littoral Côte d'Opale, ULR 4490 MabLab "Marrow Adiposity & Bone Lab", Reims, France*

**Abstract Text**

Cystic Fibrosis-related Bone Disease (CFBD) affects 50% of CF adult patients in whom bone formation has been demonstrated altered (Velard, 2014). Furthermore, osteoblasts obtained from CF patients' induced pluripotent stem cells evidenced a higher PPARG level (adipocyte transcription factor) than healthy donors-derived control line (Dumortier, 2025). A relationship exists between low bone density and increased medullar adiposity (Rozman, 1989 ; Verma, 2002 ; Zhou, 2008 ; Piccinin, 2014). We hypothesised that adipocyte differentiation would be preferentially triggered in mesenchymal stem cells (MSC) bearing mutation in *CFTR* gene, which causes CF.

MSC from healthy donors have been differentiated into osteoblasts and adipocytes during 21 days with or without $Inh_{172}$ or BPO27 (CFTR inhibitors) (n=7). Oil Red'O, Bodipy™, FABP4 and PPARG stainings were performed. Raman microspectroscopy was used to assess lipid content. MicroCT analysis of WT and F508delCFTR mice at 8 and 16 weeks after a surgical ovariectomy (OVX), or sham-operated.

The number and size of lipid vesicles increased due to CFTR inhibition ($p<0.05$). Raman analyses showed an increased saturation degree of lipids. Spontaneous lipid droplets generation was evidenced in MSC and osteoblasts. In addition, we evidenced decreased trabecular thickness in CF mice compared to WT, highlighting an alteration of bone microarchitecture. The number of bone trabeculae and bone mineral density decreased in contrast to the trabecular separation that increased in OVX-CF mice *versus* CF mice.

These data are the first proof of concept reflecting MSC commitment towards adipocyte lineage due to a loss of CFTR function. More, we evidenced a more pronounced manifestation of CFBD in OVX mice, thus highlighting the potential impact of post-menauposis on female CF patients. Finally, understanding the formation and role of lipid metabolism may be a promising axis to better understand and cure CFBD.

**P245**

**Investigating a Mouse Model for Enpp1-associated Early-Onset Osteoporosis**

Timur Yorgan[1], Sofie Dragoun Kolibová[1], Simon von Kroge[1], Michael Amling[1], Thorsten Schinke[1], Ralf Oheim[1]
[1]University Medical Center Hamburg-Eppendorf, Department of Osteology and Biomechanics, Hamburg, Germany

**Abstract Text**

**Introduction:** Heterozygous pathogenic variants of *ENPP1* (ectonucleotide pyrophosphatase/phosphodiesterase family member 1) can cause early-onset osteoporosis. While progress has been made to understand the pathomechanism of general arterial calcification of infants (GACI) and hypophosphatemic rickets (ARHR2) that are caused by biallelic *ENPP1* variants, this process is not well understood for early-onset osteoporosis. To gain better insights into the pathomechanism and explore treatment options for improving bone mass without inducing ectopic calcifications *Enpp1*-asj-2J mice were analyzed.

**Purpose:** The purpose of this study was to establish a murine model for *ENPP1*-related early-onset osteoporosis and to test if reducing Sclerostin activity is a suitable approach to increase bone mass.

**Methods:** Skeletons of *Enpp1*-asj-2J mice were analyzed at 6, 24, 52 and 75 weeks of age. To mimic osteoanabolic therapy, the *Lrp5*-A213V variant that cannot bind Sclerostin was introduced into *Enpp1*-asj2J mice. Bone structure was determined by µCT and histomorphometry. Ectopic calcifications were assessed by x-ray and histology. Ethical approvals were present.

**Results:** Regarding $Enpp1^{+/asj\text{-}2J}$ mice, only male 75-week-old animals displayed a reduction in trabecular bone mass [4.1 vs. 6.6 % BV/TV, p=0.029]. However, the bone mass of 6-week-old $Enpp1^{asj\text{-}2J/asj\text{-}2J}$ mice was significantly and consistently reduced [6.8 vs. 15.7 % BV/TV, p=0.004]. Importantly, this osteopenic phenotype occurred prior to the onset of severe ectopic calcifications. Introduction of the *Lrp5*-A213V high-bone-mass-allele resulted in a 93 % increase of bone mass in 8-week-old male $Enpp1^{asj\text{-}2J/asj\text{-}2J}$ mice, comparable to the effect observed in wildtype animals (72 %). Furthermore, no changes in the occurrence and severity of ectopic calcifications were observed in relation to the Lrp5-A213V genotype.

**Conclusions:** Young (6-8-weeks-old) homozygous *Enpp1*-asj-2J mice are a suitable model for ENPP1-related early-onset osteoporosis. Our preclinical data indicate that reduction of Sclerostin-activity may be an effective and safe option to treat this disorder.

**P246**

**Baseline characteristics of the TOPAZ study: A randomised trial of teriparatide and zoledronic acid compared with standard care in adults with osteogenesis imperfecta**

Jannie Dahl Hald[1], Christopher Weir[2], Catriona Keerie[2], Dewar Lorna[2], Jennifer Walsh[3], Richard Keen[4], Matthew Roy[5], Terry Aspray[6], Mashood Siddiqi[7], Zaki Hassan-Smith[8], Kassim Javaid[9], Kenneth Poole[10],

Hrushikesh Divyateja[11], Mike Stone[12], Rosemary Hollick[13], Ellen Malcolm[14], Nazim Ghouri[15], Zoe Paskins[16], Geeta Hampson[17], Stephen Tuck[18], Prashanth Patel[19], Harvey Nick[20], Elizabeth Curtis[20], John Lindsay[21], Rachel Crowley[22], Martine Cohen-Solal[23], Marelise Eekhof[24], Feenstra Johannes[25], Bente Langdahl[1], Jonathan Tang[26], Wayne Lam[27], Osborne Patricia[28], David Moore[27], Stuart Ralston[29]
[1]Aarhus University Hospital, Clinical Medicine – Endocrinology and Metabolism C, Aarhus, Denmark
[2]University of Edinburgh, Edinburgh Clinical Trials Unit, Edinburgh, United Kingdom
[3]University of Sheffield, Metabolic Bone Centre, Sheffield, United Kingdom
[4]Royal National Orthopaedic Hospital NHS Trust, Metabolic Bone Clinic, London, United Kingdom
[5]University Hospitals Bristol NHS Trust, Bristol Royal Infirmary, Bristol, United Kingdom
[6]University of Newcastle, Bone Clinic- Freeman Hospital, Newcastle, United Kingdom
[7]Aintree University Hospital NHS Foundation Trust, Department of Rheumatology, Liverpool, United Kingdom
[8]Queen Elizabeth Hospital, Department of Endocrinology, Birmingham, United Kingdom
[9]University of Oxford, Nuffield Orthopaedic Centre, Oxford, United Kingdom
[10]Cambridge University Hospitals NHS Trust, Addenbrookes Hospital, Cambridge, United Kingdom
[11]Nottingham University Hospitals NHS Trust, Chemical Pathology, Nottingham, United Kingdom
[12]University Hospital Llandough, Metabolic Bone Clinic, Cardiff, United Kingdom
[13]University of Aberdeen, School of Medicine- Medical Sciences and Nutrition, Aberdeen, United Kingdom
[14]Ninewells Hospital and Medical School, Department of Medicine, Dundee, United Kingdom
[15]Queen Elizabeth University Hospital, Endocrinology, Glasgow, United Kingdom
[16]University of Keele, Rheumatology, Stafford, United Kingdom
[17]Guys & St Thomas NHS Trust, Metabolic Bone Clinic, London, United Kingdom
[18]South Tees Hospitals NHS Foundation Trust, Rheumatology, Middlesborugh, United Kingdom
[19]Leicester Royal Infirmary, Chemical Pathology, Leicester, United Kingdom
[20]University Hospital Southampton, MRC Lifecourse Epidemiology Unit, Southampton, United Kingdom
[21]Belfast Health and Social Care Trust, Royal Victoria Hospital, Belfast, United Kingdom
[22]St. Vincent's University Hospital, Department of Endocrinology, Dublin, Ireland
[23]Hôpital Lariboisiére, Department of Rheumatology, Paris, France
[24]Amsterdam University Medical Centre, Endocrinology, Amsterdam, Netherlands
[25]Isala Hospital Zwolle, Endocrinology, Zwolle, Netherlands
[26]Norwich Medical School, Bioanalytical Facility, Norwich, United Kingdom
[27]South East Scotland NHS Genetics Service, Centre for Genomic and Experimental Medicine, Edinburgh, United Kingdom
[28]Brittle Bone Society, Chief Executive Office, Dundee, United Kingdom
[29]University of Edinburgh, Institute of Genetics and Cancer, Edinburgh, United Kingdom

**Abstract Text**

**Introduction:** Osteogenesis imperfecta (OI) is a rare disorder causing multiple fractures throughout life. Medical treatment of OI is based on giving drugs that are used in the treatment of osteoporosis but the evidence-base for efficacy at preventing fractures is unknown.

**Purpose:** To present the baseline characteristics of participants in the Treatment of Osteogenesis Imperfecta with Parathyroid Hormone and Zoledronic Acid (TOPAZ) trial. The aim of the trial is to determine whether Teriparatide and Zoledronic acid are superior to standard care in reducing the risk of clinical fractures

**Methods:** We summarised data on the baseline characteristics of TOPAZ including demographics, genetic diagnosis, clinical features, bone density measurements, previous treatments and fracture history.

**Results:** We recruited 350 adults with OI to the study over a 6-year period. Most (71.1%) had type I OI, 15.7% had type IV and 5.4% had type III. The remainder had other OI types. On clinical evaluation, 81% had blue sclera and 36% had dentinogenesis imperfecta. Antiresorptive treatment had been administered to 83% of subjects at some point during their lifetime but only 30% had received these drugs during the two years prior to enrolment. Genetic testing revealed 78% had mutations in *COL1A1* or *COL1A2*. In these participants, 46% were quantitative mutations, 37% were qualitative mutations and 20% were splice site mutations. Four had mutations in *IFITM5*, and two each had *SERPINF1* and *PH31* mutations. In 19%, no pathogenic mutations were found, even though all individuals had a clinical diagnosis of OI. At the present time closeout visits have all been completed and data analysis is in progress.

**Conclusions:** The TOPAZ trial is the largest randomised trial that has even been conducted in OI, and the results will provide new insights into the effect of an anabolic therapy, followed by antiresorptive treatment in the management of this condition

**P247**

**Clinical features of a multicenter Italian cohort of adult patients with X-linked hypophosphatemia**

*Silvia Carrara[1], Giampiero Baroncelli[2], Gaetano Paride Arcidiacono[3], Marco Barale[4], Maria Luisa Brandi[5,6], Valentina Camozzi[7], Elena Castellano[8], Filomena Cetani[9], Pasquale Comberiati[2,10], Simone Della Valentina[11], Cristina Eller-Vainicher[12], Nadia Edvige Foligno[13], Sandro Giannini[3], Laura Gianotti[14], Giorgia Grassi[12], Martina Laganà[15], Gemma Marcucci[16], Laura Masi[17], Francesca Paglia[18], Andrea Palermo[19], Francesca Pigliaru[20], Massimo Procopio[21], Vincenzo Rochira[18,22], Rosaria Maddalena Ruggeri[15], Marco Onofrio Torres[3], Silvia Vai[23], Giuseppe Vezzoli[13], Marta Zampogna[1], Sabrina Corbetta[23,24]*
*[1]University of Milan, Department of Medical Biotechnology and Translational Medicine, Milan, Italy*
*[2]University Hospital of Pisa, Pediatric and Adolescent Endocrinology- Division of Pediatrics- Department of Obstetrics- Gynecology and Pediatrics, Pisa, Italy*
*[3]University of Padova, Clinica Medica 1- Department of Medicine, Padua, Italy*
*[4]University of Turin, Division of Oncological Endocrinology- Department of Medical Sciences, Turin, Italy*
*[5]Villa Donatello Hospital, Donatello Bone Clinic, Florence, Italy*
*[6]F.I.R.M.O. Onlus Italian Foundation for the Research on Bone Diseases, F.I.R.M.O. Onlus Italian Foundation for the Research on Bone Diseases, Florence, Italy*
*[7]University-Hospital of Padova, Endocrine Unit, Padua, Italy*

[8]*Santa Croce and Carle Hospital, Department of Endocrinology- Diabetes and Metabolism, Cuneo, Italy*
[9]*University of Pisa, Endocrine Unit 2- Department of Clinical and Experimental Medicine, Pisa, Italy*
[10]*University of Pisa, Department of Clinical and Experimental Medicine- Section of Paediatrics, Pisa, Italy*
[11]*University of Pisa, Department of Clinical and Experimental Medicine, Pisa, Italy*
[12]*Fondazione IRCCS Cà Granda Ospedale Maggiore Policlinico, SC Endocrinologia, Milan, Italy*
[13]*IRCCS San Raffaele Scientific Institute, Nephrology and Dialysis Unit, Milan, Italy*
[14]*ASL Cuneo 1, Struttura Complessa di Endocrinologia e Diabetologia Territoriale, Cuneo, Italy*
[15]*University of Messina,*
*Dipartimento di Patologia Umana dell'Adulto e dell'Età Evolutiva "Gaetano Barresi"- Unità di Endocrinologia, Messina, Italy*
[16]*University Hospital of Florence,*
*Bone Metabolic Diseases Unit- Department of Experimental and Clinical Biomedical Sciences, Florence, Italy*
[17]*University Hospital of Florence AOUC, Metabolic Bone Diseases Unit, Florence, Italy*
[18]*Azienda Ospedaliero-Universitaria Policlinico di Modena- Ospedale Civile di Baggiovara, Endocrinology Unit- Department of Medical Specialties, Modena, Italy*
[19]*Fondazione Policlinico Campus Bio-medico, Unit of Metabolic Bone and Thyroid Disorders, Rome, Italy*
[20]*AOU Cagliari, Endocrinology Unit, Cagliari, Italy*
[21]*University of Turin,*
*Division of Endocrinology- Diabetes and Metabolism- Department of Medical Sciences, Turin, Italy*
[22]*University of Modena and Reggio Emilia,*
*Endocrinology- Department of Biomedical- Metabolic and Neural Sciences, Modena, Italy*
[23]*IRCCS Istituto Auxologico Italiano, Bone Metabolism Disorders and Diabetes Unit, Milan, Italy*
[24]*University of Milan, Department of Biomedical- Surgical and Dental Sciences, Milan, Italy*

**Abstract Text**

X-linked hypophosphatemia (XLH) is a rare genetic disease characterized by renal phosphate wasting, rickets and osteomalacia.

A cross-sectional study involving 14 Italian tertiary centers was performed, with the aim of describing the clinical features of XLH adults.

The Italian cohort includes 100 females and 58 males, aged 45.0±14.7 years (19-83). *PHEX* mutations were identified in 96.4% of cases, without evident genotype/phenotype correlation.

The most prevalent feature was skeletal deformities, mainly genu varus, recorded in 87.3% of XLH individuals. Fractures/pseudofractures occurred in 43.7% of cases. Two third of patients had marked osteophytosis, while enthesopathies, affecting knees and scapulohumeral joints, were detected in about a half of the individuals. Severe dental disease was experienced by two thirds of patients. Males showed a more severe phenotype than females, with higher prevalence of fractures/pseudofractures (58.6% vs. 35.4%, p=0.005) and dental disease (84.5% vs. 66.0%, p=0.012).

Plasma FGF23 levels – measured in 51 patients (median 109 pg/ml, IQR 104 pg/ml) – were higher than the upper limit of normal range in 58.8% of individuals, though they were >30 pg/ml in all patients except one. Interestingly, higher FGF-23 levels were associated with higher prevalence of hypertension (26.7% vs. 4.8%; p=0.043).

Regarding their cardiometabolic profile, 22 patients were hypertensive (13.9%), 16 dyslipidemic (10.1%), while none had diabetes. Two patients reported previous cerebrovascular events.

At the time of evaluation, 43.7% of patients were on conventional therapy, whereas 34.1% were on burosumab; 22.2% was not taking any specific treatment.

In conclusion, the burden of XLH disease in adulthood is determined by musculoskeletal manifestations and dental disease, being more severe in males. Cardiovascular risk does not appear to be increased and may be related to FGF23 levels. The disease burden affects a proportion of patients higher than that treated with burosumab, which should be more extensively provided.

**P249**

**Presenting features, risk factors and treatment effectiveness in pregnancy associated osteoporosis: a systematic review and meta-analysis**

*Elizabeth Orhadje*[1], *Kathryn Berg*[1], *Navnit Makaram*[1], *Barbara Hauser*[1], *Stuart H Ralston*[1]
[1]*Centre for Genomic and Experimental Medicine- Institute of Genetics and Cancer, University of Edinburgh, Edinburgh, United Kingdom*

**Abstract Text**

**Introduction:** Pregnancy-associated osteoporosis (PAO) is a rare condition typically presenting with multiple vertebral fractures during pregnancy or lactation.

**Purpose:** We conducted a systematic review of the presenting features and risk factors for PAO, and performed a meta-analysis to evaluate the effects of bisphosphonates, teriparatide and calcium/vitamin D supplements on bone mineral density (BMD).

**Methods:** We searched PubMed, EMBASE and Web of Science for clinical studies of PAO, defined by the authors as having fractures and/or low BMD during the last trimester of pregnancy or the first six months postpartum. This resulted in the identification of 35 studies with 943 cases.

**Results:** Their (weighted) mean age was 34.2 and 80.6% were first-time mothers. Most presented with vertebral fractures (89.2%) and back pain (90.2%). A family history of osteoporosis was recorded in 40.5%. Genetic analysis was performed in 21 women, revealing pathogenic or likely pathogenic variants in 8 affecting *COL1A1*, *COL1A2*, *LRP5* and *WNT1*. Calcium (31.3%) and vitamin D (38.1%) supplements were the most common treatments, followed by teriparatide (30.8%). Lack of standardisation of reporting BMD changes hampered attempts to conduct a quantitative analysis of different treatments. The change in BMD at lumbar spine was greater with teriparatide (but based on only 2 studies) compared with calcium/vitamin D and bisphosphonates (Table 1), but there was no difference in BMD response at the femoral neck. Recurrent fractures were reported in 12.9% with no difference between treatment groups.

**Conclusion:** This review provides insights into the presentation and management of PAO while highlighting the limitations of knowledge about the condition and its treatment. This might help to inform conduct of a Delphi process on the diagnosis and management of this disorder, pending conduct of randomised controlled trials. Defining a core outcome set would also help strengthen future systematic reviews by standardising data collection and reporting.

| | Number of studies | Mean change (%) | Lower 95% CI | Upper 95% CI |
|---|---|---|---|---|
| **Lumbar spine BMD** | | | | |
| Calcium/vitamin D | 7 | 0.306 | 0.225 | 0.397 |
| Bisphosphonate | 4 | 0.354 | 0.245 | 0.463 |
| Teriparatide | 2 | 0.830 | 0.458 | 1.202 |
| **Femoral neck BMD** | | | | |
| Calcium/vitamin D | 4 | 0.291 | 0.054 | 0.529 |
| Bisphosphonate | 3 | 0.104 | 0.066 | 0.141 |
| Teriparatide | 2 | NC | NC | NC |

**Table 1. Meta-analysis of bone mineral density (BMD) response to treatment.**
CI = confidence interval, NC = not calculable.

**P250**

**Highlighting the bone remodeling alterations in Mucopolysaccharidosis type II**

*Sara Terreri[1], Michela Rossi[1], Laura Di Giuseppe[1], Giulia Battafarano[1], Jacopo Di Gregorio[2], Olivia Pagliarosi[3], Federica Deodato[4], Andrea Del Fattore[1]*
*[1]Bambino Gesù Children's Hospital, Bone Physiopathology Research Unit, Rome, Italy*
*[2]University of L'Aquila, Department of Biotechnology and Applied Clinical Sciences, L'Aquila, Italy*
*[3]Policlinico Umberto I- "Sapienza" University of Rome,*
*Department of Clinical- Internal- Anesthesiology and Cardiovascular Sciences, Rome, Italy*
*[4]Bambino Gesù Children's Hospital, Metabolic Diseases and Hepatology Unit, Rome, Italy*

**Abstract Text**

Mucopolysaccharidosis type II (Hunter Syndrome) is a rare, x-linked recessive disease caused by the deficiency of iduronate-2-sulfatase, which leads to the pathological storage of glycosaminoglycans (GAG) in multiple tissues causing progressive damage and a variety of multi-organ clinical manifestations. Currently, the therapeutic approaches are based on enzyme replacement therapy and hematopoietic stem cell transplantation, but their effect on bone lesions remains limited. In this study we dissected the defects of bone cells in MPSII patients to identify potential targets for testing new therapeutic approaches.
Our preliminary data showed a reduced ability of Peripheral Blood Mononuclear Cells (PBMC) of MPSII patients to differentiate into osteoclasts (OC) compared to healthy donors (HD) (number of OC/field; HD: 74.6±25.6, MPSII: 30.5±16.3. p<0.01). MPSII OC displayed reduced number of nuclei/osteoclasts, suggesting altered fusion ability of patient-derived osteoclast precursors. Accordingly, MPSII osteoclasts appeared smaller than control cells (OC size; HD: 1.04±0.16, MPSII: 0.43±0.07. p<0.001). To investigate whether the altered osteoclastogenesis could be also due to changes of osteoclast precursors (pOC), we performed FACS analysis of PBMC; although we did not reveal alterations of pOC number in patients, we found a reduction of $CD16^-$ and an increase of $CD16^+$ inside pOC population [$CD16^-$ (%); HD: 90.6±5.7, MPSII: 78.6±16.5. p<0.01. $CD16^+$ (%); HD: 8.8±5.5, MPSII: 21±16.2. p<0.01.], thus supporting the reduced ability of patients' PBMC to differentiate into osteoclasts.
In conclusion, our preliminary data revealed alterations of osteoclasts in MPSII, revealing how GAG accumulation could affect osteoclast differentiation in patients.

**P252**

**Higher incidence of fracture in adults with osteogenesis imperfecta: identification of the risk factors**

*Camille Blandin*[1], *Corinne Collet*[2], *agnes Ostertag*[2], *Thomas Funck Brentano*[2], *Martine Cohen Solal*[2]
[1]*Lariboisiere hospital, rheumatology, Paris, France*
[2]*lariboisiere hospital, inserm u1132, paris, France*

**Abstract Text**

**Background:** Fractures are recurrent in adults with osteogenesis imperfecta (OI). There are no identified risk factors that predict the occurrence of fracture in adult life.

**Objectives:** We investigated prospectively the factors associated to the occurrence of fractures in adults with OI followed in a Reference Centre of Rare Bone Diseases. Methods : Among the 106 patients with OI, we included those with BMD measures at one or more skeletal sites (total hip [TH], lumbar spine [LS] or the radius [Rd]), and naïve of bone treatment at baseline. From 2000 to 2022, we collected the clinical data, genotype, BMD and incident fractures from inclusion. We investigated the probability of fracture during follow-up via a logistic regression analysis as a function of age, Z-Score BMD and variant pathogenicity.

**Results:** 35 patients could not analysable because BMD measures were not reliable for technical limitations. 71 patients were included at the initial visit (44 women and 27 men, mean age of 41.4 ± 13.7 years) and were followed for 5.05 years (IQR: 3.18 – 8.75 y). Baseline BMD revealed a low Z-Score (-2.7 ± 1.5 SD) at the LS only affecting mainly men (Z-Score -3 ± 1.6 SD). The mean number of fractures that occurred during the follow-up was higher in the presence of missense variants of *COL1* ($p<0.05$). The logistic regression adjusted for the determinants revealed a high probability of fracture with BMD Z-score < -2 SD at any of the 3 skeletal sites (OR 4.38, IC 1.10-21.75, p=0.048) and with missense variants only (stop codon, frameshift variants, OR 29, IC 2.56-1503, p=0.02).

**Conclusion:** our OI cohort revealed a low BMD z-score at the LS. The probability of fractures was higher with low BMD and missense variants. These data identify the risk factors associated with fractures that could be used for treatment decision.

**P253**

**No knuckles, four dimples: Acrodysostosis - a case report**

*Pradakshna Porchezhian*[1], *Eram Nahid*[1], *Kanav Khanna*[1], *Nayankumar G. Tada*[2], *Ravindra Shukla*[1], *Kuldeep Singh*[3], *Gopal Krishana Bohra*[1]

[1]All India Institute of Medical Sciences, Endocrinology & Metabolism, Jodhpur, India
[2]BJ Medical College, Pediatrics, Ahmedabad, India
[3]All India Institute of Medical Sciences, Pediatrics, Jodhpur, India

**Abstract Text**

**Background:** Acrodysostosis is an extremely rare skeletal dysplasia due to mutations in the parathyroid hormone (PTH) receptor pathway.

**Case description:** A 16-years-old girl presented with obesity and inadequate height gain since four years of age. Earlier at 10 years of age, she was diagnosed with celiac disease and hypothyroidism, was started on gluten-free diet and levothyroxine. In view of no clinical response, she presented to us at 16-years. Examination revealed height 138cm (-3.37Z score), weight 52.3Kg (0.18Z), BMI 27.5kg/m$^2$. She had severe extensive acanthosis nigricans, round facies, and brachydactyly resembling Albright's hereditary osteodystrophy (AHO) phenotype. Systemic examination within normal limits.

**Results:** Hemogram, renal and liver function tests were normal, with low-normal serum calcium, elevated phosphorus and intact parathormone and low 25-OH-vitamin-D levels. Thyroid function test revealed elevated TSH despite a compliant intake of levothyroxine (2mcg/kg/day). With anti-TPO antibodies negativity, high $LT_4$ requirement and well-managed celiac disease, TSH resistance was considered and the dose tapered gradually to 1mcg/kg/day (50mcg/day). NCCT head showed bilateral punctate basal ganglia calcifications. Pure tone audiometry and IQ assessment unfolded right moderate mixed hearing loss and below-average IQ. Therefore, inactivating PTH/PTHrP signaling disorders (iPPSDs) were considered and genetic testing revealed a heterozygous pathogenic variant of c.453T>A(p.Asp151Glu) in exon-5 of *PRKAR1A* gene. She was continued on a gluten-free diet, thyroxine replacement and cholecalciferol supplementation.

**Discussion:** Acrodysostosis (MIM#101800), a rare skeletal dysplasia comprises hormonal resistance, intellectual disability, hearing defects, obesity and metabolic syndrome with several overlapping features with pseudohypoparathyroidism (PHP-1A) and pseudo-PHP. Although varying autoimmune diseases are reported with pseudohypoparathyroidism, no such reports exist with acrodysostosis. With only a handful of cases reported so far, outright clinical spectrum of this relatively contemporary disorder needs to be unveiled.

**Conclusion:** We report a novel mutation of PRKAR1A gene in a case of acrodysostosis with celiac disease which has not been previously reported.

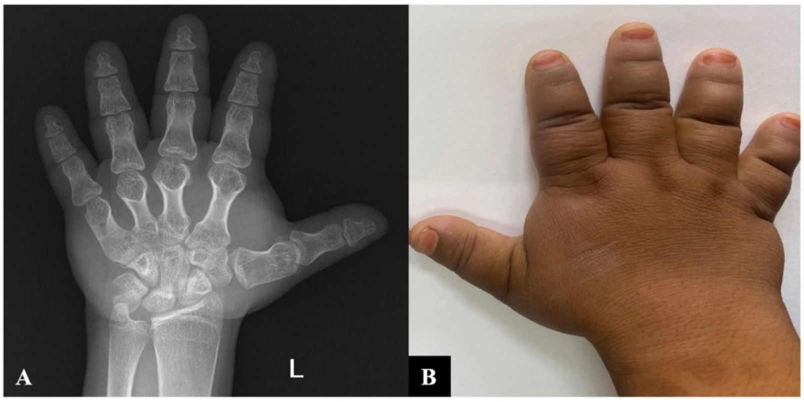

**Figure 1: Brachydactyly in Acrodysostosis**

**P254**

### A novel form of autosomal dominant spondylocostal dysostosis in three unrelated families caused by the same heterozygous pathogenic variant in MESP2

*Aneta Malesa[1], Maria Arismendi[2], Gabriela Andrade Vasques[2], Alexander A. L. Jorge[2], Enrique Galán-Gómez[3], Karen E. Heath[4], Wim Van Hul[5], Geert Mortier[1,6], Gretl Hendrickx[1]*
*[1]KU Leuven, Department of Human Genetics, Leuven, Belgium*
*[2]University of São Paulo School of Medicine, Division of Endocrinology and Genetic Endocrinology, São Paulo, Brazil*
*[3]University Children's Hospital, Clinical Genetics Unit, Badajoz, Spain*
*[4]Hospital Universitario la Paz and UMDE-ERN BOND,*
*Institute of Medical & Molecular Genetics INGEMM, Madrid, Spain*
*[5]Antwerp University Hospital and University of Antwerp, Department of Medical Genetics, Antwerp, Belgium*
*[6]University Hospital Leuven, Center of Human Genetics, Leuven, Belgium*

**Abstract Text**

**Background:** Spondylocostal dysostosis (SCD) is a rare genetic bone disorder characterized by disproportionate short stature, scoliosis, vertebral segmentation defects (e.g. hemivertebrae, butterfly vertebrae) and rib abnormalities occurring during embryonic development. SCD typically shows a recessive inheritance and several causal genes have been identified. These genes play a crucial role in somitogenesis and the subsequent formation of the vertebral column. Families with an autosomal dominant form of SCD have been reported, but only in one family a heterozygous mutation in *TBX6* was identified.

**Purpose:** This study aimed to identify the genetic and mechanistic basis in *TBX6*-negative families with a dominant form of SCD.

**Methods:** Genome sequencing was performed on three unrelated families with dominant SCD. Prediction programs were used to assess the impact of the identified mutation. The effect of the mutation on Notch signaling activity and subcellular protein localization was verified by immunostaining and confocal microscopy and luciferase reporter assays in HEK293T cells, respectively.

**Results:** Genome sequencing on a Belgian family with a dominant form of SCD led to the identification of a heterozygous pathogenic *MESP2* variant (c.268G>A; p.Glu90Lys). Interestingly, the same *MESP2* mutation was identified in a Brazilian and a Spanish family with a dominant form of SCD. The p.Glu90Lys variant was evaluated as disease-causing *in silico* and affects a highly-conserved amino acid in the basic helix-loop-helix domain of the MESP2 transcription factor, responsible for DNA binding. Functional studies demonstrated a defect in the transcriptonal regulation of *LFNG* expression and a more dominant nuclear localization of p.Glu90Lys MESP2, compared to wildtype MESP2. These data strongly suggest a dominant negative effect of p.Glu90Lys MESP2.

**Conclusions:** We provide the first evidence that a heterozygous mutation in *MESP2* can cause an autosomal dominant form of SCD. Ongoing studies aim to further determine the functional effects of this mutation on somitogenesis and vertebral segmentation, both *in vitro* and *in vivo*.

**P255**

**Induction of ER stress as a mechanism to modulate RANKL expression in Fibrous dysplasia of bone**

*Chiara Tavanti*[1], *Federica Campolo*[2], *Giorgia Farinacci*[1], *Alessandro Corsi*[1], *Mary Anna Venneri*[2], *Antonella Forlino*[3], *Roberta Besio*[3], *Biagio Palmisano*[1], *Mara Riminucci*[1]
[1]*Sapienza University of Rome, Department of Molecular Medicine, Rome, Italy*
[2]*Sapienza University of Rome, Department of Experimental Medicine, Rome, Italy*
[3]*University of Pavia, Department of Molecular Medicine- Biochemistry Unit, Pavia, Italy*

**Abstract Text**

Fibrous dysplasia (FD) of bone is a skeletal disorder caused by post-zygotic activating mutations (R201C/R201H) of the Gαs gene. FD leads to the replacement of normal bone and marrow with a pathological fibro-osseous tissue, resulting in severe bone fragility and pain. FD bone lesions arise from the abnormal function of both osteoprogenitor cells, which are the main target of the mutation, and osteoclastic cells, which are recruited at sites of lesion development through enhanced expression of Receptor Activator of Nuclear Factor-kappa B Ligand (RANKL).

The endoplasmic reticulum (ER) stress/unfolded protein response (UPR) is known to contribute to various skeletal diseases by affecting osteogenic cell fate and function as well as RANKL secretion and osteoclast formation. However, its role in FD has never been investigated.

To explore this, we cultured osteogenic cells isolated from caudal vertebrae of wild-type (WT) and FD transgenic mice (EF1a-Gαs$^{R201C}$ mice) and exposed them to the ER stress activator tunicamycin according to different concentrations and treatment duration: 1) 2 mg/ml, 4 hours; 2) 2 mg/ml, 16 hours; 3) 5 mg/ml, 16 hours.

Gαs-mutated osteoprogenitor cells showed an ER stress response similar to WT cells, as assessed by the gene expression of *Edem1*, *Chop* and *Atf4*, and the quantification of the spliced version of *Xbp1* (*Xbp1s*). Interestingly, ER stress activation in both WT and Gas-mutated osteogenic cells led to a significant reduction in the level of expression of *Rankl* expression, especially in the 16-hourt treatment groups.

Although very preliminary, these results confirm that the ER stress/UPR pathway modulates RANKL expression in osteogenic cells. Most important, they suggest that this molecular pathway may represent a potential pharmacological target for FD.

**P256**

**Addressing the Unmet Challenge of Pain in Rare Bone Diseases**

Melanie Legrand[1], Roland Chapurlat[2], Kassim M Javaid[1]
[1]University of Oxford, Botnar institute for Musculoskeletal Sciences- NDORMS, Oxford, United Kingdom
[2]Hospices Civils de Lyon, Reference center for fibrous dysplasia- Rheumatology department, Lyon, France

**Abstract Text**

**Introduction**: Pain is a common symptom in many rare bone disorders, often linked to depression and a substantial decline in quality of life. However, there is little information on the quality of the pain as this may provide insights into pain mechanisms.

**Purpose**: To describe the self-reported frequency and characteristics of pain in adults withFibrous Dysplasia of Bone/McCune-Albright Syndrome (FD/MAS), Osteogenesis Imperfecta (OI), and X-linked Hypophosphatemia (XLH).

**Methods**: A retrospective cross-sectional study was conducted using the online UK RUDY registry. Adults with FD/MAS, OI, and XLH who were registered in the UK-based RUDY registry and completed the painDETECT questionnaire (PD-Q) at baseline were included. Pain prevalence and the distribution of nociceptive, neuropathic-like, and unclear pain phenotypes were assessed based on baseline PD-Q results. Descriptive statistical analyses were performed using R® software.

**Results**: 281 adults completed the PD-Q at baseline : 94 patients with FD/MAS, 94 with OI, and 93 with XLH. Of these, 86% experienced pain currently with similar proportions across the FD/MAS, OI, and XLH groups ($p=0.07$). Furthermore, 47% described their strongest pain as severe (≥8/10) in the past four weeks, with no significant differences between the three conditions ($p=0.06$).

**Conclusion**: Despite differences in the mechanisms underlying these bone diseases, the prevalence of pain was consistently high, with nearly half of the participants reporting severe pain in the past four weeks. The pain phenotypes were also similar across the three conditions. Further work is needed to explore the distribution of pain and associated features that would be consistent with nociplastic pain.

**P257**

**Osteopetrosis and Spinal Lipomatosis: A Rare Case of Spinal Stenosis in Autosomal Dominant Osteopetrosis**

Kristina Linder Ekberg[1], Stavros Stergianos[1], Mikael Skorpil[2], Hanna Gry[2]
[1]Karolinska University Hospital- Solna, Endocrinology, Stockholm, Sweden
[2]Karolinska University Hospital- Solna, Neuroradiology, Stockholm, Sweden

**Abstract Text**

**Background:** Osteopetrosis is a rare hereditary disorder characterized by excessive densification of the skeleton due to dysfunction of osteoclasts. The result is dense but brittle bones that are prone tofractures. Mutation in the CLCN7 gene is the most common cause of osteopetrosis, leading to autosomal dominant osteopetrosis (ADO).
ADO is considered the mildest form of osteopetrosis and often presents with fractures, primarily in ribs and long bones, paranasal sinus problems but also extramedullary hematopoiesis due to bonemarrow compression by excessive bone.

**Case presentation:** A 61 year old male with hypertension, diabetes mellitus type 2, and osteopetrosis seeks primary care in winter 2020-2021 due to numbness in his feet and legs. Osteopetrosis due to a CLCN7 mutation is present in the family, affecting also his father, brother, and two of his three children.They have primarily suffered from rib and metatarsal fractures as well as paranasal sinus problems.

MRI of the spine 2021 showed an abnormal bone marrow signal, and CT revealed extensive sclerosis. Initially metastatic prostate cancer was suspected but PSA was normal. In 2022, his symptoms progressed proximally including problems with urination and defecation. MRI in April 2022 showed spinal stenosis with bone marrow compression due to lipomatosis and extramedullary hematopoiesis at levels Th4-Th5. Neurosurgeon recommended surgery, and laminectomy was performed in August 2022.

Histopathology analysis revealed lipomatosis with extramedullary hematopoiesis. Postoperatively, there was improvement in urinary and defecation function, but persistent back and leg pain.

**Conclusion:** This case highlights a rare presentation of spinal lipomatosis with extramedullary hematopoiesis in ADO, emphasizing the role of increased bone mass in constricting bone marrow hematopoiesis. Despite normal blood counts and spleen size, extramedullary hematopoiesis caused significant complications. The findings underscore the importance of multidisciplinary care involving specialized radiologists and endocrinologists to improve diagnostic accuracy and management of such rare conditions.

**P258**

**Serum D-2-Hydroxyglutarate: a non-invasive diagnostic tool for Maffucci syndrome over Ollier disease or other enchondromatosis**

*Victor Viala[1], Lauriane Goldwirt[2], Annouk Bisdorff-Bresson[3], Martine Cohen-Solal[4], Emmanuel Mandonnet[5], Thomas Funck-Brentano[6]*
*[1]Université Paris Cité, Rheumatology, Paris, France*
*[2]AP-HP and INSERM, Hôpital Saint-Louis  Department of Pharmacology, Paris, France*
*[3]AP-HP- Lariboisière Hospital,*
*Neuroradiology- Center of vascular anomalies clinic VASCERN VASCA European Reference Centre, Paris, France*
*[4]INSERM, U1132 Bioscar, Paris, France*
*[5]AP-HP, Neurosurgery- Lariboisière Hospital, Paris, France*

[6]Université Paris Cité & APHP, Rheumatology, Paris, France

**Abstract Text**

**Introduction:** Ollier disease (OD) and Maffucci syndrome (MS) are rare disorders characterized by multiple enchondromas, associated with veinous malformations in MS, caused by somatic mosaicism of Isocitrate deshydrogenase 1 and 2 (IDH1 and IDH2) pathogenic variants. OD-MS patients are at high risk of developing IDH1/2-mutant-related malignancies, justifying repeated whole-body screening. To date, no available non-invasive procedure is available to ascertain the molecular diagnosis to differentiate OD-MS from other enchondromatosis. IDH1/2 mutations harbor enzymatic activity to catalyze α-ketoglutarate to oncometabolite D-2-hydroxyglutarate (D-2HG). The aim of this preliminary study is to assess D-2HG in serum as a potential biomarker for the diagnosis of OD and MS.

**Material and methods:** In this monocentric cross-sectional study, we included consecutive OD-MS patients referred to our adult reference center with at least one serum quantification of D-2HG using LC-MS/MS. Past medical history and screening for malignancy was recorded at the inclusion visit. Participants were informed and provided their informed consent that data would be collected and analyzed for research purposes.

**Results**: Twenty-four patients were included with a median [min-max] of 33.9 [18.0-68.1] years, of which 11 patients with OD; 11 with MS; and 2 with other enchondromatosis. Ten patients (41.7%) had a history of mutant IDH-related cancer and 11 patients had a documented IDH1 mutation identified in affected tissues. Serum D-2HG was quantifiable in 10 of 11 MS patients and was below LOQ in other enchondromatosis and 10 of 11 OD. Levels of serum D-2HG were associated with the number of localizations ($R^2$=0.234) in MS.

**Discussion/conclusion**: These preliminary results show that measurement of serum D-2HG may be of diagnostic interest in patients with MS without requiring a biopsy. In MS, it may be associated with disease severity. Further evaluation is needed to assess the possible prognostic interest of D-2HG concentration to predict tumor risk.

**P259**

**Progression of spinal fibrous dysplasia lesions in adults**

*<u>Arnaud Vanjak</u>[1,2], Alice Bel[3], Caroline Guinot Sauvent[1], Amandine Laporte[4], Valérie Bousson[4], Pascal Bizot[5], Thomas Funck-Brentano[1,2]*
*[1]Lariboisière Hospital- APHP, Rheumatology, Paris, France*
*[2]CRMR Dysplasie fibreuse des os et syndrome de McCune Albright, Rheumatology, Paris, France*
*[3]La Milétrie Hospital, Rheumatology, Poitiers, France*
*[4]Lariboisière Hospital- APHP, Osteoarticular radiology, Paris, France*
*[5]Lariboisière Hospital- APHP, Orthopedic Surgery, Paris, France*

**Abstract Text**

Fibrous dysplasia (FD) lesions can enlarge during growth, in cases involving aneurysmal cystic transformation, in association with acromegaly, or during hormonal changes like pregnancy. In adults, structural changes suggest potential sarcomatous degeneration or coexisting malignant lesions. Routine imaging follow-up for adults' dysplastic lesions is generally not recommended, except in specific situations, such as scoliosis. [Javaid et al 2019].
We here present five adult cases of spinal FD who exhibited lesion progression, outside the aforementioned contexts.

Between October 2023 and December 2024, 135 patients consulted at our center; 13 patients (9.6%) with spinal involvement. Among them, five patients (38.5%) had a progression of FD spinal lesions, observed through CT scans performed on average 10.4 ± 2.9 years apart. Sixty percent are women with an average age of 53.4 years ± 1.1. Eighty percent have a polyostotic form, without McCune-Albright syndrome. The lumbar and cervical spine are affected in 40% each, while the thoracic spine is involved in 20%. Both men exhibit phosphate diabetes without hypophosphatemia. CTX levels average 429.5 pg/mL ± 121.7. Only one patient received bisphosphonates therapy, last administered in May 2019. Two underwent intervention due to spinal instability threatening nearby neurological structures: one at the lumbar spine via cementoplasty and one at the thoracic level via extended arthrodesis.
This series of five cases showing progression of spinal lesions of fibrous dysplasia in adults since 2023 prompts us to recommend CT monitoring for patients with axial skeletal dysplastic lesions. The monitoring frequency is not fully determined and should be adjusted based on lesion size and clinical symptoms. Structural progression screening in a larger patient population would help clarify the incidence of this phenomenon and identify potential risk factors.

**P260**

**Tofacitinib as a good therapeutic option for the managing fibrodysplasia ossificans progressiva**

*<u>Irina Nikishina</u>[1], Valeria Matkava[1], Svetlana Arsenyeva[1], Alia Arefieva[1], Emil Gasymov[2], Leonid Blank[2]*
*[1]V.A. Nasonova Scientific Research Institute of Rheumatology, Paediatric, Moscow, Russia C.I.S.*
*[2]V.A. Nasonova Scientific Research Institute of Rheumatology, Radiology, Moscow, Russia C.I.S.*

**Abstract Text**

**Introduction:** Fibrodysplasia ossificans progressiva(FOP) is an ultra-rare monogenic condition which caused by a mutation in the ACVR1 gene and leads to appearance of multiple heterotopic ossification(HO).It also has signs of «classic» rheumatic diseases(RD).Treatment management for these pts is a huge challenge.Oral rout of JAK-inhibitors is the most preferable in pts with contraindications to any tissue impairment.

**Purpose**:To analyze experience of target anti-inflammatory drug Tofacitinib(TOFA) and Palovarotene(PALO) in real clinical practice, evaluate efficacy and safety.

**Methods**:In 35 pts with severe course of FOP confirmed by ACVR1 gene mutation TOFA was administrated and evaluated.In 8 pts TOFA combined with PALO, a selective retinoic acid receptor gamma agonist.

**Results**:In all 35 pts(21 male/14 female)the diagnosis was verified by "classic" phenotype:malformed great toes-35/100%;malformed thumbs-11/31%;abnormalities of cervical spine,peripheral osteochondromas and multiple HO in 35/100%.Genetic test confirmed typical mutation(p.Arg206His) in 32 pts or other ultra-rare mutations(in 3 pts).We found a lot of similarities with RD such as large joints synovitis,signs of active sacroiliitis,patterns of bone ankylosis.All patients initially fell to treatment NSAIDs(35/100%),corticosteroids(oral–22/63%,intravenous–16/46%) and bisphosphonates(10/29%).The average age of disease onset was 3.7 years(0.5 to 16).The average age of initiation was 10.6 years(from 2 to 28 years).Duration of therapy is from 5 to 60 months.Amount of flares significantly decreased after treatment from average 10-12/year to 0-2 after 1-year treatment.In all 22 pts oral steroids were withdrawn.Tolerance was good,no AE were registered.8 pts received combination PALO and TOFA. All pts had different intensity adverse reactions connected with skin and depended on the palovarotene dosage.

**Conclusion**:long-term experience shows that TOFA is highly efficient,well-tolerated option for treatment of acute FOP stage which provide functional status improvement, decreasing amount of flares.It's possible to assume a synergism of the anti-inflammatory effect of TOFA and PALO for preventing the development of new ossifications.Combination PALO and TOFA can be considered as a new strategy but need to further investigations.

**ND-P027**

**Burosumab Therapy in Adult Patients with X-Linked Hypophosphatemia: A Three-Year Follow-Up**

*Adolfo Marco Perrotta[1], Silverio Rotondi[1], Lida Tartaglione[1], Sandro Mazzaferro[1]*
*[1]"Sapienza" University of Rome, Translational and Precision Medicine, Roma, Italy*

**Abstract Text**

**Background and Aims:** X-linked hypophosphatemic rickets (XLH) is due to mutations in PHEX gene, resulting in high FGF23 levels, renal phosphate loss, hypophosphatemia, low vitamin D, high alkaline phosphatase (ALP) and bone disorders. Conventional therapy with vitamin D and phosphate supplements is partial effective. Burosumab, a monoclonal antibody against FGF23, is a novel drug capable to normalize phosphatemia (Ps) and vitamin D levels in pediatric patients.

However, its effectiveness in adult patients with bone lesions remains unclear. This study aims to evaluate safety and efficacy on biochemical and clinical features of Burosumab in a cohort of adult XLH patients along three-year follow-up.

**Method:** We considered eligible 18 years patients or older, with a genetic diagnosis of XLH, previous naïve for burosumab and in standard therapy for three years. Biochemical parameters, as Ps, ALP, Tubular Maximum reabsorption of Phosphate on Glomerular Filtration Rate (TMP/GFR) were tested monthly before Burosumab administration. Clinical tests included the six-minute walk test, UP and Go Timed Test, and WOMAC Index performed every six months.

Burosumab was administered monthly at 1 mg/kg throughout the entire follow-up period.

**Results:** We enrolled five female with a median age 45±10 years and a median body weight of 60±10 kg. The average Burosumab dose was 60±10 mg/month. After therapy, there was a stable and statistically significant improvement of Ps, TMP/GFR, WOMAC Index, six- minute walk, and UP and Go Timed Test (table 1 and figure 1).

No significant adverse events were reported during the follow-up.

**Conclusion:** In adult XLH patients, Burosumab seems a safe therapy with a long lasting efficacy on Ps and TMP/GFR. ALP reach a normal range after three year of therapy, suggesting improvement of bone mineralization. Clinical tests show a significant benefit for the patients, improvement of strength and pain tests suggest significant clinical benefit for the patients.

Figure 1

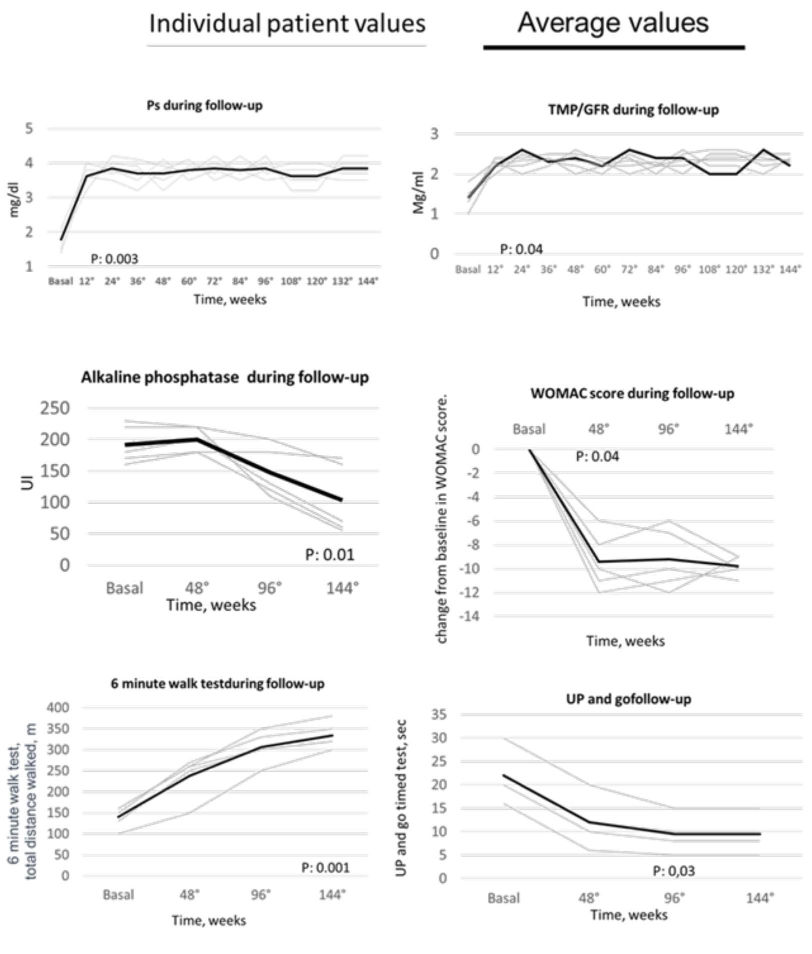

**ND-P028**

**Health-related quality of life in individuals with PLS3 genetic variants**

*Zografia Zervou[1], <u>Evert F.S. van Velsen</u>[1], M. Carola Zillikens[1]*
[1]*Erasmus MC Bone Center- Erasmus University Medical Center, Internal Medicine, Rotterdam, Netherlands*

**Abstract Text**

X-linked osteoporosis, due to *PLS3* genetic variants, is a rare disorder, marked by low bone mineral density (BMD) and early-onset fractures, predominantly affecting men. We evaluated the health-related quality of life (HRQoL), including sex-differences, in 24 individuals with *PLS3* variants (14 men and 10 women), utilizing four questionnaires: 36-Item Short Form (SF-36), EQ-5D-5L, Brief Pain Inventory (BPI) and Brief fatigue Inventory (BFI). Data from the SF-36 were compared with a reference Dutch population from literature. Individuals with *PLS3* variants demonstrated significant impairments in physical function (12.6 vs. 83, p<0.01), general health (51.5 vs. 70.7, p<0.016) and vitality (55.0 vs. 68.6, p=0.01) compared to healthy controls. Physical function was significantly correlated with the total number of fractures (p=0.038). Additionally, pain affected both sexes differently (prevalence in BPI: 58.4% men, 90.0% women), and there was interference with daily activities in a large number of patients (42.9% men, 30.0% women BPI). Furthermore, moderate fatigue impacts a significant proportion of men (58.3%) and women (40.0%). However, mental health appeared unaffected, evaluated by both the SF-36 (76.6 vs. 76.8, p=0.958) and EQ-5D-5L (84.6% men and 77.8% women no issues in the anxiety/depression domain). In this first study on HRQoL of patients with *PLS3* variants, a considerable number of both sexes experienced pain and fatigue. Results may motivate healthcare professionals to prioritize the physical component and fatigue aspect of HRQoL in these patients. Further prospective studies are essential to enlighten the effects of different types of treatment in the HRQoL of patients with *PLS3* variants.

**Poster Sessions: Innovative concepts, new technologies, case reports**

**P261**

**Three-dimensional Volumetric Assessment of Fracture Healing after Open Repair of Distal Radius Fracture and Immediate Use of Antiresorptive Agents**

*Ji Sup Hwang*[1], *Hyun Sik Gong*[2]
[1]*Seoul National University Hospital, Orthopedic Surgery, Seoul, Korea- South*
[2]*Seoul National University Bundang Hospital, Orthopedic Surgery, Seongnam, Korea- South*

**Abstract Text**

**Purpose:** Although anti-resorptive agents did not affect fracture healing in previous studies, the amount of healing was not examined quantitatively. Thus, the purpose of this study was to examine the amount of healing using three-dimensional (3D) volumetric assessment after distal radius fracture (DRF) in patients who started anti-resorptive agents.

**Methods:** A total of 74 patients who underwent surgery for DRF and started denosumab (n = 46) or risedronate (n = 28) immediately after surgery were retrospectively reviewed. At a mean of 6.3 weeks postoperatively, fracture healing was assessed with plain radiographs and CT scans. CT images were analyzed using an imaging software for 3D assessment of cortical volume at fracture site (Figure 1).

Volumetric fracture healing ratio defined as healing to healed cortical bone volume ratio was calculated and correlated with radiographic healing, demographic characteristics, and osteoporosis-related parameters. We also compared this ratio between the two medication groups after controlling for BMD.

**Results:** Volumetric fracture healing ratio showed positive correlations with radiographic healing (R = 0.400, $p < 0.01$), lumbar BMD (R = 0.271, $p = 0.02$), and femur neck BMD (R = 0.231, $p = 0.05$). When BMD values were adjusted, denosumab users showed a significantly higher healing ratio than risedronate users (85.9 ± 1.4 % vs. 81.0 ± 1.8 %; $p = 0.04$).

**Conclusion:** 3D assessment of fracture healing indicated that one's osteoporosis status was related to the amount of healing after DRF. This study further confirms that starting anti-resorptive therapy does not impede fracture healing, with denosumab showing a higher healing ratio than risedronate.

Figure 1. Three-dimensional cortical bone model of distal radius was obtained. The left image represents the healing cortical bone of the distal radius, and the right image healed cortical bone. Ratio of the volume of each model was defined as fracture healing ratio.

**P262**

### A rare case of forearm ectomesenchymoma in a pediatric patient

*Aleksandra Osińska[1], Michalina Boruch[1], Marta Druszcz[1], Andrzej Materniak[2], Magdalena Woźniak[2]*
*[1]Medical University of Lublin, Student Scientific Society at the Department of Pediatric Radiology, Lublin, Poland*
*[2]Medical University of Lublin, Department of Pediatric Radiology, Lublin, Poland*

### Abstract Text

**Introduction:** Malignant ectomesenchymoma (MEM) is an uncommon and rapidly progressing malignant tumor affecting the nervous system or soft tissues, composed of both mesenchymal and neuroectodermal components. It primarily occurs in pediatric patients, with the pelvic region, retroperitoneal area, and genitourinary system being the most frequently affected primary sites. Current literature cites approximately 70 documented cases of MEM globally.

**Case discussion:** A 22-month-old child was hospitalized in the Pediatric Hematology and Oncology Department for a follow-up ultrasound of the soft tissues and related laboratory evaluations. During the consultation, the parents reported observing a painless nodular mass on the right forearm. An ultrasound examination revealed an intramuscular, polycyclic, well-defined, and heterogeneous lesion measuring 57x20x27mm on the palmar side of the forearm, with a chaotic blood flow pattern observed in the Color Doppler assessment. The sonographic findings were inconclusive and raised suspicion for a neoplastic condition. A biopsy was conducted, and histopathological analysis confirmed the presence of ectomesenchymoma. The patient was classified for chemotherapy for non-RMS-like conditions following the CWS - VAIA III protocol and initial staging categorized as HRG group; IRS III, N1.

**Conclusions**: Due to the rarity of MEM and its heterogeneous nature, making a diagnosis is difficult. This results in a prolonged diagnostic and therapeutic process. In the presented case, the ultrasound findings indicated malignant features, including a subfascial position and a lesion size exceeding 5 cm. Furthermore, the irregular vessel morphology, including trifurcations and a disorganized, tortuous distribution of vessels with avascular areas likely indicating necrosis, suggested a chaotic vascular architecture. Surgical intervention remains the primary treatment strategy. The use of chemotherapy and radiotherapy is generally discouraged in children due to their potential adverse effects on neurodevelopment.
A deeper insight into the tumorigenesis of MEM may aid in identifying more suitable systemic treatments within current therapeutic frameworks.

**P263**

**Prevalence of sagittal malalignment and its measurement reliability in total body radiographic images**

*Kirsty Huininga*[1], *Fjorda Koromani*[1], *Rivadeneira Fernando*[1]
[1]*Erasmus MC, Internal Medicine, Rotterdam, Netherlands*

**Abstract Text**

Sagittal balance (SB) is altered with ageing, often resulting in sagittal malalignment (SM). The latter can manifest as hidden imbalance, imbalance with compensation, or imbalance without compensation; and is hypothesized to be associated with degenerative changes, falling and increased fracture risk. We aimed to assess the prevalence of sagittal malalignment and reliability of its measurement in a sample of participants from a population-based cohort study.

Methods: Two readers assessed 59 total body images scanned in standing position with an EOSEdge system and scored Sacral Vertical Axis (SVA), Pelvic Incidence (PI), Lumbar Lordosis (LL), Pelvic Tilt (PT), Thoracic Kyphosis (TK) and SM scored on a sterEOS workstation. Hidden imbalance was defined as SVA 50mm and PI-LL >10°, imbalance with compensation as SVA >50mm and PT>20 or TK>-30, and imbalance without compensation as SVA >50mm and PT≤20° and ≤-30°). Interclass correlation coefficient (ICC) and Cohen's Kappa were calculated to estimate agreement between the two readers.

Results: Mean age of participants was 71.8 (SD 6.2) years of which 49% women. The balance compensated group was predominantly male and older (p=<0.05). Some degree of hidden or compensated imbalance was observed in 69.5% of participants (Table 1). ICC for SVA, PI, LL, PT and TK were good to excellent (0.83-0.92). Classification agreement was moderate for balanced (κ =0.52), and hidden imbalance (κ =0.61); and high for compensated imbalance (κ =1.00), all p-values <0.001. No individuals were scored as having severe imbalance without compensation.

Conclusions: Sagittal malalignment can be reliably measured although scoring hidden imbalance may be challenging. Overall, hidden and compensated imbalance is prevalent in the general population, predominately in elderly men, while uncompensated imbalance is expected to be more prevalent in individuals with severe conditions. Follow-up on a larger sample will be done for further validation and to explore implications for falling and fracture risk.

| /Rater 2<br>/Rater 1 | Balanced | Imbalance hidden | Imbalance compensated | Imbalance uncompensated | TOTAL |
|---|---|---|---|---|---|
| Balanced | 14 | 2 | 2 | 0 | 18 |
| Imbalance hidden | 4 | 20 | 0 | 0 | 24 |
| Imbalance compensated | 0 | 1 | 16 | 0 | 17 |
| Imbalance uncompensated | 0 | 0 | 0 | 0 | 0 |
| TOTAL | 18 | 23 | 18 | 0 | 59 |

**Table 1: Cross-Tabulation of Classification Categories Between Rater 1 and 2**

**P264**

**Basic and clinical scientists working together? Do we make the best of both worlds?! From the basic- and clinical action groups of ECTS**

*Willem Lems[1], Athanasios Anastasilakis[2], Christina Møller Andreasen[3], Julien Paccou[4], Tim Rolvien[5], Michaela Tencerova[6], Jan Tuckermann[7], Maria Yavropoulou[8], Kent Søe[9]*
*[1]AmsterdamUMC, Rheumatology, Amsterdam, Netherlands*
*[2]general miltary training hospital -, Endocrinology, athens, Greece*
*[3]University of Southern Denmark- Odense- Denmark, Pathology, Odense, Denmark*
*[4]University of Lille- Lille University Hospital, Rheumatology, Lille, France*
*[5]University Medical Center Hamburg-Eppendorf, Department of Trauma and Orthopaedic Surgery, Hamburg, Germany*
*[6]Institute of Physiology of the Czech Academy of Sciences,*
*Laboratory of Molecular Physiology of Bone, Prague, Czech Republic*
*[7]Institute of Comparative Molecular Endocrinology, Ulm University, Ulm, Germany*
*[8]University of Athens, Department of Internal Medicine- Endocrinology, Athens, Greece*
*[9]Odense University, Pathology, Odense, Denmark*

**Abstract Text**

**Background:** Translational research is of often far from optimal, also in the field of musculoskeletal disorders, highlighting the need for stronger collaboration between basic and clinical scientists.

**Methods/results:** Members of the basic and clinical action groups of the European Calcified Tissue Society (ECTS) and endorsed by the Board of the ECTS, examines the key barriers to effective translational research in musculoskeletal diseases, including clinician workload, differences in professional language and culture, physical distance between research sites, and insufficient interdisciplinary funding. Through interviews with eight institutional managers across five European countries, we observed that in some institutions the collaboration between basic scientists and clinicians was regarded as no concern (but with room for improvement), and in most institutions it was recognized as a serious issue. We found consensus on the importance of collaboration yet identified discrepancies in the provision of structural and financial support.

**Discussion**: Based on these findings, we propose strategic initiatives to bridge the gap between basic and clinical research. Suggested measures include dedicatedtranslational funding, integrated research facilities, collaborative scientific forums, strategic collaborations, establishment of physician-scientists, and, finally, bringing basic and clinical researchers together in the same building or even in a combined department. Notable successes, such as the development of the anti-osteoporotic drugs, romosozumab and denosumab, underscore the value of a coordinated approach and exemplify how shared insights between laboratory research and clinical practice can lead to impactful therapeutic advances. Moving forward, we advocate for institutional commitments to foster a robust translational research environment, awareness in the respective study curricula of medicine and basic scientific subjects, as well as tailored funding initiatives to support such efforts.

**Conclusion:** This abstract serves as a call for discussion within ECTS and other organisations to enhance interdisciplinary cooperation to advance musculoskeletal medicine and improve outcomes for patients with musculoskeletal diseases.

**P265**

**Humanized Bone Niche-Based In Vitro Models for Primary Bone Tumour Research**

*Tina Frankenbach[1], Natalia Cabeza-Boeddinghaus[1], Aarushi Davesar[1], Anna Litovskikh[1], Giles M Cheers[1], Sandy Walter[1], Hans Roland Dürr[2], Alexander Klein[2], Thomas Knösel[3], Dietmar W Hutmacher[4,5], Boris M Holzapfel[1], Susanne Mayer-Wagner[1]*
*[1]LMU University Hospital- LMU Munich,*
*Department of Orthopaedics and Trauma Surgery- Musculoskeletal UniversityCenter Munich MUM, Munich, Germany*
*[2]LMU University Hospital- LMU Munich,*
*Department of Orthopaedics and Trauma Surgery- Orthopaedic Oncology- Musculoskeletal University Center Munich MUM, Munich, Germany*
*[3]Ludwig-Maximilians-Universität LMU Munich, Institute of Pathology, Munich, Germany*
*[4]Queensland University of Technology QUT,*
*Centre for Biomedical Technologies- School of Medical- Mechanical and Process Engineering, Brisbane, Australia*
*[5]Queensland University of Technology QUT,*
*Max Planck Queensland Center for the Materials Science of Extracellular Matrices, Brisbane, Australia*

**Abstract Text**

**Background:** Malignant bone tumours, such as osteosarcoma, Ewing's sarcoma, and chondrosarcoma, present significant health challenges, particularly for adolescents and young adults. To improve treatment outcomes, innovative preclinical models are essential for (a) understanding patient- and tumour-specific mechanisms, and (b) enabling personalized drug trials.

**Purpose:** State-of-the-art preclinical models incorporating both the bone microenvironment and patient-specific tumour components are crucial for bridging the gap to clinical applications. To address this, we developed a novel *in vitro* model combining fresh patient-derived tumour tissue with a tissue-engineered bone niche, designed to enhance tumour understanding and drug testing.

**Methods:** Our model integrates tumour tissue from resection surgery after informed consent was given (n=4 osteosarcoma, n=2 Ewing's sarcoma, n=2 chondrosarcoma) with a tissue-engineered bone construct (TEBC) made of osteoprogenitor cells seeded onto 3D-printed mPCL scaffolds coated with calcium phosphate. The tumour tissue and TEBC are bonded with fibrin glue, forming tumor-TEBC constructs. After a 3-week *in vitro* culture period, we assessed the co-constructs using live-dead fluorescence imaging, micro-CT, and histological analysis.

**Results:** The TEBCs mimic key bone characteristics, such as osteoblast-driven mineralization and bone extracellular matrix. The tumour-TEBC model supports tumour proliferation and maintains original tumour features. Histological analysis shows tissue interaction via the extracellular matrix at the contact points, highlighting the merging of tumour and bone tissues.

**Conclusion:** In conclusion, this advanced bone tumour model, incorporating both patient-specific tumour tissue and a human bone component, will improve the reliability of *in vitro* drug testing and provide valuable insights for personalized bone tumour care.

**P266**

### Ultrasound stimulation of a piezoelectric nanocomposite hydrogel and adipose derived mesenchymal stromal cells for osteoarthritis: in vivo efficacy studies

*Giorgia Codispoti*[1], *Melania Carniato*[1], *Lucia Martini*[1], *Milena Fini*[2], *Gianluca Giavaresi*[1], *Cristina Manferdini*[3], *Gina Lisignoli*[3], *Leonardo Ricotti*[4], *Matilde Tschon*[1]
[1]*Rizzoli Orthopaedic Institute, Complex Structure of Surgical Sciences and Technologies, Bologna, Italy*
[2]*Rizzoli Orthopaedic Institute, Scientific Director, Bologna, Italy*
[3]*Rizzoli Orthopaedic Institute, Laboratory of Immunoreumatology and Tissue Regeneration, Bologna, Italy*
[4]*The BioRobotics Institute- Scuola Superiore Sant'Anna, Department of Excellence in Robotics & AI, Pisa, Italy*

### Abstract Text

**Background/Introduction:** The *in vivo* studies aimed to assess the efficacy of a personalized therapy for mild osteoarthritis (OA) using a nanocomposite hydrogel enriched with smart nanomaterials and autologous adipose tissue-derived stem cells (ASCs) to be responsive to low intensity pulsed ultrasound (LIPUS), and stimulating chondrogenesis.

**Purpose:** To prove that intra-articular injection of the smart hydrogel can slow down the progression of OA.

**Methods:** Knee OA was induced bilaterally in rabbits and sheep by anterior cruciate ligament transection and lateral meniscectomy, respectively. Male and female rabbits received different i.a.

treatments (Hydrogel+ASCs+LIPUS, Hydrogel+ASCs, LIPUS, or No treatment), while each sheep received Hydrogel+ASCs+LIPUS in the left knee in comparison with the untreated right knee. ASCs were isolated from rabbit infrascapular tissue and sheep infrapatellar fat pad. LIPUS was applied in five sessions over ten days. Macroscopic and microscopic OA grading, histomorphometric analyses, and local inflammatory response were assessed at 1 and 3 months after treatments for rabbits and after 3 months for sheep.

**Results:** Macroscopic analysis revealed improved cartilage integrity in Hydrogel+ASCs+LIPUS male and female rabbits at 3 months post-treatments; better OARSI score in Hydrogel+ASCs+LIPUS was measured in females than males at 1 month (medial femoral condyle $p=0.006$; lateral femoral condyle $p=0.011$. Generalized Linear Model, $p<0.01$). No significant differences were found in histomorphometric parameters. Knees treated with Hydrogel+ASCs+LIPUS ($p = 0.007$; GLM, $p<0.01$) or Hydrogel+ASCs ($p < 0.0005$; GLM, $p<0.01$) showed a stronger inflammatory response in females than males. In sheep, joints treated with Hydrogel+ASCs+LIPUS showed better OARSI scores ($p=0.002$; GLM, $p<0.01$) and reduced tenosynovitis observed by ultrasonographic analysis.

**Conclusion:** The multi-approach treatment slowed the OA progression in male and female rabbits with better results in females, demonstrating the efficacy of personalized OA treatment.

**P267**

**MODA project: randomized controlled trial to evaluate the decellularized human dermis in combination with orthobiologic stimuli for the arthroscopic augmentation of massive rotator cuff tears**

*Matilde Tschon*[1]*, Marco Cavallo*[2]*, Giorgia Codispoti*[1]*, Valentina Brunello*[2]*, Enrico Guerra*[2]
[1]*IRCCS Istituto Ortopedico Rizzoli, Surgical Sciences and Technologies, Bologna, Italy*
[2]*IRCCS Istituto ortopedico Rizzoli, Shoulder and Elbow Surgery, Bologna, Italy*

**Abstract Text**

**Introduction:** Rotator cuff (RC) injuries are very common and characterized by retear rates up to 90%, higher in young people.

**Purpose**: The aim of the study is to evaluate the therapeutic efficacy of decellularized human dermis (ADM) alone or in combination with orthobiologic stimuli, such as bone marrow concentrate and subacromial bursa.

**Methods**: The randomized controlled clinical trial involves arthroscopic repair of the RC in large to massive lesions: patients were randomly treated with ADM (group A) or ADM enriched with autologous orthobiologic stimuli (group B: bone marrow concentrate from humeral head and subacromial bursa).

The retear rate, VAS and clinical and functional scores were evaluated before surgery and at different Follow Up (1, 3, 6 and 12 months).

**Results**: At present, 43 patients (mean age 53.35 ± 6.71) were included in the study and treated. At 6-month follow-up, 17 patients were evaluated: Group A (n=9) reported a CONSTANT-MURLEY of 73. 78 ± 14.32, VAS: 2.39 ± 2.96 and DASH: 65.89 ± 27.62, while Group B (n=8) CONSTANT-MURLEY: 72.50 ± 18.45, VAS: 3.25 ± 3.74 and DASH: 70.63 ± 35.61. All patients at 6 months resumed normal activities of daily living, 15 patients resumed extensive shoulder mobility, while two cases (one in group A and one in group B) at 6 months still had limited ROM in abduction and the persistence of post-surgical adhesive capsulitis.

**Conclusions**: Preliminary short-term results showed that the surgical technique does not lead to an increase in postoperative complications, allowing recovery similar to standard arthroscopic cuff repair. Follow up at 6 months showed a good functional outcome, with no substantial differences between Group A and B.

**P268**

**The MODA project: in vitro comparison of mesenchimal stromal cell potentials derived from humeral bone marrow concentrate and subacromial bursa**

*Matilde Tschon*[1]*, Giorgia Codispoti*[1]*, Melania Carniato*[1]*, Valentina Brunello*[2]*, Stefania Pagani*[1]*,*
*Marco Cavallo*[3]*, Enrico Guerra*[2]
[1]*IRCCS Istituto Ortopedico Rizzoli, Surgical Sciences and Technologies, Bologna, Italy*
[2]*IRCCS Istituto Ortopedico Rizzoli, Shoulder and Elbow Surgery, Bologna, Italy*
[3]*IRCCS Istuto Ortopedico Rizzoli, Shoulder and Elbow Surgery, Bologna, Italy*

**Abstract Text**

**Introduction:** The treatment of massive rotator cuff (RC) injuries is a challenging goal due to poor tendon quality, hypovascularization, and biomechanically incompetent scar formation. In recent years, orthobiologic products have been proposed to improve RC healing in addition to surgical techniques. The proximal humerus has been shown to be a possible source of mesenchymal as well as the subacromial bursa rich in mesenchymal cells and growth factors that promote vascularization.

**Purpose:** The aim of this research is the in vitro biological and comparative characterization of subacromial bursa and humeral bone marrow concentrate (cHBM), harvested from patients as surgical waste materials.

**Methods:** From the operating room, cHBM and subacromial bursa samples are cultured for isolation, expansion and characterization of the mesenchymal stromal cells they contain. The mesenchymal potential is analyzed by quantification of colony forming units (CFUs), expression of membrane CD markers, proliferation and trilineages differentiation analyses.

**Results:** The cHBM and subacromial bursa samples proved to be excellent cell sources of mesenchymal stromal cells. All cultures, both of cHBM and bursal tissue, differentiated in osteogenic, chondrogenic and adipogenic tissues with high cell yield, proliferation indices, membrane CD markers and CFU-forming capacity. Histological analysis conducted on the subacromial bursa revealed an extremely vascularized tissue.

**Conclusions:** The cHBM and subacromial bursa represent an optimal source of mesenchymal stromal cells, to make them easily harvested orthobiologic stimuli that can be used in the repair of even massive RC lesions.

**P269**

**Comparison of two mathematic models for assessment of biological bone age**

*Nataliia Grygorieva*[1], *Anatoly Pisaruk*[2], *Vitaly Gurianov*[3], *Anna Musiienko*[1]
[1]*D.F. Chebotarev Institute of Gerontology of the National Academy of Medical Sciences of Ukraine, Department of Clinical Physiology and pathology of the musculoskeletal system, Kyiv, Ukraine*
[2]*D.F. Chebotarev Institute of Gerontology of the National Academy of Medical Sciences of Ukraine, Laboratory of mathematical modeling of ageing processes, Kyiv, Ukraine*
[3]*O.O. Bogomolets National Medical University, Department of health care management, Kyiv, Ukraine*

**Abstract Text**

**Background:** Biological bone age (BBA) modelling is a fascinating area of research for assessing bone health. Effective BBA models can be helpful for screening osteoporosis and improving the accessibility of medical care.

**Objective:** This study aimed to evaluate two different mathematic methodologies (stepwise multiple linear regression (MLR) and neural network (NN) analysis) for modelling BBA.

**Materials and Methods:** We conducted a retrospective analysis on data from 3,071 healthy women aged 40-90. We included anthropometric data, dual-energy X-ray absorptiometry indices, and FRAX-based 10-year probabilities of major osteoporotic fractures (MOFs) and hip fractures (HFs). BBA models were developed using MLR and a multilayer perceptron NN.

**Results:** As a result of the calculation, we received the MLR formula, which incorporated variables such as bone mineral density (BMD) from various sites of the skeleton (lumbar spine (LS), ultradistal radius (UDR), minimal femoral neck (FN) or hip (H)), Trabecular Bone Score, and FRAX indices (Y = 92.22 + 29.57*FRAX HFs/FRAX MOFs + 15.91*LS BMD – 37.67 X*UDR BMD – 16.47*FN BMD + 8.01*H BMD – 19.74*TBS.

Assessment of this formula demonstrated a strong correlation between BBA and chronological age (R=0.77, p<0.00001) with a mean error of less than 4.9 years. In contrast, the NN model, designed with 6 input variables and 1 internal layer of 7 neurons, demonstrated better results. The correlation between BBA and chronological age was significantly higher (R=0.88, p<0.000001), and the mean error was reduced to less than 3.7 years.

**Conclusions:** While NN analysis provided greater accuracy, its implementation requires specialized software and computational resources. The MLR formula, while less precise, offers simplicity and ease of use without additional technological demands. This comparison underscores the potential of advanced machine learning techniques like NN for BBA modelling while highlighting the practicality of regression-based methods for widespread clinical adoption.

**P270**

### Busselton Healthy Ageing Study Hip DEXA scan to assess Kellgren Lawrence Hip OA Grading and DEXA scores in identifying progression to Total Hip Replacement.

*Charles Inderjeeth*[1], *Kun Zhu*[2], *Diren Che Inderjeeth*[3], *Kevin Murray*[4], *Michael Hunter*[5], *Jennie Hui*[5]
[1]*Sir Charles Gardner Hospital, Geriatric- Acute and Rehabilitation Medicine, Nedlands, Australia*
[2]*Sir Charles Gairdner Osborne Park Health Care Group, Department of Endocrinology, Perth, Australia*
[3]*Sir Charles Gairdner Osborne Park Health Care Group, Geriatric- Acute and Rehabilitation Medicine, Nedlands, Australia*
[4]*University of Western Australia, School of Population and Global Health, Perth, Australia*
[5]*University of Western Australia,*
*School of Population and Global Health- Population and Public Health, Perth, Australia*

**Abstract Text**

**Background:** Osteoporosis and hip osteoarthritis (HipOA)/ hip replacement (THR) often coexist. Hip DEXA provides an opportunity to screen patients for HipOA. In The Busselton Healthy Ageing Study, baseline May 2010 and December 2015), 5107 participants (80% of those eligible) were recruited. Baseline bone density (BMD) of both hips was measured. This study aims to identify factors associated with hip replacement.

**Methods:** Total 145 patients had THR. This cohort was matched with 445 (no THR). Kellgren-Lawrence (KL) HipOA grading was performed (grades 0 to 4). Groups were compared at baseline (means and SD). Correlations between KL grades, BMD, and hip replacement status were assessed using Pearson's correlation coefficient and multivariate logistic regression analysis.

**Results:** 590 patients (59.15% male; mean age 59.95±5.16 years, range 47.1-68.9). Patients with THR had significantly higher baseline Neck of Femur BMD (1.005 vs 0.941 g/cm2, p<0.001) and higher baseline OA grades (Higher Grade: 1.63 vs 0.93, p<0.001; Composite score: 2.79 vs 1.54, p<0.001). Males had higher OA grades compared to females (Composite score: 1.99 vs 1.65; Higher Grade: 1.17 vs 1.00). The correlation between OA grade and BMD was stronger in females (right hip: r=0.175) than males (r=0.078). The combination of high KL grade (≥2) and low neck of femur BMD (≤0.8 $g/cm^2$) was associated with a 4.5-fold increased risk of hip replacement (OR 4.50, 95% CI 2.61-7.75, p<0.001).

**Conclusions:** This study identifies combined KL grade and BMD as significant risk factors for hip replacement in middle-aged Australians. Predictive models incorporating both scores on DEXA could help identify high-risk patients, enabling targeted interventions to delay or prevent the need for hip replacement. Future research should focus on developing and validating such models, as well as evaluating the effectiveness of early intervention strategies in reducing hip replacement risk based on baseline DEXA scans.

**Ethics:** University of Western Australia

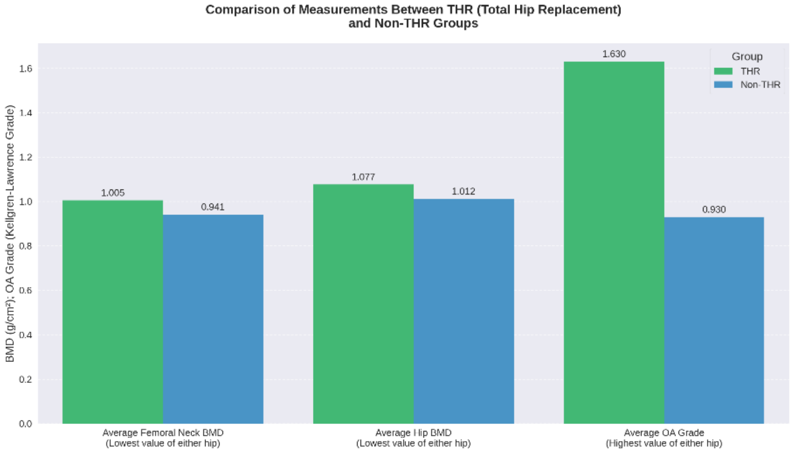

**P271**

**Unravelling the microvasculature of human cortical bone: a concept for a comparative study between microcomputed tomography and histopathology**

*Steven Van Offel*[1], *Bruno Lapauw*[1], *Charlotte Verroken*[1]
[1]*Ghent University Hospital, Endocrinology, Ghent, Belgium*

**Abstract Text**

**Background**: Microangiopathy of the cortical bone has frequently been suggested to contribute to the skeletal fragility in patients with diabetes. However, so far this has not been solidly proven. Histopathological studies are technically challenging and give only 2D information which does not allow the visualization of the complex 3D structure of the microvascular bed.

**Objective**: To find a method to quantify the microvasculature in human cortical bone. If this proposed pilot study delivers favourable results, we want to set up a larger study comparing the bone tissue of diabetes patients with healthy controls.

**Methods**: Bone samples are collected from the surgical waste material of patients undergoing hip replacement surgery. From the femoral neck, a cylinder with a diameter of 5 mm is drilled out and stored on ethanol 70% until further analysis using µCT (SkyScan 1273, Bruker). We would like to discuss the optimal histopathological methodology during the conference.

**Results**: We present the µCT images of an 18y-old man. The network of Haversian canals can easily be appreciated in the 3D reconstructions. However, these µCT images do not allow the direct visualization of capillaries. Therefore, the distinction cannot be made between canals containing a viable capillary and those without. Interestingly, distinctive differences in grayscale exist in the surrounding bone tissue, suggestive of a varying degree of maturation of the respective osteons.

**Discussion/conclusion**: µCT allows to visualize the canalicular structure of human cortical bone in 3D, but not the direct presence of capillaries. If correlation with 2D histology (including immunohistochemistry with anti-CD31 and anti-endomucin) would confirm that the canals within hypodense osteons contain viable capillaries, these gray scale differences in µCT might be used as a proxy for the presence of a viable capillary allowing bone turnover.

**P272**

**Digital experiences of Fracture Liaison Services (FLS) key performance indicators and treatment recommendations.**

*Muhammad Kassim Javaid*[1], *Naveed Dogar*[2], *John Chelsom*[3]
[1]*University of Oxford,*
*Nuffield Department of Orthopaedics Rheumatology and Musculoskeletal Sciences NDORMS, Oxford, United Kingdom*
[2]*University of Oxford,*
*Nuffield Department of Orthopaedics- Rheumatology and Musculoskeletal Sciences NDORMS, Oxford, United Kingdom*
[3]*Seven Informatics, Seven Informatics, Oxford, United Kingdom*

**Abstract Text**

**Background:** FLS need to aggregate clinical data, DXA and laboratory results, treatment recommendations and monitoring outcomes. This process can be enhanced using digital tools.

**Purpose:** To describe the experiences of working in an FLS from a digital perspective.

**Methods:** A literature review analysed the different assessment criteria for digital health records usability. The review domains were used to develop an online survey for FLS practitioners to describe their experience of using digital tools as part of their FLS work. The survey was circulated to the FLS networks in the UK and internationally.

**Results:** 48 responses were received from 10 FLSs in the UK and 12 FLSs from other countries, this included 30 FLS practitioners, 6 administrators and 10 consultants. Only 3 FLSs exclusively used their hospital record system, with excel the most common digital tool used (45.7%). The most time consuming aspects were patient identification, assessment and monitoring. 17 (35%) agreed their local FLS database increased their productivity, 15 (31.3%) were neutral and 17 (35%) disagreed, with no significant differences between international/UK FLSs or respondents' role ($p>0.1$). 2 FLS had multiple respondents (n=6,7) and the reported productivity by respondents varied from "Strongly disagree" (mainly nurses) to "Strongly Agree" (mainly administrators). The highest scoring ideal features for an ideal local FLS database were accuracy of data, patient privacy, security, interoperability and reducing administrative tasks. The top 4 desired new features were importing patient information, generating letters, monitoring reminders and importing DXA/laboratory results.

**Conclusion:** While FLS practitioners can identify the key features needed for an effective local FLS database, many FLS practitioners (35%) felt their local systems did not enhance their productivity, with observed variation within an FLS, indicating a need to improve the digital support provided to FLSs.

Image:

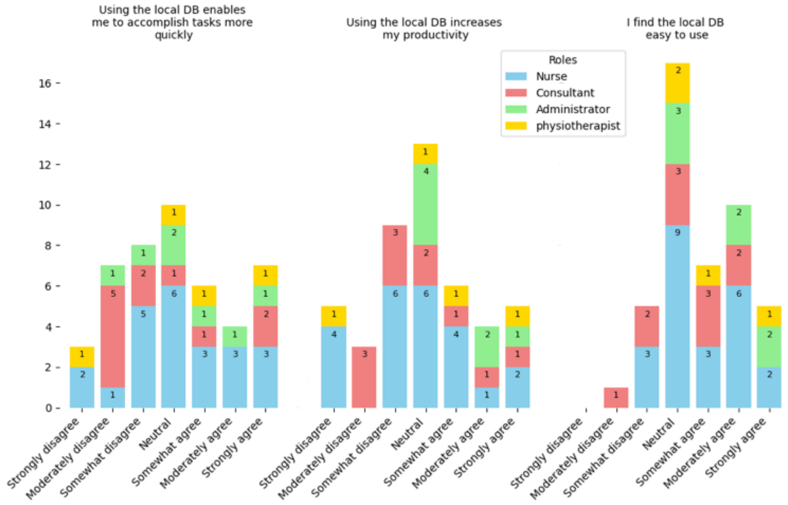

Productivity and usability reported by various roles

**P273**

**Cortical Bone Ultrasound Backscatter Spectroscopy is Superior to Bone Mineral Density in Detecting Fragility Fractures in in Women and Men**

*Kay Raum*[1], *Edgar Wiebe*[2], *Gabriele Armbrecht*[3]
[1]*Charité-Universitätsmedizin Berlin, Center for Biomedicine, Berlin, Germany*
[2]*Charité-Universitätsmedizin Berlin, Department of Rheumatology and Clinical Immunology, Berlin, Germany*
[3]*Charité-Universitätsmedizin Berlin, Department of Radiology, Berlin, Germany*

**Abstract Text**

Cortical ultrasound spectroscopy (CortBS) is an innovative non-ionizing imaging method that measures viscoelastic properties and intracortical pore morphology in the tibia. A pilot study in postmenopausal women demonstrated that CortBS has superior fracture discrimination compared to dual-energy X-ray absorptiometry (DXA). This study aims to evaluate the effectiveness of CortBS in distinguishing fractured (Fx) from non-fractured (nFx) in both male (M) and female patients (W), as well as in young healthy volunteers (C), and to compare its performance with that of DXA.

The study included 144 women and 33 men, aged 30 to 86 years. DXA measurements were taken at the lumbar spine and both femoral sites, while CortBS measurements were conducted on the anteromedial region of the tibia using a medical ultrasound scanner with custom software. Multivariate analysis of variance assessed the influence of gender, age, and anthropometric (AP) information (height, weight, BMI) on CortBS and DXA results. Partial least squares (PLS) discriminant analyses with cross-validation compared the effectiveness of the two methods in detecting vertebral, non-vertebral, and any fragility fractures, accounting for gender, age, and AP information.

The CortBS score was influenced by age, sex and prevalent fractures. CortBS and DXA scores were both reduced in patients with fractures, but the relative impact of fractures was higher on the CortBS score (Fig. 1). CortBS outperformed DXA in fracture discrimination both vertebral and non-vertebral fractures ($0.60 \leq AUC_{DXA} \leq 0.63$, $0.68 \leq AUC_{CortBS} < 0.74$). Regardless of gender, patients with fractures (Fx) showed an elevated fracture risk in the ultrasound score, but not in the DXA T-score (Fig. 1).

CortBS reflects gender specific alterations of pore density, morphology and viscolelastic attenuation during normal ageing and is suitable for discriminating prevalent fractures in women and men.

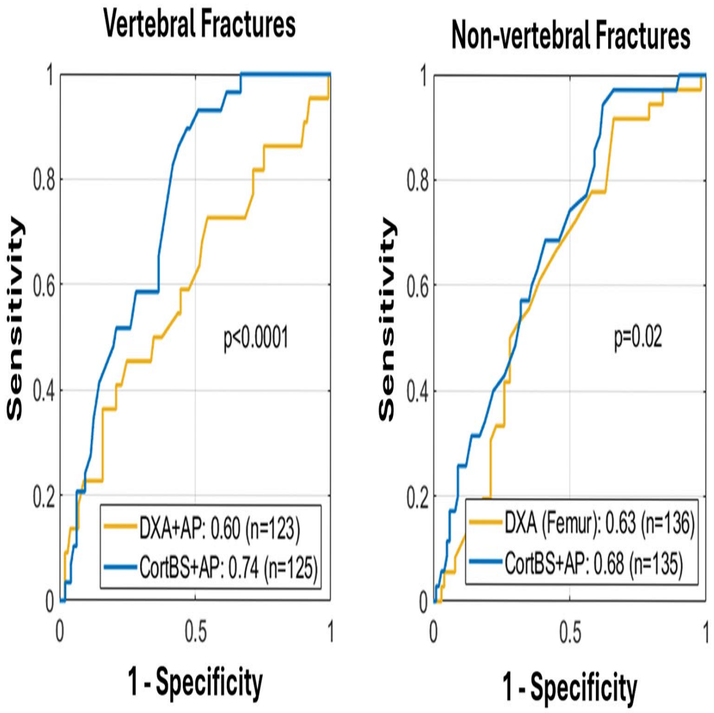

**P274**

**Delivering romosozumab for high fracture risk patients in the Fracture Liaison Service (FLS) setting: service modifications and early results**

*Claudia Maushart*[1], *Rachel Eckert*[2], *Sarah Connacher*[2], *Muhammad Kassim Javaid*[1]
[1]*University of Oxford,*
*Nuffield Department of Orthopaedics- Rheumatology and Musculoskeletal Sciences, Oxford, United Kingdom*
[2]*Oxford University Hospital, Metabolic Bone Unit, Oxford, United Kingdom*

**Abstract Text**

**Background:** The UK guidelines recommend first line romosozumab for postmenopausal people with a recent hip, vertebral, humerus or wrist fracture and a BMD T-score ≤-3.5 or T-score ≤-2.5 with a VF within 24 months, a history of ≥2 osteoporotic VFs, or a very high FRAX-based fracture risk. These patients are routinely seen by FLS where anabolic treatment usually requires referral to the specialist bone clinic.

**Purpose:** Describing modifications in a local FLS pathway to enable access to romosozumab.

**Methods:** A patient co-developed, multidisciplinary team (including pharmacist, DXA, administrators) identified changes to the FLS pathway. To improve identification, fractures below the knee were excluded, and spine fracture detection enhanced by standardising referral from positive vertebral fracture assessments and Artificial Intelligence analyses of existing CT scans. To streamline assessment, patients without a history of cardiovascular disease were automatically referred for DXA where they completed a DXA questionnaire that included QRISK3. If eligible by DXA, patients were sent lab tests (including HbA1c, non-fasting lipids) and the Royal Osteoporosis Society romosozumab leaflet before their FLS Nurse telemed assessment. Eligible cases were then reviewed in weekly multidisciplinary team (MDT) meeting with the clinical lead and treatment was initiated using homecare providers.

**Results:** From 01/2024 to 09/2024, the FLS identified a total of 1705 postmenopausal women with newly diagnosed fractures, including hip (n=361) , spine (n=376), humerus (n=193) or wrist (n=1232). After clinical assessment, romosozumab was recommended to 61 patients (figure1). The mean patient age was 71.2 years (SD 8.4). The median time from first fracture to treatment recommendation was 147 (IQR 104-194) days and from fracture to romosozumab initiation was 170 (IQR 119-209) days.

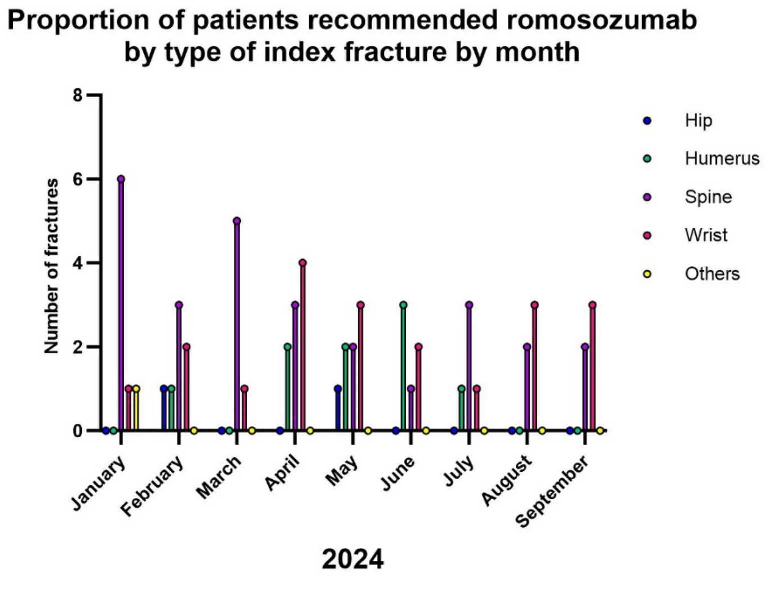

**Conclusion:** Integrating romosozumab into the FLS setting required substantial pathway redesign for patient identification, assessment, treatment recommendation and monitoring. Further work is required to assessment clinical and patient experience and satisfaction.

**ND-P029**

**A Case of Osteogenesis Imperfecta Type II and Recessive Alport Syndrome: unveiling novel COL1A2 and COL4A4 Variants in Prenatal Diagnosis**

Luca Celli[1], Marianxhela Dajko[1], Linda Maria Azzurra Pirollo[2], Norman Veccia[3], Alessandra Cacciavillani[4], Simone Bargiacchi[5], Luigi Laino[5], Paola Grammatico[5], Michele Valiante[5], Daniela Formicola[5]
[1]Sapienza University of Rome, Department of Experimental Medicine, Rome, Italy
[2]Azienda Ospedaliera San Camillo Forlanini, Gynecology and Obstetric Department, Rome, Italy
[3]Azienda Ospedaliera San Camillo Forlanini, Department of Pathology, Rome, Italy
[4]Azienda Ospedaliera San Camillo Forlanini, General Radiology Department, Rome, Italy
[5]Azienda Ospedaliera San Camillo Forlanini, Laboratory of Medical Genetics Unit, Rome, Italy

**Abstract Text**

**Background:** Skeletal dysplasias encompass an heterogenic group of genetic disorders affecting bone development, often with severe phenotypes, which may be detectable prenatally by ultrasound. To date, whole-exome sequencing (WES) is increasingly used when cytogenetic analysis are not diagnostic for fetal malformations thus providing a tool to optimize clinical management and future reproductive counseling.

**Purpose:** Investigate the morphological and genetic characteristics of a fetus with ultrasound bone anomalies in an Egyptian first cousins couple.

**Methods:** A couple with two healthy daughters and three spontaneous miscarriages was referred to our hospital during the sixth pregnancy followed up with routine prenatal imaging exams. Skeletal deformities at ultrasound morphological examination was found (shortening <3° percentile and bowing of the femurs, slight reduction in the length of other long bones, suggesting skeletal dysplasia). A trio-WES with amniocentesis was performed.

**Results:** WES sequencing unexpectedly revealed a de novo new heterozygous missense variant in the COL1A2 gene: NM_000089.4:c.1720G>T (p.Gly574Cys). Bioinformatic analysis showed that p. Gly574, was deleterious and may have an impact on protein function (Revel = 0.986, dbscSNV ADA = 1.00, dbscSNV RF = 0.95). This variant is in highly conserved glycine residues of Gly-X-Y sequence repeats of the collagen type I alpha chain in the first base of exon 30 probably causing OI type II. Also WES identified two homozygous variants in COL4A4 (c.2320G>C, p.Gly774Arg and c.4394G>A, p.Gly1465Asp) described in literature as complex alleles associated with Alport syndrome and basement membrane disease. Parents were found to be heterozygous for both variants in cis. Couple opted for pregnancy termination.

**Conclusion:** This study expands the genetic spectrum of OI type II and Alport syndrome, identifying a novel pathogenic COL1A2 variant and complex COL4A4 alleles combination. This findings emphasize the importance of prenatal WES for precise diagnosis, inheritance determination and reproductive decision-making.
